# Supplementary figures and images for: Compensatory sequence variation between trans-species small RNAs and their target sites
Source: eLife. 2019 Dec 17;8:e49750. doi: 10.7554/eLife.49750 (PMC6917502; doi:10.7554/eLife.49750)

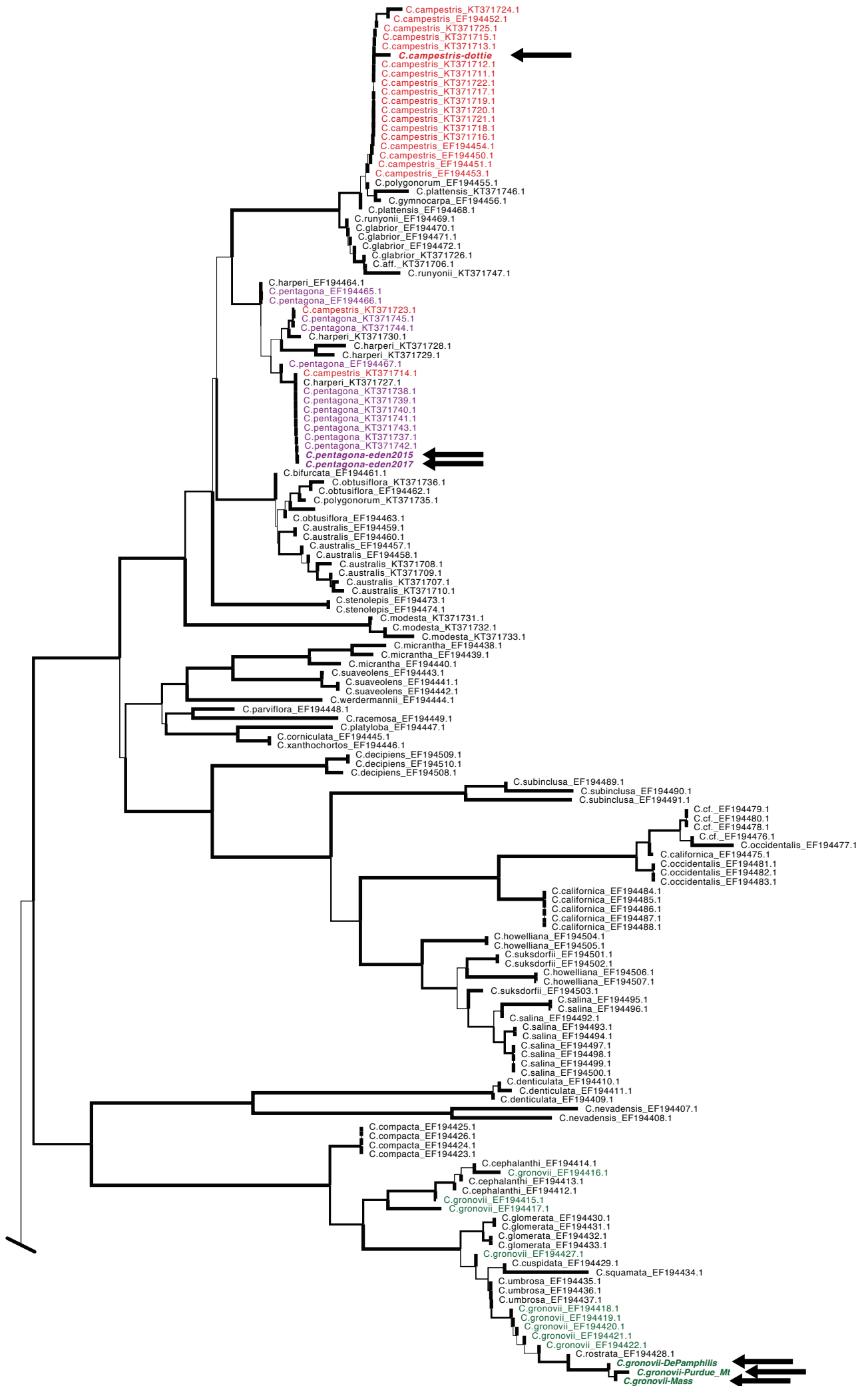

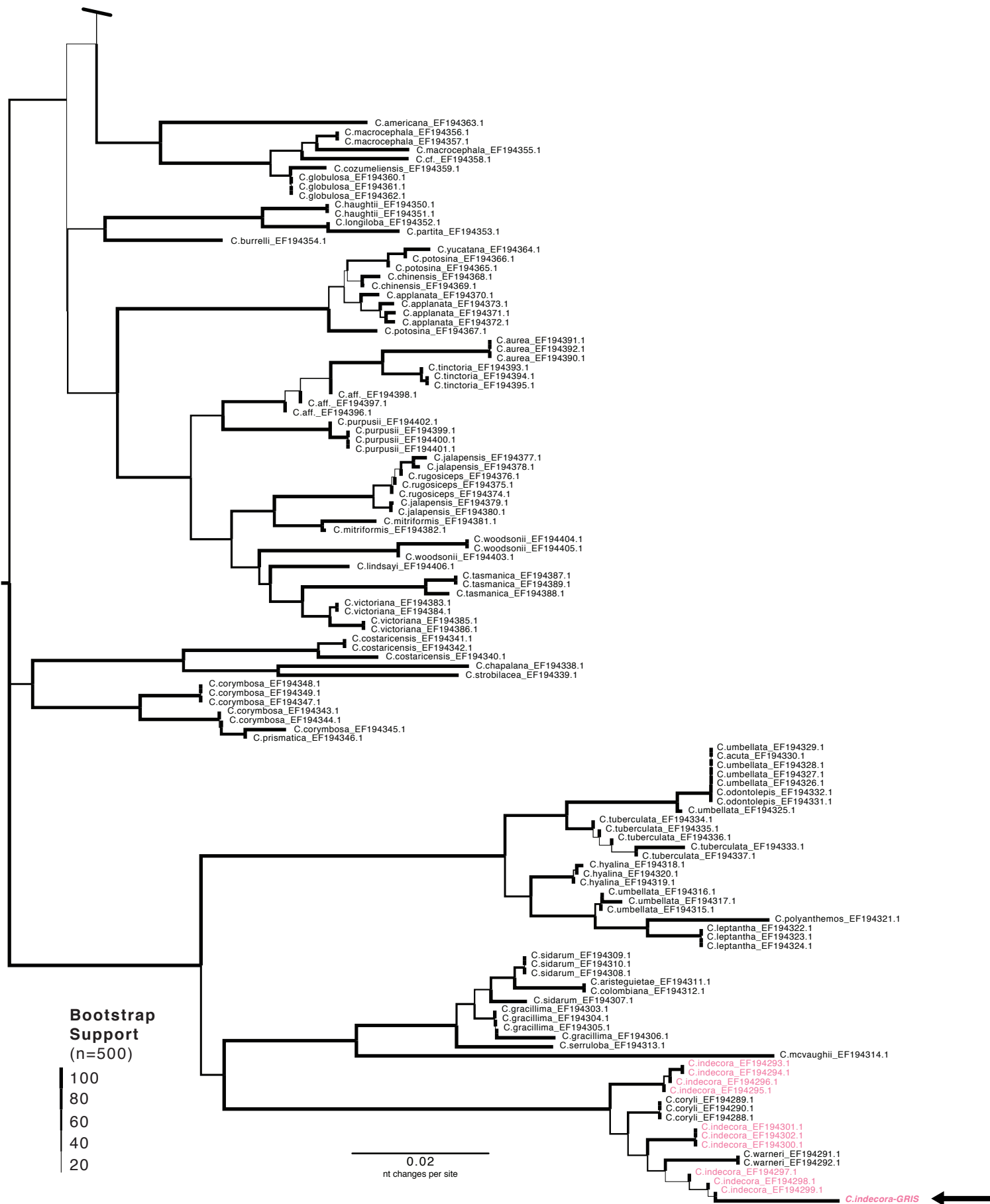

Supplement: Supplementary file 1. — Isolates used in this study are in bold and indicated with arrows. Samples identified as members of species examined in this study are highlighted with color; red - C. campestris, purple - C. pentagona, green - C. gronovii, pink - C. indecora. Format: PDF [file elife-49750-supp1.pdf]
