# Supplementary material for: Compensatory sequence variation between trans-species small RNAs and their target sites
Source: eLife. 2019 Dec 17;8:e49750. doi: 10.7554/eLife.49750 (PMC6917502; doi:10.7554/eLife.49750)
Supplement: Supplementary file 5. — Predicted RNA secondary structures and expression profiles of loci that produce HI-sRNAs and have an apparent miRNA hairpin. Format: PDF [file elife-49750-supp5.pdf]

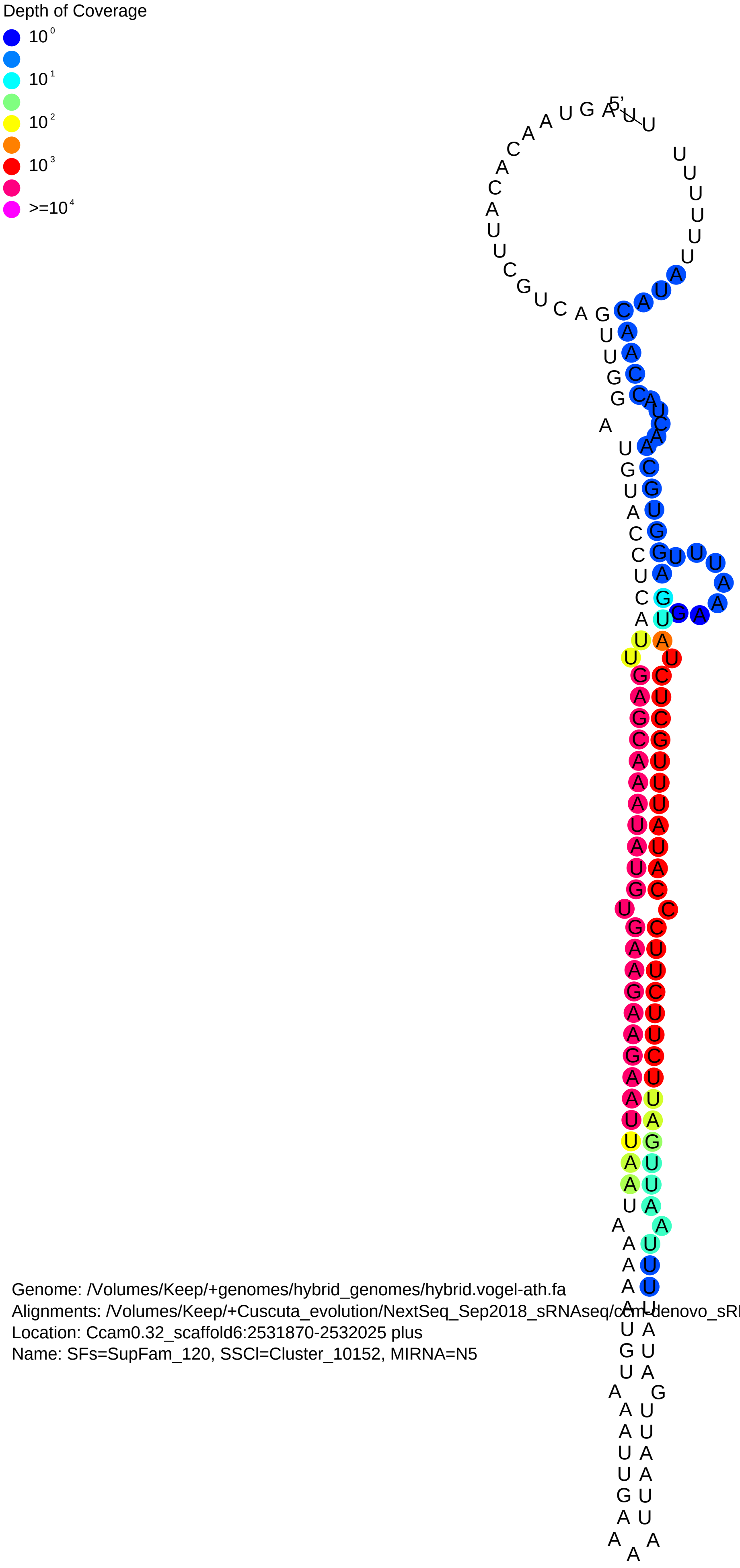

Genome: /Volumes/Keep/+genomes/hybrid_genomes/hybrid.vogel-ath.fa
Alignments: /Volumes/Keep/+Cuscuta_evolution/NextSeq_Sep2018_sRNAseq/ccm-denovo_sRNA_annotation/02out-cca.hybrid.ShortStack/merged_alig
Location: Ccam0.32_scaffold6:2531870-2532025 plus
Name: SFs=SupFam_120, SSCl=Cluster_10152, MIRNA=N5

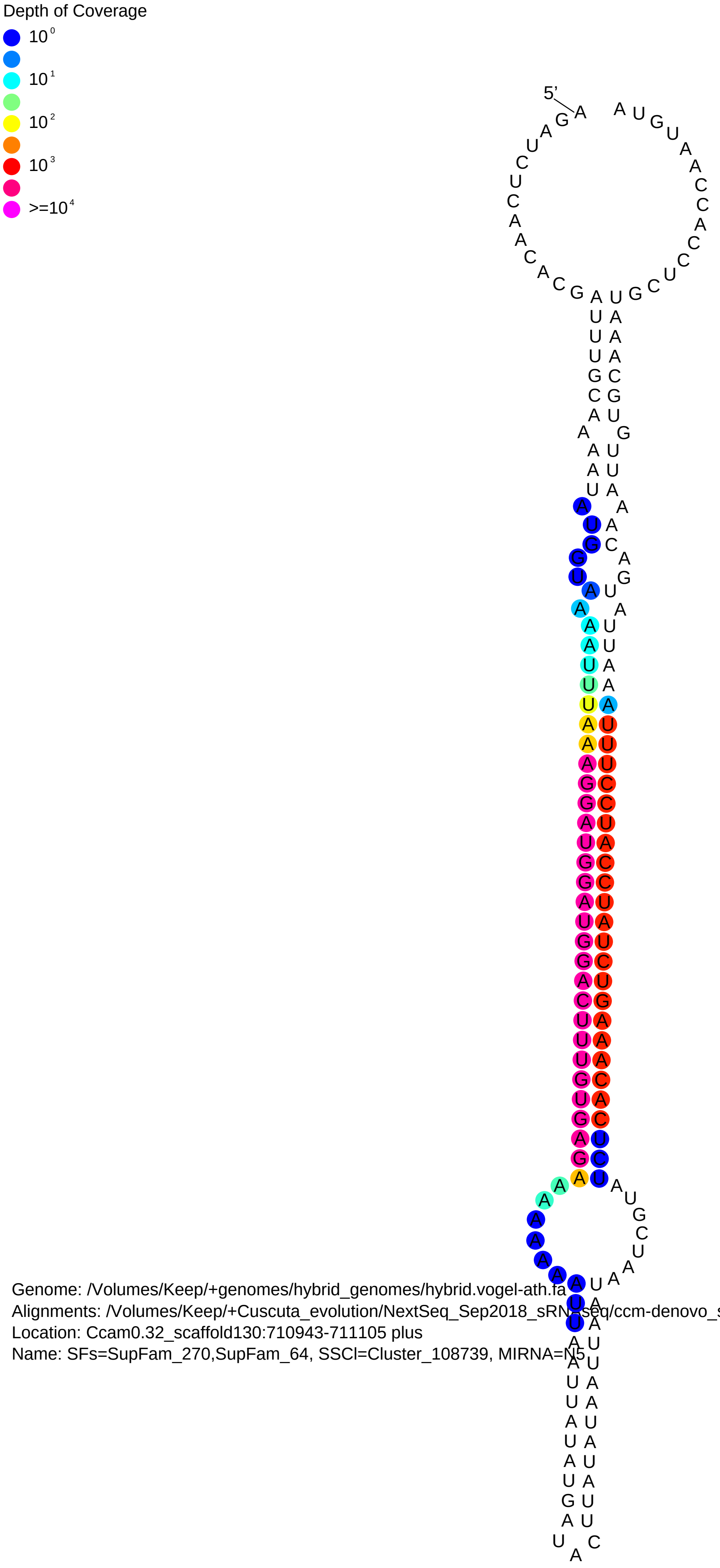

Genome: /Volumes/Keep/+genomes/hybrid_genomes/hybrid.vogel-ath.fa
Alignments: /Volumes/Keep/+Cuscuta_evolution/NextSeq_Sep2018_sRN[illegible]seq/ccm-denovo_sRNA_annotation/02out-cca.hybrid.ShortStack/merged_alig
Location: Ccam0.32_scaffold130:710943-711105 plus
Name: SFs=SupFam_270,SupFam_64, SSCl=Cluster_108739, MIRNA=N[illegible]

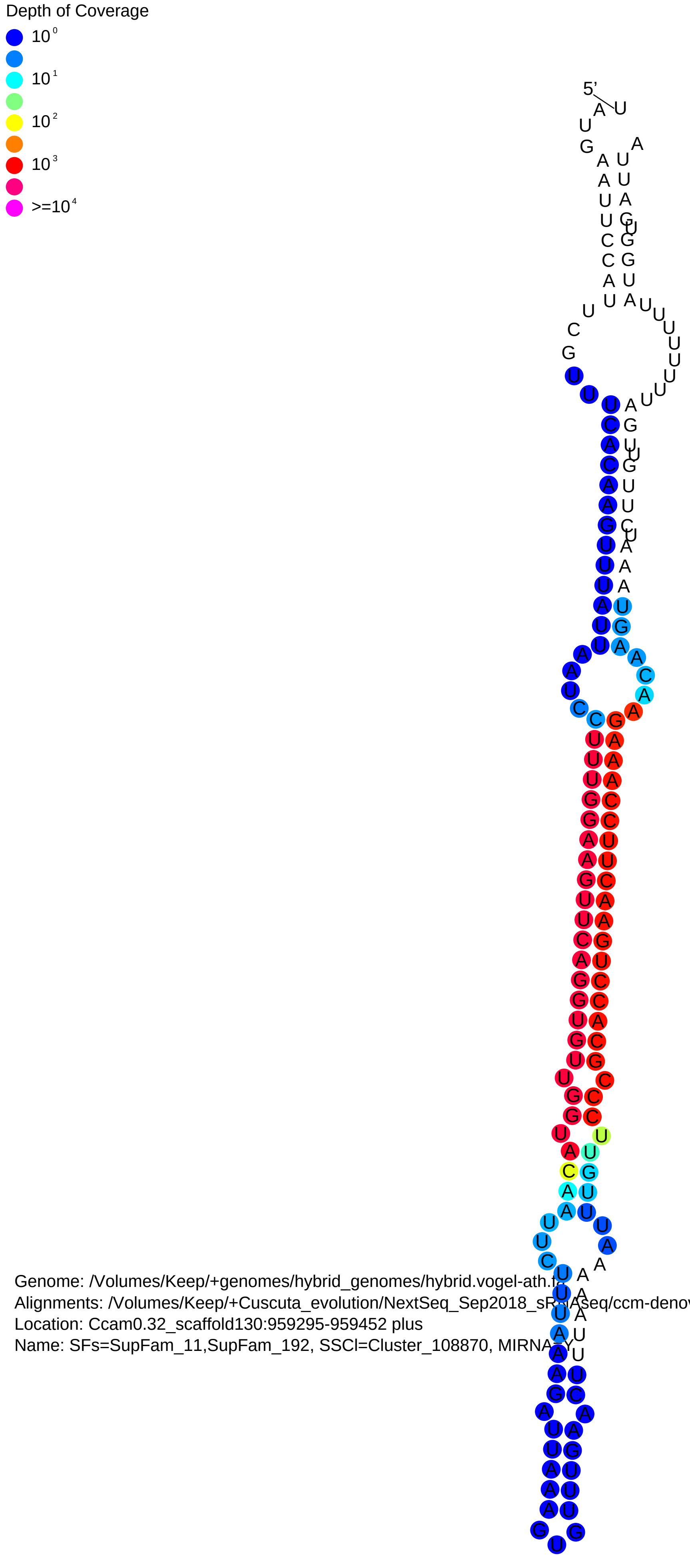

Genome: /Volumes/Keep/+genomes/hybrid_genomes/hybrid.vogel-ath.f[illegible]
Alignments: /Volumes/Keep/+Cuscuta_evolution/NextSeq_Sep2018_sR[illegible]Aseq/ccm-denovo_sRNA_annotation/02out-cca.hybrid.ShortStack/merged_alig
Location: Ccam0.32_scaffold130:959295-959452 plus
Name: SFs=SupFam_11,SupFam_192, SSCl=Cluster_108870, MIRNA=Y

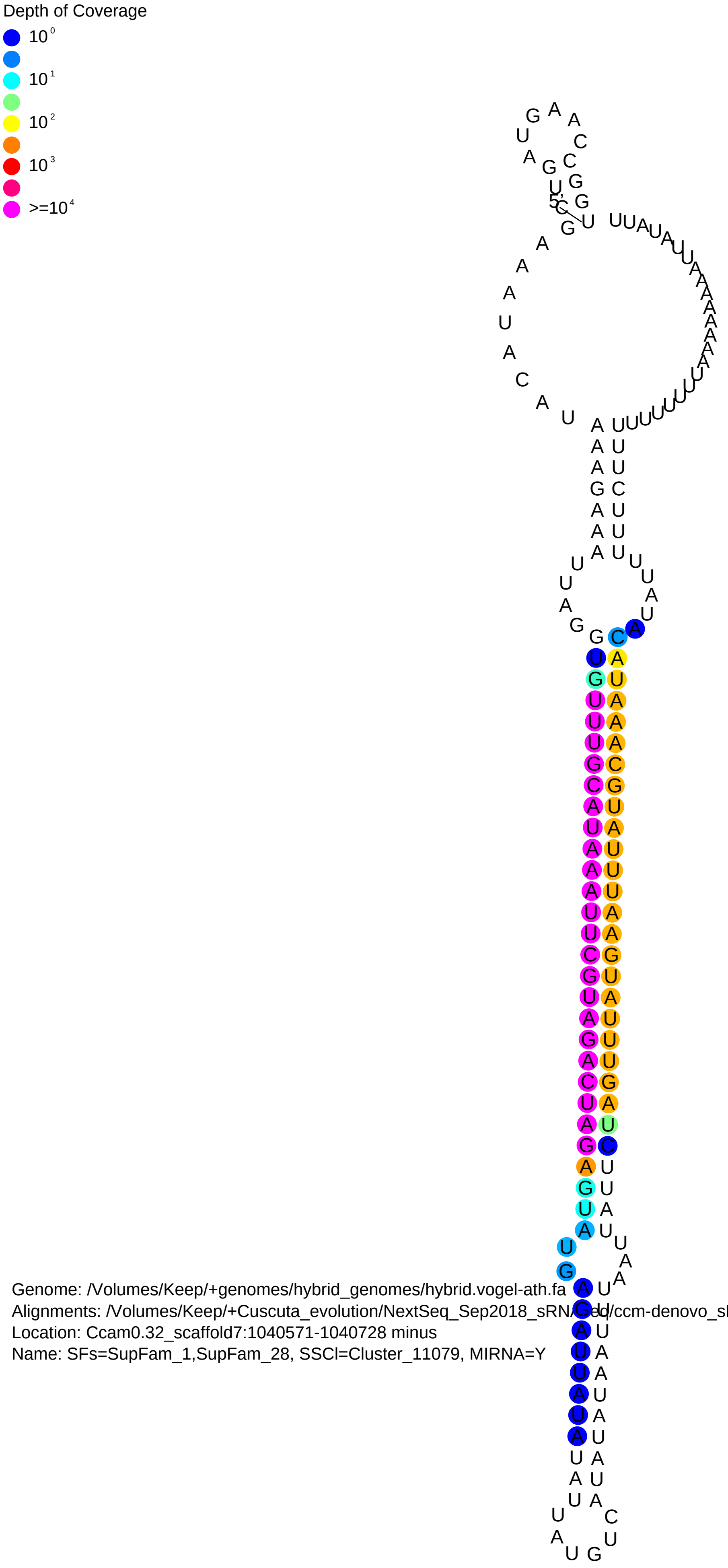

Genome: /Volumes/Keep/+genomes/hybrid_genomes/hybrid.vogel-ath.fa
Alignments: /Volumes/Keep/+Cuscuta_evolution/NextSeq_Sep2018_sRN[illegible]q/ccm-denovo_sRNA_annotation/02out-cca.hybrid.ShortStack/merged_alig
Location: Ccam0.32_scaffold7:1040571-1040728 minus
Name: SFs=SupFam_1,SupFam_28, SSCl=Cluster_11079, MIRNA=Y

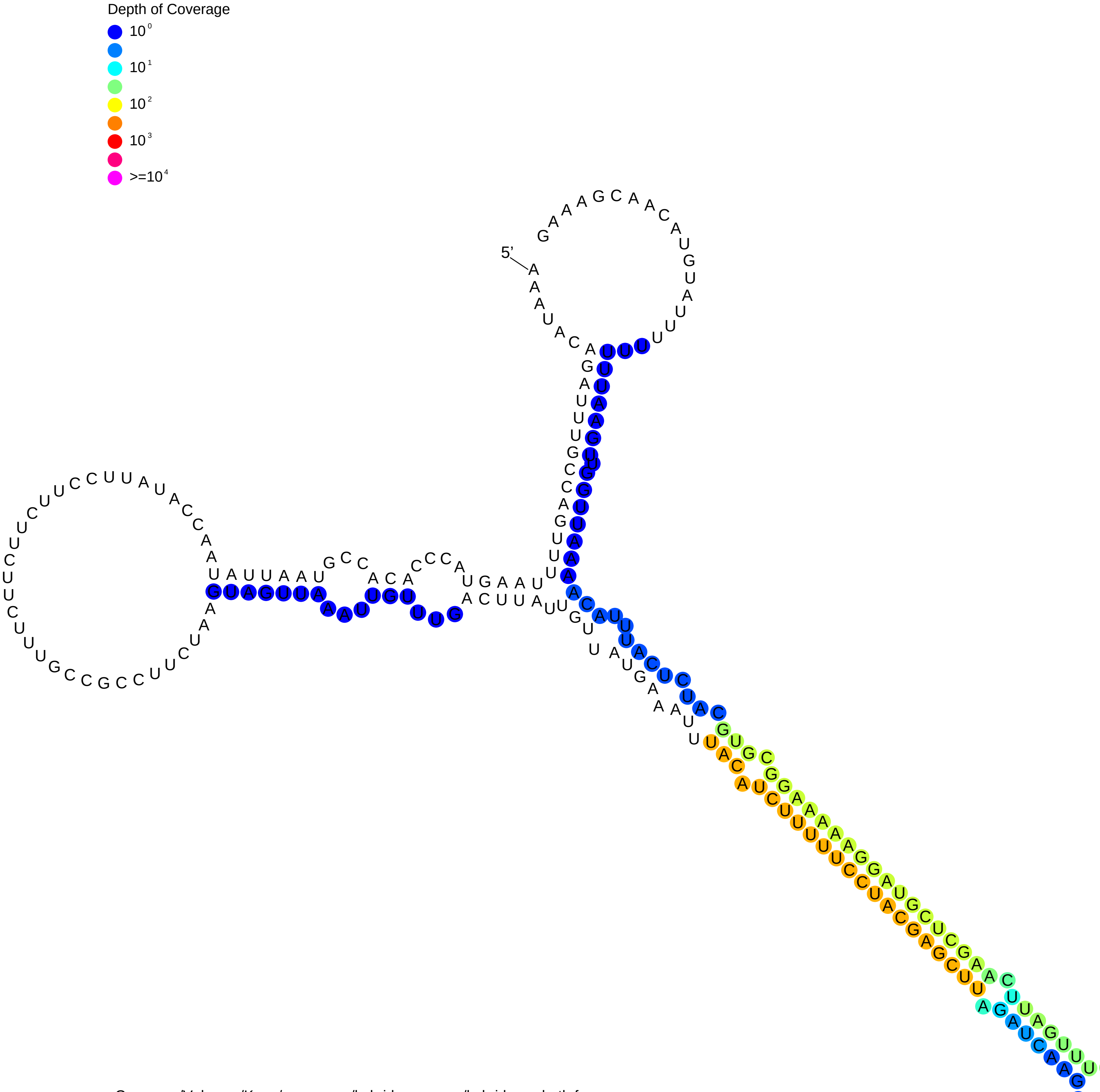

Genome: /Volumes/Keep/+genomes/hybrid_genomes/hybrid.vogel-ath.fa
Alignments: /Volumes/Keep/+Cuscuta_evolution/NextSeq_Sep2018_sRNAseq/ccm-denovo_sRNA_annotation/02out-cca.hybrid.ShortStack/merged_a[illegible]
Location: Ccam0.32_scaffold7:1043792-1044034 minus
Name: SFs=SupFam_305, SSCl=Cluster_11081, MIRNA=Y

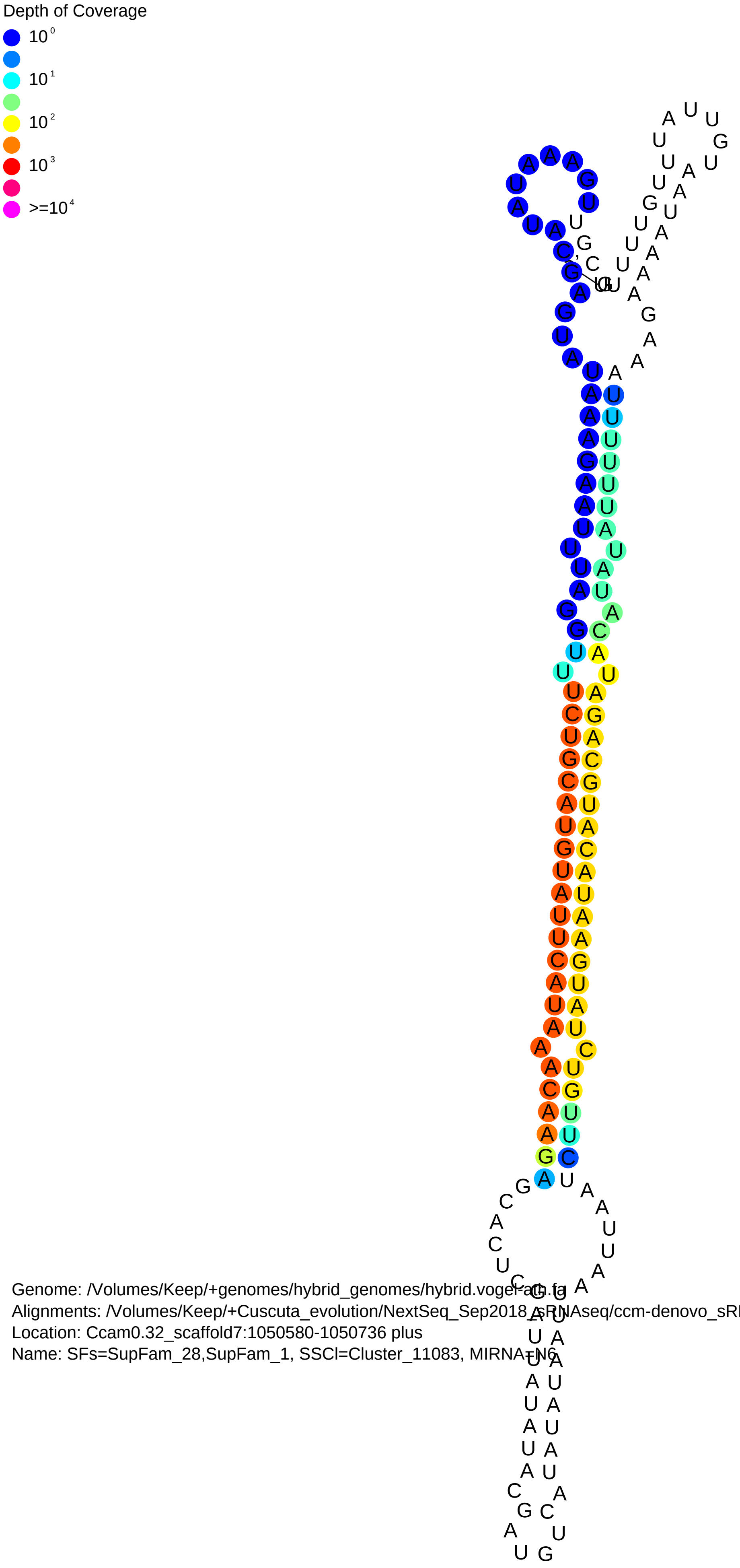

Genome: /Volumes/Keep/+genomes/hybrid_genomes/hybrid.vogel-cch.fa
Alignments: /Volumes/Keep/+Cuscuta_evolution/NextSeq_Sep2018_sRNAseq/ccm-denovo_sRNA_annotation/02out-cca.hybrid.ShortStack/merged_alig
Location: Ccam0.32_scaffold7:1050580-1050736 plus
Name: SFs=SupFam_28,SupFam_1, SSCl=Cluster_11083, MIRNA=N6

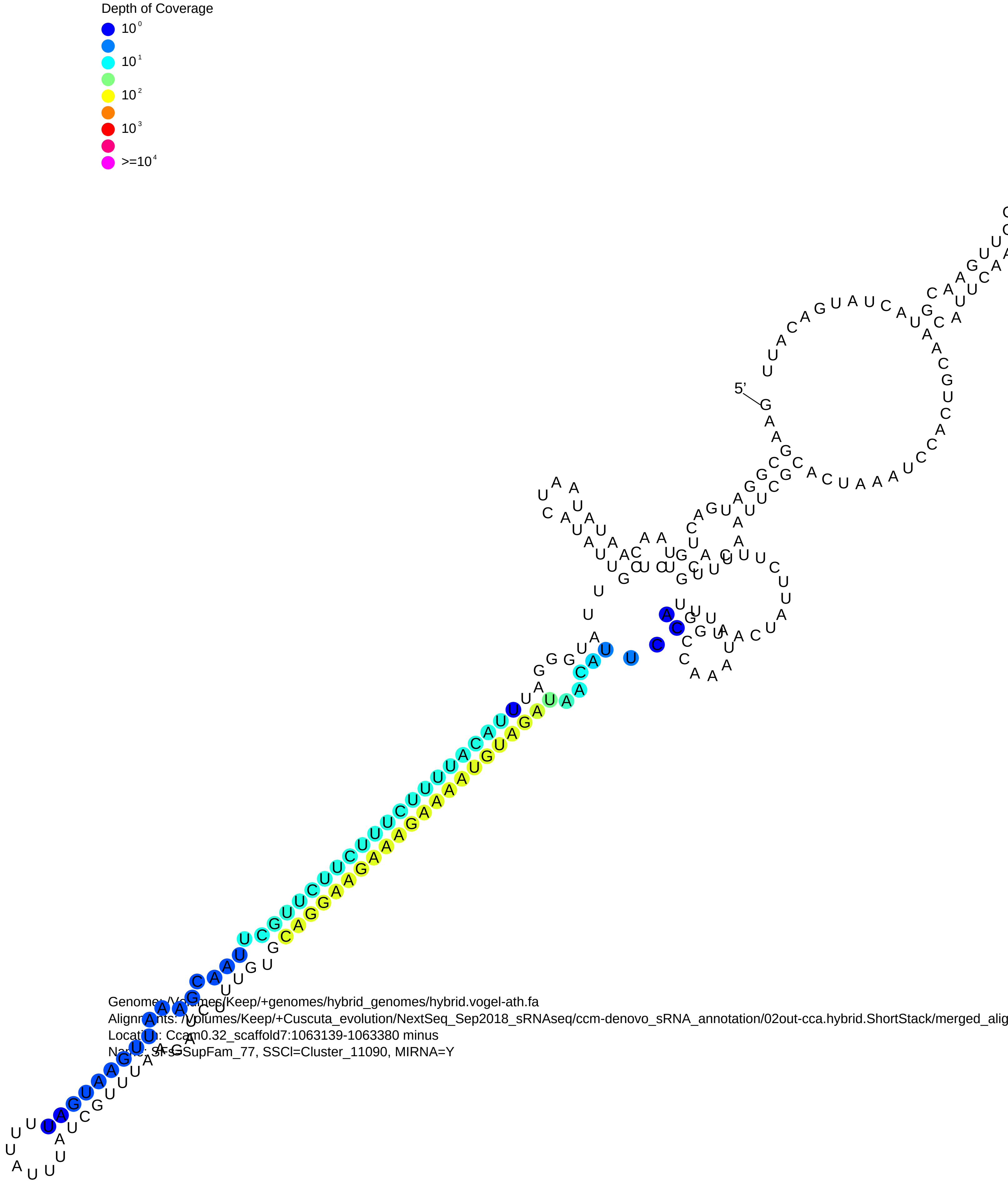

Genome: /Volumes/Keep/+genomes/hybrid_genomes/hybrid.vogel-ath.fa
Alignments: /Volumes/Keep/+Cuscuta_evolution/NextSeq_Sep2018_sRNAseq/ccm-denovo_sRNA_annotation/02out-cca.hybrid.ShortStack/merged_alig
Location: Ccam0.32_scaffold7:1063139-1063380 minus
Name: SFs=SupFam_77, SSCl=Cluster_11090, MIRNA=Y

Depth of Coverage

- 10^0
-
- 10^1
-
- 10^2
-
- 10^3
-
- $>=10^4$

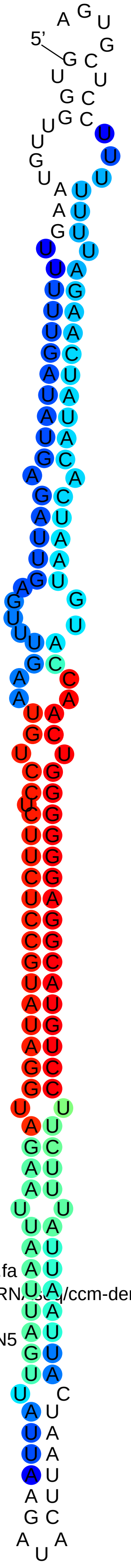

Genome: /Volumes/Keep/+genomes/hybrid_genomes/hybrid.vogel-ath.fa

Alignments: /Volumes/Keep/+Cuscuta_evolution/NextSeq_Sep2018_sRN[illegible]/ccm-denovo_sRNA_annotation/02out-cca.hybrid.ShortStack/merged_alig

Location: Ccam0.32_scaffold136:450372-450528 minus

Name: SFs=SupFam_5,SupFam_40, SSCl=Cluster_111275, MIRNA=N5

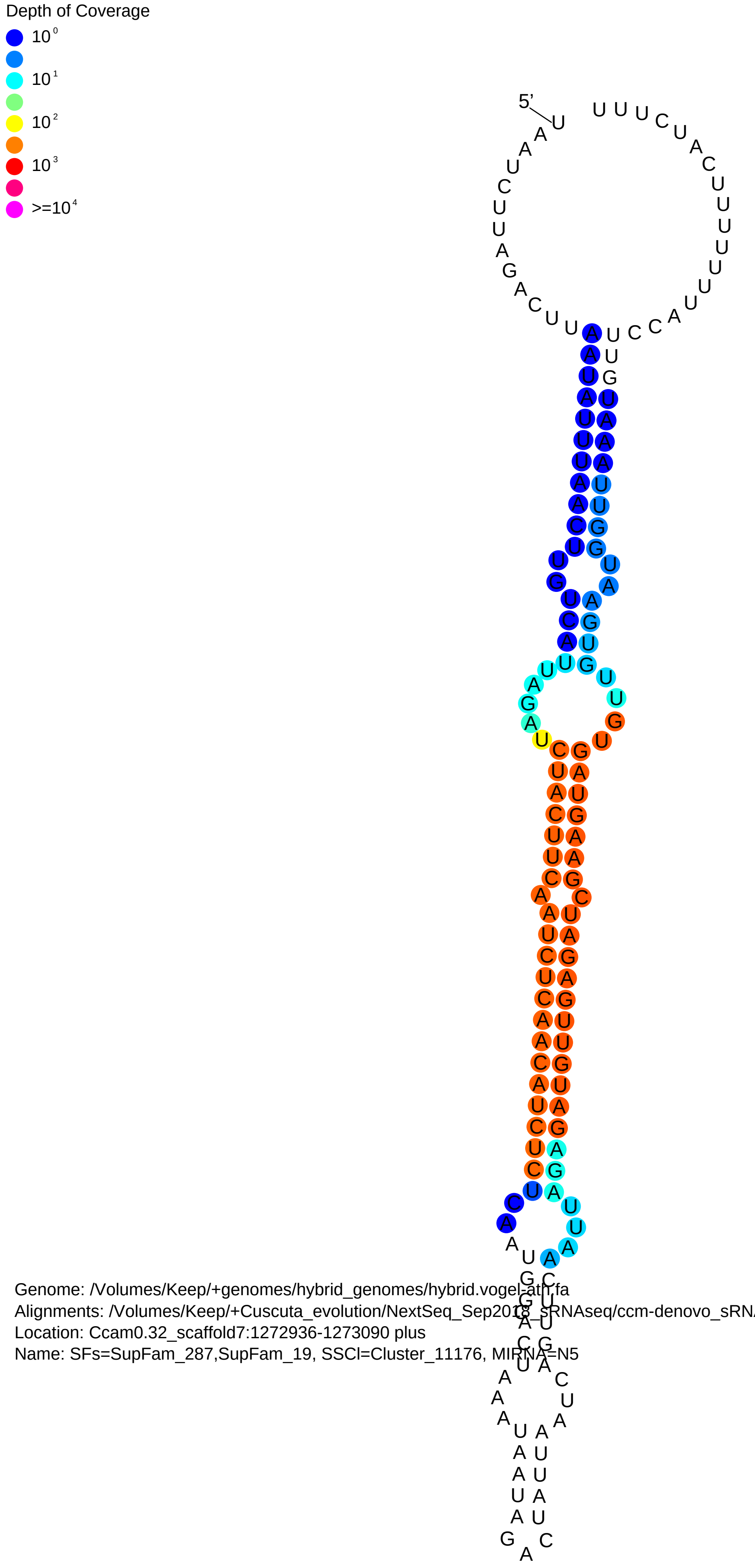

Genome: /Volumes/Keep/+genomes/hybrid_genomes/hybrid.vogel-ath.fa
Alignments: /Volumes/Keep/+Cuscuta_evolution/NextSeq_Sep2018_sRNAseq/ccm-denovo_sRNA_annotation/02out-cca.hybrid.ShortStack/merged_alig
Location: Ccam0.32_scaffold7:1272936-1273090 plus
Name: SFs=SupFam_287,SupFam_19, SSCl=Cluster_11176, MIRNA=N5

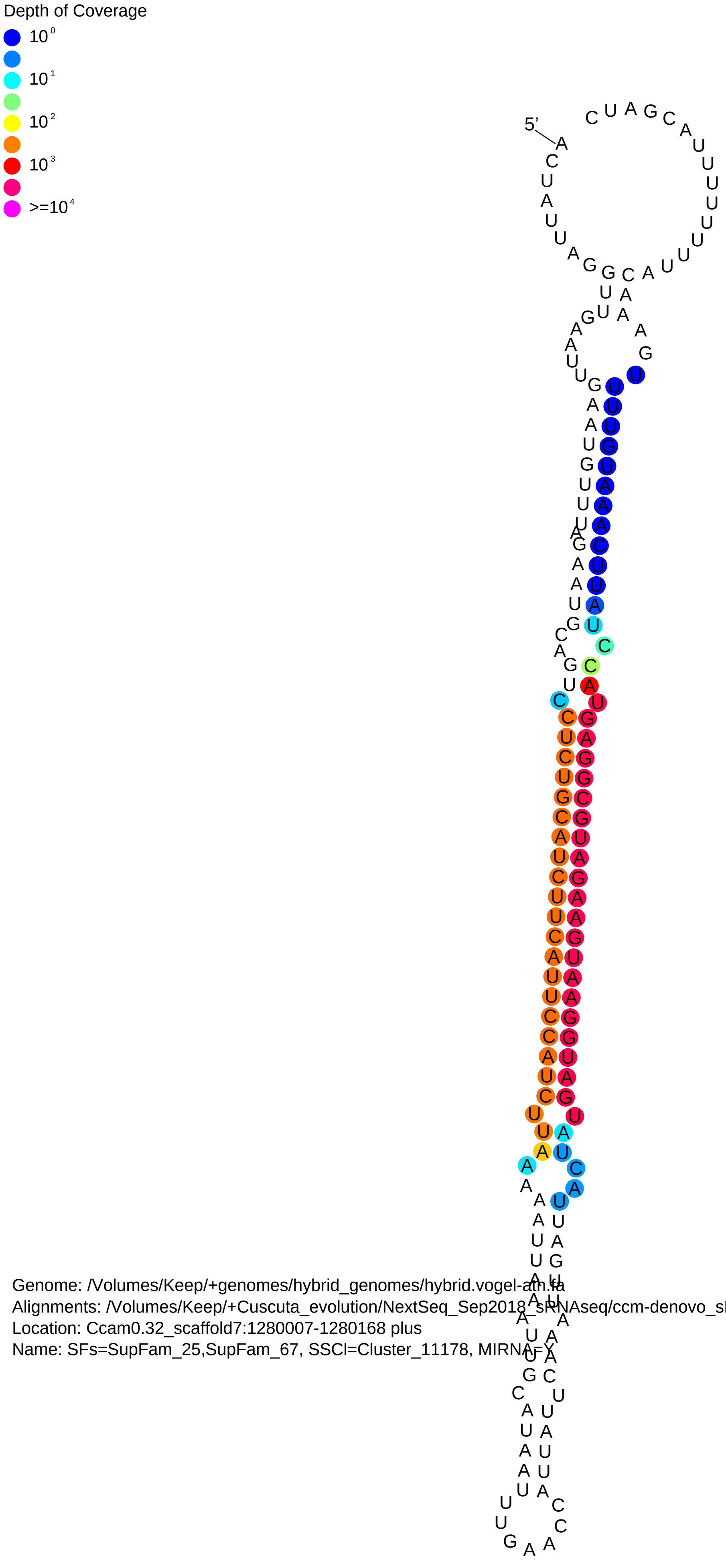

Genome: /Volumes/Keep/+genomes/hybrid_genomes/hybrid.vogel-ath.fa
Alignments: /Volumes/Keep/+Cuscuta_evolution/NextSeq_Sep2018_sRNAseq/ccm-denovo_sRNA_annotation/02out-cca.hybrid.ShortStack/merged_alig
Location: Ccam0.32_scaffold7:1280007-1280168 plus
Name: SFs=SupFam_25,SupFam_67, SSCl=Cluster_11178, MIRNA=Y

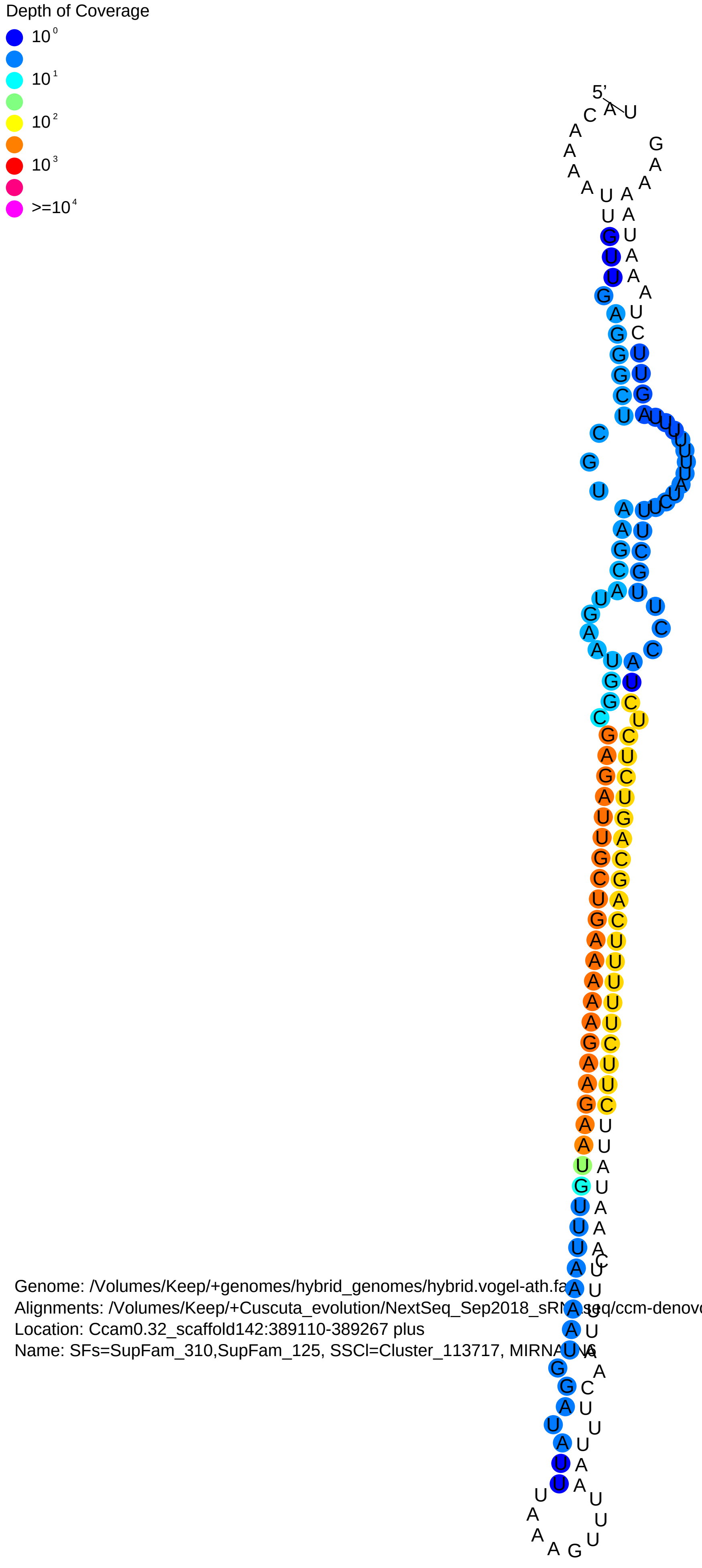

Genome: /Volumes/Keep/+genomes/hybrid_genomes/hybrid.vogel-ath.fa
Alignments: /Volumes/Keep/+Cuscuta_evolution/NextSeq_Sep2018_sRNAseq/ccm-denovo_sRNA_annotation/02out-cca.hybrid.ShortStack/merged_alig
Location: Ccam0.32_scaffold142:389110-389267 plus
Name: SFs=SupFam_310,SupFam_125, SSCl=Cluster_113717, MIRNA=N

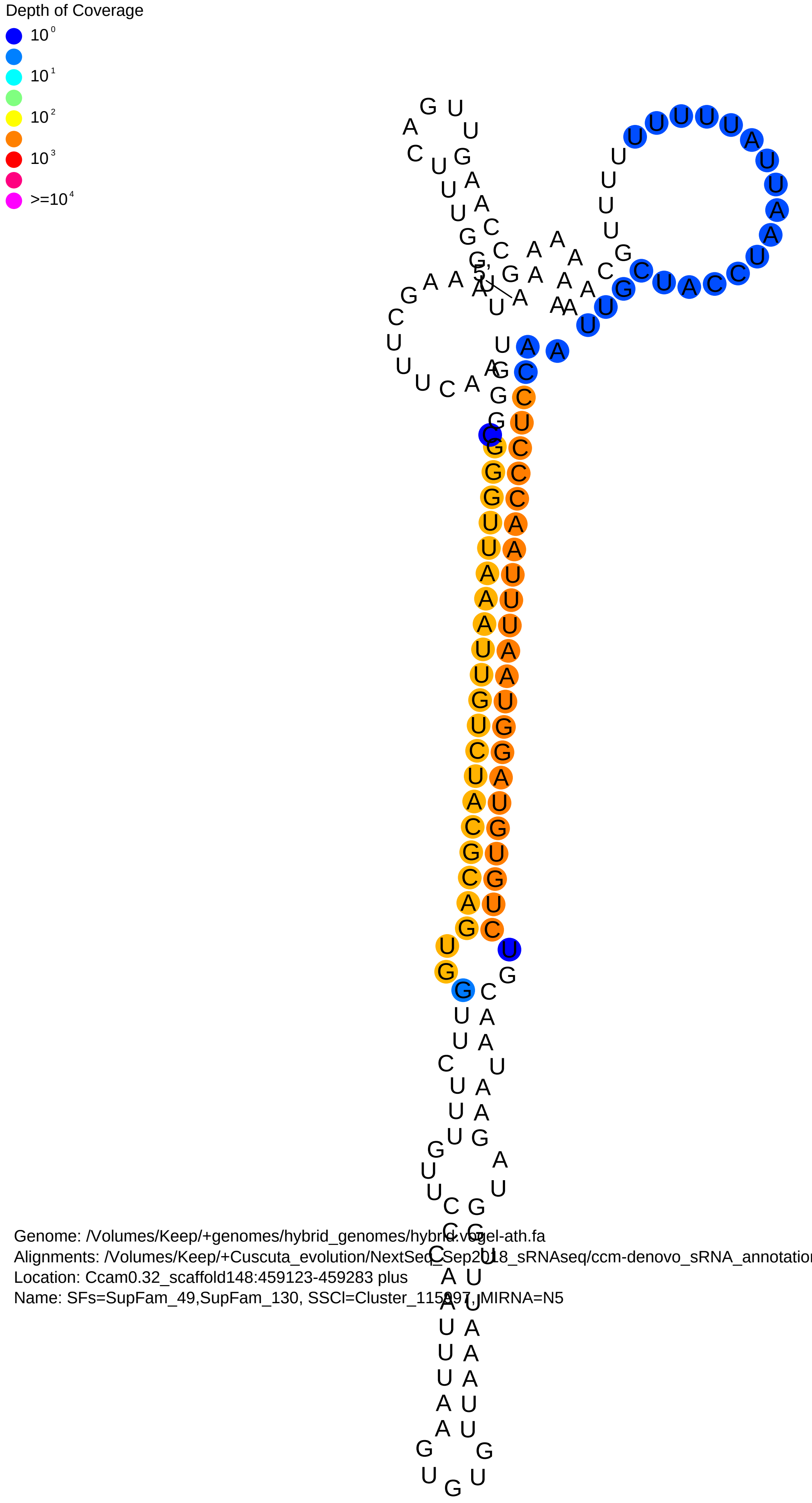

Genome: /Volumes/Keep/+genomes/hybrid_genomes/hybrid.vogel-ath.fa
Alignments: /Volumes/Keep/+Cuscuta_evolution/NextSeq_Sep2018_sRNAseq/ccm-denovo_sRNA_annotation/02out-cca.hybrid.ShortStack/merged_alig
Location: Ccam0.32_scaffold148:459123-459283 plus
Name: SFs=SupFam_49,SupFam_130, SSCl=Cluster_115997, MIRNA=N5

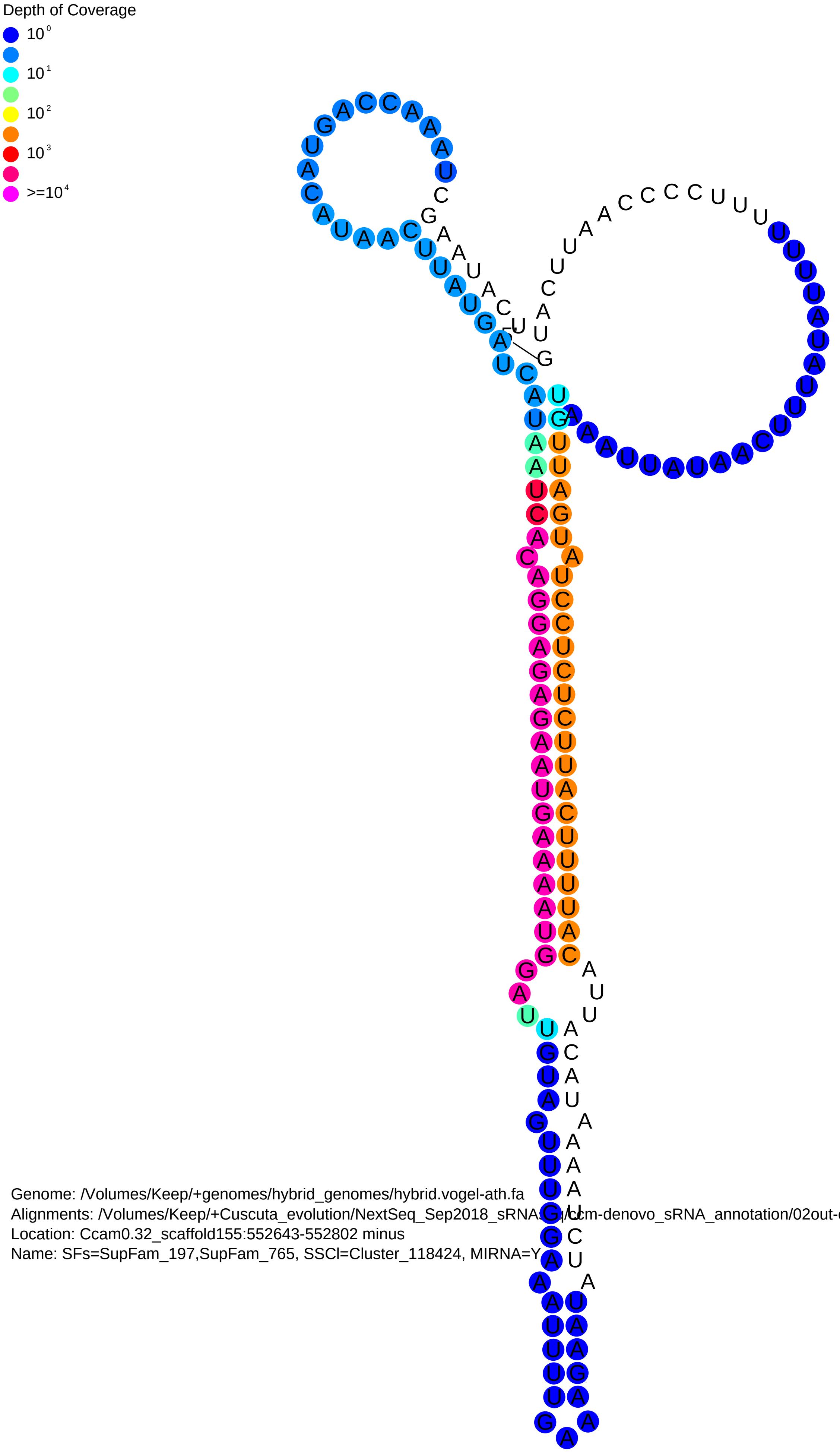

Genome: /Volumes/Keep/+genomes/hybrid_genomes/hybrid.vogel-ath.fa
Alignments: /Volumes/Keep/+Cuscuta_evolution/NextSeq_Sep2018_sRNA[illegible]/ccm-denovo_sRNA_annotation/02out-cca.hybrid.ShortStack/merged_alig
Location: Ccam0.32_scaffold155:552643-552802 minus
Name: SFs=SupFam_197,SupFam_765, SSCl=Cluster_118424, MIRNA=Y

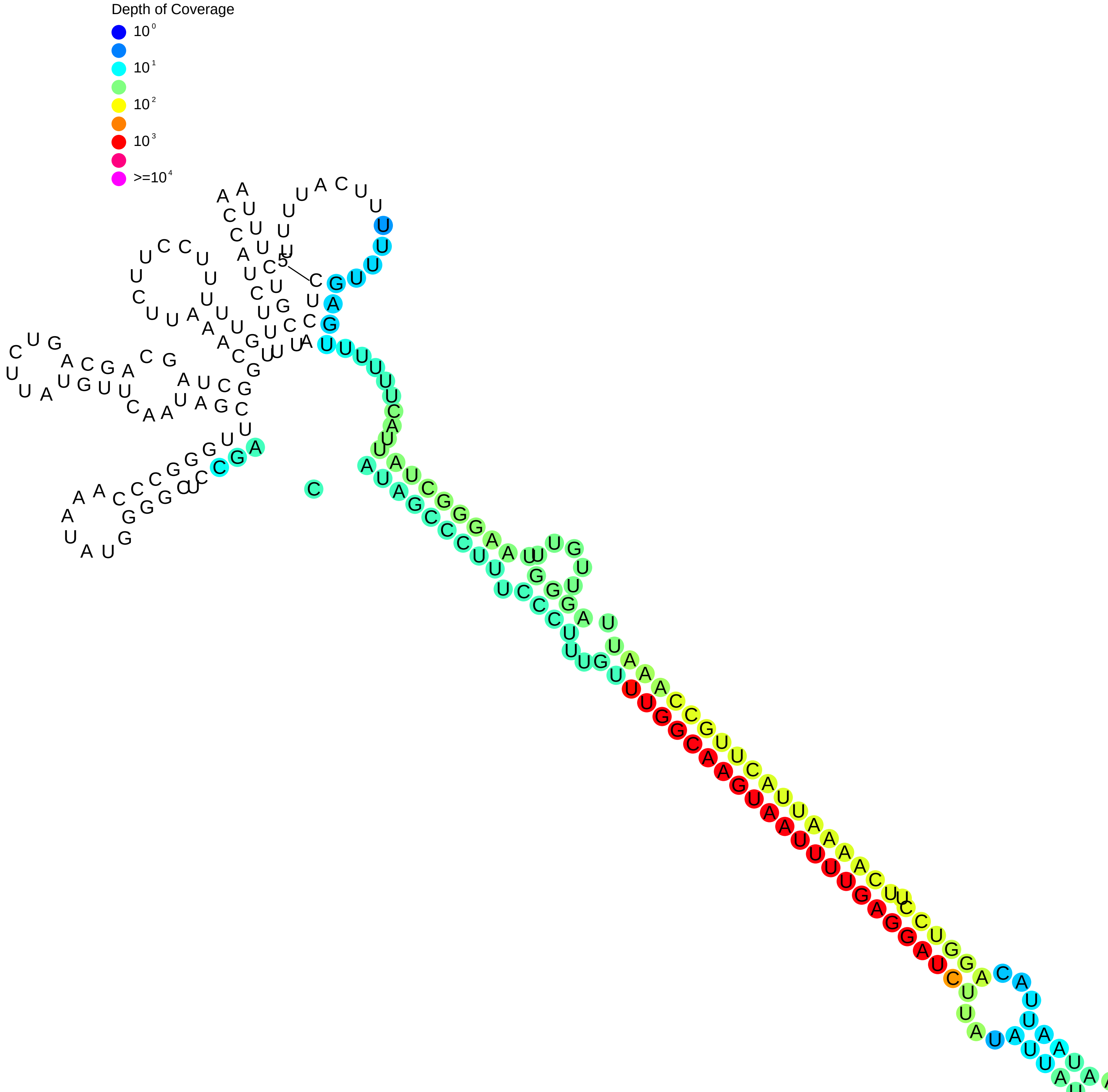

Genome: /Volumes/Keep/+genomes/hybrid_genomes/hybrid.vogel-ath.fa
Alignments: /Volumes/Keep/+Cuscuta_evolution/NextSeq_Sep2018_sRNAseq/ccm-denovo_sRNA_annotation/02out-cca.hybrid.ShortStack/merged_a
Location: Ccam0.32_scaffold157:653384-653625 minus
Name: SFs=SupFam_759, SSCl=Cluster_119071, MIRNA=N5

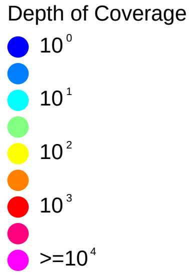

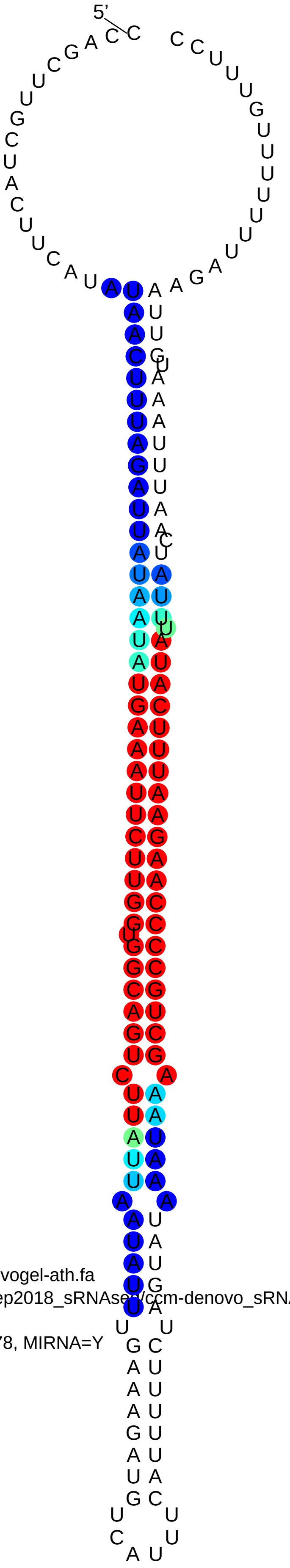

Genome: /Volumes/Keep/+genomes/hybrid_genomes/hybrid.vogel-ath.fa
Alignments: /Volumes/Keep/+Cuscuta_evolution/NextSeq_Sep2018_sRNAseq/ccm-denovo_sRNA_annotation/02out-cca.hybrid.ShortStack/merged_alig
Location: Ccam0.32_scaffold161a:621305-621462 minus
Name: SFs=SupFam_26,SupFam_123, SSCl=Cluster_120378, MIRNA=Y

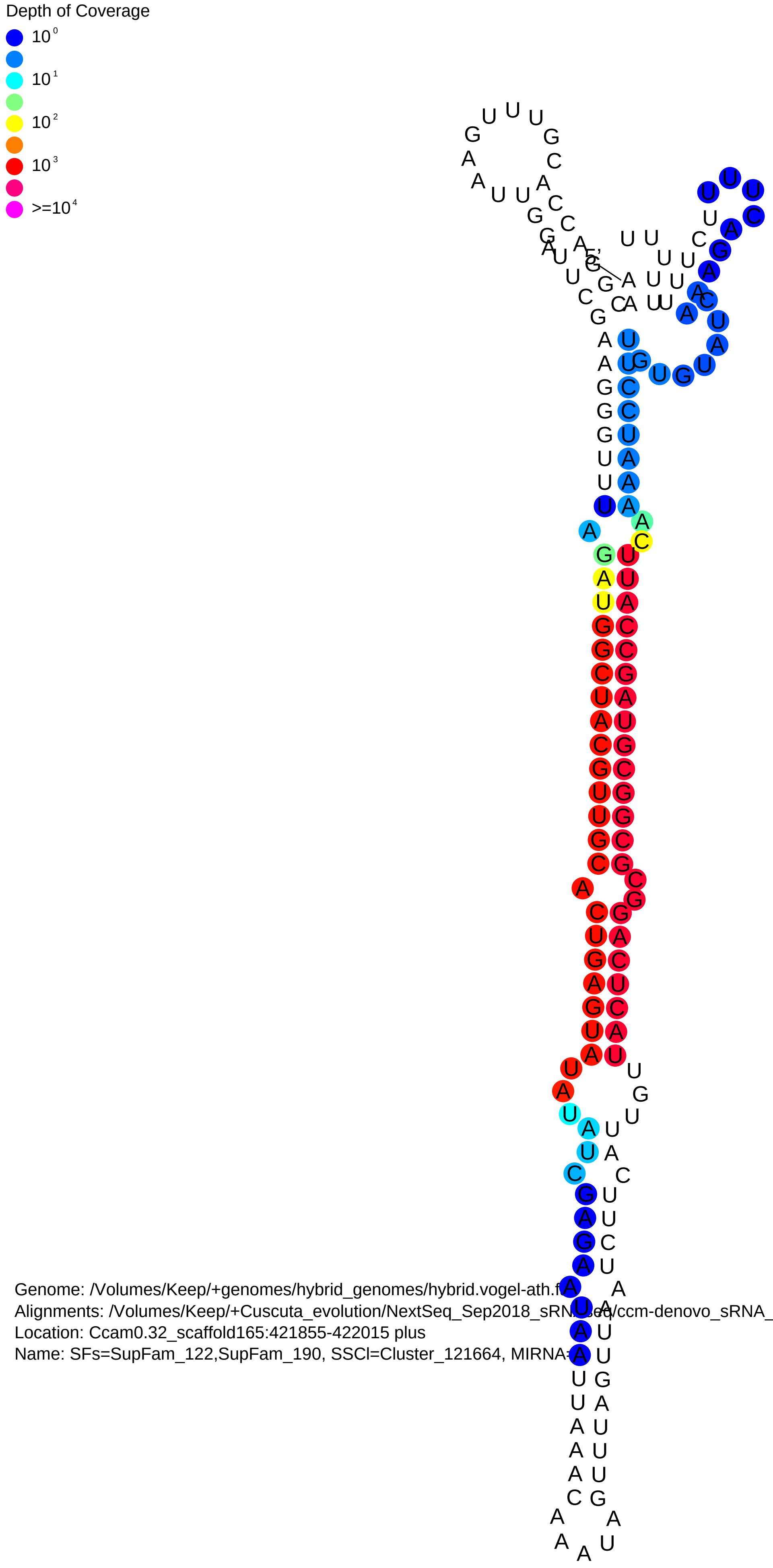

Genome: /Volumes/Keep/+genomes/hybrid_genomes/hybrid.vogel-ath.f[illegible]
Alignments: /Volumes/Keep/+Cuscuta_evolution/NextSeq_Sep2018_sRN[illegible]eq/ccm-denovo_sRNA_annotation/02out-cca.hybrid.ShortStack/merged_alig
Location: Ccam0.32_scaffold165:421855-422015 plus
Name: SFs=SupFam_122,SupFam_190, SSCl=Cluster_121664, MIRNA=[illegible]

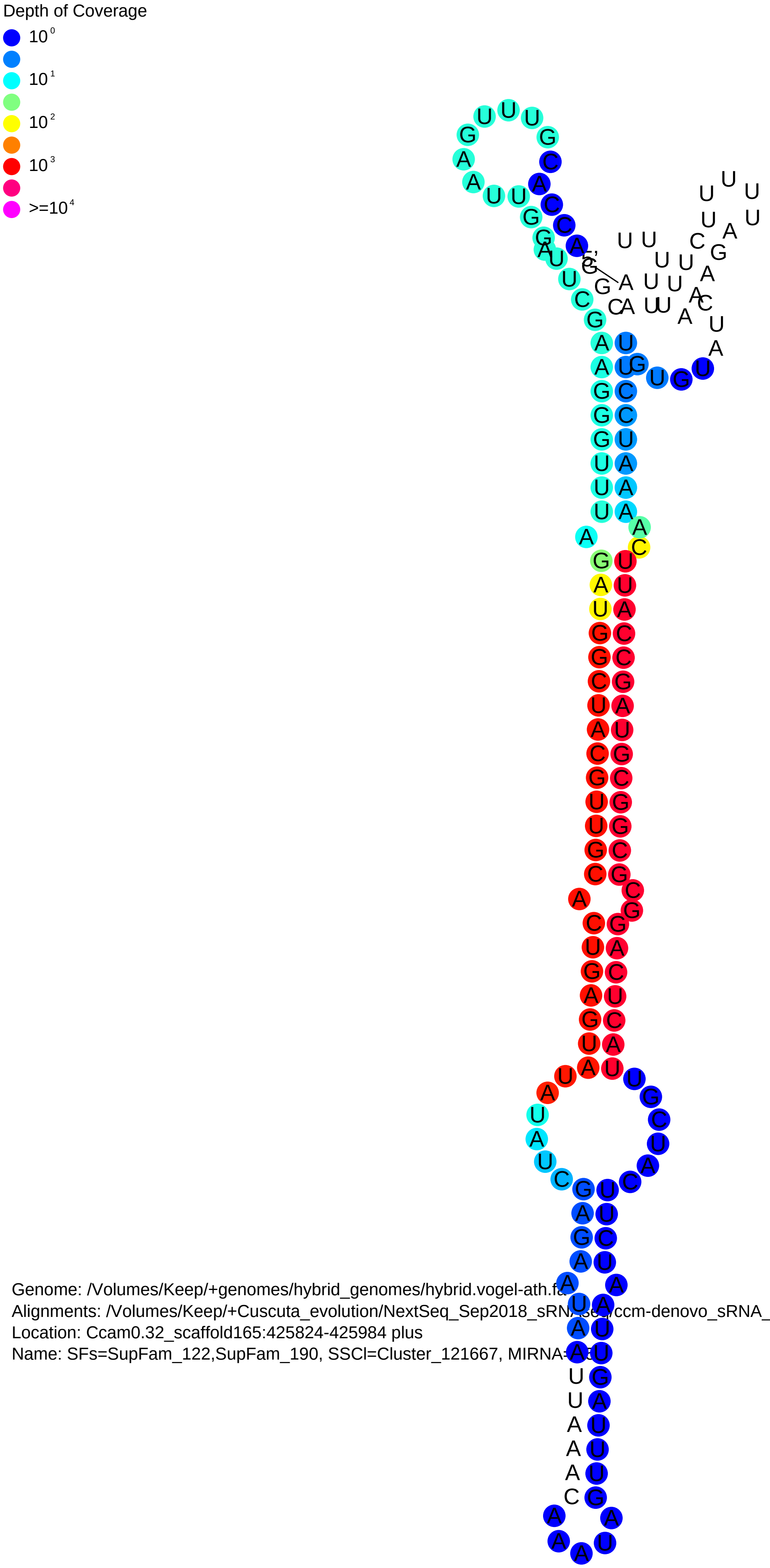

Genome: /Volumes/Keep/+genomes/hybrid_genomes/hybrid.vogel-ath.fa
Alignments: /Volumes/Keep/+Cuscuta_evolution/NextSeq_Sep2018_sRN[illegible]ccm-denovo_sRNA_annotation/02out-cca.hybrid.ShortStack/merged_alig
Location: Ccam0.32_scaffold165:425824-425984 plus
Name: SFs=SupFam_122,SupFam_190, SSCl=Cluster_121667, MIRNA=[illegible]

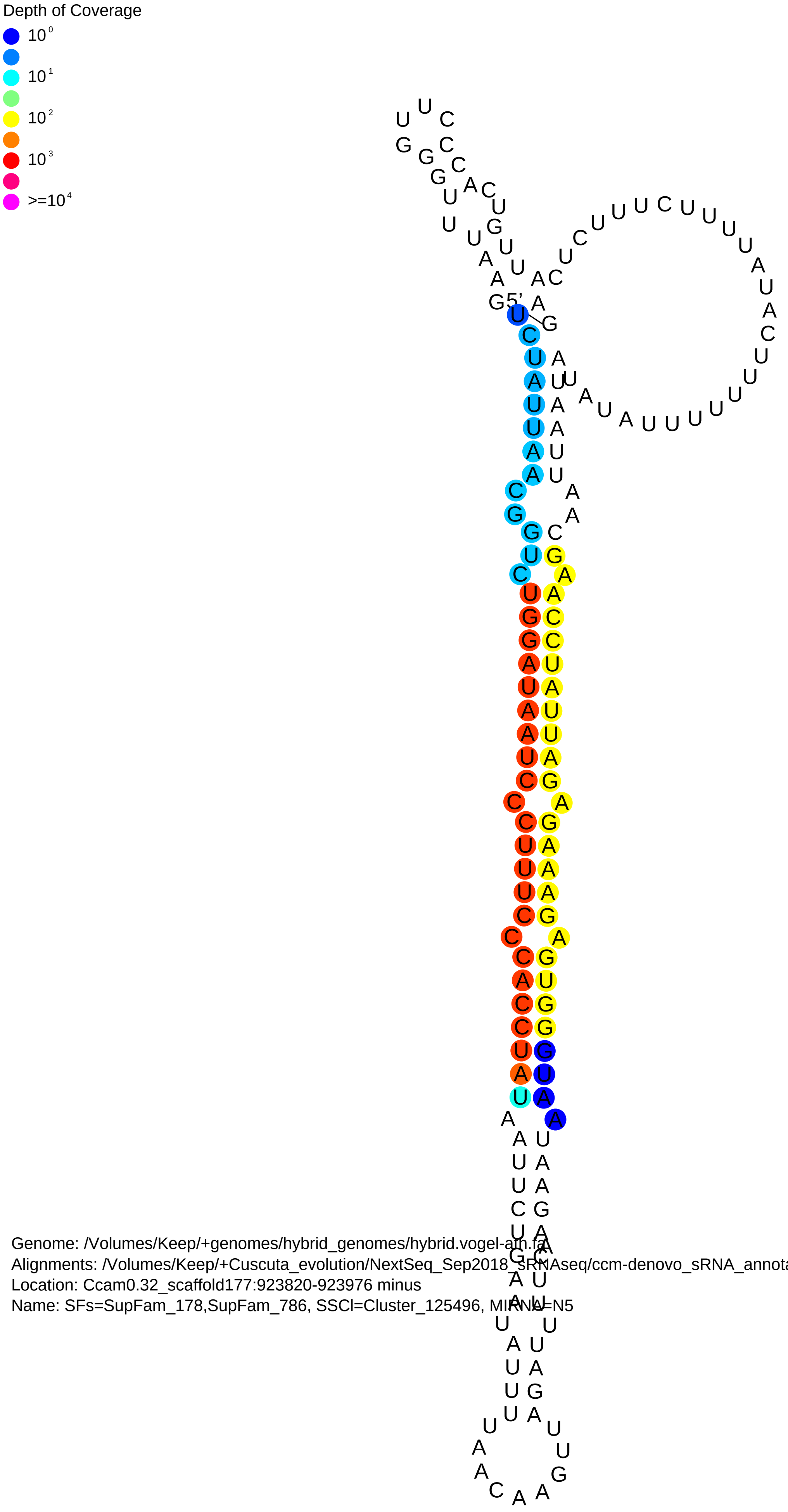

Genome: /Volumes/Keep/+genomes/hybrid_genomes/hybrid.vogel-ath.fa
Alignments: /Volumes/Keep/+Cuscuta_evolution/NextSeq_Sep2018_sRNAseq/ccm-denovo_sRNA_annotation/02out-cca.hybrid.ShortStack/merged_alig
Location: Ccam0.32_scaffold177:923820-923976 minus
Name: SFs=SupFam_178,SupFam_786, SSCl=Cluster_125496, MIRNA=N5

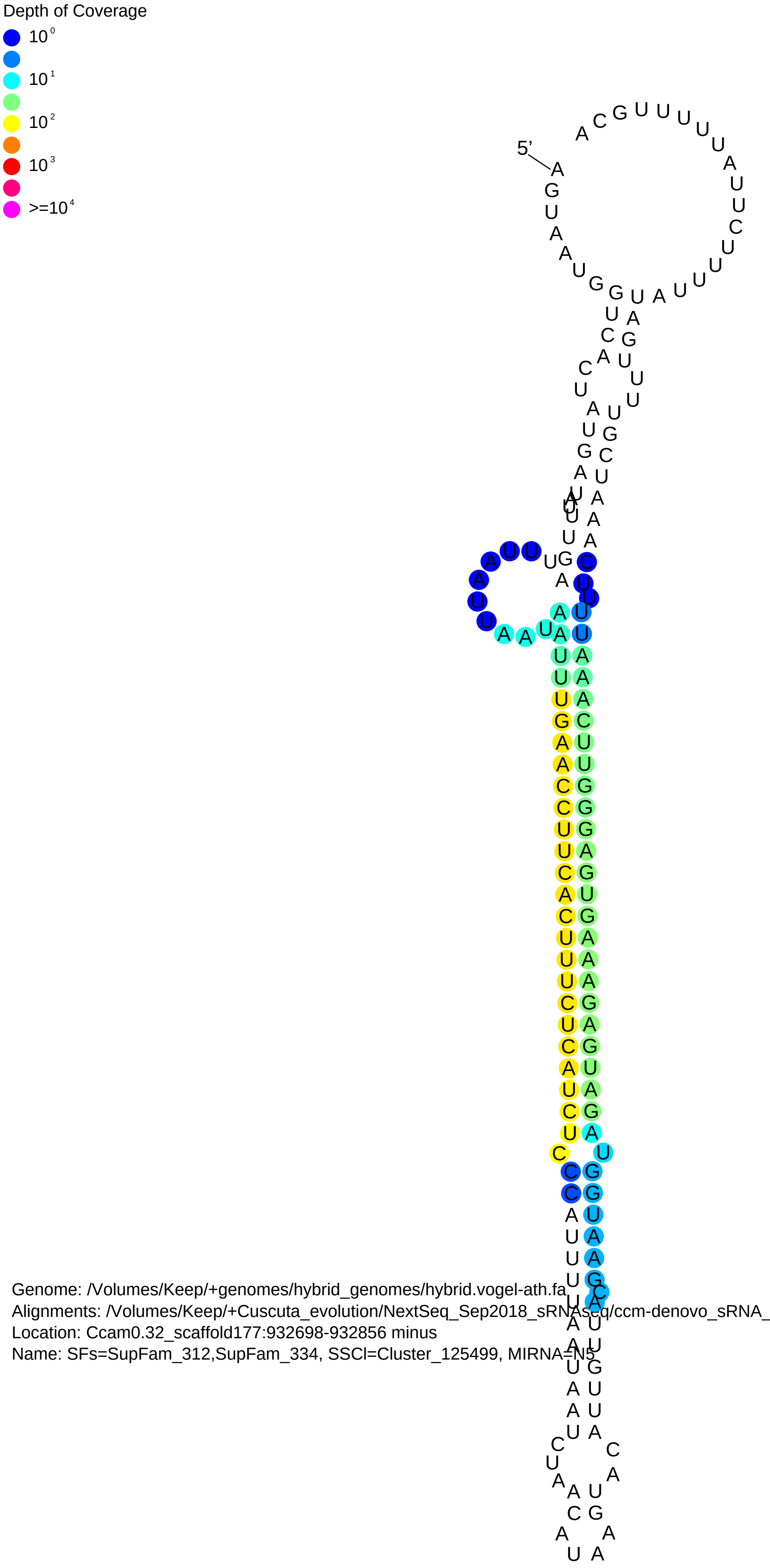

Genome: /Volumes/Keep/+genomes/hybrid_genomes/hybrid.vogel-ath.fa
Alignments: /Volumes/Keep/+Cuscuta_evolution/NextSeq_Sep2018_sRNAseq/ccm-denovo_sRNA_annotation/02out-cca.hybrid.ShortStack/merged_alig
Location: Ccam0.32_scaffold177:932698-932856 minus
Name: SFs=SupFam_312,SupFam_334, SSCl=Cluster_125499, MIRNA=N5

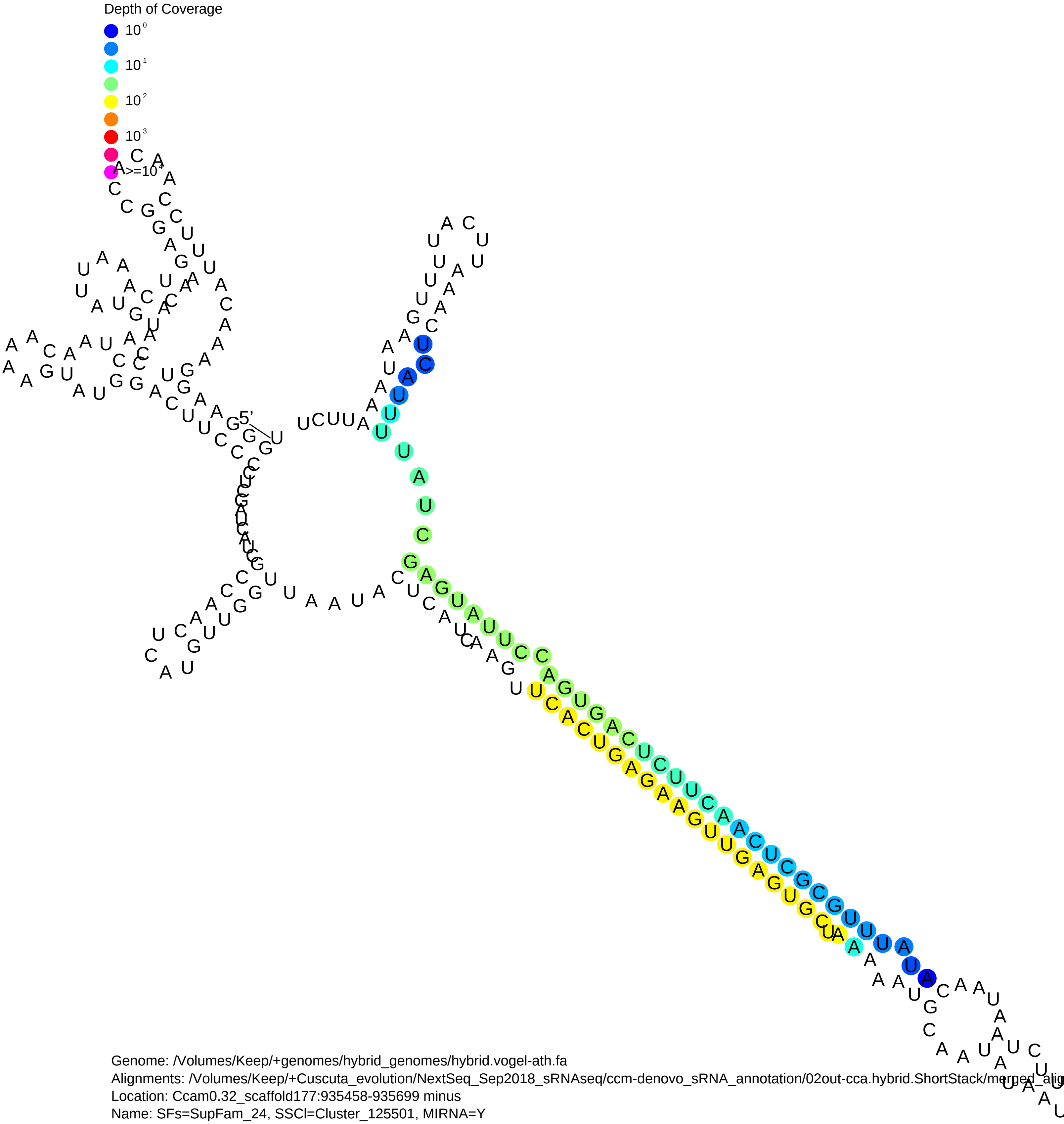

Genome: /Volumes/Keep/+genomes/hybrid_genomes/hybrid.vogel-ath.fa
Alignments: /Volumes/Keep/+Cuscuta_evolution/NextSeq_Sep2018_sRNAseq/ccm-denovo_sRNA_annotation/02out-cca.hybrid.ShortStack/merged_alig
Location: Ccam0.32_scaffold177:935458-935699 minus
Name: SFs=SupFam_24, SSCl=Cluster_125501, MIRNA=Y

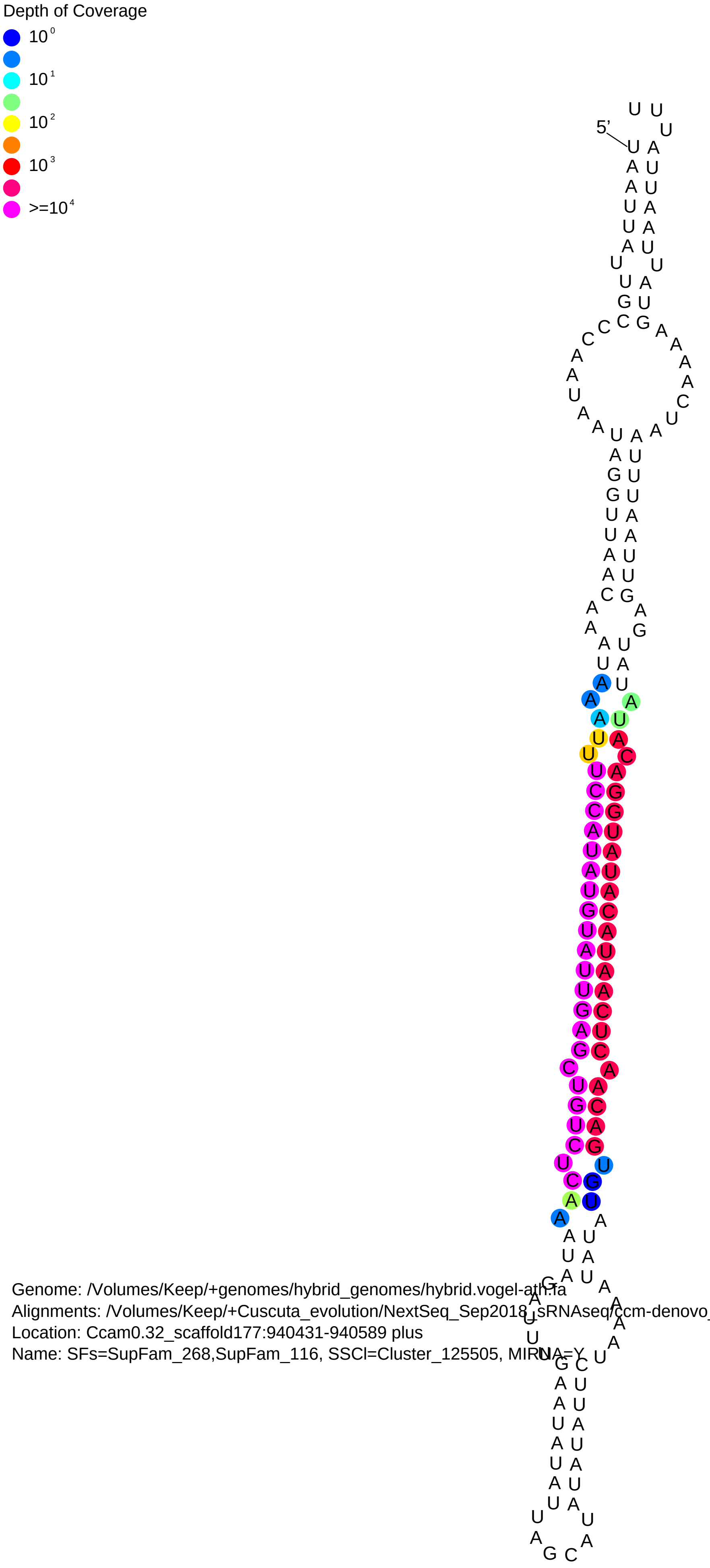

Genome: /Volumes/Keep/+genomes/hybrid_genomes/hybrid.vogel-ath.fa
Alignments: /Volumes/Keep/+Cuscuta_evolution/NextSeq_Sep2018_sRNAseq/ccm-denovo_sRNA_annotation/02out-cca.hybrid.ShortStack/merged_alig
Location: Ccam0.32_scaffold177:940431-940589 plus
Name: SFs=SupFam_268,SupFam_116, SSCl=Cluster_125505, MIRNA=Y

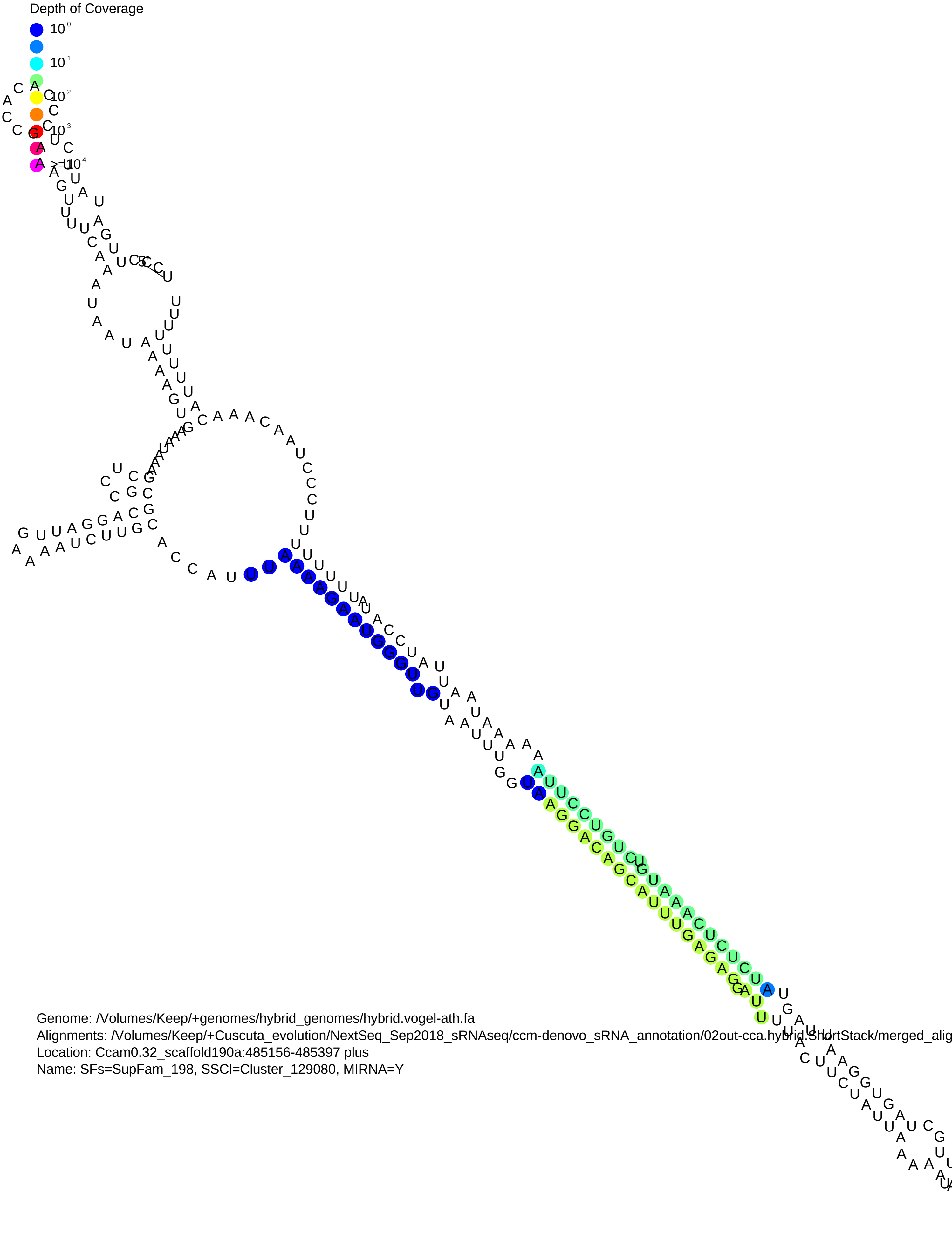

Genome: /Volumes/Keep/+genomes/hybrid_genomes/hybrid.vogel-ath.fa
Alignments: /Volumes/Keep/+Cuscuta_evolution/NextSeq_Sep2018_sRNAseq/ccm-denovo_sRNA_annotation/02out-cca.hybrid.ShortStack/merged_alig
Location: Ccam0.32_scaffold190a:485156-485397 plus
Name: SFs=SupFam_198, SSCl=Cluster_129080, MIRNA=Y

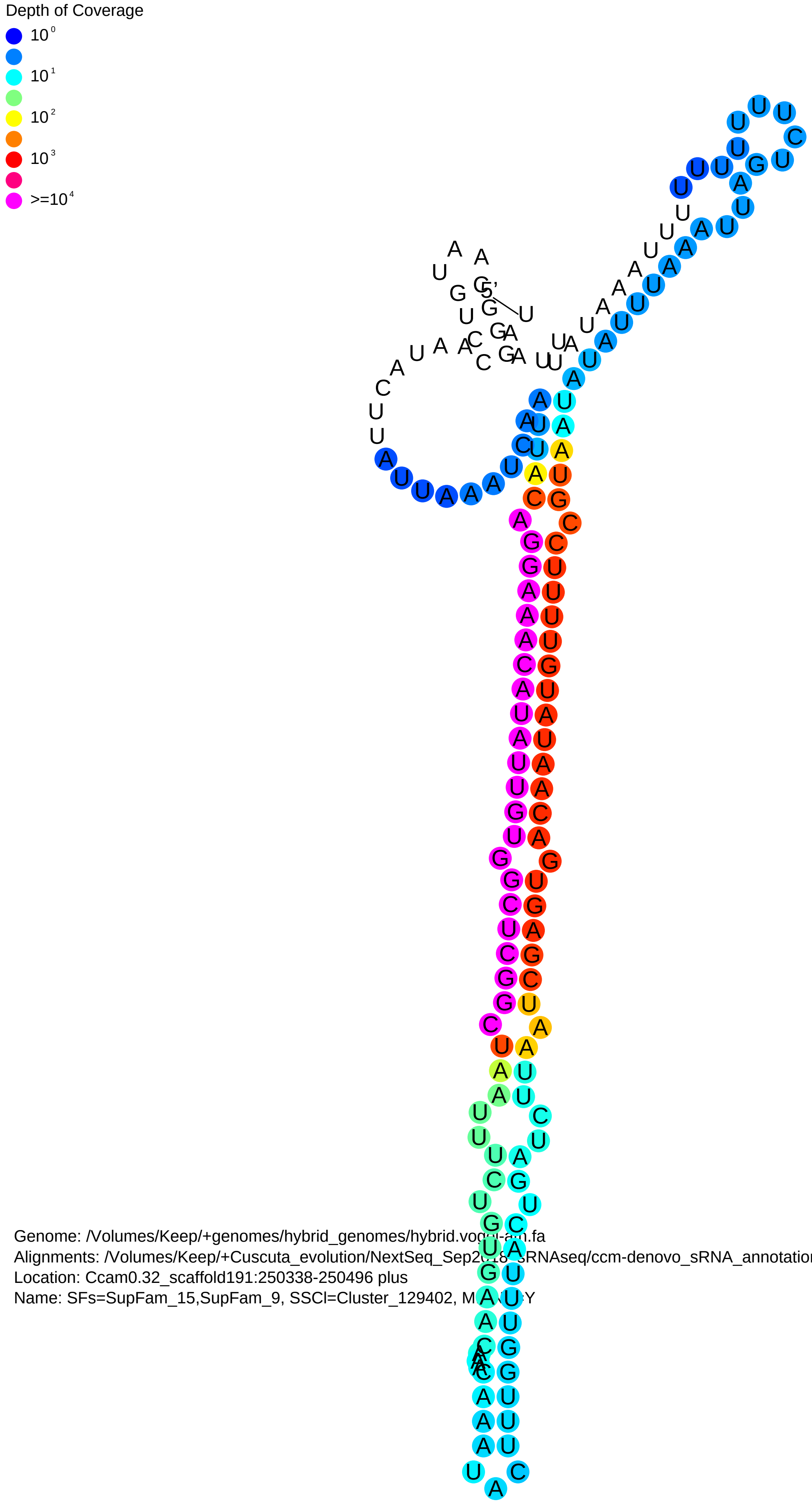

Genome: /Volumes/Keep/+genomes/hybrid_genomes/hybrid.vo[illegible]n.fa
Alignments: /Volumes/Keep/+Cuscuta_evolution/NextSeq_Sep2[illegible]sRNAseq/ccm-denovo_sRNA_annotation/02out-cca.hybrid.ShortStack/merged_alig
Location: Ccam0.32_scaffold191:250338-250496 plus
Name: SFs=SupFam_15,SupFam_9, SSCl=Cluster_129402, M[illegible]Y

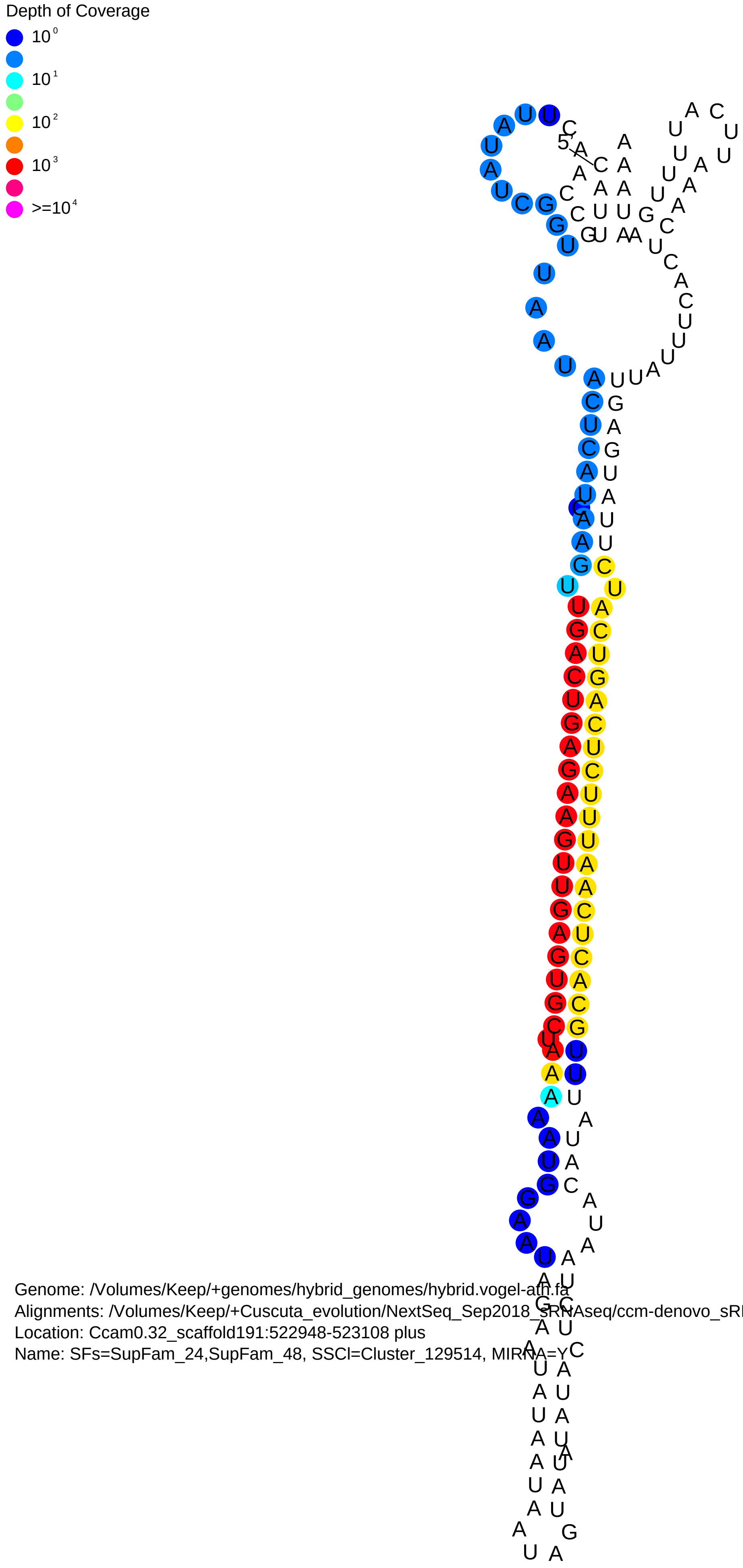

Genome: /Volumes/Keep/+genomes/hybrid_genomes/hybrid.vogel-ath.fa
Alignments: /Volumes/Keep/+Cuscuta_evolution/NextSeq_Sep2018_sRNAseq/ccm-denovo_sRNA_annotation/02out-cca.hybrid.ShortStack/merged_alig
Location: Ccam0.32_scaffold191:522948-523108 plus
Name: SFs=SupFam_24,SupFam_48, SSCl=Cluster_129514, MIRNA=Y

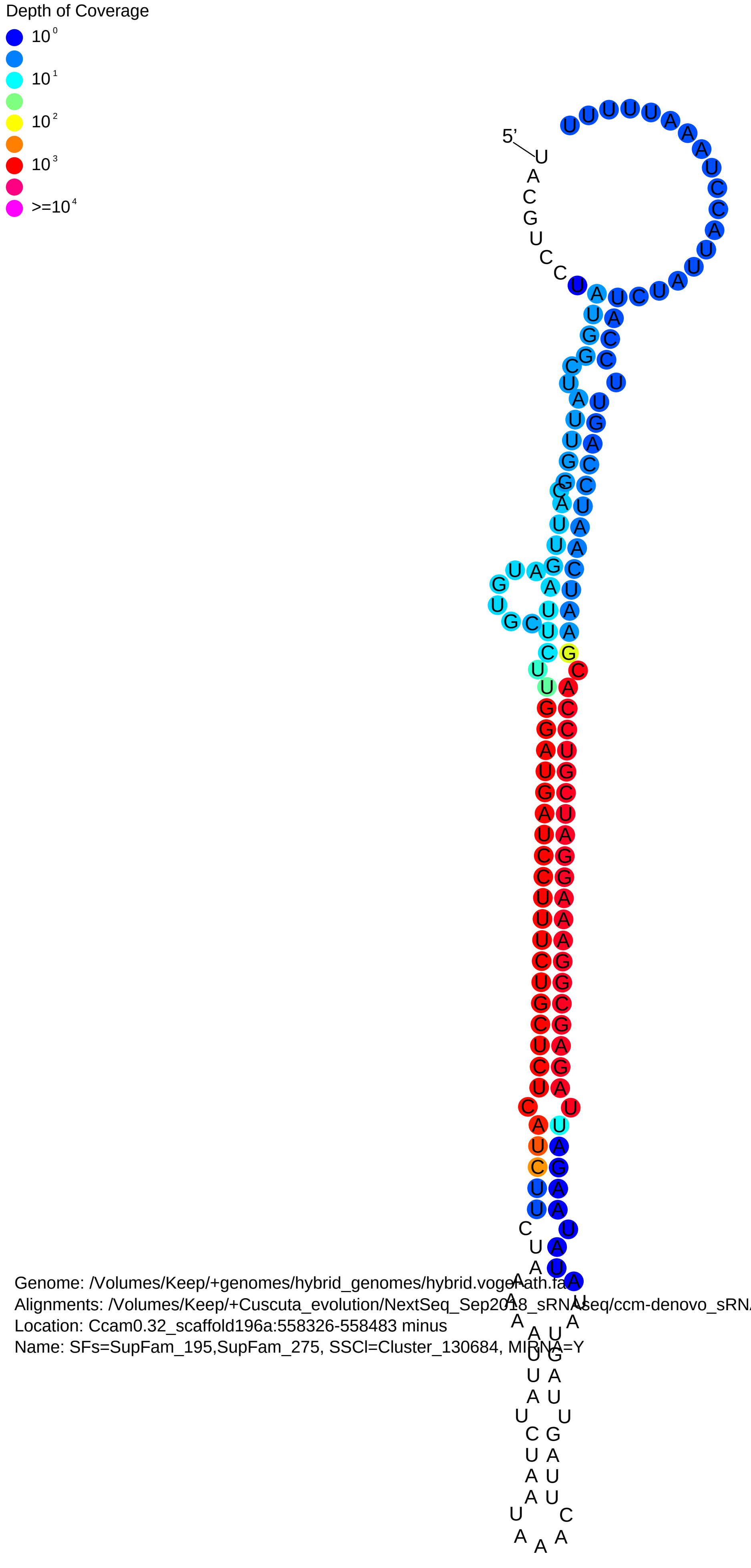

Genome: /Volumes/Keep/+genomes/hybrid_genomes/hybrid.vogel-ath.fa
Alignments: /Volumes/Keep/+Cuscuta_evolution/NextSeq_Sep2018_sRNAseq/ccm-denovo_sRNA_annotation/02out-cca.hybrid.ShortStack/merged_alig
Location: Ccam0.32_scaffold196a:558326-558483 minus
Name: SFs=SupFam_195,SupFam_275, SSCl=Cluster_130684, MIRNA=Y

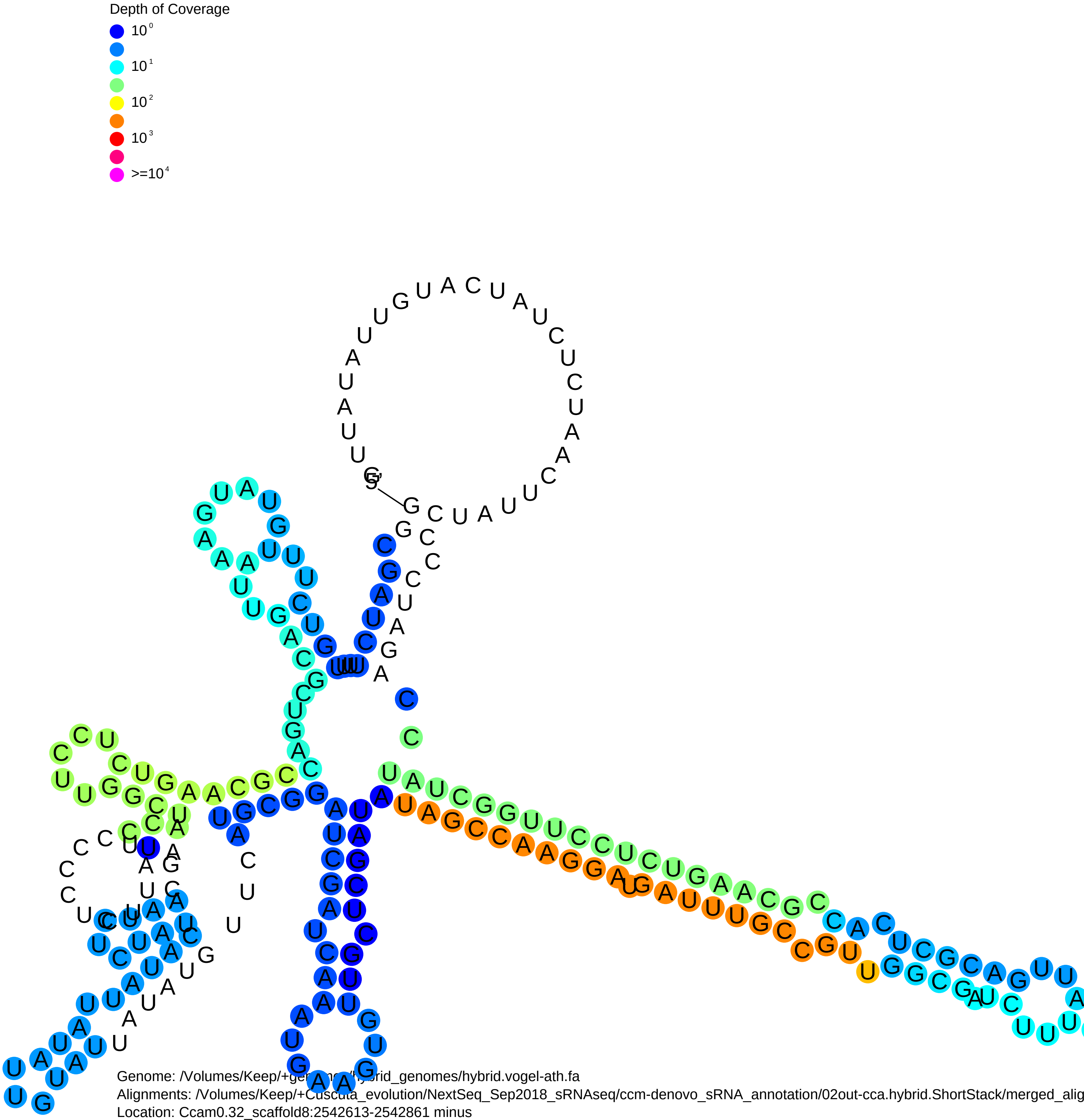

Genome: /Volumes/Keep/+ge[illegible]brid_genomes/hybrid.vogel-ath.fa

Alignments: /Volumes/Keep/+Cuscuta_evolution/NextSeq_Sep2018_sRNAseq/ccm-denovo_sRNA_annotation/02out-cca.hybrid.ShortStack/merged_alig

Location: Ccam0.32_scaffold8:2542613-2542861 minus

Name: SFs=SupFam_333,SupFam_72, SSCl=Cluster_13071, MIRNA=N5

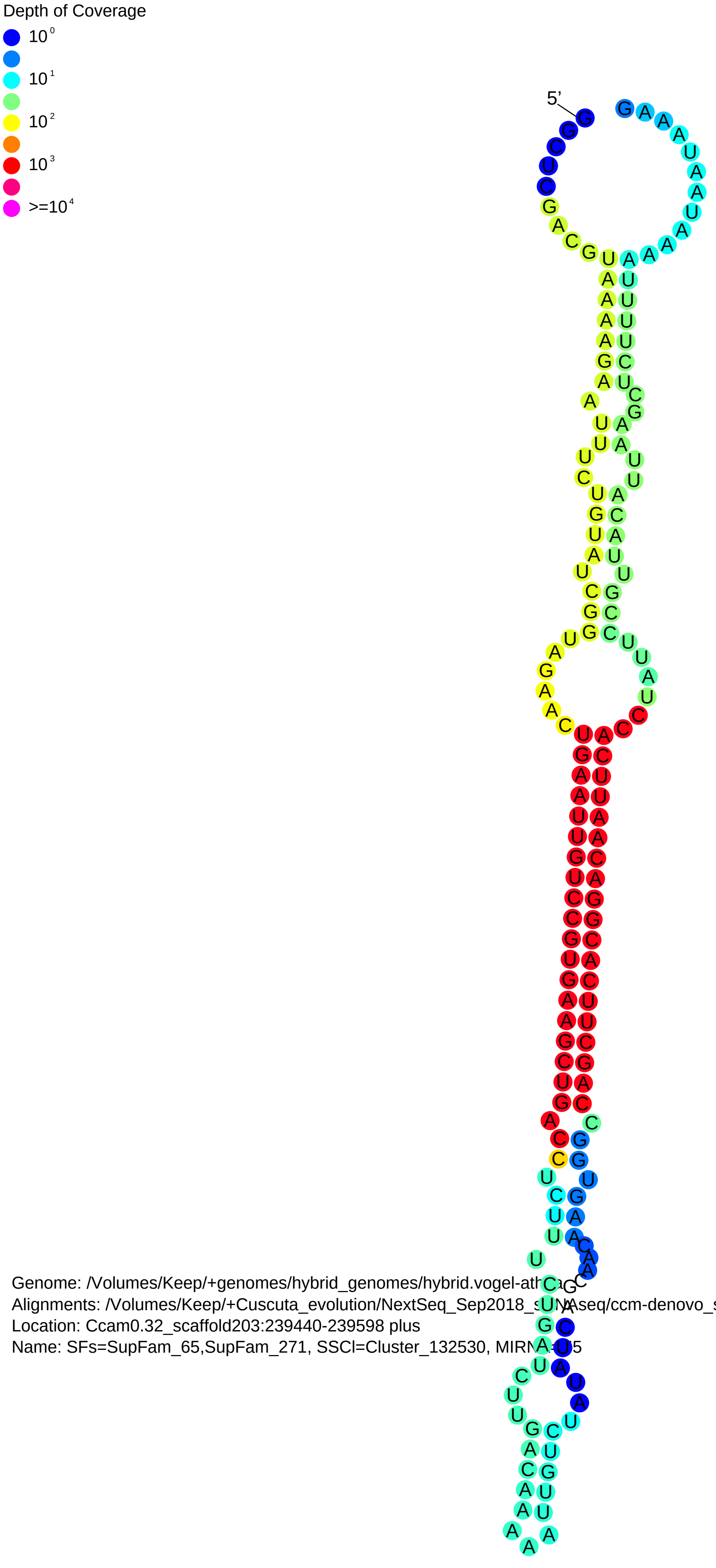

Genome: /Volumes/Keep/+genomes/hybrid_genomes/hybrid.vogel-ath[illegible]
Alignments: /Volumes/Keep/+Cuscuta_evolution/NextSeq_Sep2018_s[illegible]Aseq/ccm-denovo_sRNA_annotation/02out-cca.hybrid.ShortStack/merged_alig
Location: Ccam0.32_scaffold203:239440-239598 plus
Name: SFs=SupFam_65,SupFam_271, SSCl=Cluster_132530, MIRN[illegible]5

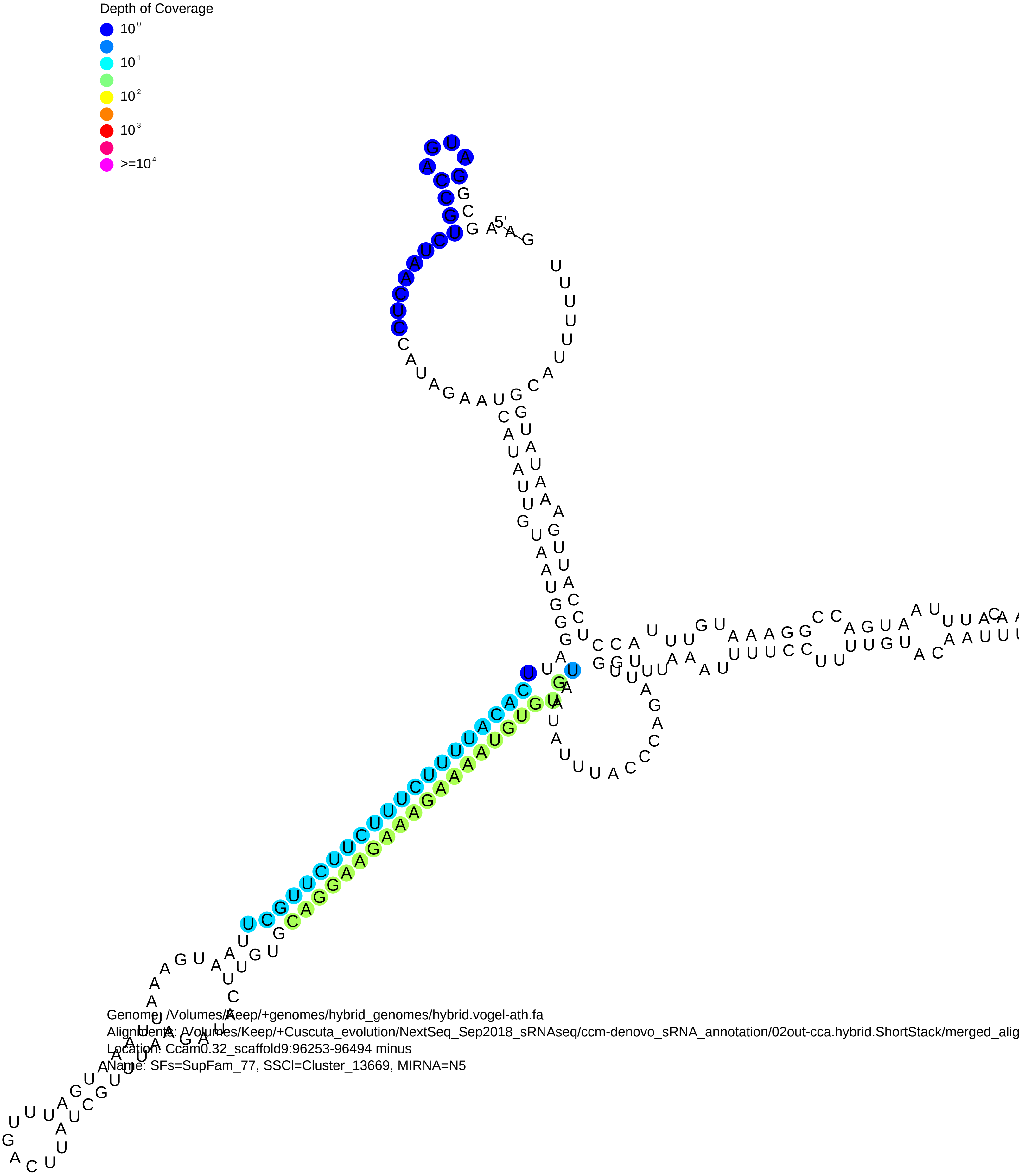

Genome: /Volumes/Keep/+genomes/hybrid_genomes/hybrid.vogel-ath.fa
Alignments: /Volumes/Keep/+Cuscuta_evolution/NextSeq_Sep2018_sRNAseq/ccm-denovo_sRNA_annotation/02out-cca.hybrid.ShortStack/merged_alig
Location: Ccam0.32_scaffold9:96253-96494 minus
Name: SFs=SupFam_77, SSCl=Cluster_13669, MIRNA=N5

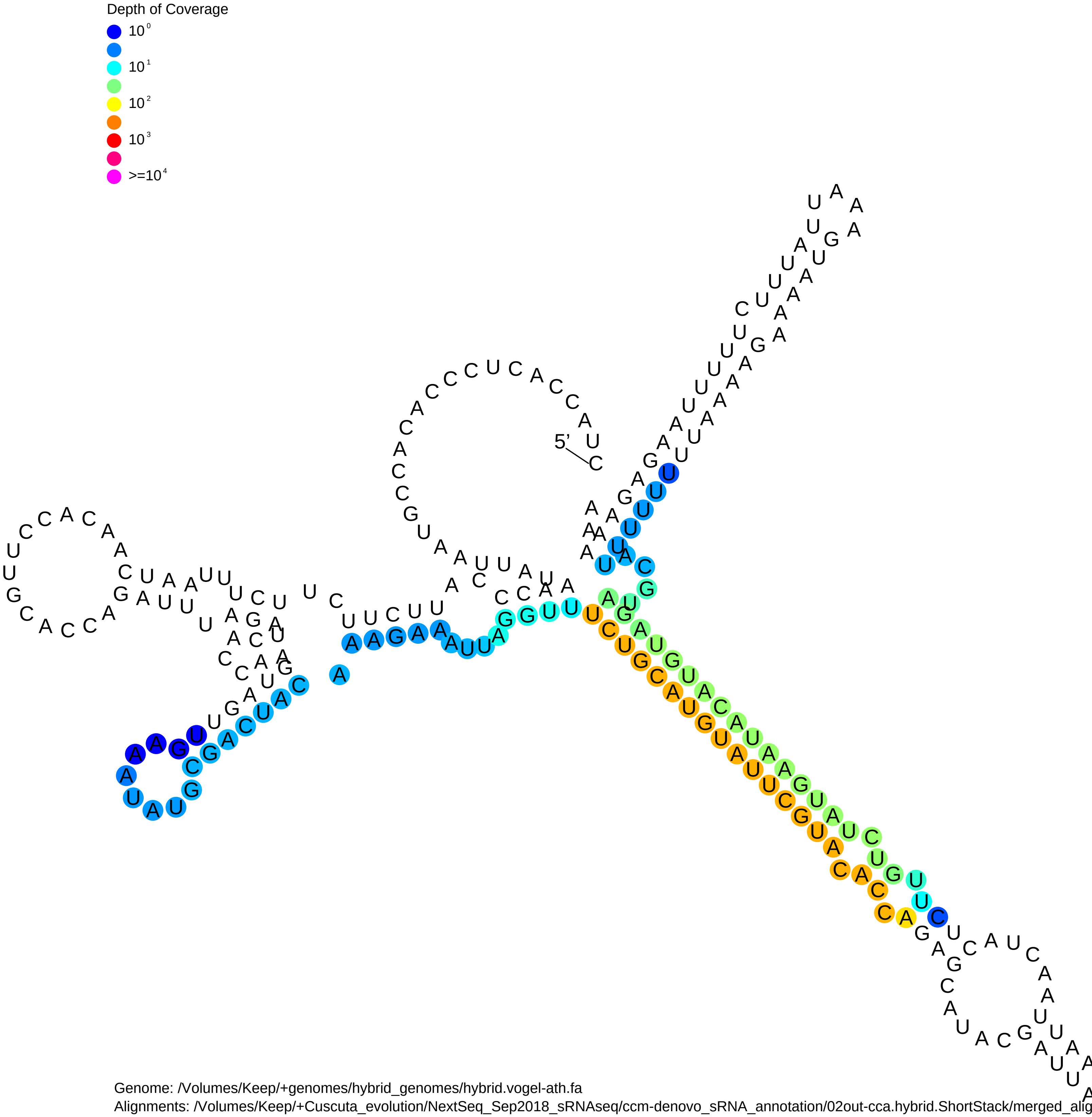

Genome: /Volumes/Keep/+genomes/hybrid_genomes/hybrid.vogel-ath.fa
Alignments: /Volumes/Keep/+Cuscuta_evolution/NextSeq_Sep2018_sRNAseq/ccm-denovo_sRNA_annotation/02out-cca.hybrid.ShortStack/merged_alig
Location: Ccam0.32_scaffold9:103105-103346 plus
Name: SFs=SupFam_1, SSCl=Cluster_13673, MIRNA=Y

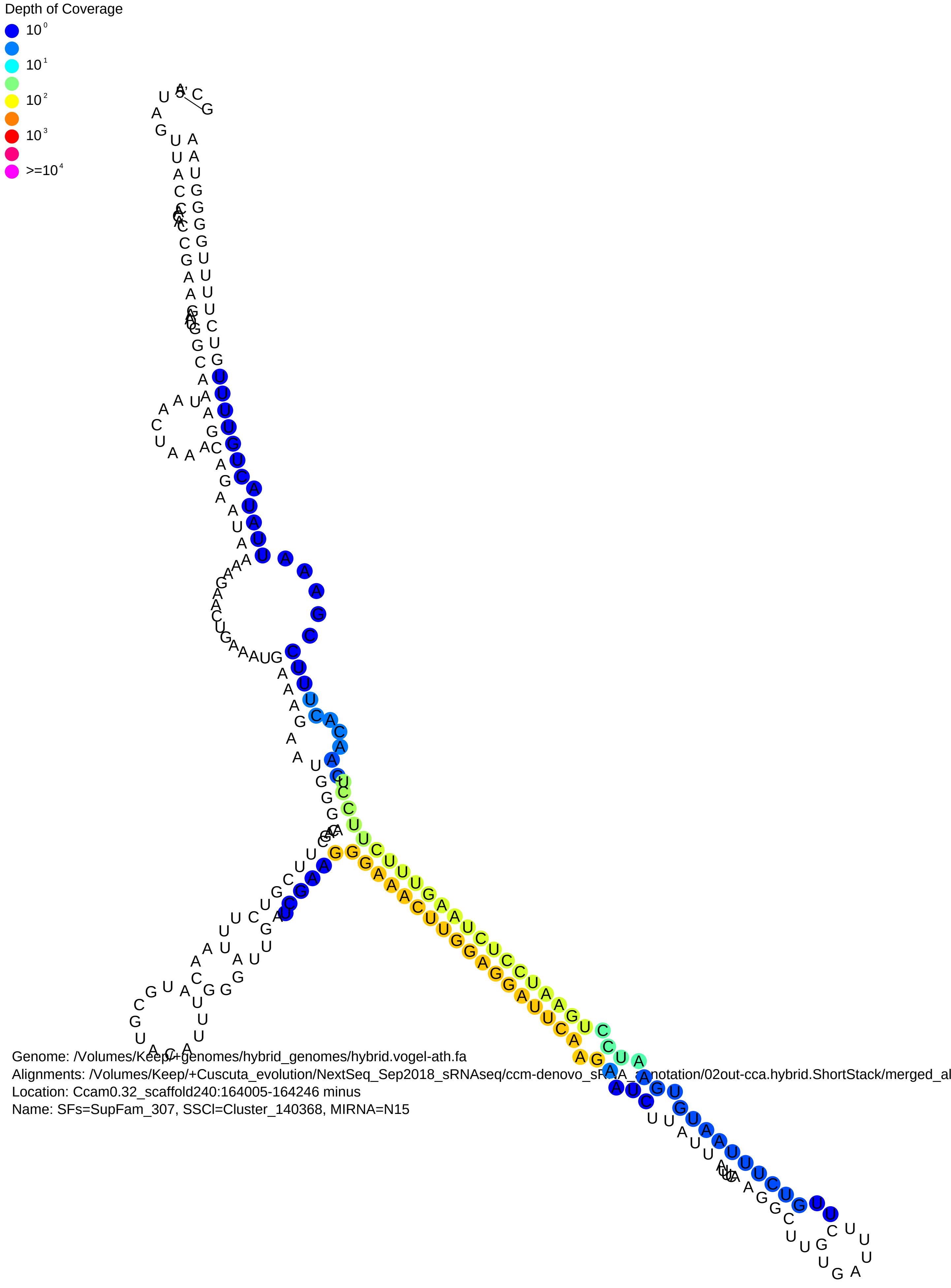

Genome: /Volumes/Keep/+genomes/hybrid_genomes/hybrid.vogel-ath.fa
Alignments: /Volumes/Keep/+Cuscuta_evolution/NextSeq_Sep2018_sRNAseq/ccm-denovo_sRNA_annotation/02out-cca.hybrid.ShortStack/merged_alig
Location: Ccam0.32_scaffold240:164005-164246 minus
Name: SFs=SupFam_307, SSCl=Cluster_140368, MIRNA=N15

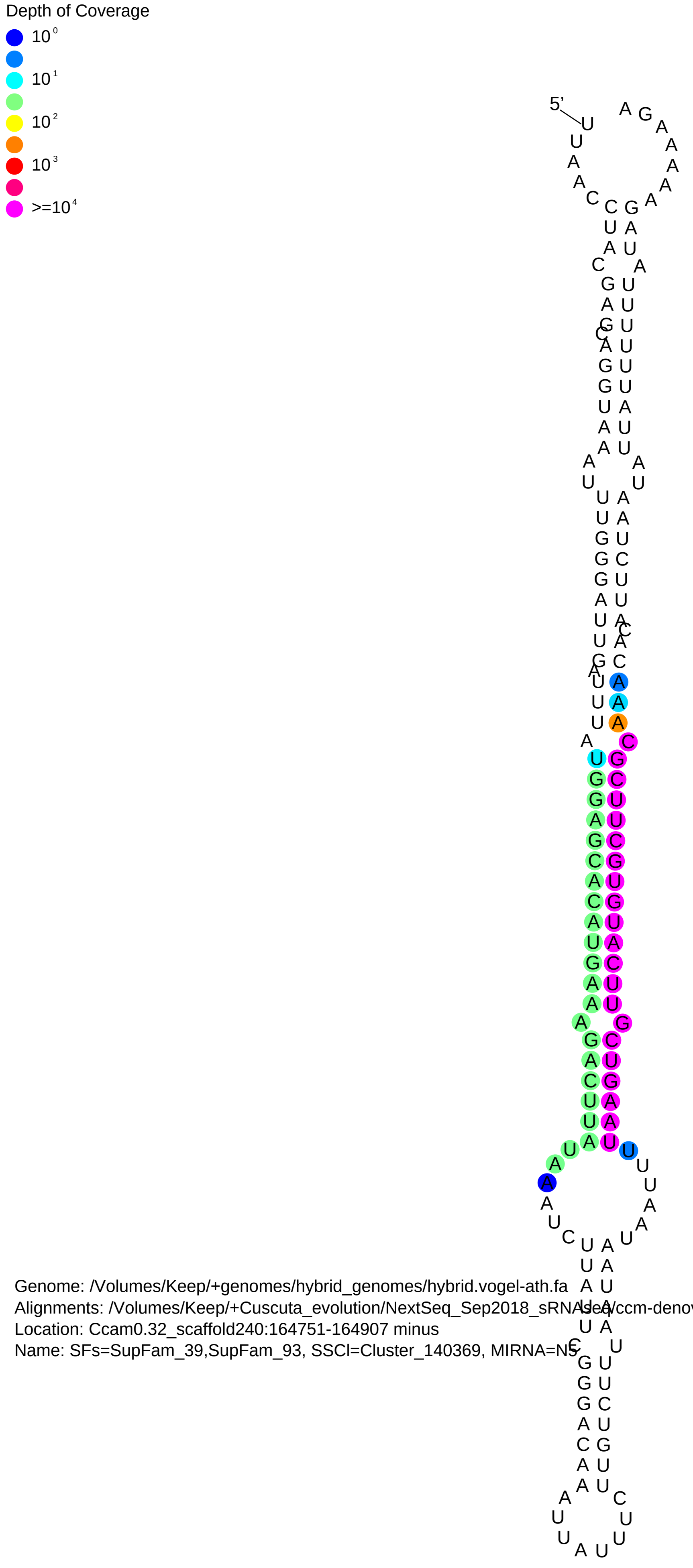

Genome: /Volumes/Keep/+genomes/hybrid_genomes/hybrid.vogel-ath.fa
Alignments: /Volumes/Keep/+Cuscuta_evolution/NextSeq_Sep2018_sRNAseq/ccm-denovo_sRNA_annotation/02out-cca.hybrid.ShortStack/merged_alig
Location: Ccam0.32_scaffold240:164751-164907 minus
Name: SFs=SupFam_39,SupFam_93, SSCl=Cluster_140369, MIRNA=NS

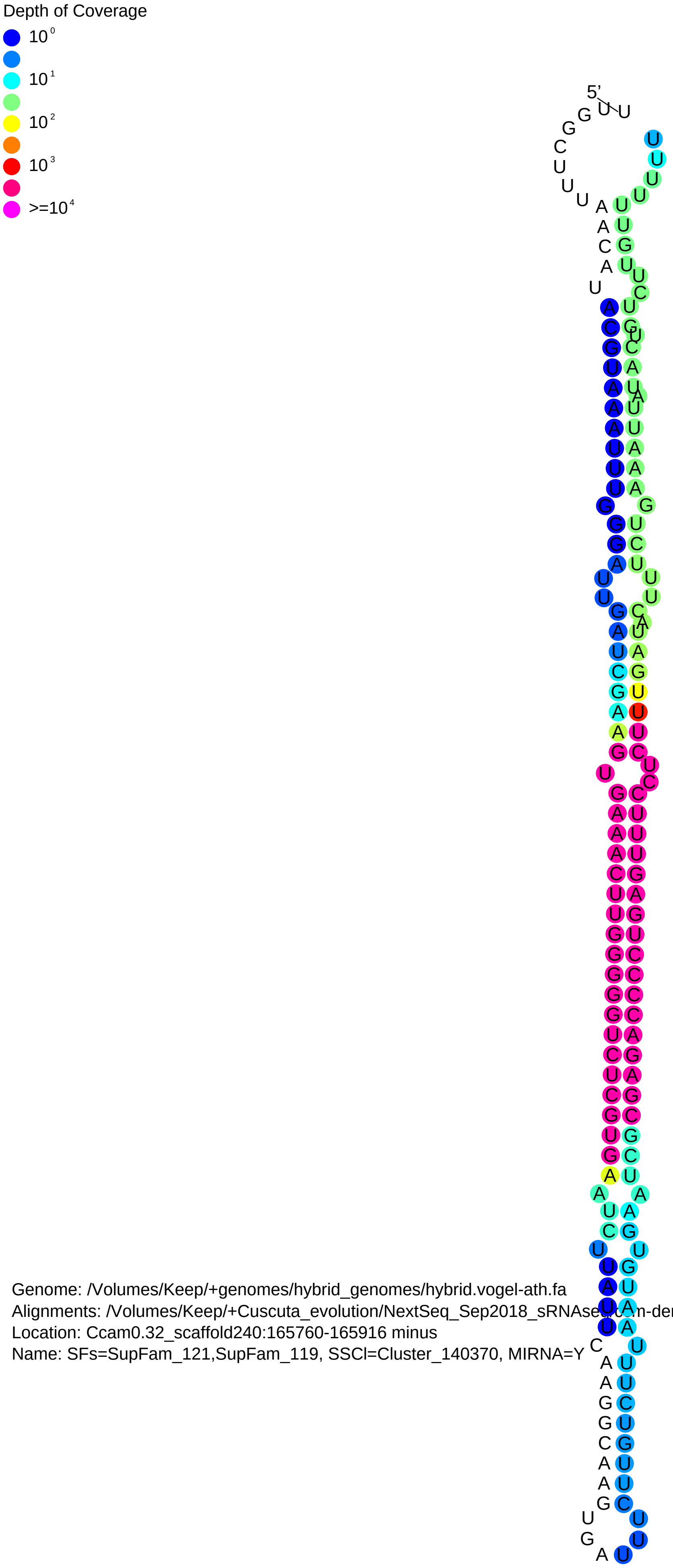

Genome: /Volumes/Keep/+genomes/hybrid_genomes/hybrid.vogel-ath.fa
Alignments: /Volumes/Keep/+Cuscuta_evolution/NextSeq_Sep2018_sRNAse[illegible]m-denovo_sRNA_annotation/02out-cca.hybrid.ShortStack/merged_alig
Location: Ccam0.32_scaffold240:165760-165916 minus
Name: SFs=SupFam_121,SupFam_119, SSCl=Cluster_140370, MIRNA=Y

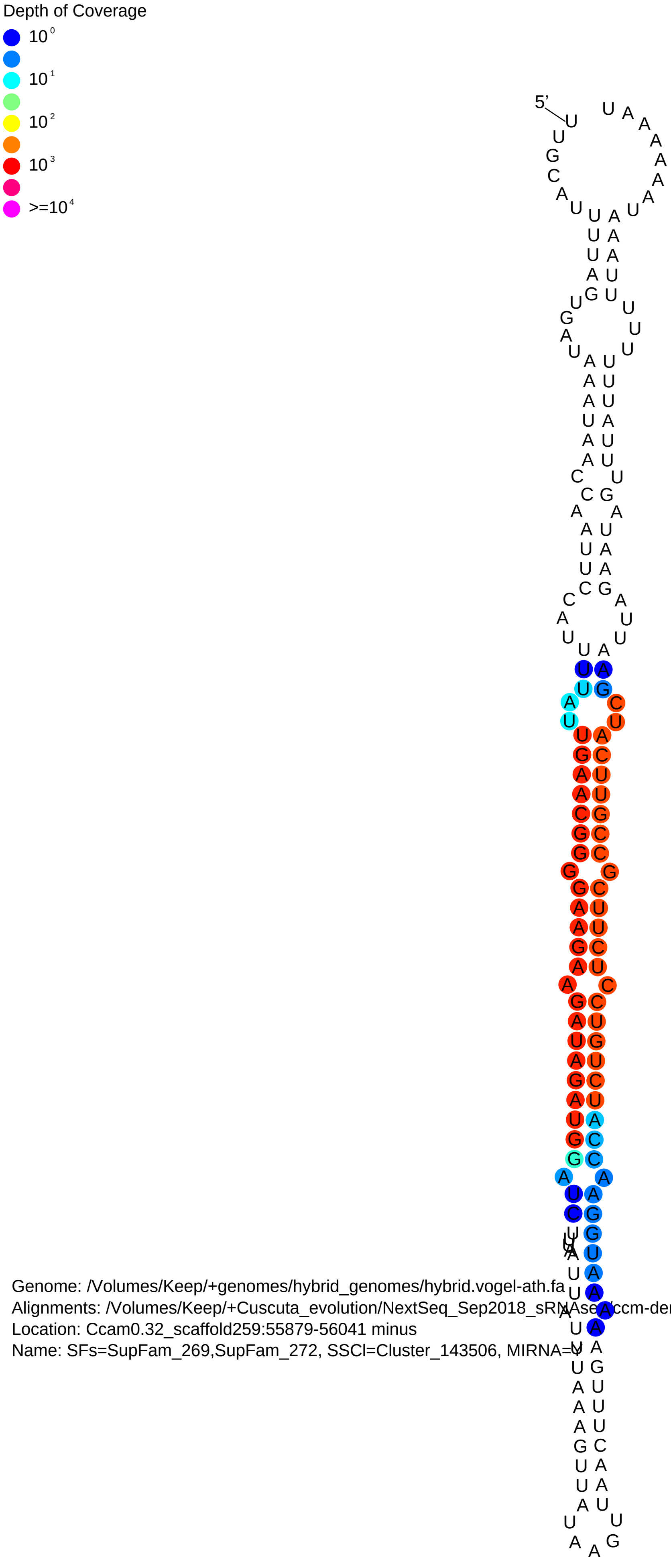

Genome: /Volumes/Keep/+genomes/hybrid_genomes/hybrid.vogel-ath.fa
Alignments: /Volumes/Keep/+Cuscuta_evolution/NextSeq_Sep2018_sRNAse[illegible]ccm-denovo_sRNA_annotation/02out-cca.hybrid.ShortStack/merged_alig
Location: Ccam0.32_scaffold259:55879-56041 minus
Name: SFs=SupFam_269,SupFam_272, SSCl=Cluster_143506, MIRNA=Y

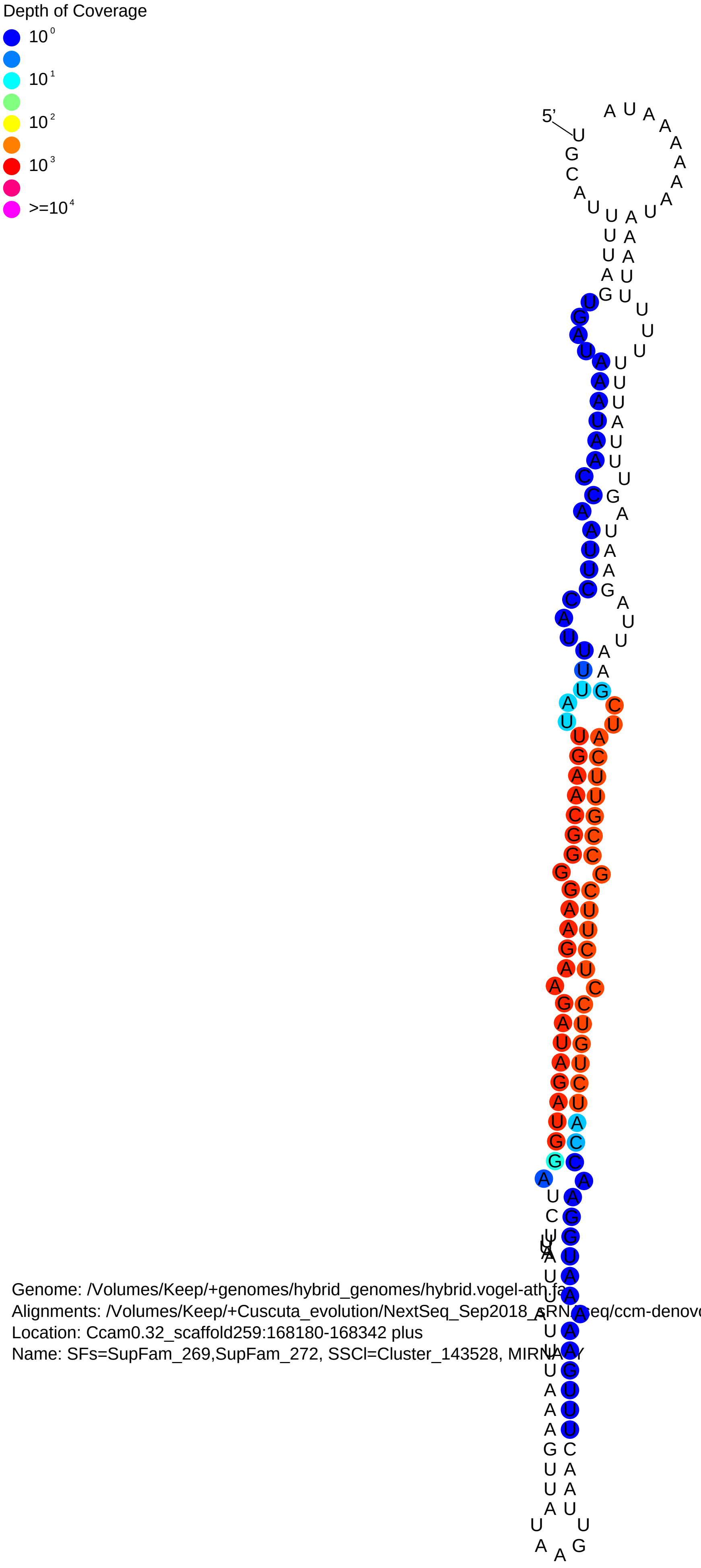

Genome: /Volumes/Keep/+genomes/hybrid_genomes/hybrid.vogel-ath.fa
Alignments: /Volumes/Keep/+Cuscuta_evolution/NextSeq_Sep2018_sRNAseq/ccm-denovo_sRNA_annotation/02out-cca.hybrid.ShortStack/merged_alig
Location: Ccam0.32_scaffold259:168180-168342 plus
Name: SFs=SupFam_269,SupFam_272, SSCl=Cluster_143528, MIRNA=Y

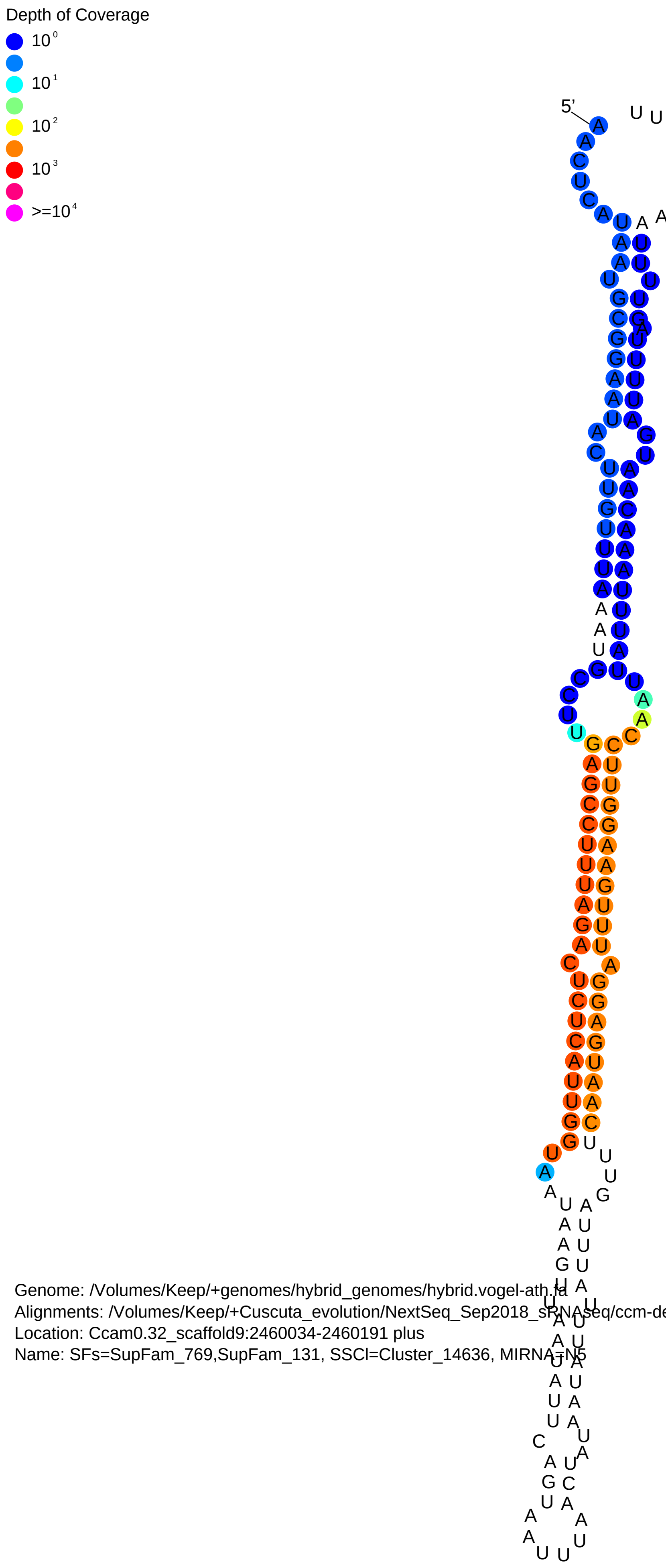

Genome: /Volumes/Keep/+genomes/hybrid_genomes/hybrid.vogel-ath.fa
Alignments: /Volumes/Keep/+Cuscuta_evolution/NextSeq_Sep2018_sRNAseq/ccm-denovo_sRNA_annotation/02out-cca.hybrid.ShortStack/merged_alig
Location: Ccam0.32_scaffold9:2460034-2460191 plus
Name: SFs=SupFam_769,SupFam_131, SSCl=Cluster_14636, MIRNA=N5

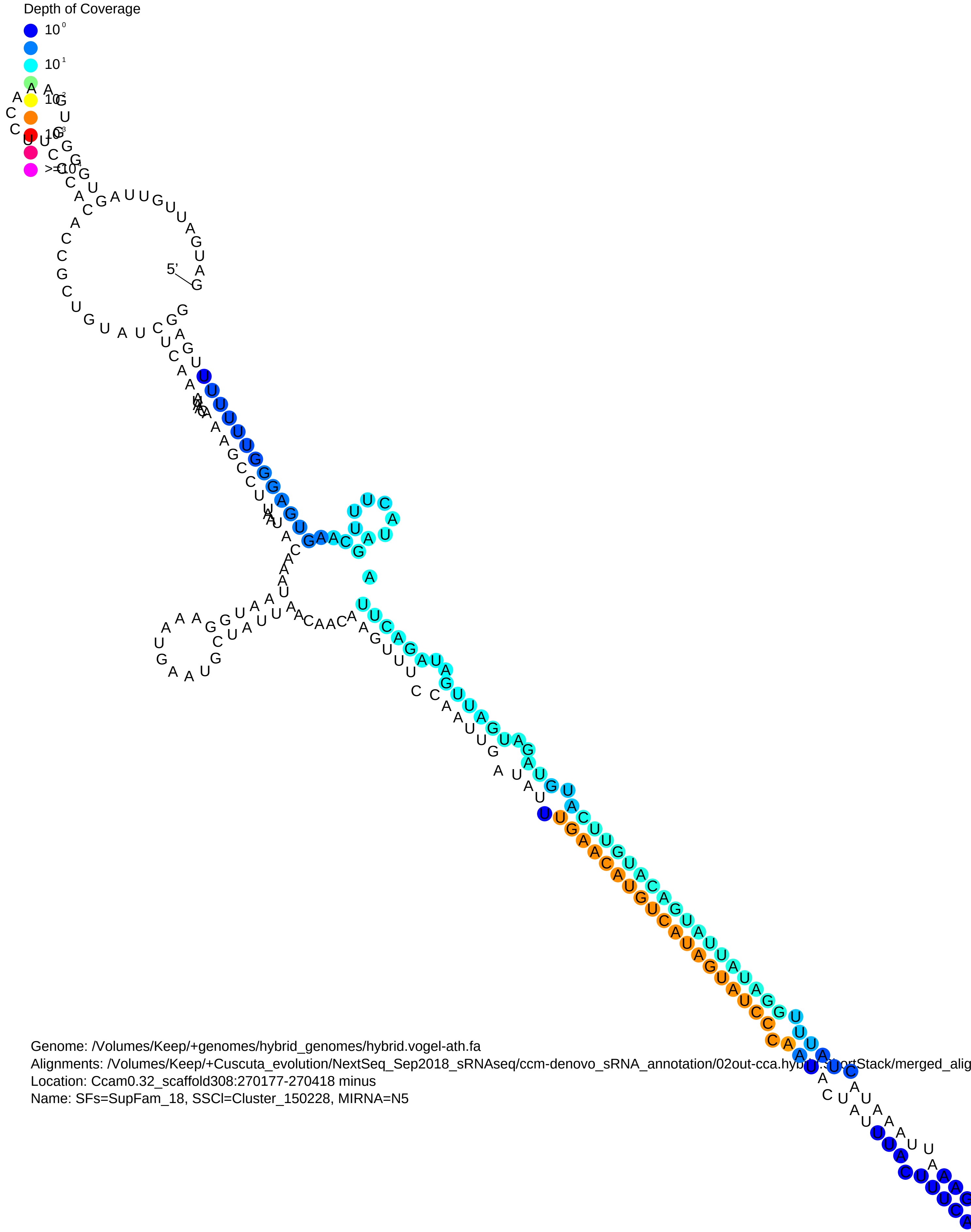

Genome: /Volumes/Keep/+genomes/hybrid_genomes/hybrid.vogel-ath.fa
Alignments: /Volumes/Keep/+Cuscuta_evolution/NextSeq_Sep2018_sRNAseq/ccm-denovo_sRNA_annotation/02out-cca.hyb[illegible]ortStack/merged_alig
Location: Ccam0.32_scaffold308:270177-270418 minus
Name: SFs=SupFam_18, SSCl=Cluster_150228, MIRNA=N5

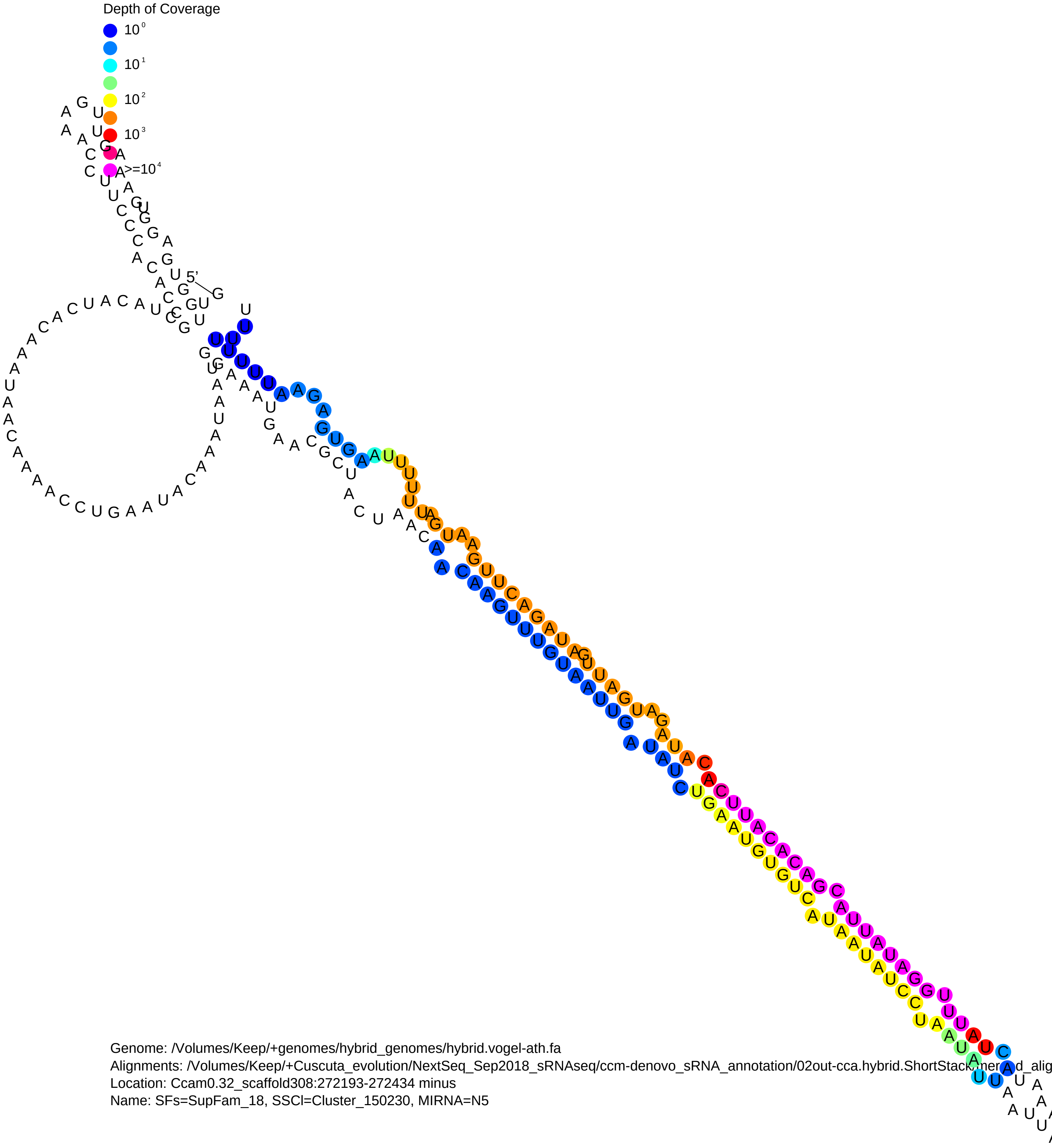

Genome: /Volumes/Keep/+genomes/hybrid_genomes/hybrid.vogel-ath.fa
Alignments: /Volumes/Keep/+Cuscuta_evolution/NextSeq_Sep2018_sRNAseq/ccm-denovo_sRNA_annotation/02out-cca.hybrid.ShortStack/merged_alig
Location: Ccam0.32_scaffold308:272193-272434 minus
Name: SFs=SupFam_18, SSCl=Cluster_150230, MIRNA=N5

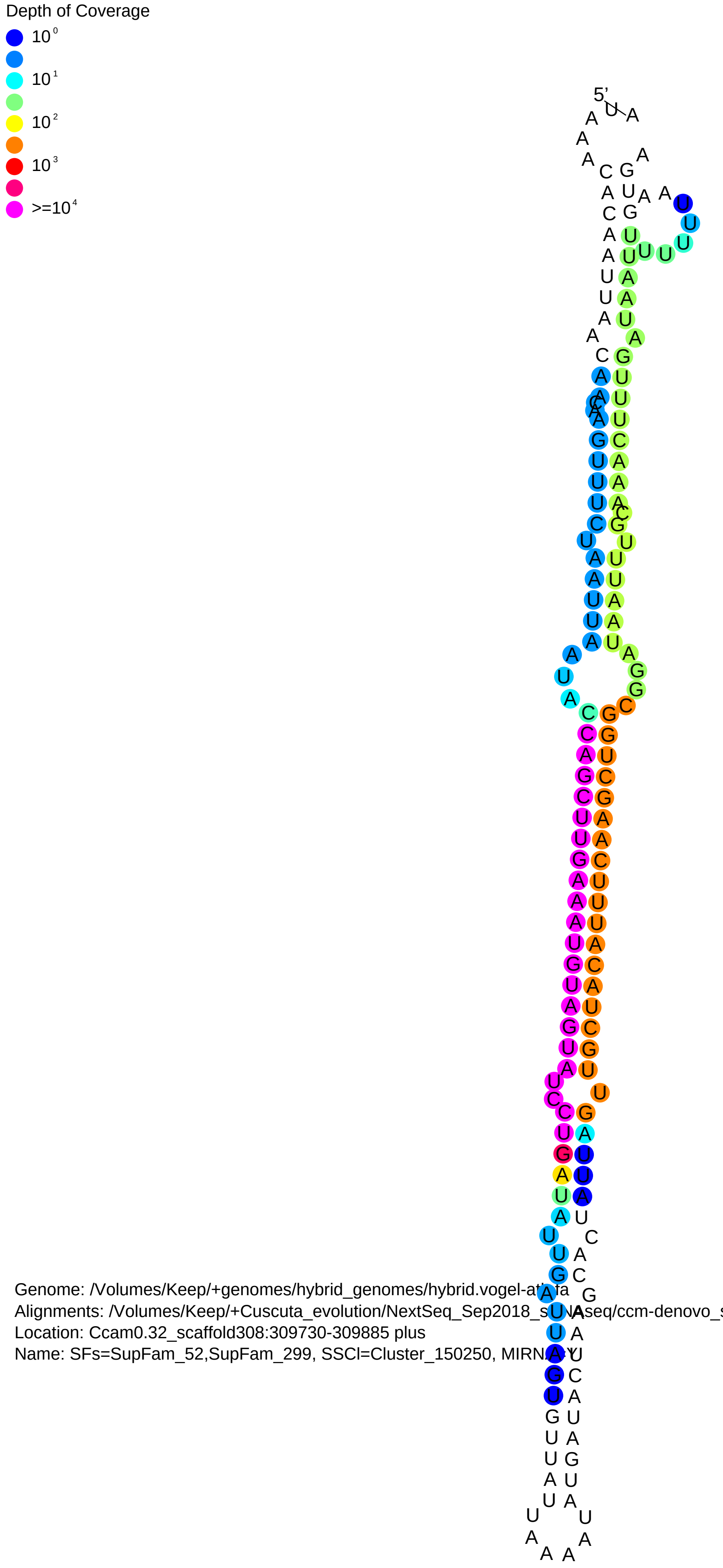

Genome: /Volumes/Keep/+genomes/hybrid_genomes/hybrid.vogel-at[illegible]fa
Alignments: /Volumes/Keep/+Cuscuta_evolution/NextSeq_Sep2018_s[illegible]seq/ccm-denovo_sRNA_annotation/02out-cca.hybrid.ShortStack/merged_alig
Location: Ccam0.32_scaffold308:309730-309885 plus
Name: SFs=SupFam_52,SupFam_299, SSCl=Cluster_150250, MIRNA[illegible]

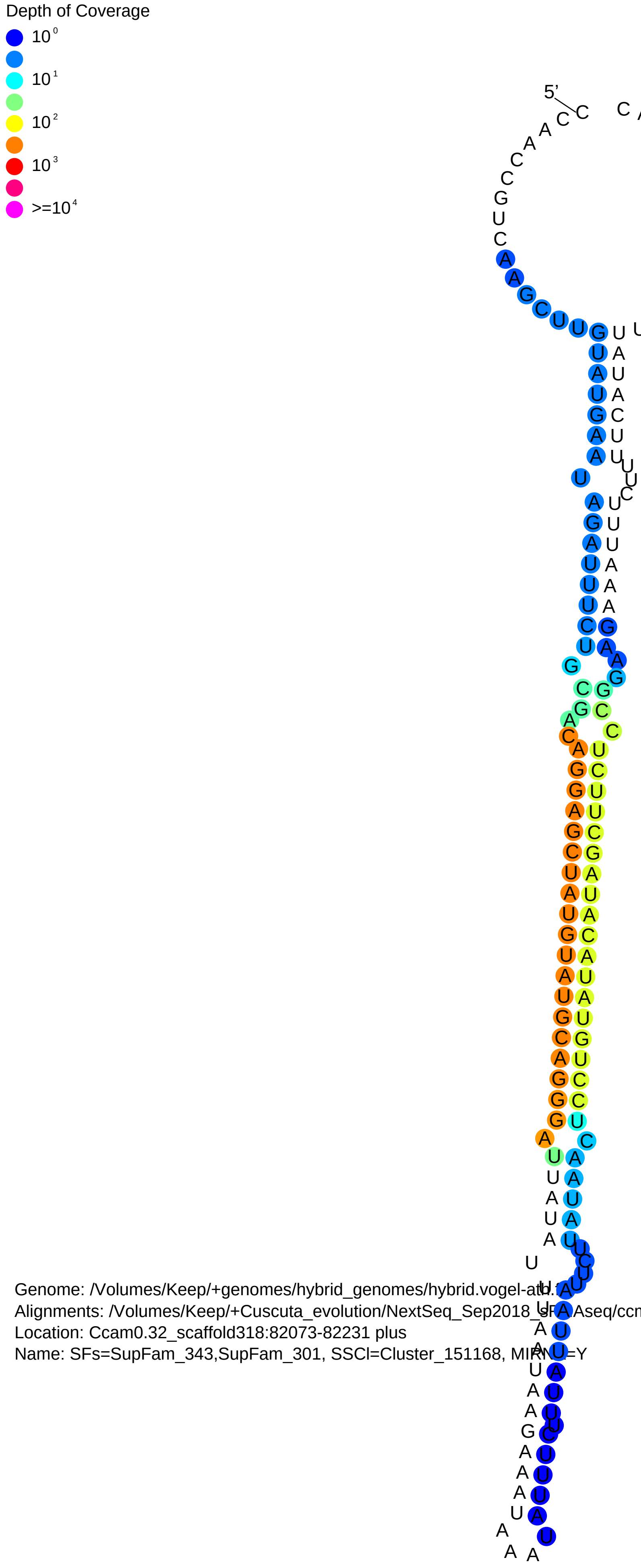

Genome: /Volumes/Keep/+genomes/hybrid_genomes/hybrid.vogel-at[illegible]
Alignments: /Volumes/Keep/+Cuscuta_evolution/NextSeq_Sep2018_s[illegible]Aseq/ccm-denovo_sRNA_annotation/02out-cca.hybrid.ShortStack/merged_alig
Location: Ccam0.32_scaffold318:82073-82231 plus
Name: SFs=SupFam_343,SupFam_301, SSCl=Cluster_151168, MIR[illegible]=Y

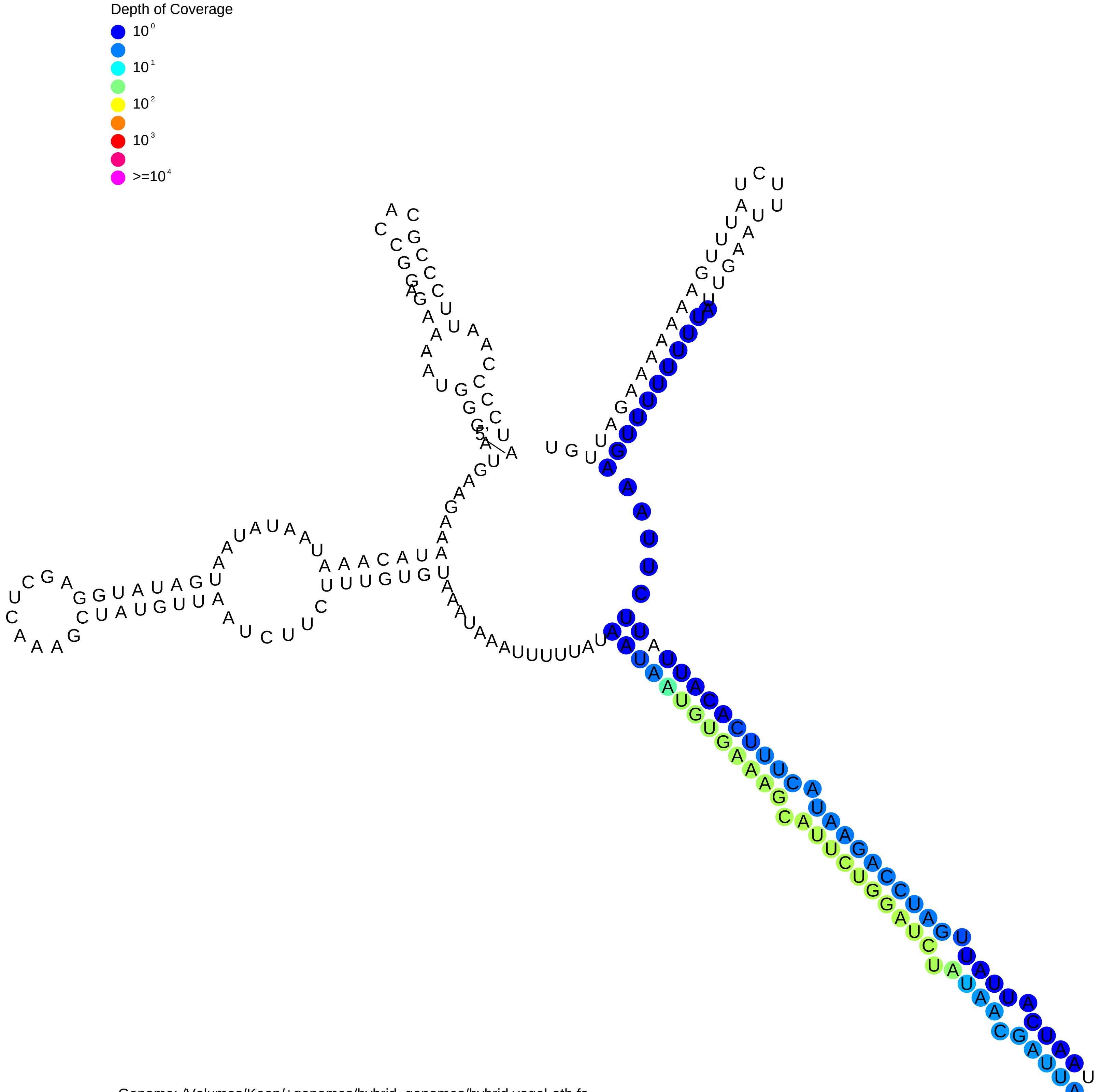

Genome: /Volumes/Keep/+genomes/hybrid_genomes/hybrid.vogel-ath.fa
Alignments: /Volumes/Keep/+Cuscuta_evolution/NextSeq_Sep2018_sRNAseq/ccm-denovo_sRNA_annotation/02out-cca.hybrid.ShortStack/merged_[illegible]
Location: Ccam0.32_scaffold318:83484-83725 plus
Name: SFs=SupFam_196, SSCl=Cluster_151171, MIRNA=N5

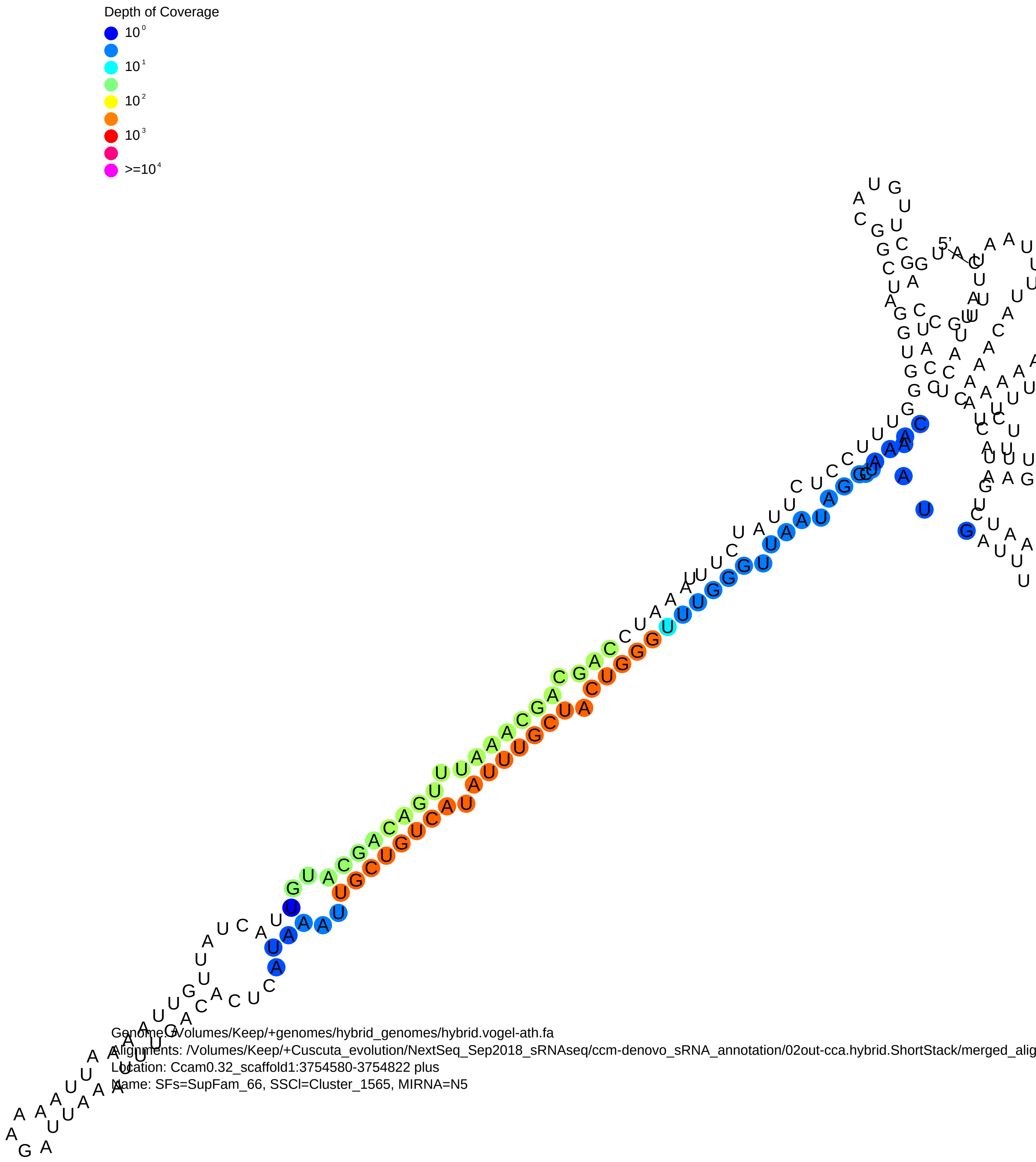

Genome: /Volumes/Keep/+genomes/hybrid_genomes/hybrid.vogel-ath.fa
Alignments: /Volumes/Keep/+Cuscuta_evolution/NextSeq_Sep2018_sRNAseq/ccm-denovo_sRNA_annotation/02out-cca.hybrid.ShortStack/merged_alig[illegible]
Location: Ccam0.32_scaffold1:3754580-3754822 plus
Name: SFs=SupFam_66, SSCl=Cluster_1565, MIRNA=N5

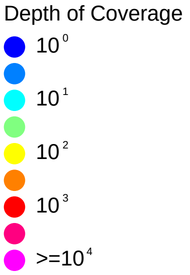

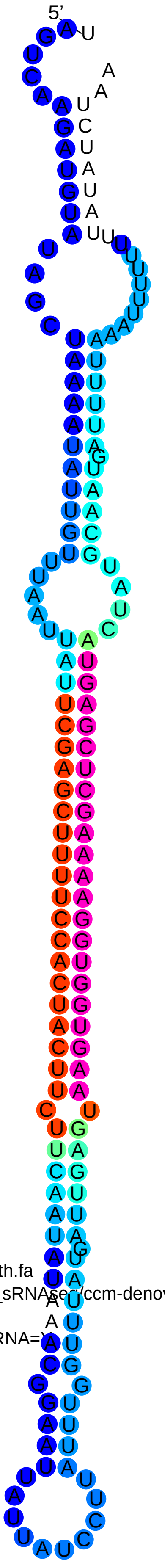

Genome: /Volumes/Keep/+genomes/hybrid_genomes/hybrid.vogel-ath.fa
Alignments: /Volumes/Keep/+Cuscuta_evolution/NextSeq_Sep2018_sRNAseq/ccm-denovo_sRNA_annotation/02out-cca.hybrid.ShortStack/merged_alig
Location: Ccam0.32_scaffold425:242866-243023 minus
Name: SFs=SupFam_263,SupFam_194, SSCl=Cluster_158194, MIRNA=Y

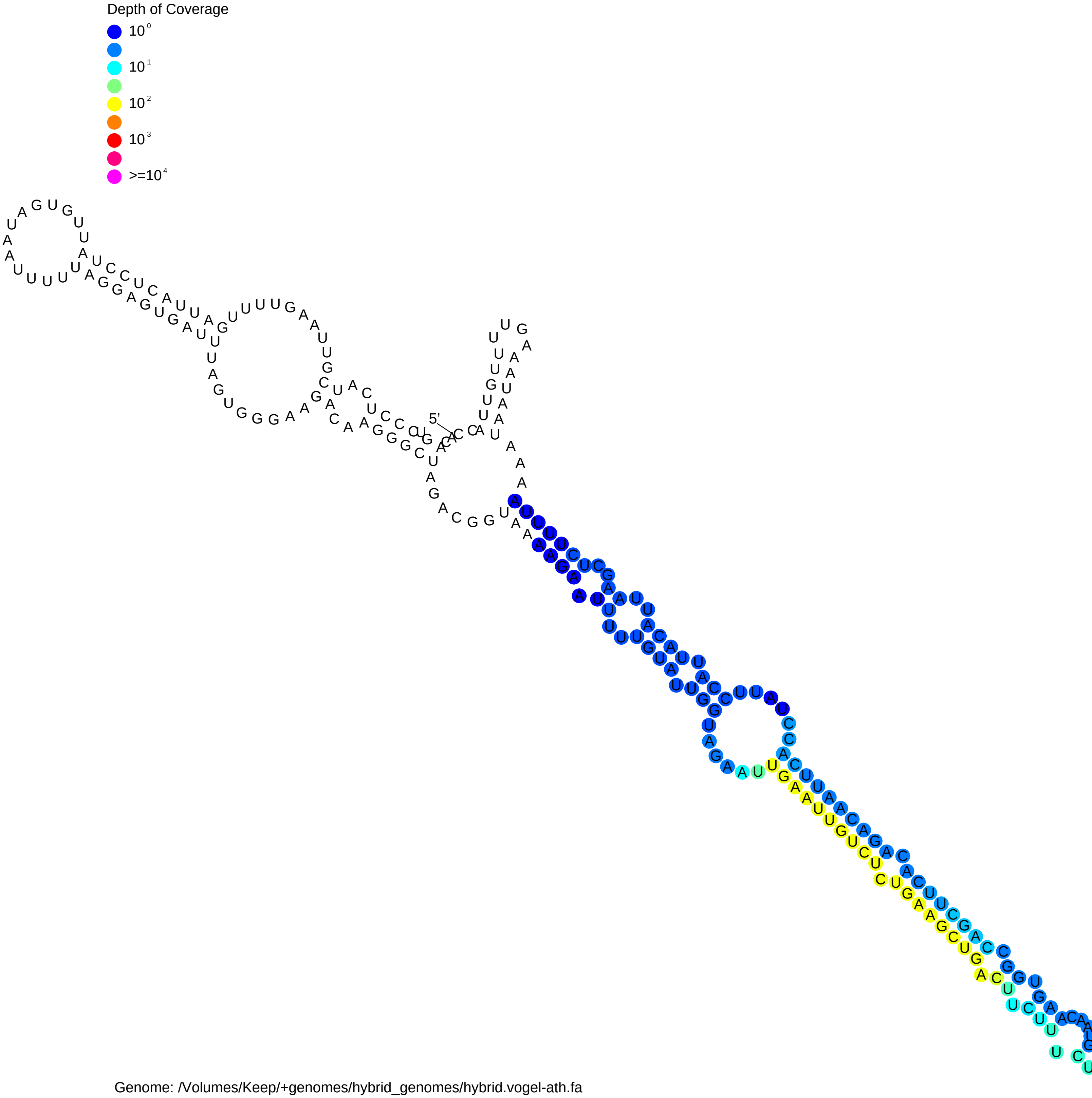

Genome: /Volumes/Keep/+genomes/hybrid_genomes/hybrid.vogel-ath.fa
Alignments: /Volumes/Keep/+Cuscuta_evolution/NextSeq_Sep2018_sRNAseq/ccm-denovo_sRNA_annotation/02out-cca.hybrid.ShortStack/merged_alig
Location: Ccam0.32_scaffold502:43425-43667 plus
Name: SFs=SupFam_65, SSCl=Cluster_160809, MIRNA=Y

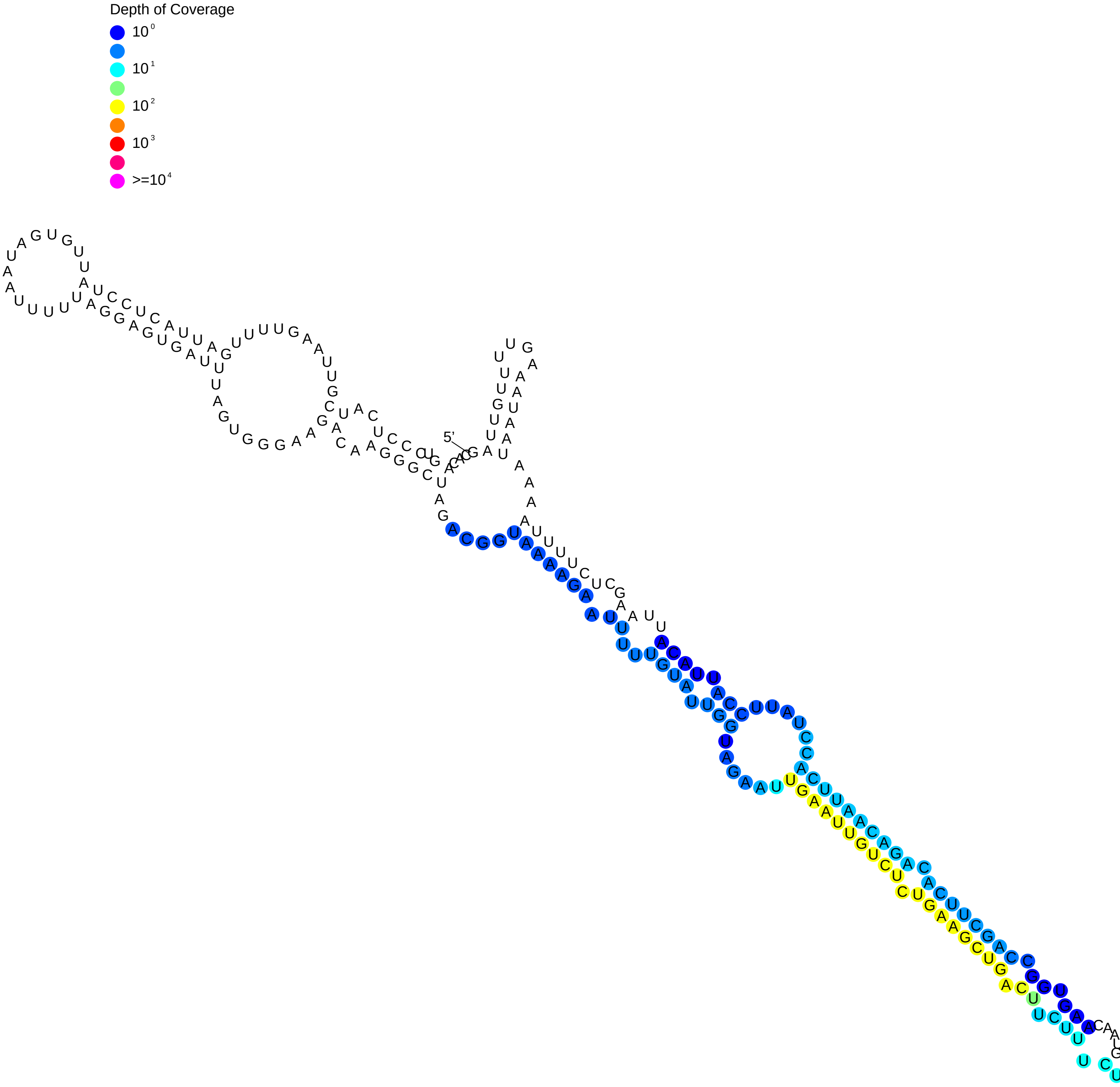

Genome: /Volumes/Keep/+genomes/hybrid_genomes/hybrid.vogel-ath.fa
Alignments: /Volumes/Keep/+Cuscuta_evolution/NextSeq_Sep2018_sRNAseq/ccm-denovo_sRNA_annotation/02out-cca.hybrid.ShortStack/merged_alig
Location: Ccam0.32_scaffold502:67624-67866 minus
Name: SFs=SupFam_65, SSCl=Cluster_160810, MIRNA=Y

Depth of Coverage

- 10^0
-
- 10^1
-
- 10^2
-
- 10^3
-
- $>=10^4$

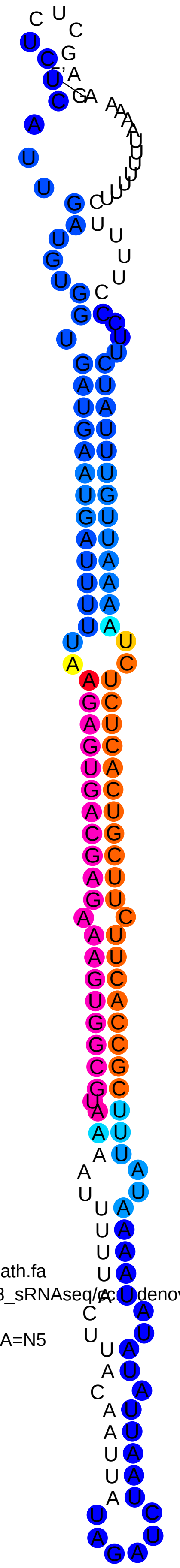

Genome: /Volumes/Keep/+genomes/hybrid_genomes/hybrid.vogel-ath.fa
Alignments: /Volumes/Keep/+Cuscuta_evolution/NextSeq_Sep2018_sRNAseq/c[illegible]denovo_sRNA_annotation/02out-cca.hybrid.ShortStack/merged_alig
Location: Ccam0.32_scaffold11:906201-906360 minus
Name: SFs=SupFam_73,SupFam_12, SSCl=Cluster_16821, MIRNA=N5

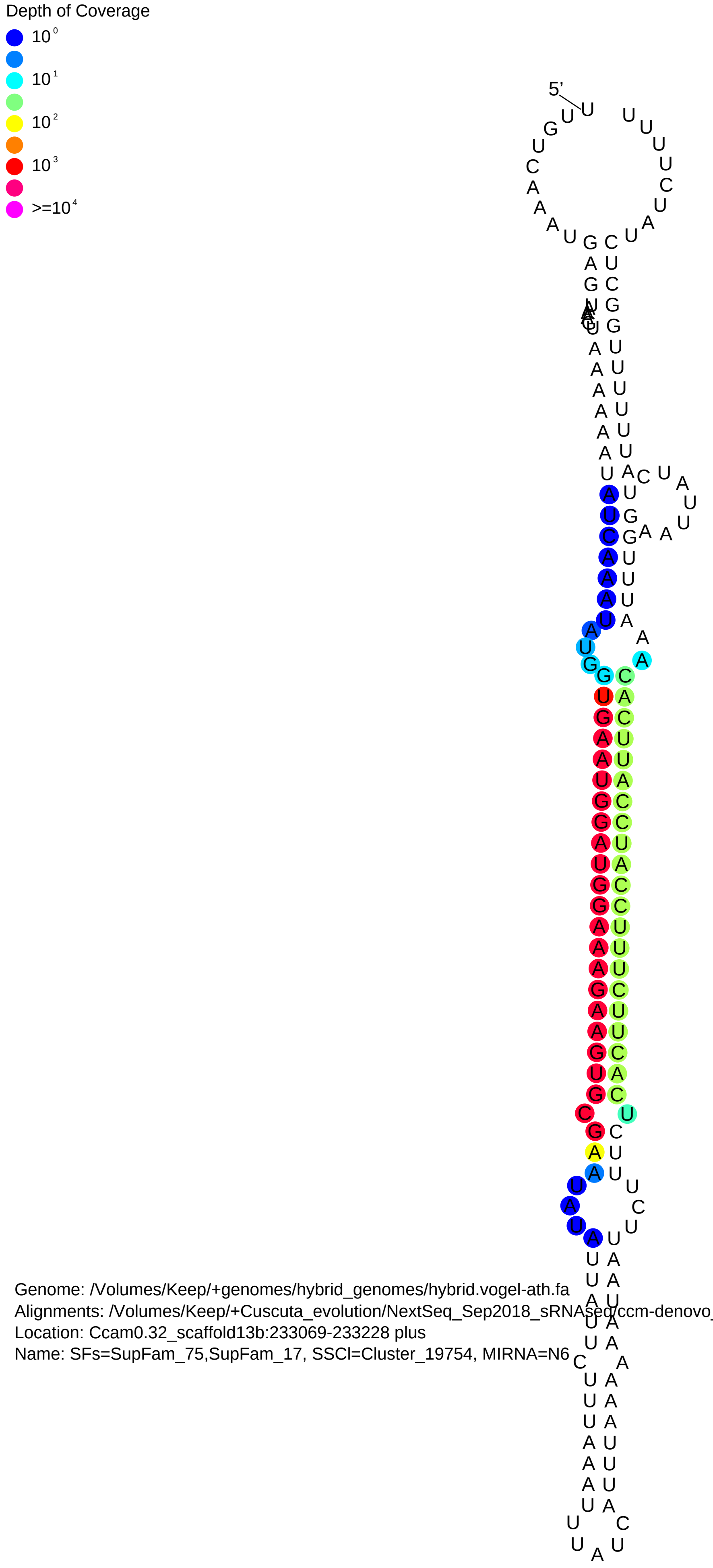

Genome: /Volumes/Keep/+genomes/hybrid_genomes/hybrid.vogel-ath.fa
Alignments: /Volumes/Keep/+Cuscuta_evolution/NextSeq_Sep2018_sRNAseq/ccm-denovo_sRNA_annotation/02out-cca.hybrid.ShortStack/merged_alig
Location: Ccam0.32_scaffold13b:233069-233228 plus
Name: SFs=SupFam_75,SupFam_17, SSCl=Cluster_19754, MIRNA=N6

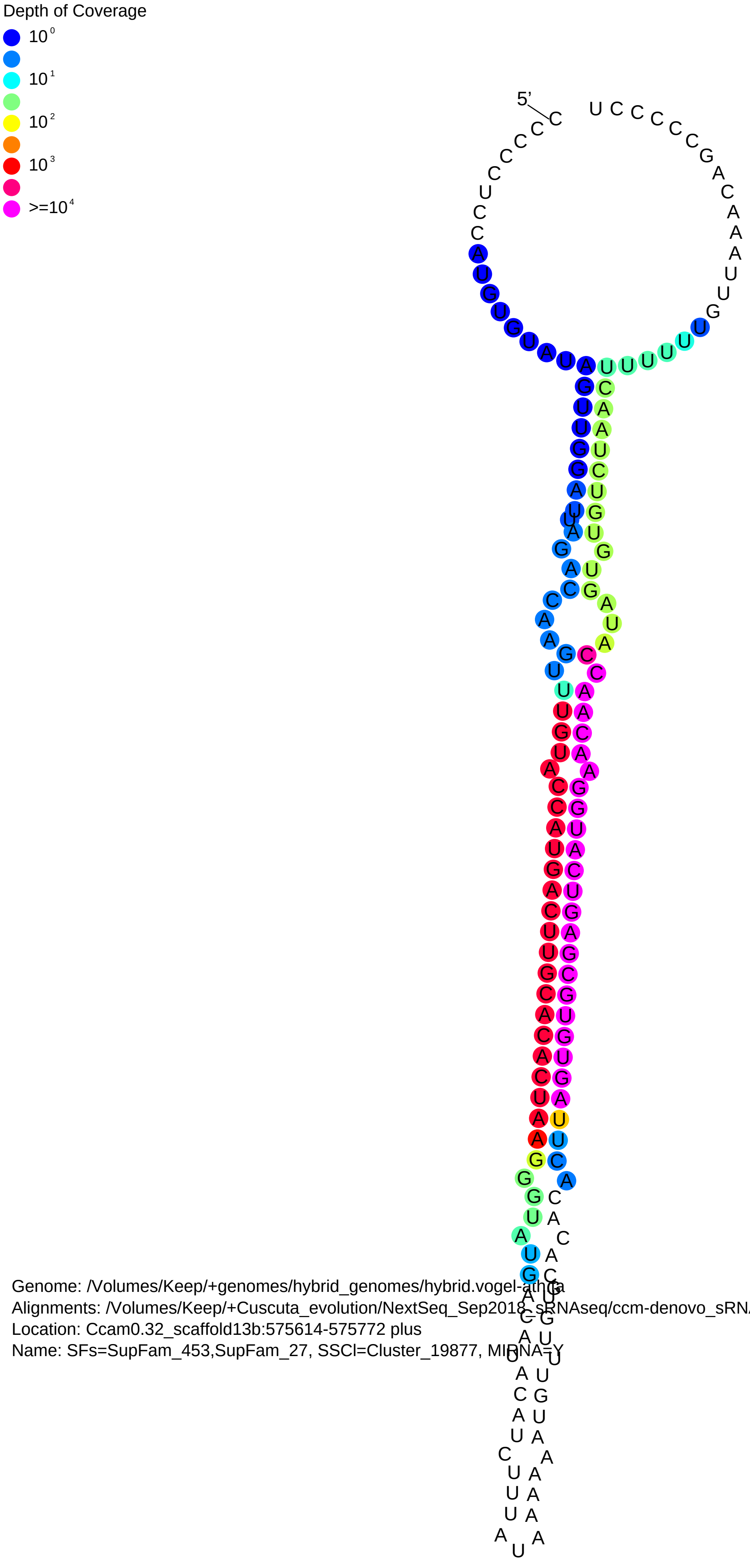

Genome: /Volumes/Keep/+genomes/hybrid_genomes/hybrid.vogel-ath.fa
Alignments: /Volumes/Keep/+Cuscuta_evolution/NextSeq_Sep2018_sRNAseq/ccm-denovo_sRNA_annotation/02out-cca.hybrid.ShortStack/merged_alig
Location: Ccam0.32_scaffold13b:575614-575772 plus
Name: SFs=SupFam_453,SupFam_27, SSCl=Cluster_19877, MIRNA=Y

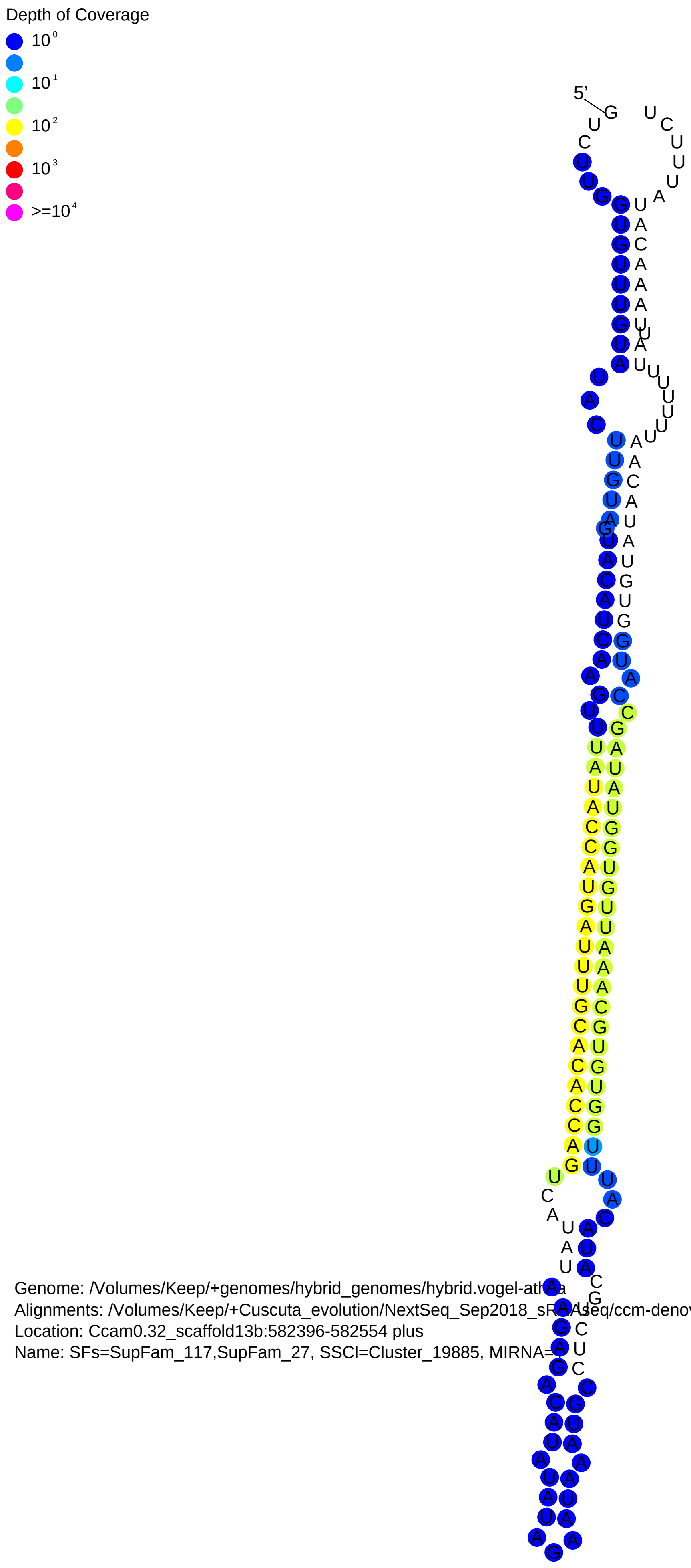

Genome: /Volumes/Keep/+genomes/hybrid_genomes/hybrid.vogel-ath[illegible]a
Alignments: /Volumes/Keep/+Cuscuta_evolution/NextSeq_Sep2018_sR[illegible]Aseq/ccm-denovo_sRNA_annotation/02out-cca.hybrid.ShortStack/merged_alig
Location: Ccam0.32_scaffold13b:582396-582554 plus
Name: SFs=SupFam_117,SupFam_27, SSCl=Cluster_19885, MIRNA=[illegible]

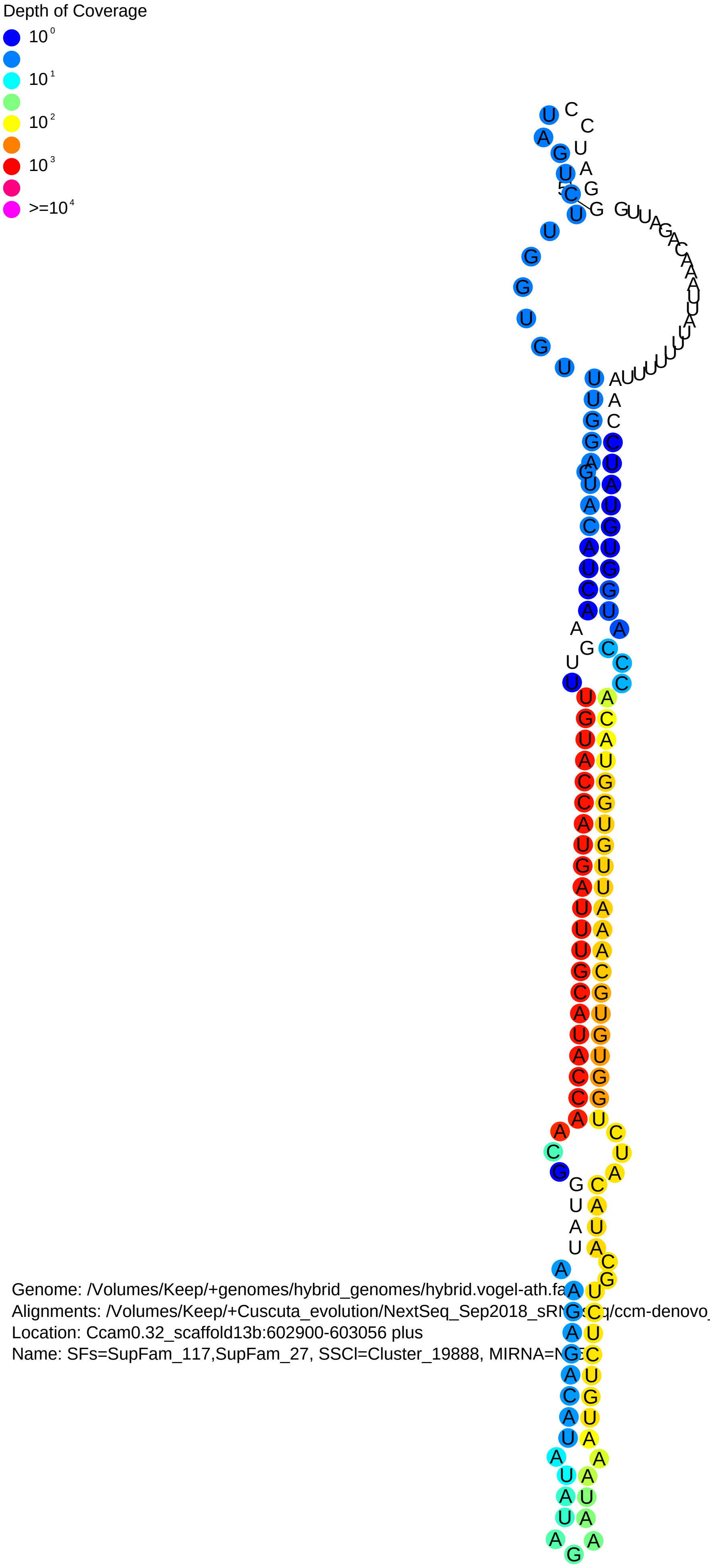

Genome: /Volumes/Keep/+genomes/hybrid_genomes/hybrid.vogel-ath.fa[illegible]
Alignments: /Volumes/Keep/+Cuscuta_evolution/NextSeq_Sep2018_sR[illegible]q/ccm-denovo_sRNA_annotation/02out-cca.hybrid.ShortStack/merged_alig
Location: Ccam0.32_scaffold13b:602900-603056 plus
Name: SFs=SupFam_117,SupFam_27, SSCl=Cluster_19888, MIRNA=N[illegible]

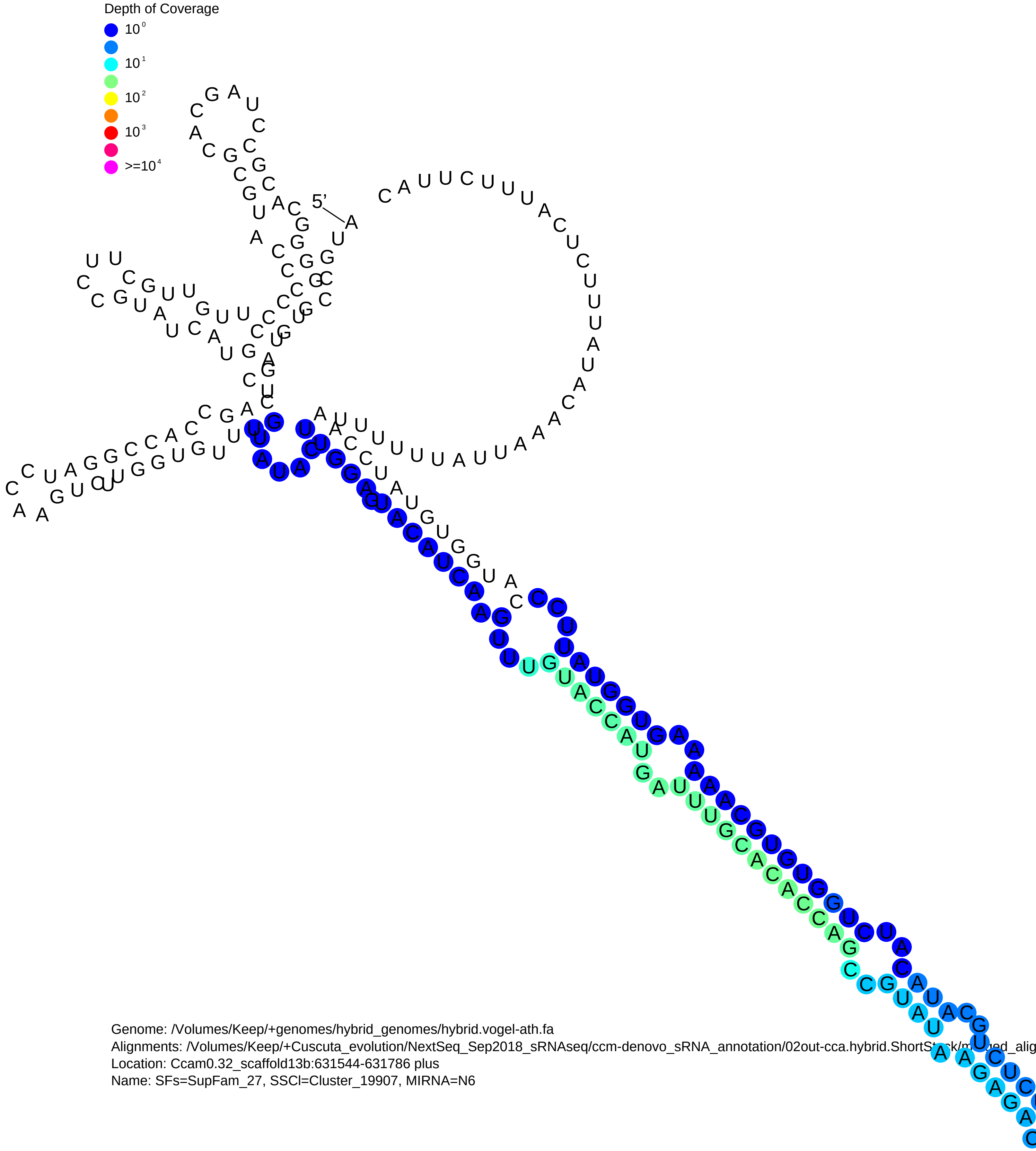

Genome: /Volumes/Keep/+genomes/hybrid_genomes/hybrid.vogel-ath.fa
Alignments: /Volumes/Keep/+Cuscuta_evolution/NextSeq_Sep2018_sRNAseq/ccm-denovo_sRNA_annotation/02out-cca.hybrid.ShortSt[illegible]
Location: Ccam0.32_scaffold13b:631544-631786 plus
Name: SFs=SupFam_27, SSCl=Cluster_19907, MIRNA=N6

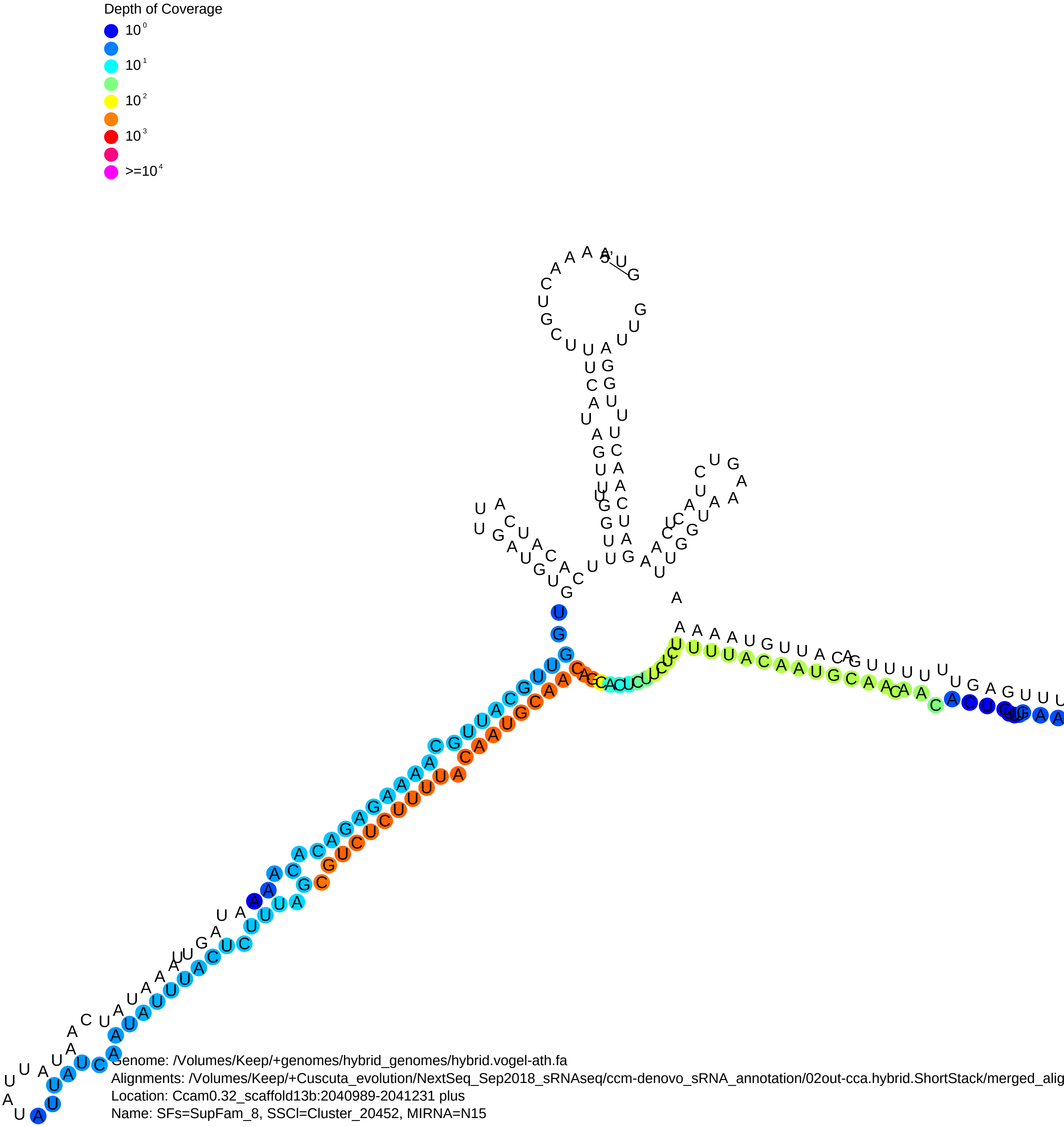

Genome: /Volumes/Keep/+genomes/hybrid_genomes/hybrid.vogel-ath.fa
Alignments: /Volumes/Keep/+Cuscuta_evolution/NextSeq_Sep2018_sRNAseq/ccm-denovo_sRNA_annotation/02out-cca.hybrid.ShortStack/merged_alig
Location: Ccam0.32_scaffold13b:2040989-2041231 plus
Name: SFs=SupFam_8, SSCl=Cluster_20452, MIRNA=N15

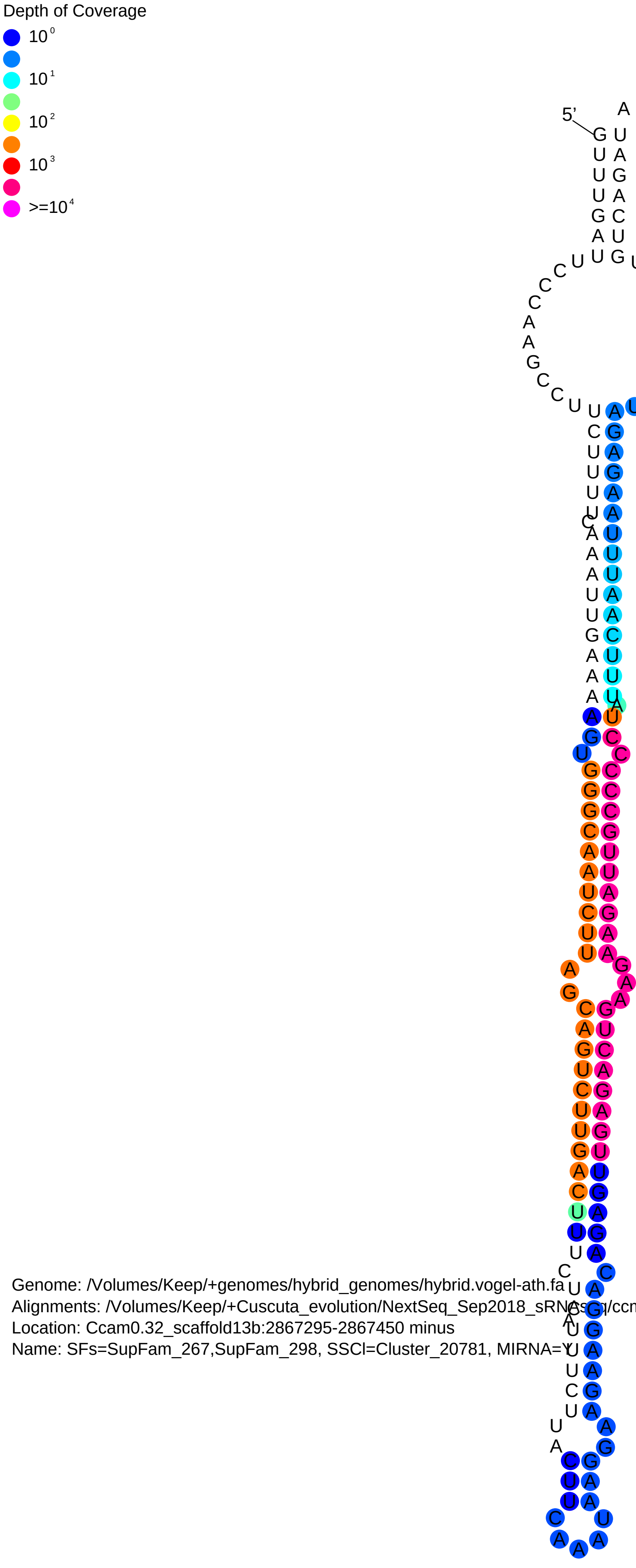

Genome: /Volumes/Keep/+genomes/hybrid_genomes/hybrid.vogel-ath.fa
Alignments: /Volumes/Keep/+Cuscuta_evolution/NextSeq_Sep2018_sRNAseq/ccm-denovo_sRNA_annotation/02out-cca.hybrid.ShortStack/merged_alig
Location: Ccam0.32_scaffold13b:2867295-2867450 minus
Name: SFs=SupFam_267,SupFam_298, SSCl=Cluster_20781, MIRNA=Y

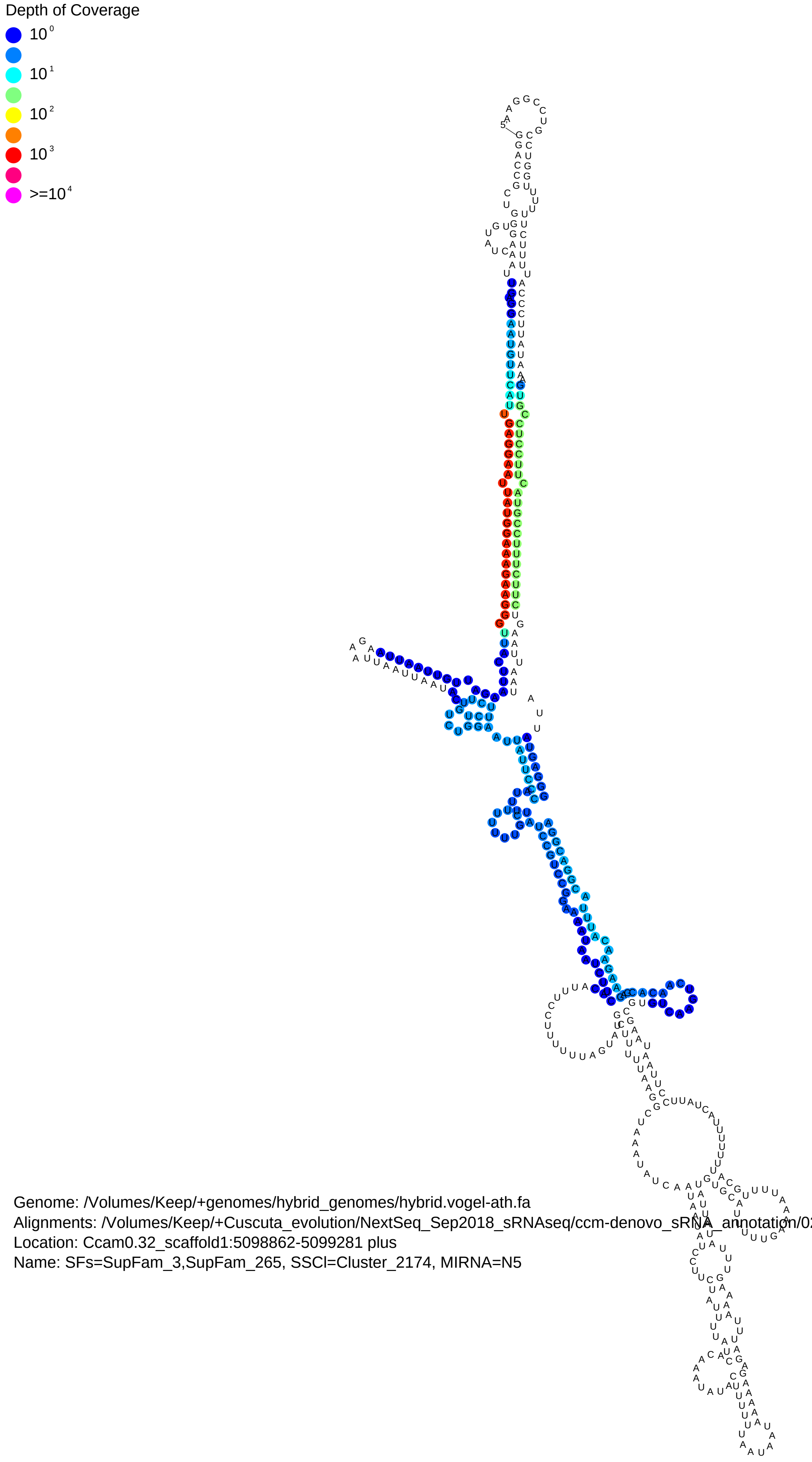

Genome: /Volumes/Keep/+genomes/hybrid_genomes/hybrid.vogel-ath.fa
Alignments: /Volumes/Keep/+Cuscuta_evolution/NextSeq_Sep2018_sRNAseq/ccm-denovo_sRNA_annotation/02out-cca.hybrid.ShortStack/merged_alig
Location: Ccam0.32_scaffold1:5098862-5099281 plus
Name: SFs=SupFam_3,SupFam_265, SSCl=Cluster_2174, MIRNA=N5

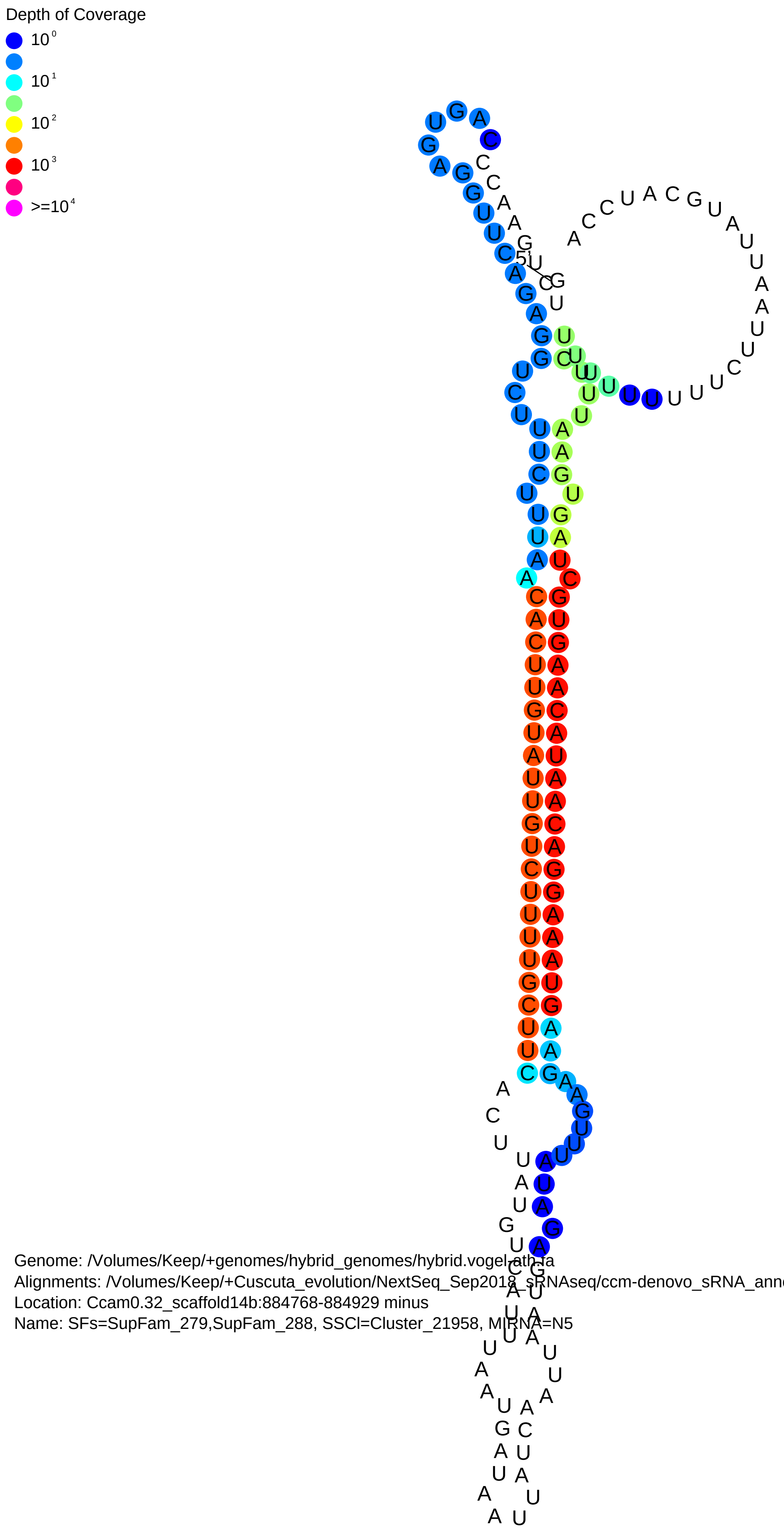

Genome: /Volumes/Keep/+genomes/hybrid_genomes/hybrid.vogel.ath.fa
Alignments: /Volumes/Keep/+Cuscuta_evolution/NextSeq_Sep2018_sRNAseq/ccm-denovo_sRNA_annotation/02out-cca.hybrid.ShortStack/merged_alig
Location: Ccam0.32_scaffold14b:884768-884929 minus
Name: SFs=SupFam_279,SupFam_288, SSCl=Cluster_21958, MIRNA=N5

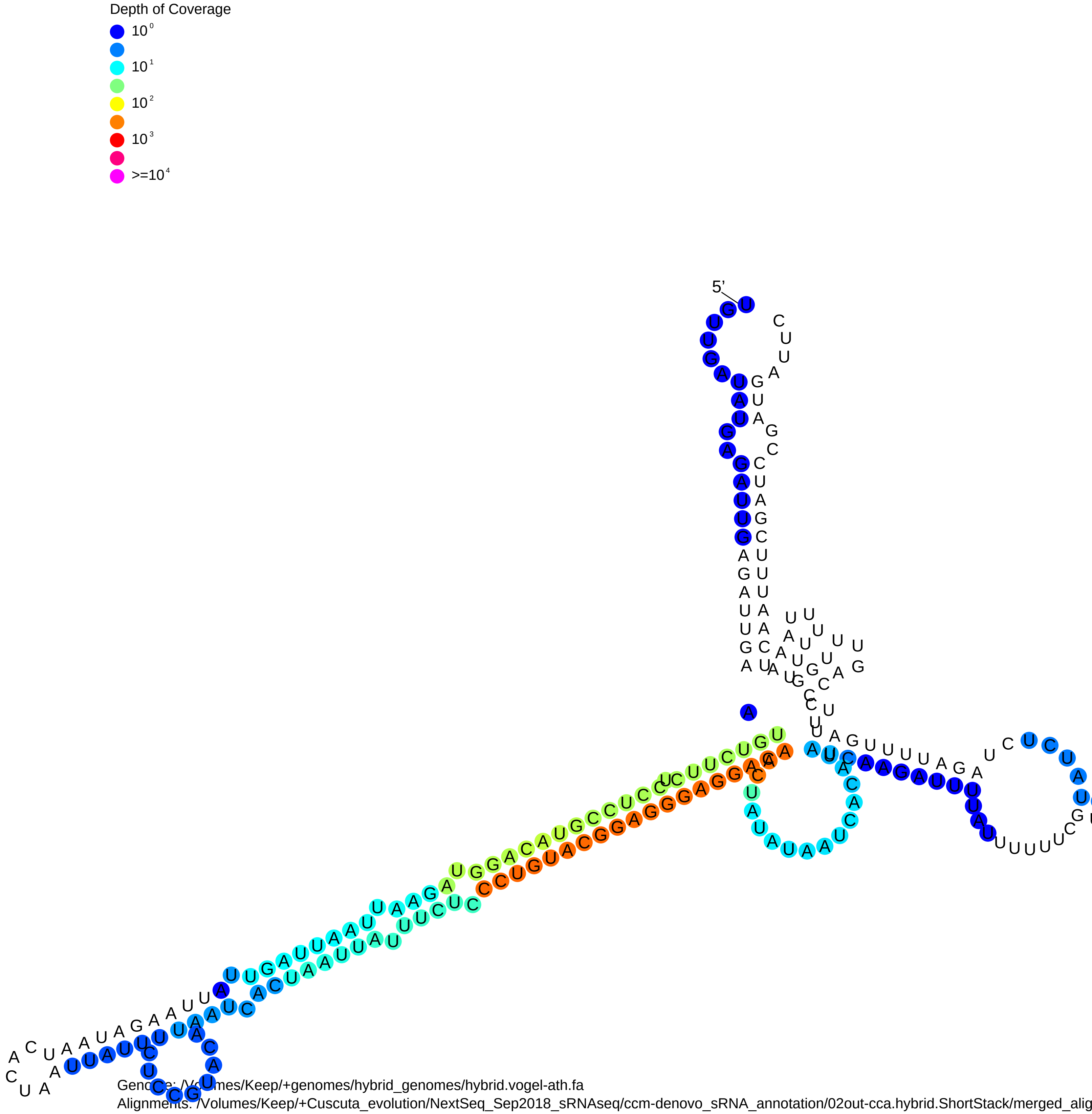

Genome: /Volumes/Keep/+genomes/hybrid_genomes/hybrid.vogel-ath.fa
Alignments: /Volumes/Keep/+Cuscuta_evolution/NextSeq_Sep2018_sRNAseq/ccm-denovo_sRNA_annotation/02out-cca.hybrid.ShortStack/merged_alig
Location: Ccam0.32_scaffold14b:1771194-1771435 plus
Name: SFs=SupFam_40, SSCl=Cluster_22371, MIRNA=N5

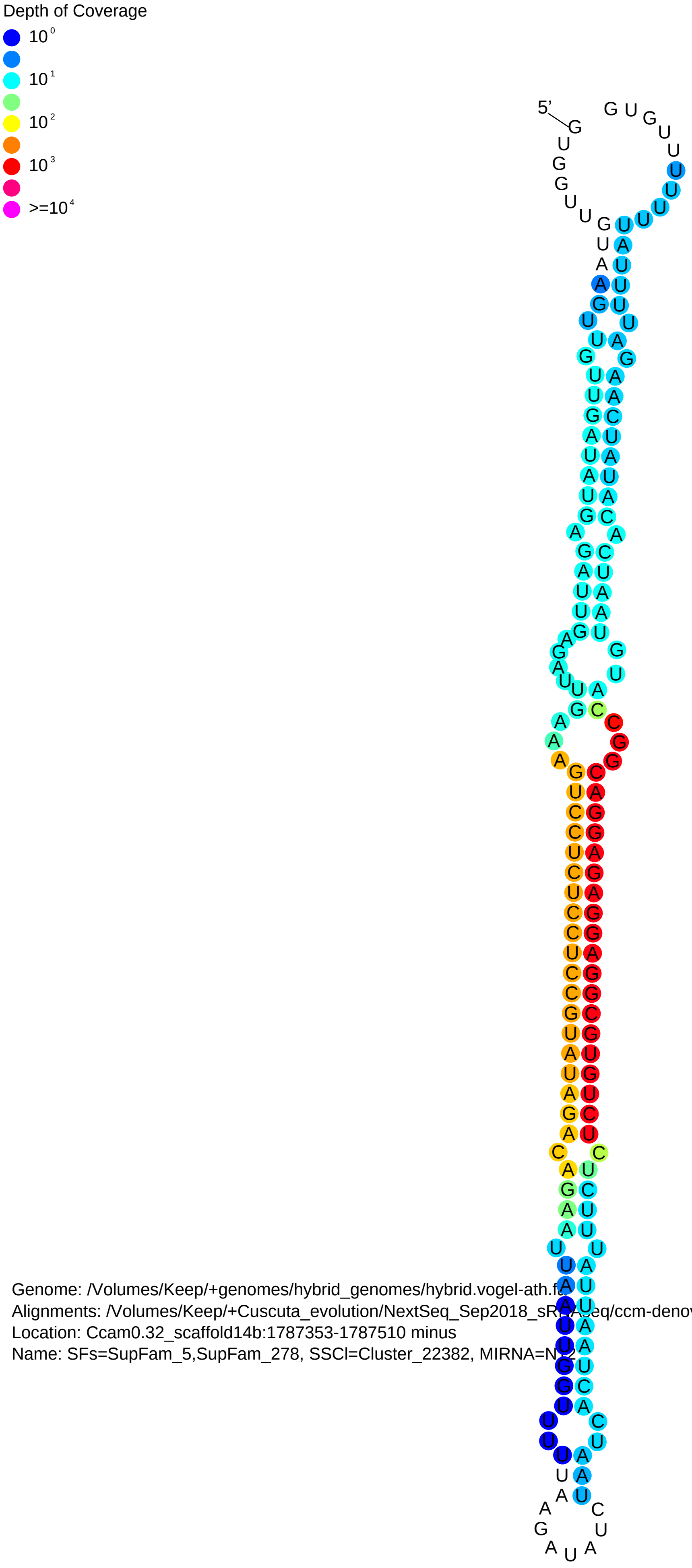

Genome: /Volumes/Keep/+genomes/hybrid_genomes/hybrid.vogel-ath.f[illegible]
Alignments: /Volumes/Keep/+Cuscuta_evolution/NextSeq_Sep2018_sR[illegible]eq/ccm-denovo_sRNA_annotation/02out-cca.hybrid.ShortStack/merged_alig
Location: Ccam0.32_scaffold14b:1787353-1787510 minus
Name: SFs=SupFam_5,SupFam_278, SSCl=Cluster_22382, MIRNA=N[illegible]

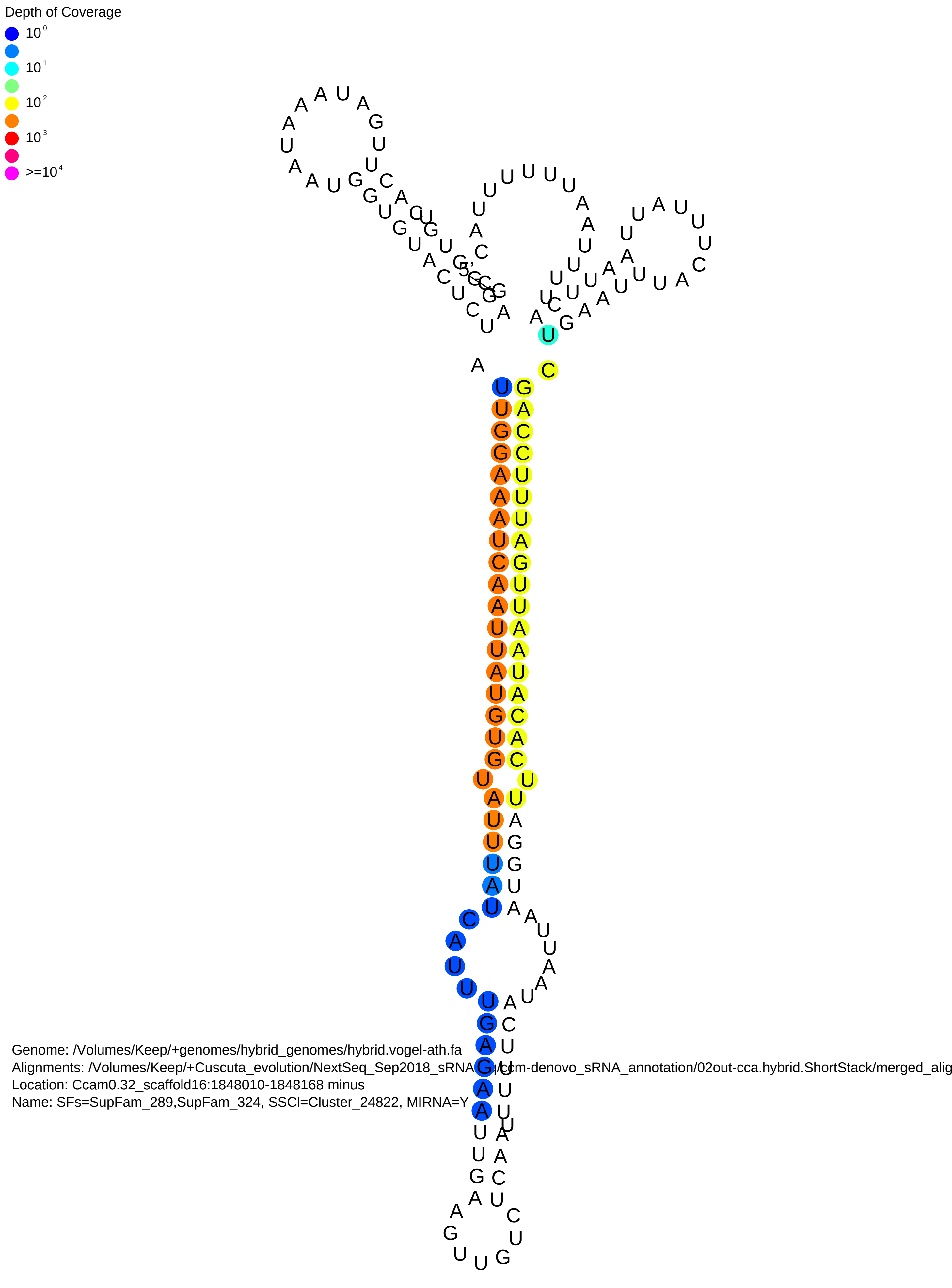

Genome: /Volumes/Keep/+genomes/hybrid_genomes/hybrid.vogel-ath.fa
Alignments: /Volumes/Keep/+Cuscuta_evolution/NextSeq_Sep2018_sRNA[illegible]/ccm-denovo_sRNA_annotation/02out-cca.hybrid.ShortStack/merged_alig
Location: Ccam0.32_scaffold16:1848010-1848168 minus
Name: SFs=SupFam_289,SupFam_324, SSCl=Cluster_24822, MIRNA=Y

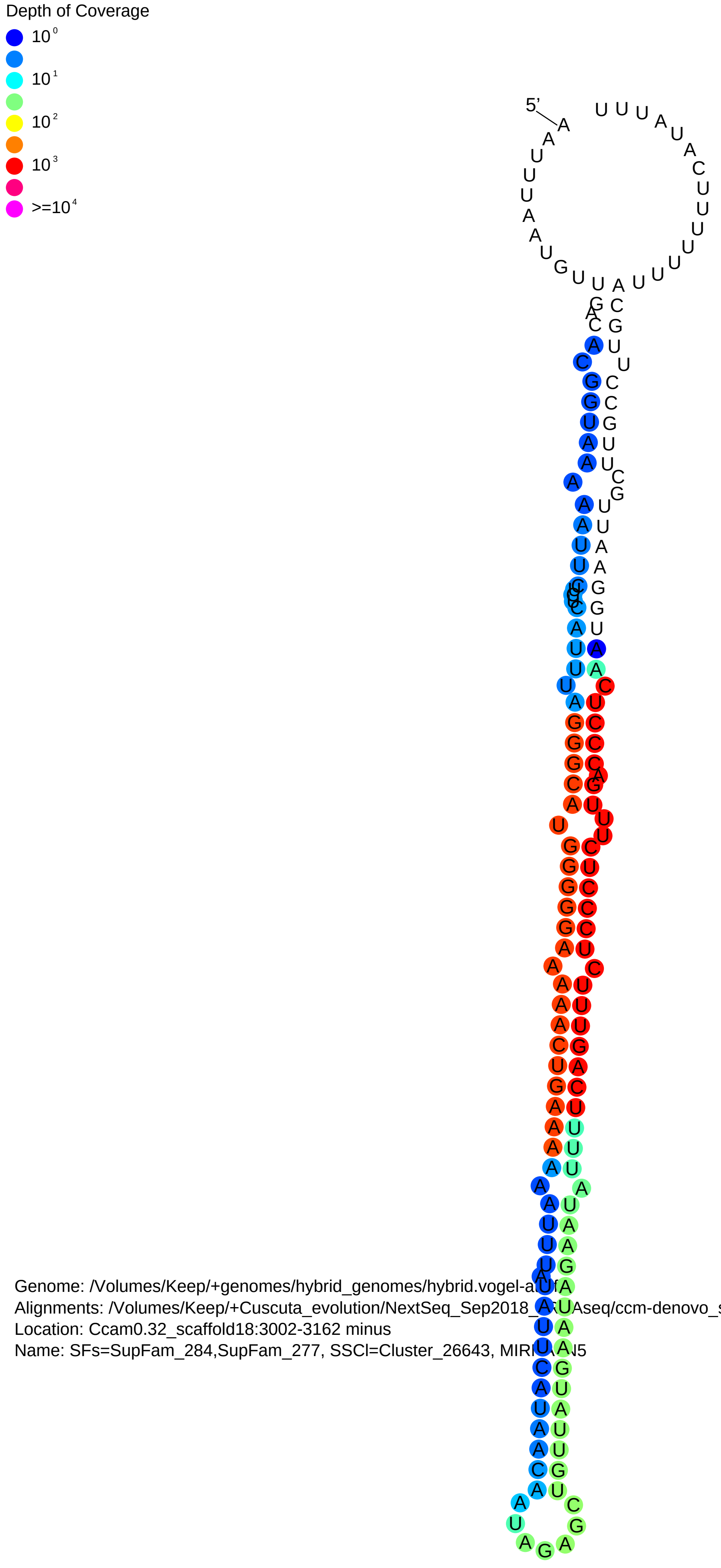

Genome: /Volumes/Keep/+genomes/hybrid_genomes/hybrid.vogel-a[illegible]
Alignments: /Volumes/Keep/+Cuscuta_evolution/NextSeq_Sep2018_[illegible]Aseq/ccm-denovo_sRNA_annotation/02out-cca.hybrid.ShortStack/merged_alig
Location: Ccam0.32_scaffold18:3002-3162 minus
Name: SFs=SupFam_284,SupFam_277, SSCl=Cluster_26643, MIR[illegible]N5

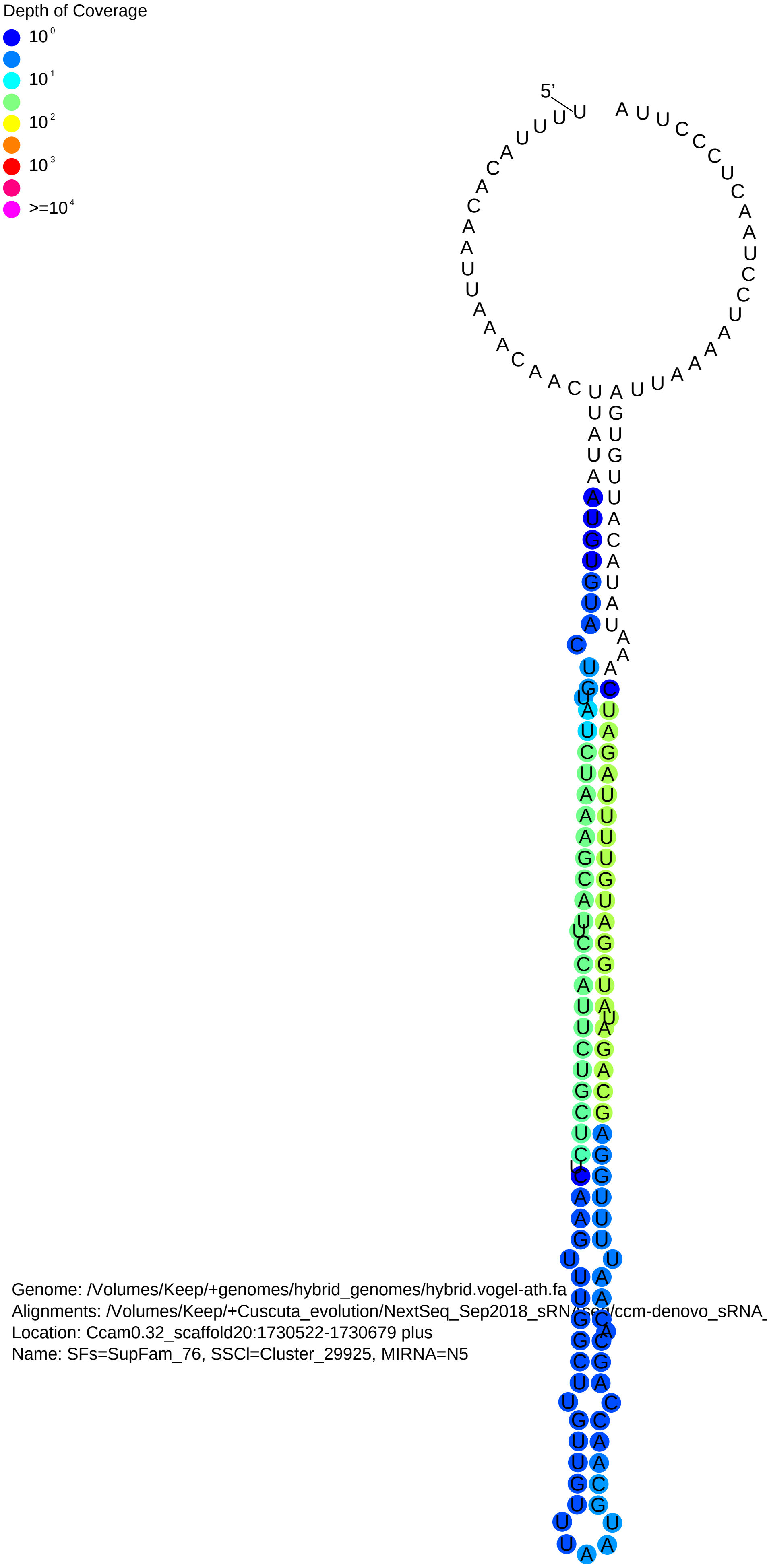

Genome: /Volumes/Keep/+genomes/hybrid_genomes/hybrid.vogel-ath.fa
Alignments: /Volumes/Keep/+Cuscuta_evolution/NextSeq_Sep2018_sRN[illegible]/ccm-denovo_sRNA_annotation/02out-cca.hybrid.ShortStack/merged_alig
Location: Ccam0.32_scaffold20:1730522-1730679 plus
Name: SFs=SupFam_76, SSCl=Cluster_29925, MIRNA=N5

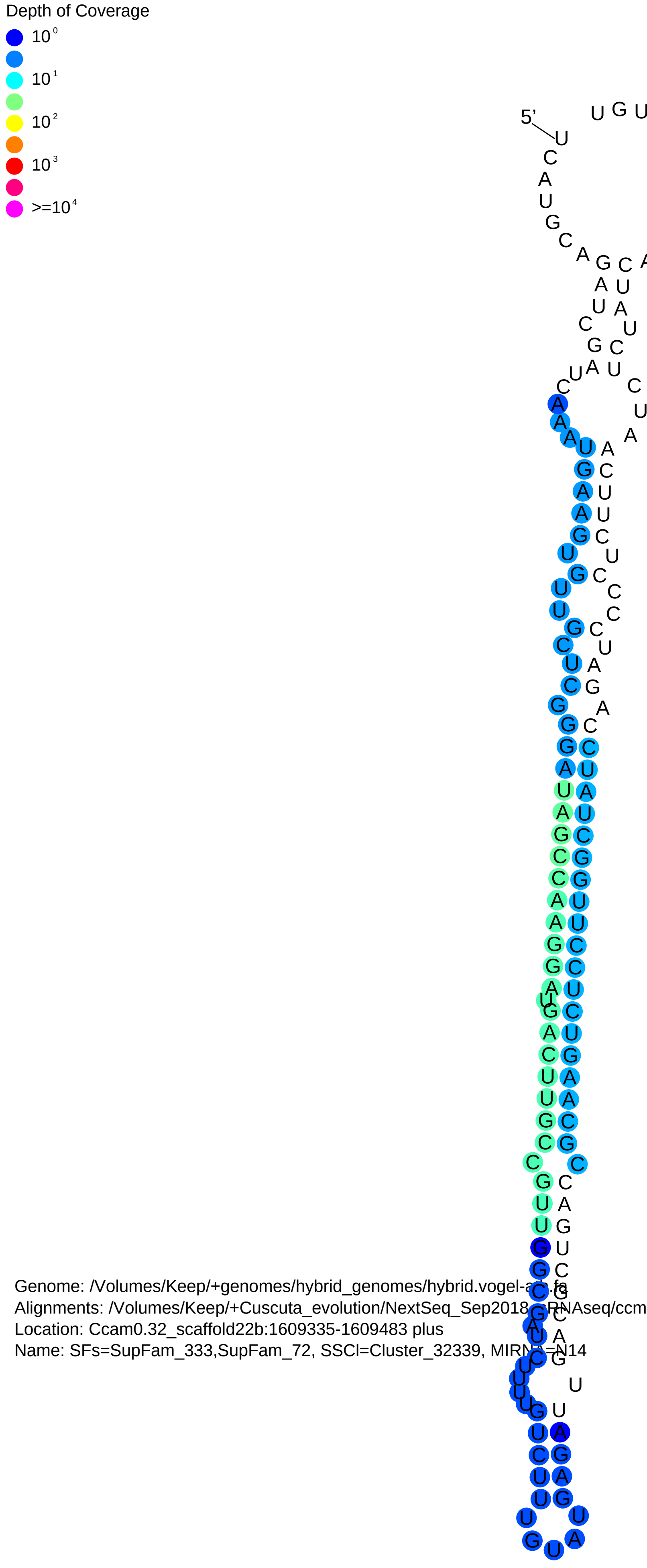

Genome: /Volumes/Keep/+genomes/hybrid_genomes/hybrid.vogel-a[illegible].fa
Alignments: /Volumes/Keep/+Cuscuta_evolution/NextSeq_Sep2018[illegible]RNAseq/ccm-denovo_sRNA_annotation/02out-cca.hybrid.ShortStack/merged_alig
Location: Ccam0.32_scaffold22b:1609335-1609483 plus
Name: SFs=SupFam_333,SupFam_72, SSCl=Cluster_32339, MIR[illegible]A=N14

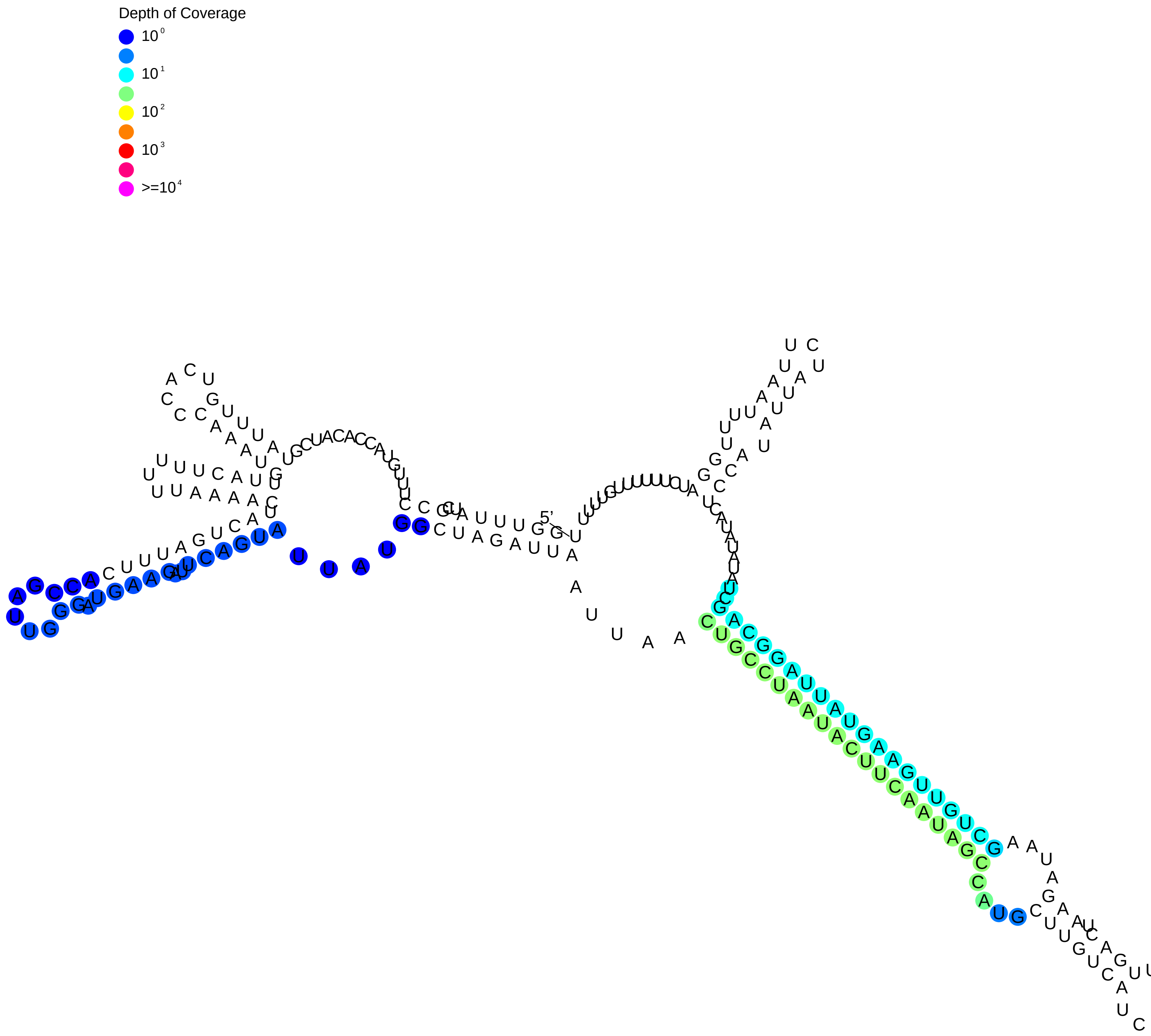

Genome: /Volumes/Keep/+genomes/hybrid_genomes/hybrid.vogel-ath.fa
Alignments: /Volumes/Keep/+Cuscuta_evolution/NextSeq_Sep2018_sRNAseq/ccm-denovo_sRNA_annotation/02out-cca.hybrid.ShortStack/merged_alig
Location: Ccam0.32_scaffold23:330582-330824 minus
Name: SFs=SupFam_342, SSCl=Cluster_32905, MIRNA=Y

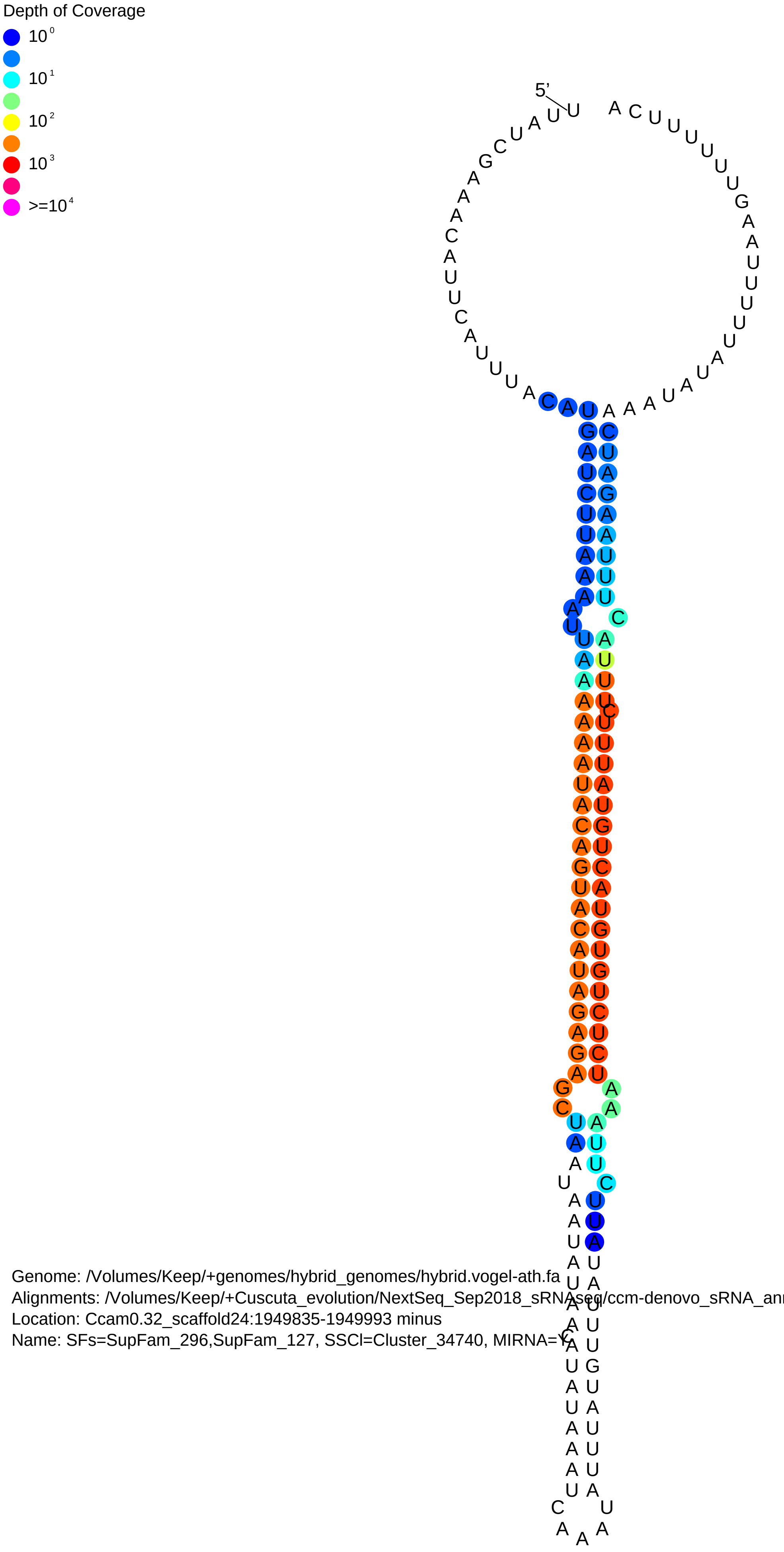

Genome: /Volumes/Keep/+genomes/hybrid_genomes/hybrid.vogel-ath.fa
Alignments: /Volumes/Keep/+Cuscuta_evolution/NextSeq_Sep2018_sRNAseq/ccm-denovo_sRNA_annotation/02out-cca.hybrid.ShortStack/merged_alig
Location: Ccam0.32_scaffold24:1949835-1949993 minus
Name: SFs=SupFam_296,SupFam_127, SSCl=Cluster_34740, MIRNA=Y

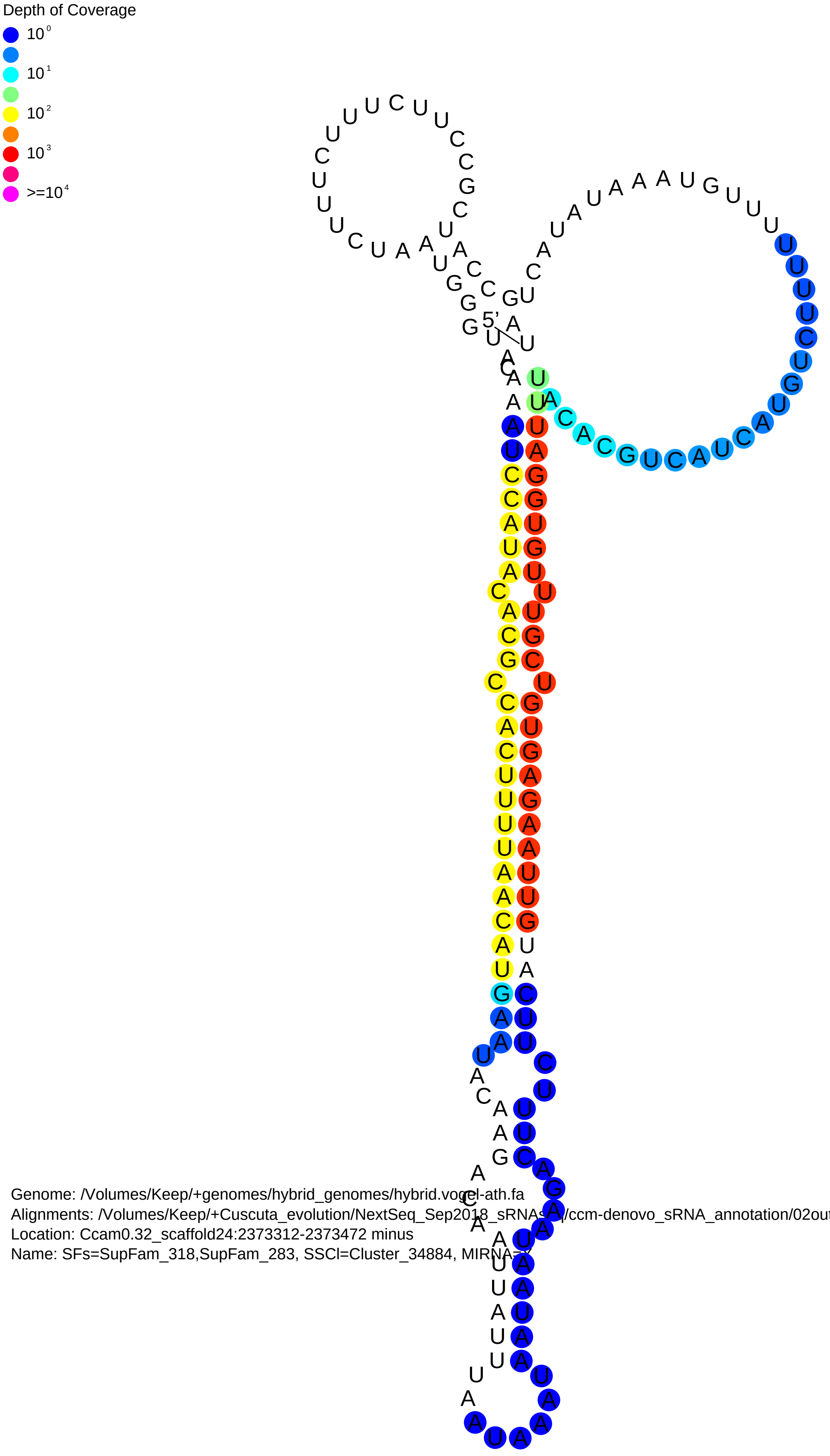

Genome: /Volumes/Keep/+genomes/hybrid_genomes/hybrid.vogel-ath.fa
Alignments: /Volumes/Keep/+Cuscuta_evolution/NextSeq_Sep2018_sRNAs[illegible]/ccm-denovo_sRNA_annotation/02out-cca.hybrid.ShortStack/merged_alig
Location: Ccam0.32_scaffold24:2373312-2373472 minus
Name: SFs=SupFam_318,SupFam_283, SSCl=Cluster_34884, MIRNA[illegible]

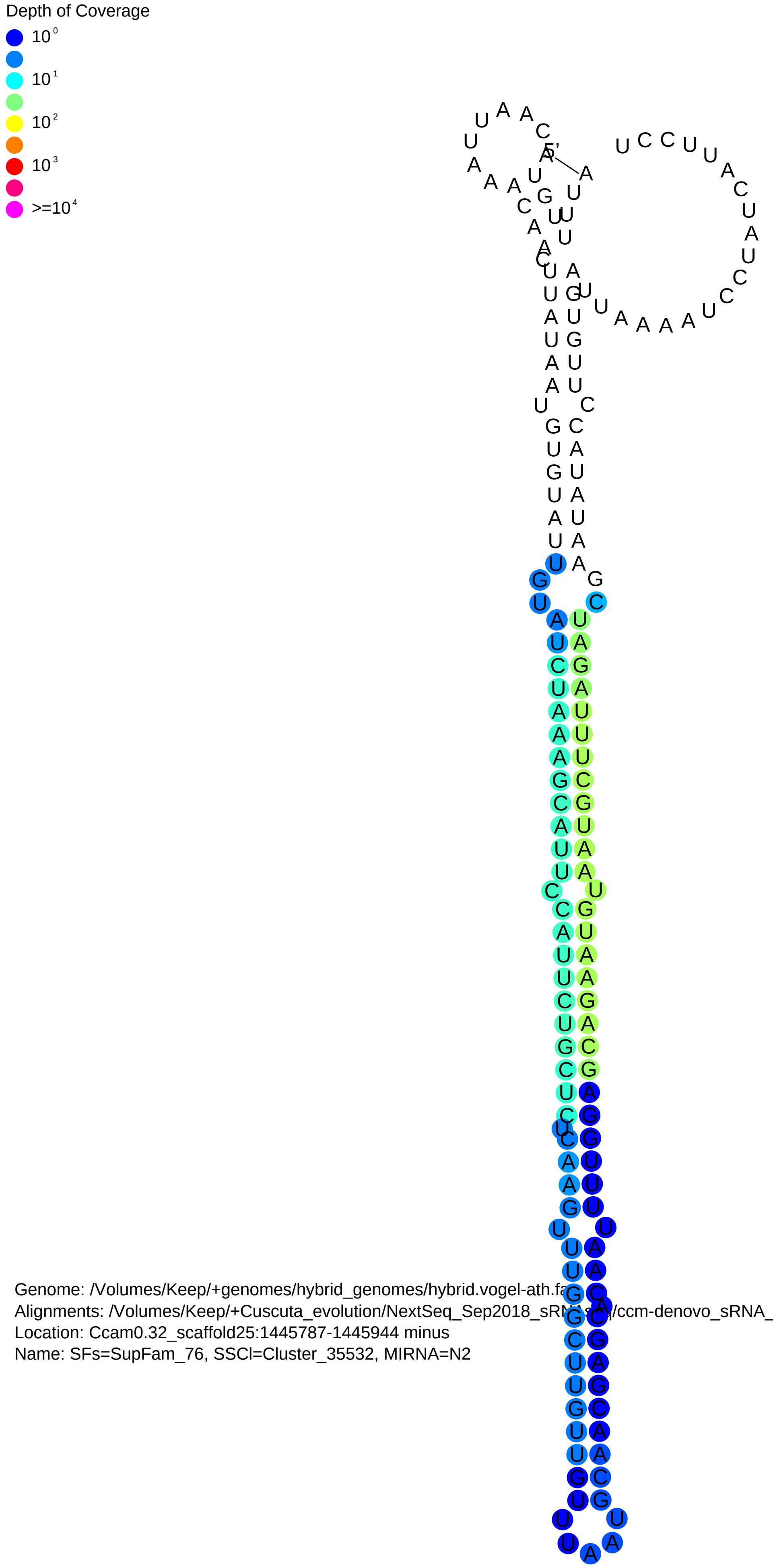

Genome: /Volumes/Keep/+genomes/hybrid_genomes/hybrid.vogel-ath.fa[illegible]
Alignments: /Volumes/Keep/+Cuscuta_evolution/NextSeq_Sep2018_sR[illegible]/ccm-denovo_sRNA_annotation/02out-cca.hybrid.ShortStack/merged_alig
Location: Ccam0.32_scaffold25:1445787-1445944 minus
Name: SFs=SupFam_76, SSCl=Cluster_35532, MIRNA=N2

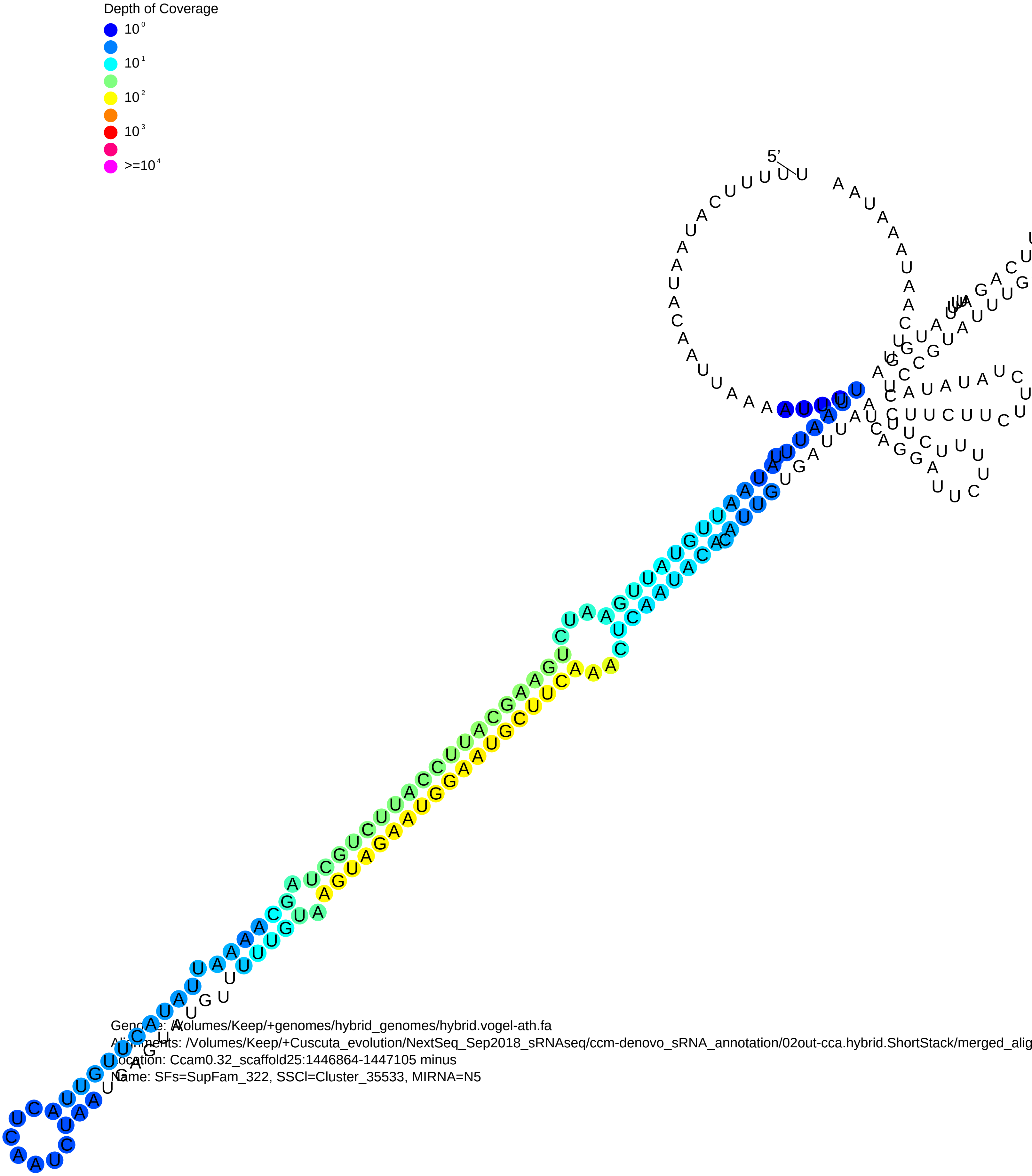

Genome: /Volumes/Keep/+genomes/hybrid_genomes/hybrid.vogel-ath.fa
Alignments: /Volumes/Keep/+Cuscuta_evolution/NextSeq_Sep2018_sRNAseq/ccm-denovo_sRNA_annotation/02out-cca.hybrid.ShortStack/merged_alig
Location: Ccam0.32_scaffold25:1446864-1447105 minus
Name: SFs=SupFam_322, SSCl=Cluster_35533, MIRNA=N5

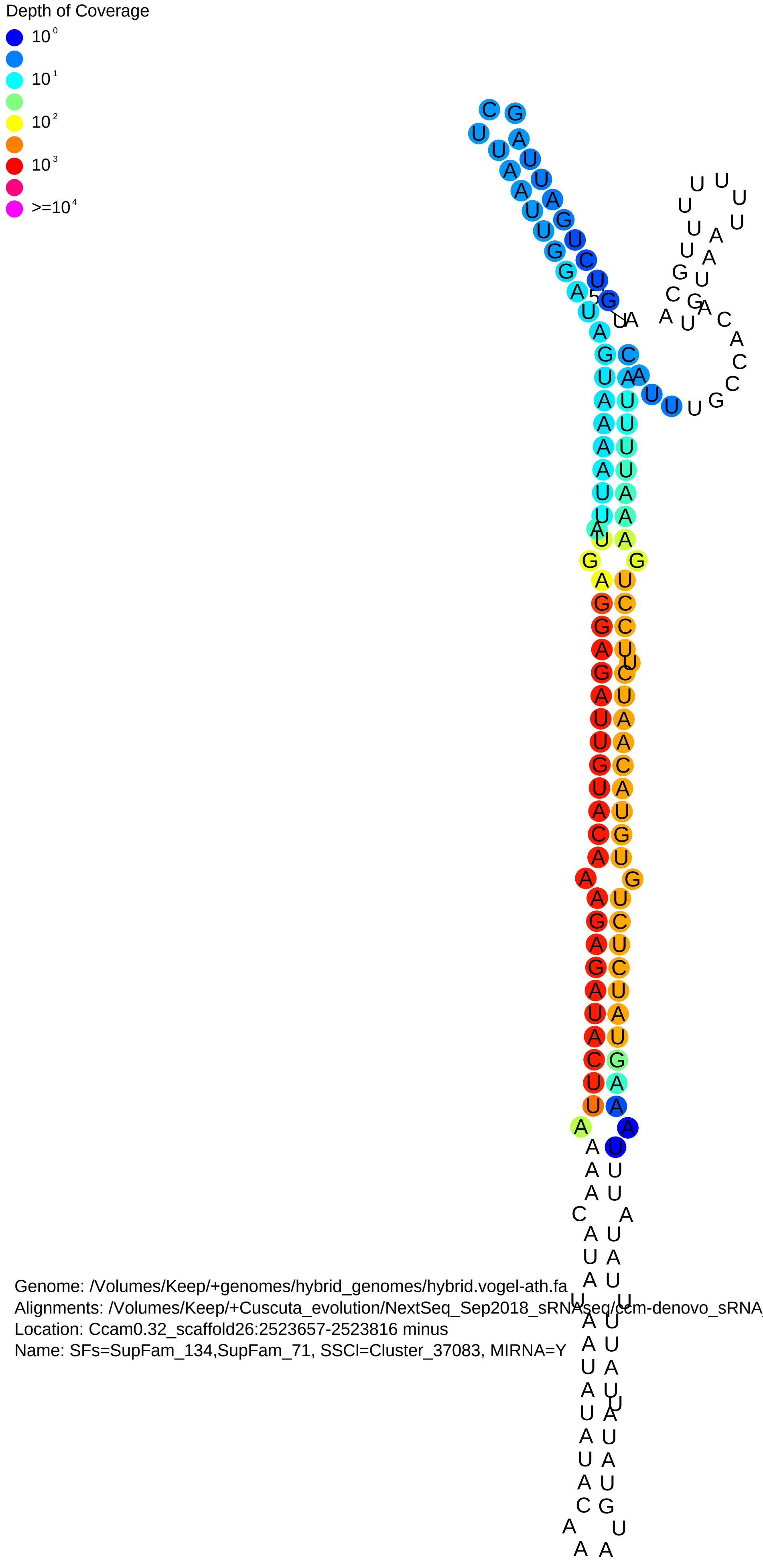

Genome: /Volumes/Keep/+genomes/hybrid_genomes/hybrid.vogel-ath.fa
Alignments: /Volumes/Keep/+Cuscuta_evolution/NextSeq_Sep2018_sRNAseq/ccm-denovo_sRNA_annotation/02out-cca.hybrid.ShortStack/merged_alig
Location: Ccam0.32_scaffold26:2523657-2523816 minus
Name: SFs=SupFam_134,SupFam_71, SSCl=Cluster_37083, MIRNA=Y

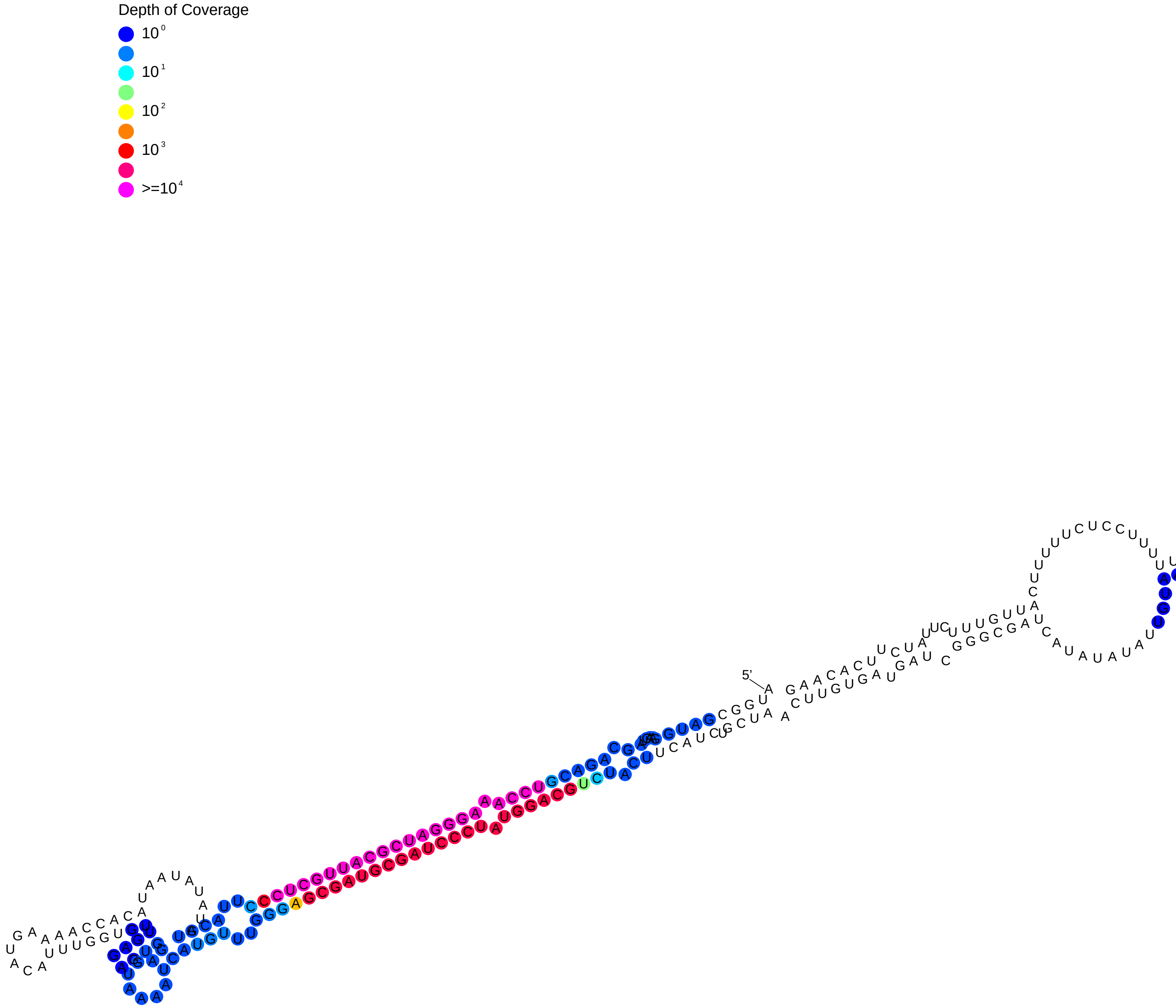

Genome: /Volumes/Keep/+genomes/hybrid_genomes/hybrid.vogel-ath.fa
Alignments: /Volumes/Keep/+Cuscuta_evolution/NextSeq_Sep2018_sRNAseq/ccm-denovo_sRNA_annotation/02out-cca.hybrid.ShortStack/merged_alig
Location: Ccam0.32_scaffold29:1201865-1202106 plus
Name: SFs=SupFam_13, SSCl=Cluster_39569, MIRNA=Y

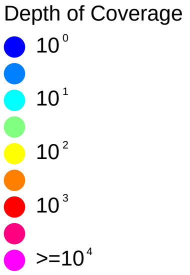

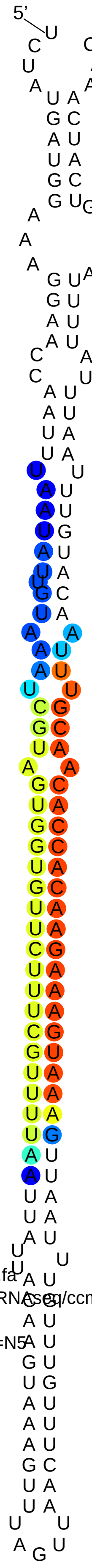

Genome: /Volumes/Keep/+genomes/hybrid_genomes/hybrid.vogel-ath.fa
Alignments: /Volumes/Keep/+Cuscuta_evolution/NextSeq_Sep2018_sRNAseq/ccm-denovo_sRNA_annotation/02out-cca.hybrid.ShortStack/merged_alig
Location: Ccam0.32_scaffold29:1787703-1787861 minus
Name: SFs=SupFam_69,SupFam_199, SSCl=Cluster_39807, MIRNA=N5

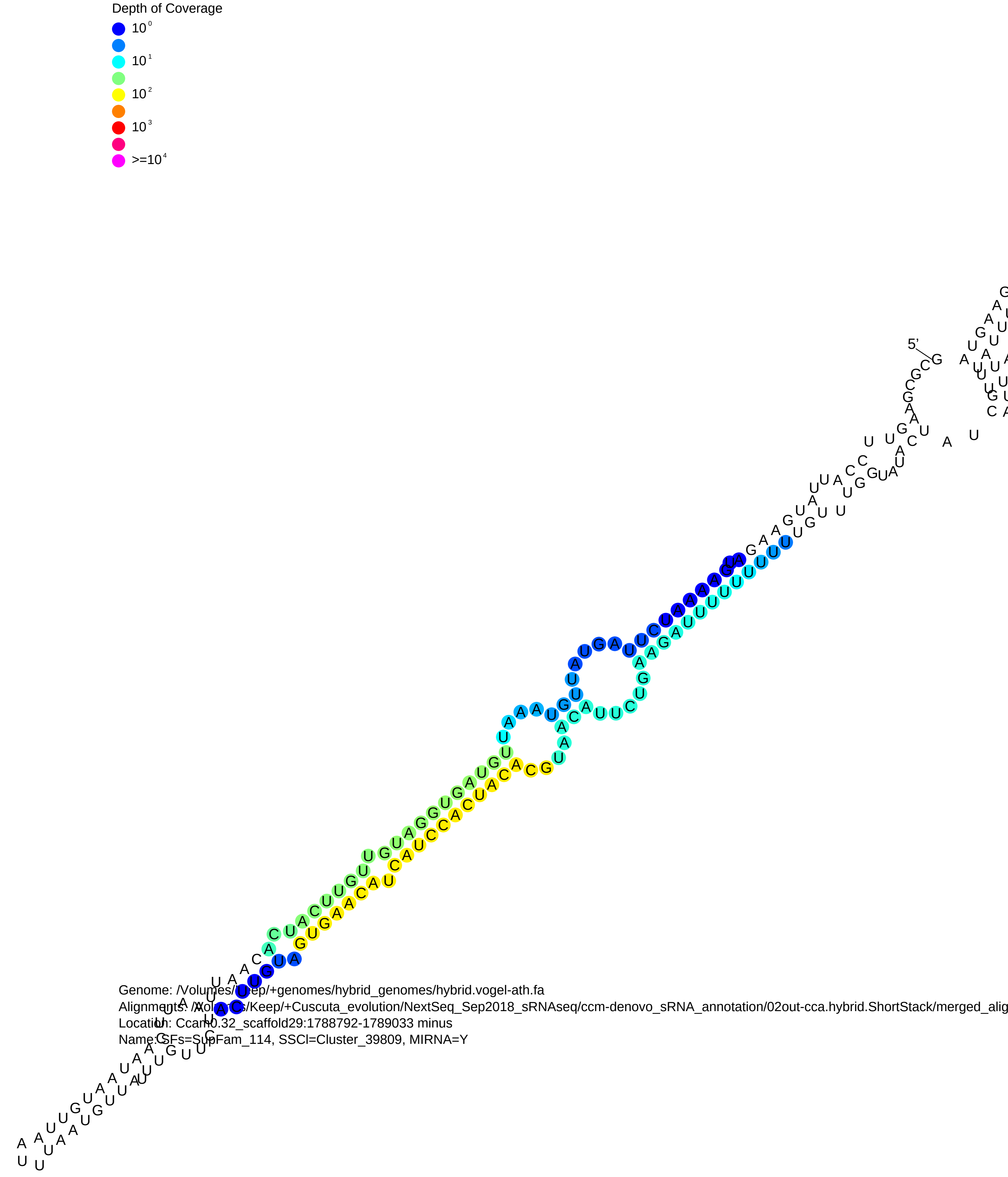

Genome: /Volumes/Keep/+genomes/hybrid_genomes/hybrid.vogel-ath.fa
Alignments: /Volumes/Keep/+Cuscuta_evolution/NextSeq_Sep2018_sRNAseq/ccm-denovo_sRNA_annotation/02out-cca.hybrid.ShortStack/merged_alig
Location: Ccam0.32_scaffold29:1788792-1789033 minus
Name: SFs=SupFam_114, SSCl=Cluster_39809, MIRNA=Y

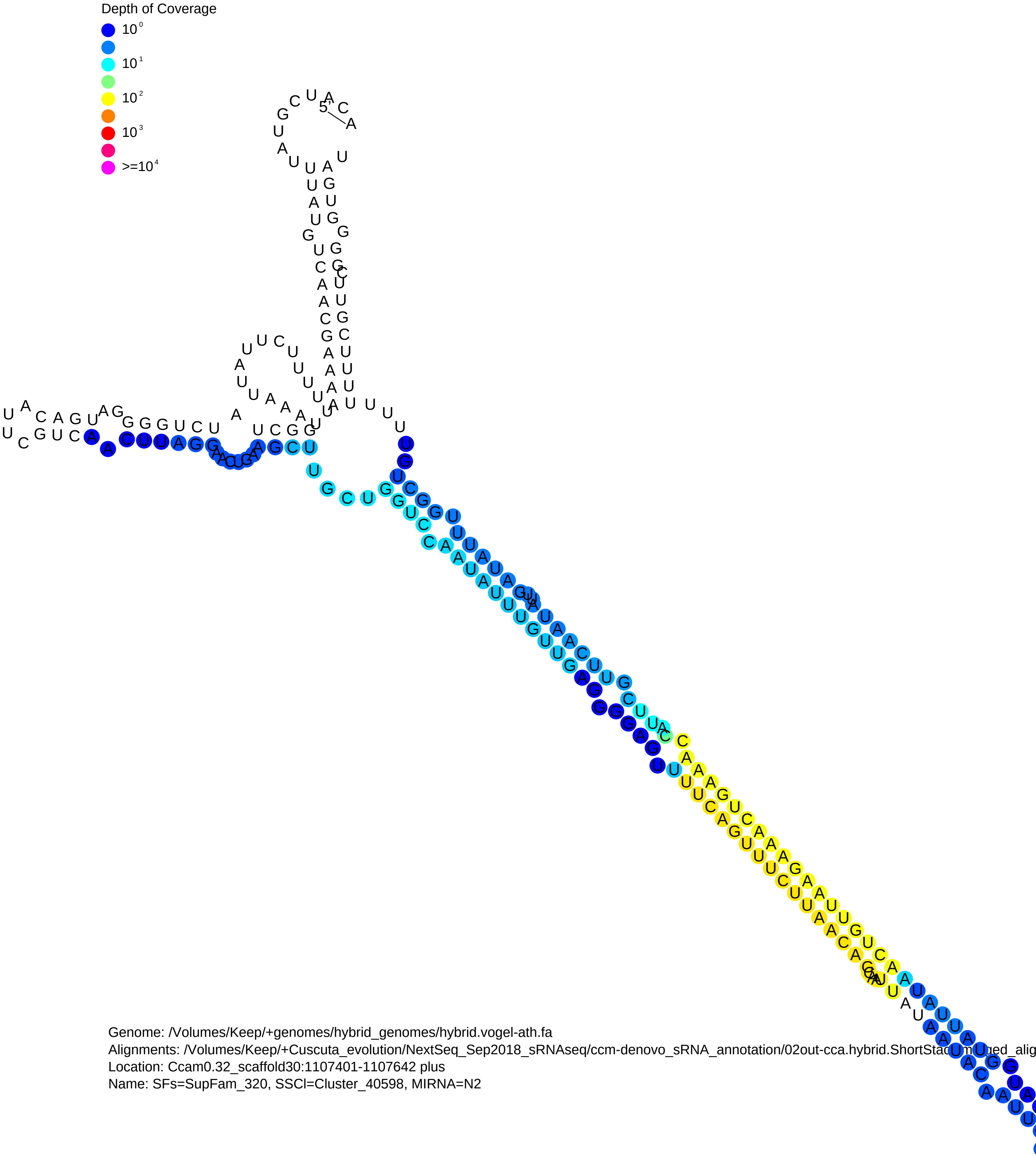

Genome: /Volumes/Keep/+genomes/hybrid_genomes/hybrid.vogel-ath.fa
Alignments: /Volumes/Keep/+Cuscuta_evolution/NextSeq_Sep2018_sRNAseq/ccm-denovo_sRNA_annotation/02out-cca.hybrid.ShortStac[illegible]ged_alig
Location: Ccam0.32_scaffold30:1107401-1107642 plus
Name: SFs=SupFam_320, SSCl=Cluster_40598, MIRNA=N2

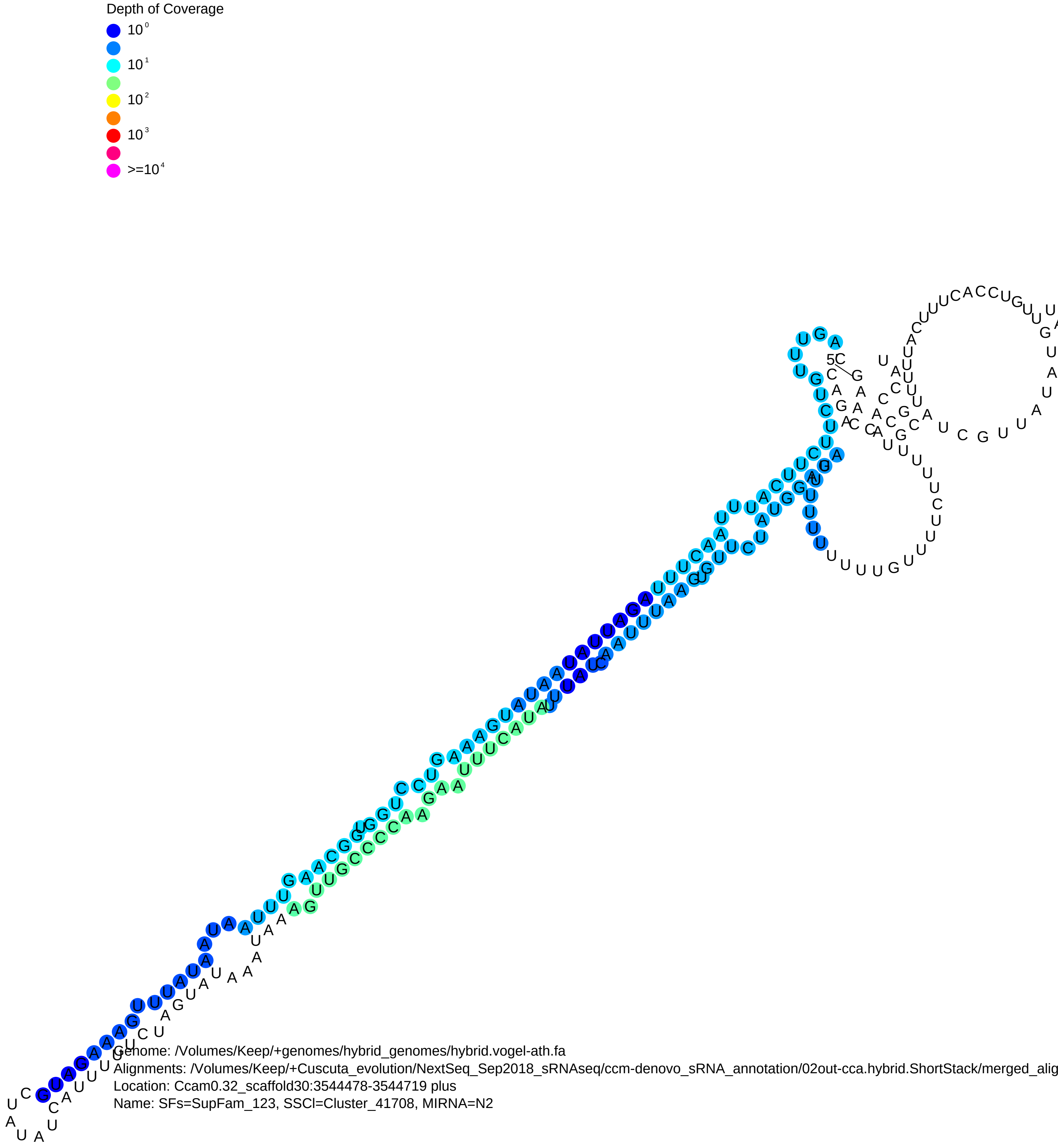

Genome: /Volumes/Keep/+genomes/hybrid_genomes/hybrid.vogel-ath.fa
Alignments: /Volumes/Keep/+Cuscuta_evolution/NextSeq_Sep2018_sRNAseq/ccm-denovo_sRNA_annotation/02out-cca.hybrid.ShortStack/merged_alig
Location: Ccam0.32_scaffold30:3544478-3544719 plus
Name: SFs=SupFam_123, SSCl=Cluster_41708, MIRNA=N2

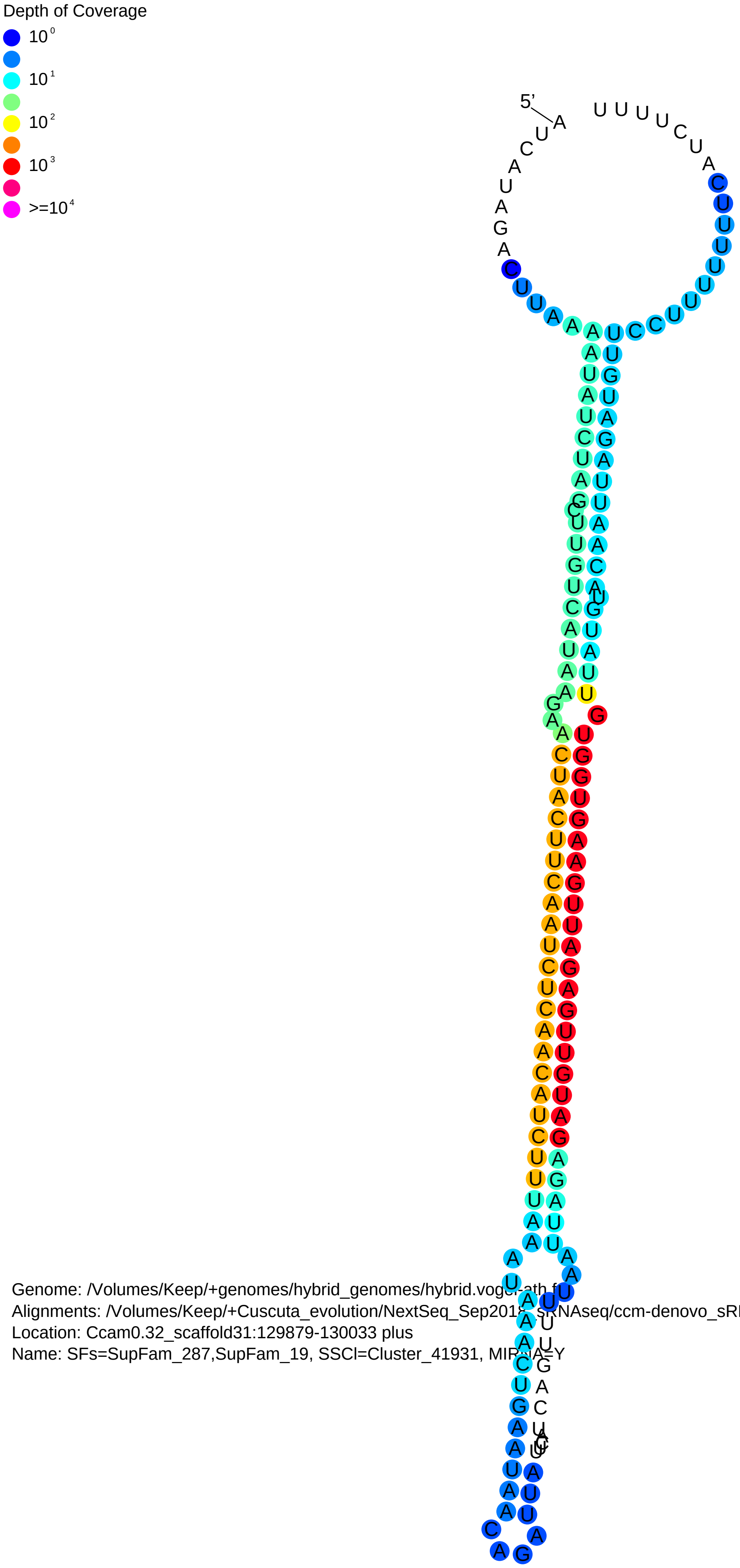

Genome: /Volumes/Keep/+genomes/hybrid_genomes/hybrid.vogel-ath.fa
Alignments: /Volumes/Keep/+Cuscuta_evolution/NextSeq_Sep2018_sRNAseq/ccm-denovo_sRNA_annotation/02out-cca.hybrid.ShortStack/merged_alig
Location: Ccam0.32_scaffold31:129879-130033 plus
Name: SFs=SupFam_287,SupFam_19, SSCl=Cluster_41931, MIRNA=Y

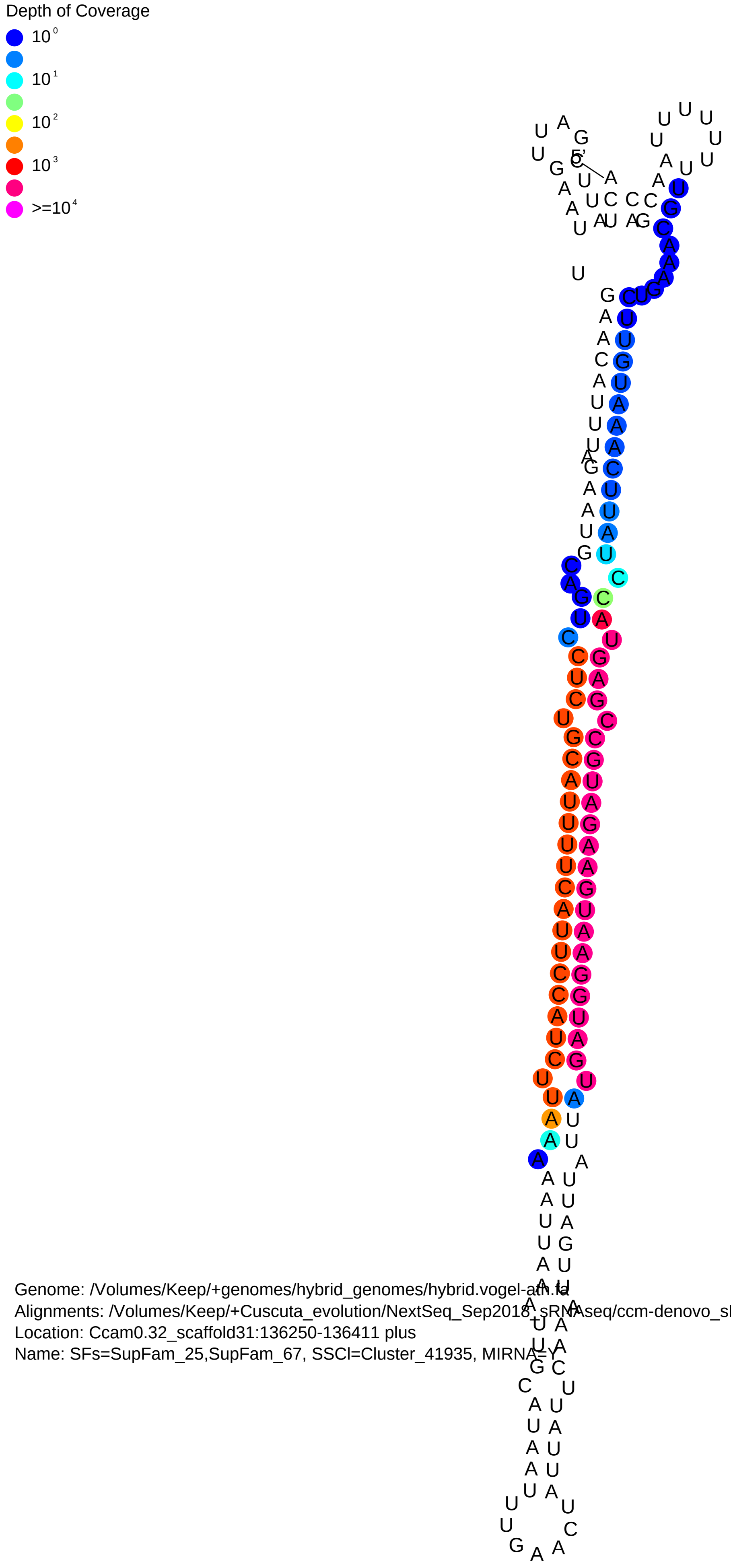

Genome: /Volumes/Keep/+genomes/hybrid_genomes/hybrid.vogel-ath.fa
Alignments: /Volumes/Keep/+Cuscuta_evolution/NextSeq_Sep2018_sRNAseq/ccm-denovo_sRNA_annotation/02out-cca.hybrid.ShortStack/merged_alig
Location: Ccam0.32_scaffold31:136250-136411 plus
Name: SFs=SupFam_25,SupFam_67, SSCl=Cluster_41935, MIRNA=Y

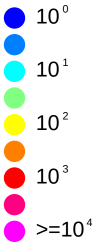

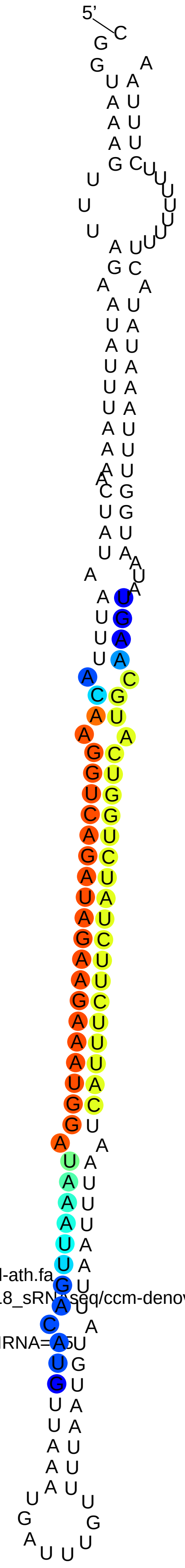

Genome: /Volumes/Keep/+genomes/hybrid_genomes/hybrid.vogel-ath.fa
Alignments: /Volumes/Keep/+Cuscuta_evolution/NextSeq_Sep2018_sRNAseq/ccm-denovo_sRNA_annotation/02out-cca.hybrid.ShortStack/merged_alig
Location: Ccam0.32_scaffold31:334915-335073 plus
Name: SFs=SupFam_294,SupFam_327, SSCl=Cluster_42008, MIRNA=[illegible]

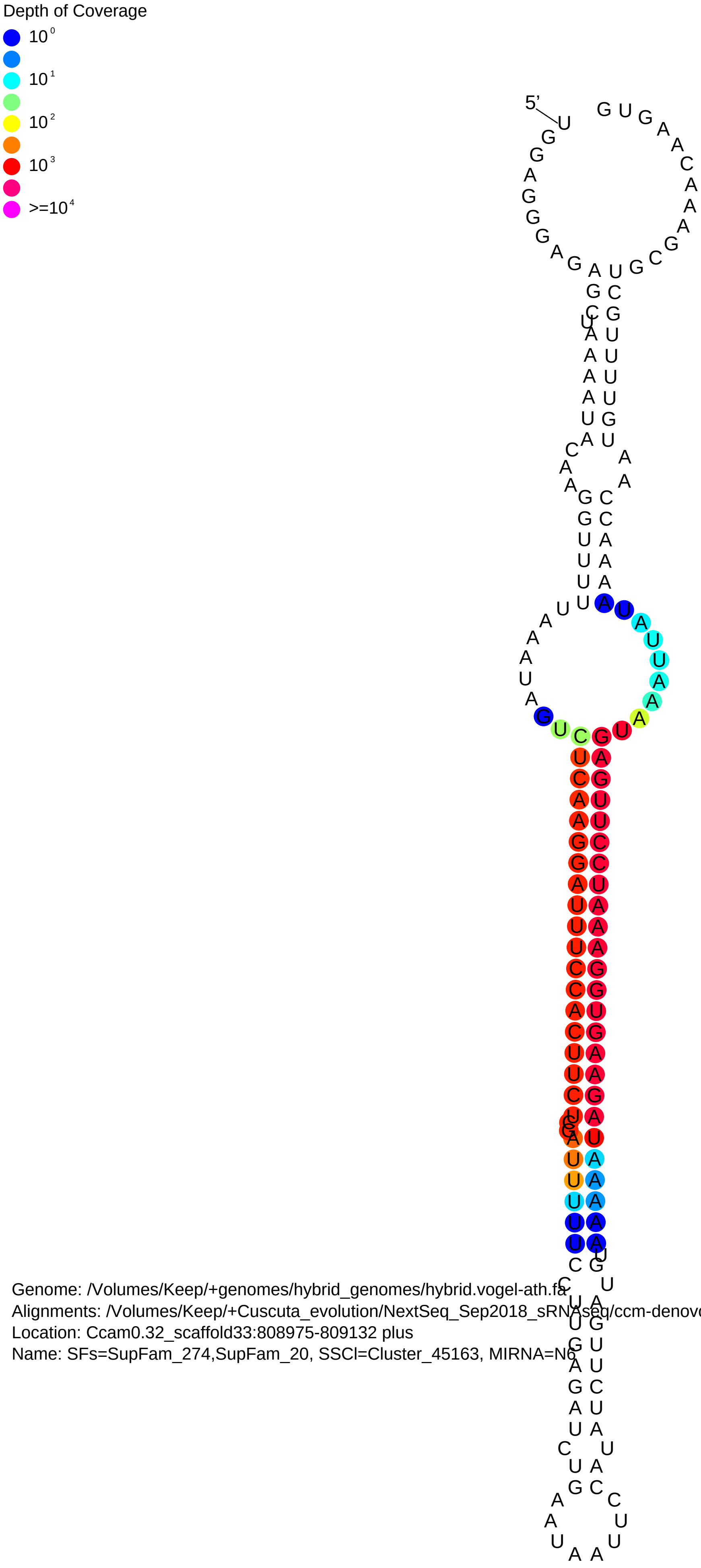

Genome: /Volumes/Keep/+genomes/hybrid_genomes/hybrid.vogel-ath.fa
Alignments: /Volumes/Keep/+Cuscuta_evolution/NextSeq_Sep2018_sRNAseq/ccm-denovo_sRNA_annotation/02out-cca.hybrid.ShortStack/merged_alig
Location: Ccam0.32_scaffold33:808975-809132 plus
Name: SFs=SupFam_274,SupFam_20, SSCl=Cluster_45163, MIRNA=N6

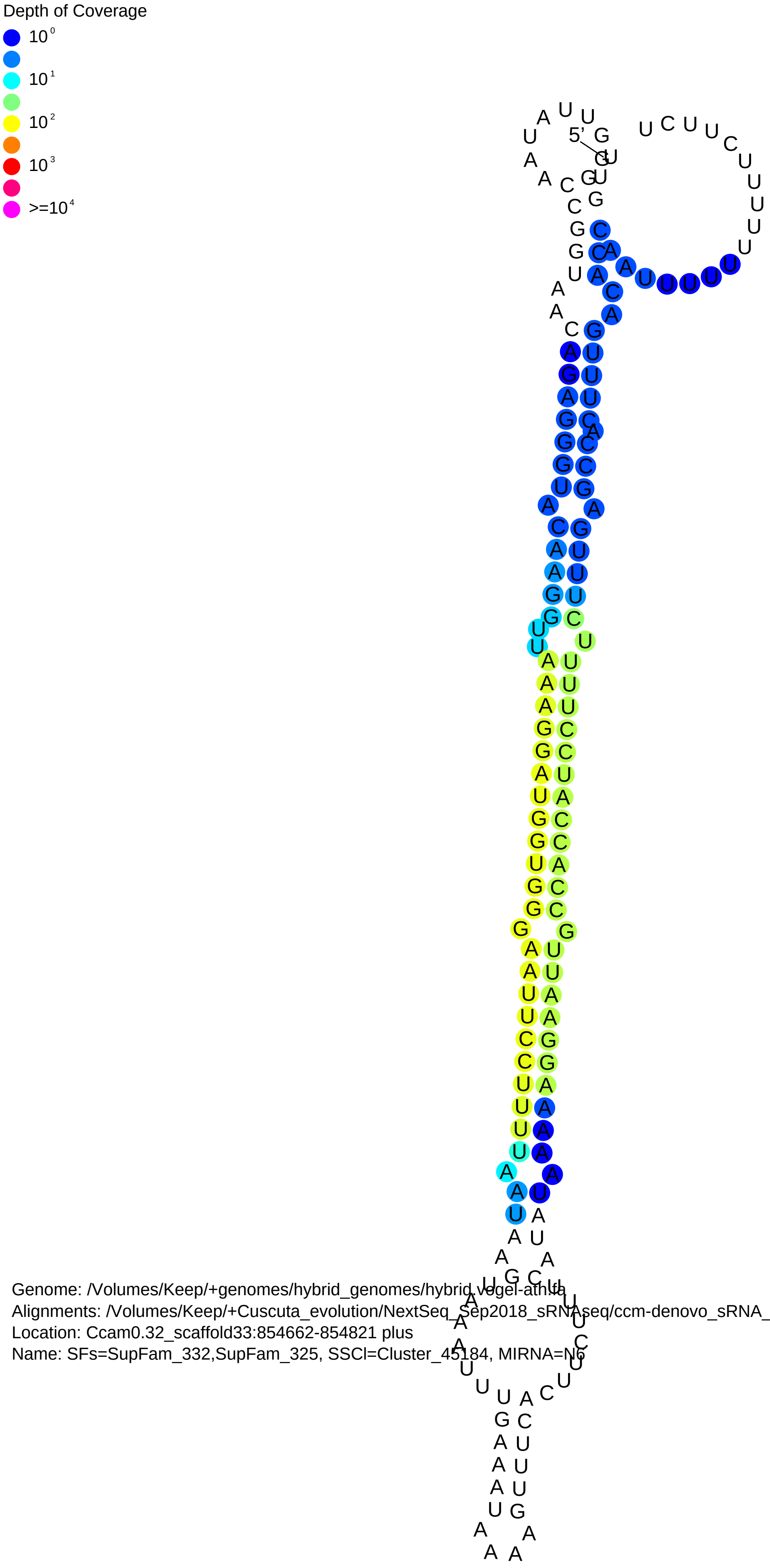

Genome: /Volumes/Keep/+genomes/hybrid_genomes/hybrid.vogel-ath.fa
Alignments: /Volumes/Keep/+Cuscuta_evolution/NextSeq_Sep2018_sRNAseq/ccm-denovo_sRNA_annotation/02out-cca.hybrid.ShortStack/merged_alig
Location: Ccam0.32_scaffold33:854662-854821 plus
Name: SFs=SupFam_332,SupFam_325, SSCl=Cluster_45184, MIRNA=N6

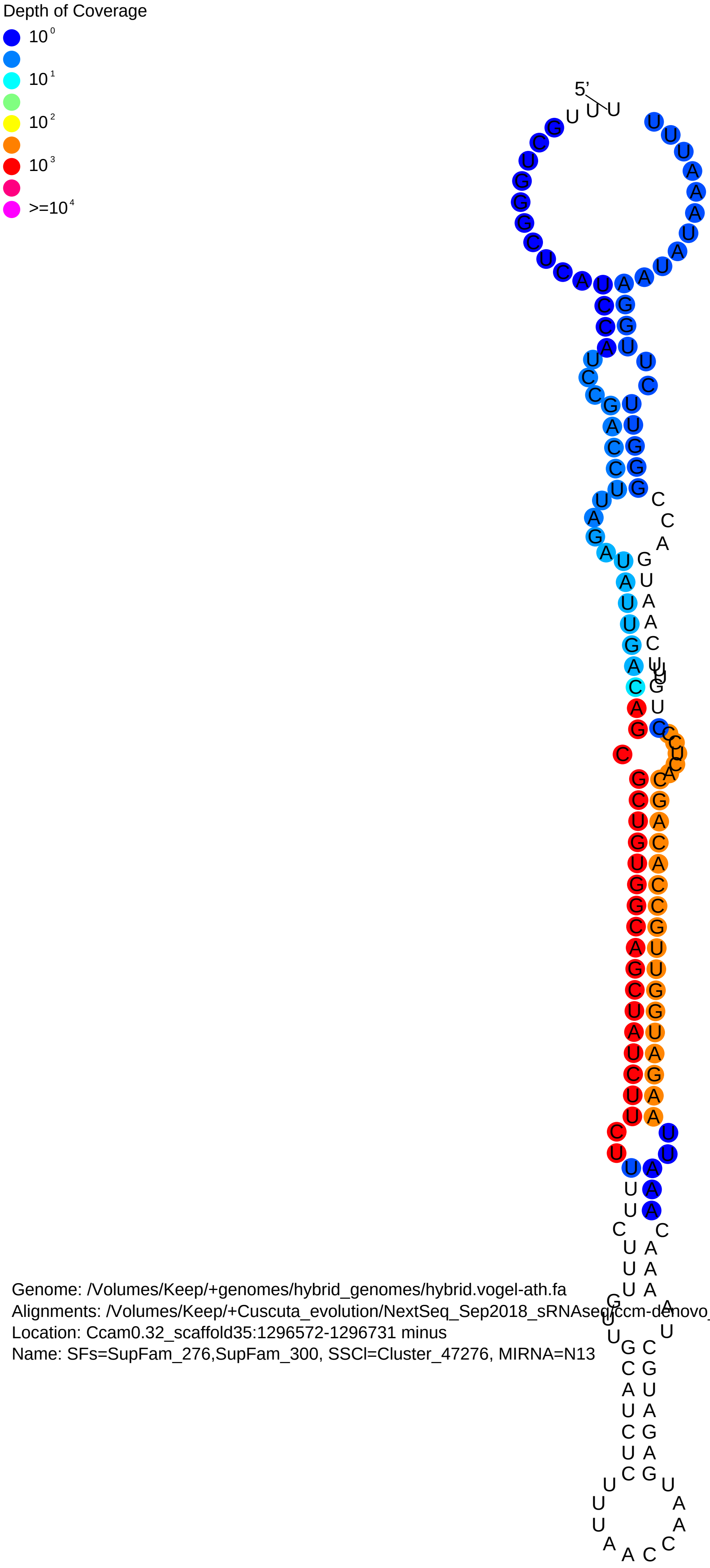

Genome: /Volumes/Keep/+genomes/hybrid_genomes/hybrid.vogel-ath.fa
Alignments: /Volumes/Keep/+Cuscuta_evolution/NextSeq_Sep2018_sRNAseq/ccm-denovo_sRNA_annotation/02out-cca.hybrid.ShortStack/merged_alig
Location: Ccam0.32_scaffold35:1296572-1296731 minus
Name: SFs=SupFam_276,SupFam_300, SSCl=Cluster_47276, MIRNA=N13

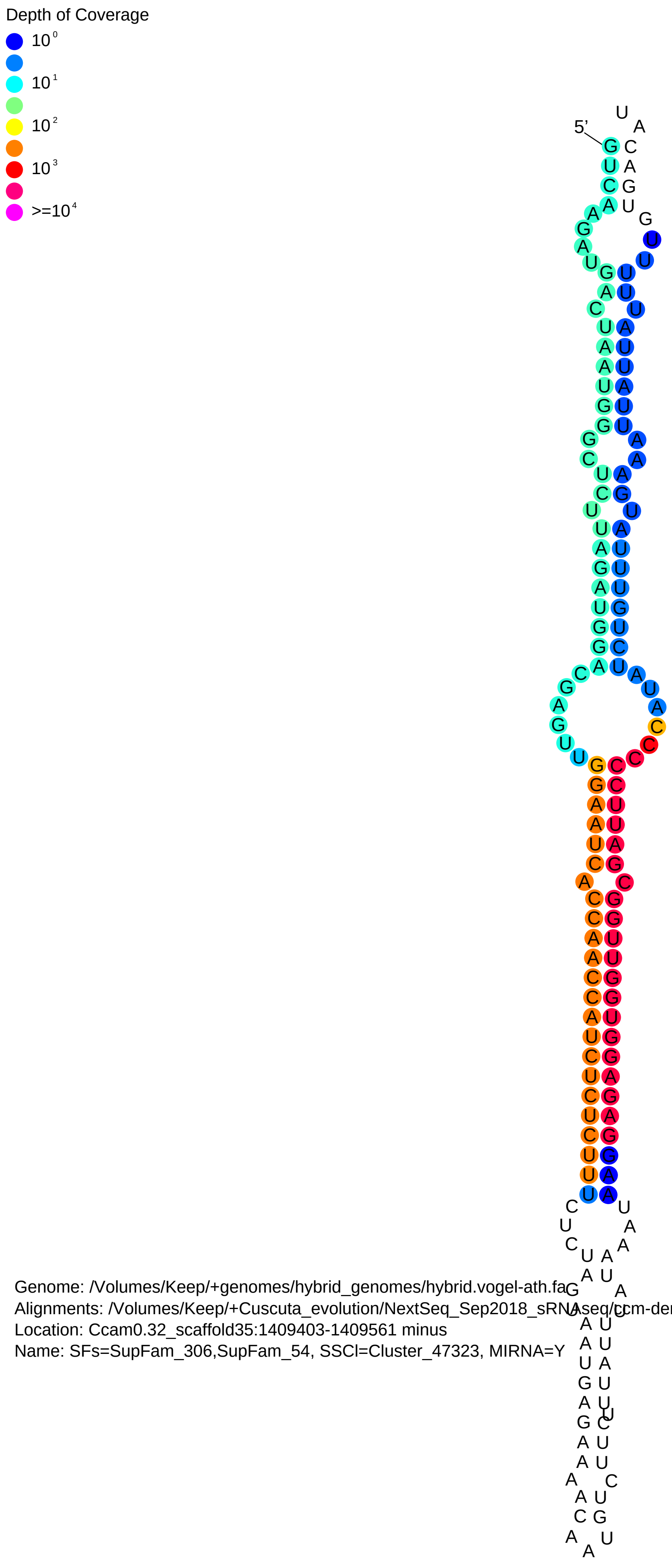

Genome: /Volumes/Keep/+genomes/hybrid_genomes/hybrid.vogel-ath.fa
Alignments: /Volumes/Keep/+Cuscuta_evolution/NextSeq_Sep2018_sRNAseq/ccm-denovo_sRNA_annotation/02out-cca.hybrid.ShortStack/merged_alig
Location: Ccam0.32_scaffold35:1409403-1409561 minus
Name: SFs=SupFam_306,SupFam_54, SSCl=Cluster_47323, MIRNA=Y

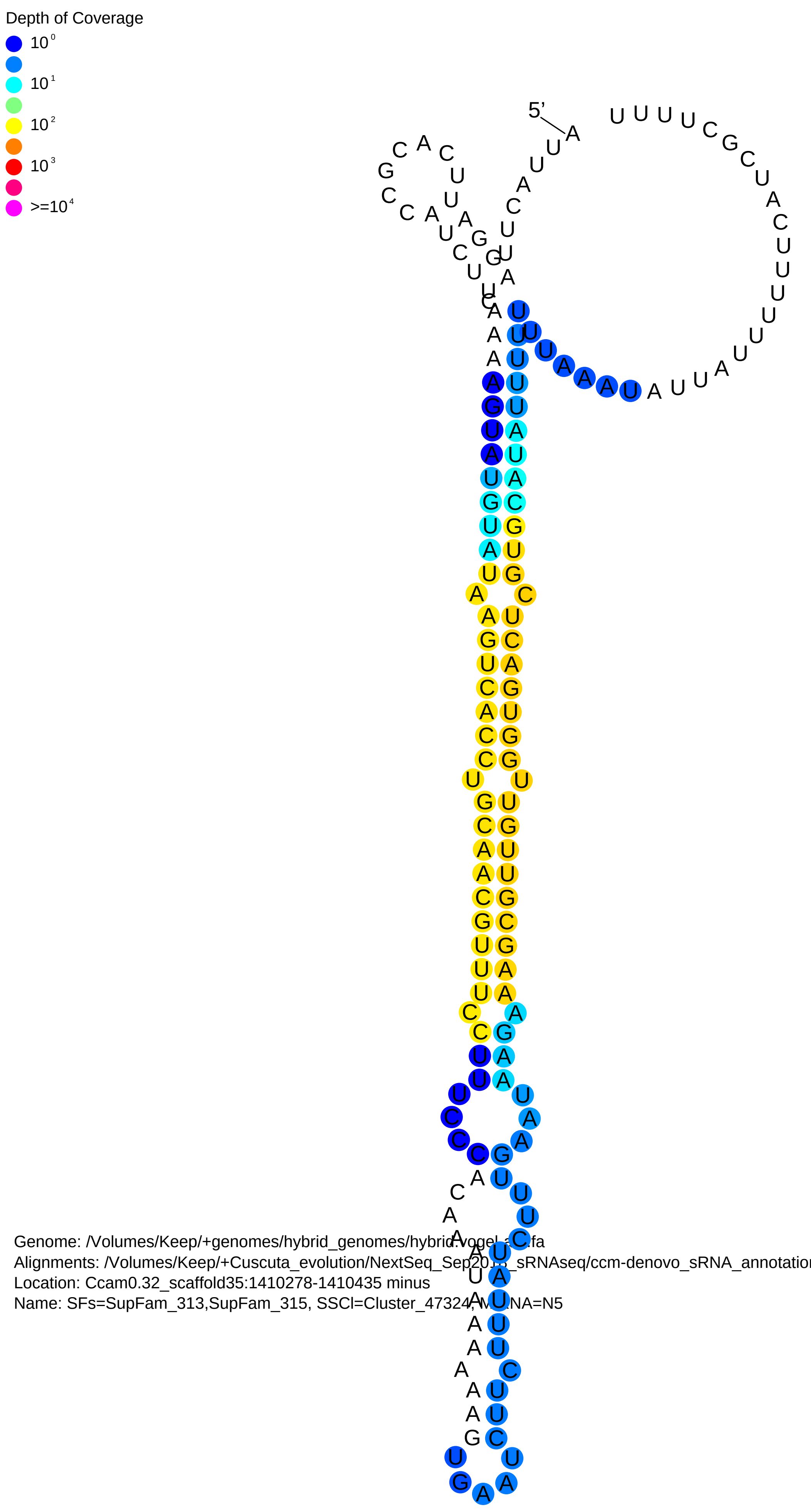

Genome: /Volumes/Keep/+genomes/hybrid_genomes/hybrid.vogel_a[illegible].fa
Alignments: /Volumes/Keep/+Cuscuta_evolution/NextSeq_Sep20[illegible]_sRNAseq/ccm-denovo_sRNA_annotation/02out-cca.hybrid.ShortStack/merged_alig
Location: Ccam0.32_scaffold35:1410278-1410435 minus
Name: SFs=SupFam_313,SupFam_315, SSCl=Cluster_47324, M[illegible]NA=N5

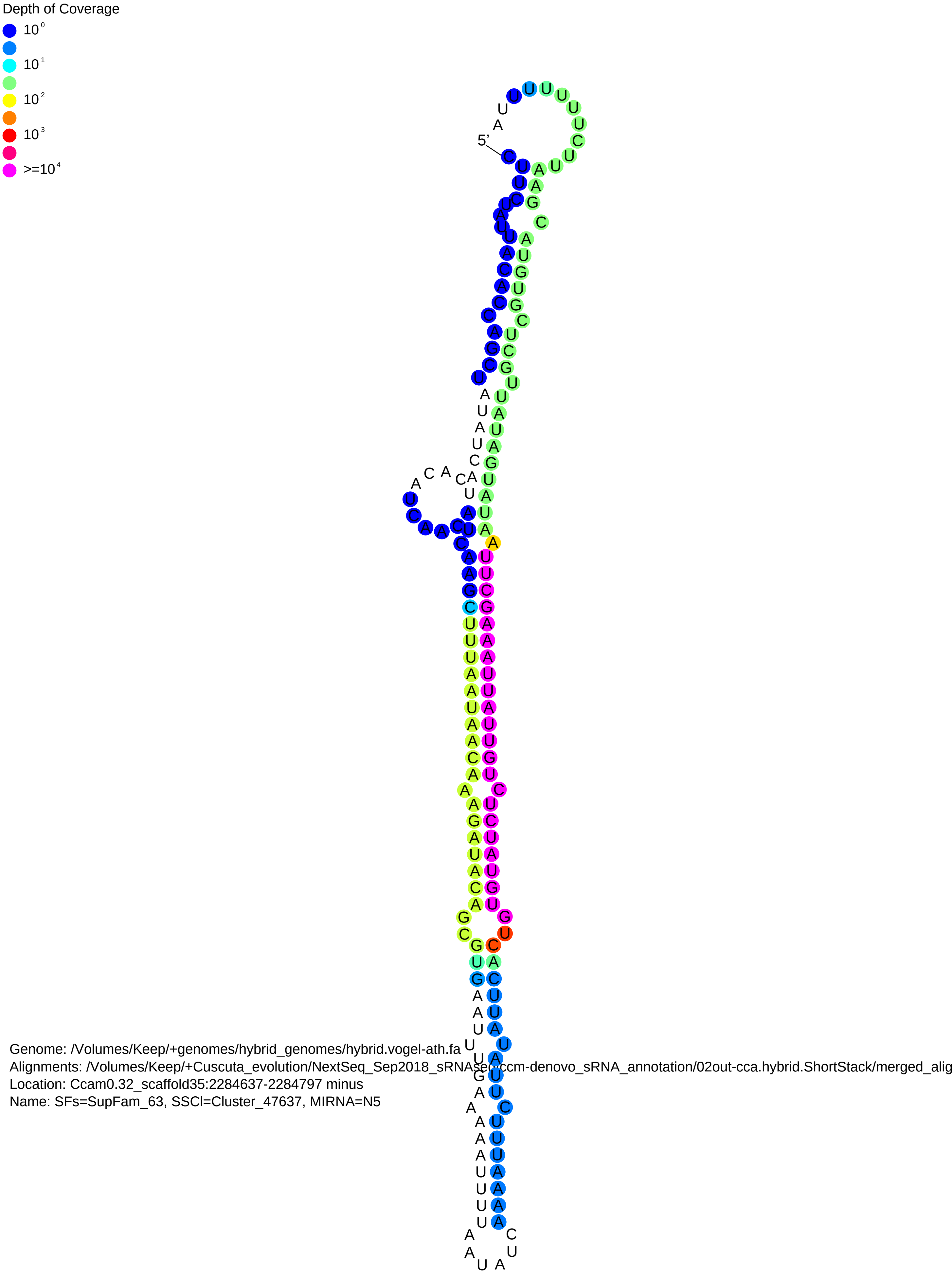
Depth of Coverage
10^0
10^1
10^2
10^3
>=10^4
5'
Genome: /Volumes/Keep/+genomes/hybrid_genomes/hybrid.vogel-ath.fa
Alignments: /Volumes/Keep/+Cuscuta_evolution/NextSeq_Sep2018_sRNAseq/ccm-denovo_sRNA_annotation/02out-cca.hybrid.ShortStack/merged_alig
Location: Ccam0.32_scaffold35:2284637-2284797 minus
Name: SFs=SupFam_63, SSCl=Cluster_47637, MIRNA=N5

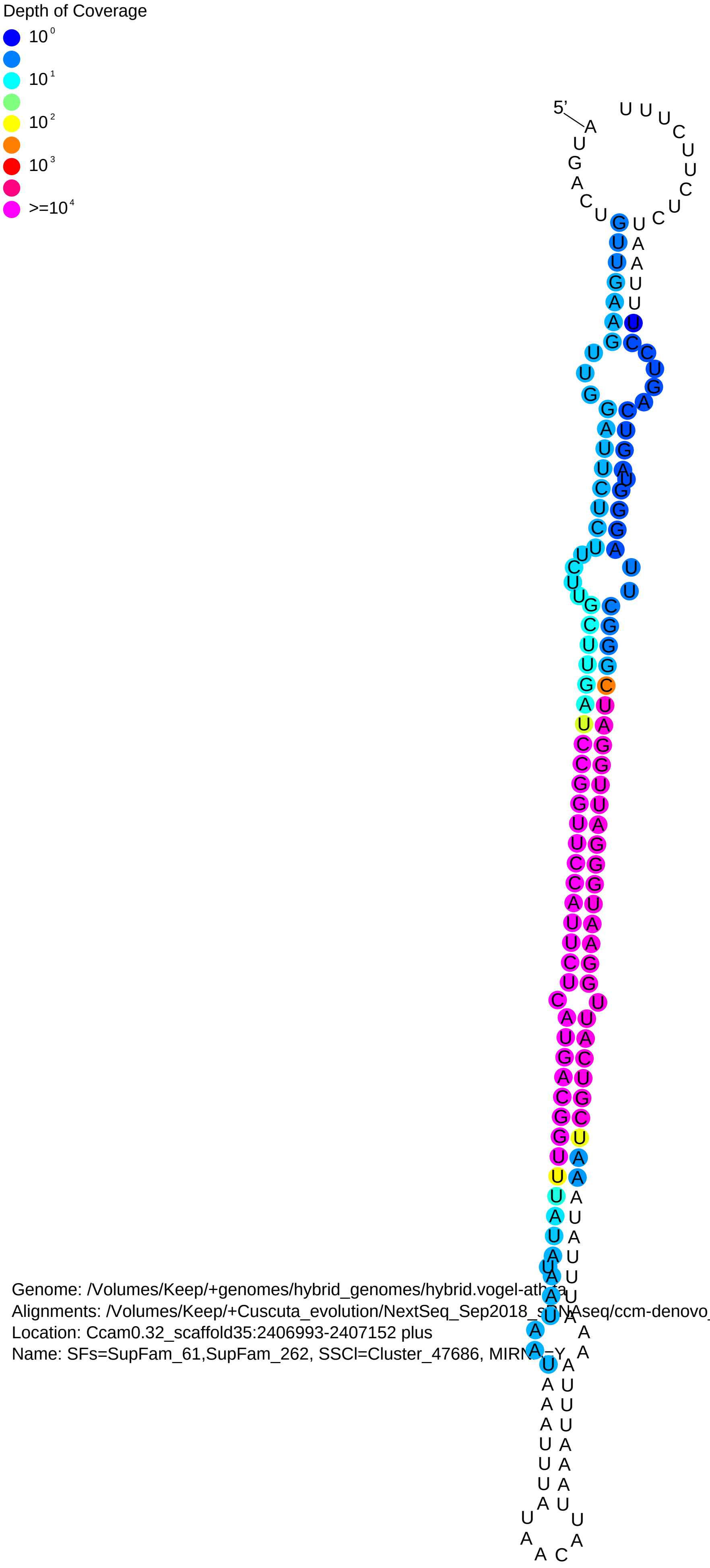

Genome: /Volumes/Keep/+genomes/hybrid_genomes/hybrid.vogel-ath[illegible]a
Alignments: /Volumes/Keep/+Cuscuta_evolution/NextSeq_Sep2018_s[illegible]NAseq/ccm-denovo_sRNA_annotation/02out-cca.hybrid.ShortStack/merged_alig
Location: Ccam0.32_scaffold35:2406993-2407152 plus
Name: SFs=SupFam_61,SupFam_262, SSCl=Cluster_47686, MIRN[illegible]=Y

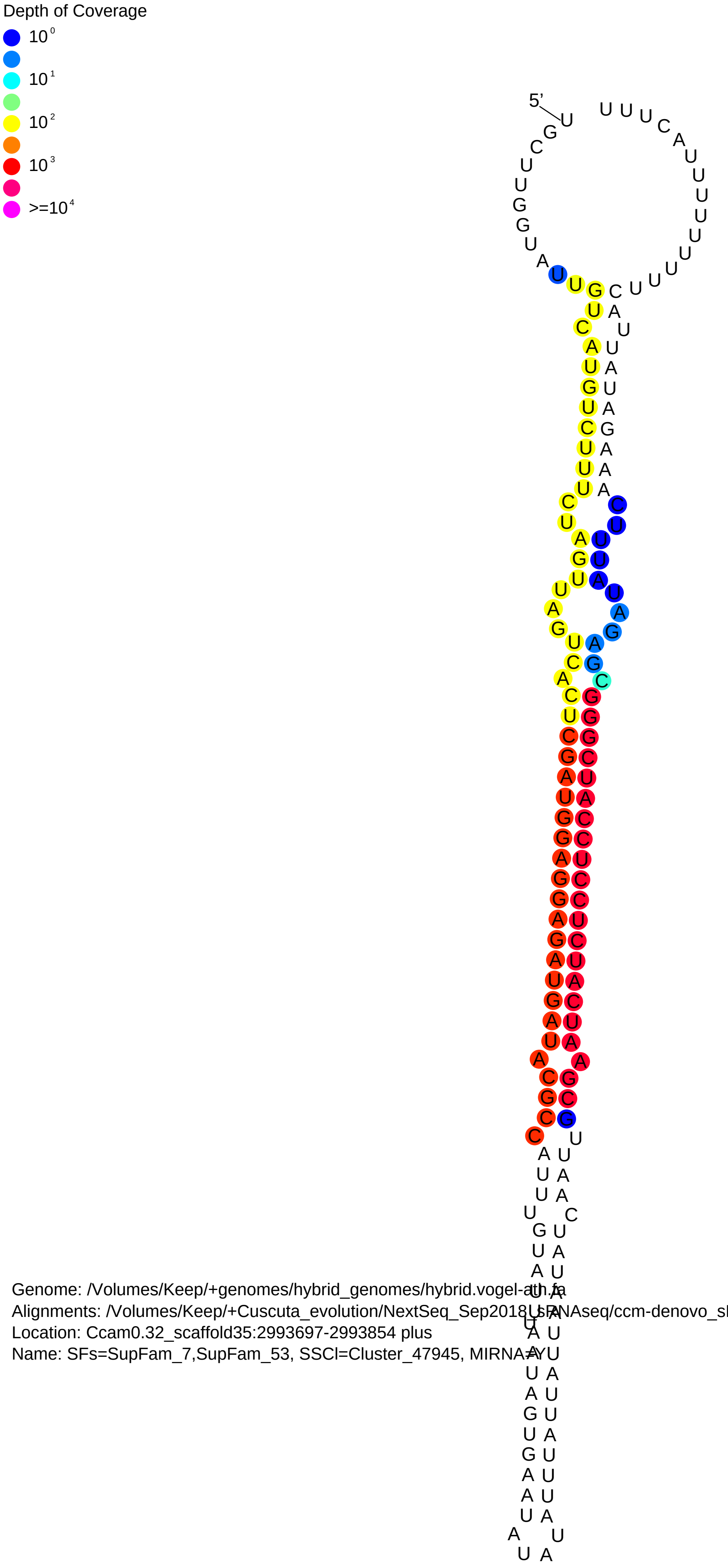

Genome: /Volumes/Keep/+genomes/hybrid_genomes/hybrid.vogel-ath.fa
Alignments: /Volumes/Keep/+Cuscuta_evolution/NextSeq_Sep2018_sRNAseq/ccm-denovo_sRNA_annotation/02out-cca.hybrid.ShortStack/merged_alig
Location: Ccam0.32_scaffold35:2993697-2993854 plus
Name: SFs=SupFam_7,SupFam_53, SSCl=Cluster_47945, MIRNA=Y

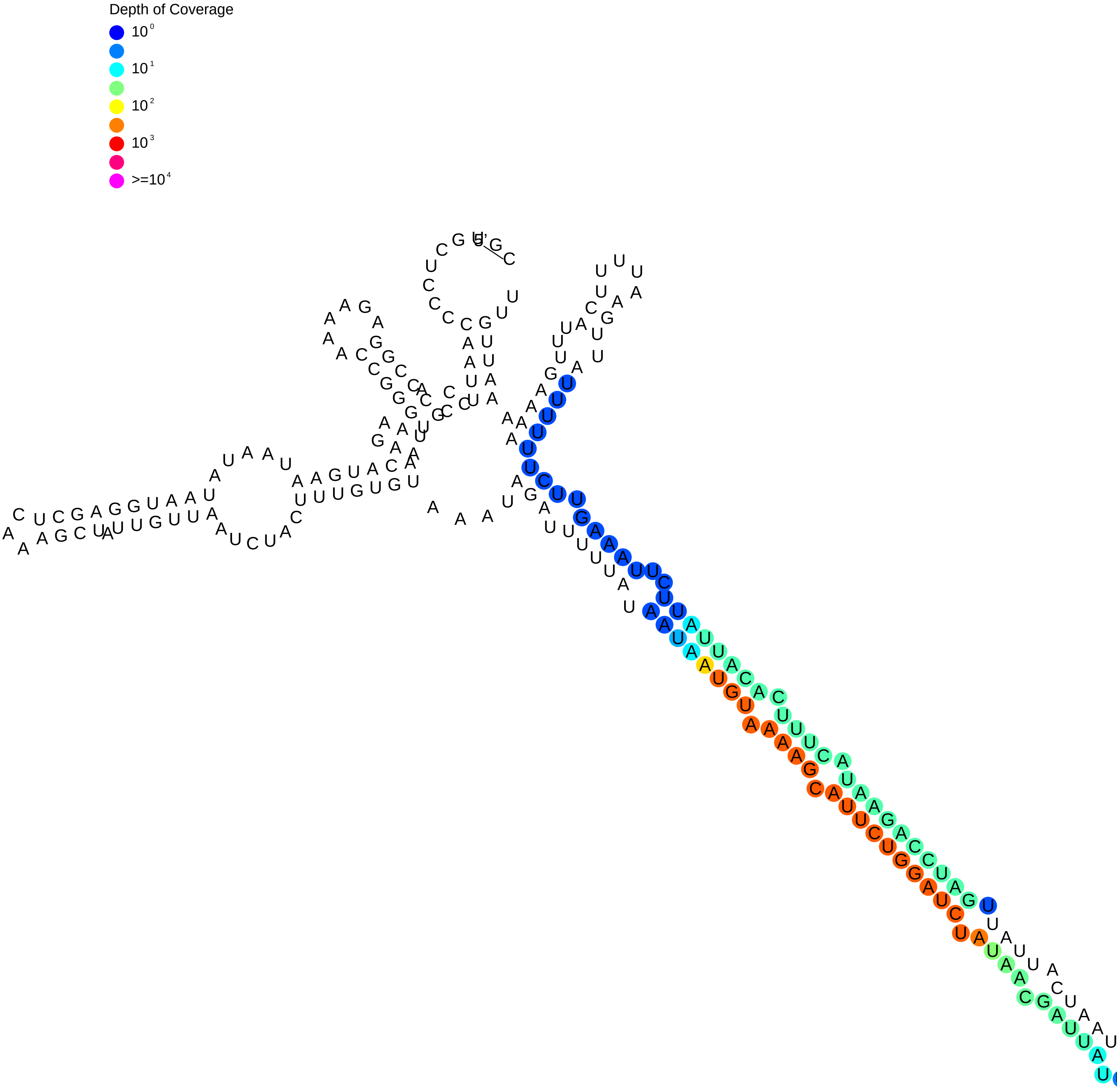

Genome: /Volumes/Keep/+genomes/hybrid_genomes/hybrid.vogel-ath.fa
Alignments: /Volumes/Keep/+Cuscuta_evolution/NextSeq_Sep2018_sRNAseq/ccm-denovo_sRNA_annotation/02out-cca.hybrid.ShortStack/merged_alig
Location: Ccam0.32_scaffold40:502244-502485 plus
Name: SFs=SupFam_196, SSCl=Cluster_52374, MIRNA=Y

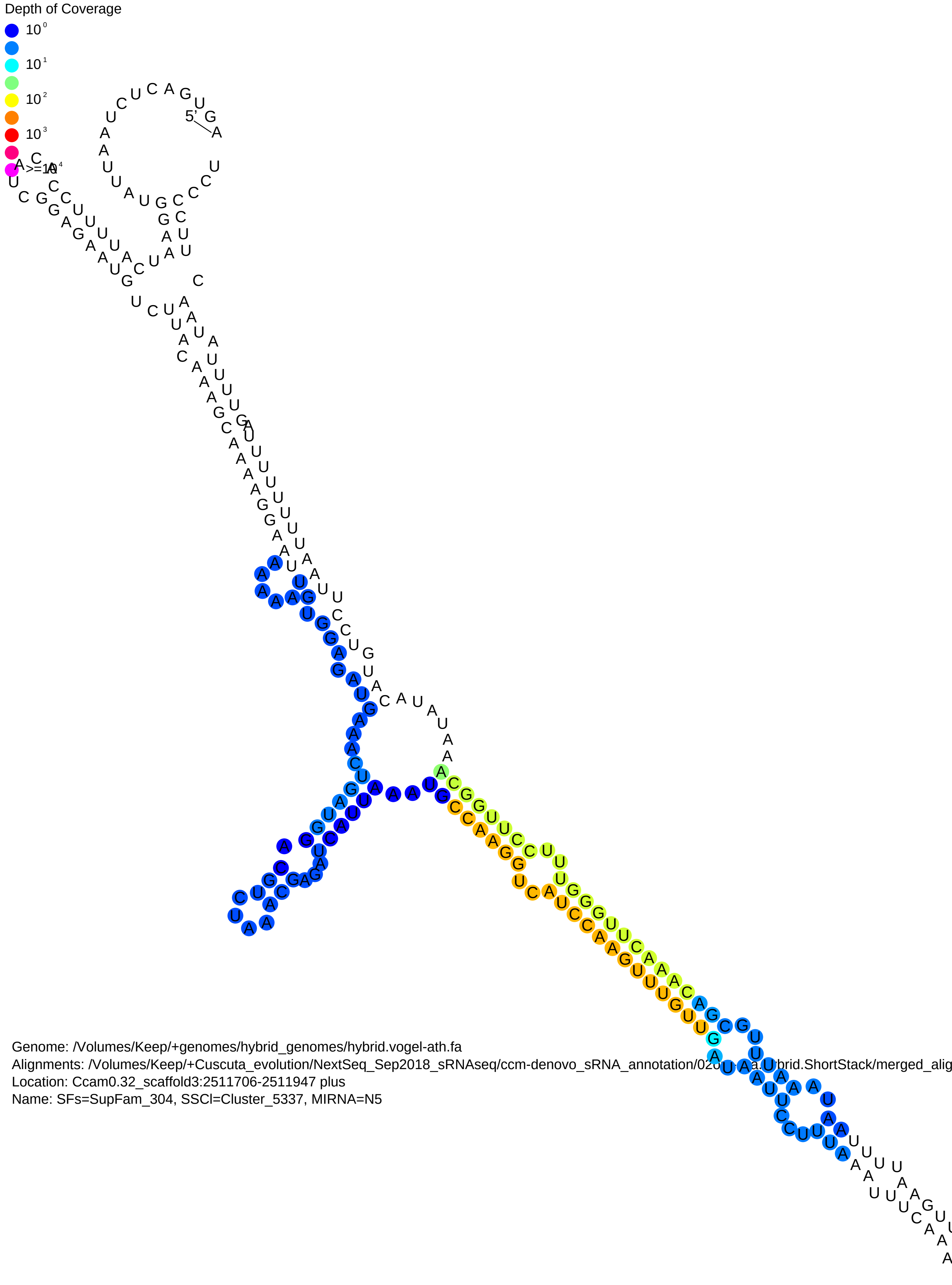

Genome: /Volumes/Keep/+genomes/hybrid_genomes/hybrid.vogel-ath.fa
Alignments: /Volumes/Keep/+Cuscuta_evolution/NextSeq_Sep2018_sRNAseq/ccm-denovo_sRNA_annotation/02[illegible]a.hybrid.ShortStack/merged_alig
Location: Ccam0.32_scaffold3:2511706-2511947 plus
Name: SFs=SupFam_304, SSCl=Cluster_5337, MIRNA=N5

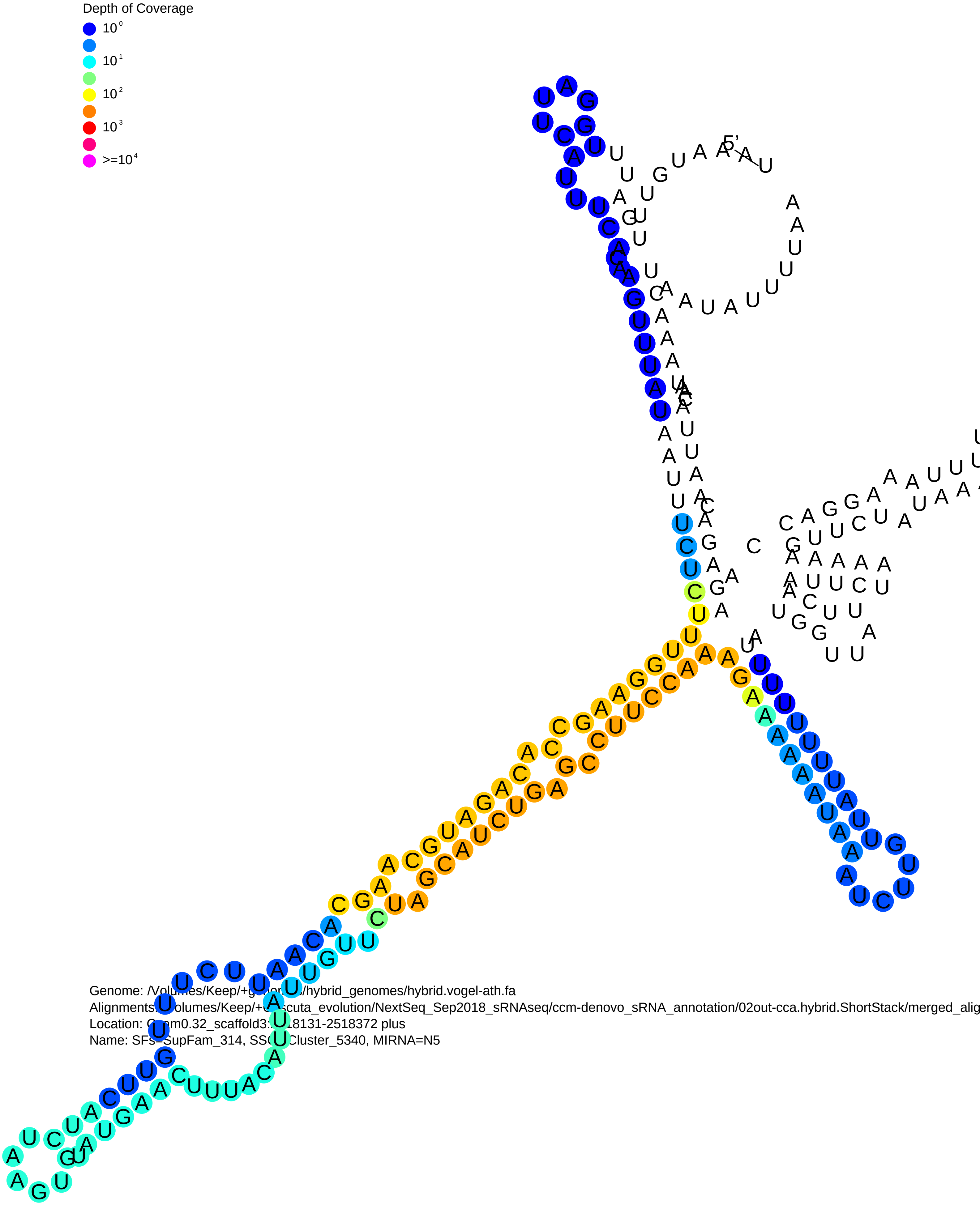

Genome: /Vo[illegible]mes/Keep/+g[illegible]s/hybrid_genomes/hybrid.vogel-ath.fa
Alignments:[illegible]olumes/Keep/+[illegible]scuta_evolution/NextSeq_Sep2018_sRNAseq/ccm-denovo_sRNA_annotation/02out-cca.hybrid.ShortStack/merged_alig[illegible]
Location: C[illegible]m0.32_scaffold3:[illegible]18131-2518372 plus
Name: SFs=SupFam_314, SS[illegible]Cluster_5340, MIRNA=N5

Depth of Coverage

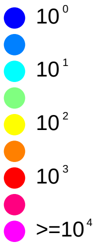

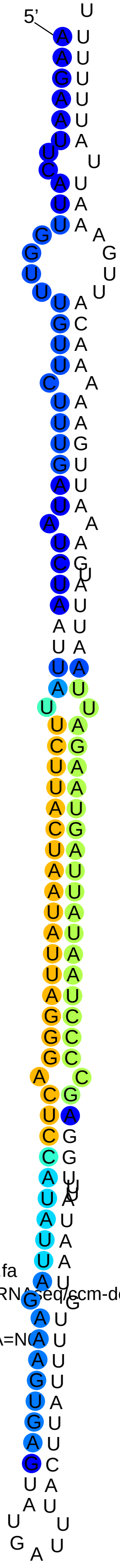

Genome: /Volumes/Keep/+genomes/hybrid_genomes/hybrid.vogel-ath.fa
Alignments: /Volumes/Keep/+Cuscuta_evolution/NextSeq_Sep2018_sRNAseq/ccm-denovo_sRNA_annotation/02out-cca.hybrid.ShortStack/merged_alig
Location: Ccam0.32_scaffold41:1115117-1115274 minus
Name: SFs=SupFam_129,SupFam_140, SSCl=Cluster_53524, MIRNA=N

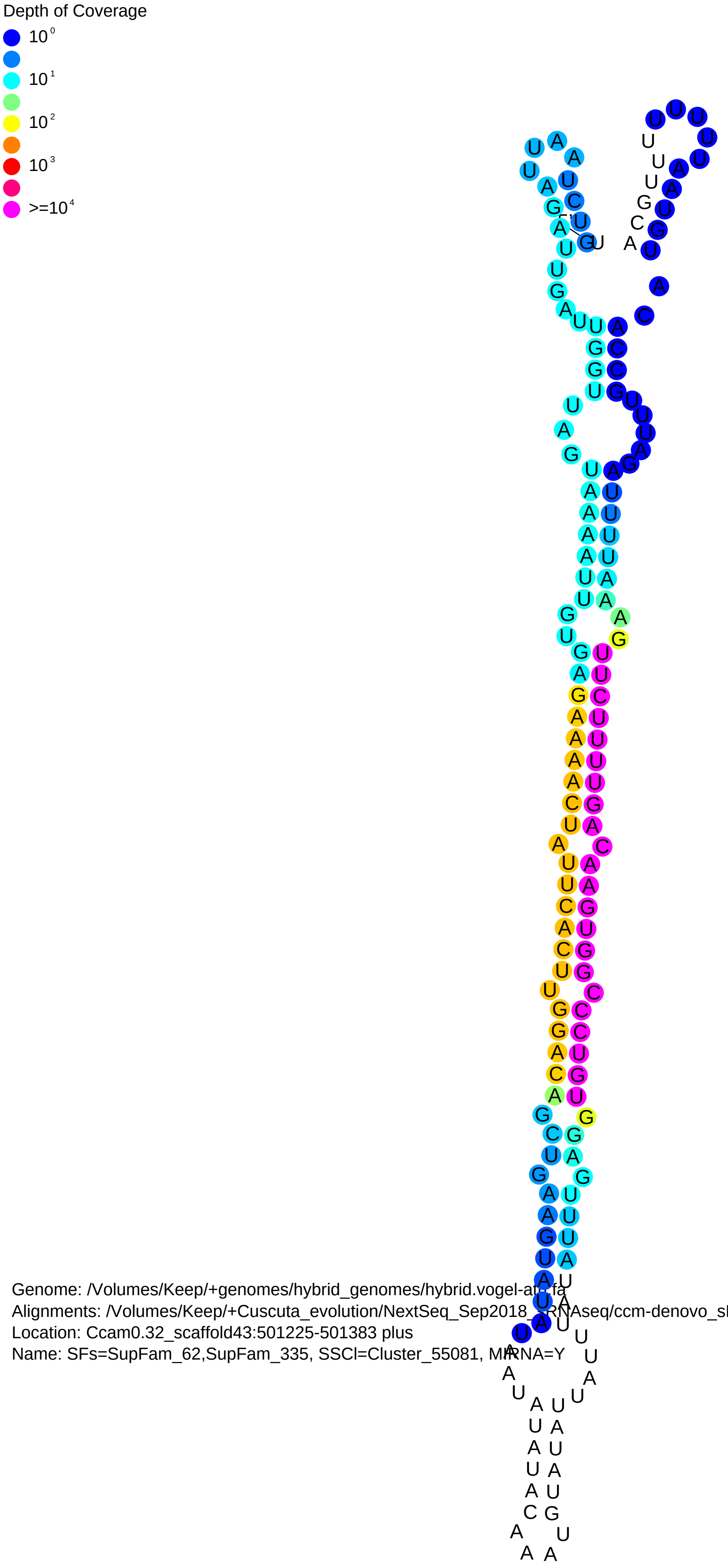

Genome: /Volumes/Keep/+genomes/hybrid_genomes/hybrid.vogel-at[illegible]fa
Alignments: /Volumes/Keep/+Cuscuta_evolution/NextSeq_Sep2018_[illegible]RNAseq/ccm-denovo_sRNA_annotation/02out-cca.hybrid.ShortStack/merged_alig[illegible]
Location: Ccam0.32_scaffold43:501225-501383 plus
Name: SFs=SupFam_62,SupFam_335, SSCl=Cluster_55081, M[illegible]RNA=Y

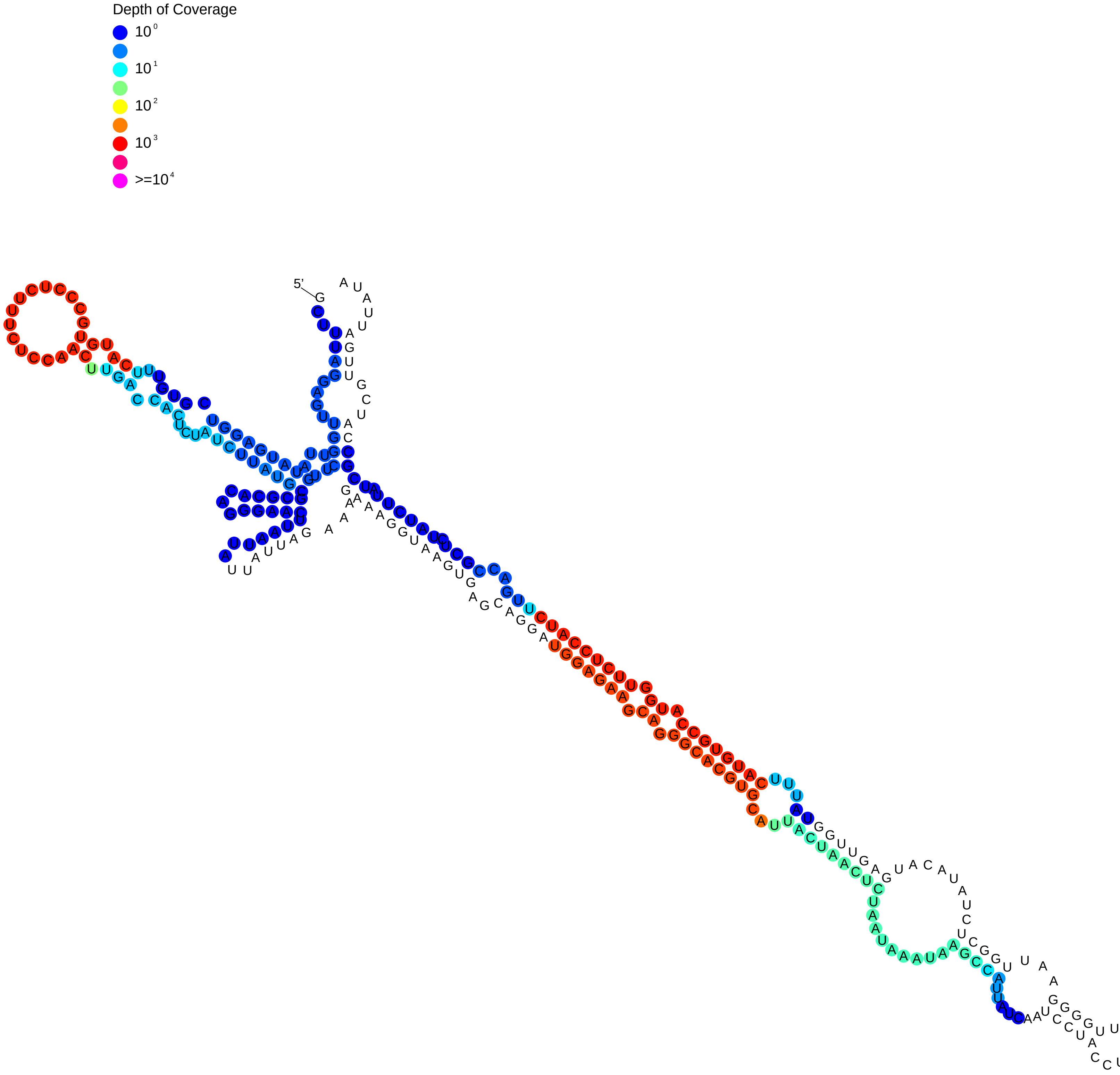

Genome: /Volumes/Keep/+genomes/hybrid_genomes/hybrid.vogel-ath.fa
Alignments: /Volumes/Keep/+Cuscuta_evolution/NextSeq_Sep2018_sRNAseq/ccm-denovo_sRNA_annotation/02out-cca.hybrid.ShortStack/merged_alig
Location: Ccam0.32_scaffold44:569410-569710 minus
Name: SFs=SupFam_2, SSCl=Cluster_56048, MIRNA=N5

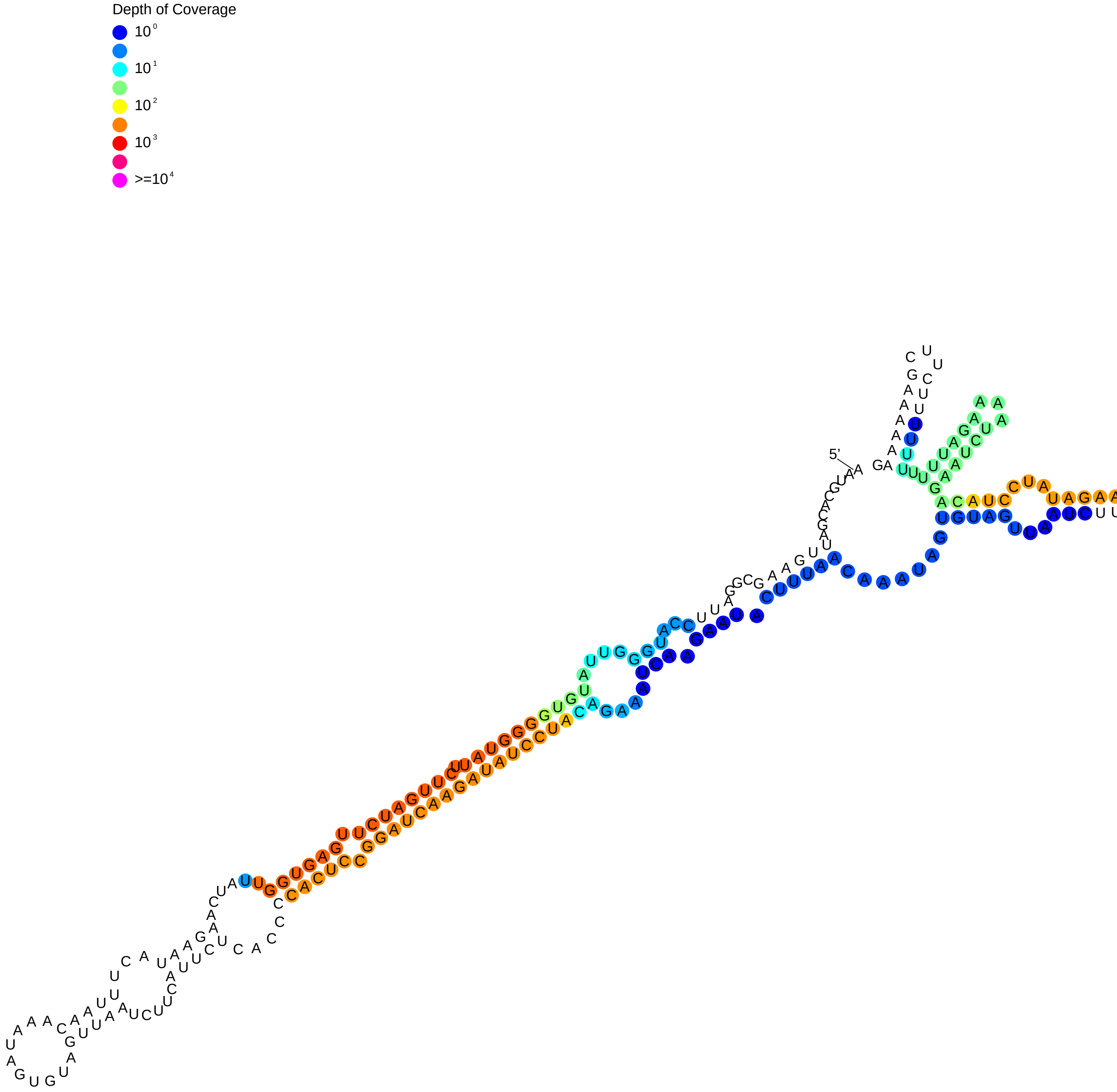

Genome: /Volumes/Keep/+genomes/hybrid_genomes/hybrid.vogel-ath.fa
Alignments: /Volumes/Keep/+Cuscuta_evolution/NextSeq_Sep2018_sRNAseq/ccm-denovo_sRNA_annotation/02out-cca.hybrid.ShortStack/merged_alig
Location: Ccam0.32_scaffold45:405815-406052 plus
Name: SFs=SupFam_771,SupFam_295, SSCl=Cluster_57731, MIRNA=N5

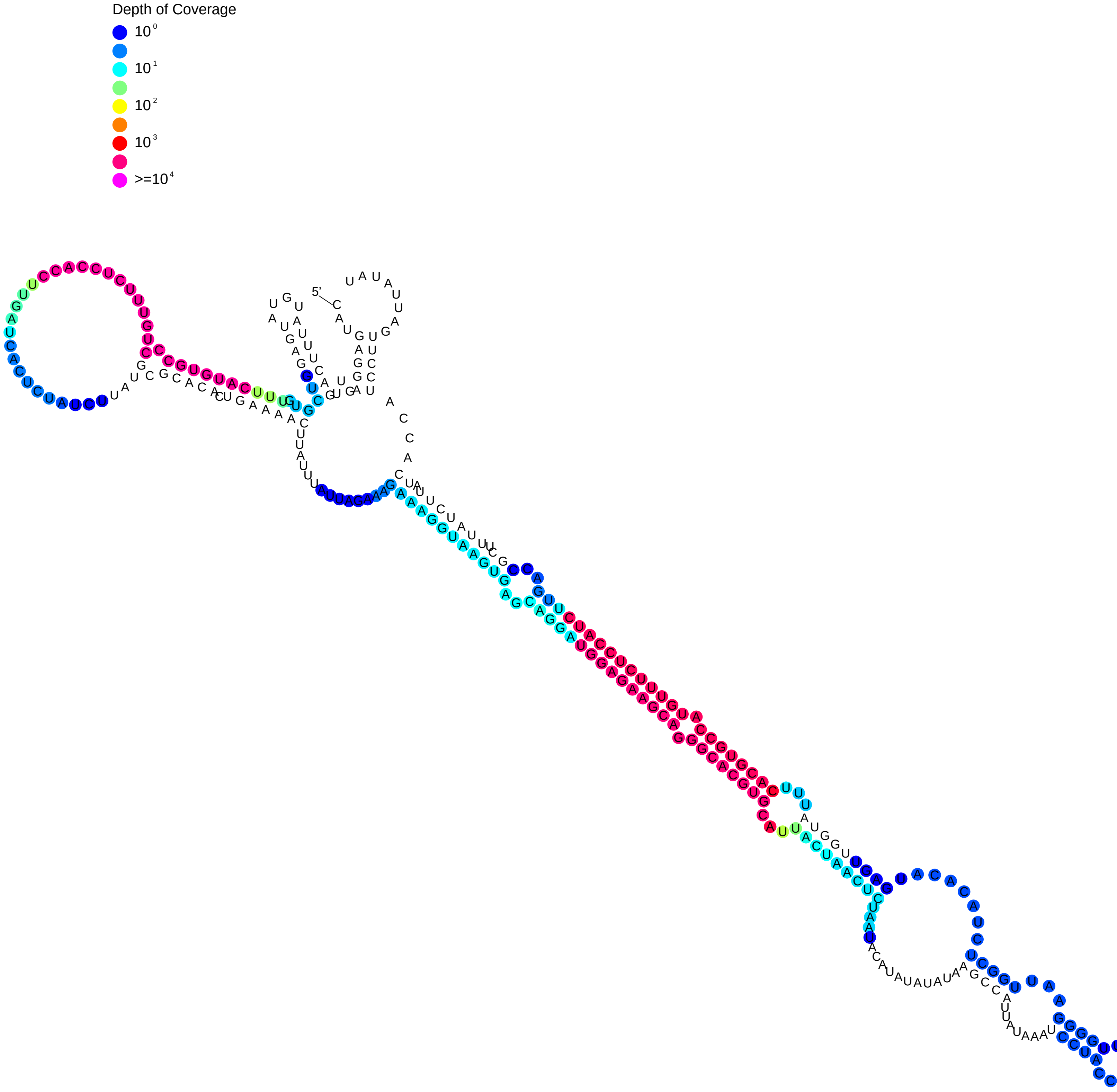

Genome: /Volumes/Keep/+genomes/hybrid_genomes/hybrid.vogel-ath.fa
Alignments: /Volumes/Keep/+Cuscuta_evolution/NextSeq_Sep2018_sRNAseq/ccm-denovo_sRNA_annotation/02out-cca.hybrid.ShortStack/merged_alig
Location: Ccam0.32_scaffold48:1350608-1350911 plus
Name: SFs=SupFam_2, SSCl=Cluster_60345, MIRNA=N5

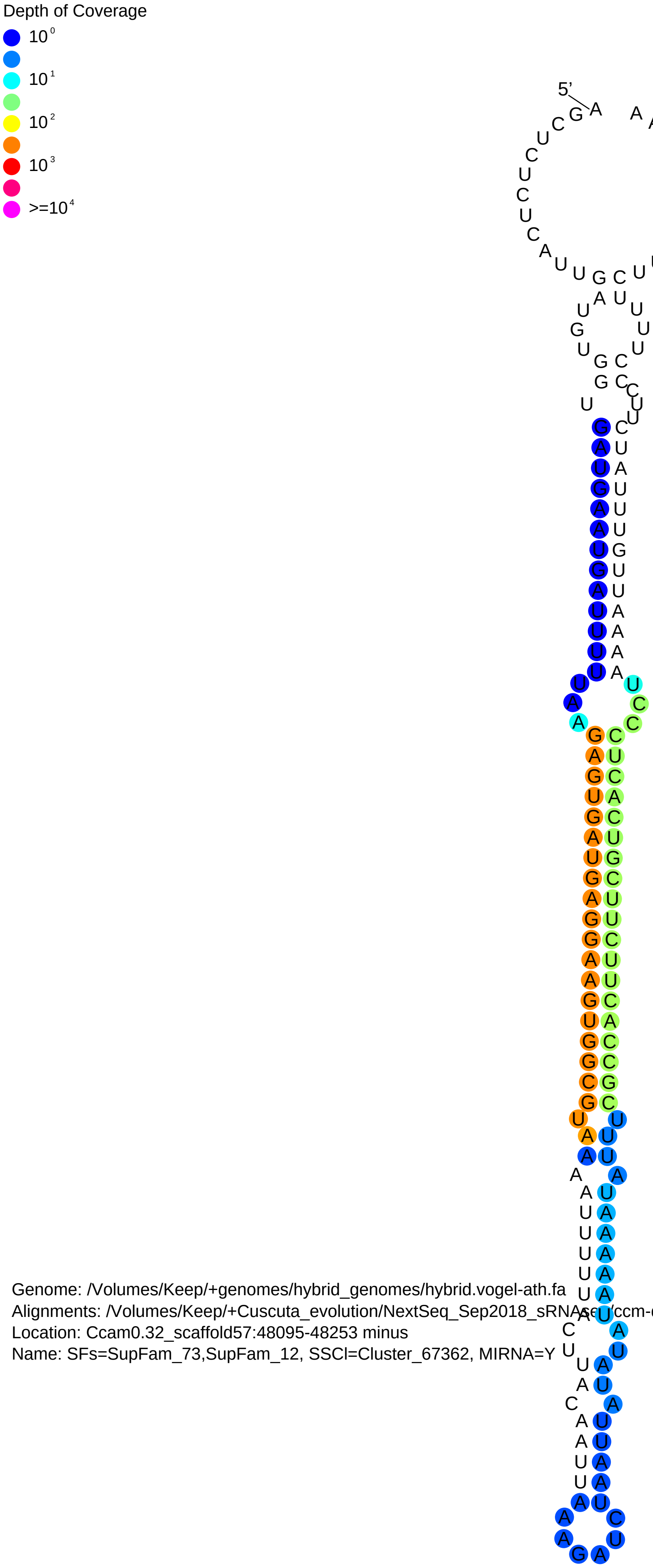

Genome: /Volumes/Keep/+genomes/hybrid_genomes/hybrid.vogel-ath.fa
Alignments: /Volumes/Keep/+Cuscuta_evolution/NextSeq_Sep2018_sRNAse[illegible]/ccm-denovo_sRNA_annotation/02out-cca.hybrid.ShortStack/merged_alig
Location: Ccam0.32_scaffold57:48095-48253 minus
Name: SFs=SupFam_73,SupFam_12, SSCl=Cluster_67362, MIRNA=Y

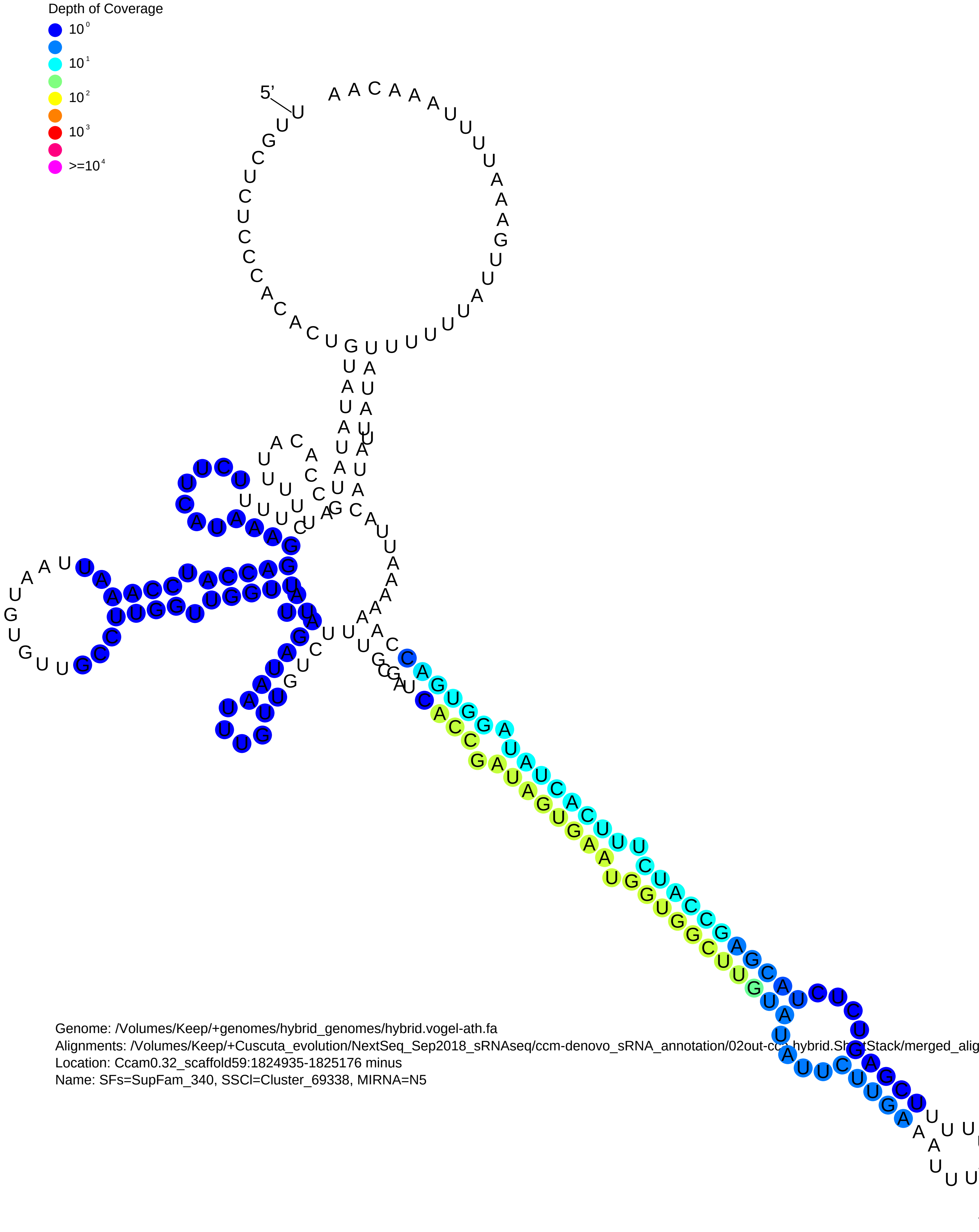

Genome: /Volumes/Keep/+genomes/hybrid_genomes/hybrid.vogel-ath.fa
Alignments: /Volumes/Keep/+Cuscuta_evolution/NextSeq_Sep2018_sRNAseq/ccm-denovo_sRNA_annotation/02out-c[illegible]hybrid.Sh[illegible]tStack/merged_alig[illegible]
Location: Ccam0.32_scaffold59:1824935-1825176 minus
Name: SFs=SupFam_340, SSCl=Cluster_69338, MIRNA=N5

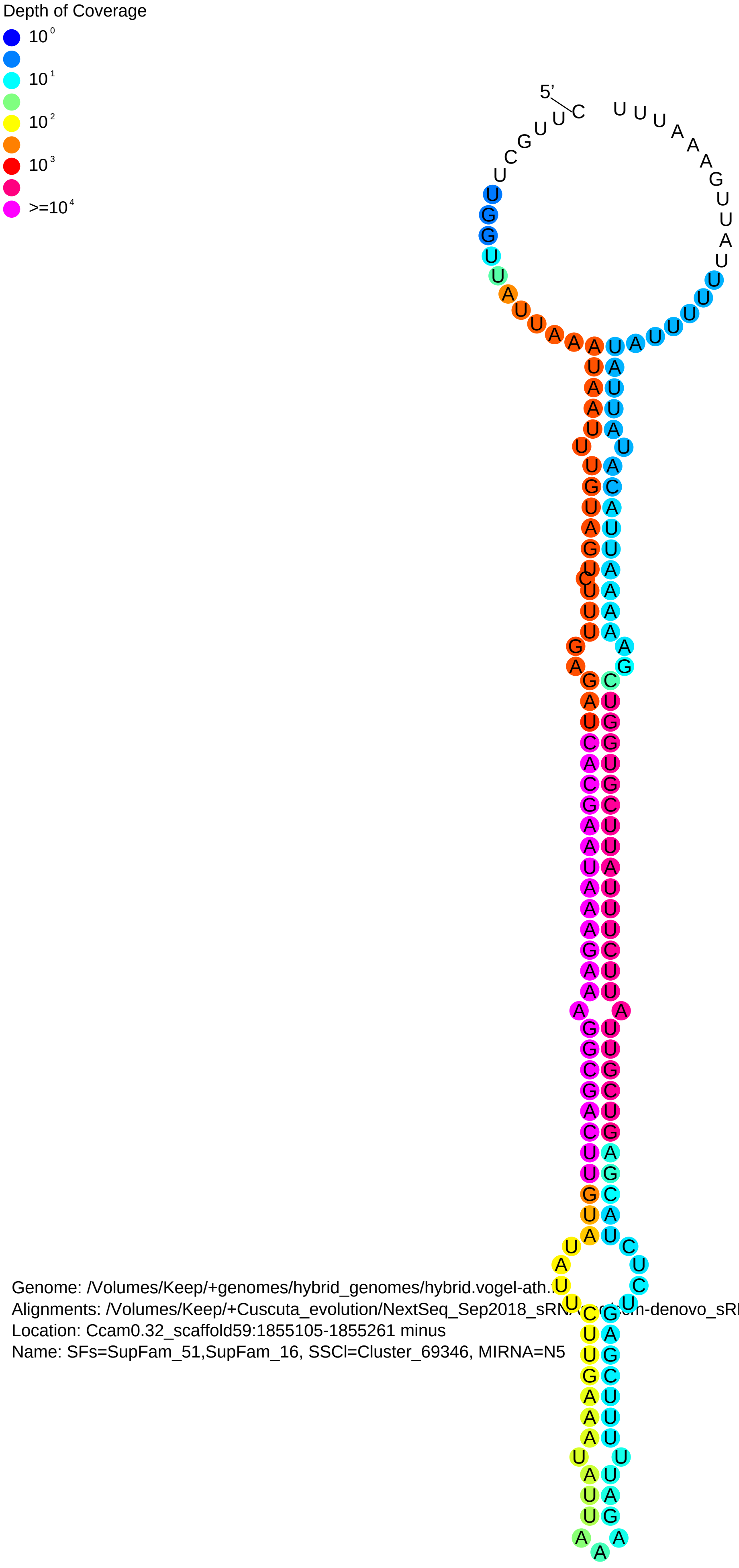

Genome: /Volumes/Keep/+genomes/hybrid_genomes/hybrid.vogel-ath.[illegible]
Alignments: /Volumes/Keep/+Cuscuta_evolution/NextSeq_Sep2018_sRN[illegible]n-denovo_sRNA_annotation/02out-cca.hybrid.ShortStack/merged_alig[illegible]
Location: Ccam0.32_scaffold59:1855105-1855261 minus
Name: SFs=SupFam_51,SupFam_16, SSCl=Cluster_69346, MIRNA=N5

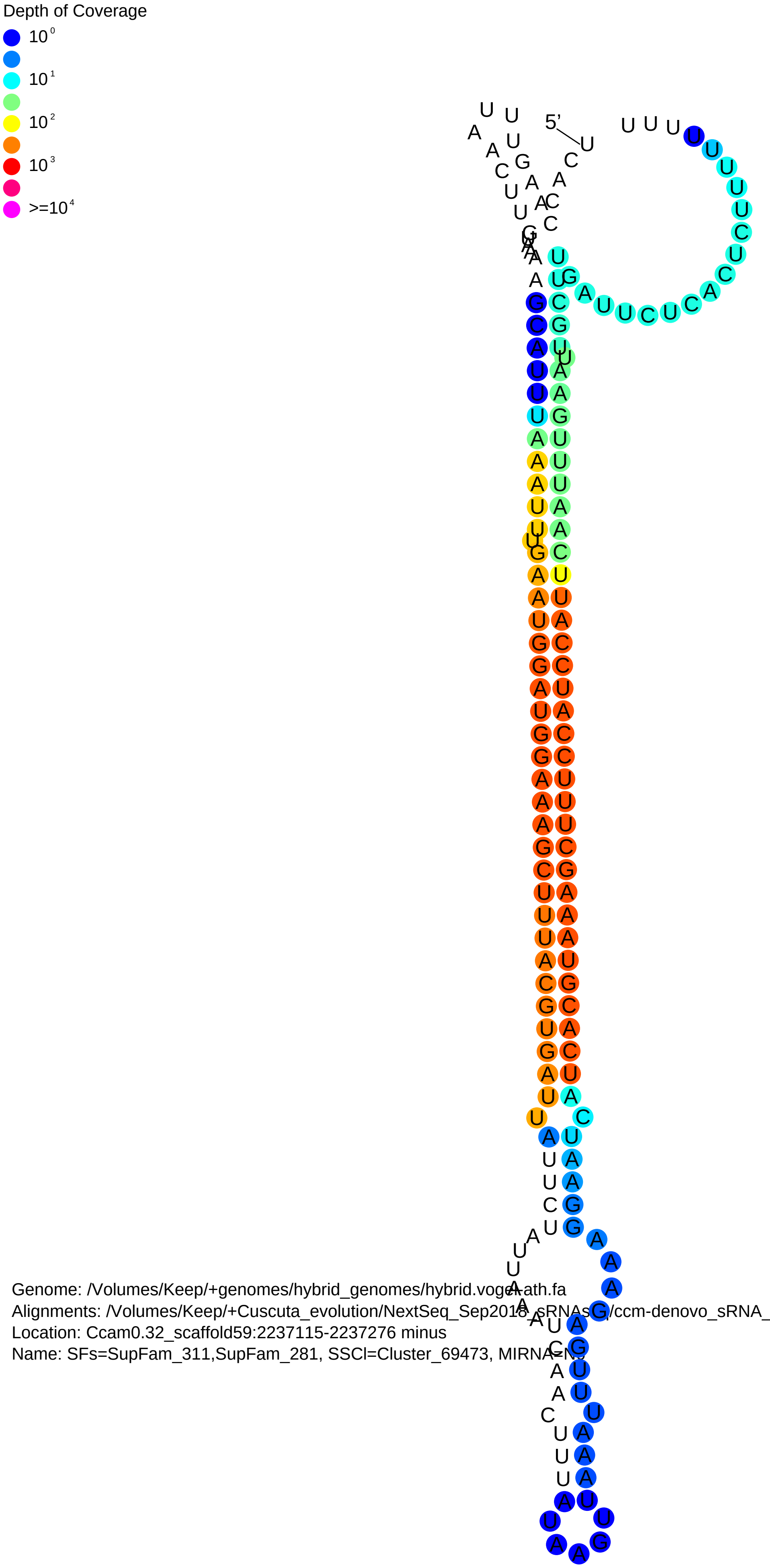

Genome: /Volumes/Keep/+genomes/hybrid_genomes/hybrid.vogel-ath.fa
Alignments: /Volumes/Keep/+Cuscuta_evolution/NextSeq_Sep2018_sRNAs/ccm-denovo_sRNA_annotation/02out-cca.hybrid.ShortStack/merged_alig
Location: Ccam0.32_scaffold59:2237115-2237276 minus
Name: SFs=SupFam_311,SupFam_281, SSCl=Cluster_69473, MIRNA=N

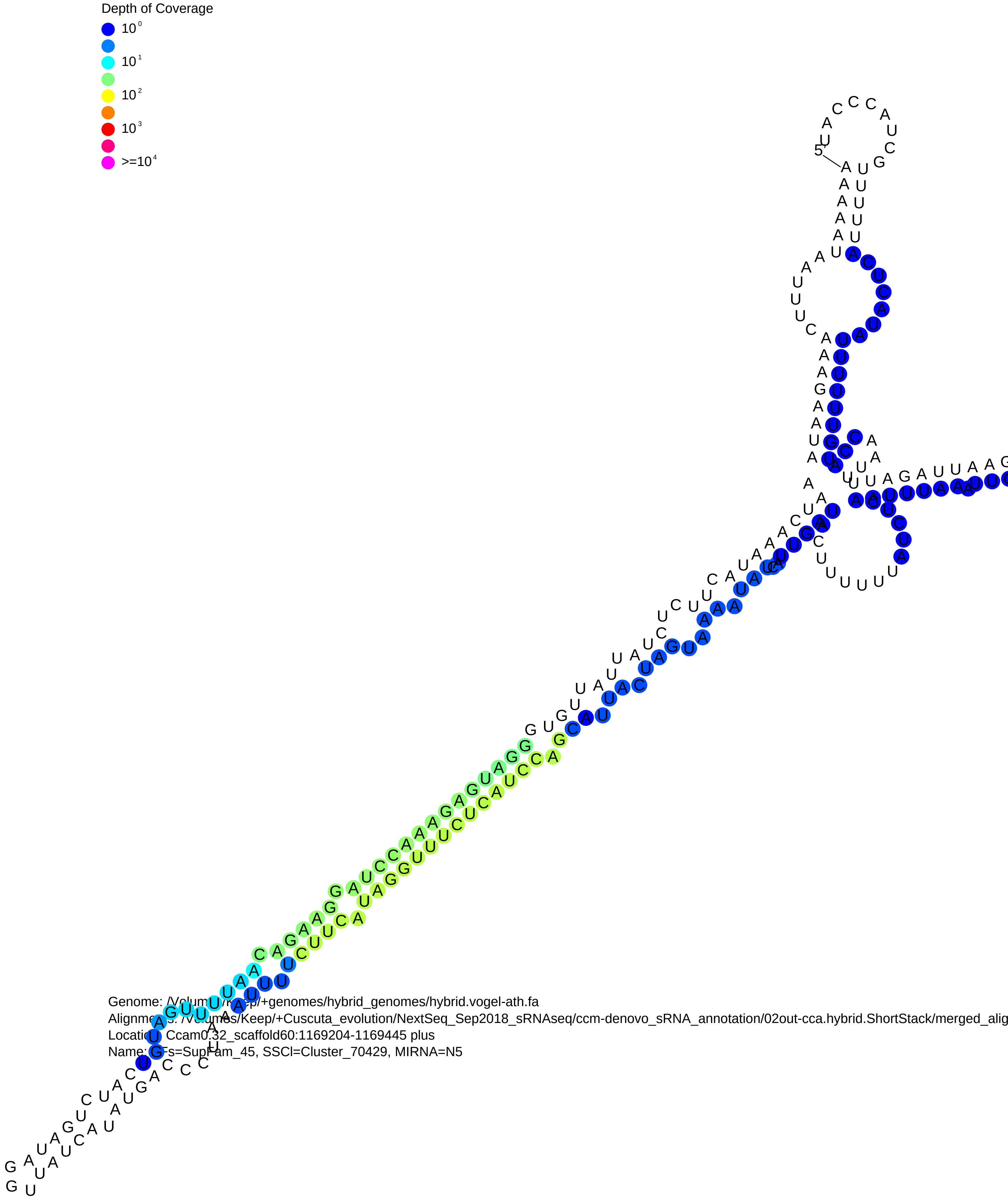
Depth of Coverage
10^0
10^1
10^2
10^3
$>=10^4$
Genome: /Volum /K ep/+genomes/hybrid_genomes/hybrid.vogel-ath.fa
Alignme s: /V umes/Keep/+Cuscuta_evolution/NextSeq_Sep2018_sRNAseq/ccm-denovo_sRNA_annotation/02out-cca.hybrid.ShortStack/merged_alig
Locatio Ccam0.32_scaffold60:1169204-1169445 plus
Name: Fs=SupFam_45, SSCl=Cluster_70429, MIRNA=N5

Depth of Coverage

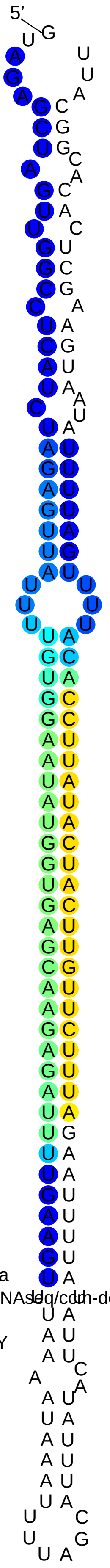

Genome: /Volumes/Keep/+genomes/hybrid_genomes/hybrid.vogel-ath.fa
Alignments: /Volumes/Keep/+Cuscuta_evolution/NextSeq_Sep2018_sRNAseq/cca-denovo_sRNA_annotation/02out-cca.hybrid.ShortStack/merged_alig
Location: Ccam0.32_scaffold5:103937-104093 minus
Name: SFs=SupFam_137,SupFam_341, SSCl=Cluster_7679, MIRNA=Y

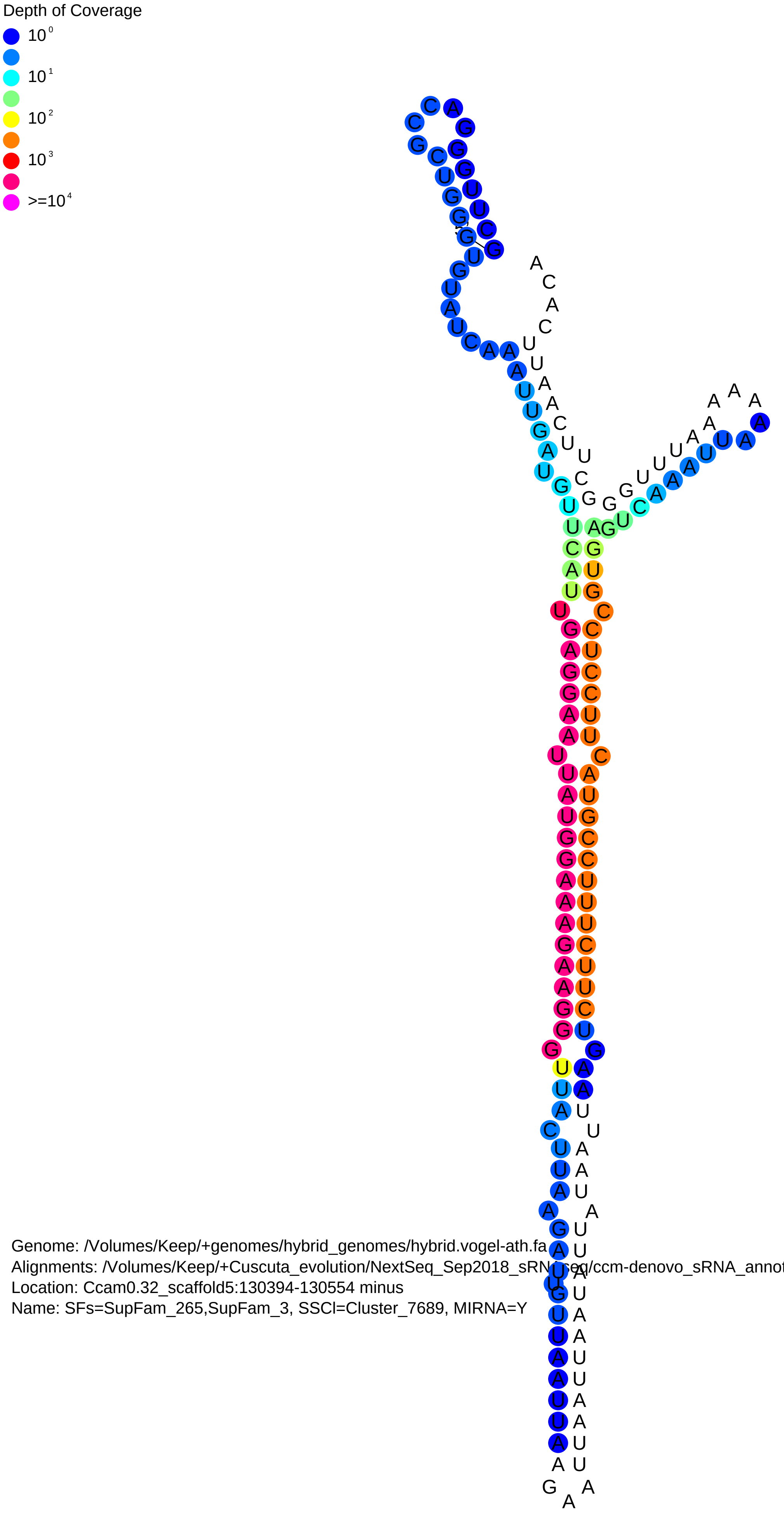

Genome: /Volumes/Keep/+genomes/hybrid_genomes/hybrid.vogel-ath.fa
Alignments: /Volumes/Keep/+Cuscuta_evolution/NextSeq_Sep2018_sRNAseq/ccm-denovo_sRNA_annotation/02out-cca.hybrid.ShortStack/merged_alig
Location: Ccam0.32_scaffold5:130394-130554 minus
Name: SFs=SupFam_265,SupFam_3, SSCl=Cluster_7689, MIRNA=Y

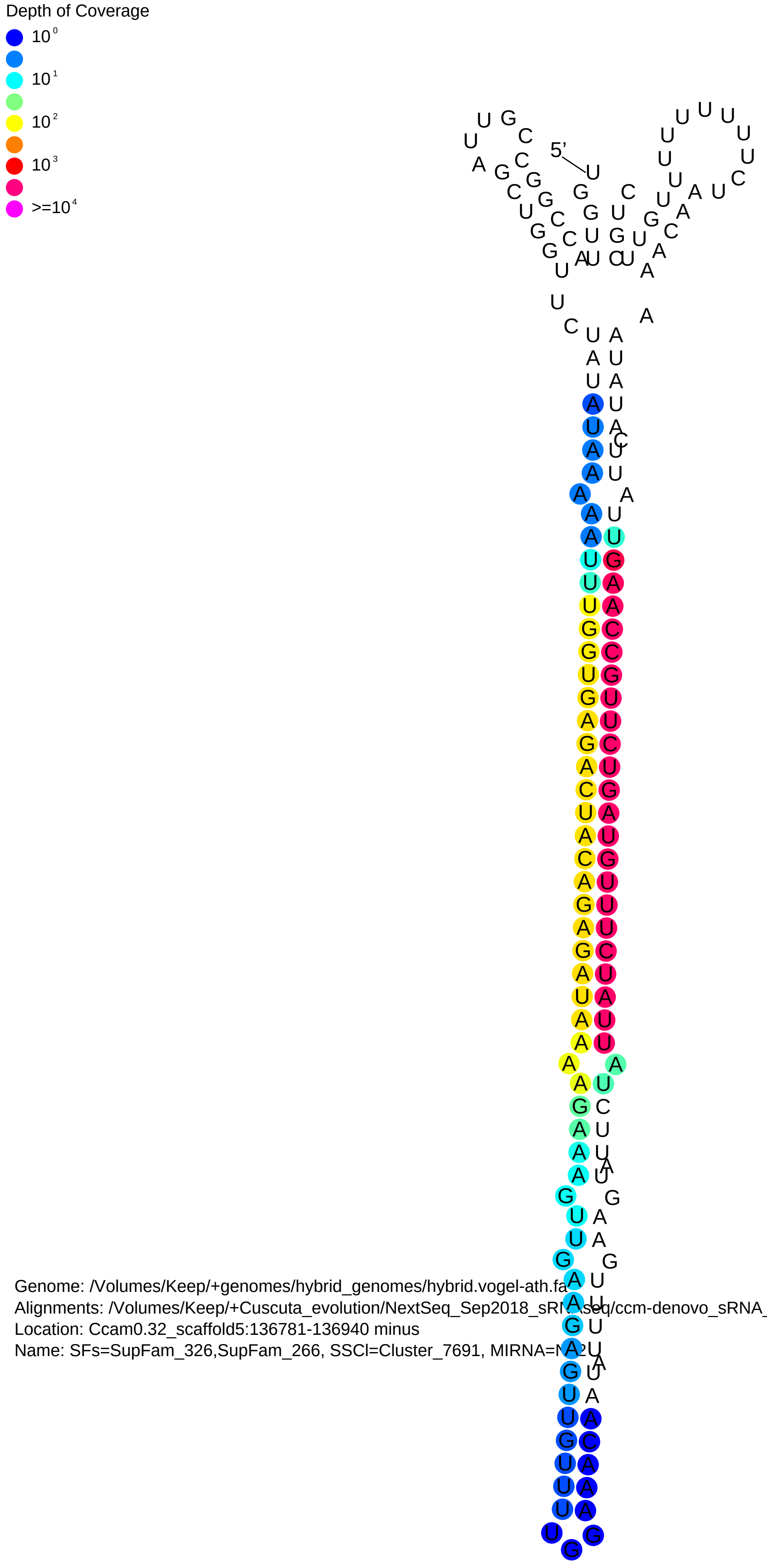

Genome: /Volumes/Keep/+genomes/hybrid_genomes/hybrid.vogel-ath.fa
Alignments: /Volumes/Keep/+Cuscuta_evolution/NextSeq_Sep2018_sR[illegible]seq/ccm-denovo_sRNA_annotation/02out-cca.hybrid.ShortStack/merged_alig
Location: Ccam0.32_scaffold5:136781-136940 minus
Name: SFs=SupFam_326,SupFam_266, SSCl=Cluster_7691, MIRNA=N[illegible]

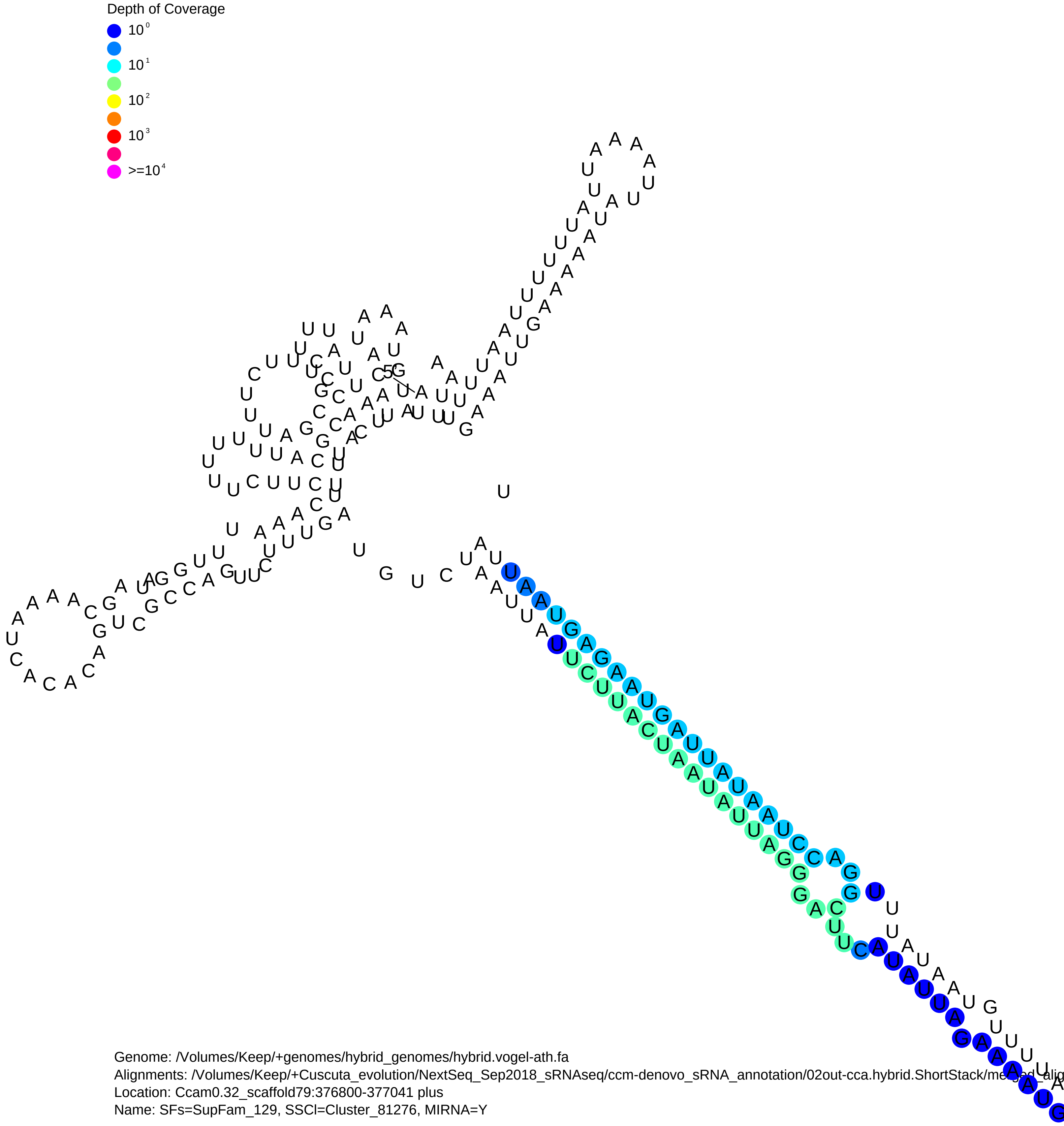

Genome: /Volumes/Keep/+genomes/hybrid_genomes/hybrid.vogel-ath.fa
Alignments: /Volumes/Keep/+Cuscuta_evolution/NextSeq_Sep2018_sRNAseq/ccm-denovo_sRNA_annotation/02out-cca.hybrid.ShortStack/me[illegible]
Location: Ccam0.32_scaffold79:376800-377041 plus
Name: SFs=SupFam_129, SSCl=Cluster_81276, MIRNA=Y

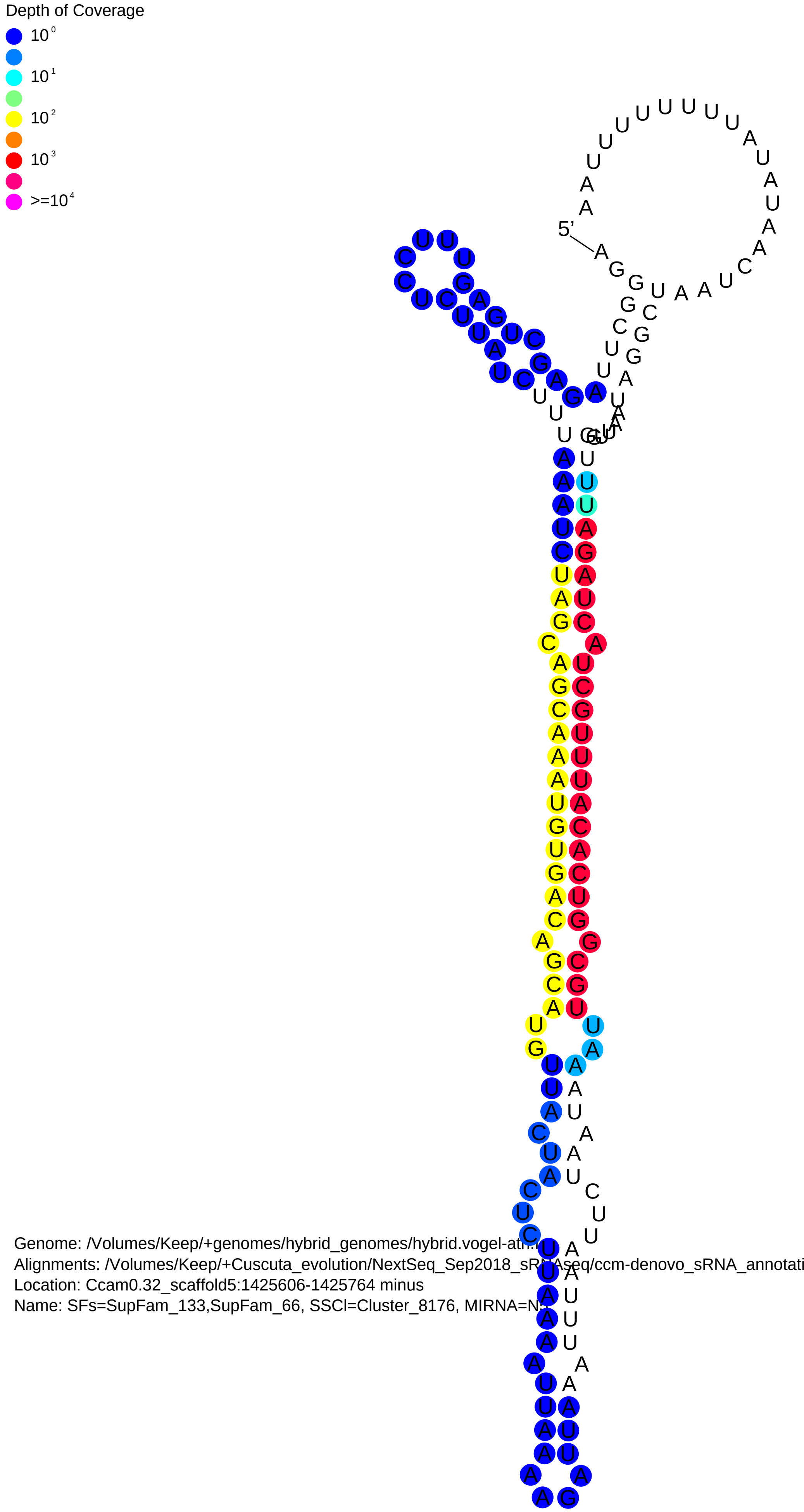

Genome: /Volumes/Keep/+genomes/hybrid_genomes/hybrid.vogel-at[illegible]
Alignments: /Volumes/Keep/+Cuscuta_evolution/NextSeq_Sep2018_sRNAseq/ccm-denovo_sRNA_annotation/02out-cca.hybrid.ShortStack/merged_alig
Location: Ccam0.32_scaffold5:1425606-1425764 minus
Name: SFs=SupFam_133,SupFam_66, SSCl=Cluster_8176, MIRNA=N;

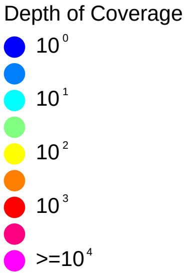

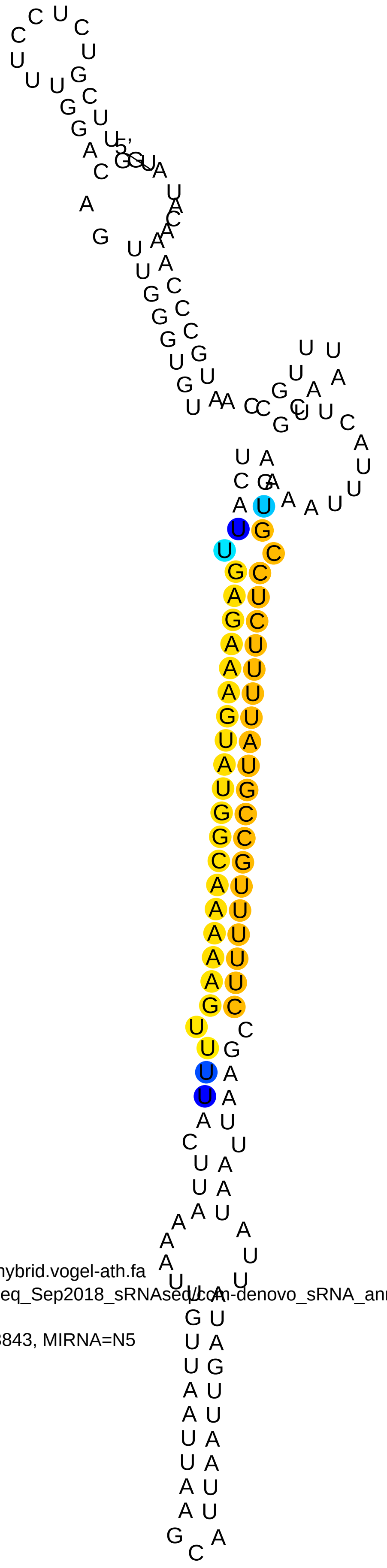

Genome: /Volumes/Keep/+genomes/hybrid_genomes/hybrid.vogel-ath.fa
Alignments: /Volumes/Keep/+Cuscuta_evolution/NextSeq_Sep2018_sRNAseq/cca-denovo_sRNA_annotation/02out-cca.hybrid.ShortStack/merged_alig
Location: Ccam0.32_scaffold83:65925-66083 plus
Name: SFs=SupFam_3,SupFam_22, SSCl=Cluster_83843, MIRNA=N5

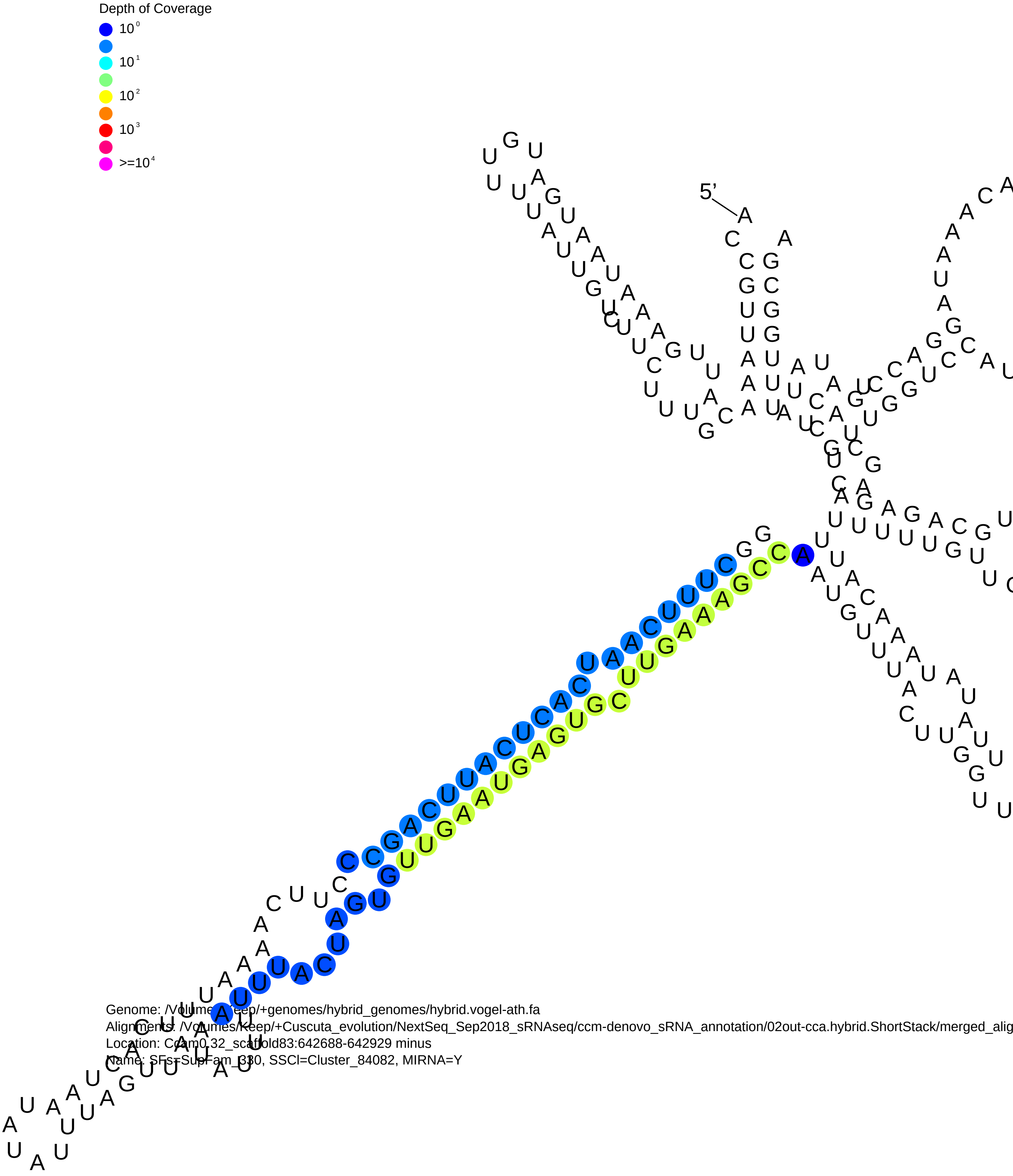

Genome: /Volumes/Keep/+genomes/hybrid_genomes/hybrid.vogel-ath.fa
Alignments: /Volumes/Keep/+Cuscuta_evolution/NextSeq_Sep2018_sRNAseq/ccm-denovo_sRNA_annotation/02out-cca.hybrid.ShortStack/merged_alig
Location: Ccam0.32_scaffold83:642688-642929 minus
Name: SFs=SupFam_330, SSCl=Cluster_84082, MIRNA=Y

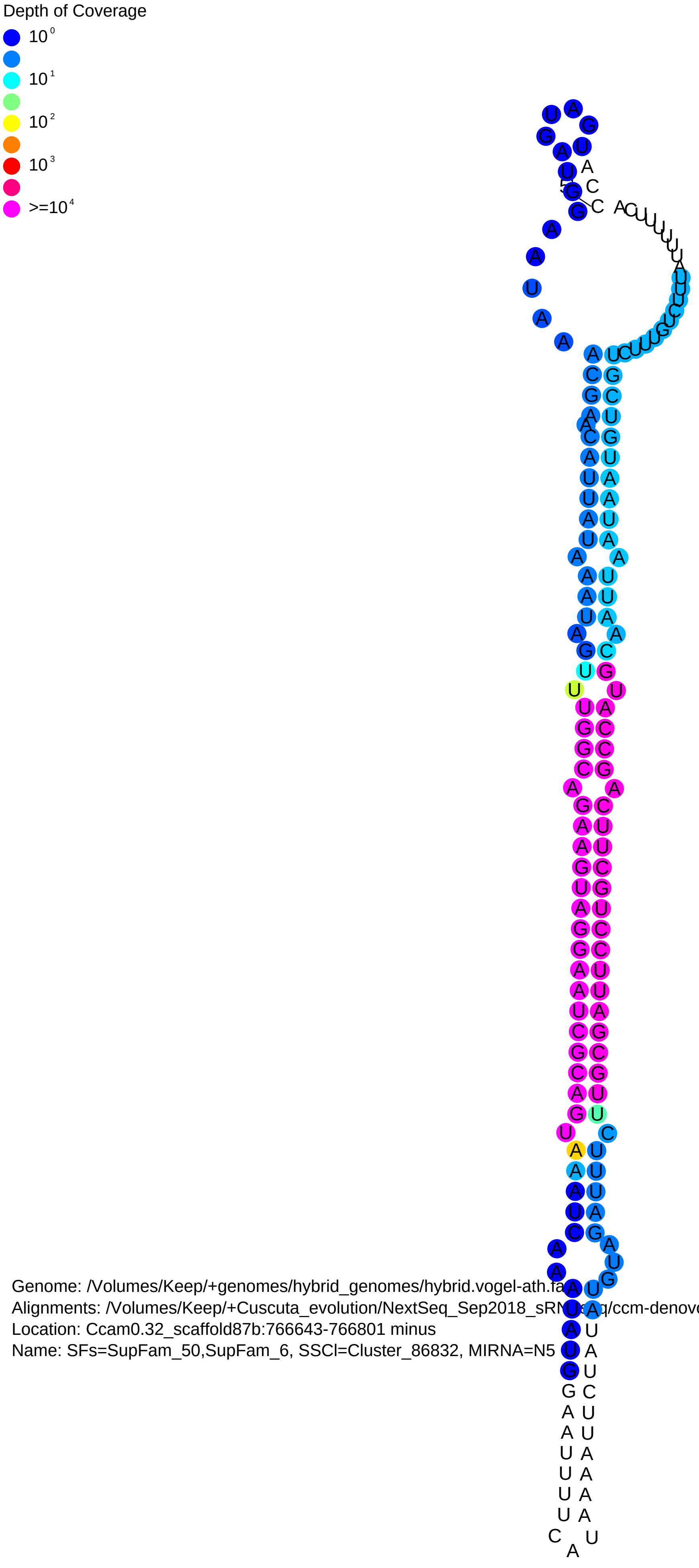

Genome: /Volumes/Keep/+genomes/hybrid_genomes/hybrid.vogel-ath.fa[illegible]
Alignments: /Volumes/Keep/+Cuscuta_evolution/NextSeq_Sep2018_sRN[illegible]q/ccm-denovo_sRNA_annotation/02out-cca.hybrid.ShortStack/merged_alig[illegible]
Location: Ccam0.32_scaffold87b:766643-766801 minus
Name: SFs=SupFam_50,SupFam_6, SSCl=Cluster_86832, MIRNA=N5

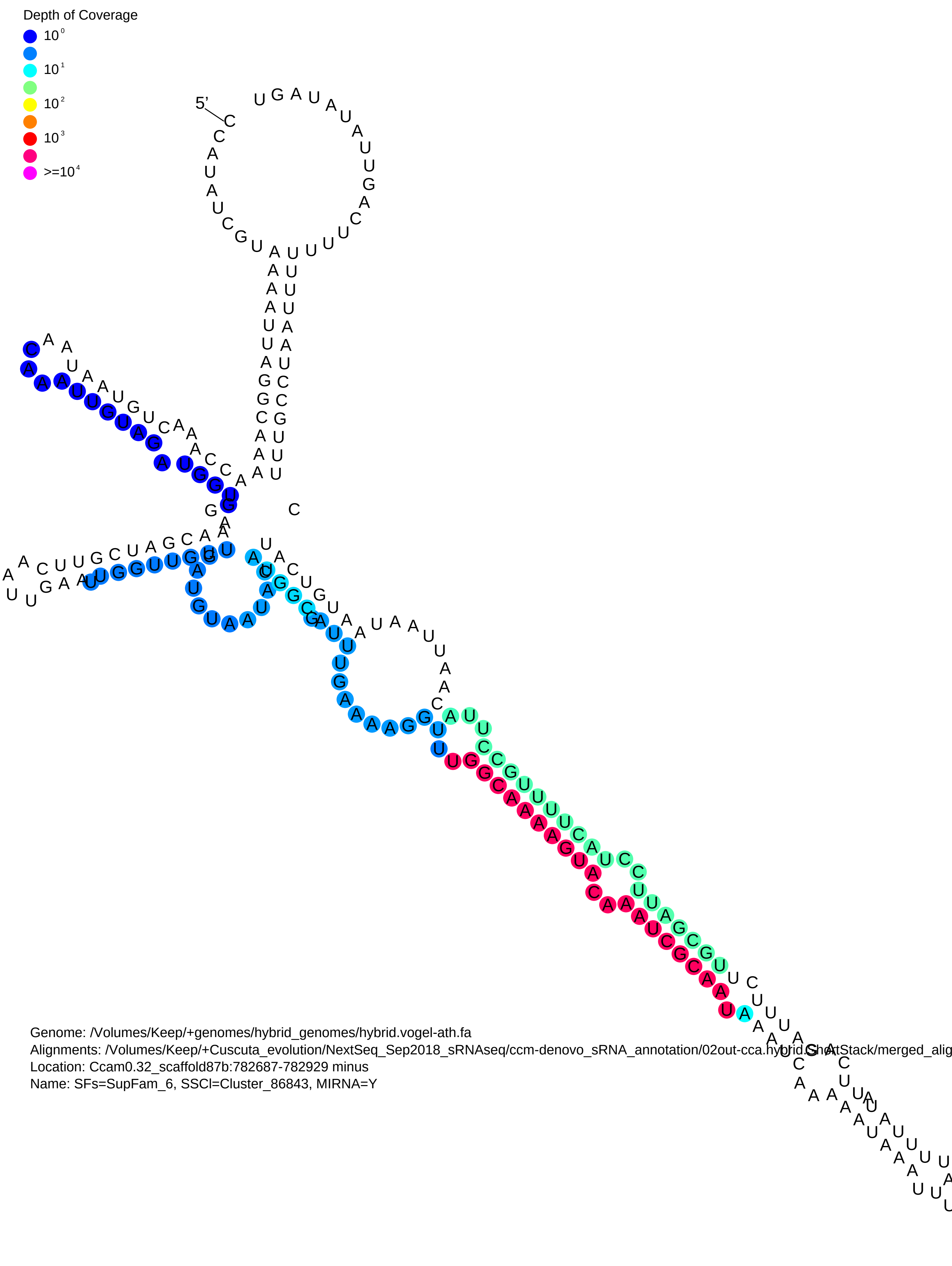

Genome: /Volumes/Keep/+genomes/hybrid_genomes/hybrid.vogel-ath.fa
Alignments: /Volumes/Keep/+Cuscuta_evolution/NextSeq_Sep2018_sRNAseq/ccm-denovo_sRNA_annotation/02out-cca.hybrid.ShortStack/merged_alig
Location: Ccam0.32_scaffold87b:782687-782929 minus
Name: SFs=SupFam_6, SSCl=Cluster_86843, MIRNA=Y

Depth of Coverage

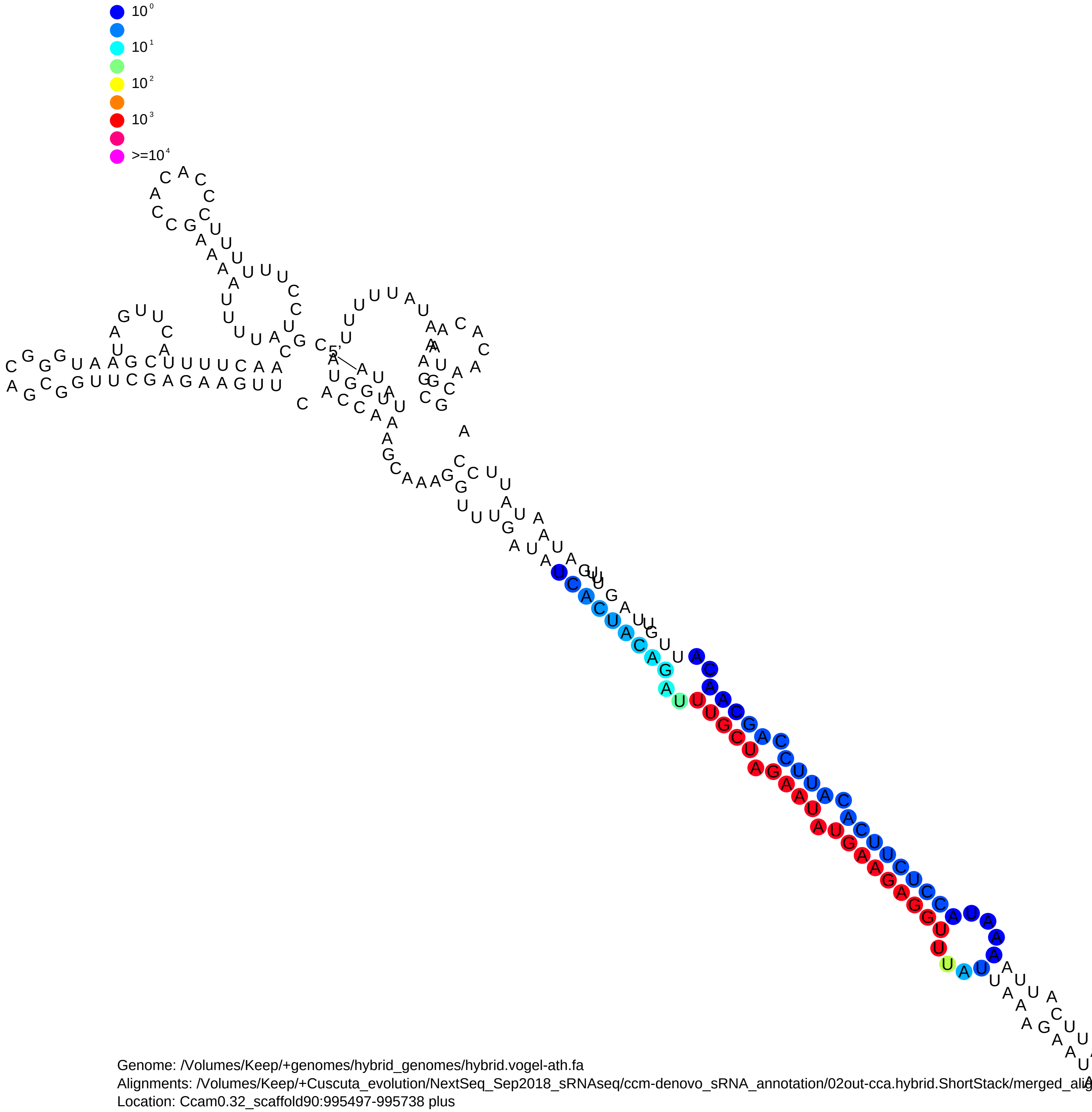

Genome: /Volumes/Keep/+genomes/hybrid_genomes/hybrid.vogel-ath.fa
Alignments: /Volumes/Keep/+Cuscuta_evolution/NextSeq_Sep2018_sRNAseq/ccm-denovo_sRNA_annotation/02out-cca.hybrid.ShortStack/merged_ali
Location: Ccam0.32_scaffold90:995497-995738 plus
Name: SFs=SupFam_273, SSCl=Cluster_88686, MIRNA=N5

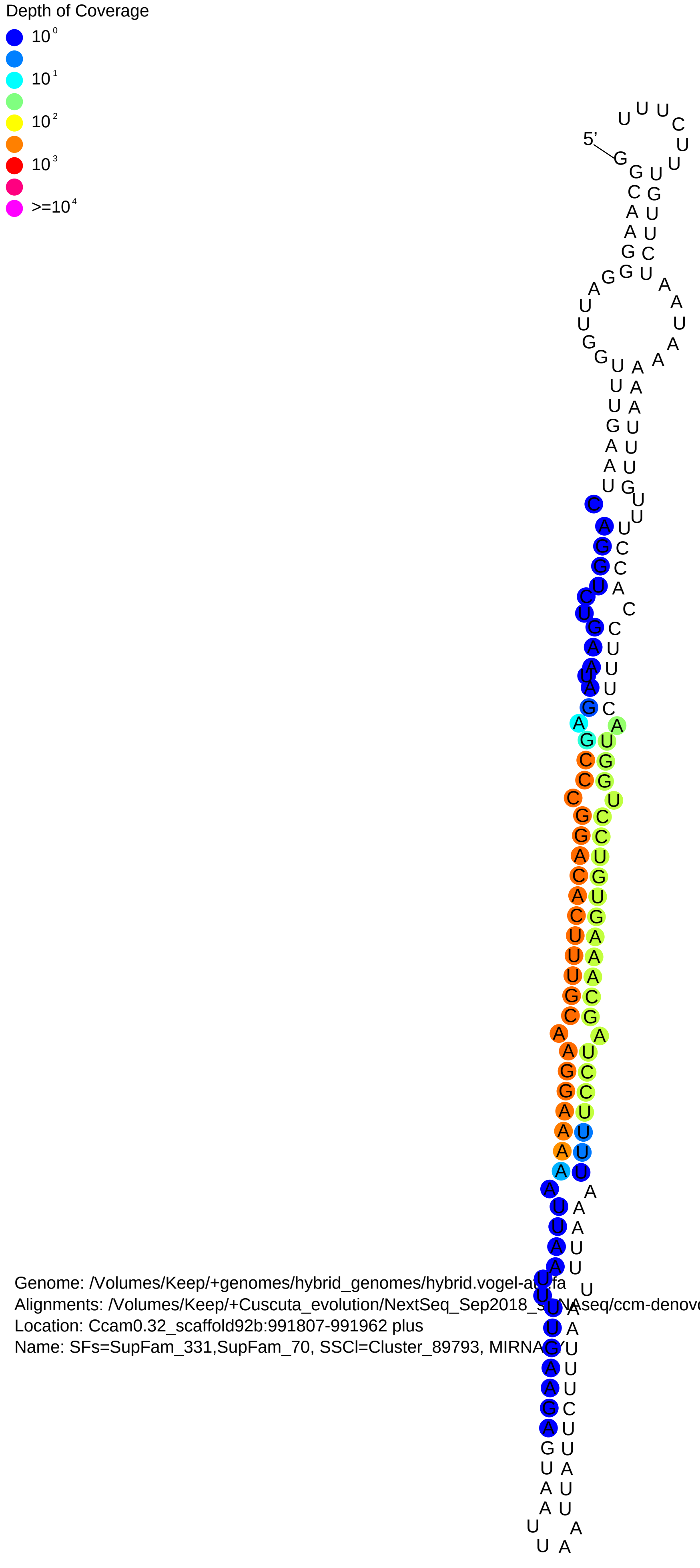

Genome: /Volumes/Keep/+genomes/hybrid_genomes/hybrid.vogel-a[illegible].fa
Alignments: /Volumes/Keep/+Cuscuta_evolution/NextSeq_Sep2018_s[illegible]Aseq/ccm-denovo_sRNA_annotation/02out-cca.hybrid.ShortStack/merged_alig
Location: Ccam0.32_scaffold92b:991807-991962 plus
Name: SFs=SupFam_331,SupFam_70, SSCl=Cluster_89793, MIRNA[illegible]

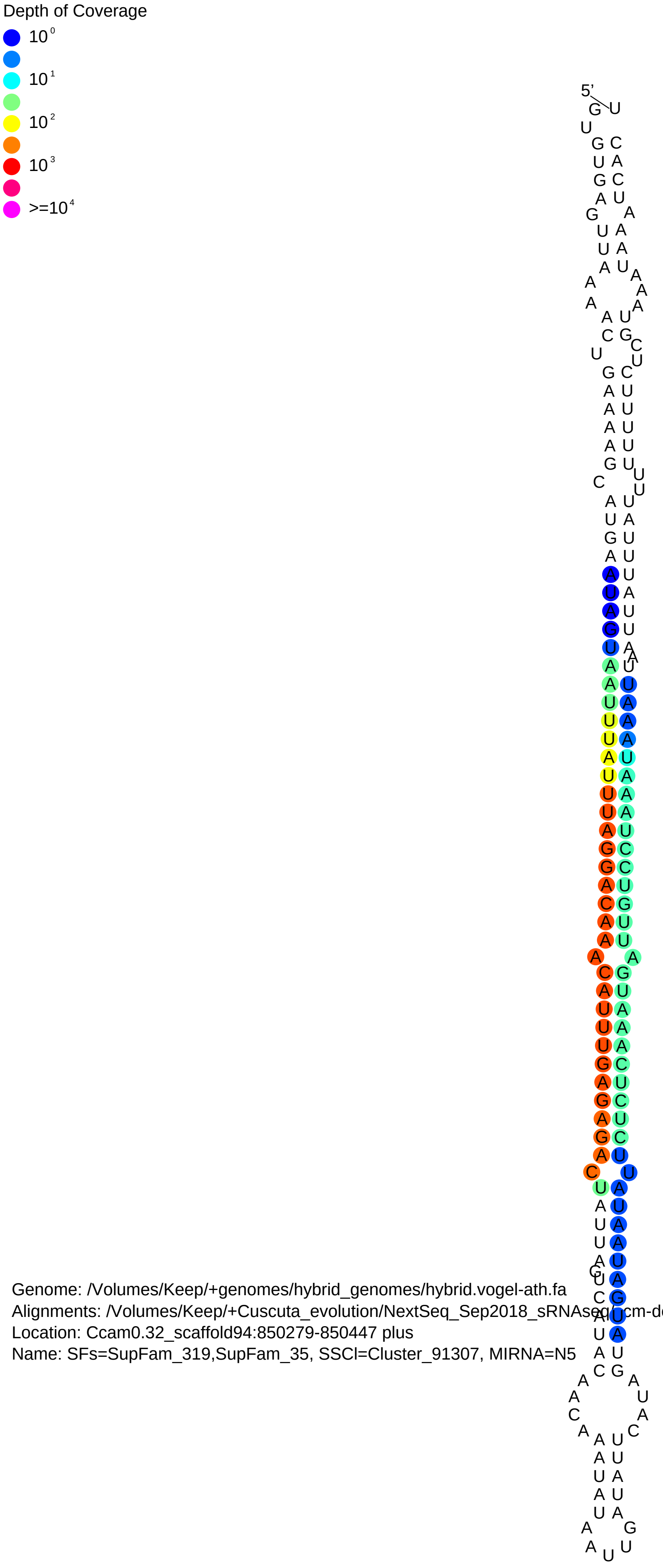

Genome: /Volumes/Keep/+genomes/hybrid_genomes/hybrid.vogel-ath.fa
Alignments: /Volumes/Keep/+Cuscuta_evolution/NextSeq_Sep2018_sRNAseq/ccm-denovo_sRNA_annotation/02out-cca.hybrid.ShortStack/merged_alig
Location: Ccam0.32_scaffold94:850279-850447 plus
Name: SFs=SupFam_319,SupFam_35, SSCl=Cluster_91307, MIRNA=N5

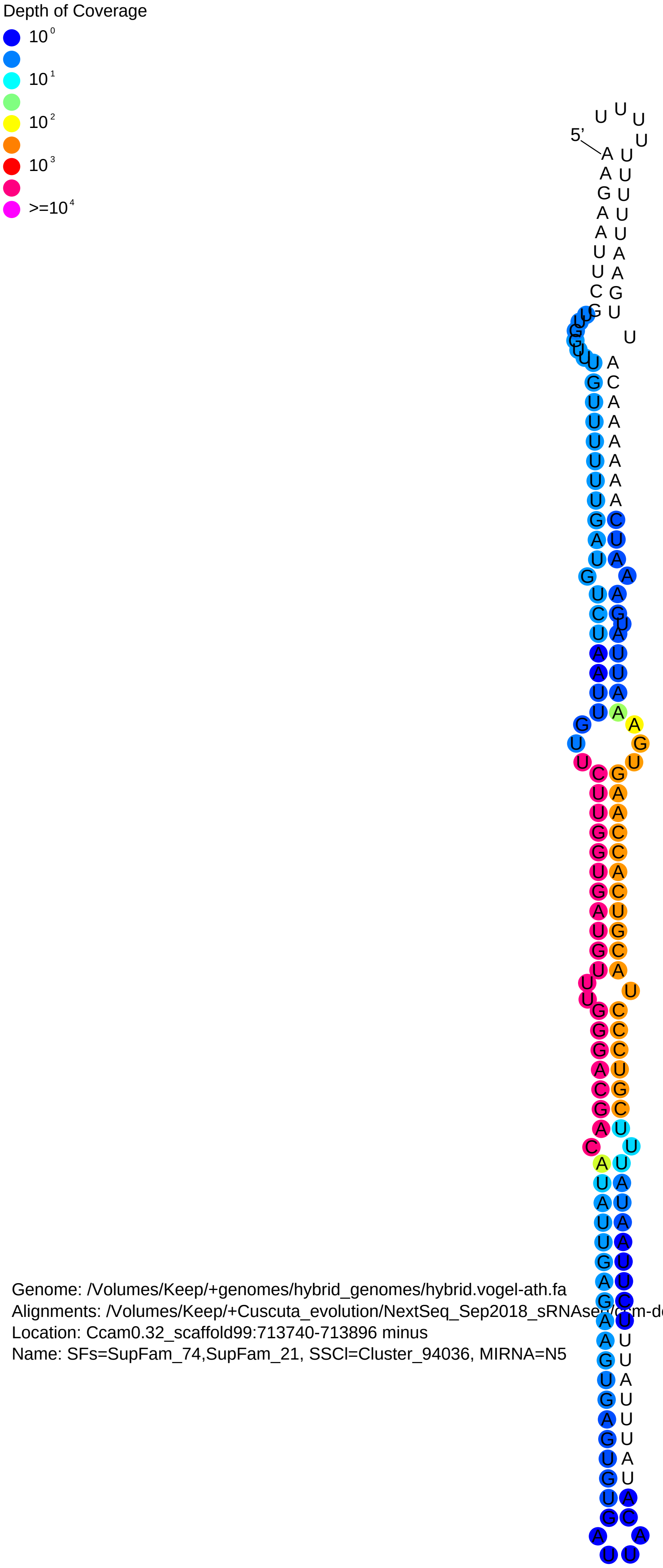

Genome: /Volumes/Keep/+genomes/hybrid_genomes/hybrid.vogel-ath.fa
Alignments: /Volumes/Keep/+Cuscuta_evolution/NextSeq_Sep2018_sRNAse[illegible]m-denovo_sRNA_annotation/02out-cca.hybrid.ShortStack/merged_alig
Location: Ccam0.32_scaffold99:713740-713896 minus
Name: SFs=SupFam_74,SupFam_21, SSCl=Cluster_94036, MIRNA=N5

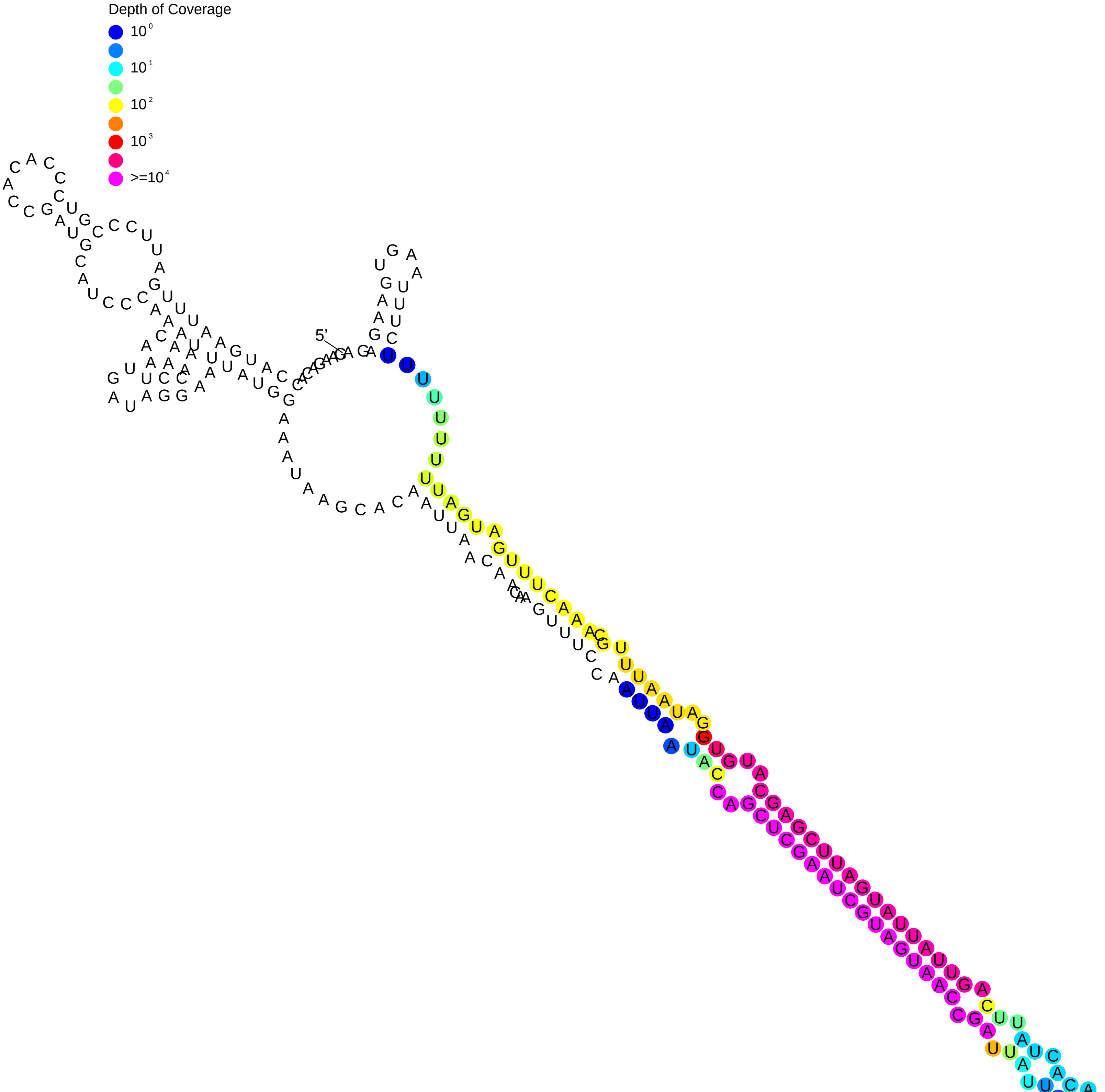

Genome: /Volumes/Keep/+genomes/hybrid_genomes/hybrid.vogel-ath.fa
Alignments: /Volumes/Keep/+Cuscuta_evolution/NextSeq_Sep2018_sRNAseq/ccm-denovo_sRNA_annotation/02out-cca.hybrid.ShortStack/merg[illegible]
Location: Ccam0.32_scaffold108:146570-146812 minus
Name: SFs=SupFam_260, SSCl=Cluster_97391, MIRNA=N5

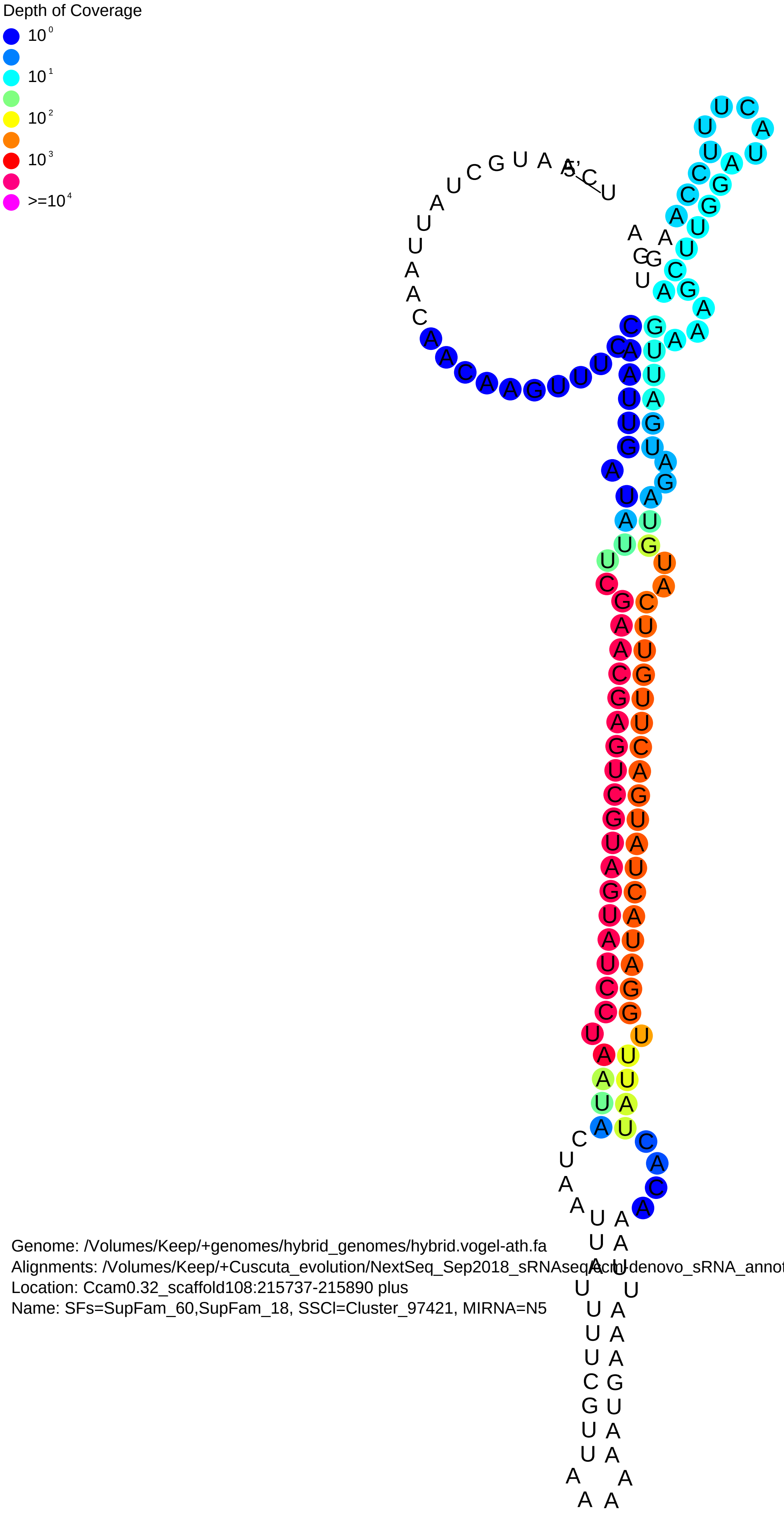

Genome: /Volumes/Keep/+genomes/hybrid_genomes/hybrid.vogel-ath.fa
Alignments: /Volumes/Keep/+Cuscuta_evolution/NextSeq_Sep2018_sRNAseq/cca[illegible]-denovo_sRNA_annotation/02out-cca.hybrid.ShortStack/merged_alig
Location: Ccam0.32_scaffold108:215737-215890 plus
Name: SFs=SupFam_60,SupFam_18, SSCl=Cluster_97421, MIRNA=N5

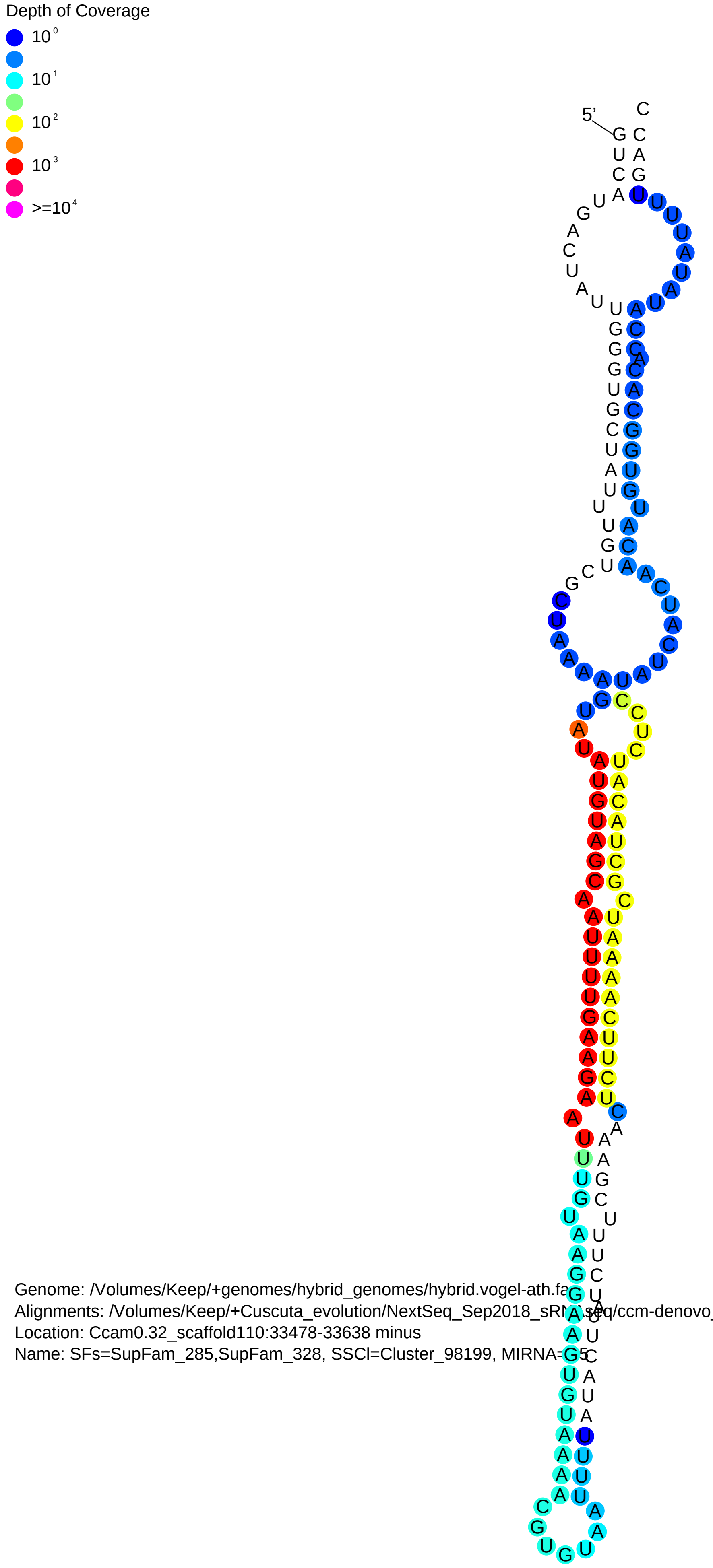

Genome: /Volumes/Keep/+genomes/hybrid_genomes/hybrid.vogel-ath.fa
Alignments: /Volumes/Keep/+Cuscuta_evolution/NextSeq_Sep2018_sRNAseq/ccm-denovo_sRNA_annotation/02out-cca.hybrid.ShortStack/merged_alig
Location: Ccam0.32_scaffold110:33478-33638 minus
Name: SFs=SupFam_285,SupFam_328, SSCl=Cluster_98199, MIRNA=[illegible]

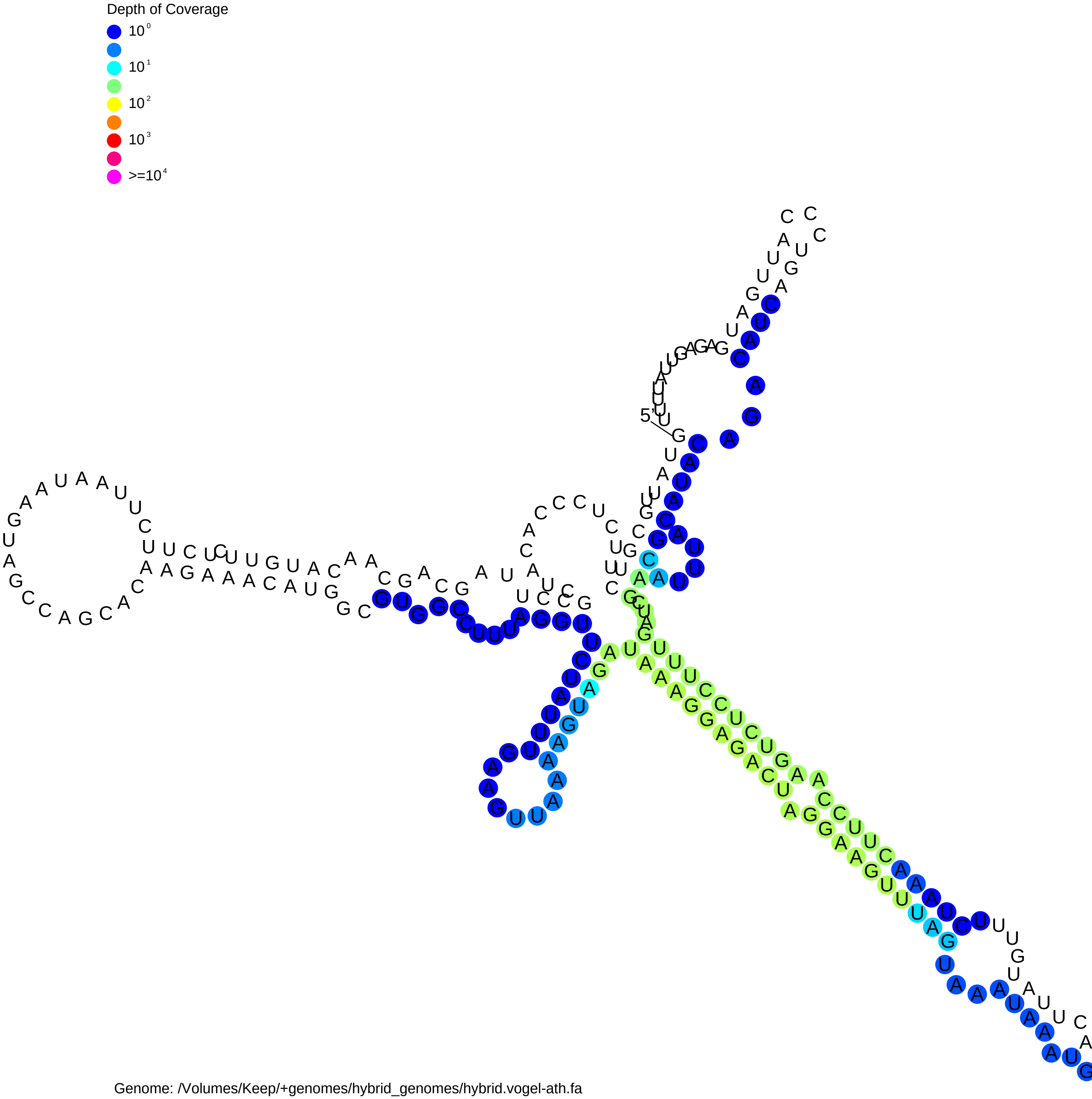

Genome: /Volumes/Keep/+genomes/hybrid_genomes/hybrid.vogel-ath.fa
Alignments: /Volumes/Keep/+Cuscuta_evolution/NextSeq_Sep2018_sRNAseq/ccm-denovo_sRNA_annotation/02out-cca.hybrid.ShortStack/merged_alig
Location: Ccam0.32_scaffold110:66589-66830 plus
Name: SFs=SupFam_339, SSCl=Cluster_98206, MIRNA=N14

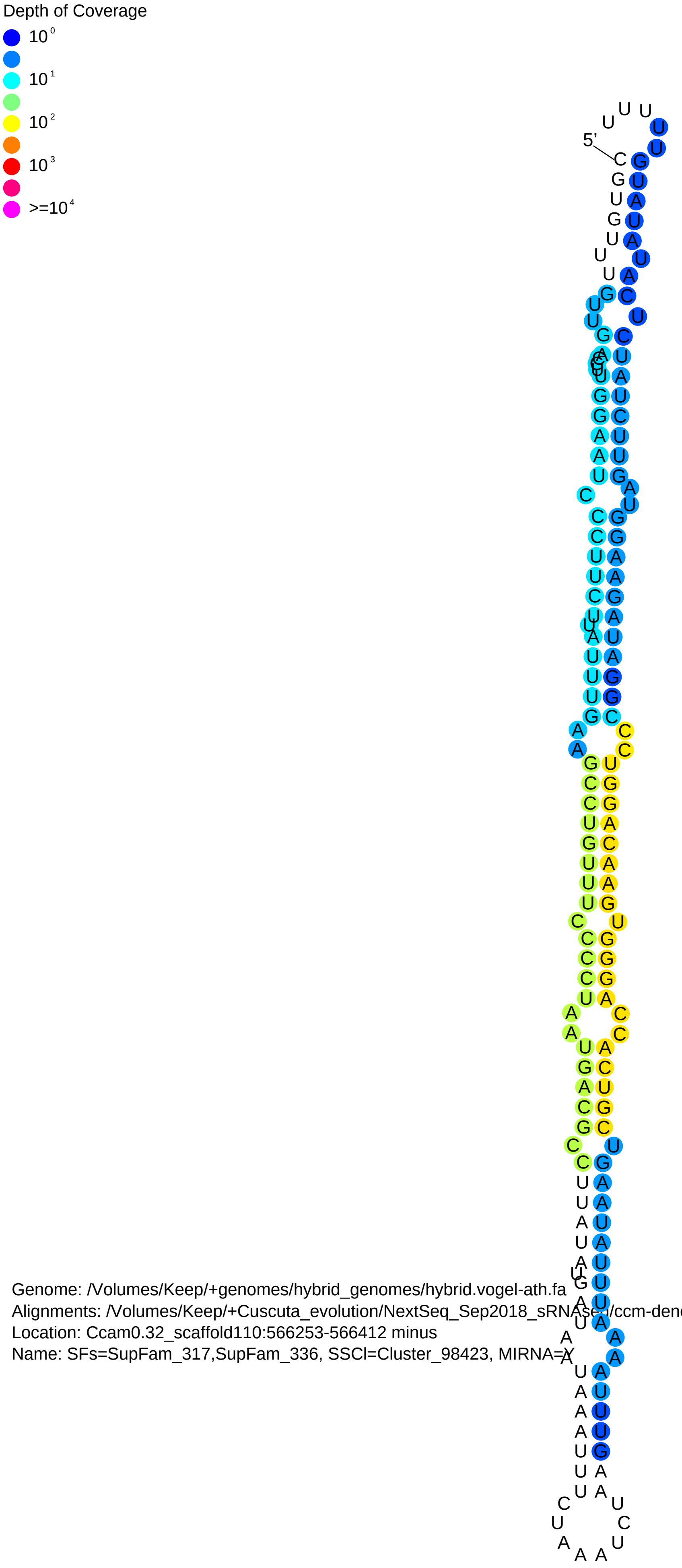

Genome: /Volumes/Keep/+genomes/hybrid_genomes/hybrid.vogel-ath.fa
Alignments: /Volumes/Keep/+Cuscuta_evolution/NextSeq_Sep2018_sRNAse[illegible]/ccm-denovo_sRNA_annotation/02out-cca.hybrid.ShortStack/merged_alig
Location: Ccam0.32_scaffold110:566253-566412 minus
Name: SFs=SupFam_317,SupFam_336, SSCl=Cluster_98423, MIRNA=Y

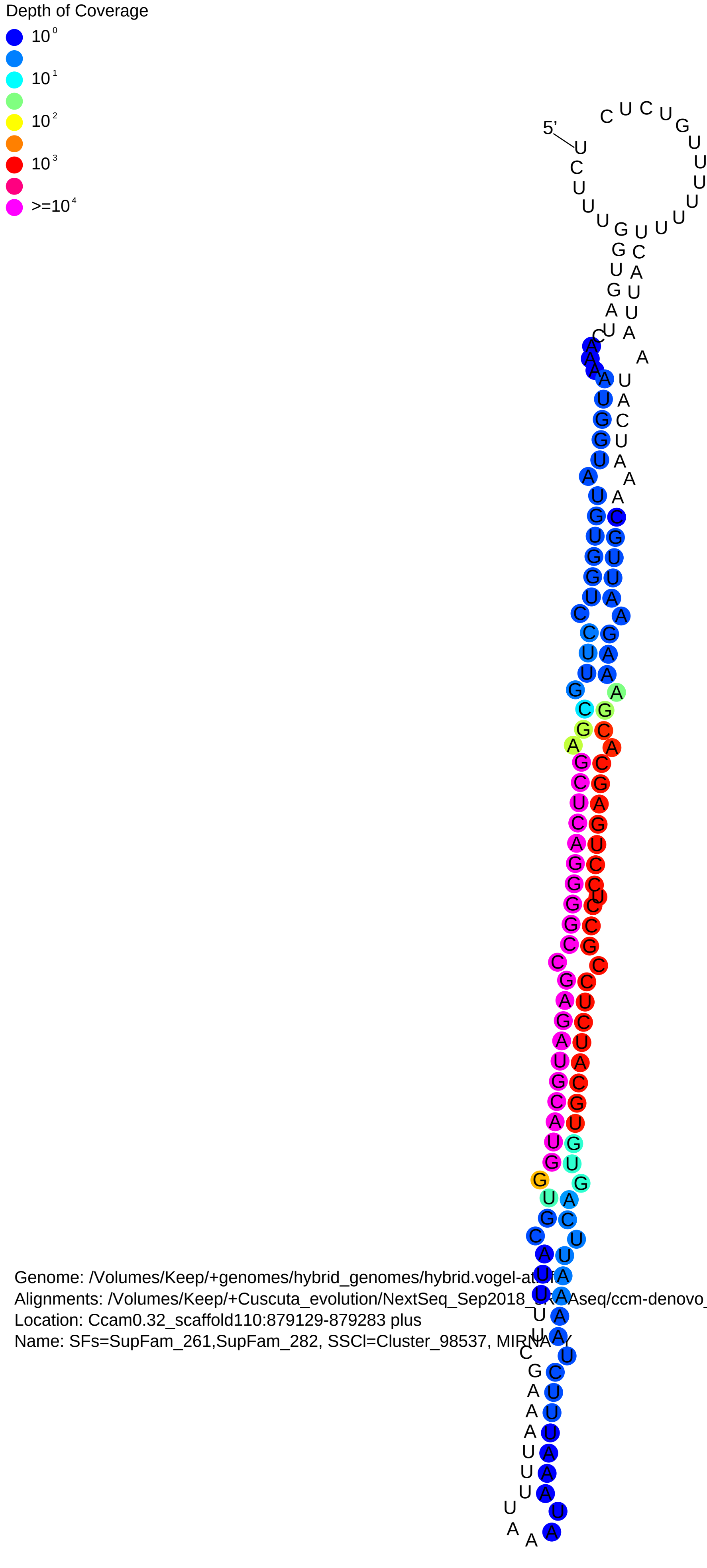

Genome: /Volumes/Keep/+genomes/hybrid_genomes/hybrid.vogel-at[illegible]
Alignments: /Volumes/Keep/+Cuscuta_evolution/NextSeq_Sep2018_[illegible]Aseq/ccm-denovo_sRNA_annotation/02out-cca.hybrid.ShortStack/merged_alig
Location: Ccam0.32_scaffold110:879129-879283 plus
Name: SFs=SupFam_261,SupFam_282, SSCl=Cluster_98537, MIR[illegible]

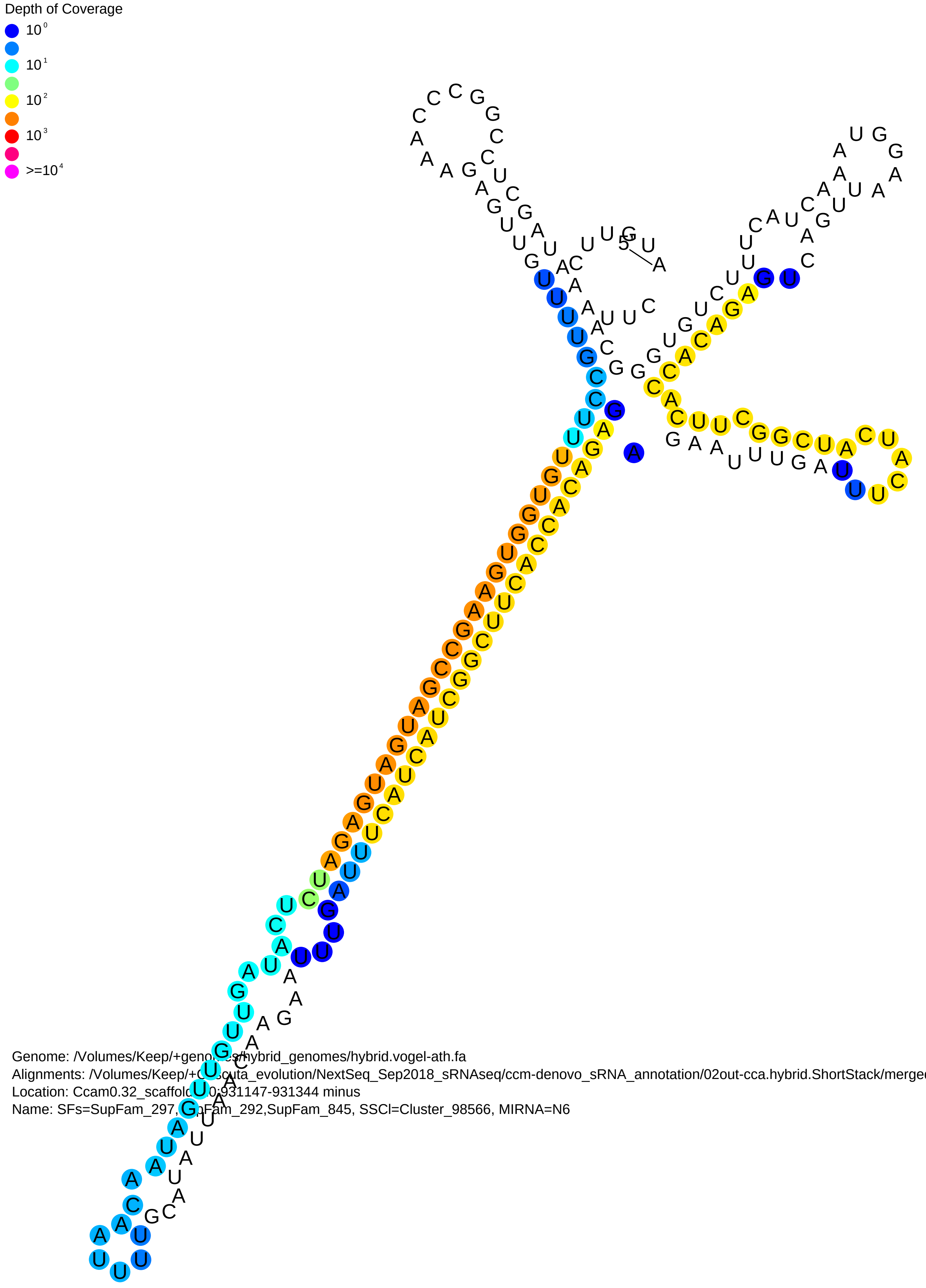

Genome: /Volumes/Keep/+geno[illegible]s/hybrid_genomes/hybrid.vogel-ath.fa
Alignments: /Volumes/Keep/+C[illegible]scuta_evolution/NextSeq_Sep2018_sRNAseq/ccm-denovo_sRNA_annotation/02out-cca.hybrid.ShortStack/merged_alig
Location: Ccam0.32_scaffold[illegible]0:931147-931344 minus
Name: SFs=SupFam_297,[illegible]pFam_292,SupFam_845, SSCl=Cluster_98566, MIRNA=N6

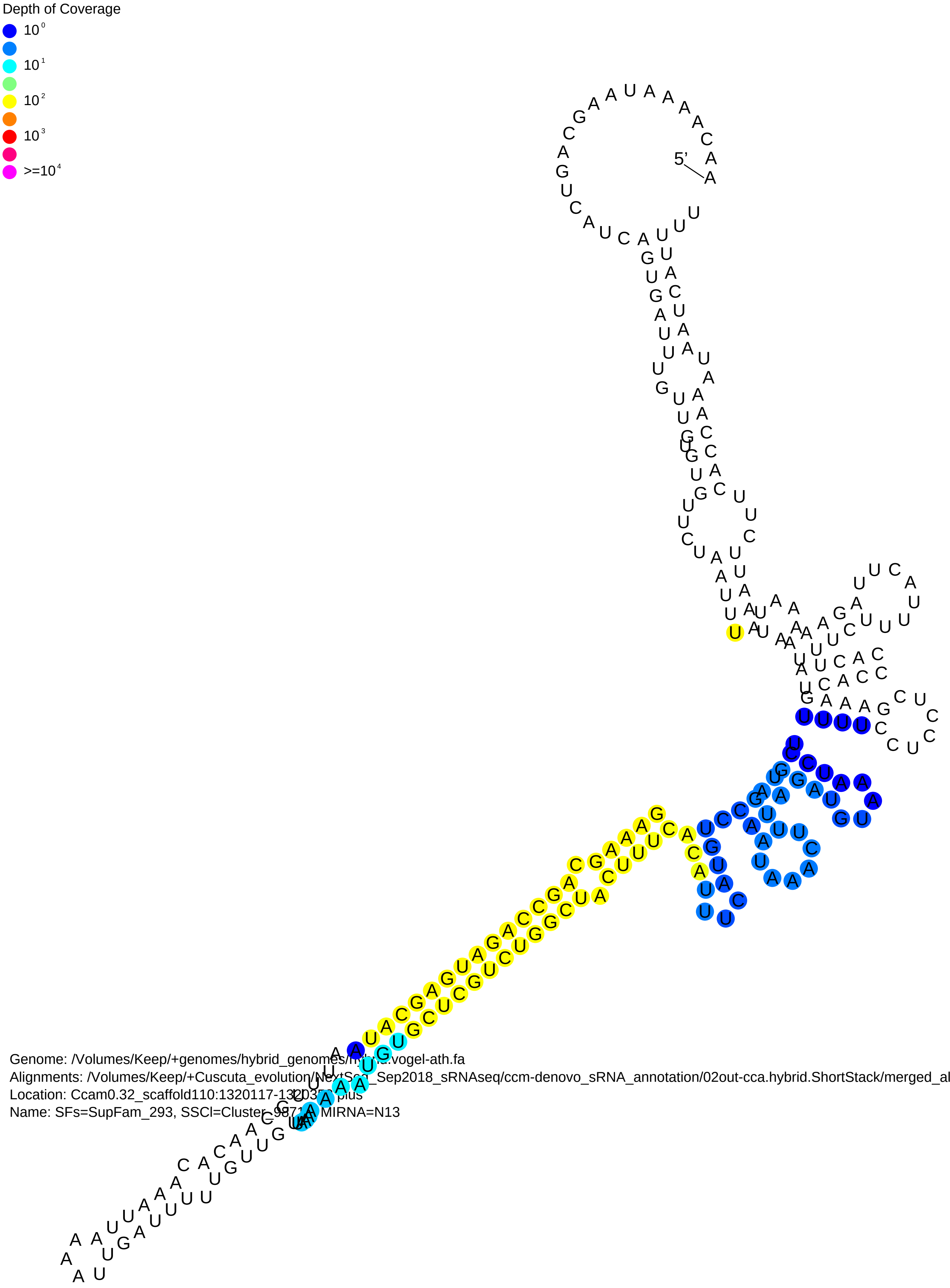

Genome: /Volumes/Keep/+genomes/hybrid_genomes/[illegible].vogel-ath.fa
Alignments: /Volumes/Keep/+Cuscuta_evolution/[illegible]_Sep2018_sRNAseq/ccm-denovo_sRNA_annotation/02out-cca.hybrid.ShortStack/merged_alig
Location: Ccam0.32_scaffold110:1320117-13[illegible] plus
Name: SFs=SupFam_293, SSCl=Cluster_[illegible], MIRNA=N13

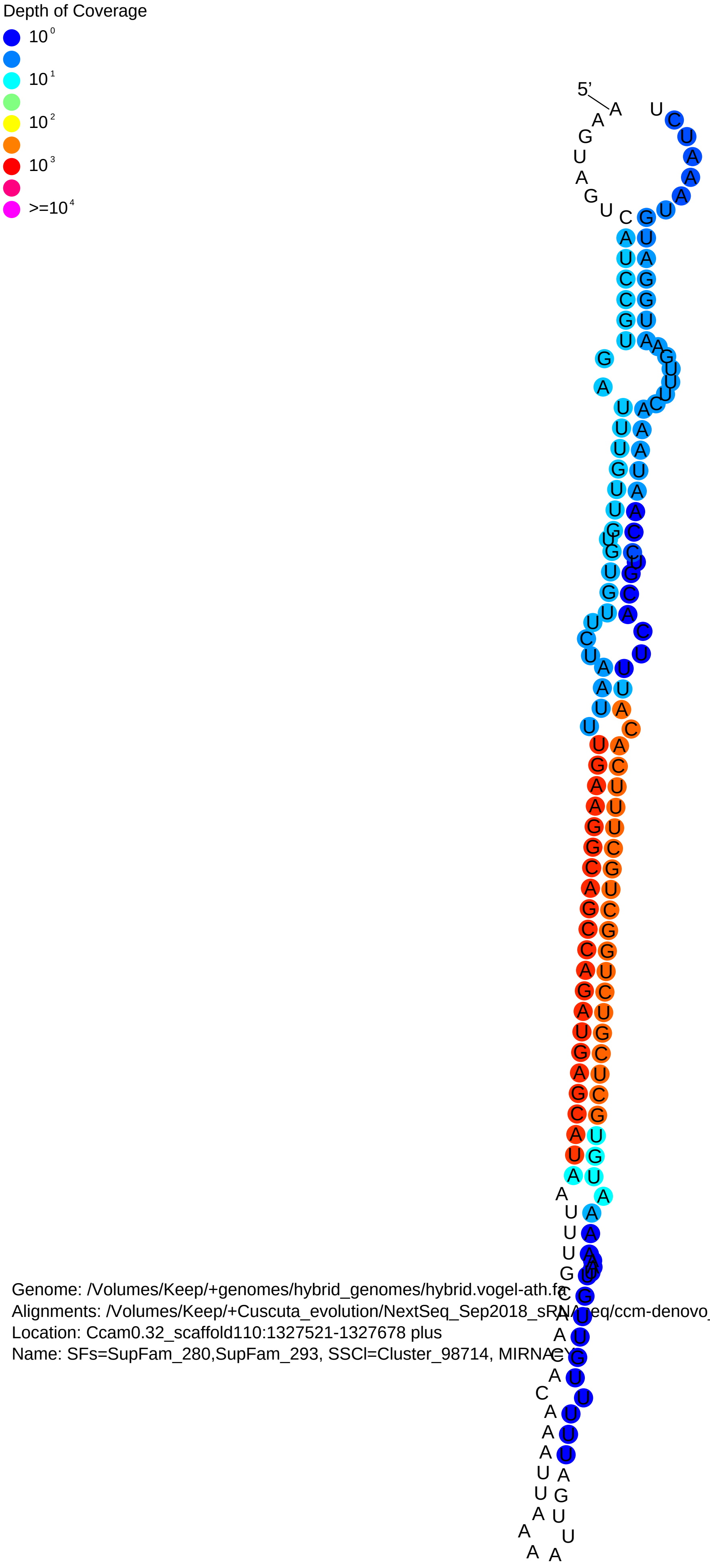

Genome: /Volumes/Keep/+genomes/hybrid_genomes/hybrid.vogel-ath.fa
Alignments: /Volumes/Keep/+Cuscuta_evolution/NextSeq_Sep2018_sRNAseq/ccm-denovo_sRNA_annotation/02out-cca.hybrid.ShortStack/merged_alig
Location: Ccam0.32_scaffold110:1327521-1327678 plus
Name: SFs=SupFam_280,SupFam_293, SSCl=Cluster_98714, MIRNA[illegible]

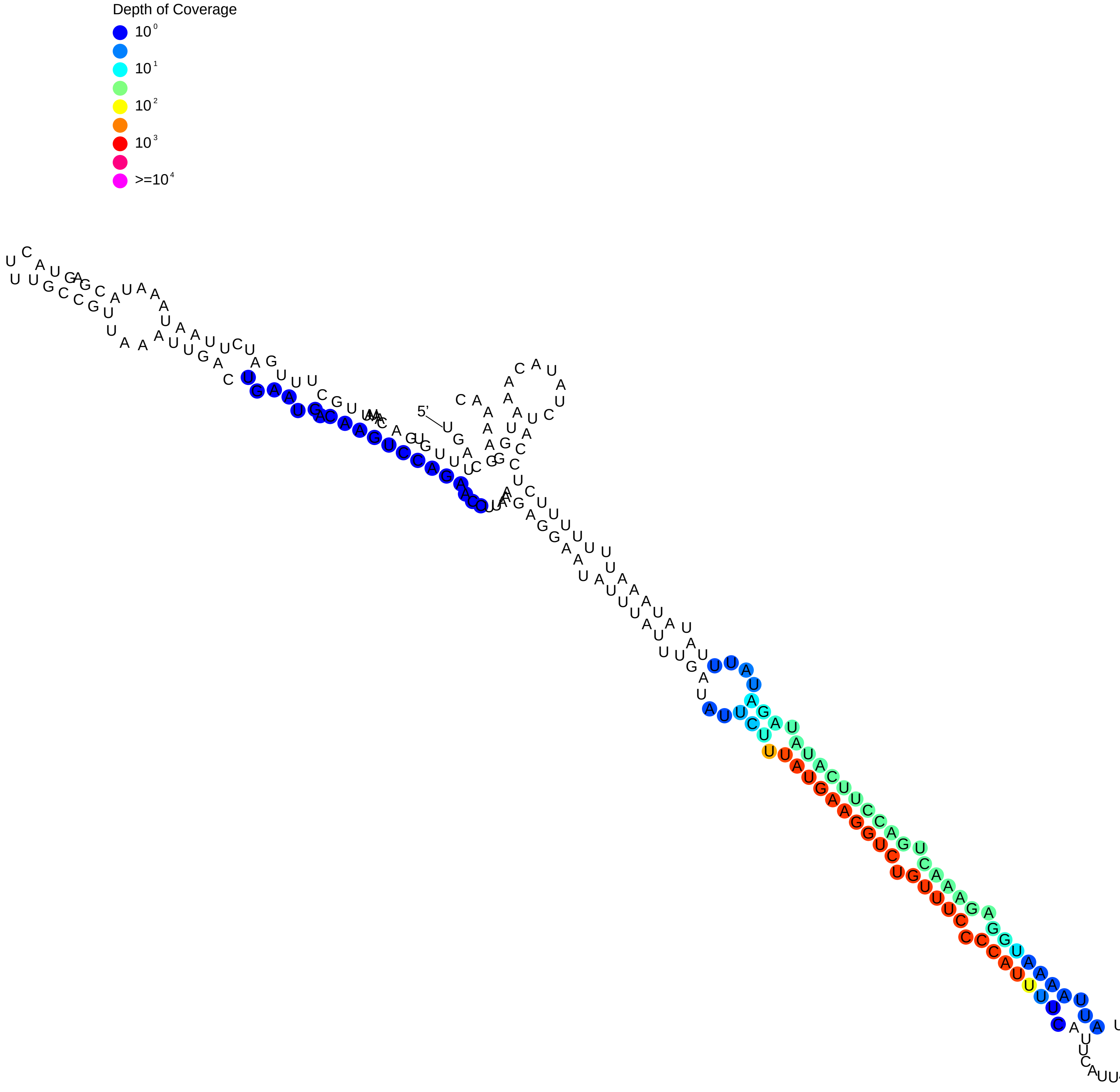

Genome: /Volumes/Keep/+genomes/hybrid_genomes/hybrid.vogel-ath.fa
Alignments: /Volumes/Keep/+Cuscuta_evolution/NextSeq_Sep2018_sRNAseq/ccm-denovo_sRNA_annotation/02out-cca.hybrid.ShortStack/merged_alig
Location: Ccam0.32_scaffold110:1394747-1394988 plus
Name: SFs=SupFam_128, SSCl=Cluster_98751, MIRNA=Y

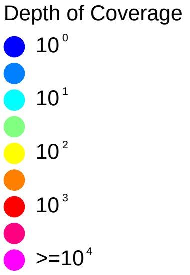

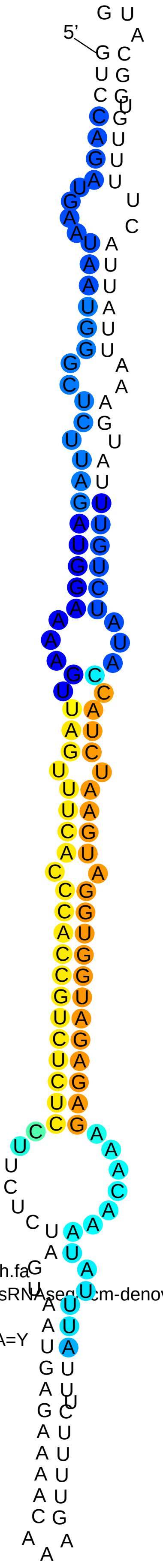

Genome: /Volumes/Keep/+genomes/hybrid_genomes/hybrid.vogel-ath.fa
Alignments: /Volumes/Keep/+Cuscuta_evolution/NextSeq_Sep2018_sRNAseq[illegible]cm-denovo_sRNA_annotation/02out-cca.hybrid.ShortStack/merged_alig
Location: Ccam0.32_scaffold110:1580746-1580905 plus
Name: SFs=SupFam_135,SupFam_54, SSCl=Cluster_98838, MIRNA=Y

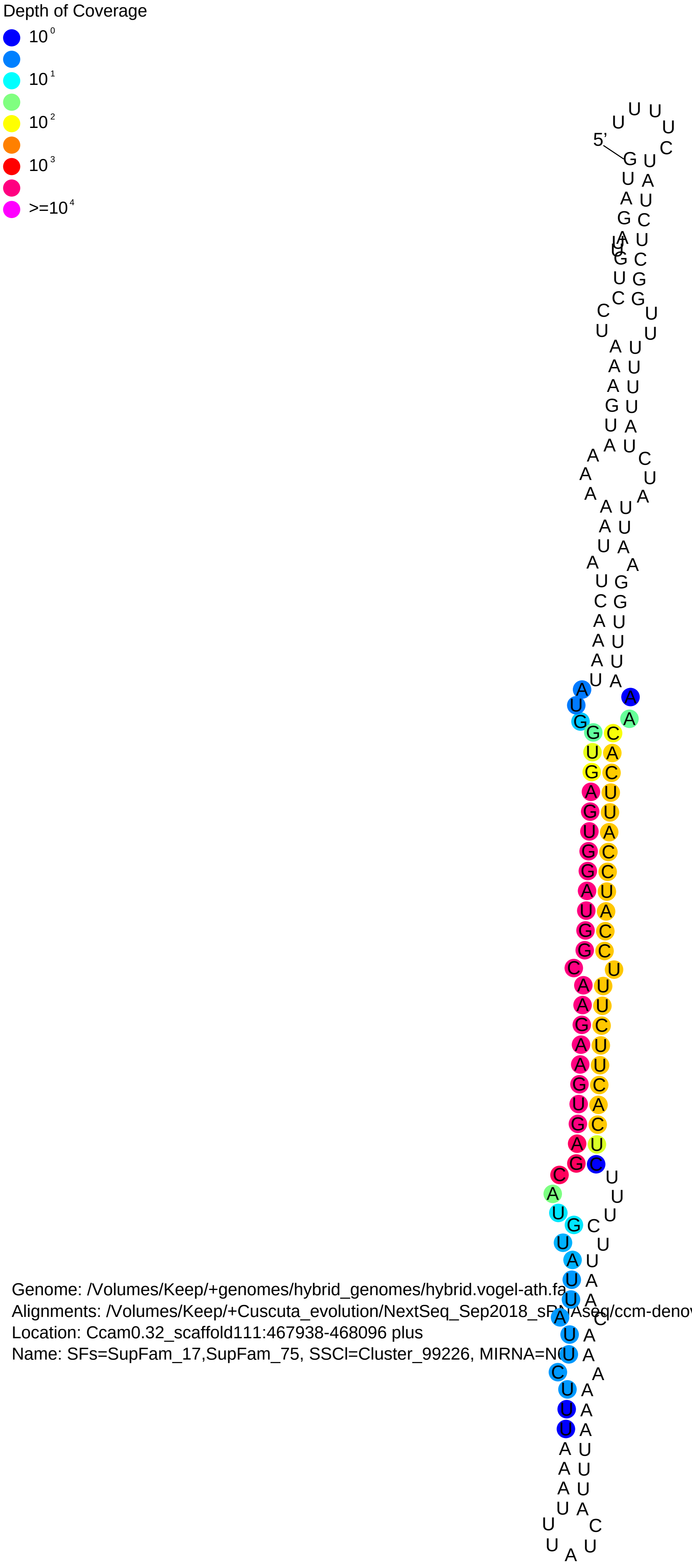

Genome: /Volumes/Keep/+genomes/hybrid_genomes/hybrid.vogel-ath.fa
Alignments: /Volumes/Keep/+Cuscuta_evolution/NextSeq_Sep2018_sRNAseq/ccm-denovo_sRNA_annotation/02out-cca.hybrid.ShortStack/merged_alig
Location: Ccam0.32_scaffold111:467938-468096 plus
Name: SFs=SupFam_17,SupFam_75, SSCl=Cluster_99226, MIRNA=N[illegible]

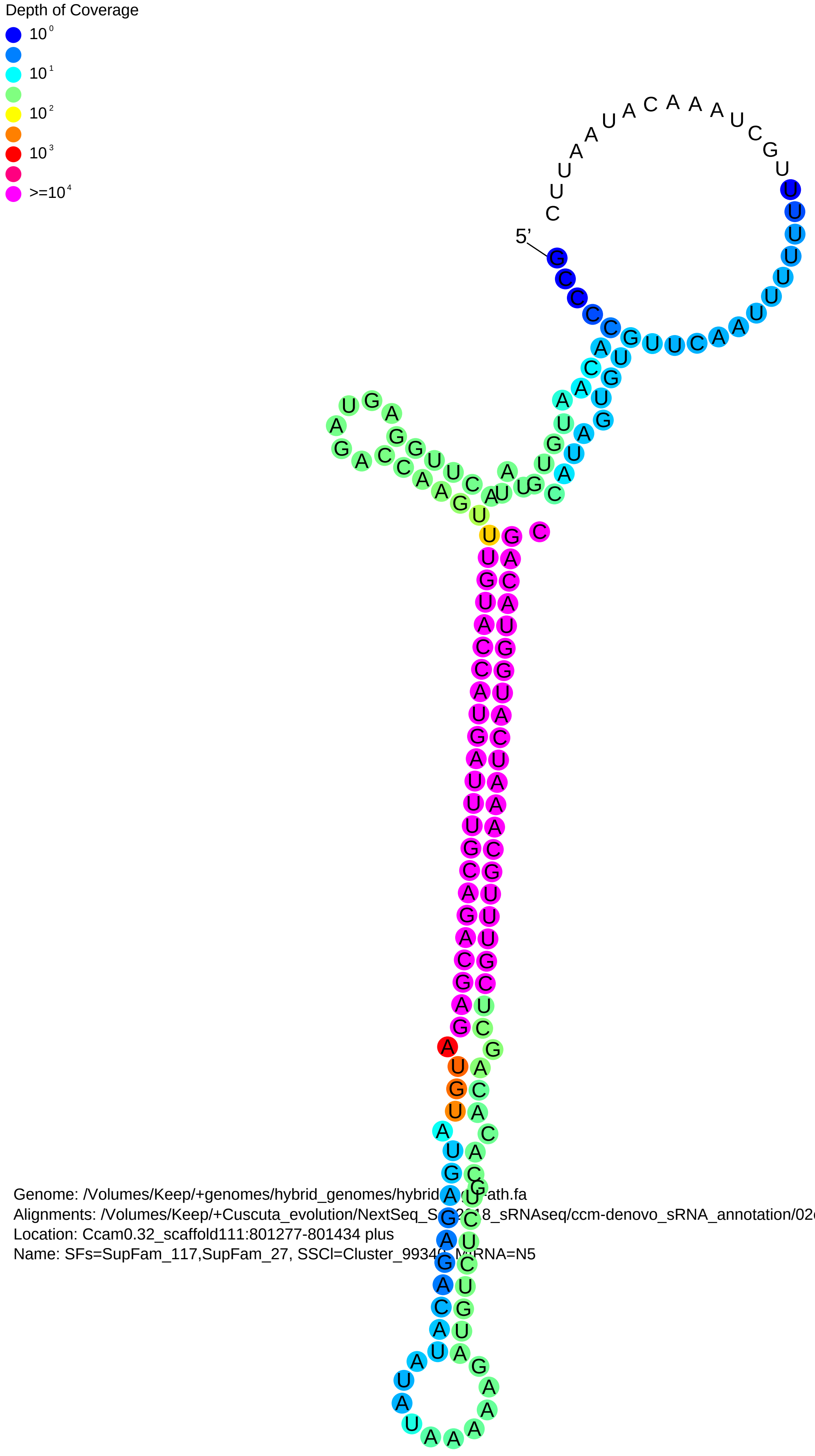

Genome: /Volumes/Keep/+genomes/hybrid_genomes/hybrid[illegible]ath.fa
Alignments: /Volumes/Keep/+Cuscuta_evolution/NextSeq_S[illegible]8_sRNAseq/ccm-denovo_sRNA_annotation/02out-cca.hybrid.ShortStack/merged_alig
Location: Ccam0.32_scaffold111:801277-801434 plus
Name: SFs=SupFam_117,SupFam_27, SSCl=Cluster_9934[illegible]RNA=N5

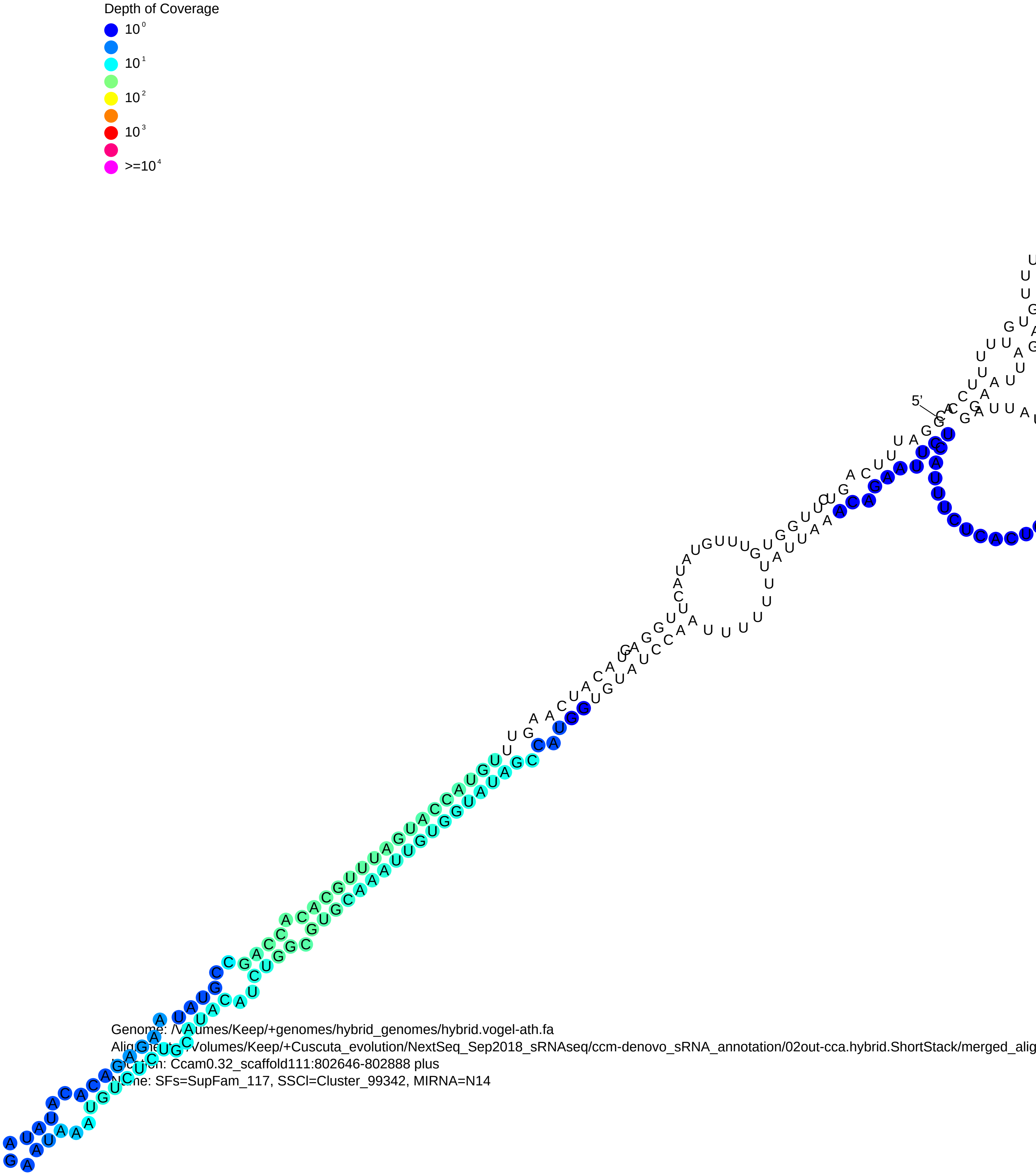

Genome: /Volumes/Keep/+genomes/hybrid_genomes/hybrid.vogel-ath.fa
Alignments: /Volumes/Keep/+Cuscuta_evolution/NextSeq_Sep2018_sRNAseq/ccm-denovo_sRNA_annotation/02out-cca.hybrid.ShortStack/merged_alig
Location: Ccam0.32_scaffold111:802646-802888 plus
Name: SFs=SupFam_117, SSCl=Cluster_99342, MIRNA=N14
