# Supplementary material for: Compensatory sequence variation between trans-species small RNAs and their target sites
Source: eLife. 2019 Dec 17;8:e49750. doi: 10.7554/eLife.49750 (PMC6917502; doi:10.7554/eLife.49750)
Supplement: Supplementary file 6. — Details of confirmed HI-sRNA targets including HI-sRNA-target complementarity, site, score, superfamily and the status of C. campestris superfamily members as a confirmed miRNA. Targeting confirmation for target mRNA is shown in upper right, with confirmed interactions in species highlighted in red. sRNA distribution at target locus is shown for experimental interface and control, demonstrating secondary siRNA phasing and size distribution for up-regulated loci. Degradome sequencing is shown where confirmed hits were discovered in NanoPARE data. Format: PDF [file elife-49750-supp6.pdf]

Allenscore: 2

Target Interaction:

```
5'- AUUGGAUUGAAGCUAUUGAUCCU   AT3G52870.1
     | | | | |   | | | | | | | | | | | | | | | |
3'- UAACCGAACUUCGAUAACUAGGU   CI_cpe-2015_676
```

Target Site: 559

Superfamily: SupFam_188

miRNAin ccm: N/A

Published name: N/A

Confirmed Targeting

2nd-siRNA	*NanoPARE*
ccm	*ccm*
cpe-2015	*cpe-2015*
cpe-2017	
cgr-dp	*cgr-dp*
cgr-pm	
cgr-mass	
cin	*cin*

IQ_calmodulin-binding_motif_family_prote

AT3G52870 - CI_cpe-2015_676

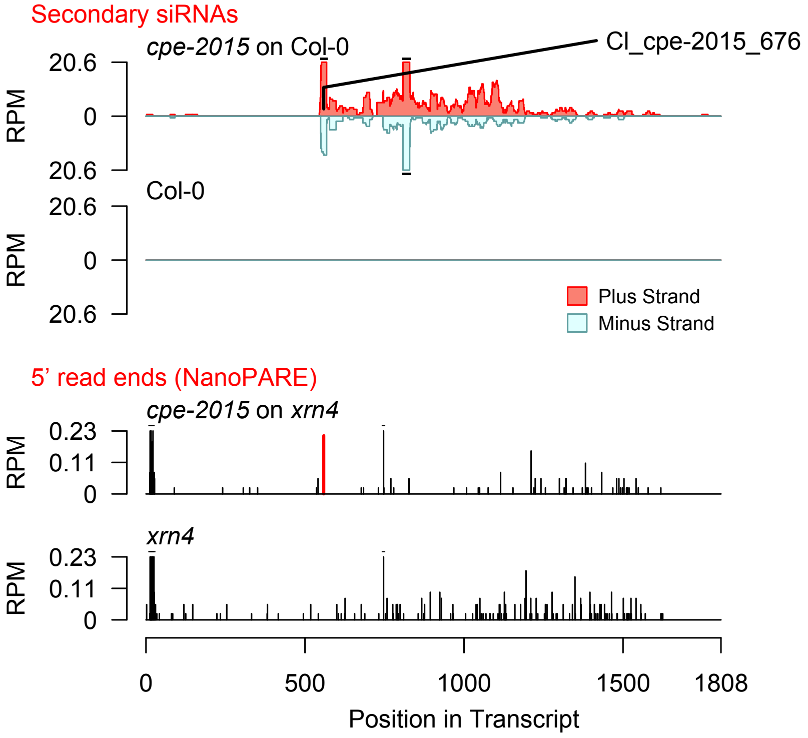

2nd siRNAs: Phase diagram

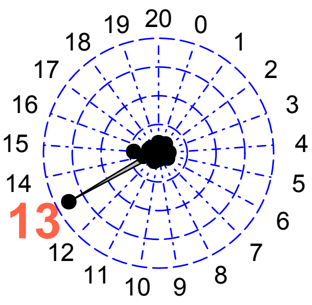

2nd siRNAs: Size distribution

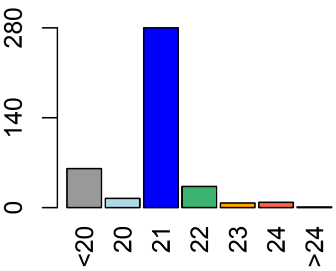

Allenscore: 2

Target Interaction:

```
5'- GGAUUGAAGCUAUUGAUCCU   AT3G52870.1
     | |   | | | | | | | | | | | | | | | |
3'- CCGAACUUCGAUAACUAGGU   CI_cpe-2015_4190
```

Target Site: 559

Superfamily: SupFam_188

miRNAin ccm: N/A

Published name: N/A

Confirmed Targeting

2nd-siRNA	*NanoPARE*
ccm	*ccm*
cpe-2015	*cpe-2015*
cpe-2017	
cgr-dp	*cgr-dp*
cgr-pm	
cgr-mass	
cin	*cin*

IQ_calmodulin-binding_motif_family_prote

AT3G52870 - CI_cpe-2015_4190

Secondary siRNAs

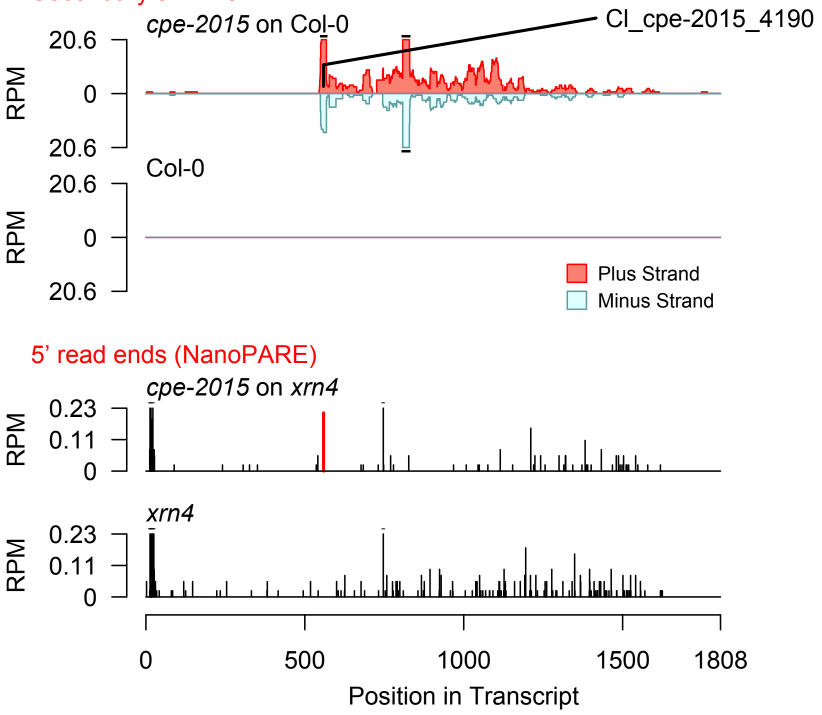

2nd siRNAs: Phase diagram

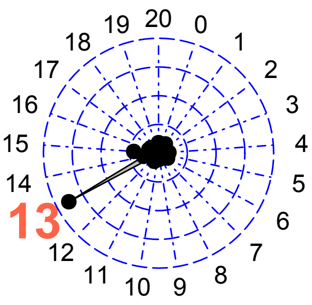

2nd siRNAs: Size distribution

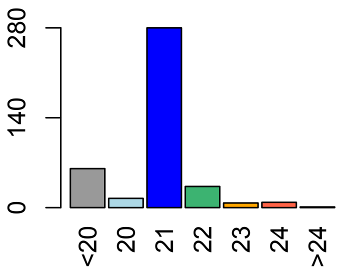

Allenscore: 2

Target Interaction:

```
5'- AUUGGAUUGAAGCUAUUGAUCCU    AT3G52870.1
     |||||  ||||||||||||||||
3'- UAACCGAACUUCGAUAACUAGGU    CI_cpe-2017_757
```

Target Site: 559

Superfamily: SupFam_188

miRNAin ccm: N/A

Published name: N/A

Confirmed Targeting

2nd-siRNA	NanoPARE
ccm	*ccm*
cpe-2015	*cpe-2015*
cpe-2017	
cgr-dp	*cgr-dp*
cgr-pm	
cgr-mass	
cin	*cin*

IQ_calmodulin-binding_motif_family_prote

AT3G52870 - CI_cpe-2017_757

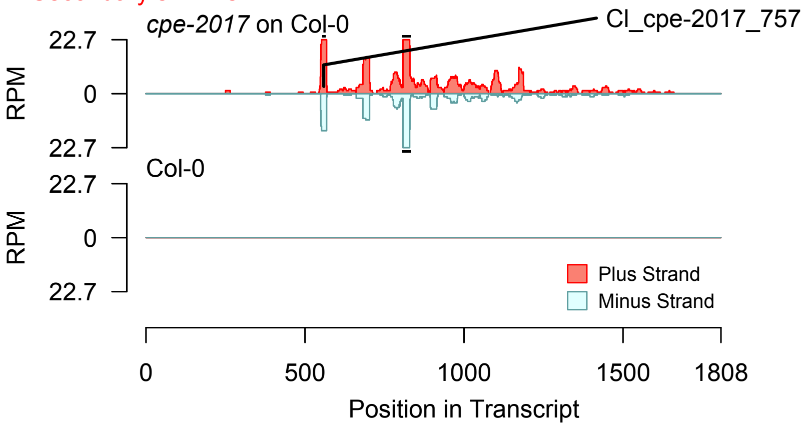

Degradome hits not found for sRNA

2nd siRNAs: Phase diagram

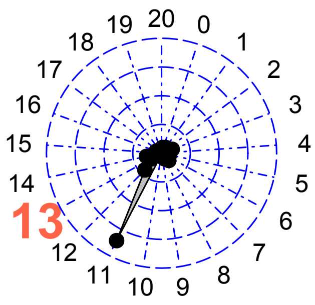

2nd siRNAs: Size distribution

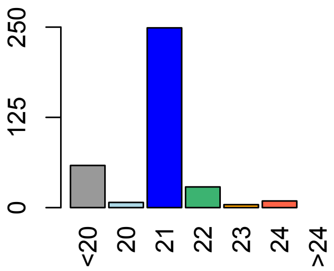

Allenscore: 2

Target Interaction:

```
5'- GGAUUGAAGCUAUUGAUCCU   AT3G52870.1
    || ||||||||||||||||
3'- CCGAACUUCGAUAACUAGGU   CI_cpe-2017_3761
```

Target Site: 559

Superfamily: SupFam_188

miRNAin ccm: N/A

Published name: N/A

Confirmed Targeting

2nd-siRNA	*NanoPARE*
ccm	*ccm*
cpe-2015	*cpe-2015*
cpe-2017	
cgr-dp	*cgr-dp*
cgr-pm	
cgr-mass	
cin	*cin*

IQ_calmodulin-binding_motif_family_prote

AT3G52870 - CI_cpe-2017_3761

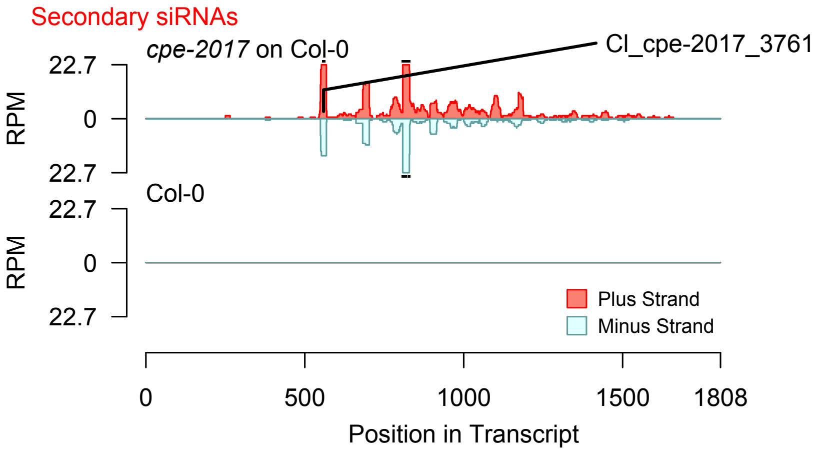

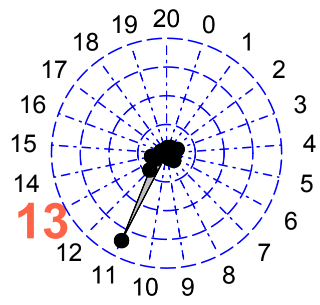

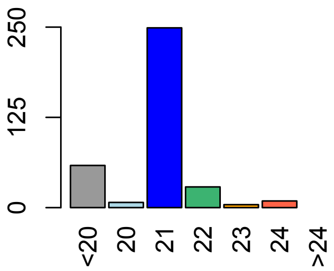

Degradome hits not found for sRNA

Allenscore: 2

Target Interaction:

```
5'- CUCCUGAGCAUUCUGCUGCUG   AT4G00830.1
      ||||||  |||||||||||||
3'- AAGGACUAGUAAGACGACGAC   Cl_cgr-dp_538
```

Target Site: 1081

Superfamily: SupFam_490

miRNAin ccm: N/A

Published name: N/A

Confirmed Targeting

2nd-siRNA	*NanoPARE*
ccm	*ccm*
cpe-2015	*cpe-2015*
cpe-2017	
cgr-dp	*cgr-dp*
cgr-pm	
cgr-mass	
cin	*cin*

LIF2

AT4G00830 - Cl_cgr-dp_538

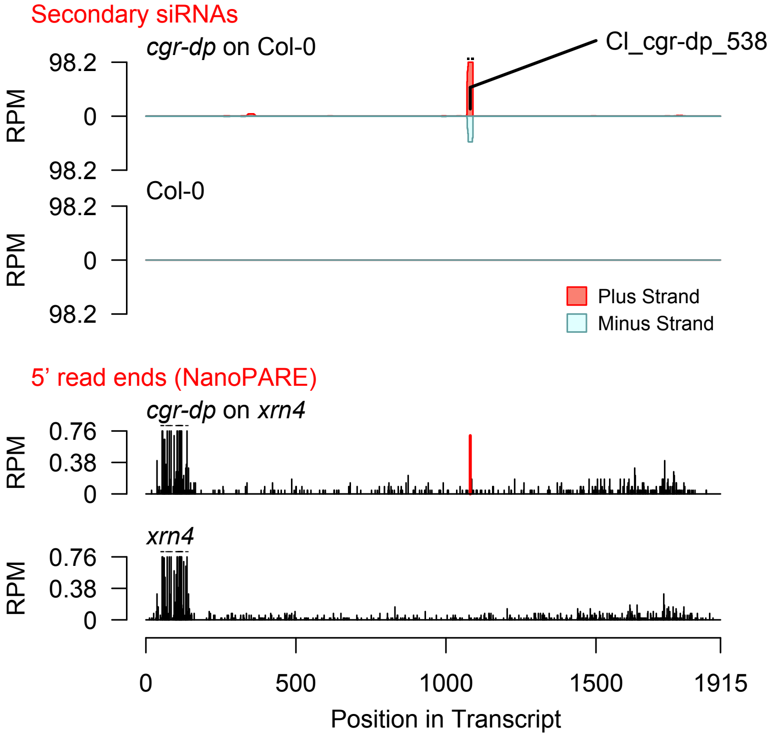

Diff. Exp. secondary siRNA locus not found

Allenscore: 3

Target Interaction:

```
5'- AUGGAUGUCAUCUGCCAAGCA   AT5G11170.1
    |||||||||||||::||||||
3'- UACCUACAGUAGGUGGUUCGA   Cl_cin_42282
```

Target Site: 382

Superfamily: SupFam_1585

miRNAin ccm: N/A

Published name: N/A

Confirmed Targeting

2nd-siRNA	*NanoPARE*
ccm	*ccm*
cpe-2015	*cpe-2015*
cpe-2017	
cgr-dp	*cgr-dp*
cgr-pm	
cgr-mass	
cin	*cin*

UAP56a

AT5G11170 - Cl_cin_42282

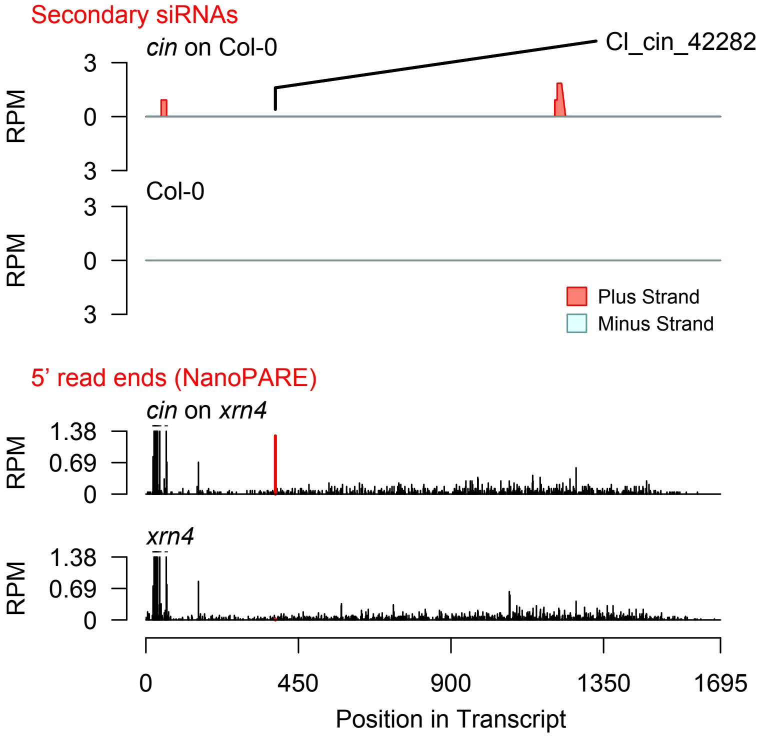

Diff. Exp. secondary siRNA locus not found

Allenscore: 4

Target Interaction:

```
5'- GUGUGCGAUGAAAGAAGUUGAG   AT5G66850.1
    | | | | | | : | | : | | | | | | |   | | | :
3'- CACACGUUAUUUUCUUC - ACUU   Cl_cgr-dp_5622
```

Target Site: 1391

Superfamily: SupFam_157

miRNAin ccm: N/A

Published name: N/A

Confirmed Targeting

2nd-siRNA	*NanoPARE*
ccm	*ccm*
cpe-2015	*cpe-2015*
cpe-2017	
cgr-dp	*cgr-dp*
cgr-pm	
cgr-mass	
cin	*cin*

MAPKKK5

AT5G66850 - Cl_cgr-dp_5622

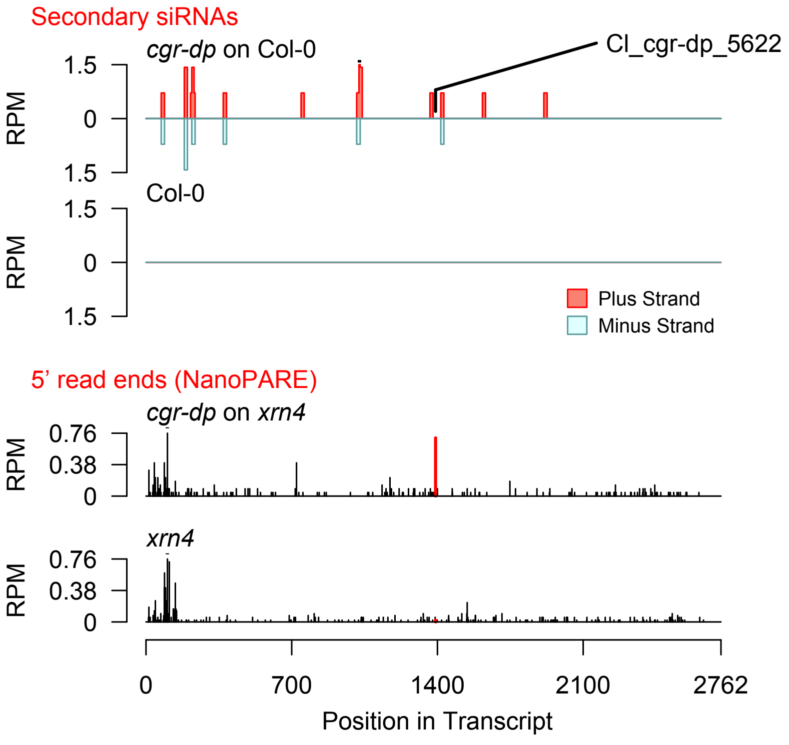

Diff. Exp. secondary siRNA locus not found

Allenscore: 1.5

Target Interaction:

```
5'- GUGUGCGAUGAAAGAAGUUGA   AT5G66850.1
     : | | : | | : | | | | | | | | | | | | | |
3'- UACGCGUUACUUUCUUCAACU   Cl_cgr-dp_866
```

Target Site: 1391

Superfamily: SupFam_157

miRNAin ccm: N/A

Published name: N/A

Confirmed Targeting

2nd-siRNA	*NanoPARE*
ccm	*ccm*
cpe-2015	*cpe-2015*
cpe-2017	
cgr-dp	*cgr-dp*
cgr-pm	
cgr-mass	
cin	*cin*

MAPKKK5

AT5G66850 - Cl_cgr-dp_866

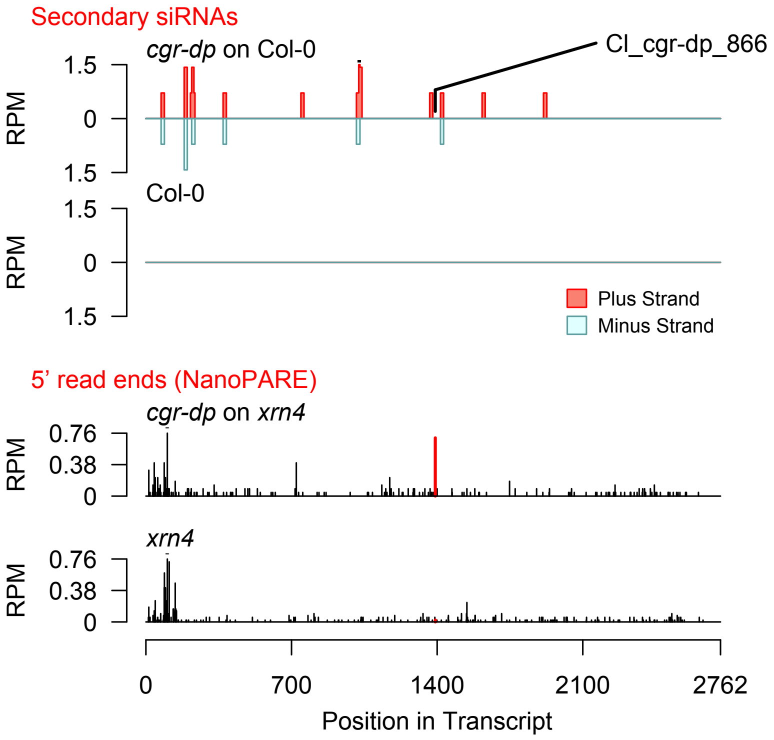

Diff. Exp. secondary siRNA locus not found

Allenscore: 5.5

Target Interaction:

```
5'- GAGAUGUUGUUCCACCACCA   AT3G45640.1
     : | | | | | | | : | | | |   | |   | | |
3'- UUCUACAAUAAGGAGGCGGU   Cl_cgr-dp_22
```

Target Site: 659

Superfamily: SupFam_203

miRNAin ccm: N/A

Published name: N/A

Confirmed Targeting

2nd-siRNA	*NanoPARE*
ccm	*ccm*
cpe-2015	*cpe-2015*
cpe-2017	
cgr-dp	*cgr-dp*
cgr-pm	
cgr-mass	
cin	*cin*

MPK3

AT3G45640 - Cl_cgr-dp_22

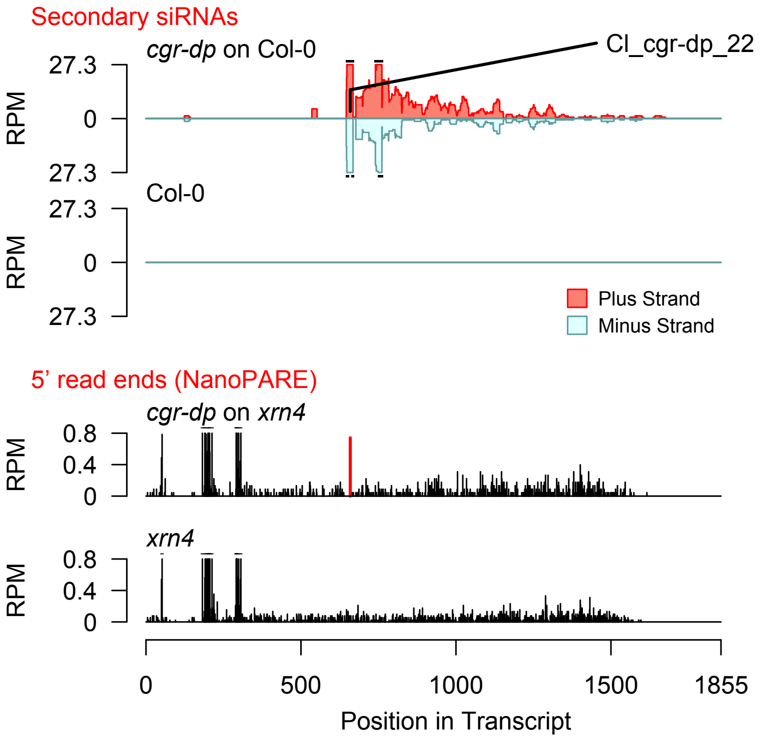

2nd siRNAs: Phase diagram

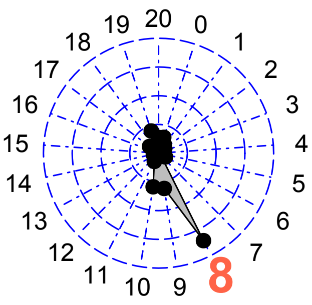

2nd siRNAs: Size distribution

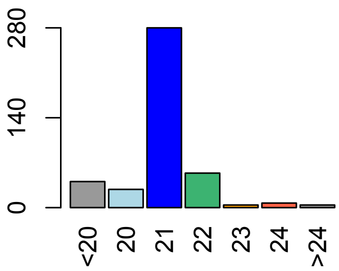

Allenscore: 5.5

Target Interaction:

```
5'- GAUGCUAAGCGUACGCUUCGUGA    AT3G45640.1
     | | | | | : | | | | |   | | : | | | | |   | |
3'- CUACGGUUCGCCUGUGAAGC - CU    CI_cin_3787
```

Target Site: 591

Superfamily: SupFam_1385

miRNAin ccm: N/A

Published name: N/A

Confirmed Targeting

2nd-siRNA	*NanoPARE*
ccm	*ccm*
cpe-2015	*cpe-2015*
cpe-2017	
cgr-dp	*cgr-dp*
cgr-pm	
cgr-mass	
cin	*cin*

MPK3

AT3G45640 - CI_cin_3787

Secondary siRNAs

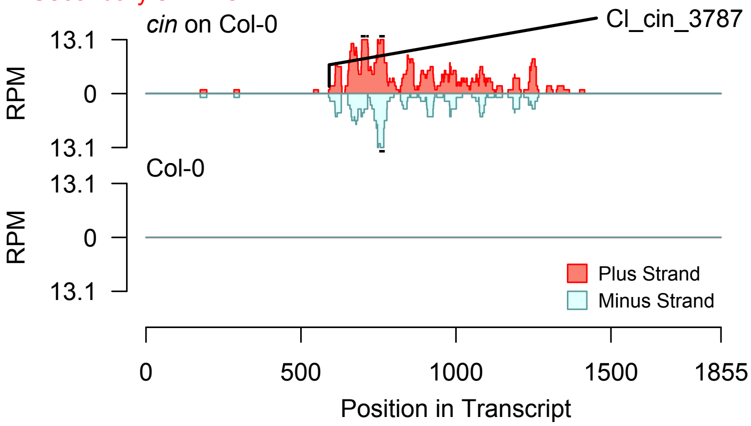

2nd siRNAs: Phase diagram

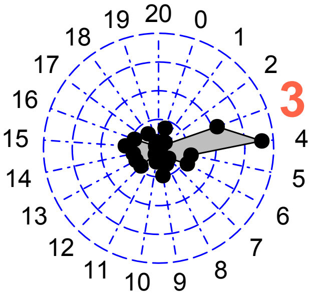

2nd siRNAs: Size distribution

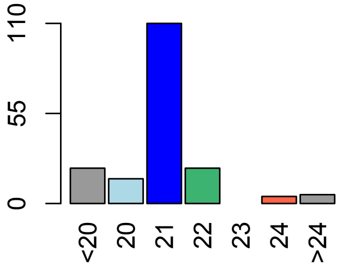

Degradome hits not found for sRNA

Allenscore: 6

Target Interaction:

```
5'- GCUCUUGGAAGAAGAUGAAAGGCUCC   AT4G02400.1
     : | : |      | | | | | | | | | | | | | : | | | |
3'- UGGG - - - CUUCUUCUACUUUUCGAGU   Cl_cgr-dp_279
```

Target Site: 1850

Superfamily: SupFam_37

miRNAin ccm: N/A

Published name: N/A

Confirmed Targeting

2nd-siRNA	*NanoPARE*
ccm	*ccm*
cpe-2015	*cpe-2015*
cpe-2017	
cgr-dp	*cgr-dp*
cgr-pm	
cgr-mass	
cin	*cin*

U3_ribonucleoprotein_family_protein

AT4G02400 - Cl_cgr-dp_279

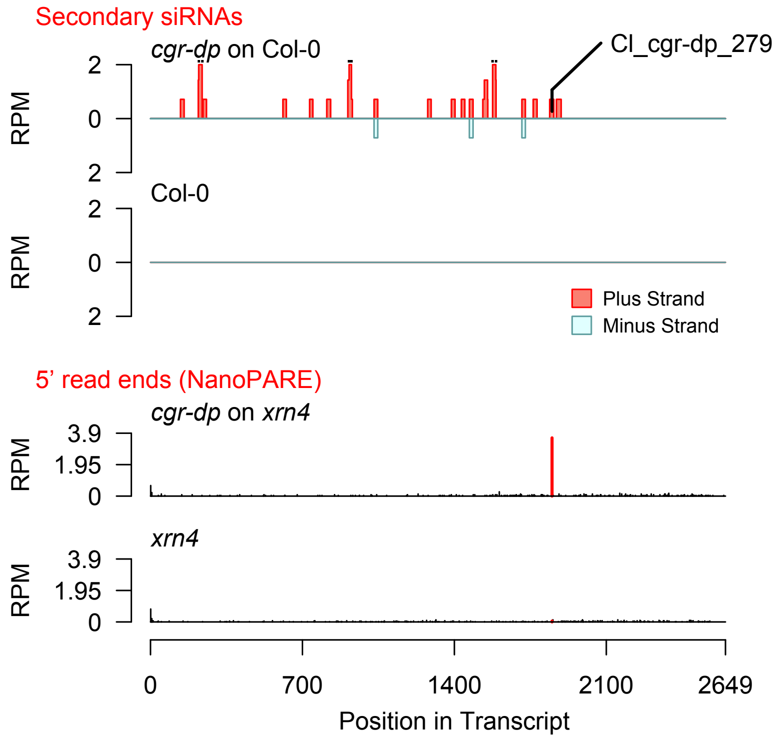

Diff. Exp. secondary siRNA locus not found

Allenscore: 3

Target Interaction:

```
5'- GGAAGAAGAUGAAAGGCUCC   AT4G02400.1
      | | | | | | | | | | | | | : | | | |
3'- GCUUCUUCUACUUUUCGAGU   CI_cgr-dp_37384
```

Target Site: 1850

Superfamily: SupFam_37

miRNAin ccm: N/A

Published name: N/A

Confirmed Targeting

2nd-siRNA	*NanoPARE*
ccm	*ccm*
cpe-2015	*cpe-2015*
cpe-2017	
cgr-dp	*cgr-dp*
cgr-pm	
cgr-mass	
cin	*cin*

U3_ribonucleoprotein_family_protein

AT4G02400 - CI_cgr-dp_37384

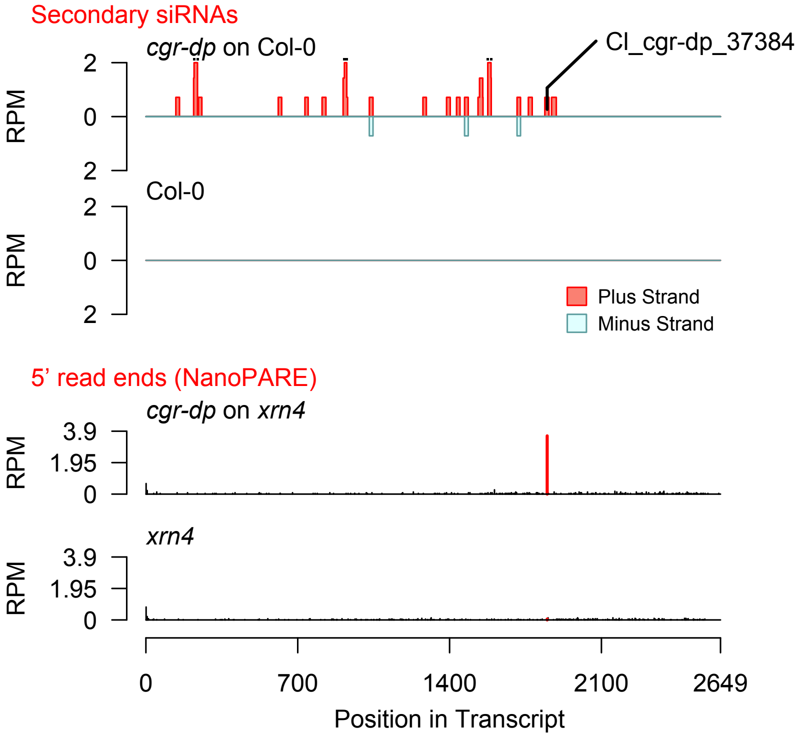

Diff. Exp. secondary siRNA locus not found

Allenscore: 3.5

Target Interaction:

```
5'- AGAGGAGGAGGAGGAAGACUA   AT3G09070.1
    | ||||||:|| ||||||||||
3'- UGUCCUCUUCAUCCUUCUGAU   Cl_cin_11140
```

Target Site: 1028

Superfamily: SupFam_1431

miRNAin ccm: N/A

Published name: N/A

Confirmed Targeting

2nd-siRNA	*NanoPARE*
ccm	*ccm*
cpe-2015	*cpe-2015*
cpe-2017	
cgr-dp	*cgr-dp*
cgr-pm	
cgr-mass	
cin	*cin*

OPS

AT3G09070 - Cl_cin_11140

Secondary siRNAs

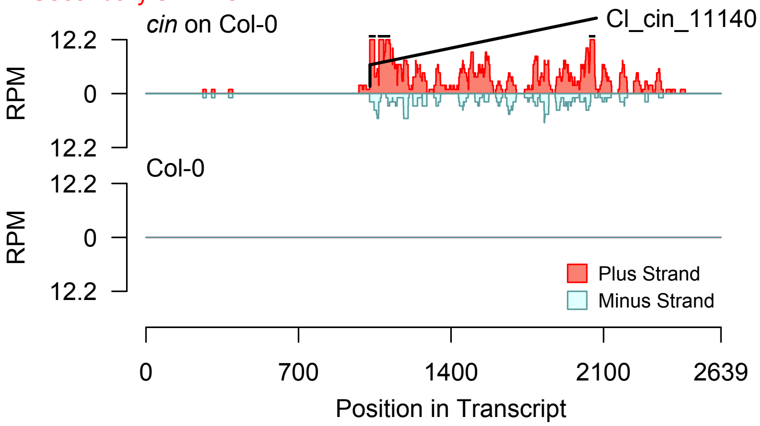

2nd siRNAs: Phase diagram

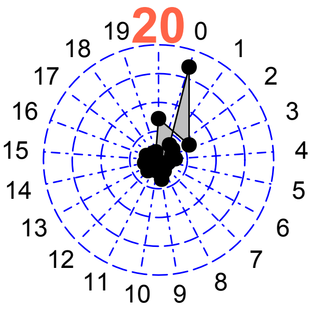

2nd siRNAs: Size distribution

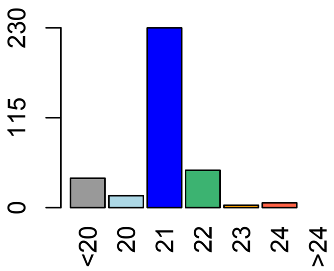

Degradome hits not found for sRNA

Allenscore: 4

Target Interaction:

```
5'- UUGGCUACUUAGCUCCAGAGUA    AT1G21590.1
     | | | |   | | | | | | | |   | | | | | | | | |
3'- AACCAAUGAAUCUAGGUCUCAC    Cl_cpe-2015_439
```

Target Site: 2056

Superfamily: SupFam_257

miRNAin ccm: N/A

Published name: N/A

Confirmed Targeting

2nd-siRNA	*NanoPARE*
ccm	*ccm*
cpe-2015	*cpe-2015*
cpe-2017	
cgr-dp	*cgr-dp*
cgr-pm	
cgr-mass	
cin	*cin*

Protein_kinase_superfamily_protein

AT1G21590 - Cl_cpe-2015_439

Secondary siRNAs

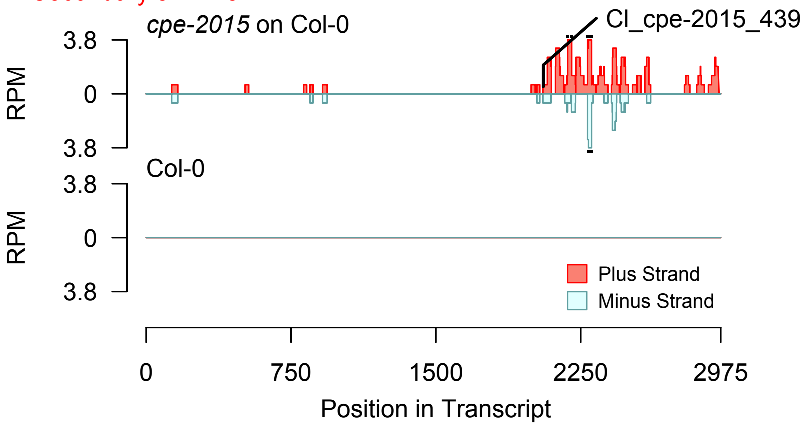

2nd siRNAs: Phase diagram

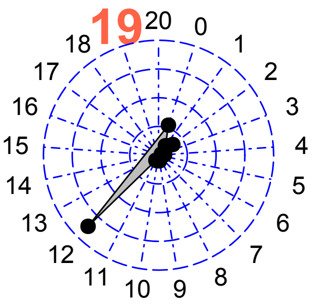

2nd siRNAs: Size distribution

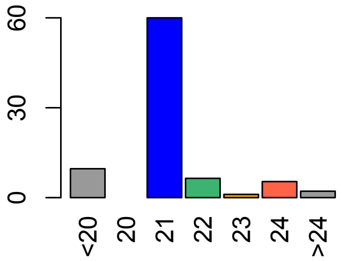

Degradome hits not found for sRNA

Allenscore: 4.5

Target Interaction:

```
5'- GGCUACUUAGCUCCAGAGUA    AT1G21590.1
     : |   | | | | | | | |   | | | | | | | | |
3'- UCAAUGAAUCUAGGUCUCAC    CI_cpe-2015_45087
```

Target Site: 2056

Superfamily: SupFam_257

miRNAin ccm: N/A

Published name: N/A

Confirmed Targeting

2nd-siRNA	*NanoPARE*
ccm	*ccm*
cpe-2015	*cpe-2015*
cpe-2017	
cgr-dp	*cgr-dp*
cgr-pm	
cgr-mass	
cin	*cin*

Protein_kinase_superfamily_protein

AT1G21590 - CI_cpe-2015_45087

2nd siRNAs: Phase diagram

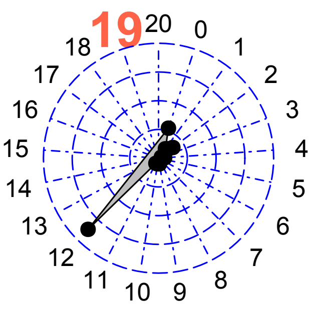

Secondary siRNAs

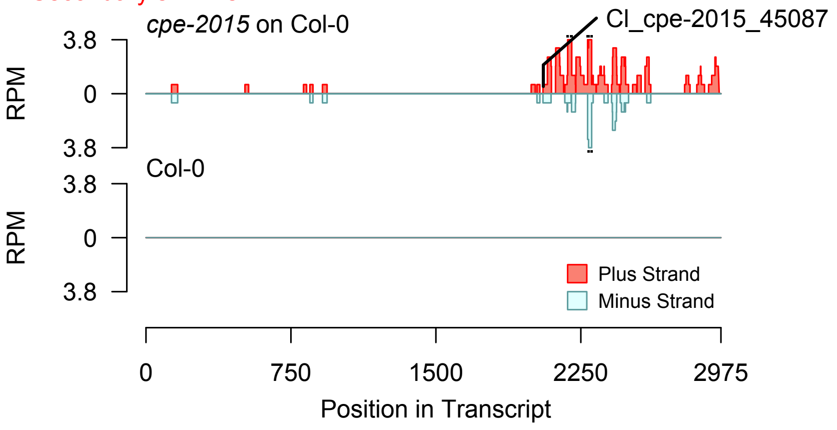

2nd siRNAs: Size distribution

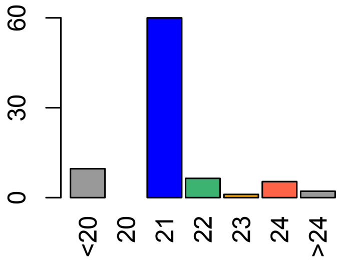

Degradome hits not found for sRNA

Allenscore: 4

Target Interaction:

```
5'- UUGGCUACUUAGCUCCAGAGUA    AT1G21590.1
     ||||  ||||||||  ||||||||
3'- AACCAAUGAAUCUAGGUCUCAC    Cl_cpe-2017_503
```

Target Site: 2056

Superfamily: SupFam_257

miRNAin ccm: N/A

Published name: N/A

Confirmed Targeting

2nd-siRNA	*NanoPARE*
ccm	*ccm*
cpe-2015	*cpe-2015*
cpe-2017	
cgr-dp	*cgr-dp*
cgr-pm	
cgr-mass	
cin	*cin*

Protein_kinase_superfamily_protein

AT1G21590 - Cl_cpe-2017_503

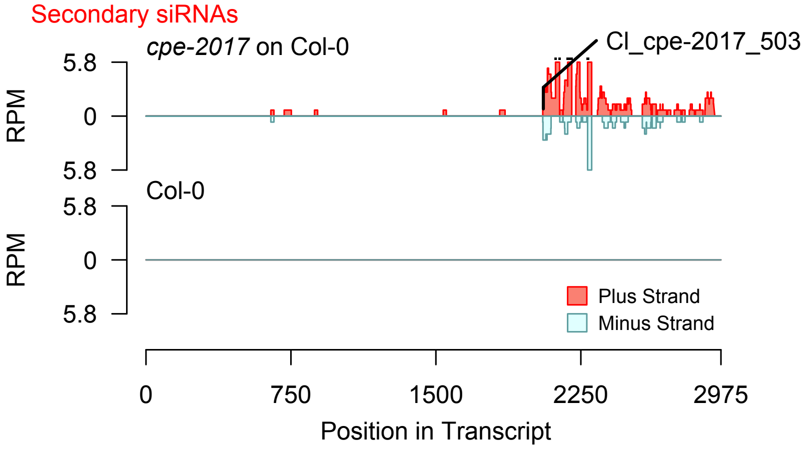

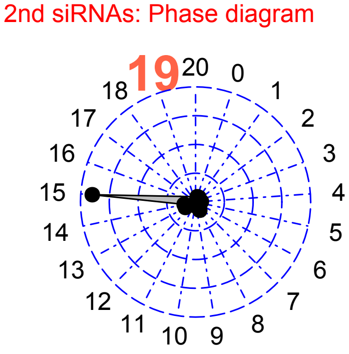

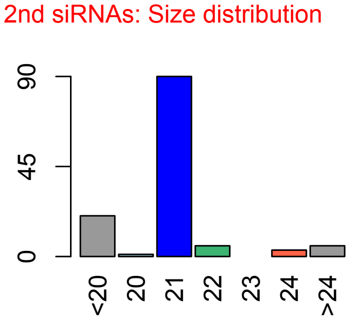

Degradome hits not found for sRNA

Allenscore: 2.5

Target Interaction:

```
5'- UAGGUUACAUGGCUCCAGAGUA   AT1G53440.1
     ||||:||||||| ||||||||||
3'- AUCCGAUGUACGGAGGUCUCAU   Cl_cgr-mass_20246
```

Target Site: 2600

Superfamily: SupFam_59

miRNAin ccm: N/A

Published name: N/A

Confirmed Targeting

2nd-siRNA	*NanoPARE*
ccm	*ccm*
cpe-2015	*cpe-2015*
cpe-2017	
cgr-dp	*cgr-dp*
cgr-pm	
cgr-mass	
cin	*cin*

Leucine-rich_repeat_transmembrane_protei

AT1G53440 - Cl_cgr-mass_20246

Secondary siRNAs

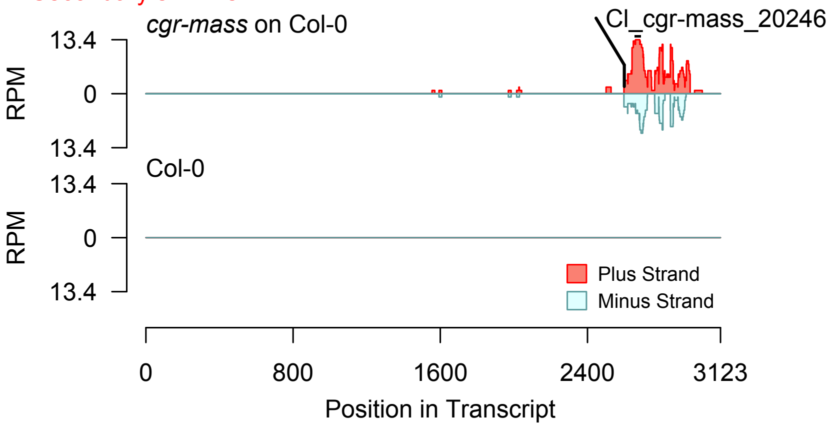

2nd siRNAs: Phase diagram

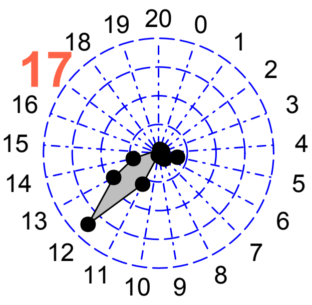

2nd siRNAs: Size distribution

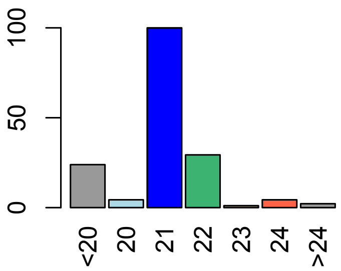

Degradome hits not found for sRNA

Allenscore: 4

Target Interaction:

```
5'- GAGAAUGGCUGGCAGAGGUGA    AT5G56460.1
      | | | | | | | |   | | | | | | : | | | |
3'- GUCUUACCGUCCGUCUUCACU    Cl_cgr-dp_8691
```

Target Site: 820

Superfamily: SupFam_4

miRNAin ccm: N/A

Published name: N/A

Confirmed Targeting

2nd-siRNA	*NanoPARE*
ccm	*ccm*
cpe-2015	*cpe-2015*
cpe-2017	
cgr-dp	*cgr-dp*
cgr-pm	
cgr-mass	
cin	*cin*

Protein_kinase_superfamily_protein

AT5G56460 - Cl_cgr-dp_8691

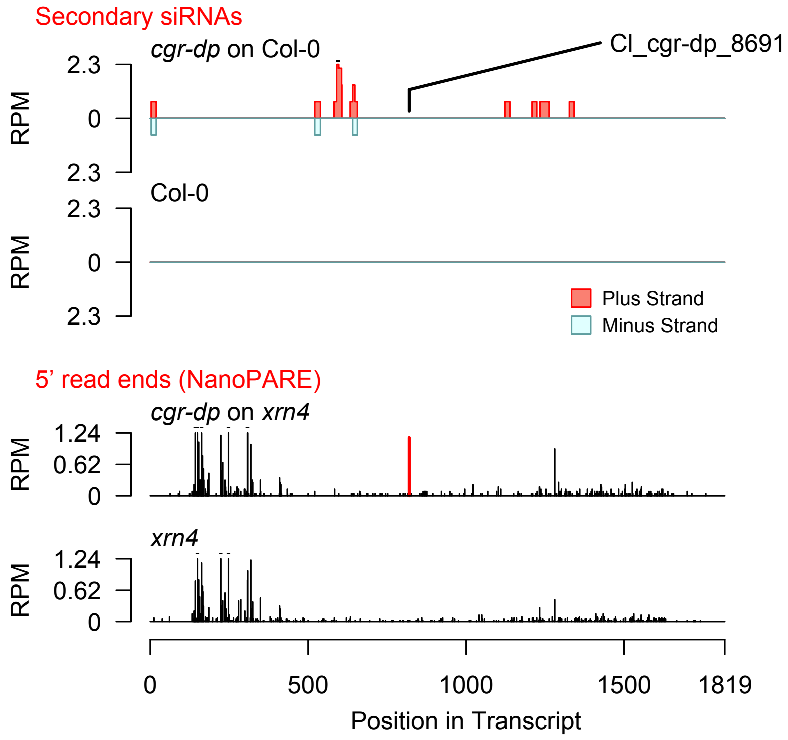

Diff. Exp. secondary siRNA locus not found

Allenscore: 5.5

Target Interaction:

```
5'- GAGAAUGGCUGGCAGAGGUGA    AT5G56460.1
     | | | | | | | : |   | |   | | | | | | | |
3'- GUCUUACUGUCCAUCUCCACU    Cl_cgr-dp_67
```

Target Site: 820

Superfamily: SupFam_4

miRNAin ccm: N/A

Published name: N/A

Confirmed Targeting

2nd-siRNA	*NanoPARE*
ccm	*ccm*
cpe-2015	*cpe-2015*
cpe-2017	
cgr-dp	*cgr-dp*
cgr-pm	
cgr-mass	
cin	*cin*

Protein_kinase_superfamily_protein

AT5G56460 - Cl_cgr-dp_67

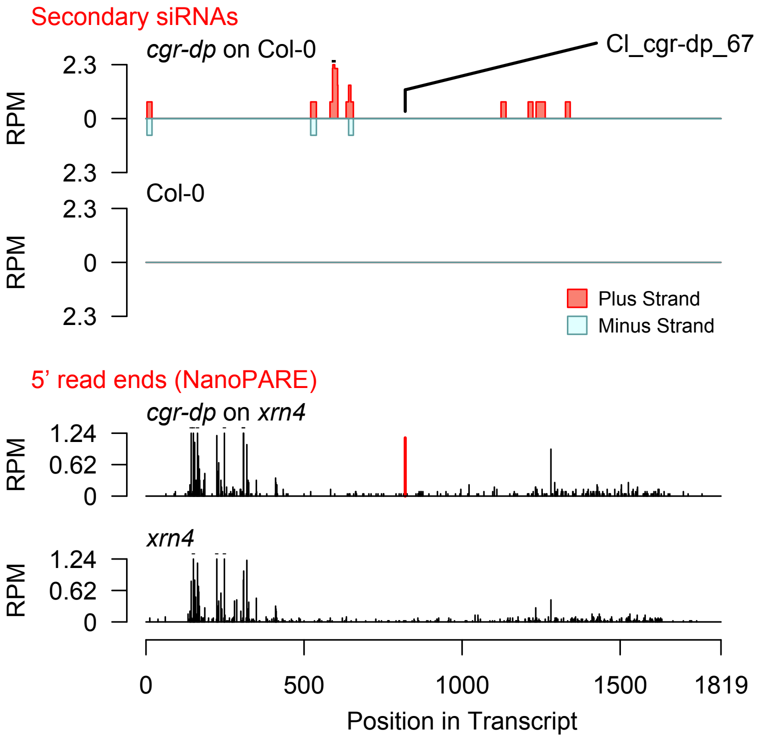

Diff. Exp. secondary siRNA locus not found

Allenscore: 5

Target Interaction:

```
5'- GAGAAUGGCUGGCAGAGGUGA    AT5G56460.1
     | | | | | | | |   | | |   | | | | | | |
3'- AUCUUACCGUCCGGCUCCACU    Cl_cgr-dp_1335
```

Target Site: 820

Superfamily: SupFam_4

miRNAin ccm: N/A

Published name: N/A

Confirmed Targeting

2nd-siRNA	*NanoPARE*
ccm	*ccm*
cpe-2015	*cpe-2015*
cpe-2017	
cgr-dp	*cgr-dp*
cgr-pm	
cgr-mass	
cin	*cin*

Protein_kinase_superfamily_protein

AT5G56460 - Cl_cgr-dp_1335

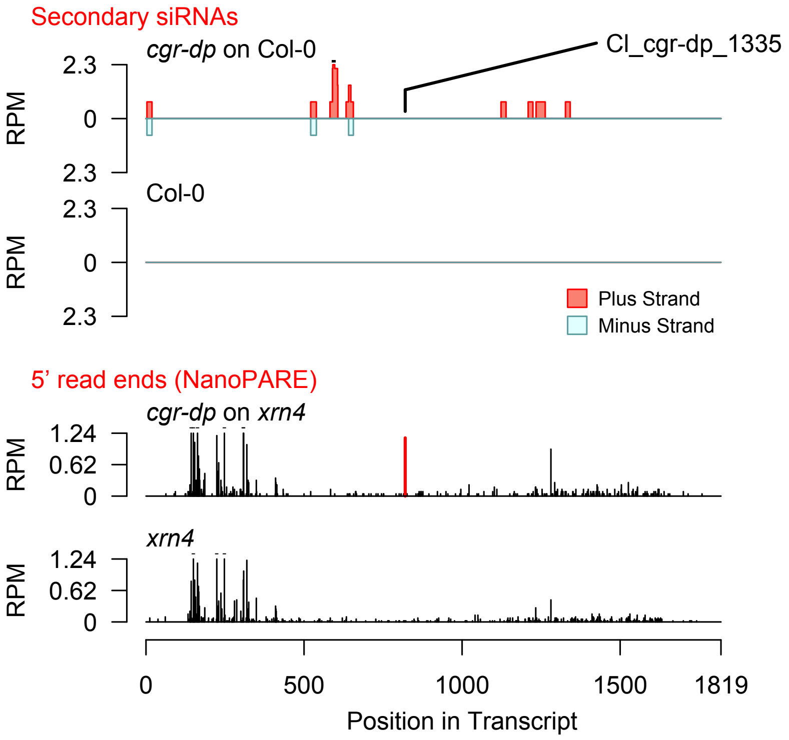

Diff. Exp. secondary siRNA locus not found

Allenscore: 3.5

Target Interaction:

```
5'- GGAGACUAGUACUAGGAGACAUAAUCA    AT3G27925.1
     | |     | | | | | | | : | | | | | | | | | | | | | |
3'- CC - - - GAUCAUGGUCCUCUGUAUUAGU    CI_cgr-dp_83
```

Target Site: 1305

Superfamily: SupFam_33

miRNAin ccm: N/A

Published name: N/A

Confirmed Targeting

2nd-siRNA	*NanoPARE*
ccm	*ccm*
cpe-2015	*cpe-2015*
cpe-2017	
cgr-dp	*cgr-dp*
cgr-pm	
cgr-mass	
cin	*cin*

DEG1

AT3G27925 - CI_cgr-dp_83

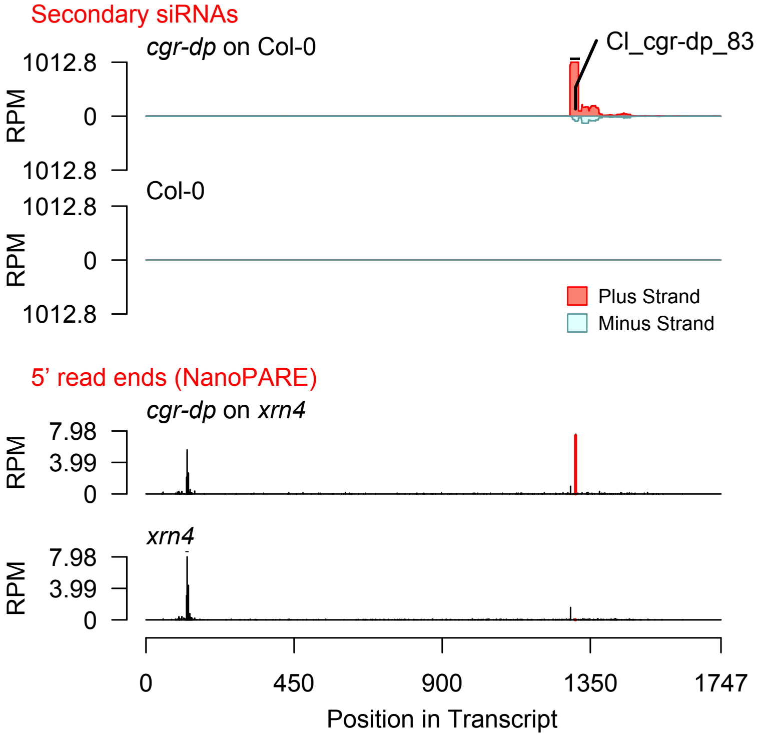

2nd siRNAs: Phase diagram

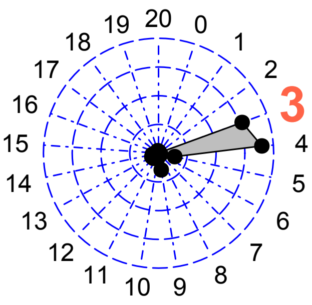

2nd siRNAs: Size distribution

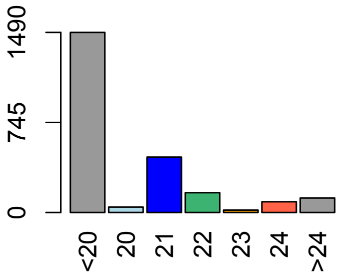

Allenscore: 0.5

Target Interaction:

```
5'- AGUACUAGGAGACAUAAUCA   AT3G27925.1
    | | | | | : | | | | | | | | | | | | | |
3'- UCAUGGUCCUCUGUAUUAGU   CI_cgr-dp_775
```

Target Site: 1305

Superfamily: SupFam_33

miRNAin ccm: N/A

Published name: N/A

Confirmed Targeting

2nd-siRNA	*NanoPARE*
ccm	*ccm*
cpe-2015	*cpe-2015*
cpe-2017	
cgr-dp	*cgr-dp*
cgr-pm	
cgr-mass	
cin	*cin*

DEG1

AT3G27925 - CI_cgr-dp_775

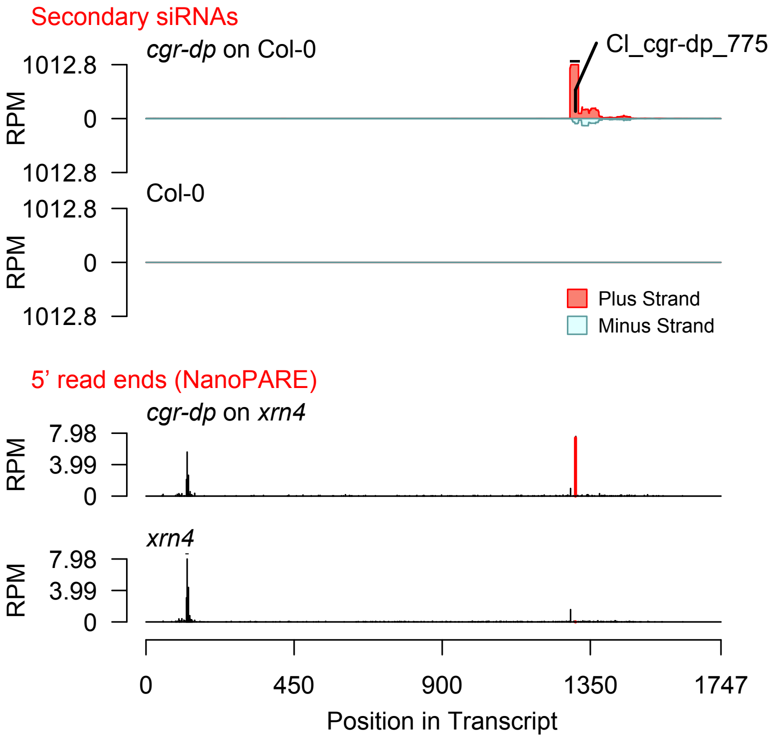

2nd siRNAs: Phase diagram

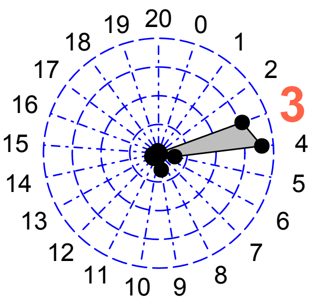

2nd siRNAs: Size distribution

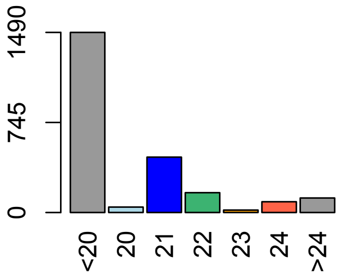

Allenscore: 3.5

Target Interaction:

```
5'- GGAGACUAGUACUAGGAGACAUAAUCA    AT3G27925.1
      | |      | | | | | | | : | | | | | | | | | | | | | |
3'- CC - - - GAUCAUGGUCCUCUGUAUUAGU    Cl_cgr-pm_154
```

Target Site: 1305

Superfamily: SupFam_33

miRNAin ccm: N/A

Published name: N/A

Confirmed Targeting

2nd-siRNA	*NanoPARE*
ccm	*ccm*
cpe-2015	*cpe-2015*
cpe-2017	
cgr-dp	*cgr-dp*
cgr-pm	
cgr-mass	
cin	*cin*

DEG1

AT3G27925 - Cl_cgr-pm_154

Secondary siRNAs

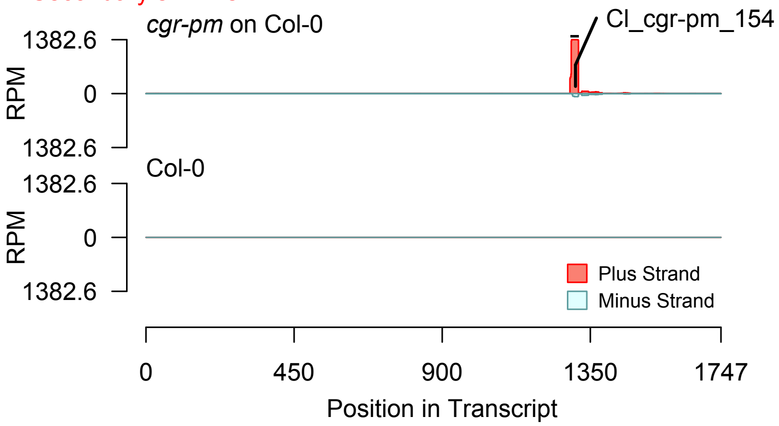

Degradome hits not found for sRNA

2nd siRNAs: Phase diagram

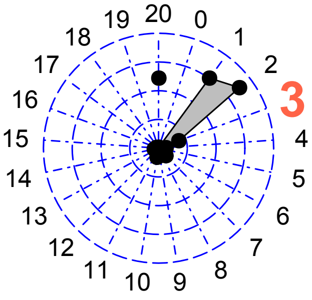

2nd siRNAs: Size distribution

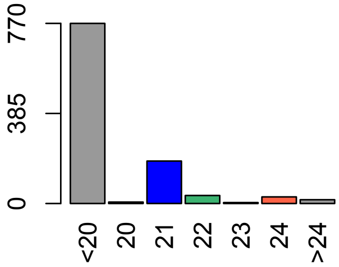

Allenscore: 1.5

Target Interaction:

```
5'- CUAGUACUAGGAGACAUAAUCA    AT3G27925.1
     |||||||:||||||||||||||
3'- AAUCAUGGUCCUCUGUAUUAGU    Cl_cgr-pm_456
```

Target Site: 1305

Superfamily: SupFam_33

miRNAin ccm: N/A

Published name: N/A

Confirmed Targeting

2nd-siRNA	*NanoPARE*
ccm	*ccm*
cpe-2015	*cpe-2015*
cpe-2017	
cgr-dp	*cgr-dp*
cgr-pm	
cgr-mass	
cin	*cin*

DEG1

AT3G27925 - Cl_cgr-pm_456

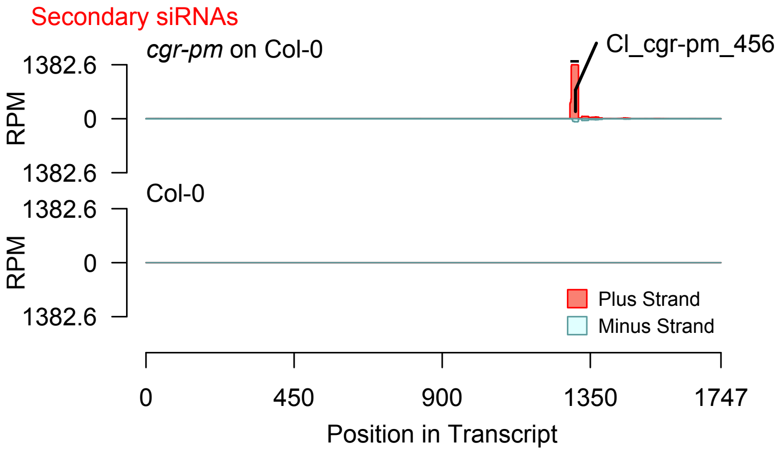

Degradome hits not found for sRNA

2nd siRNAs: Phase diagram

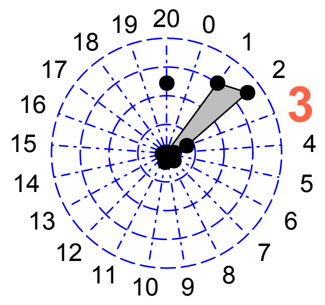

2nd siRNAs: Size distribution

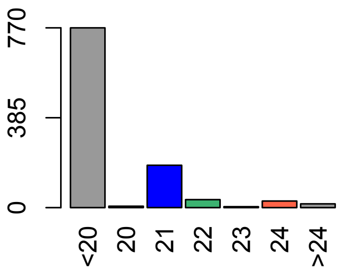

Allenscore: 1.5

Target Interaction:

```
5'- AGUACUAGGAGACAUAAUCA   AT3G27925.1
     ||||:||||||||||||||
3'- CCAUGGUCCUCUGUAUUAGU   Cl_cgr-pm_11629
```

Target Site: 1305

Superfamily: SupFam_33

miRNAin ccm: N/A

Published name: N/A

Confirmed Targeting

2nd-siRNA	*NanoPARE*
ccm	*ccm*
cpe-2015	*cpe-2015*
cpe-2017	
cgr-dp	*cgr-dp*
cgr-pm	
cgr-mass	
cin	*cin*

DEG1

AT3G27925 - Cl_cgr-pm_11629

Secondary siRNAs

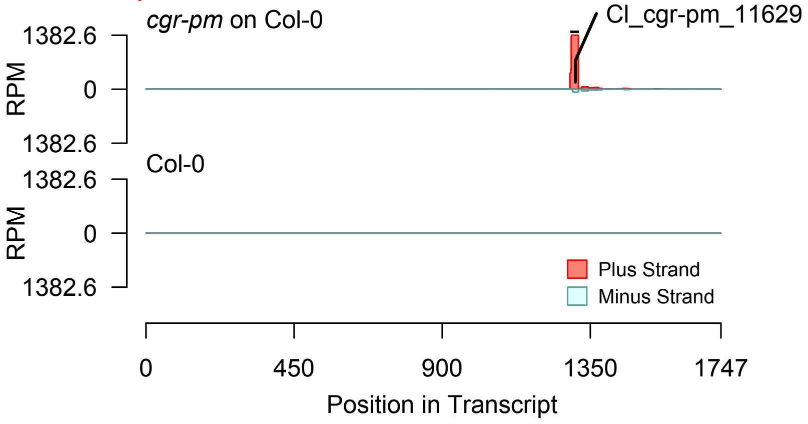

2nd siRNAs: Phase diagram

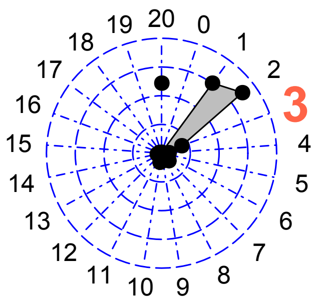

2nd siRNAs: Size distribution

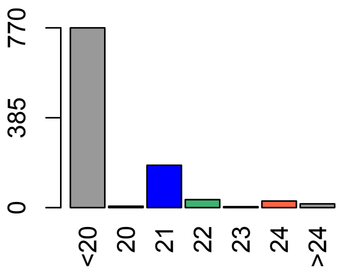

Degradome hits not found for sRNA

Allenscore: 3.5

Target Interaction:

```
5'- GGAGACUAGUACUAGGAGACAUAAUCA    AT3G27925.1
     | |       | | | | | | | : | | | | | | | | | | | | | |
3'- CC - - - GAUCAUGGUCCUCUGUAUUAGU   Cl_cgr-mass_424
```

Target Site: 1305

Superfamily: SupFam_33

miRNAin ccm: N/A

Published name: N/A

Confirmed Targeting

2nd-siRNA	*NanoPARE*
ccm	*ccm*
cpe-2015	*cpe-2015*
cpe-2017	
cgr-dp	*cgr-dp*
cgr-pm	
cgr-mass	
cin	*cin*

DEG1

AT3G27925 - Cl_cgr-mass_424

Secondary siRNAs

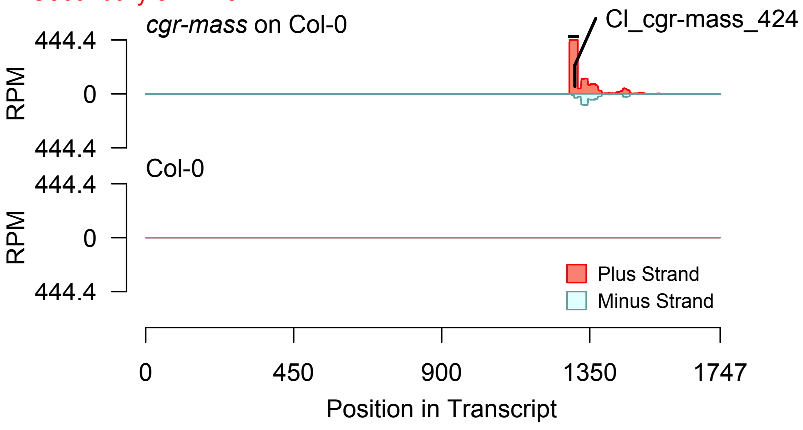

2nd siRNAs: Phase diagram

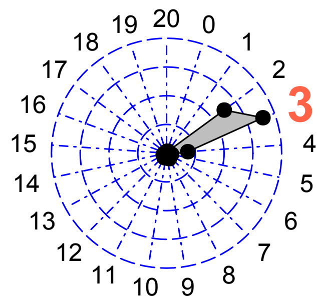

2nd siRNAs: Size distribution

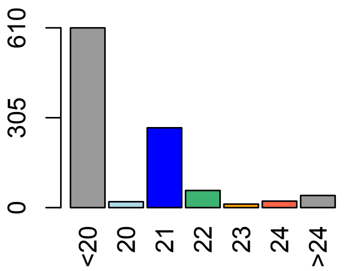

Degradome hits not found for sRNA

Allenscore: 0.5

Target Interaction:

```
5'- AGUACUAGGAGACAUAAUCA   AT3G27925.1
     | | | | | : | | | | | | | | | | | | | |
3'- UCAUGGUCCUCUGUAUUAGU   Cl_cgr-mass_5502
```

Target Site: 1305

Superfamily: SupFam_33

miRNAin ccm: N/A

Published name: N/A

Confirmed Targeting

2nd-siRNA	*NanoPARE*
ccm	*ccm*
cpe-2015	*cpe-2015*
cpe-2017	
cgr-dp	*cgr-dp*
cgr-pm	
cgr-mass	
cin	*cin*

DEG1

AT3G27925 - Cl_cgr-mass_5502

Secondary siRNAs

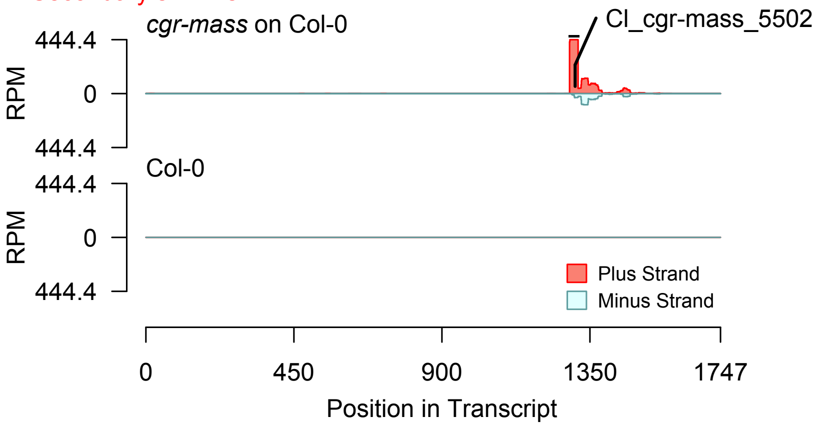

2nd siRNAs: Phase diagram

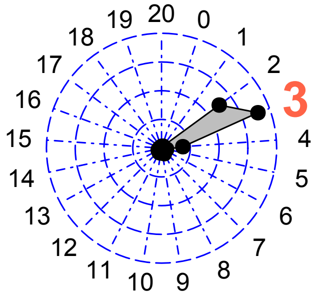

2nd siRNAs: Size distribution

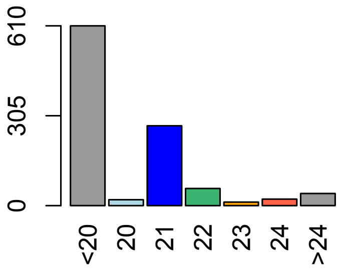

Degradome hits not found for sRNA

Allenscore: 4.5

Target Interaction:

```
5'- UGUGGUUACAUUGCACCAGAAUA   AT4G28490.1
     : :   | | | | | |   : | | | | | | | | | | | | |
3'- GUCCCAAUGCGACGUGGUCUUAU   Cl_ccm_818
```

Target Site: 2815

Superfamily: SupFam_124

miRNAin ccm: No

Published name: Not published

Confirmed Targeting

2nd-siRNA	*NanoPARE*
ccm	*ccm*
cpe-2015	*cpe-2015*
cpe-2017	
cgr-dp	*cgr-dp*
cgr-pm	
cgr-mass	
cin	*cin*

HAE

AT4G28490 - Cl_ccm_818

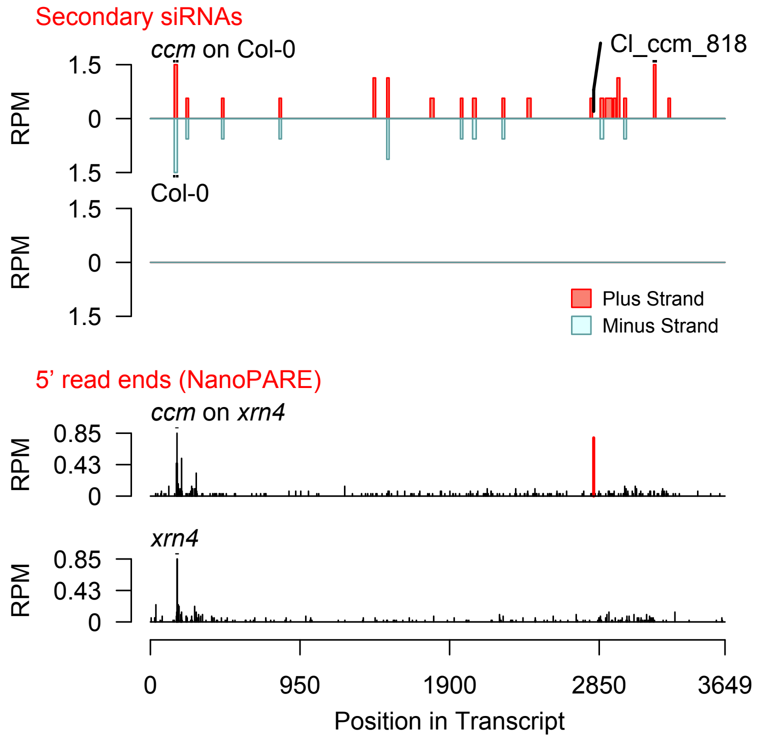

Diff. Exp. secondary siRNA locus not found

Allenscore: 5

Target Interaction:

```
5'- UAAACGCCAAGAGGAACUUCC   AT5G46580.1
     | | | | | | | |   | | | : | | | | | |
3'- UUUUGCGGUGCUCUUUGAAGU   Cl_cgr-dp_29
```

Target Site: 1929

Superfamily: SupFam_26

miRNAin ccm: Yes

Published name: N/A

Confirmed Targeting

2nd-siRNA	*NanoPARE*
ccm	*ccm*
cpe-2015	*cpe-2015*
cpe-2017	
cgr-dp	*cgr-dp*
cgr-pm	
cgr-mass	
cin	*cin*

PPR_repeat-containing_protein

AT5G46580 - Cl_cgr-dp_29

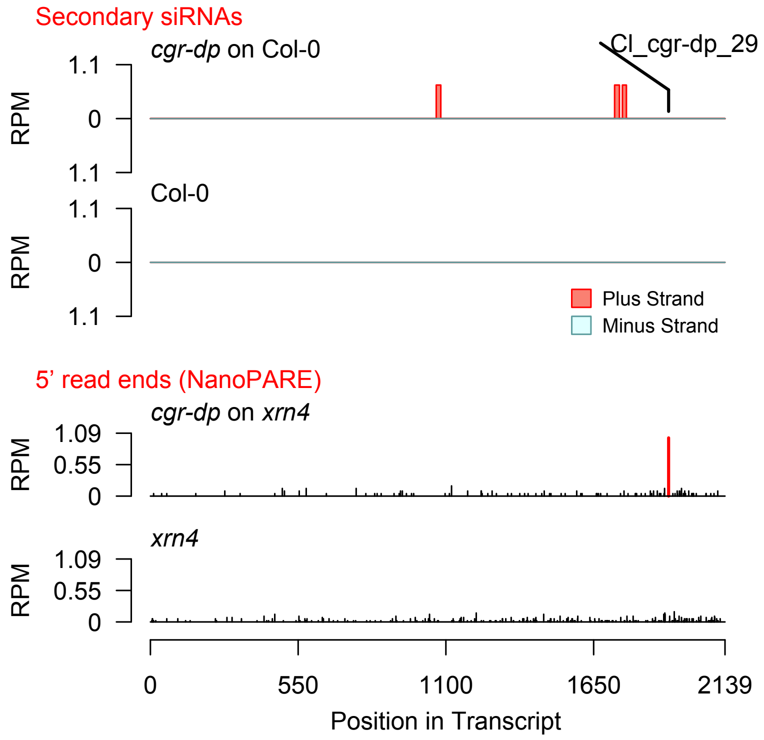

Diff. Exp. secondary siRNA locus not found

Allenscore: 4.5

Target Interaction:

```
5'- AAAGAUGCUACGACUCGUUCA   AT3G52610.1
      |   | | | : | | | | | | | | | | | | |
3'- AUCCUAUGAUGCUGAGCAAGC   CI_ccm_205
```

Target Site: 1115

Superfamily: SupFam_18

miRNAin ccm: Yes

Published name: Not published

Confirmed Targeting

2nd-siRNA	*NanoPARE*
ccm	*ccm*
cpe-2015	*cpe-2015*
cpe-2017	
cgr-dp	*cgr-dp*
cgr-pm	
cgr-mass	
cin	*cin*

GATA_zinc_finger_protein

AT3G52610 - CI_ccm_205

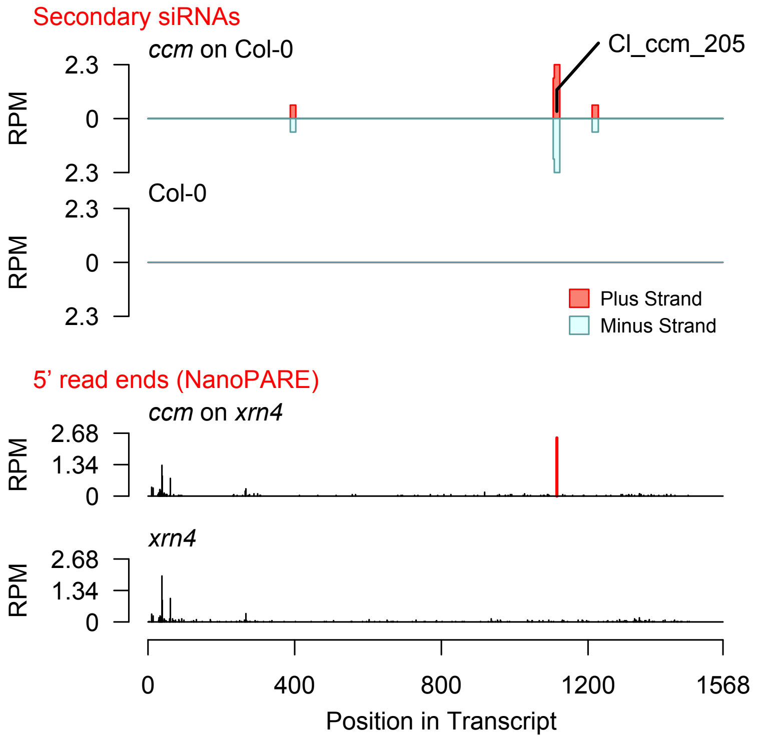

Diff. Exp. secondary siRNA locus not found

Allenscore: 2.5

Target Interaction:

```
5'- UAUAGUUGAAGCAGGAAUUGCG   AT2G46110.1
     : | | | | : | | | | | | | | | | | | : | | :
3'- GUAUCGACUUCGUCCUUAGCGU   CI_ccm_30
```

Target Site: 681

Superfamily: SupFam_50

miRNAin ccm: Yes

Published name: Not published

Confirmed Targeting

2nd-siRNA	*NanoPARE*
ccm	*ccm*
cpe-2015	*cpe-2015*
cpe-2017	
cgr-dp	*cgr-dp*
cgr-pm	
cgr-mass	
cin	*cin*

KPHMT1

AT2G46110 - CI_ccm_30

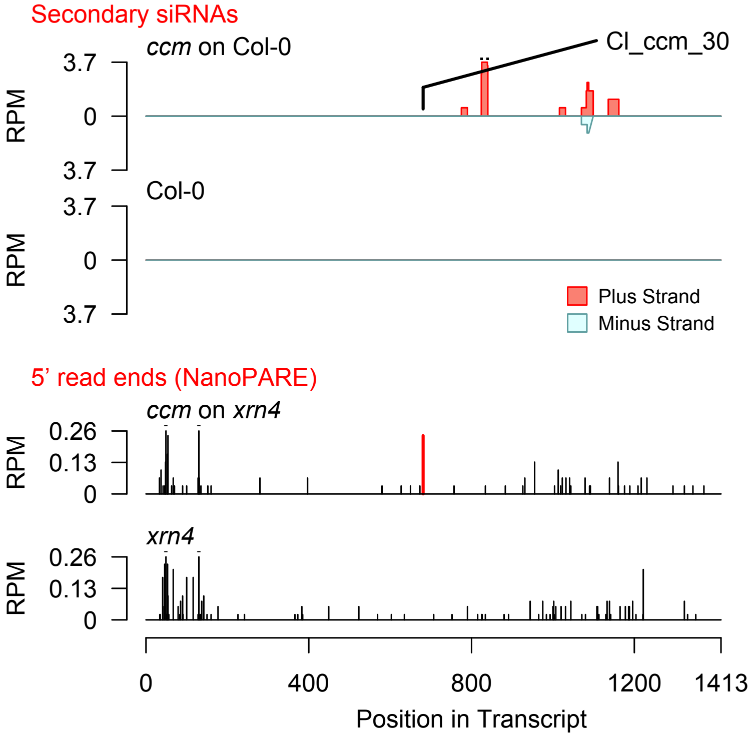

Diff. Exp. secondary siRNA locus not found

Allenscore: 5

Target Interaction:

```
5'- ACAGGAGAAGAAGGAAGACCA   AT1G18540.1
     ||||||||||  ||||||||   |
3'- UGUCCUCUUCAUCCUUCUGAU   Cl_cin_11140
```

Target Site: 607

Superfamily: SupFam_1431

miRNAin ccm: N/A

Published name: N/A

Confirmed Targeting

2nd-siRNA	*NanoPARE*
ccm	*ccm*
cpe-2015	*cpe-2015*
cpe-2017	
cgr-dp	*cgr-dp*
cgr-pm	
cgr-mass	
cin	*cin*

Ribosomal_protein_L6_family_protein

AT1G18540 - Cl_cin_11140

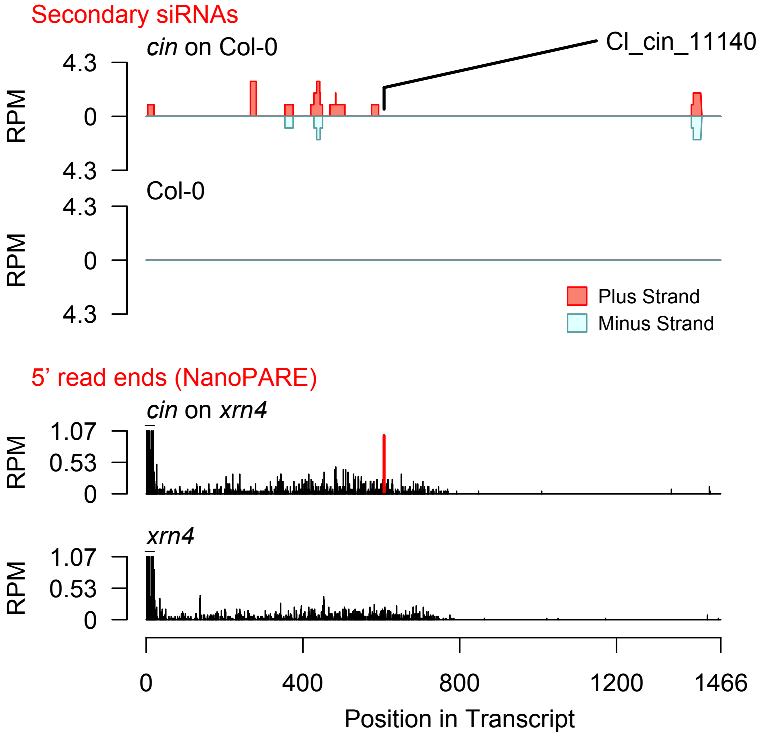

Diff. Exp. secondary siRNA locus not found

Allenscore: 0.5

Target Interaction:

```
5'- GAUUGCUGUUAAGAAACUGAA   AT2G17220.1
     : | | | | | | | | | | | | | | | | | | | |
3'- UUAACGACAAUUCUUUGACUU   Cl_ccm_9385
```

Target Site: 799

Superfamily: SupFam_320

miRNAin ccm: Yes

Published name: Not published

Confirmed Targeting

2nd-siRNA	*NanoPARE*
ccm	*ccm*
cpe-2015	*cpe-2015*
cpe-2017	
cgr-dp	*cgr-dp*
cgr-pm	
cgr-mass	
cin	*cin*

Kin3

AT2G17220 - Cl_ccm_9385

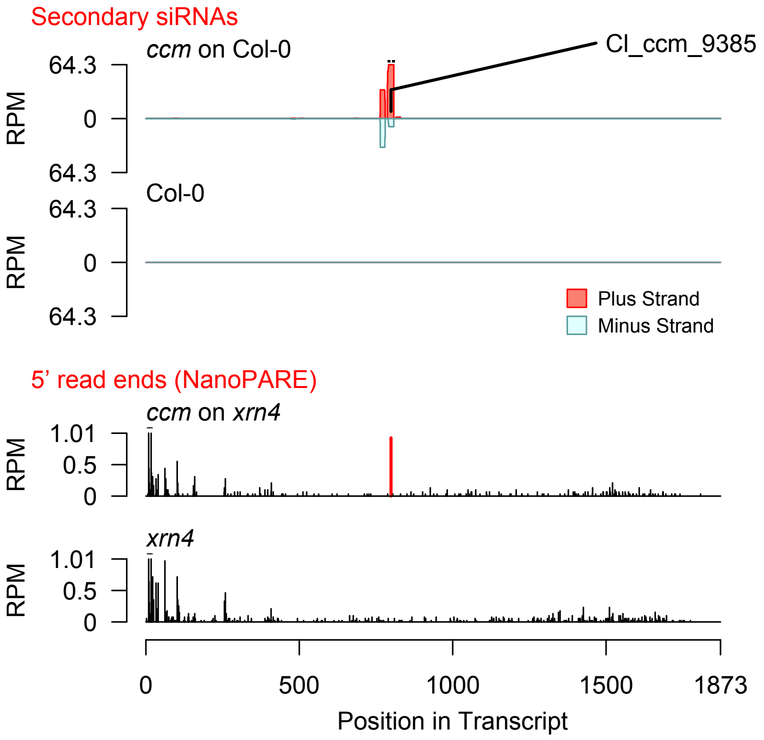

Diff. Exp. secondary siRNA locus not found

Allenscore: 2

Target Interaction:

```
5'- UCGGGAUGCUGAAGAUGCUA   AT1G02840.1
       ||||||||||||||||||
3'- CUCCCUACGACUUCUACGAU   Cl_cin_3533
```

Target Site: 370

Superfamily: SupFam_742

miRNAin ccm: N/A

Published name: N/A

Confirmed Targeting

2nd-siRNA	*NanoPARE*
ccm	*ccm*
cpe-2015	*cpe-2015*
cpe-2017	
cgr-dp	*cgr-dp*
cgr-pm	
cgr-mass	
cin	*cin*

SR34

AT1G02840 - Cl_cin_3533

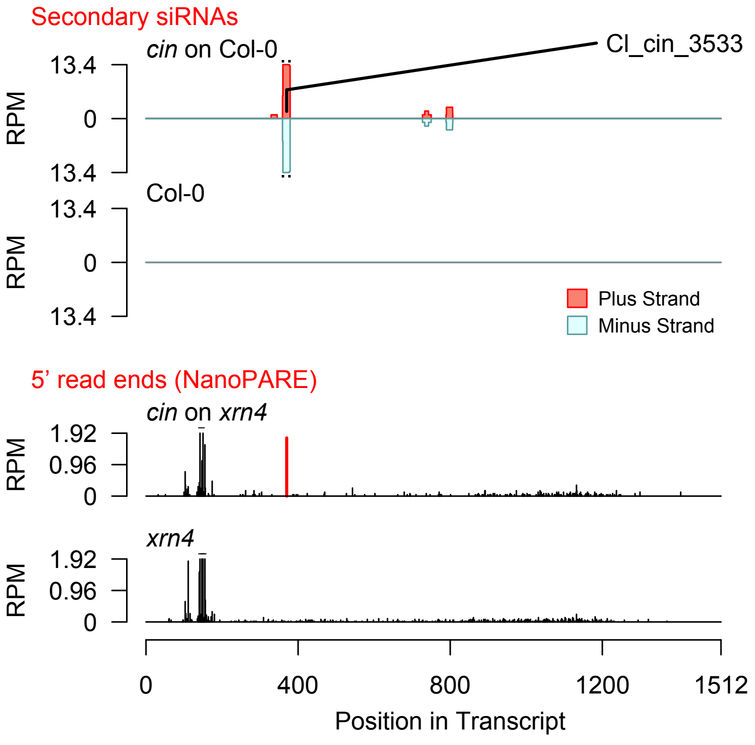

Diff. Exp. secondary siRNA locus not found

Allenscore: 3

Target Interaction:

```
5'- AGGAAGAUUGUGCAAAGAGAUA    AT4G01650.1
     | | | | | | | | | | | : | |   | | | | | | |
3'- UCCUUCUAACAUGUGUCUCUAU    CI_ccm_6791
```

Target Site: 1000

Superfamily: SupFam_134

miRNAin ccm: Yes

Published name: Not published

Confirmed Targeting

2nd-siRNA	*NanoPARE*
ccm	*ccm*
cpe-2015	*cpe-2015*
cpe-2017	
cgr-dp	*cgr-dp*
cgr-pm	
cgr-mass	
cin	*cin*

Polyketide_cyclase_dehydrase_and_lipid_t

AT4G01650 - CI_ccm_6791

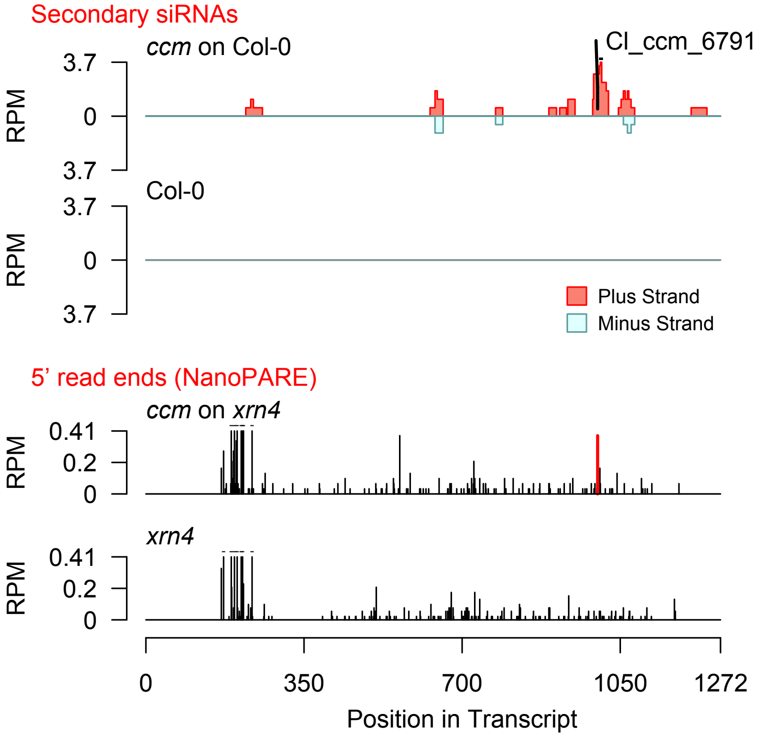

Diff. Exp. secondary siRNA locus not found

Allenscore: 3

Target Interaction:

```
5'- UGGACUUGUGAUGGAGGAGAUAA   AT3G46740.1
     | : |   | | | | | : | | | | | : | | | | | | |
3'- AUC - GAACAUUACCUUCUCUAUU   CI_cin_10092
```

Target Site: 1871

Superfamily: SupFam_1423

miRNAin ccm: N/A

Published name: N/A

Confirmed Targeting

2nd-siRNA	*NanoPARE*
ccm	*ccm*
cpe-2015	*cpe-2015*
cpe-2017	
cgr-dp	*cgr-dp*
cgr-pm	
cgr-mass	
cin	*cin*

TOC75-III

AT3G46740 - CI_cin_10092

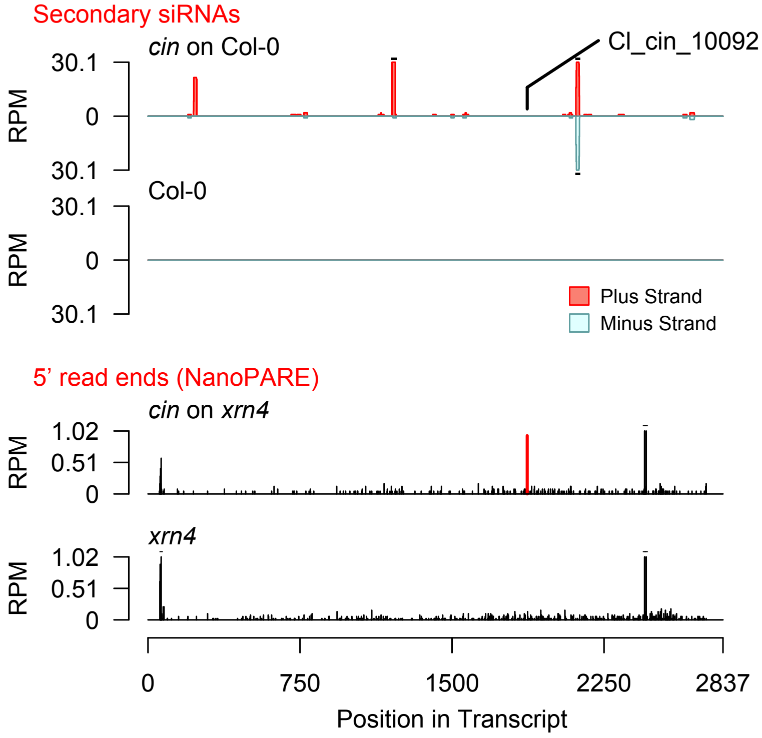

Diff. Exp. secondary siRNA locus not found

Allenscore: 5

Target Interaction:

```
5'- AAGAAUGACAGGAGGAGGUGC   AT5G24760.1
      ||||||||||| :||||||
3'- GUCUUACUGUCCAUCUCCACU   Cl_cgr-dp_67
```

Target Site: 1124

Superfamily: SupFam_4

miRNAin ccm: N/A

Published name: N/A

Confirmed Targeting

2nd-siRNA	*NanoPARE*
ccm	*ccm*
cpe-2015	*cpe-2015*
cpe-2017	
cgr-dp	*cgr-dp*
cgr-pm	
cgr-mass	
cin	*cin*

GroES-like_zinc-binding_dehydrogenase_fa

AT5G24760 - Cl_cgr-dp_67

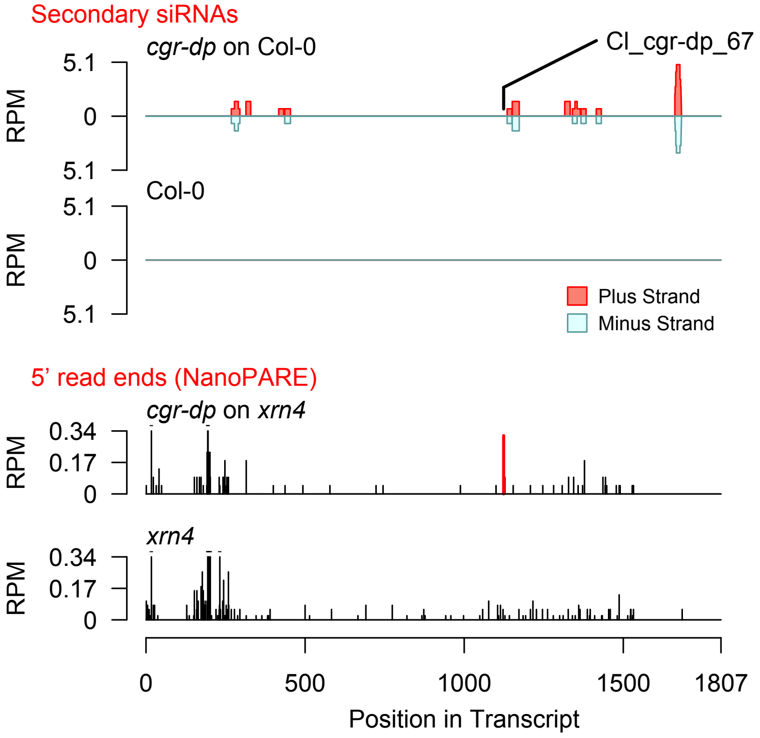

Diff. Exp. secondary siRNA locus not found

Allenscore: 3.5

Target Interaction:

```
5'- CUGGUCUGCAAAUCAUGGCACA   AT1G12820.1
     | |   | | | | : | | | | | | | | | |   | | |
3'- GAGCAGAUGUUUAGUACCAUGU   CI_ccm_2
```

Target Site: 472

Superfamily: SupFam_27

miRNAin ccm: Yes

Published name: ccm-MIR12497a*

Confirmed Targeting

2nd-siRNA	*NanoPARE*
ccm	*ccm*
cpe-2015	*cpe-2015*
cpe-2017	
cgr-dp	*cgr-dp*
cgr-pm	
cgr-mass	
cin	*cin*

AFB3

AT1G12820 - CI_ccm_2

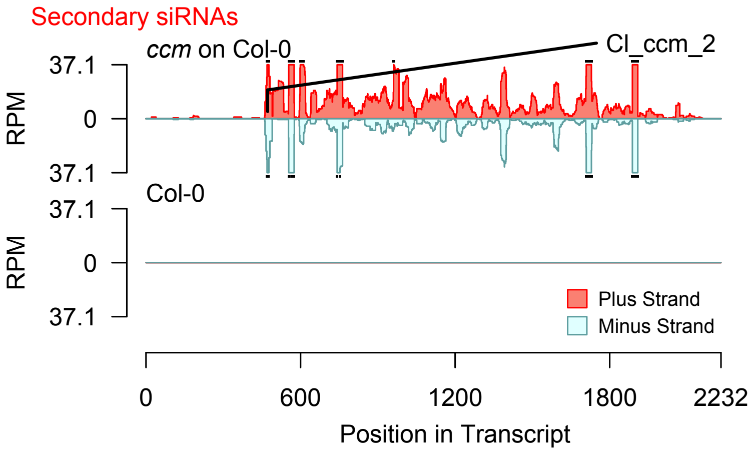

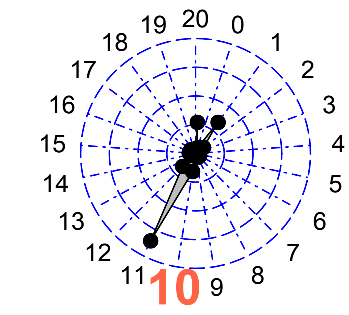

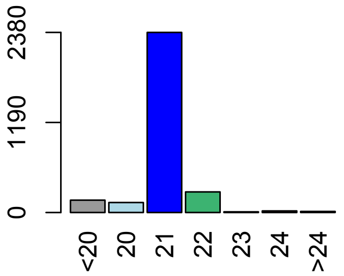

Degradome hits not found for sRNA

Allenscore: 4

Target Interaction:

```
5'- UCUGGUCUGCAAAUCAUGGC   AT1G12820.1
      | |   | | | | : | | | | | | | | | |
3'- UGAGCAGAUGUUUAGUACCA   Cl_ccm_98
```

Target Site: 469

Superfamily: SupFam_27

miRNAin ccm: Yes

Published name: Not published

Confirmed Targeting

2nd-siRNA	*NanoPARE*
ccm	*ccm*
cpe-2015	*cpe-2015*
cpe-2017	
cgr-dp	*cgr-dp*
cgr-pm	
cgr-mass	
cin	*cin*

AFB3

AT1G12820 - Cl_ccm_98

Secondary siRNAs

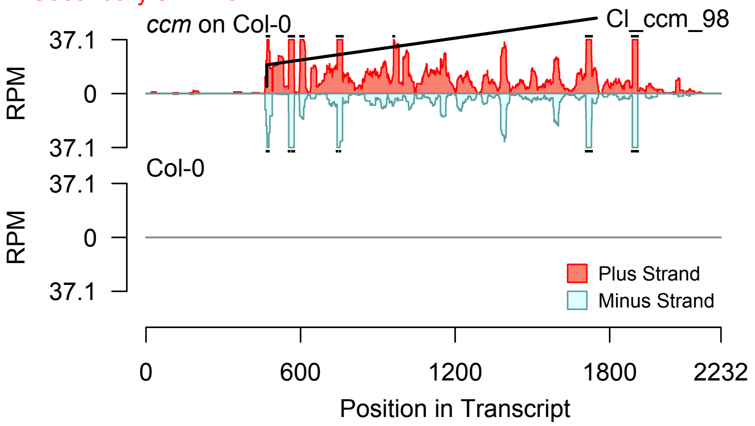

2nd siRNAs: Phase diagram

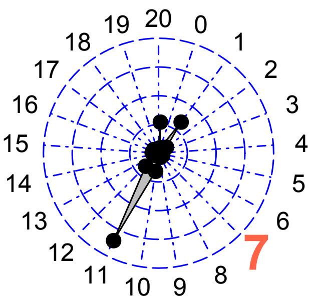

2nd siRNAs: Size distribution

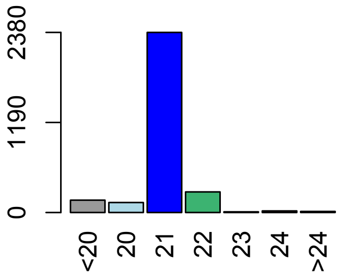

Degradome hits not found for sRNA

Allenscore: 4

Target Interaction:

```
5'- CUGGUCUGCAAAUCAUGGCACA   AT1G12820.1
      ||||  ||||||||||||  |||
3'- AACCAUACGUUUAGUACCAUGU   CI_ccm_614
```

Target Site: 472

Superfamily: SupFam_27

miRNAin ccm: Yes

Published name: ccm-MIR12497b

Confirmed Targeting

2nd-siRNA	*NanoPARE*
ccm	*ccm*
cpe-2015	*cpe-2015*
cpe-2017	
cgr-dp	*cgr-dp*
cgr-pm	
cgr-mass	
cin	*cin*

AFB3

AT1G12820 - CI_ccm_614

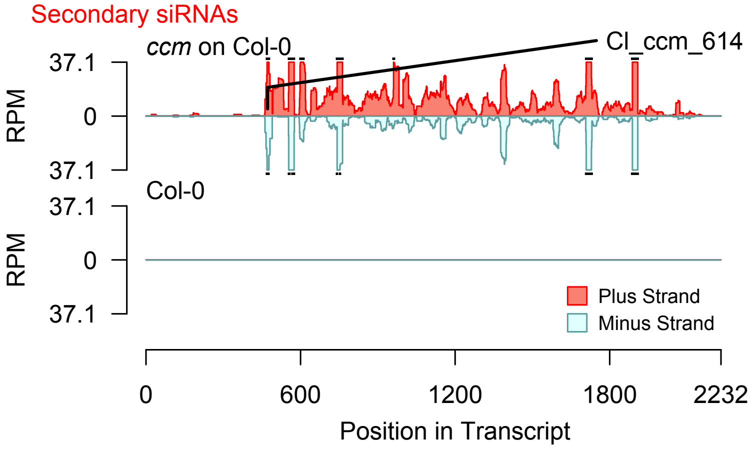

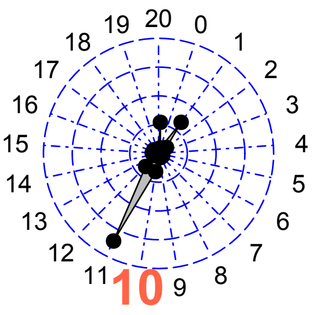

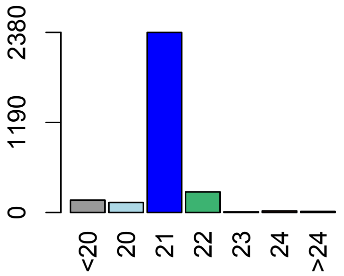

Degradome hits not found for sRNA

Allenscore: 5

Target Interaction:

```
5'- CAGAUCCUGAAU-CUUUUGCA    AT1G12820.1
      ||||||  ||||  |||||:||
3'- AUCUAGGUCUUACGAAAAUGU    CI_ccm_2562
```

Target Site: 1123

Superfamily: SupFam_196

miRNAin ccm: Yes

Published name: Not published

Confirmed Targeting

2nd-siRNA	*NanoPARE*
ccm	*ccm*
cpe-2015	*cpe-2015*
cpe-2017	
cgr-dp	*cgr-dp*
cgr-pm	
cgr-mass	
cin	*cin*

AFB3

AT1G12820 - CI_ccm_2562

Secondary siRNAs

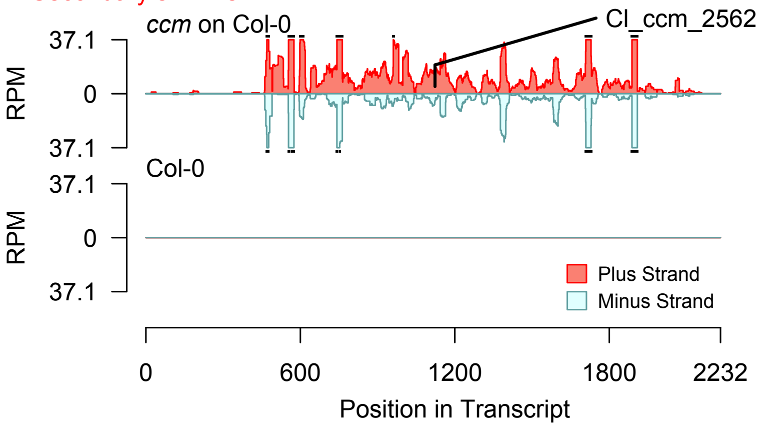

2nd siRNAs: Phase diagram

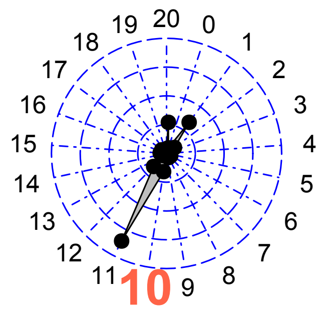

2nd siRNAs: Size distribution

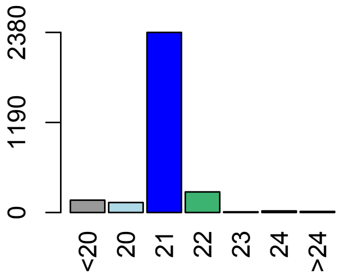

Degradome hits not found for sRNA

Allenscore: 3

Target Interaction:

```
5'- CUGGUCUGCAAAUCAUGGCACA   AT1G12820.1
     ||  |||||||||||||||  |||
3'- GAGCAGACGUUUAGUACCAUGU   Cl_cpe-2015_7
```

Target Site: 472

Superfamily: SupFam_27

miRNAin ccm: Yes

Published name: N/A

Confirmed Targeting

2nd-siRNA	*NanoPARE*
ccm	*ccm*
cpe-2015	*cpe-2015*
cpe-2017	
cgr-dp	*cgr-dp*
cgr-pm	
cgr-mass	
cin	*cin*

AFB3

AT1G12820 - Cl_cpe-2015_7

Secondary siRNAs

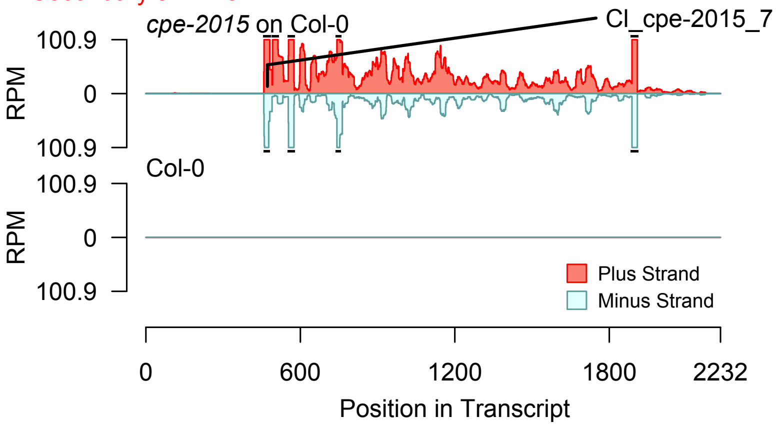

2nd siRNAs: Phase diagram

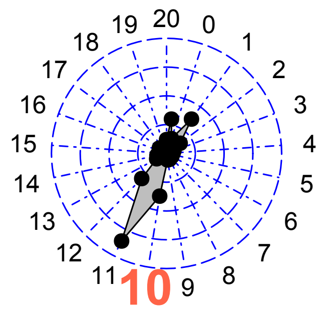

2nd siRNAs: Size distribution

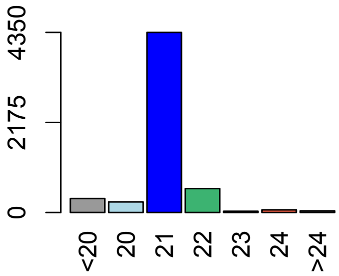

Degradome hits not found for sRNA

Allenscore: 3

Target Interaction:

```
5'- CUGGUCUGCAAAUCAUGGCACA   AT1G12820.1
     ||  ||||||||||||||||  |||
3'- GAGCAGACGUUUAGUACCAUGU   CI_cpe-2017_11
```

Target Site: 472

Superfamily: SupFam_27

miRNAin ccm: Yes

Published name: N/A

Confirmed Targeting

2nd-siRNA	*NanoPARE*
ccm	*ccm*
cpe-2015	*cpe-2015*
cpe-2017	
cgr-dp	*cgr-dp*
cgr-pm	
cgr-mass	
cin	*cin*

AFB3

AT1G12820 - CI_cpe-2017_11

Secondary siRNAs

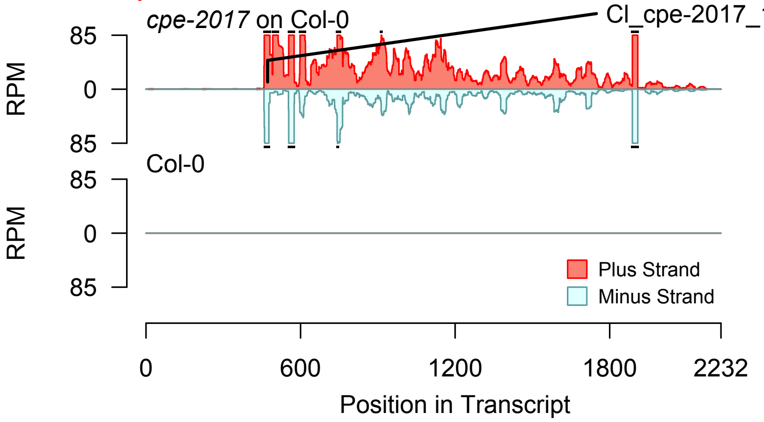

2nd siRNAs: Phase diagram

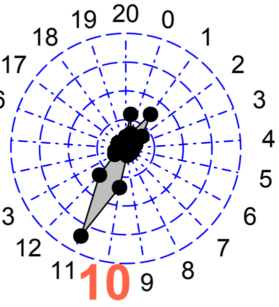

2nd siRNAs: Size distribution

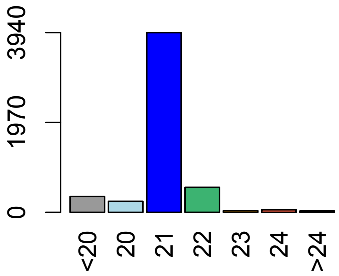

Degradome hits not found for sRNA

Allenscore: 5.5

Target Interaction:

```
5'- UUCCUGUACGGAGGAGAUGACU    AT3G01680.1
     |   | | | : | | | | | | | | | | |   | | |
3'- AUGGAUAUGCCUCCUCUCCUGU    CI_ccm_535
```

Target Site: 1753

Superfamily: SupFam_5

miRNAin ccm: Yes

Published name: ccm-MIR12480

Confirmed Targeting

2nd-siRNA	*NanoPARE*
ccm	*ccm*
cpe-2015	*cpe-2015*
cpe-2017	
cgr-dp	*cgr-dp*
cgr-pm	
cgr-mass	
cin	*cin*

SEOR1

AT3G01680 - CI_ccm_535

Secondary siRNAs

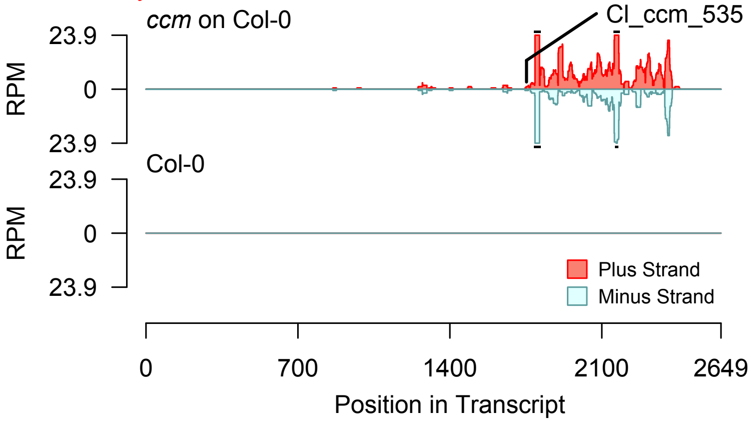

2nd siRNAs: Phase diagram

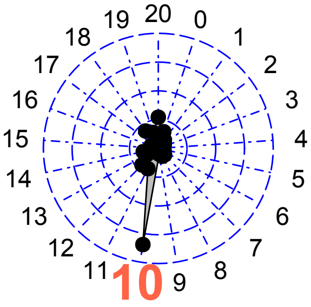

2nd siRNAs: Size distribution

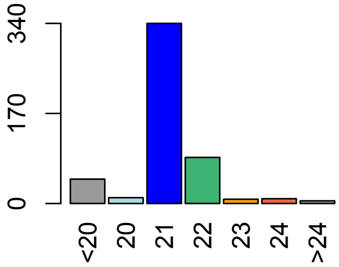

Degradome hits not found for sRNA

Allenscore: 5.5

Target Interaction:

```
5'- UUCCUGUACGGAGGAGAUGACU    AT3G01680.1
    |    | | : | | | | | | | | | | |   | | | |
3'- ACAGAUAUGCCUCCUCUCCUGA    Cl_ccm_6321
```

Target Site: 1753

Superfamily: SupFam_5

miRNAin ccm: Yes

Published name: Not published

Confirmed Targeting

2nd-siRNA	*NanoPARE*
ccm	*ccm*
cpe-2015	*cpe-2015*
cpe-2017	
cgr-dp	*cgr-dp*
cgr-pm	
cgr-mass	
cin	*cin*

SEOR1

AT3G01680 - Cl_ccm_6321

Secondary siRNAs

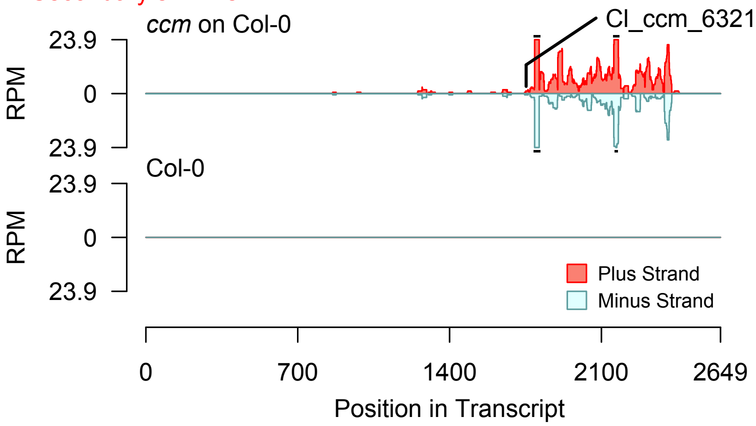

2nd siRNAs: Phase diagram

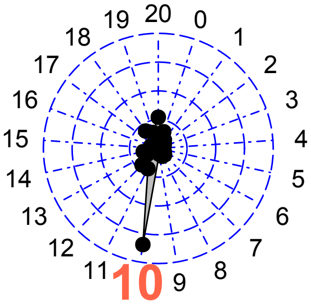

2nd siRNAs: Size distribution

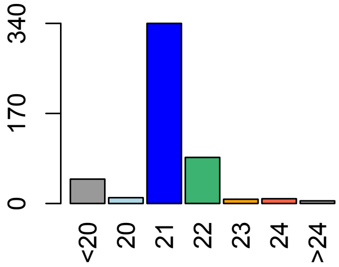

Degradome hits not found for sRNA

Allenscore: 6

Target Interaction:

```
5'- GGCCACUUUGAUUUCCACAUC   AT3G01680.1
     : | | | | | | | : : | | : | |   | | | | |
3'- GUGGUGAAGUUAGAGUUGUAG   CI_ccm_315
```

Target Site: 2250

Superfamily: SupFam_19

miRNAin ccm: Yes

Published name: ccm-MIR12464

Confirmed Targeting

2nd-siRNA	NanoPARE
ccm	*ccm*
cpe-2015	*cpe-2015*
cpe-2017	
cgr-dp	*cgr-dp*
cgr-pm	
cgr-mass	
cin	*cin*

SEOR1

AT3G01680 - CI_ccm_315

Secondary siRNAs

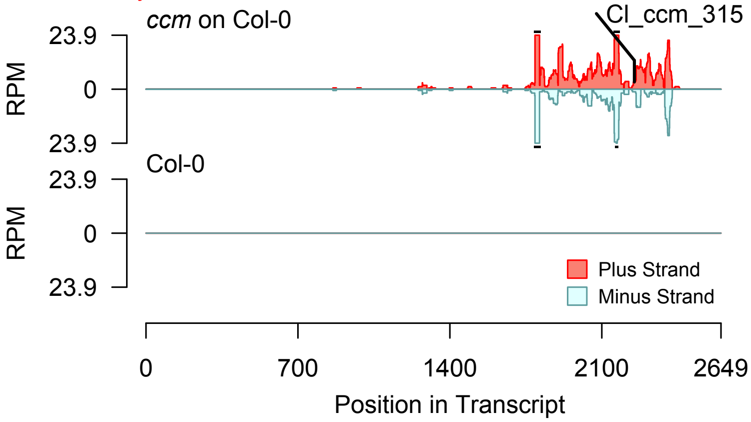

2nd siRNAs: Phase diagram

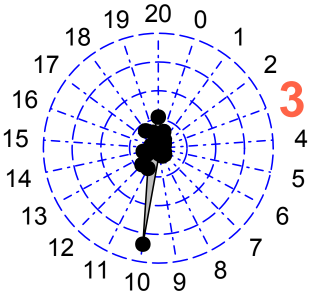

2nd siRNAs: Size distribution

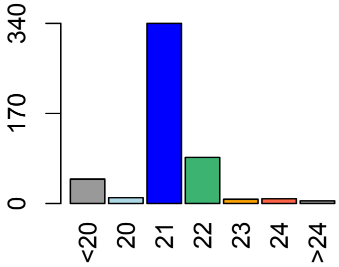

Degradome hits not found for sRNA

Allenscore: 5

Target Interaction:

```
5'- UCCUGUACGGAGGAGAUGACU   AT3G01680.1
      ||||||||||| ||||||
3'- UCGACAUGCCUCCGCUACUGU   Cl_cgr-mass_4275
```

Target Site: 1753

Superfamily: SupFam_5

miRNAin ccm: Yes

Published name: N/A

Confirmed Targeting

2nd-siRNA	*NanoPARE*
ccm	*ccm*
cpe-2015	*cpe-2015*
cpe-2017	
cgr-dp	*cgr-dp*
cgr-pm	
cgr-mass	
cin	*cin*

SEOR1

AT3G01680 - Cl_cgr-mass_4275

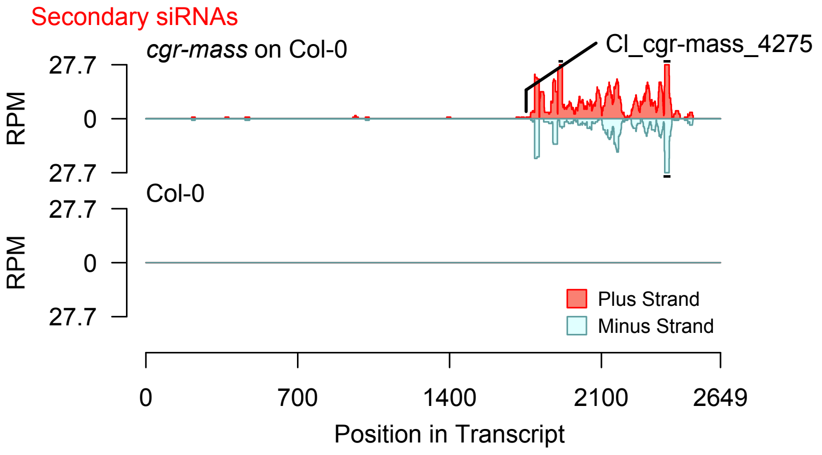

2nd siRNAs: Phase diagram

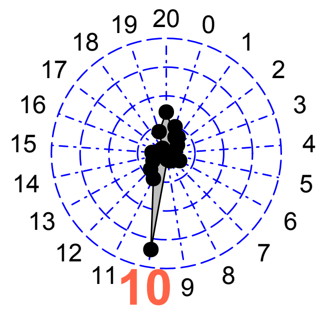

2nd siRNAs: Size distribution

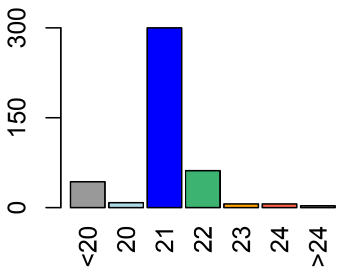

Degradome hits not found for sRNA

Allenscore: 5.5

Target Interaction:

```
5'- UUCCUGUACGGAGGAGAUGACU    AT3G01680.1
     |   | | | : | |   | | | | | | | |   | | | |
3'- AUGGAUAUACCUCCUCUUCUGA    CI_cgr-mass_5049
```

Target Site: 1753

Superfamily: SupFam_5

miRNAin ccm: Yes

Published name: N/A

Confirmed Targeting

2nd-siRNA	*NanoPARE*
ccm	*ccm*
cpe-2015	*cpe-2015*
cpe-2017	
cgr-dp	*cgr-dp*
cgr-pm	
cgr-mass	
cin	*cin*

SEOR1

AT3G01680 - CI_cgr-mass_5049

Secondary siRNAs

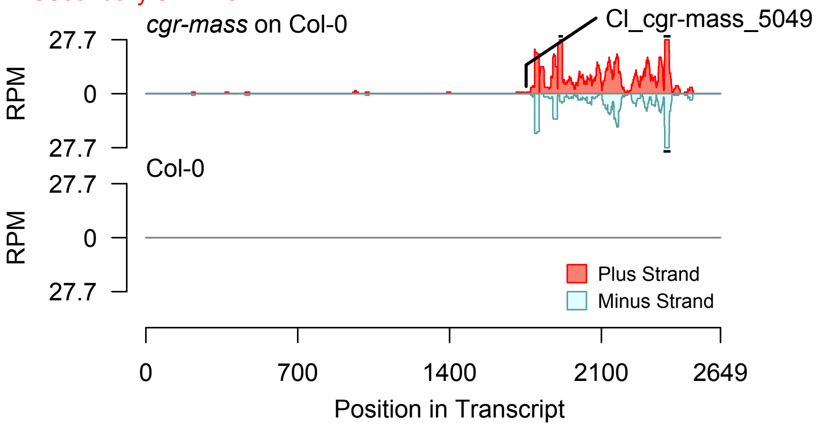

2nd siRNAs: Phase diagram

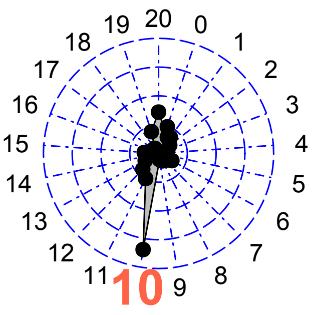

2nd siRNAs: Size distribution

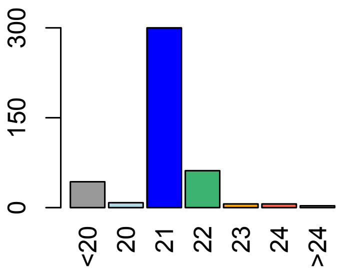

Degradome hits not found for sRNA

Allenscore: 3.5

Target Interaction:

```
5'- UUCCUGUACGGAGGAGAUGACU    AT3G01680.1
     | : | | | | | | | | | | | | | | | |    | | |
3'- AGGGACAUGCCUCCUCUUCUGU    Cl_cpe-2015_74
```

Target Site: 1753

Superfamily: SupFam_5

miRNAin ccm: Yes

Published name: N/A

Confirmed Targeting

2nd-siRNA	*NanoPARE*
ccm	*ccm*
cpe-2015	*cpe-2015*
cpe-2017	
cgr-dp	*cgr-dp*
cgr-pm	
cgr-mass	
cin	*cin*

SEOR1

AT3G01680 - Cl_cpe-2015_74

Secondary siRNAs

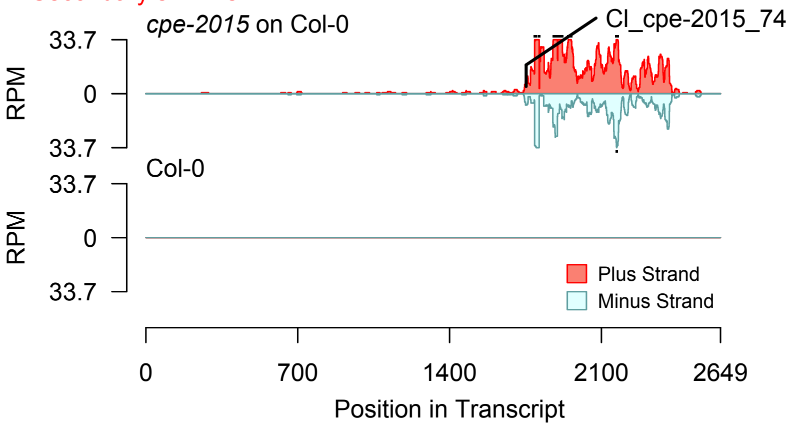

2nd siRNAs: Phase diagram

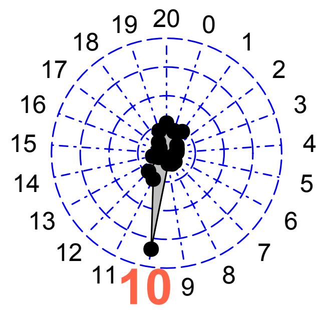

2nd siRNAs: Size distribution

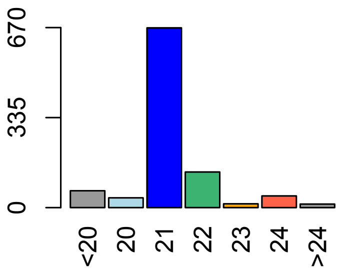

Degradome hits not found for sRNA

Allenscore: 5.5

Target Interaction:

```
5'- UUCCUGUACGGAGGAGAUGACU    AT3G01680.1
     |   | | | : | | | | | | | | | | |    | | |
3'- AUGGAUAUGCCUCCUCUCCUGU    CI_cpe-2015_154
```

Target Site: 1753

Superfamily: SupFam_5

miRNAin ccm: Yes

Published name: N/A

Confirmed Targeting

2nd-siRNA	*NanoPARE*
ccm	*ccm*
cpe-2015	*cpe-2015*
cpe-2017	
cgr-dp	*cgr-dp*
cgr-pm	
cgr-mass	
cin	*cin*

SEOR1

AT3G01680 - CI_cpe-2015_154

Secondary siRNAs

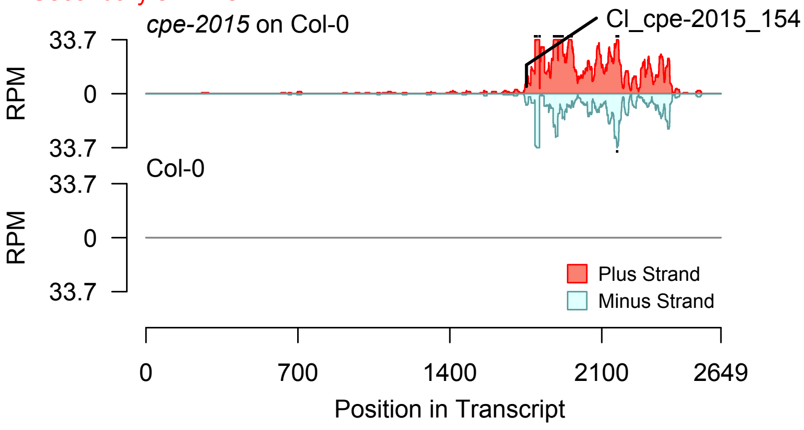

2nd siRNAs: Phase diagram

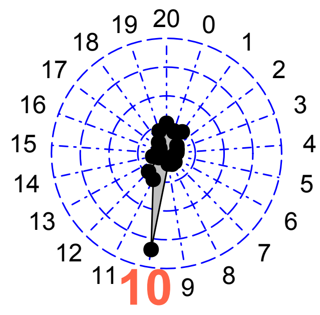

2nd siRNAs: Size distribution

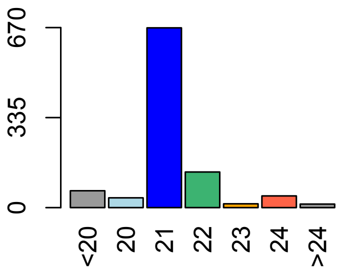

Degradome hits not found for sRNA

Allenscore: 5.5

Target Interaction:

```
5'- UUCCUGUACGGAGGAGAUGACU    AT3G01680.1
    |    | | : | | | | | | | | | | | |   | | | |
3'- ACAGAUAUGCCUCCUCUCCUGA    Cl_cpe-2015_1417
```

Target Site: 1753

Superfamily: SupFam_5

miRNAin ccm: Yes

Published name: N/A

Confirmed Targeting

2nd-siRNA	*NanoPARE*
ccm	*ccm*
cpe-2015	*cpe-2015*
cpe-2017	
cgr-dp	*cgr-dp*
cgr-pm	
cgr-mass	
cin	*cin*

SEOR1

AT3G01680 - Cl_cpe-2015_1417

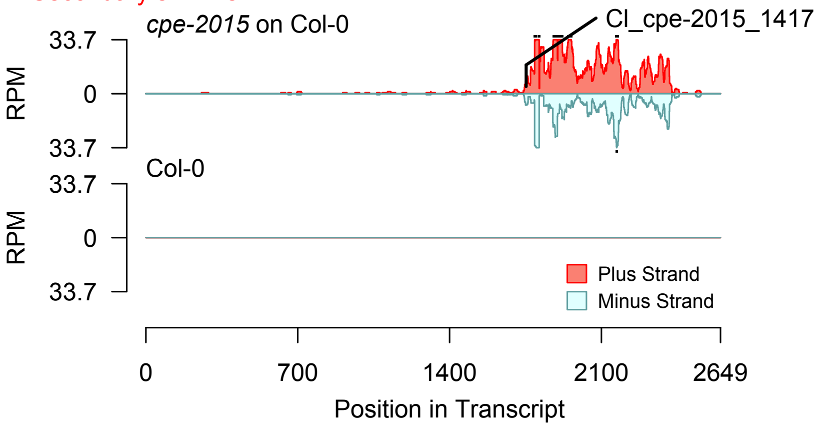

2nd siRNAs: Phase diagram

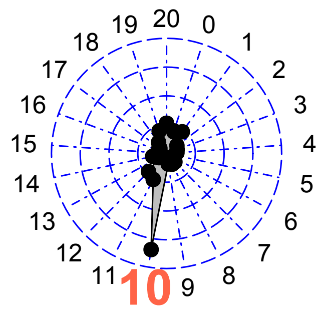

2nd siRNAs: Size distribution

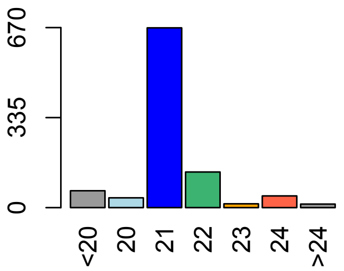

Degradome hits not found for sRNA

Allenscore: 6

Target Interaction:

```
5'- GGCCACUUUGAUUUCCACAUC   AT3G01680.1
      : | | | | | | | : : | | : | |   | | | | |
3'- GUGGUGAAGUUAGAGUUGUAG   CI_cpe-2015_191
```

Target Site: 2250

Superfamily: SupFam_19

miRNAin ccm: Yes

Published name: N/A

Confirmed Targeting

2nd-siRNA	*NanoPARE*
ccm	*ccm*
cpe-2015	*cpe-2015*
cpe-2017	
cgr-dp	*cgr-dp*
cgr-pm	
cgr-mass	
cin	*cin*

SEOR1

AT3G01680 - CI_cpe-2015_191

Secondary siRNAs

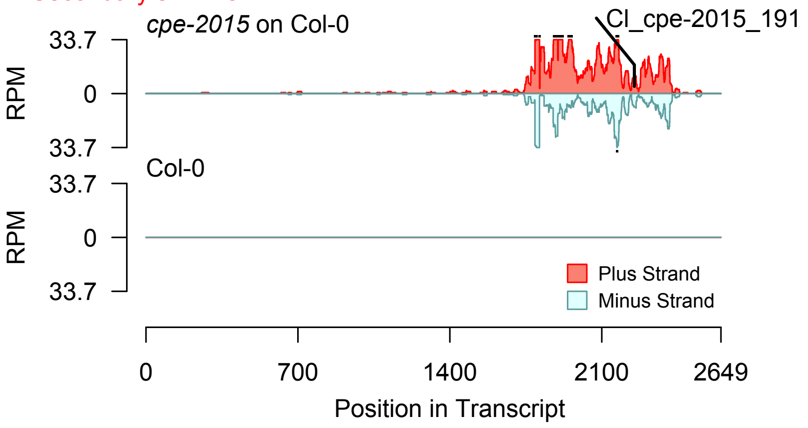

2nd siRNAs: Phase diagram

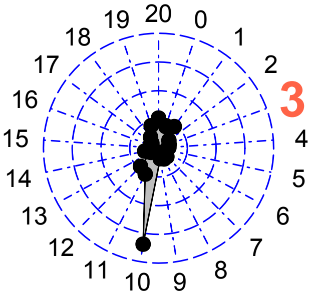

2nd siRNAs: Size distribution

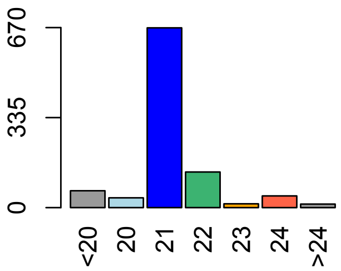

Degradome hits not found for sRNA

Allenscore: [illegible]

Target Interaction:

```
5'- AUUGCUUCGAGUGUCAGCGUC   AT3G01680.1
     | : | : | | | | | | |   |   | | | : | : | |
3'- UGAUGAAGCU - AGAGUUGUAG   Cl_cpe-2015_3720
```

Target Site: [illegible]

Superfamily: SupFam_19

miRNAin ccm: Yes

Published name: N/A

Confirmed Targeting

2nd-siRNA	*NanoPARE*
ccm	*ccm*
cpe-2015	*cpe-2015*
cpe-2017	
cgr-dp	*cgr-dp*
cgr-pm	
cgr-mass	
cin	*cin*

SEOR1

AT3G01680 - Cl_cpe-2015_3720

Secondary siRNAs

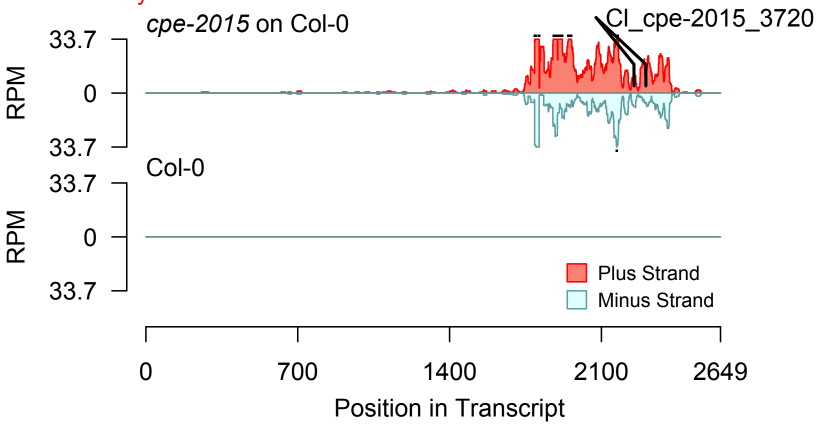

2nd siRNAs: Phase diagram

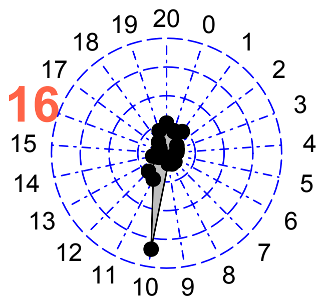

2nd siRNAs: Size distribution

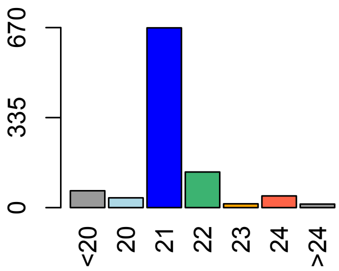

Degradome hits not found for sRNA

Allenscore: 3.5

Target Interaction:

```
5'- UUCCUGUACGGAGGAGAUGACU   AT3G01680.1
     | : | | | | | | | | | | | | | | |   | | |
3'- AGGGACAUGCCUCCUCUUCUGU   Cl_cpe-2017_89
```

Target Site: 1753

Superfamily: SupFam_5

miRNAin ccm: Yes

Published name: N/A

Confirmed Targeting

2nd-siRNA	*NanoPARE*
ccm	*ccm*
cpe-2015	*cpe-2015*
cpe-2017	
cgr-dp	*cgr-dp*
cgr-pm	
cgr-mass	
cin	*cin*

SEOR1

AT3G01680 - Cl_cpe-2017_89

Secondary siRNAs

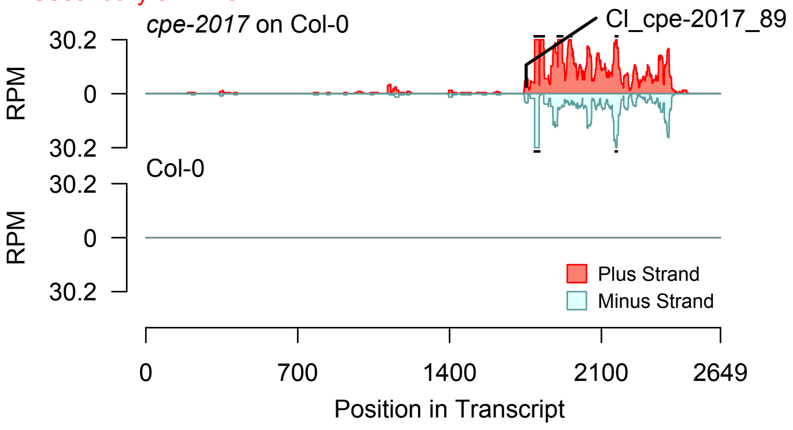

2nd siRNAs: Phase diagram

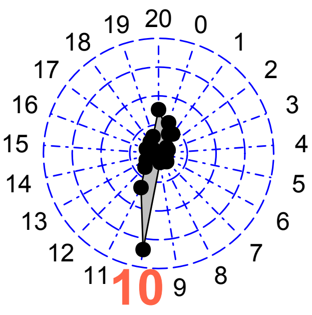

2nd siRNAs: Size distribution

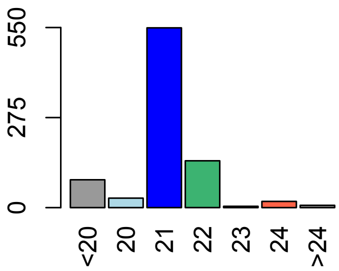

Degradome hits not found for sRNA

Allenscore: 5.5

Target Interaction:

```
5'- UUCCUGUACGGAGGAGAUGACU    AT3G01680.1
     |   | | | : | | | | | | | | | | | |    | | |
3'- AUGGAUAUGCCUCCUCUCCUGU    Cl_cpe-2017_181
```

Target Site: 1753

Superfamily: SupFam_5

miRNAin ccm: Yes

Published name: N/A

Confirmed Targeting

2nd-siRNA	*NanoPARE*
ccm	*ccm*
cpe-2015	*cpe-2015*
cpe-2017	
cgr-dp	*cgr-dp*
cgr-pm	
cgr-mass	
cin	*cin*

SEOR1

AT3G01680 - Cl_cpe-2017_181

2nd siRNAs: Phase diagram

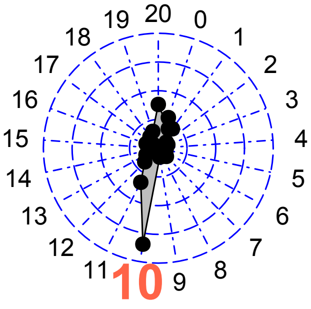

Secondary siRNAs

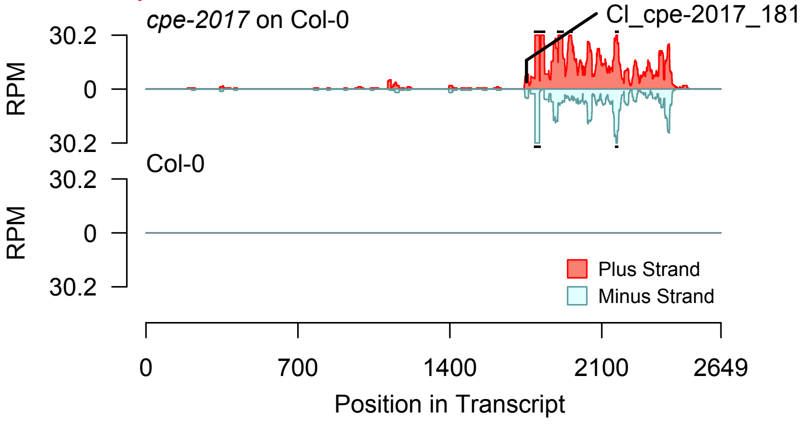

2nd siRNAs: Size distribution

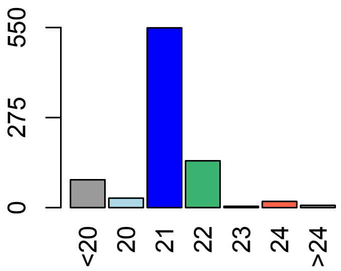

Degradome hits not found for sRNA

Allenscore: 5.5

Target Interaction:

```
5'- UUCCUGUACGGAGGAGAUGACU   AT3G01680.1
    |    ||:|||||||||||  ||||
3'- ACAGAUAUGCCUCCUCUCCUGA   Cl_cpe-2017_1111
```

Target Site: 1753

Superfamily: SupFam_5

miRNAin ccm: Yes

Published name: N/A

Confirmed Targeting

2nd-siRNA	*NanoPARE*
ccm	*ccm*
cpe-2015	*cpe-2015*
cpe-2017	
cgr-dp	*cgr-dp*
cgr-pm	
cgr-mass	
cin	*cin*

SEOR1

AT3G01680 - Cl_cpe-2017_1111

Secondary siRNAs

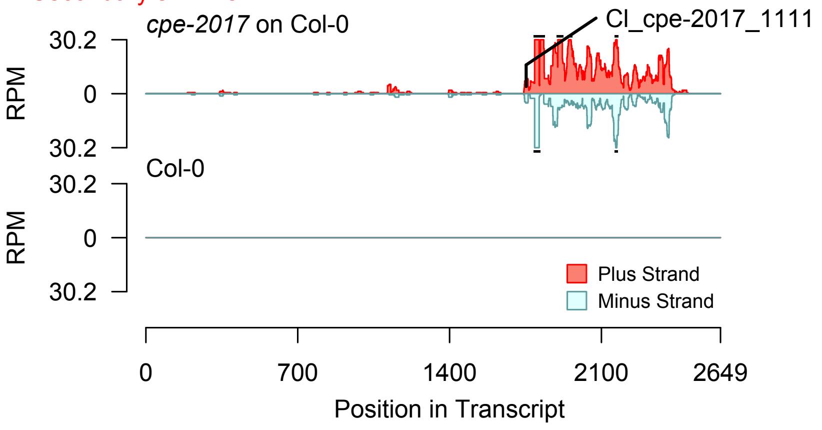

Degradome hits not found for sRNA

2nd siRNAs: Phase diagram

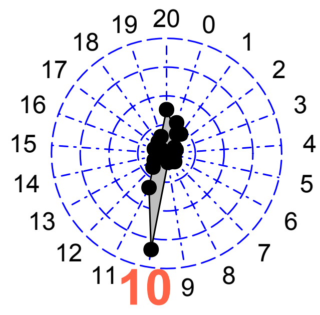

2nd siRNAs: Size distribution

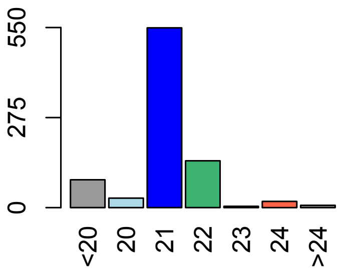

Allenscore: 6

Target Interaction:

```
5'- GGCCACUUUGAUUUCCACAUC   AT3G01680.1
      : | | | | | | | : : | | : | |   | | | | |
3'- GUGGUGAAGUUAGAGUUGUAG   Cl_cpe-2017_172
```

Target Site: 2250

Superfamily: SupFam_19

miRNAin ccm: Yes

Published name: N/A

Confirmed Targeting

2nd-siRNA	NanoPARE
ccm	ccm
cpe-2015	cpe-2015
cpe-2017	
cgr-dp	cgr-dp
cgr-pm	
cgr-mass	
cin	cin

SEOR1

AT3G01680 - Cl_cpe-2017_172

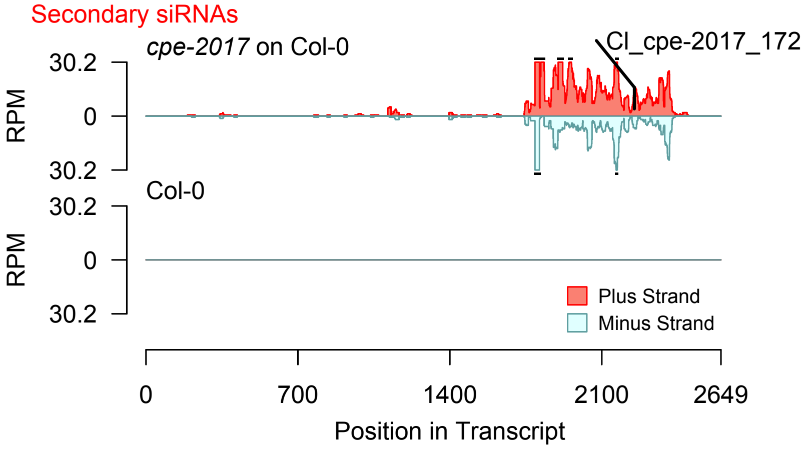

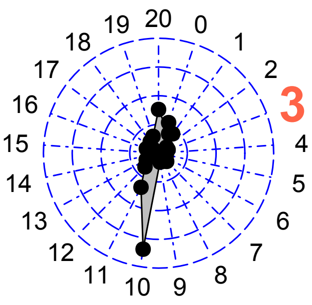

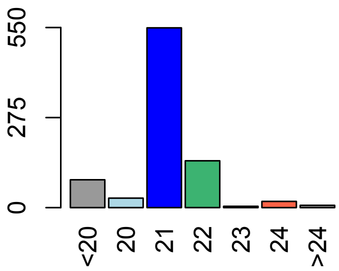

Degradome hits not found for sRNA

Allenscore: [illegible]

Target Interaction:

```
5'- AUUGCUUCGAGUGUCAGCGUC   AT3G01680.1
     | : | : | | | | | |   |   | | | : | : | |
3'- UGAUGAAGCU - AGAGUUGUAG   Cl_cpe-2017_2830
```

Target Site: [illegible]

Superfamily: SupFam_19

miRNAin ccm: Yes

Published name: N/A

Confirmed Targeting

2nd-siRNA	*NanoPARE*
ccm	*ccm*
cpe-2015	*cpe-2015*
cpe-2017	
cgr-dp	*cgr-dp*
cgr-pm	
cgr-mass	
cin	*cin*

SEOR1

AT3G01680 - Cl_cpe-2017_2830

Secondary siRNAs

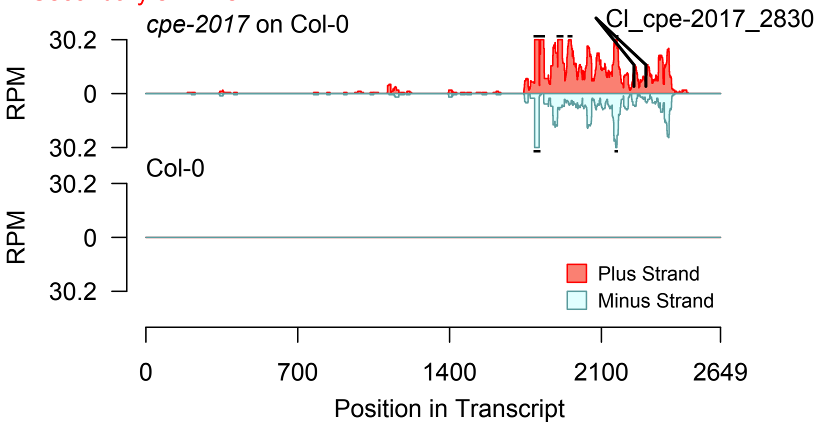

2nd siRNAs: Phase diagram

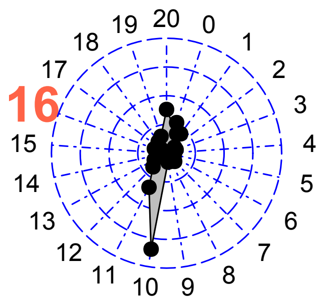

2nd siRNAs: Size distribution

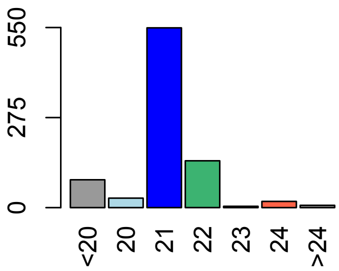

Degradome hits not found for sRNA

Allenscore: 5.5

Target Interaction:

```
5'- UUGUAUGGUGAGCCUGGAACU   AT4G29040.1
     |||||||:  |||  |||||||
3'- UACAUACUUCUCAGACCUUGU   Cl_cin_1915
```

Target Site: 776

Superfamily: SupFam_1368

miRNAin ccm: N/A

Published name: N/A

Confirmed Targeting

2nd-siRNA	*NanoPARE*
ccm	*ccm*
cpe-2015	*cpe-2015*
cpe-2017	
cgr-dp	*cgr-dp*
cgr-pm	
cgr-mass	
cin	*cin*

RPT2a

AT4G29040 - Cl_cin_1915

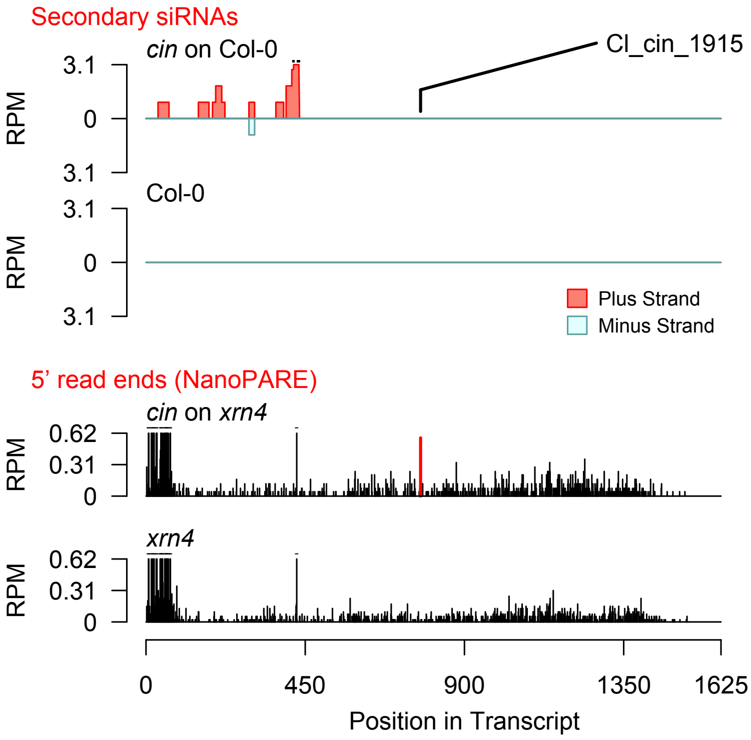

Diff. Exp. secondary siRNA locus not found

Allenscore: 5

Target Interaction:

```
5'- GUCCCCUCCAAAGAUUCCUGA   AT4G23630.1
     | | |   | |   | | | | | : | | | | |   | |
3'- CAGAGGUGGUUUUUAAGGUCU   Cl_cgr-dp_91
```

Target Site: 1080

Superfamily: SupFam_207

miRNAin ccm: N/A

Published name: N/A

Confirmed Targeting

2nd-siRNA	*NanoPARE*
ccm	*ccm*
cpe-2015	*cpe-2015*
cpe-2017	
cgr-dp	*cgr-dp*
cgr-pm	
cgr-mass	
cin	*cin*

BTI1

AT4G23630 - Cl_cgr-dp_91

Secondary siRNAs

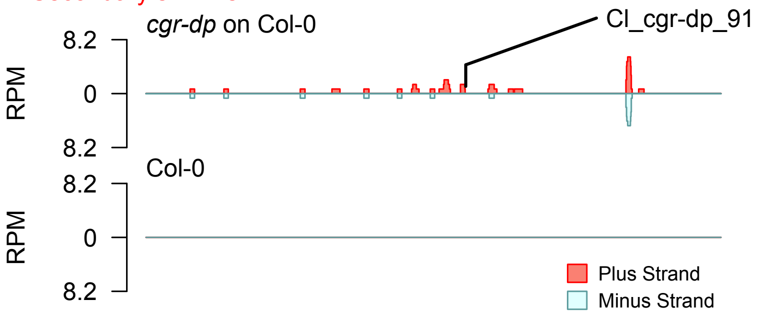

Diff. Exp. secondary siRNA locus not found

5' read ends (NanoPARE)

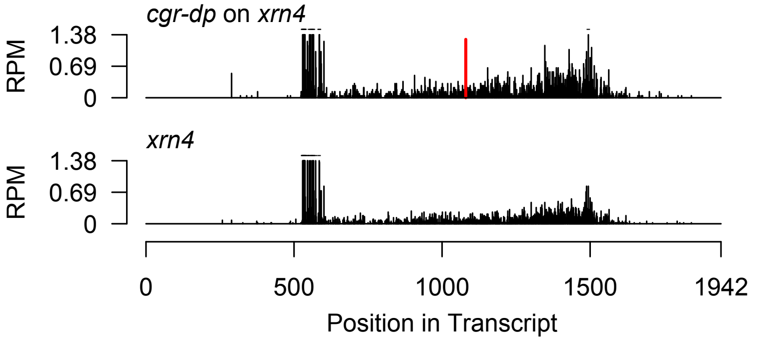

Allenscore: 4.5

Target Interaction:

```
5'- UGCAGUGUCGAGGGUUGGAAGU    AT4G27630.1
     | | | |    | |    | : | | | : | | | | | | |
3'- ACGU - AC - GUUCCUAACCUUCU    CI_cgr-dp_190
```

Target Site: 1649

Superfamily: SupFam_895

miRNAin ccm: N/A

Published name: N/A

Confirmed Targeting

2nd-siRNA	*NanoPARE*
ccm	*ccm*
cpe-2015	*cpe-2015*
cpe-2017	
cgr-dp	*cgr-dp*
cgr-pm	
cgr-mass	
cin	*cin*

GTG2

AT4G27630 - CI_cgr-dp_190

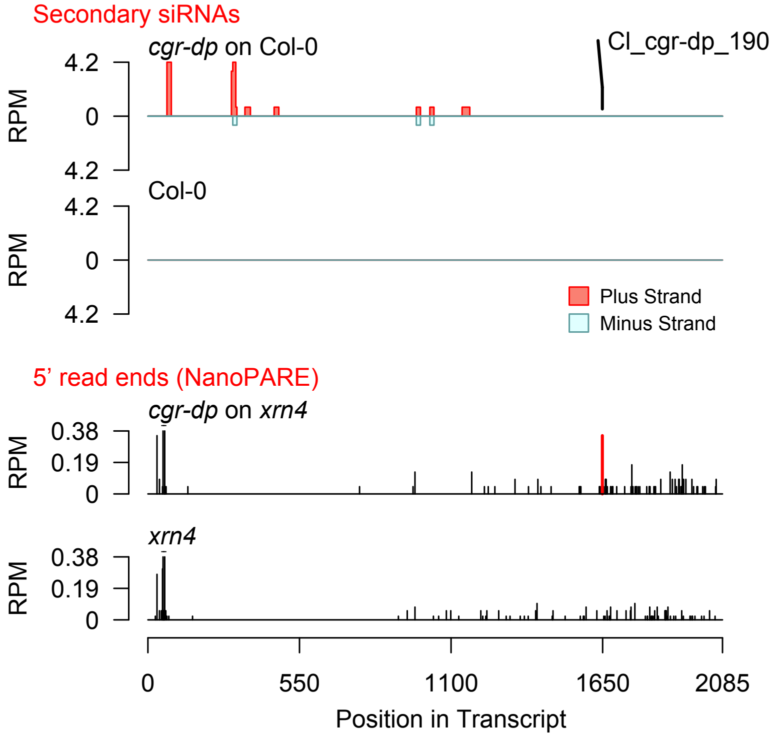

Diff. Exp. secondary siRNA locus not found

Allenscore: 5

Target Interaction:

```
5'- UUGAGAAAGAAUGGGAAGAAC   AT1G67230.1
     :  | | | | | | |   | | | | | | | | | |
3'- GUCUCUUUCAUACCCUUCUUC   Cl_cgr-dp_10674
```

Target Site: 1930

Superfamily: SupFam_3

miRNAin ccm: Yes

Published name: N/A

Confirmed Targeting

2nd-siRNA	*NanoPARE*
ccm	*ccm*
cpe-2015	*cpe-2015*
cpe-2017	
cgr-dp	*cgr-dp*
cgr-pm	
cgr-mass	
cin	*cin*

LINC1

AT1G67230 - Cl_cgr-dp_10674

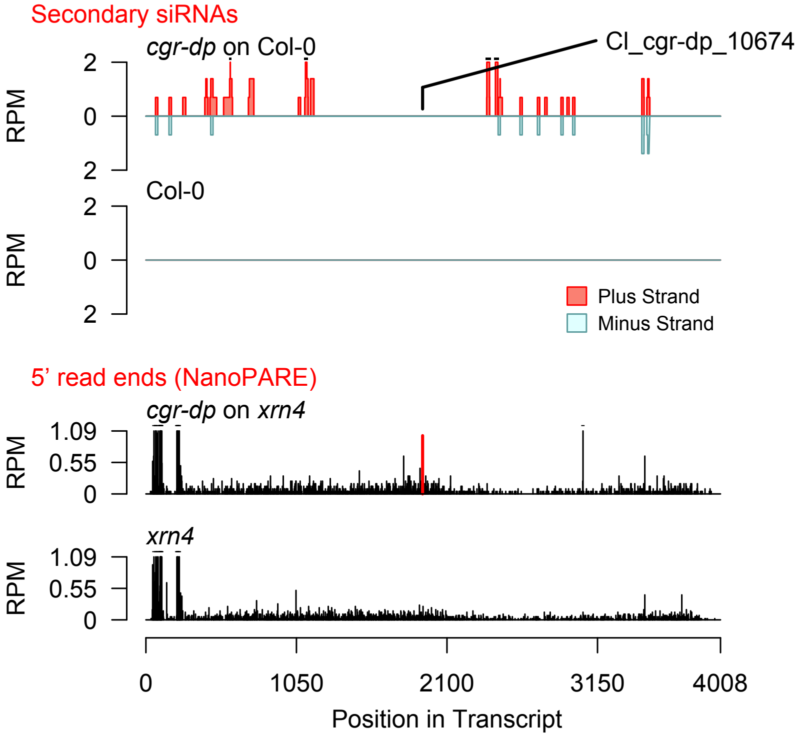

Diff. Exp. secondary siRNA locus not found

Allenscore: 6

Target Interaction:

```
5'- AACCGAAGAAGAUG-AAAGCUACA    AT3G51670.1
     |  ||||||||||||  ||||||  ||
3'- UGGGCUUCUUCUACUUUUCGA-GU    Cl_cgr-dp_279
```

Target Site: 1326

Superfamily: SupFam_37

miRNAin ccm: N/A

Published name: N/A

Confirmed Targeting

2nd-siRNA	*NanoPARE*
ccm	*ccm*
cpe-2015	*cpe-2015*
cpe-2017	
cgr-dp	*cgr-dp*
cgr-pm	
cgr-mass	
cin	*cin*

SEC14_cytosolic_factor_family_protein___

AT3G51670 - Cl_cgr-dp_279

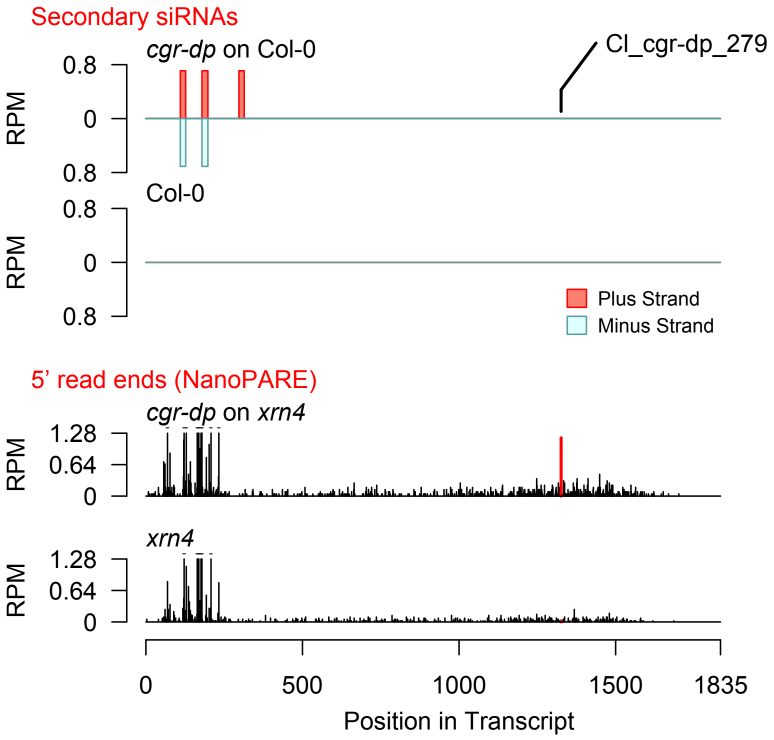

Diff. Exp. secondary siRNA locus not found

Allenscore: 5

Target Interaction:

```
5'- CUCACGUGACCUGCUUCUCCG   AT5G39610.1
        |||||  ||||||||||| :
3'- UUUUGCACGGGACGAAGAGGU   Cl_cgr-dp_26729
```

Target Site: 904

Superfamily: SupFam_187

miRNAin ccm: N/A

Published name: N/A

Confirmed Targeting

2nd-siRNA	*NanoPARE*
ccm	*ccm*
cpe-2015	*cpe-2015*
cpe-2017	
cgr-dp	*cgr-dp*
cgr-pm	
cgr-mass	
cin	*cin*

NAC6

AT5G39610 - Cl_cgr-dp_26729

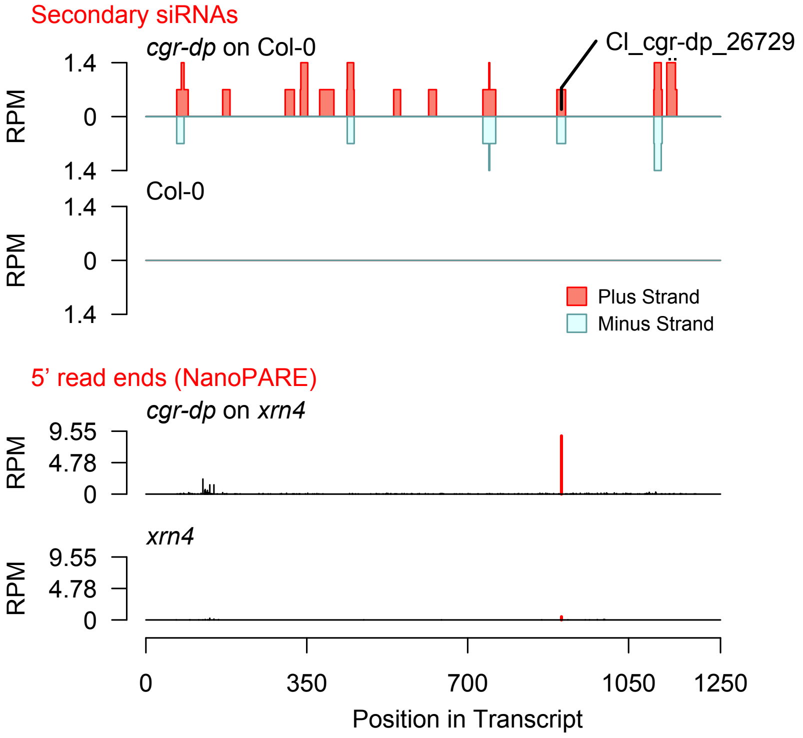

Diff. Exp. secondary siRNA locus not found

Allenscore: 4.5

Target Interaction:

```
5'- CGGGUUACGUUCCUCCAGAGUA    AT4G39400.1
      : | | : | | | : |   | | | | | | | | | | |
3'- AUCCGAUGUACGGAGGUCUCAU    CI_cgr-mass_20246
```

Target Site: 3530

Superfamily: SupFam_59

miRNAin ccm: N/A

Published name: N/A

Confirmed Targeting

2nd-siRNA	*NanoPARE*
ccm	*ccm*
cpe-2015	*cpe-2015*
cpe-2017	
cgr-dp	*cgr-dp*
cgr-pm	
cgr-mass	
cin	*cin*

BRI1

AT4G39400 - CI_cgr-mass_20246

Secondary siRNAs

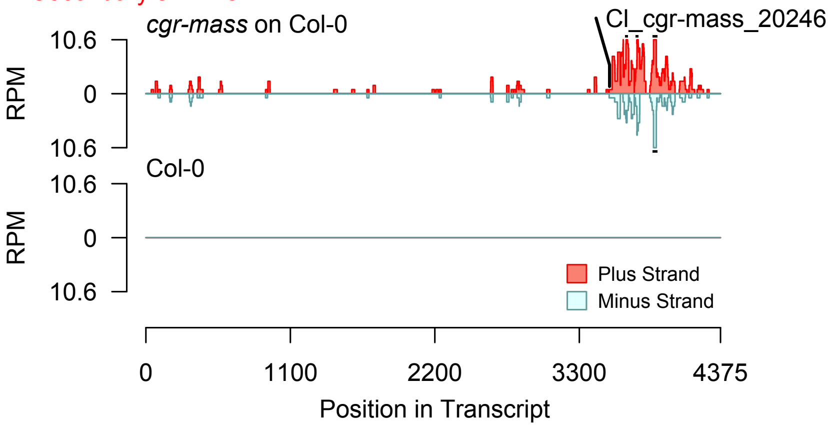

2nd siRNAs: Phase diagram

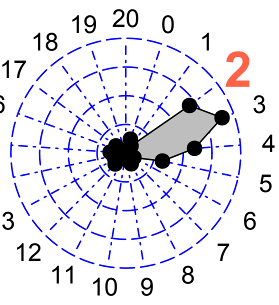

2nd siRNAs: Size distribution

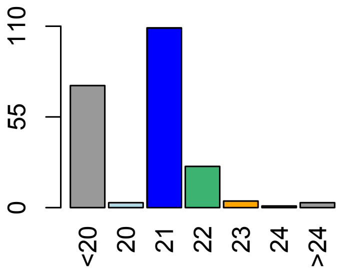

Degradome hits not found for sRNA

Allenscore: 3.5

Target Interaction:

```
5'- AAAGGGAAGUCAUCCUUGGCU   AT1G17590.1
       | | | : | | | | | | | | | | | | | | |
3'- AGUCCUUUCAGUAGGAACCGU   CI_cin_1673
```

Target Site: 1329

Superfamily: SupFam_1364

miRNAin ccm: N/A

Published name: N/A

Confirmed Targeting

2nd-siRNA	*NanoPARE*
ccm	*ccm*
cpe-2015	*cpe-2015*
cpe-2017	
cgr-dp	*cgr-dp*
cgr-pm	
cgr-mass	
cin	*cin*

NF-YA8

AT1G17590 - CI_cin_1673

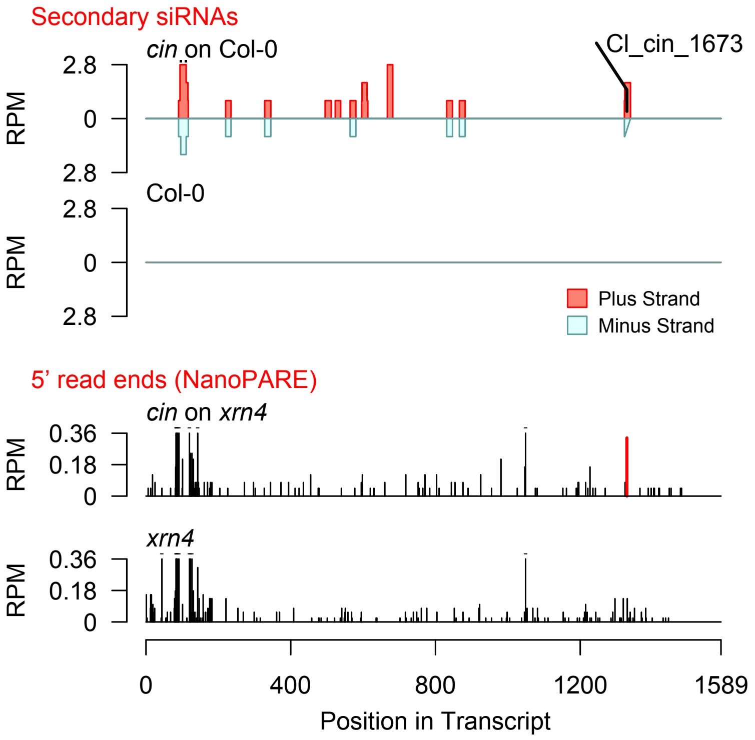

Diff. Exp. secondary siRNA locus not found

Allenscore: 3

Target Interaction:

5'- CAGGGAUACGCUGCUCCAGAAUA AT5G02290.1
||||| |||||||| ||||||||
3'- GUCCCAAUGCGACGUGGUCUUAU CI_ccm_818

Target Site: 1120

Superfamily: SupFam_124

miRNAin ccm: No

Published name: Not published

Confirmed Targeting

2nd-siRNA	*NanoPARE*
ccm	*ccm*
cpe-2015	*cpe-2015*
cpe-2017	
cgr-dp	*cgr-dp*
cgr-pm	
cgr-mass	
cin	*cin*

NAK

AT5G02290 - CI_ccm_818

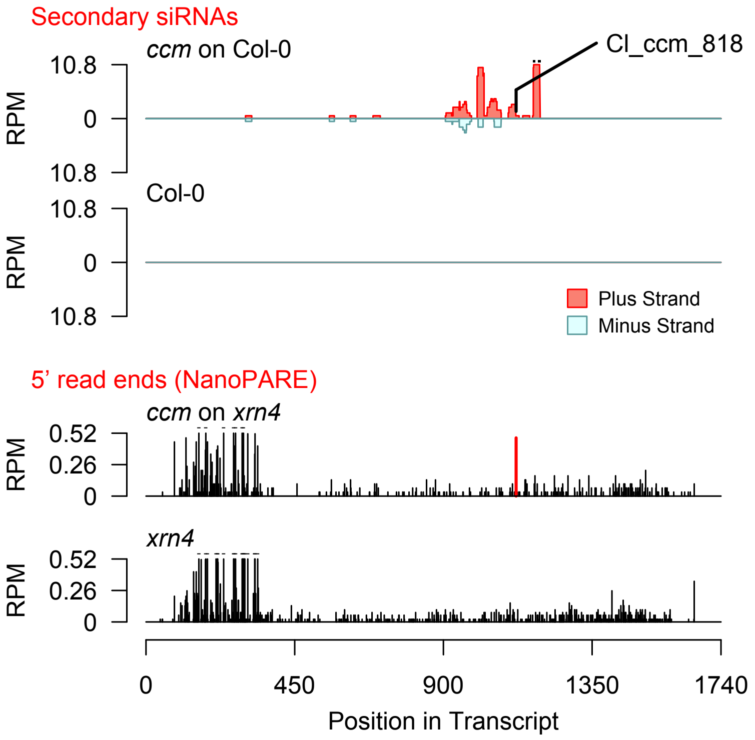

Diff. Exp. secondary siRNA locus not found

Allenscore: 4.5

Target Interaction:

```
5'- CCGUUUCUACUCCACCAUCACA    AT4G28300.1
       | | | | | : |   | | | | | | | : | | | |
3'- UUCAAAGGUAAGGUGGUGGUGU    CI_cgr-dp_307
```

Target Site: 990

Superfamily: SupFam_897

miRNAin ccm: N/A

Published name: N/A

Confirmed Targeting

2nd-siRNA	*NanoPARE*
ccm	*ccm*
cpe-2015	*cpe-2015*
cpe-2017	
cgr-dp	*cgr-dp*
cgr-pm	
cgr-mass	
cin	*cin*

DUF1421_containing_protein

AT4G28300 - CI_cgr-dp_307

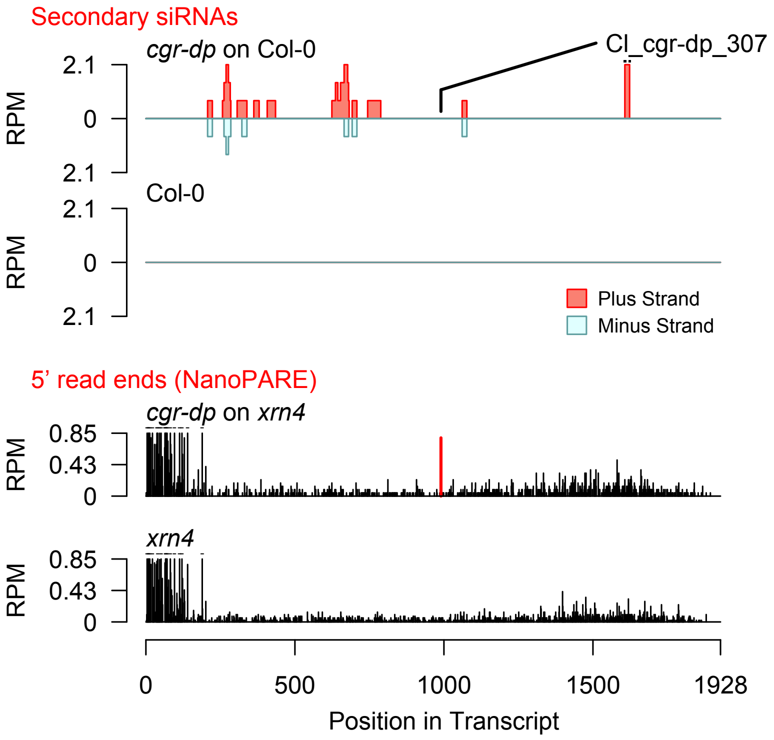

Diff. Exp. secondary siRNA locus not found

Allenscore: 3.5

Target Interaction:

```
5'- GAGAUGUUGAGAUUGAAGUUG   AT5G07350.1
     : | | | | | | | | | | | | | | | | | |     |
3'- UUCUACAACUCUAACUUCAUC   CI_ccm_935
```

Target Site: 2216

Superfamily: SupFam_287

miRNAin ccm: Yes

Published name: ccm-MIR12464*

Confirmed Targeting

2nd-siRNA	*NanoPARE*
ccm	*ccm*
cpe-2015	*cpe-2015*
cpe-2017	
cgr-dp	*cgr-dp*
cgr-pm	
cgr-mass	
cin	*cin*

Tudor1

AT5G07350 - CI_ccm_935

Secondary siRNAs

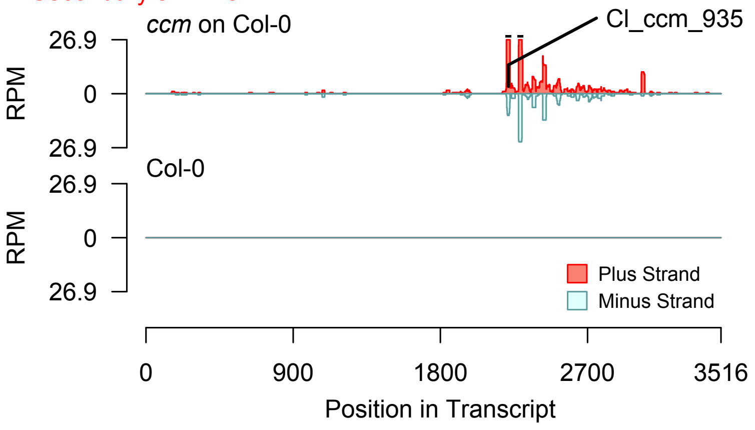

2nd siRNAs: Phase diagram

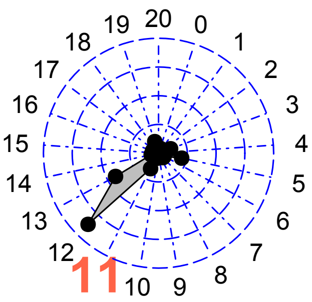

2nd siRNAs: Size distribution

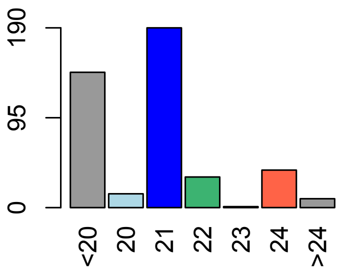

Degradome hits not found for sRNA

Allenscore: 3.5

Target Interaction:

```
5'- GAGAUGUUGAGAUUGAAGUUG   AT5G07350.1
     :|||||||||||||||||||   |
3'- UUCUACAACUCUAACUUCAUC   Cl_cpe-2015_358
```

Target Site: 2216

Superfamily: SupFam_287

miRNAin ccm: Yes

Published name: N/A

Confirmed Targeting

2nd-siRNA	*NanoPARE*
ccm	*ccm*
cpe-2015	*cpe-2015*
cpe-2017	
cgr-dp	*cgr-dp*
cgr-pm	
cgr-mass	
cin	*cin*

Tudor1

AT5G07350 - Cl_cpe-2015_358

Secondary siRNAs

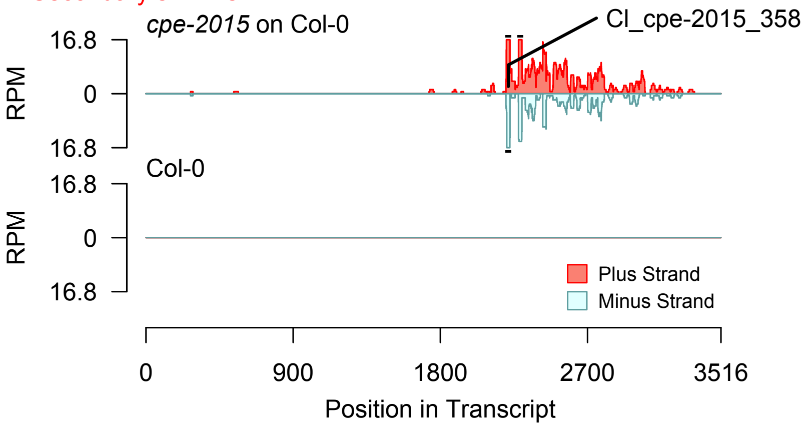

2nd siRNAs: Phase diagram

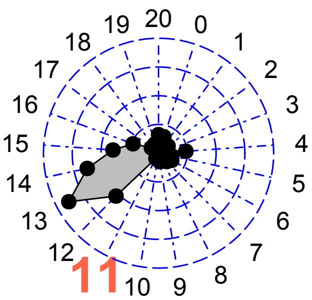

2nd siRNAs: Size distribution

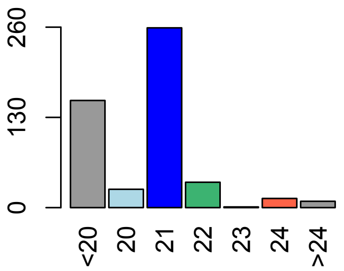

Degradome hits not found for sRNA

Allenscore: 3.5

Target Interaction:

```
5'- GAGAUGUUGAGAUUGAAGUUG   AT5G07350.1
     : | | | | | | | | | | | | | | | | | |    |
3'- UUCUACAACUCUAACUUCAUC   CI_cpe-2017_269
```

Target Site: 2216

Superfamily: SupFam_287

miRNAin ccm: Yes

Published name: N/A

Confirmed Targeting

2nd-siRNA	*NanoPARE*
ccm	*ccm*
cpe-2015	*cpe-2015*
cpe-2017	
cgr-dp	*cgr-dp*
cgr-pm	
cgr-mass	
cin	*cin*

Tudor1

AT5G07350 - CI_cpe-2017_269

Secondary siRNAs

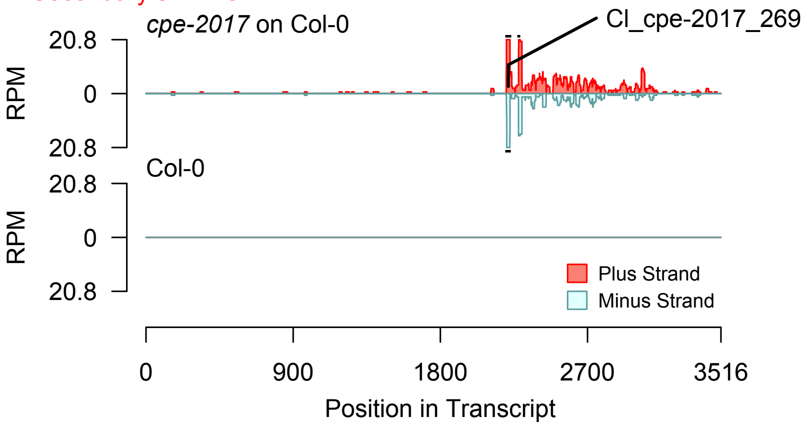

2nd siRNAs: Phase diagram

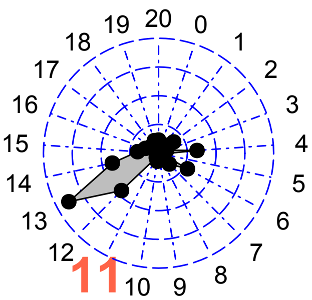

2nd siRNAs: Size distribution

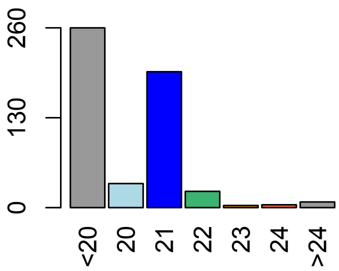

Degradome hits not found for sRNA

Allenscore: 1.5

Target Interaction:

```
5'- GCGGCAAUUCAUUCUUGGCUU   AT3G20910.1
     : | | | | | | | | | | | | : | | | | | | | |
3'- UGCCGUUAAGUAGGAACCGAA   CI_ccm_2887
```

Target Site: 1073

Superfamily: SupFam_72

miRNAin ccm: Yes

Published name: Not published

Confirmed Targeting

2nd-siRNA	*NanoPARE*
ccm	*ccm*
cpe-2015	*cpe-2015*
cpe-2017	
cgr-dp	*cgr-dp*
cgr-pm	
cgr-mass	
cin	*cin*

NF-YA9

AT3G20910 - CI_ccm_2887

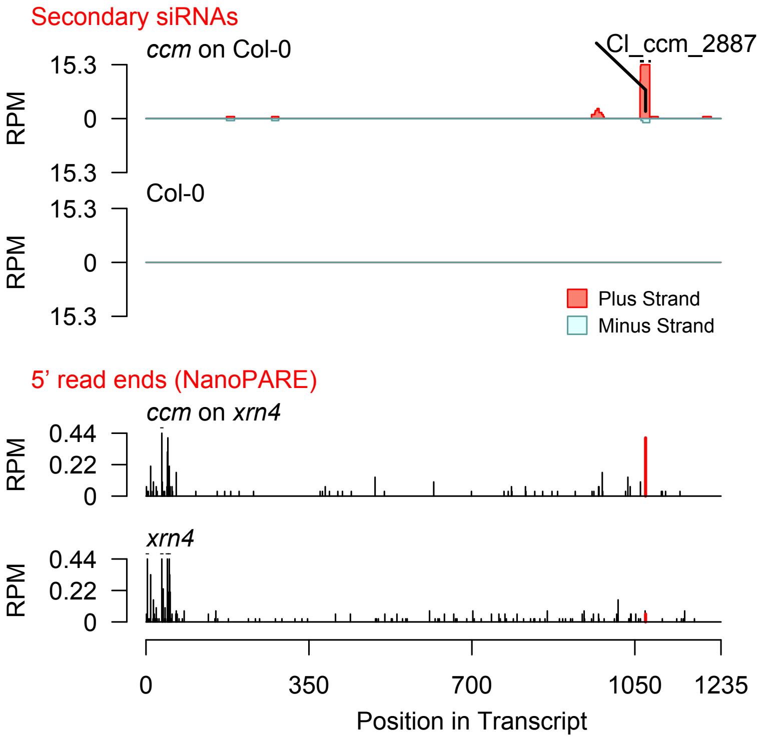

Diff. Exp. secondary siRNA locus not found

Allenscore: 4

Target Interaction:

```
5'- GGCGGCAAUUCAUUCUUGGCUU   AT3G20910.1
    : : | | | | | |   | | | | : | | | | | | |
3'- UUGCCGUUUAGUAGGAACCGAU   CI_ccm_2720
```

Target Site: 1073

Superfamily: SupFam_72

miRNAin ccm: Yes

Published name: Not published

Confirmed Targeting

2nd-siRNA	*NanoPARE*
ccm	*ccm*
cpe-2015	*cpe-2015*
cpe-2017	
cgr-dp	*cgr-dp*
cgr-pm	
cgr-mass	
cin	*cin*

NF-YA9

AT3G20910 - CI_ccm_2720

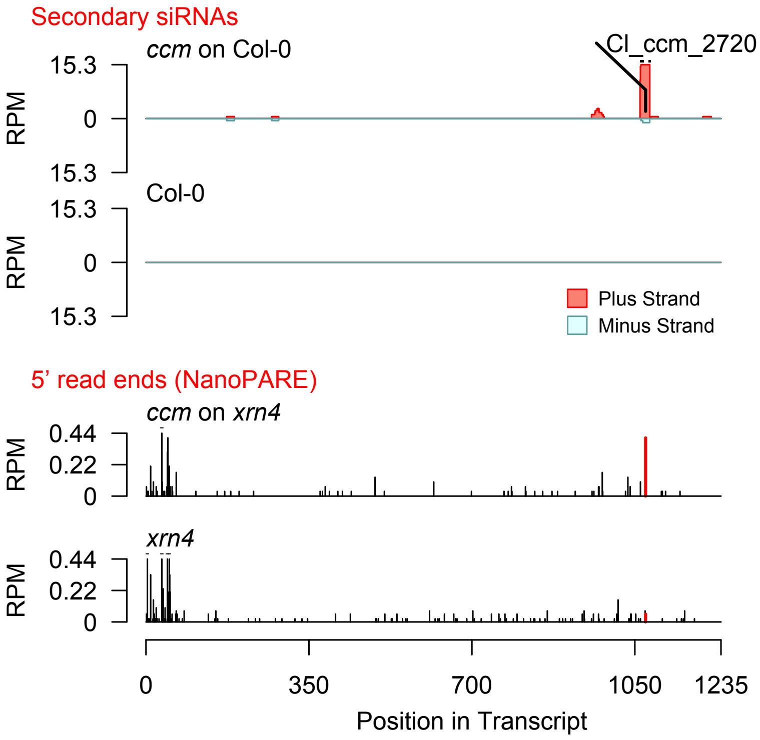

Diff. Exp. secondary siRNA locus not found

Allenscore: 6

Target Interaction:

```
5'- UCAAGAACGCUACGAGGAACUUUC    AT2G02800.1
       | | : | | | | | | : | | | | | : | | | | | | :
3'- UUUUUUUGCGGUGCUCUUUGAAGU    Cl_cgr-dp_62946
```

Target Site: 582

Superfamily: SupFam_26

miRNAin ccm: Yes

Published name: N/A

Confirmed Targeting

2nd-siRNA	*NanoPARE*
ccm	*ccm*
cpe-2015	*cpe-2015*
cpe-2017	
cgr-dp	*cgr-dp*
cgr-pm	
cgr-mass	
cin	*cin*

APK2B

AT2G02800 - Cl_cgr-dp_62946

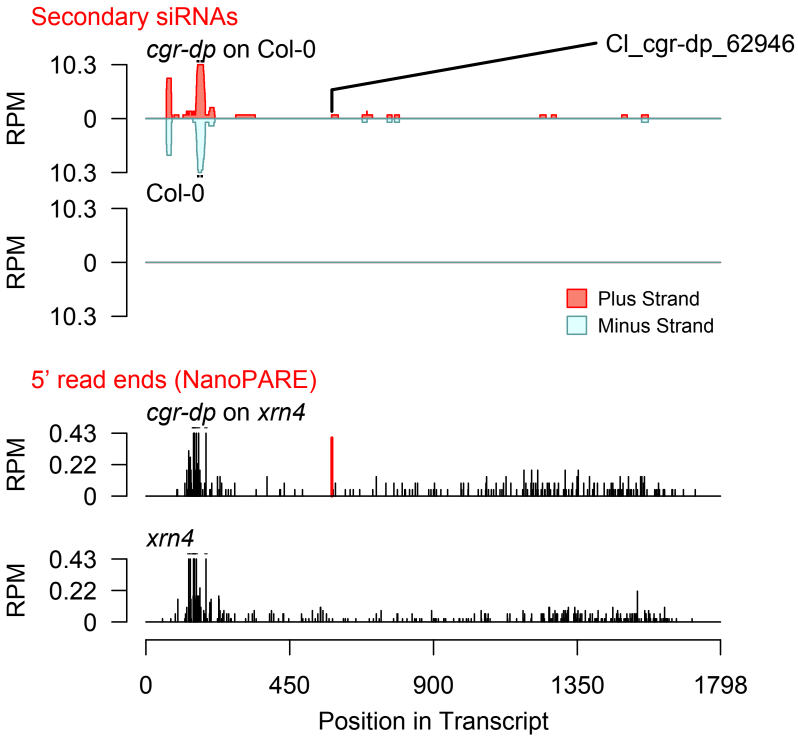

Diff. Exp. secondary siRNA locus not found

Allenscore: 4

Target Interaction:

```
5'- AGAACGCUACGAGGAACUUUC    AT2G02800.1
     | : | | | | | : | | | | | : | | | | | :
3'- UUUUGCGGUGCUCUUUGAAGU    Cl_cgr-dp_29
```

Target Site: 582

Superfamily: SupFam_26

miRNAin ccm: Yes

Published name: N/A

Confirmed Targeting

2nd-siRNA	*NanoPARE*
ccm	*ccm*
cpe-2015	*cpe-2015*
cpe-2017	
cgr-dp	*cgr-dp*
cgr-pm	
cgr-mass	
cin	*cin*

APK2B

AT2G02800 - Cl_cgr-dp_29

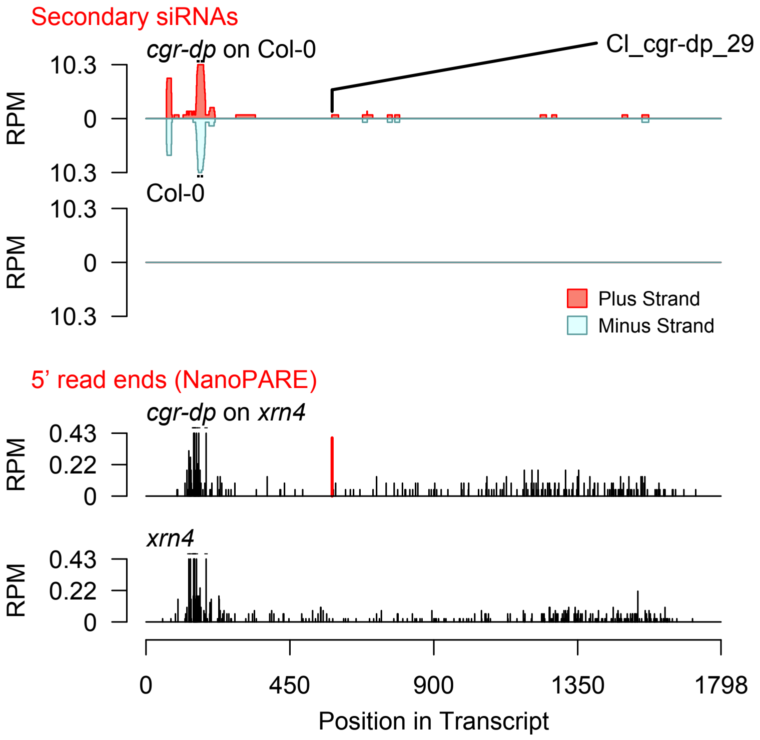

Diff. Exp. secondary siRNA locus not found

Allenscore: 3.5

Target Interaction:

```
5'- AGAGAGGUGGAGAUUCUUGA   AT3G14280.1
     ||||:|||||:||:|||||
3'- GCUCUUCACCUUUAGGAACU   CI_ccm_1979
```

Target Site: 470

Superfamily: SupFam_20

miRNAin ccm: Yes

Published name: Not published

Confirmed Targeting

2nd-siRNA	*NanoPARE*
ccm	*ccm*
cpe-2015	*cpe-2015*
cpe-2017	
cgr-dp	*cgr-dp*
cgr-pm	
cgr-mass	
cin	*cin*

LL-diaminopimelate_aminotransferase

AT3G14280 - CI_ccm_1979

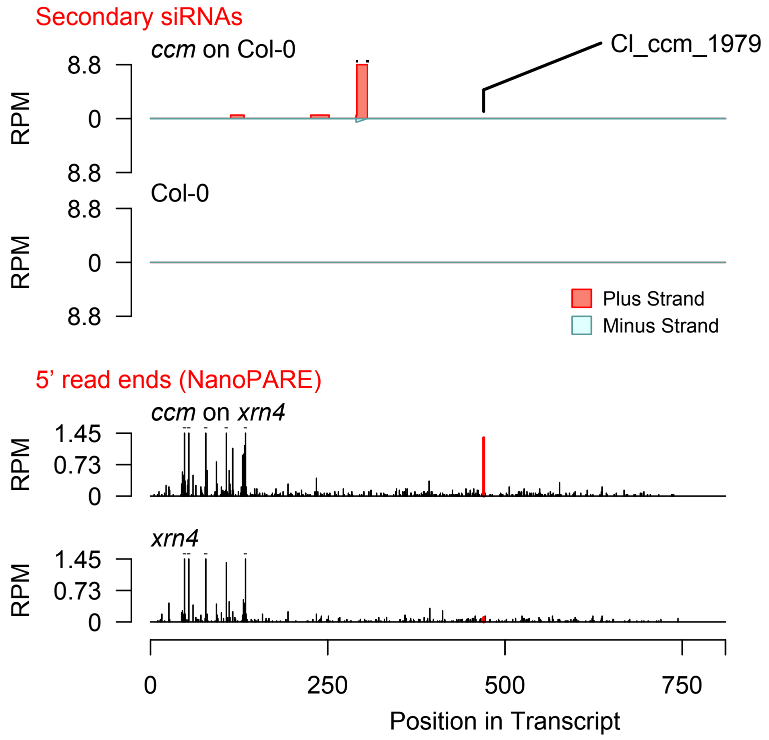

Diff. Exp. secondary siRNA locus not found

Allenscore: 3

Target Interaction:

```
5'- CUCGCAU - UCUUGAAAGAUUC    AT2G25170.1
      | | | | | |   | | | | | | | | | | | |
3'- AAGCGUAUAGAACUUUCUAAU    Cl_cgr-dp_80
```

Target Site: 3195

Superfamily: SupFam_206

miRNAin ccm: N/A

Published name: N/A

Confirmed Targeting

2nd-siRNA	*NanoPARE*
ccm	*ccm*
cpe-2015	*cpe-2015*
cpe-2017	
cgr-dp	*cgr-dp*
cgr-pm	
cgr-mass	
cin	*cin*

PKL

AT2G25170 - Cl_cgr-dp_80

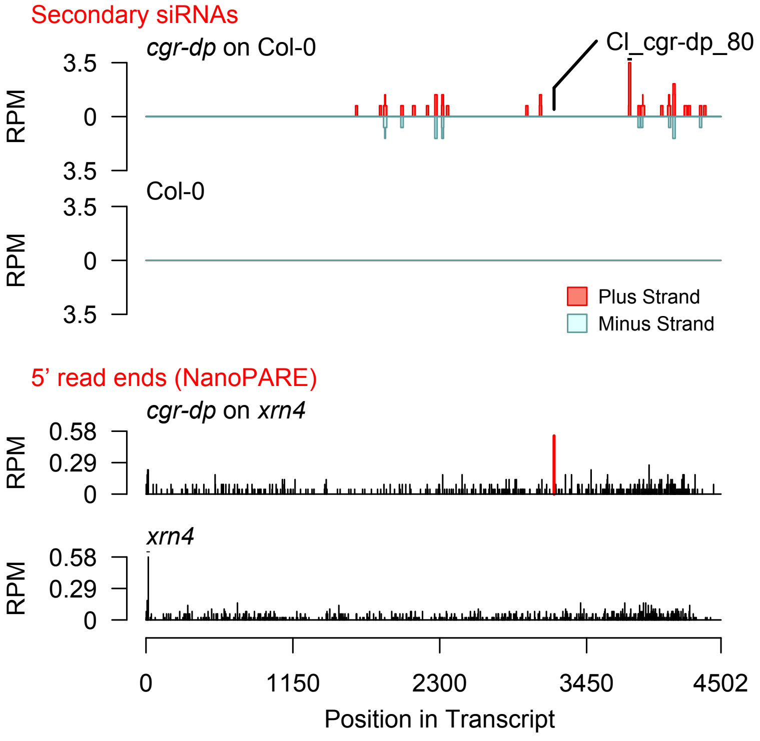

Diff. Exp. secondary siRNA locus not found

Allenscore: 4.5

Target Interaction:

```
5'- UCCAAACCGACACACACGGCAA    AT2G36570.1
    | | | | | | | | : | | | | | | | |    | | |
3'- AGGUUUGGUUGUGUGUGG-GUU    CI_cgr-pm_525
```

Target Site: 408

Superfamily: SupFam_161

miRNAin ccm: N/A

Published name: N/A

Confirmed Targeting

2nd-siRNA	*NanoPARE*
ccm	*ccm*
cpe-2015	*cpe-2015*
cpe-2017	
cgr-dp	*cgr-dp*
cgr-pm	
cgr-mass	
cin	*cin*

PXC1

AT2G36570 - CI_cgr-pm_525

Secondary siRNAs

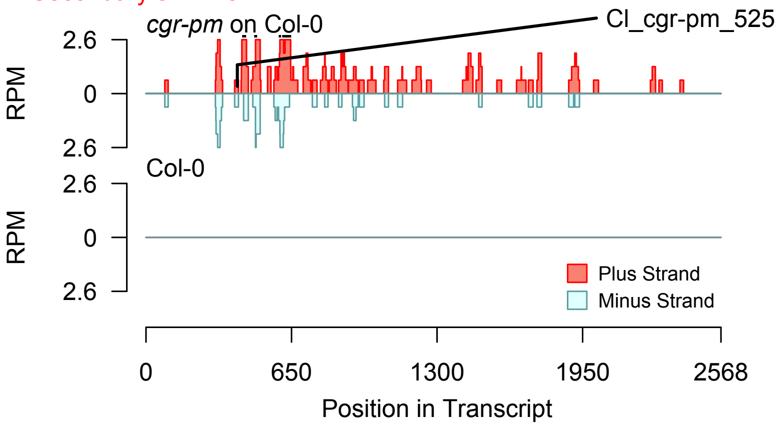

2nd siRNAs: Phase diagram

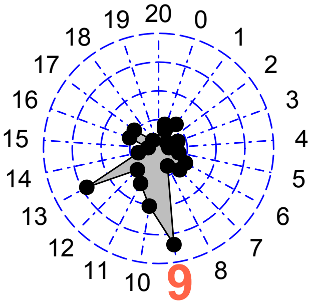

2nd siRNAs: Size distribution

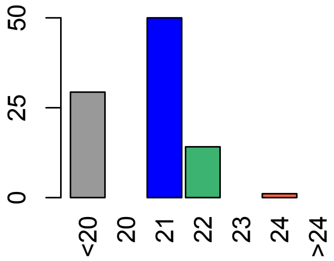

Degradome hits not found for sRNA

Allenscore: 4.5

Target Interaction:

```
5'- GCAGGGCAGACAUCUGAGAGGA    AT3G06380.1
     | | | | | | | : | | | |   : | | | | | | |
3'- AGUCCCGUUUGUAAGCUCUCCU    CI_cgr-dp_8594
```

Target Site: 1320

Superfamily: SupFam_14

miRNAin ccm: N/A

Published name: N/A

Confirmed Targeting

2nd-siRNA	*NanoPARE*
ccm	*ccm*
cpe-2015	*cpe-2015*
cpe-2017	
cgr-dp	*cgr-dp*
cgr-pm	
cgr-mass	
cin	*cin*

TLP9

AT3G06380 - CI_cgr-dp_8594

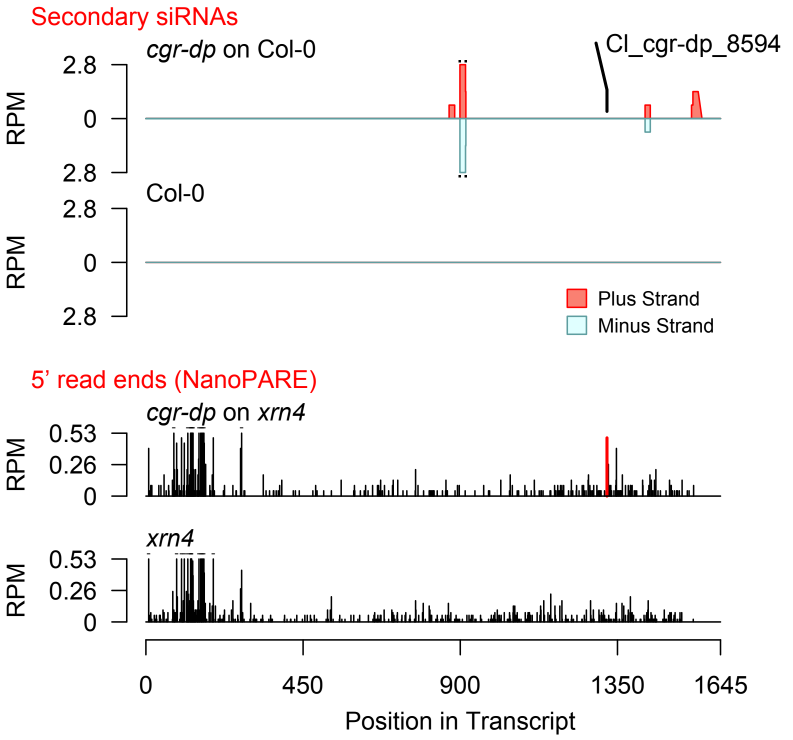

Diff. Exp. secondary siRNA
locus not found

Allenscore: 5

Target Interaction:

```
5'- UGGCUCUUAACUUCUCAGUGU   AT5G65430.1
     | : | |   | | : | | | | | | | | | | |
3'- AUCGUGAGUUGAAGAGUCAGU   CI_cpe-2015_129
```

Target Site: 885

Superfamily: SupFam_24

miRNAin ccm: Yes

Published name: N/A

Confirmed Targeting

2nd-siRNA	*NanoPARE*
ccm	*ccm*
cpe-2015	*cpe-2015*
cpe-2017	
cgr-dp	*cgr-dp*
cgr-pm	
cgr-mass	
cin	*cin*

GRF8

AT5G65430 - CI_cpe-2015_129

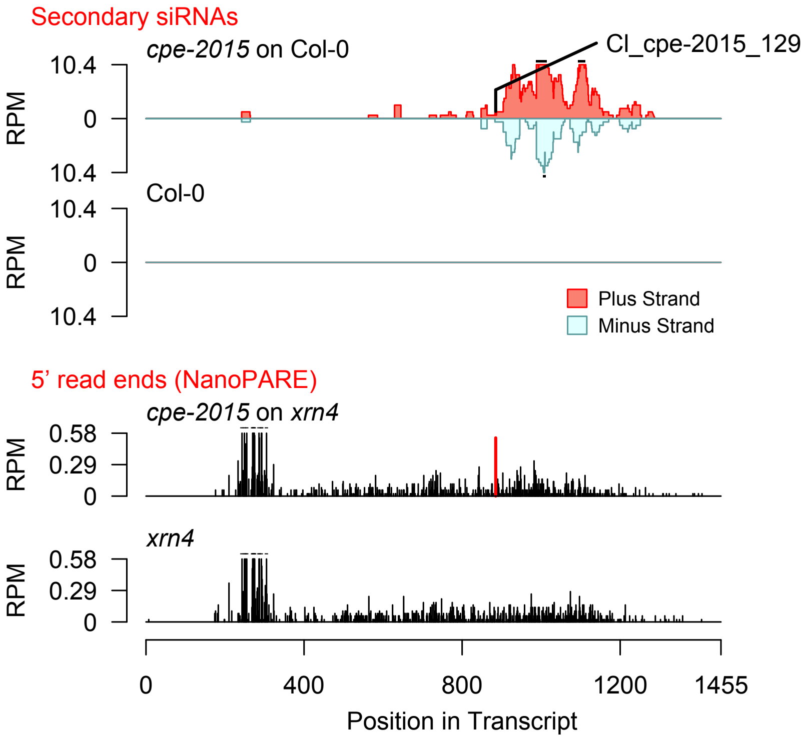

Diff. Exp. secondary siRNA locus not found

Allenscore: 4.5

Target Interaction:

```
5'- GAGGACUUCAUUACCUUCACA   AT5G24010.1
     : | | | | | | | |     | | | | | | | | | |
3'- UUCCUGAAGAUAUGGAAGUGU   CI_cgr-dp_14631
```

Target Site: 1954

Superfamily: SupFam_49

miRNAin ccm: Yes

Published name: N/A

Confirmed Targeting

2nd-siRNA	*NanoPARE*
ccm	*ccm*
cpe-2015	*cpe-2015*
cpe-2017	
cgr-dp	*cgr-dp*
cgr-pm	
cgr-mass	
cin	*cin*

Protein_kinase_superfamily_protein

AT5G24010 - CI_cgr-dp_14631

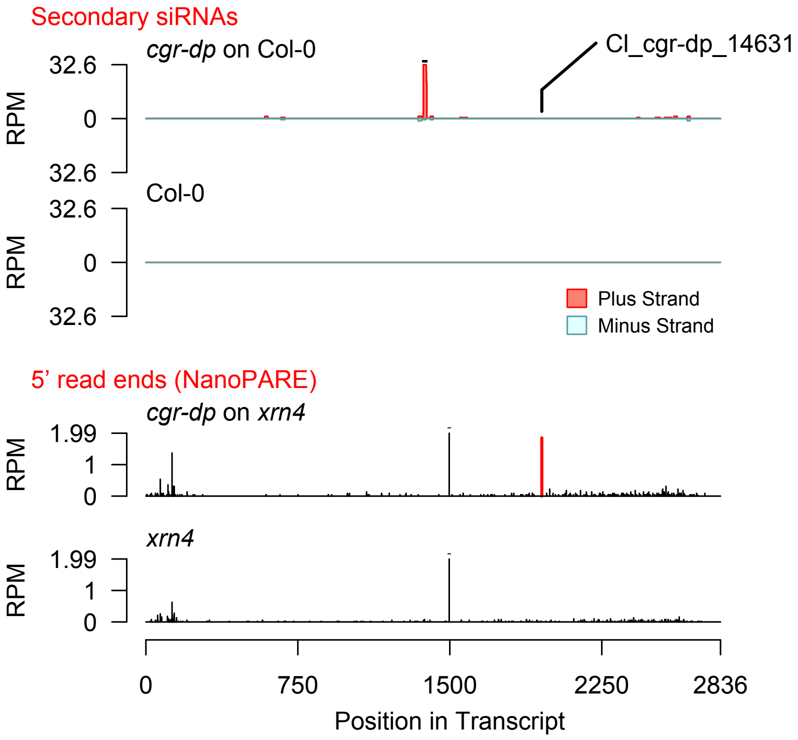

Diff. Exp. secondary siRNA locus not found

Allenscore: 4.5

Target Interaction:

```
5'- CUGGUGUGCAAGUCAUGGUACG   AT3G62980.1
     ||  ||  |:|||:|||||||||:
3'- GAGCAGAUGUUUAGUACCAUGU   Cl_ccm_2
```

Target Site: 555

Superfamily: SupFam_27

miRNAin ccm: Yes

Published name: ccm-MIR12497a*

Confirmed Targeting

2nd-siRNA	*NanoPARE*
ccm	*ccm*
cpe-2015	*cpe-2015*
cpe-2017	
cgr-dp	*cgr-dp*
cgr-pm	
cgr-mass	
cin	*cin*

TIR1

AT3G62980 - Cl_ccm_2

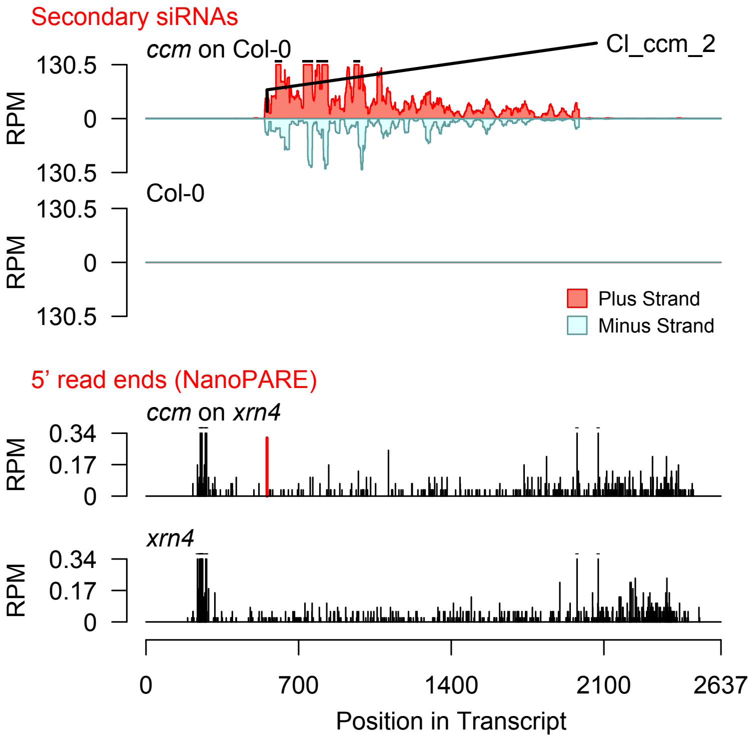

2nd siRNAs: Phase diagram

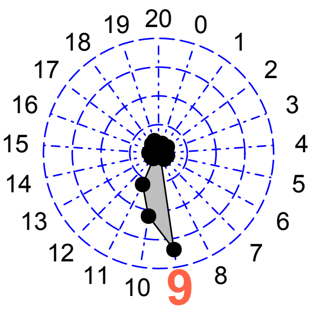

2nd siRNAs: Size distribution

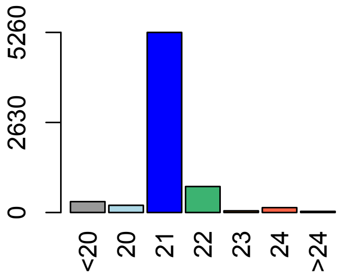

Allenscore: 2.5

Target Interaction:

```
5'- CUGGUGUGCAAGUCAUGGUACG   AT3G62980.1
     | : | | | | | | | | | | | | | | | | | | | :
3'- AAUCACACGUUCAGUACCAUGU   CI_ccm_438
```

Target Site: 555

Superfamily: SupFam_27

miRNAin ccm: Yes

Published name: Not published

Confirmed Targeting

2nd-siRNA	*NanoPARE*
ccm	*ccm*
cpe-2015	*cpe-2015*
cpe-2017	
cgr-dp	*cgr-dp*
cgr-pm	
cgr-mass	
cin	*cin*

TIR1

AT3G62980 - CI_ccm_438

Secondary siRNAs

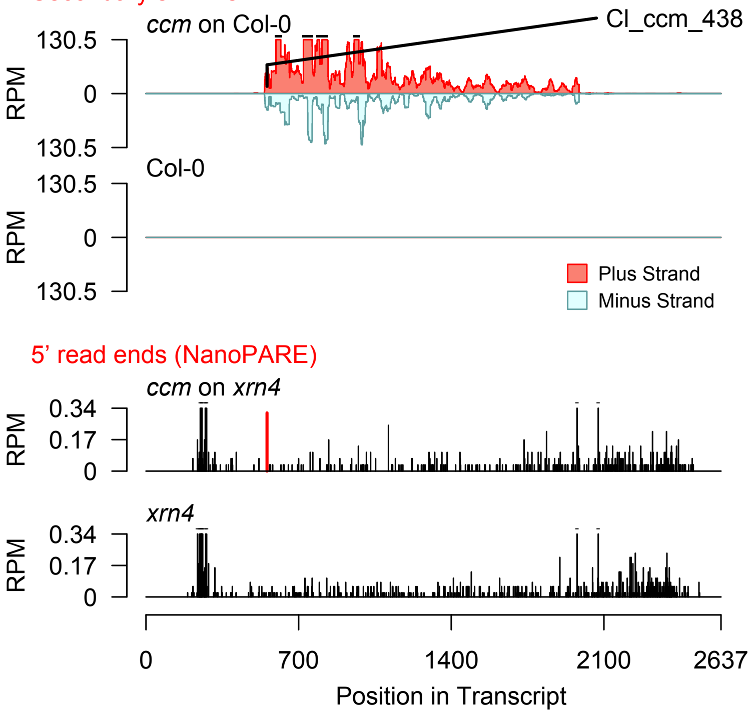

2nd siRNAs: Phase diagram

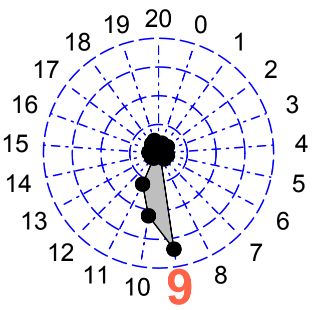

2nd siRNAs: Size distribution

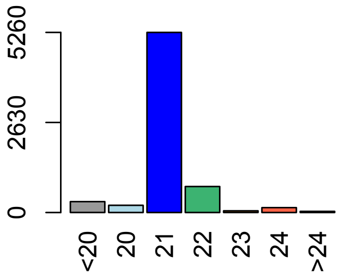

Allenscore: 3.5

Target Interaction:

```
5'- CUGGUGUGCAAGUCAUGGUACG   AT3G62980.1
     | | | | : | | | | | : | | | | | | | | | :
3'- AACCAUACGUUUAGUACCAUGU   Cl_ccm_614
```

Target Site: 555

Superfamily: SupFam_27

miRNAin ccm: Yes

Published name: ccm-MIR12497b

Confirmed Targeting

2nd-siRNA	*NanoPARE*
ccm	*ccm*
cpe-2015	*cpe-2015*
cpe-2017	
cgr-dp	*cgr-dp*
cgr-pm	
cgr-mass	
cin	*cin*

TIR1

AT3G62980 - Cl_ccm_614

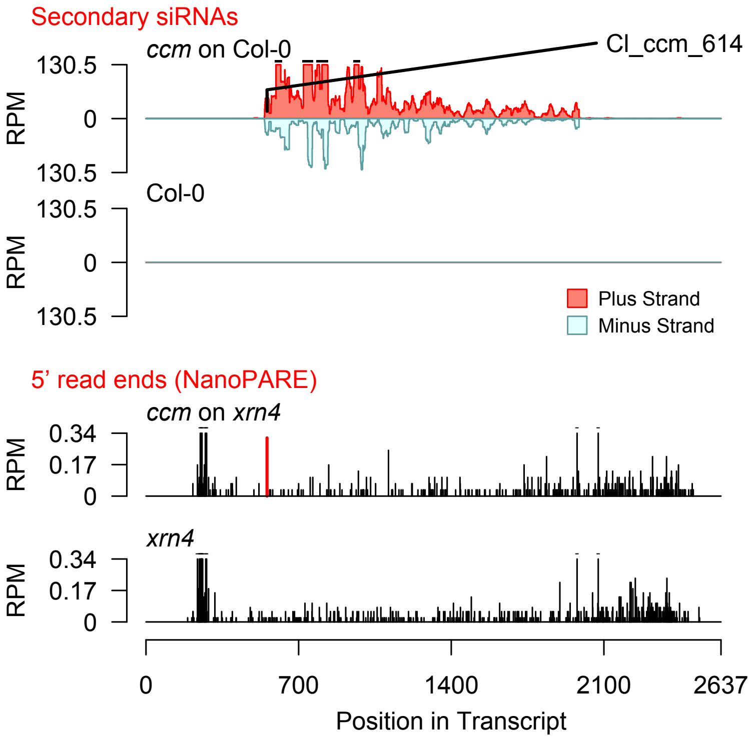

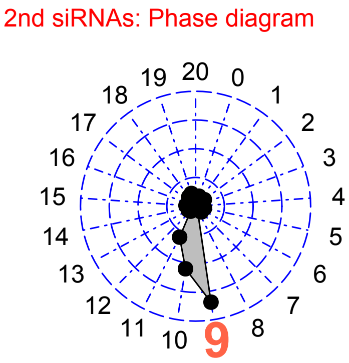

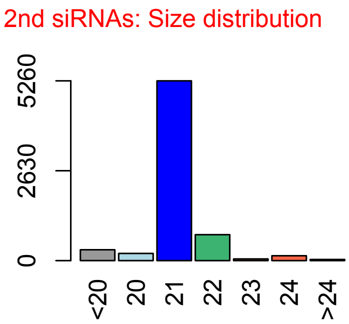

Allenscore: 4

Target Interaction:

```
5'- CUGGUGUGCAAGUCAUGGUACG    AT3G62980.1
     | | | | | | | | | | | : | | | | | | | |     :
3'- GACCACACGUUUAGUACCAUAU    CI_ccm_13108
```

Target Site: 555

Superfamily: SupFam_27

miRNAin ccm: Yes

Published name: Not published

Confirmed Targeting

2nd-siRNA	*NanoPARE*
ccm	*ccm*
cpe-2015	*cpe-2015*
cpe-2017	
cgr-dp	*cgr-dp*
cgr-pm	
cgr-mass	
cin	*cin*

TIR1

AT3G62980 - CI_ccm_13108

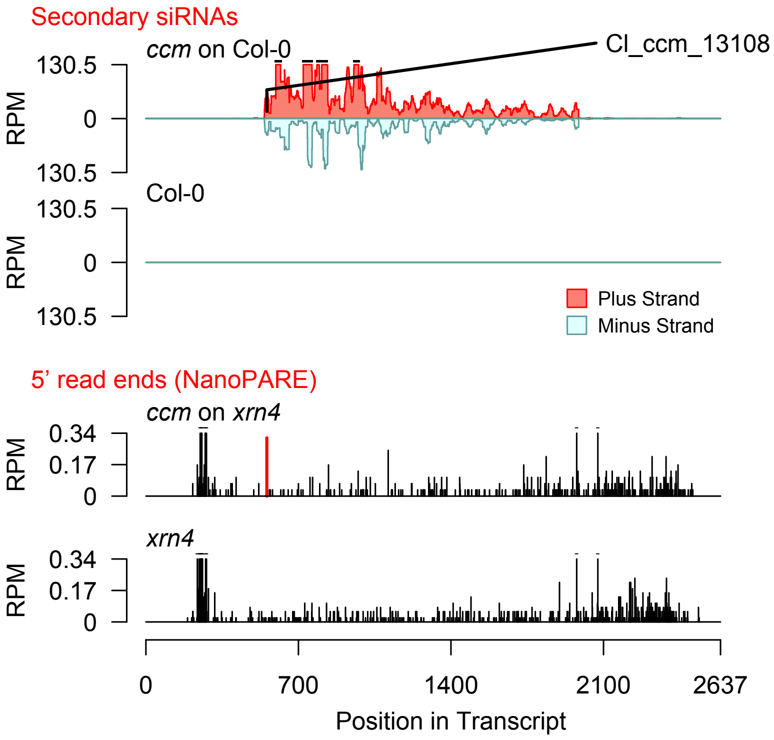

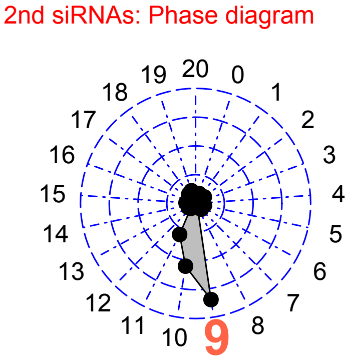

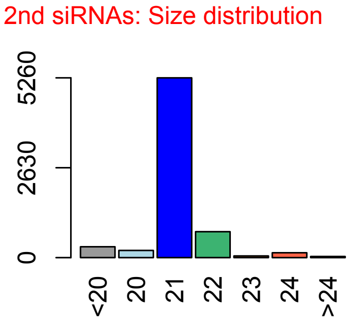

Allenscore: 1

Target Interaction:

```
5'- GGUGUGCAAGUCAUGGUACG   AT3G62980.1
     |||||||||||||||||||:
3'- CCACACGUUCAGUACCAUGU   Cl_ccm_51538
```

Target Site: 555

Superfamily: SupFam_27

miRNAin ccm: Yes

Published name: Not published

Confirmed Targeting

2nd-siRNA	*NanoPARE*
ccm	*ccm*
cpe-2015	*cpe-2015*
cpe-2017	
cgr-dp	*cgr-dp*
cgr-pm	
cgr-mass	
cin	*cin*

TIR1

AT3G62980 - Cl_ccm_51538

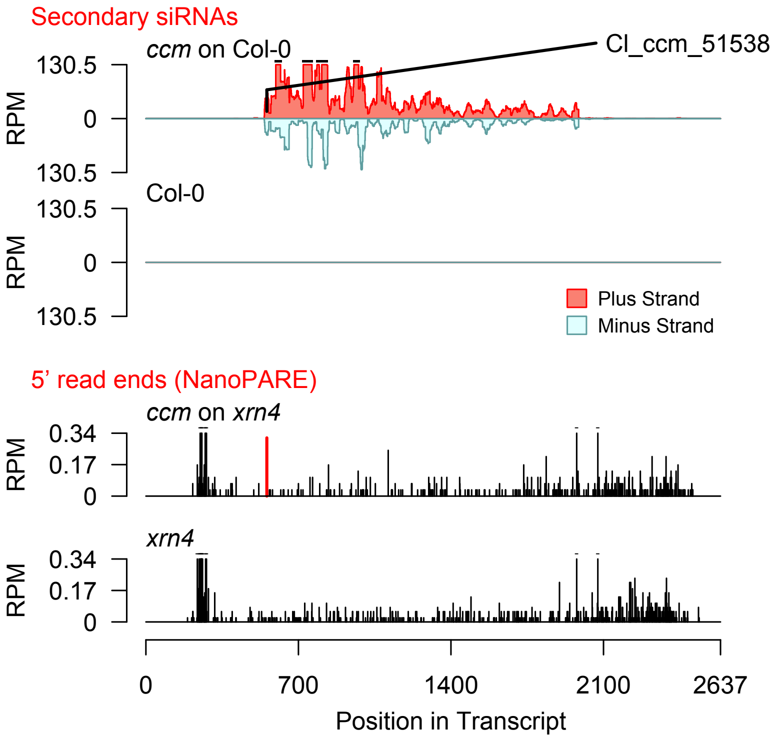

2nd siRNAs: Phase diagram

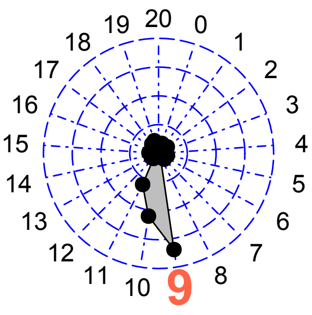

2nd siRNAs: Size distribution

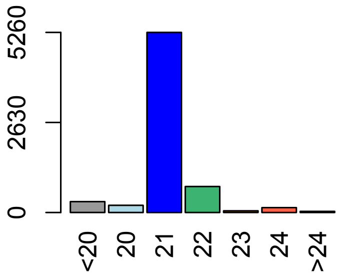

Allenscore: 4

Target Interaction:

```
5'- CUGGUGUGCAAGUCAUGGUACG    AT3G62980.1
     ||  ||  |||||:|||||||||:
3'- GAGCAGACGUUUAGUACCAUGU    Cl_cpe-2015_7
```

Target Site: 555

Superfamily: SupFam_27

miRNAin ccm: Yes

Published name: N/A

Confirmed Targeting

2nd-siRNA	*NanoPARE*
ccm	*ccm*
cpe-2015	*cpe-2015*
cpe-2017	
cgr-dp	*cgr-dp*
cgr-pm	
cgr-mass	
cin	*cin*

TIR1

AT3G62980 - Cl_cpe-2015_7

Secondary siRNAs

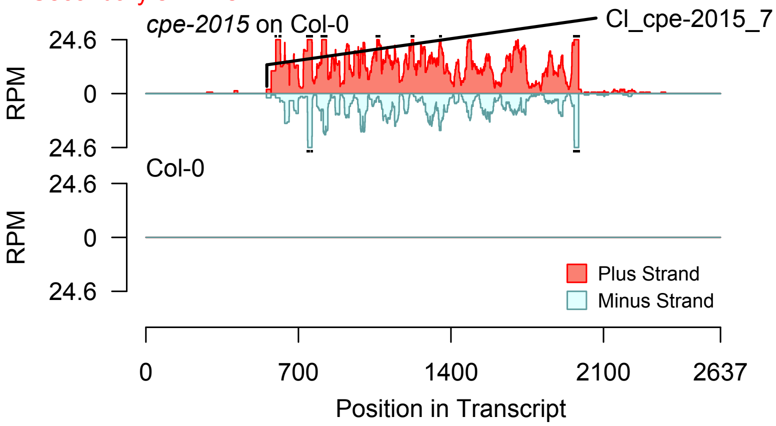

2nd siRNAs: Phase diagram

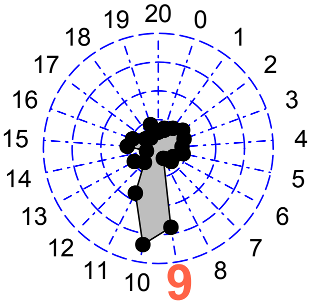

2nd siRNAs: Size distribution

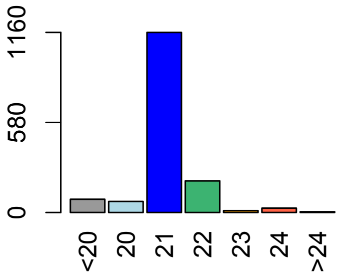

Degradome hits not found for sRNA

Allenscore: 4

Target Interaction:

```
5'- CUGGUGUGCAAGUCAUGGUACG    AT3G62980.1
     | |   | |   | | | | | : | | | | | | | | | :
3'- GAGCAGACGUUUAGUACCAUGU    Cl_cpe-2017_11
```

Target Site: 555

Superfamily: SupFam_27

miRNAin ccm: Yes

Published name: N/A

Confirmed Targeting

2nd-siRNA	*NanoPARE*
ccm	*ccm*
cpe-2015	*cpe-2015*
cpe-2017	
cgr-dp	*cgr-dp*
cgr-pm	
cgr-mass	
cin	*cin*

TIR1

AT3G62980 - Cl_cpe-2017_11

Secondary siRNAs

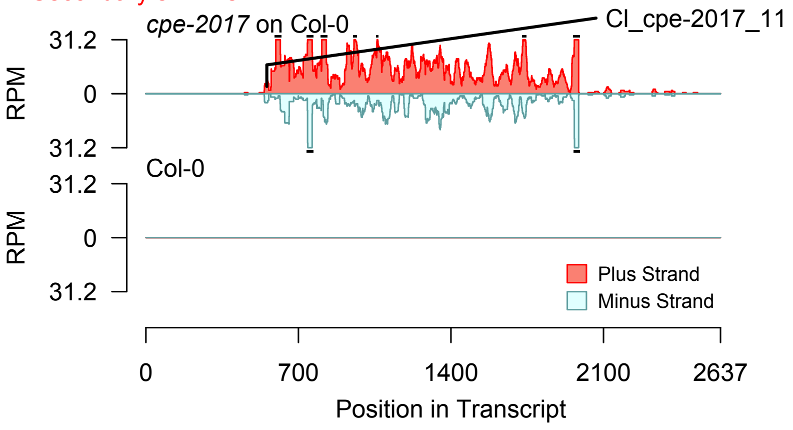

2nd siRNAs: Phase diagram

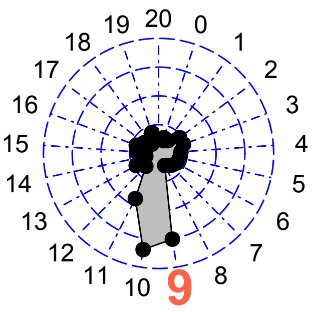

2nd siRNAs: Size distribution

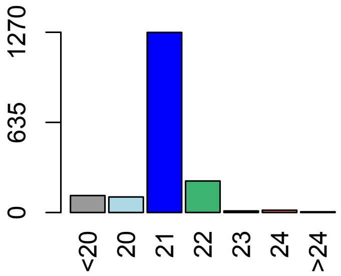

Degradome hits not found for sRNA

Allenscore: 5.5

Target Interaction:

```
5'- UGGUGAAAAGUGGAAAUCCUUGU    AT1G20696.1
      :   : | |   | | | | | | | | | | | | | | |
3'- UUAGCUCUUCACCUUUAGGAACU    CI_ccm_4973
```

Target Site: 572

Superfamily: SupFam_20

miRNAin ccm: Yes

Published name: Not published

Confirmed Targeting

2nd-siRNA	*NanoPARE*
ccm	*ccm*
cpe-2015	*cpe-2015*
cpe-2017	
cgr-dp	*cgr-dp*
cgr-pm	
cgr-mass	
cin	*cin*

HMGB3

AT1G20696 - CI_ccm_4973

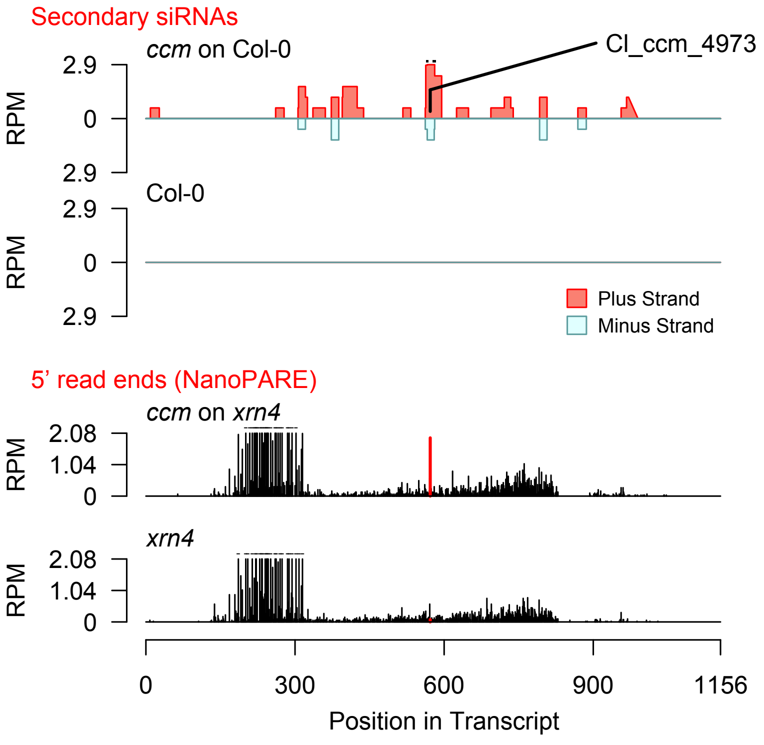

Diff. Exp. secondary siRNA locus not found

Allenscore: 2.5

Target Interaction:

```
5'- UGAAAAGUGGAAAUCCUUGU   AT1G20696.1
     : | |   | | | | | | | | | | | | | | |
3'- GCUCUUCACCUUUAGGAACU   CI_ccm_1979
```

Target Site: 572

Superfamily: SupFam_20

miRNAin ccm: Yes

Published name: Not published

Confirmed Targeting

2nd-siRNA	*NanoPARE*
ccm	*ccm*
cpe-2015	*cpe-2015*
cpe-2017	
cgr-dp	*cgr-dp*
cgr-pm	
cgr-mass	
cin	*cin*

HMGB3

AT1G20696 - CI_ccm_1979

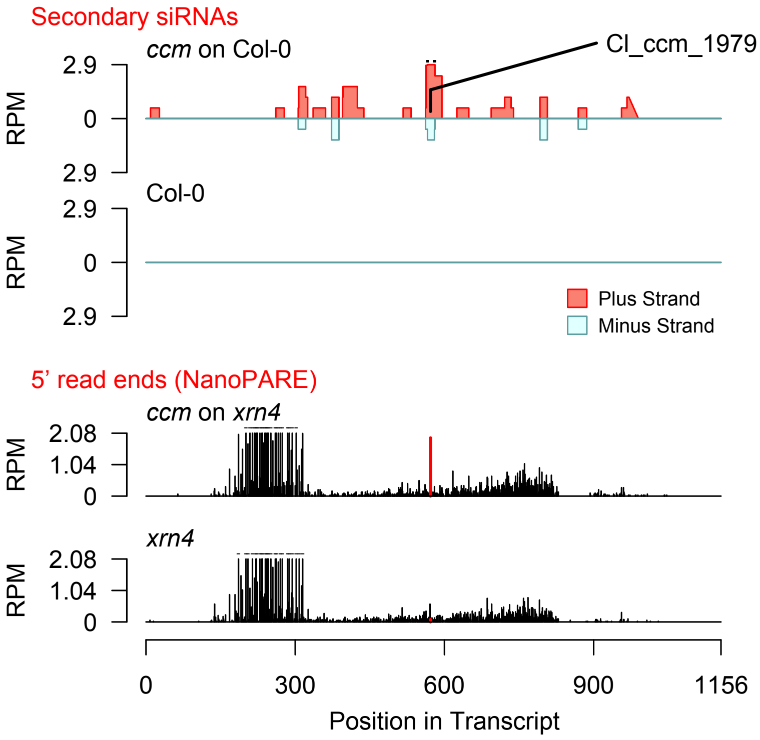

Diff. Exp. secondary siRNA locus not found

Allenscore: 3

Target Interaction:

```
5'- UCUGGUCUGCAAGUCAUGGU   AT4G03190.1
      ||  |||||||||:|||||||
3'- UGAGCAGACGUUUAGUACCA   CI_cpe-2015_526
```

Target Site: 436

Superfamily: SupFam_27

miRNAin ccm: Yes

Published name: N/A

Confirmed Targeting

2nd-siRNA	*NanoPARE*
ccm	*ccm*
cpe-2015	*cpe-2015*
cpe-2017	
cgr-dp	*cgr-dp*
cgr-pm	
cgr-mass	
cin	*cin*

AFB1

AT4G03190 - CI_cpe-2015_526

Secondary siRNAs

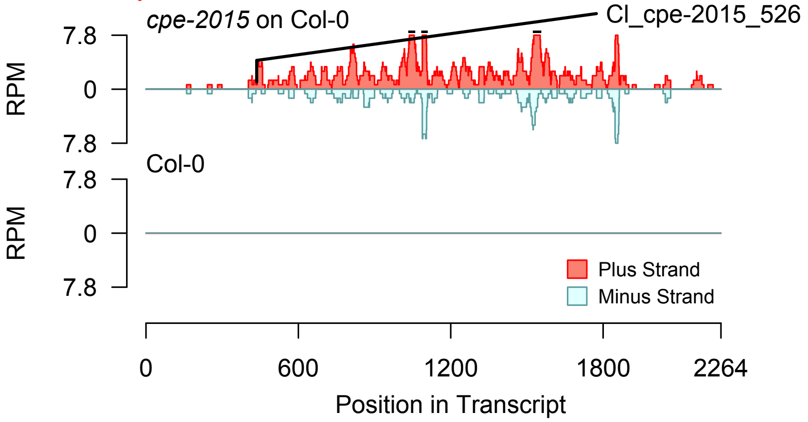

2nd siRNAs: Phase diagram

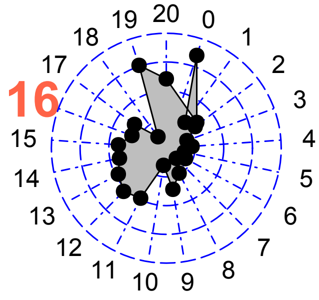

2nd siRNAs: Size distribution

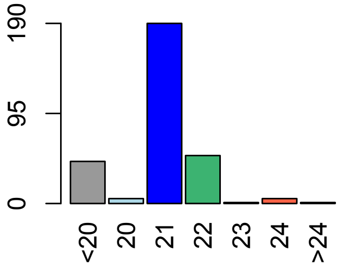

Degradome hits not found for sRNA

Allenscore: 3

Target Interaction:

```
5'- UCUGGUCUGCAAGUCAUGGU   AT4G03190.1
      ||  |||||||||:|||||||
3'- UGAGCAGACGUUUAGUACCA   Cl_cpe-2017_486
```

Target Site: 436

Superfamily: SupFam_27

miRNAin ccm: Yes

Published name: N/A

Confirmed Targeting

2nd-siRNA	*NanoPARE*
ccm	*ccm*
cpe-2015	*cpe-2015*
cpe-2017	
cgr-dp	*cgr-dp*
cgr-pm	
cgr-mass	
cin	*cin*

AFB1

AT4G03190 - Cl_cpe-2017_486

Secondary siRNAs

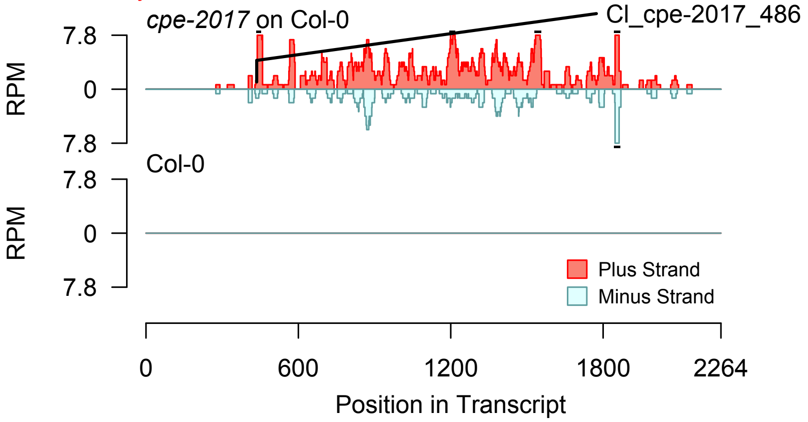

2nd siRNAs: Phase diagram

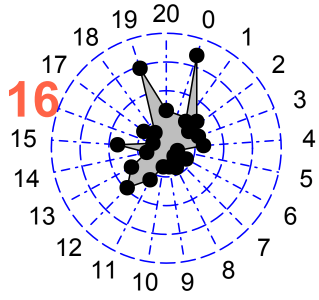

2nd siRNAs: Size distribution

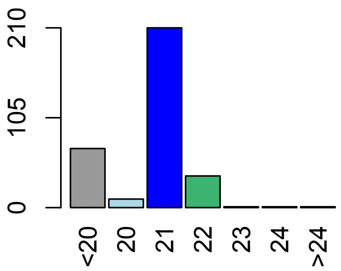

Degradome hits not found for sRNA

Allenscore: 5

Target Interaction:

```
5'- UUCUCUGGUCUGCAAGUCAUGG   AT4G03190.1
          |||  |||||||||:|||||||
3'- UUAAGAGCAGACGUUUAGUACC   Cl_cpe-2017_29015
```

Target Site: 435

Superfamily: SupFam_27

miRNAin ccm: Yes

Published name: N/A

Confirmed Targeting

2nd-siRNA	*NanoPARE*
ccm	*ccm*
cpe-2015	*cpe-2015*
cpe-2017	
cgr-dp	*cgr-dp*
cgr-pm	
cgr-mass	
cin	*cin*

AFB1

AT4G03190 - Cl_cpe-2017_29015

Secondary siRNAs

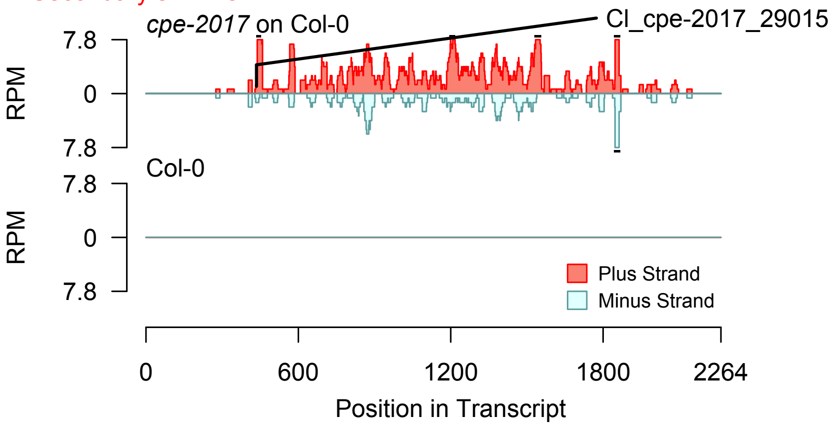

2nd siRNAs: Phase diagram

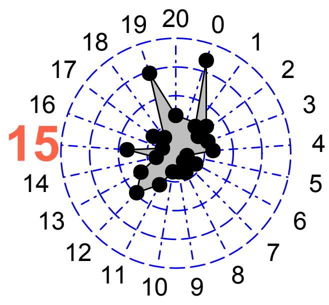

2nd siRNAs: Size distribution

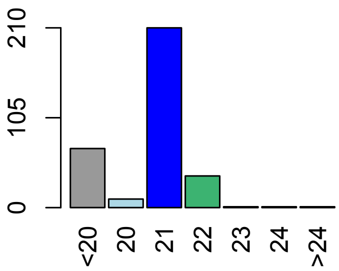

Degradome hits not found for sRNA

Allenscore: 2

Target Interaction:

```
5'- CUUGUAUGCAAAUCAUGGUACA   AT3G26810.1
    ||:|| |:||||||||||||||
3'- GAGCAGAUGUUUAGUACCAUGU   CI_ccm_2
```

Target Site: 781

Superfamily: SupFam_27

miRNAin ccm: Yes

Published name: ccm-MIR12497a*

Confirmed Targeting

2nd-siRNA	*NanoPARE*
ccm	*ccm*
cpe-2015	*cpe-2015*
cpe-2017	
cgr-dp	*cgr-dp*
cgr-pm	
cgr-mass	
cin	*cin*

AFB2

AT3G26810 - CI_ccm_2

2nd siRNAs: Phase diagram

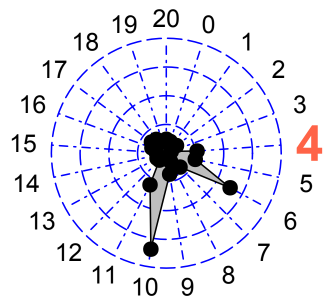

Secondary siRNAs

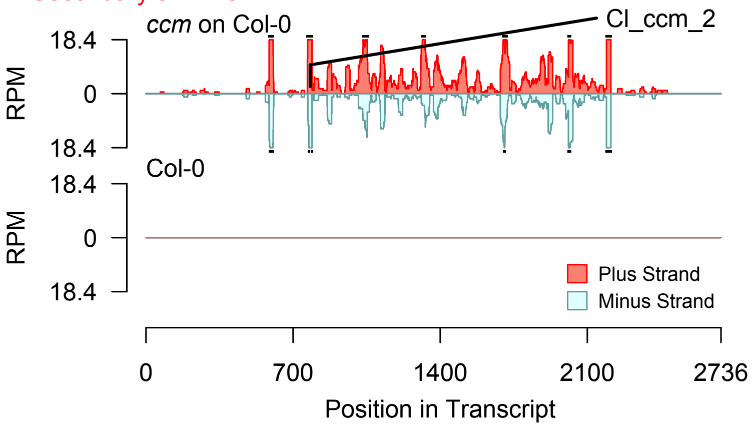

2nd siRNAs: Size distribution

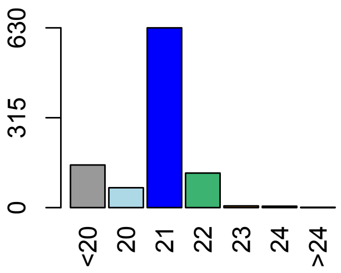

Degradome hits not found for sRNA

Allenscore: 3

Target Interaction:

```
5'- CUUGUAUGCAAAUCAUGGUACA    AT3G26810.1
       |   | | | | | | | | | | | | | | | | | | |
3'- AACCAUACGUUUAGUACCAUGU    Cl_ccm_614
```

Target Site: 781

Superfamily: SupFam_27

miRNAin ccm: Yes

Published name: ccm-MIR12497b

Confirmed Targeting

2nd-siRNA	*NanoPARE*
ccm	*ccm*
cpe-2015	*cpe-2015*
cpe-2017	
cgr-dp	*cgr-dp*
cgr-pm	
cgr-mass	
cin	*cin*

AFB2

AT3G26810 - Cl_ccm_614

Secondary siRNAs

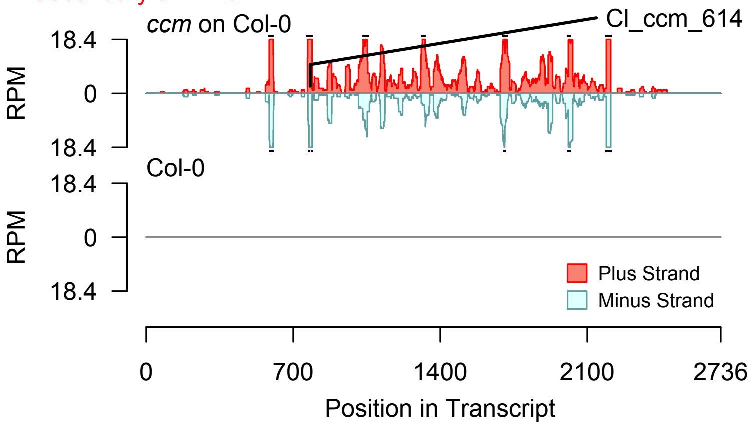

Degradome hits not found for sRNA

2nd siRNAs: Phase diagram

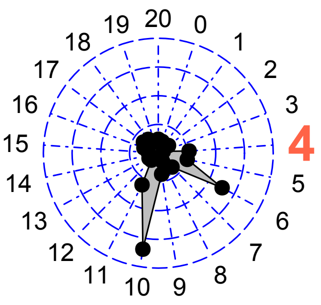

2nd siRNAs: Size distribution

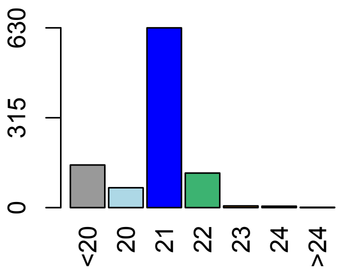

Allenscore: 1.5

Target Interaction:

```
5'- CUUGUAUGCAAAUCAUGGUACA    AT3G26810.1
     | | : | |   | | | | | | | | | | | | | | | |
3'- GAGCAGACGUUUAGUACCAUGU    CI_cpe-2015_7
```

Target Site: 781

Superfamily: SupFam_27

miRNAin ccm: Yes

Published name: N/A

Confirmed Targeting

2nd-siRNA	*NanoPARE*
ccm	*ccm*
cpe-2015	*cpe-2015*
cpe-2017	
cgr-dp	*cgr-dp*
cgr-pm	
cgr-mass	
cin	*cin*

AFB2

AT3G26810 - CI_cpe-2015_7

Secondary siRNAs

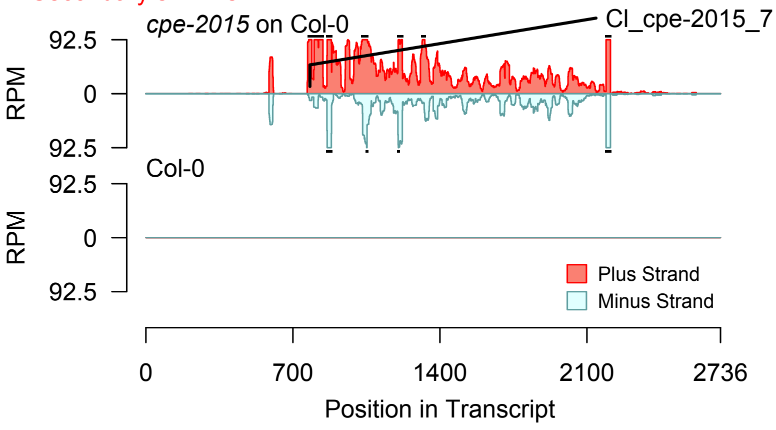

2nd siRNAs: Phase diagram

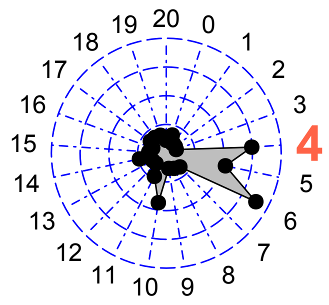

2nd siRNAs: Size distribution

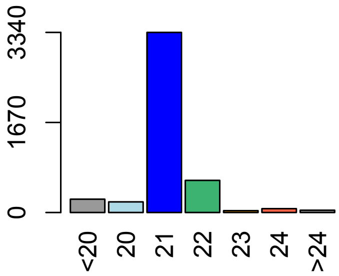

Degradome hits not found for sRNA

Allenscore: 1.5

Target Interaction:

5'- CUUGUAUGCAAAUCAUGGUACA AT3G26810.1
| | : | | | | | | | | | | | | | | | | | | |
3'- GAGCAGACGUUUAGUACCAUGU CI_cpe-2017_11

Target Site: 781

Superfamily: SupFam_27

miRNAin ccm: Yes

Published name: N/A

Confirmed Targeting

2nd-siRNA	*NanoPARE*
ccm	*ccm*
cpe-2015	*cpe-2015*
cpe-2017	
cgr-dp	*cgr-dp*
cgr-pm	
cgr-mass	
cin	*cin*

AFB2

AT3G26810 - CI_cpe-2017_11

Secondary siRNAs

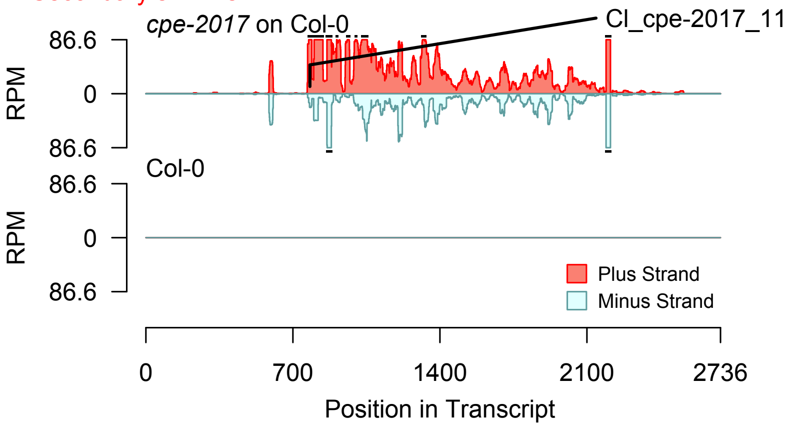

2nd siRNAs: Phase diagram

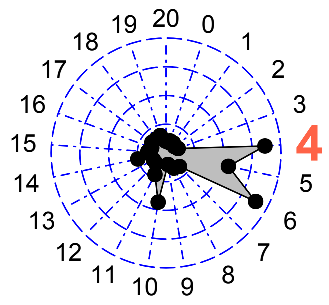

2nd siRNAs: Size distribution

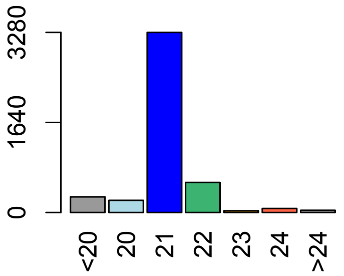

Degradome hits not found for sRNA

Allenscore: 1.5

Target Interaction:

```
5'- GGGAUGAGAAAGUGAAGGUUCU    AT3G27300.1
     | : | | | | | | | | | | | | | | | | | | | |
3'- CUCUACUCUUUCACUUCCAAGU    Cl_cpe-2015_537
```

Target Site: 1099

Superfamily: SupFam_312

miRNAin ccm: Yes

Published name: N/A

Confirmed Targeting

2nd-siRNA	*NanoPARE*
ccm	*ccm*
cpe-2015	*cpe-2015*
cpe-2017	
cgr-dp	*cgr-dp*
cgr-pm	
cgr-mass	
cin	*cin*

G6PD5

AT3G27300 - Cl_cpe-2015_537

Secondary siRNAs

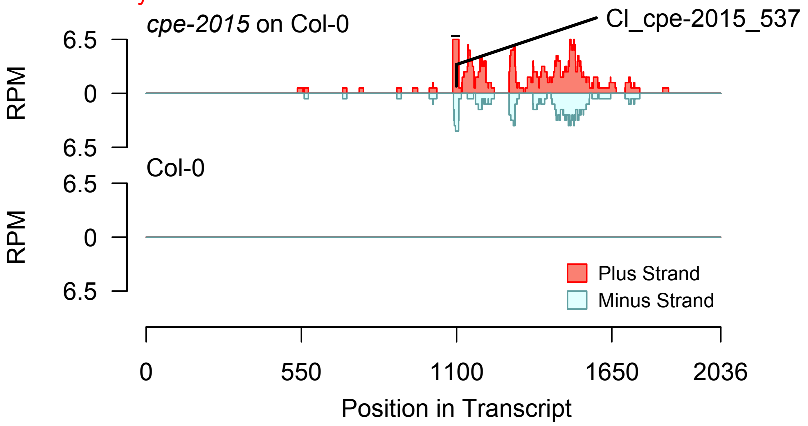

2nd siRNAs: Phase diagram

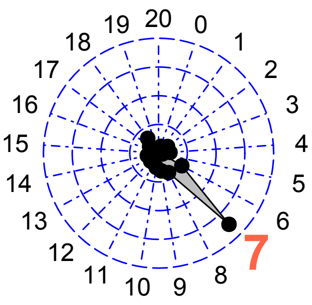

2nd siRNAs: Size distribution

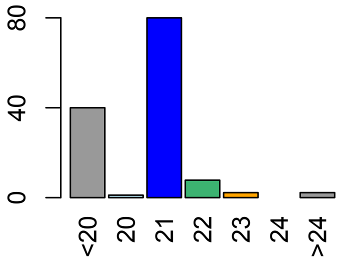

Degradome hits not found for sRNA

Allenscore: 1.5

Target Interaction:

```
5'- GGGAUGAGAAAGUGAAGGUUCU    AT3G27300.1
     | : | | | | | | | | | | | | | | | | | | |
3'- CUCUACUCUUUCACUUCCAAGU    CI_cpe-2017_615
```

Target Site: 1099

Superfamily: SupFam_312

miRNAin ccm: Yes

Published name: N/A

Confirmed Targeting

2nd-siRNA	*NanoPARE*
ccm	*ccm*
cpe-2015	*cpe-2015*
cpe-2017	
cgr-dp	*cgr-dp*
cgr-pm	
cgr-mass	
cin	*cin*

G6PD5

AT3G27300 - CI_cpe-2017_615

Secondary siRNAs

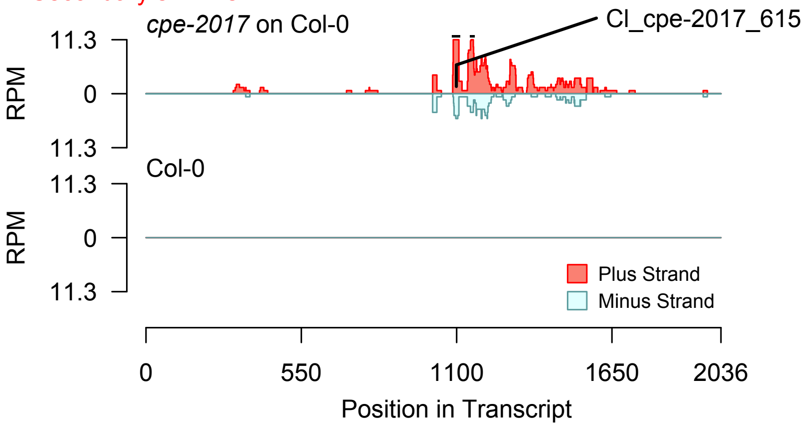

2nd siRNAs: Phase diagram

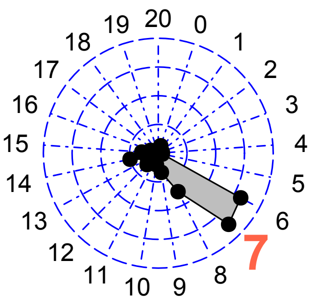

2nd siRNAs: Size distribution

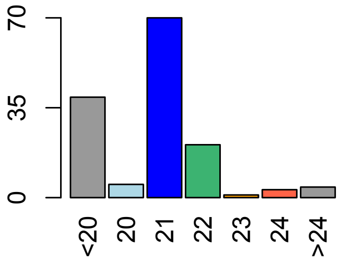

Degradome hits not found for sRNA

Allenscore: 1.5

Target Interaction:

```
5'- GGGAUGAGAAAGUGAAGGUUCU   AT3G27300.1
     | : | | | | | | | | | | | | | | | | | | | |
3'- CUCUACUCUUUCACUUCCAAGU   CI_ccm_5718
```

Target Site: 1099

Superfamily: SupFam_312

miRNAin ccm: Yes

Published name: Not published

Confirmed Targeting

2nd-siRNA	*NanoPARE*
ccm	*ccm*
cpe-2015	*cpe-2015*
cpe-2017	
cgr-dp	*cgr-dp*
cgr-pm	
cgr-mass	
cin	*cin*

G6PD5

AT3G27300 - CI_ccm_5718

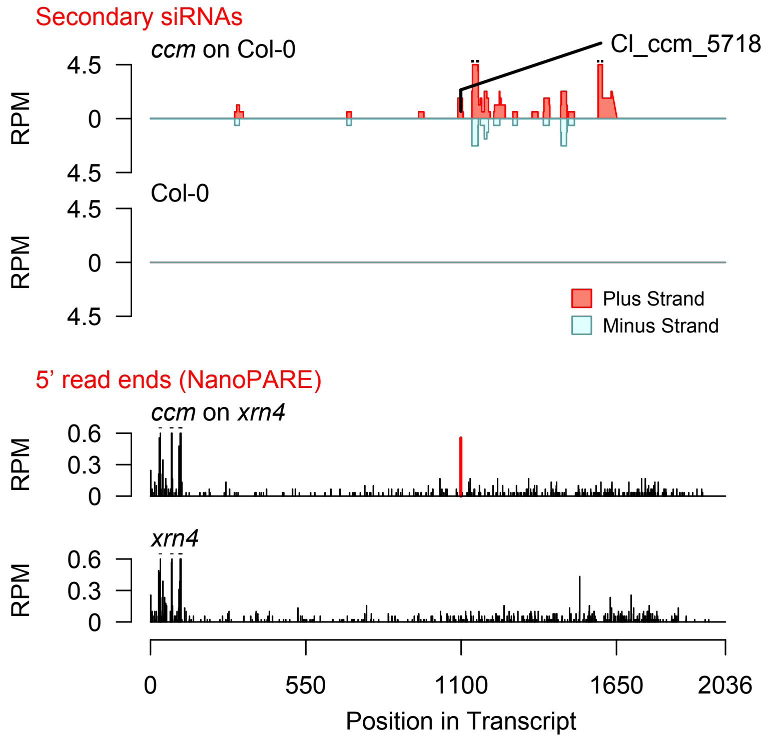

Diff. Exp. secondary siRNA locus not found

Allenscore: 4

Target Interaction:

5'- CUGGUUACUUAGACCCAGAGUA AT4G00330.1
| | | | | | | | | | | | | | | | | | | | |
3'- AACCAAUGAAUCUAGGUCUCAC Cl_cpe-2015_439

Target Site: 1015

Superfamily: SupFam_257

miRNAin ccm: N/A

Published name: N/A

Confirmed Targeting

2nd-siRNA	*NanoPARE*
ccm	*ccm*
cpe-2015	*cpe-2015*
cpe-2017	
cgr-dp	*cgr-dp*
cgr-pm	
cgr-mass	
cin	*cin*

CRCK2

AT4G00330 - Cl_cpe-2015_439

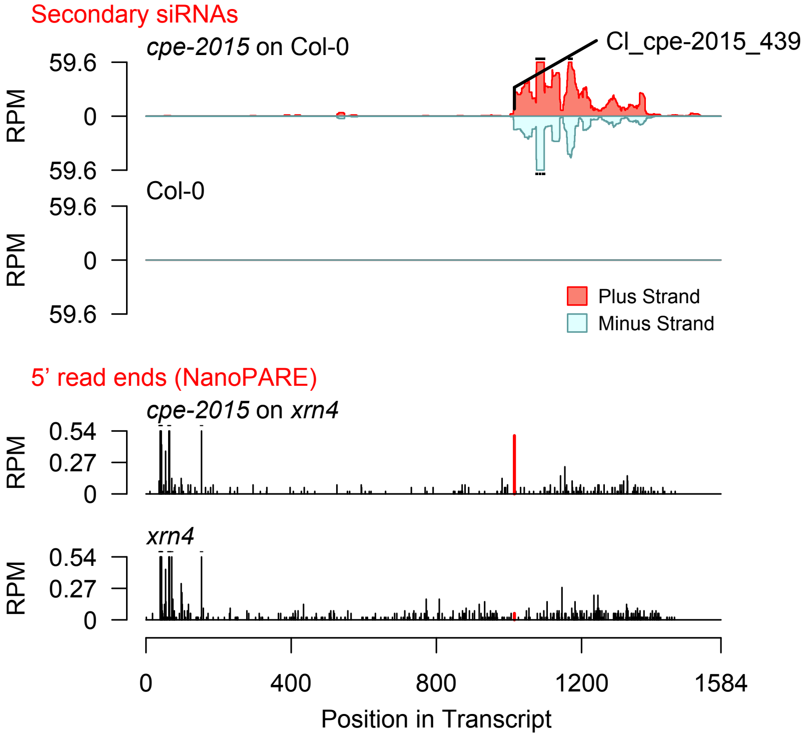

2nd siRNAs: Phase diagram

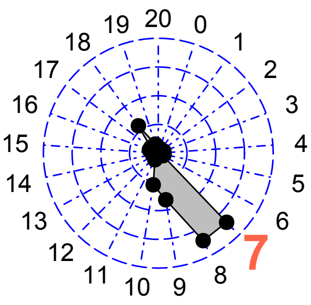

2nd siRNAs: Size distribution

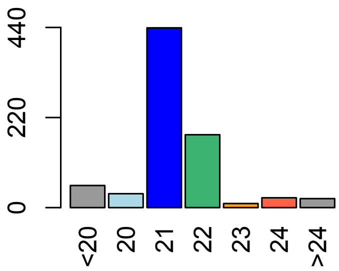

Allenscore: 3.5

Target Interaction:

```
5'- GGUUACUUAGACCCAGAGUA   AT4G00330.1
     : | | | | | | | | | | |    | | | | | | | |
3'- UCAAUGAAUCUAGGUCUCAC   CI_cpe-2015_45087
```

Target Site: 1015

Superfamily: SupFam_257

miRNAin ccm: N/A

Published name: N/A

Confirmed Targeting

2nd-siRNA	*NanoPARE*
ccm	*ccm*
cpe-2015	*cpe-2015*
cpe-2017	
cgr-dp	*cgr-dp*
cgr-pm	
cgr-mass	
cin	*cin*

CRCK2

AT4G00330 - CI_cpe-2015_45087

Secondary siRNAs

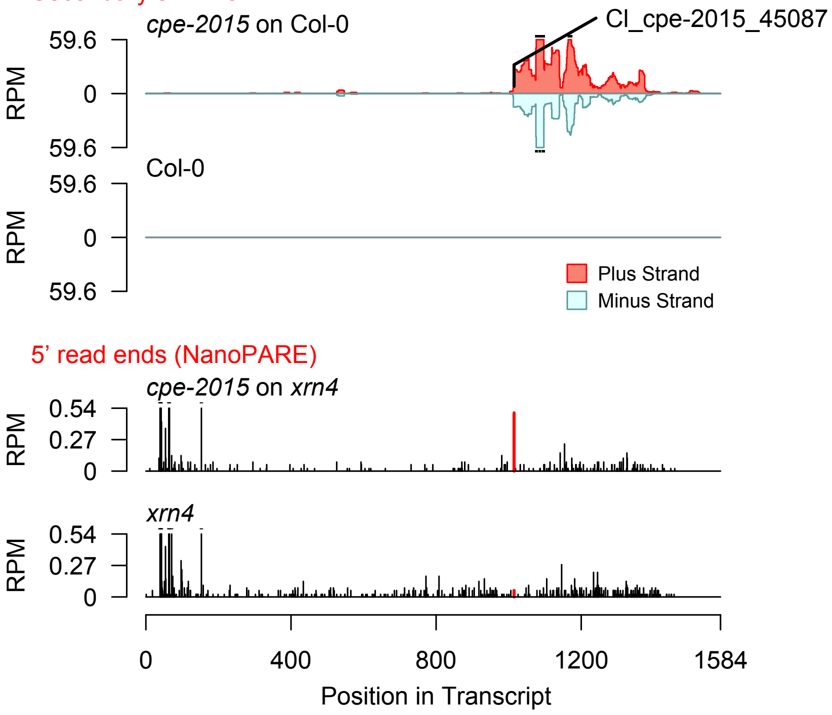

2nd siRNAs: Phase diagram

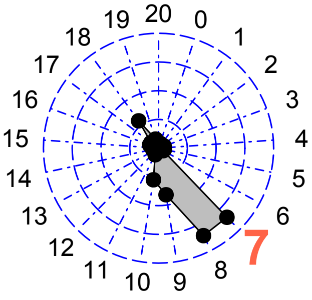

2nd siRNAs: Size distribution

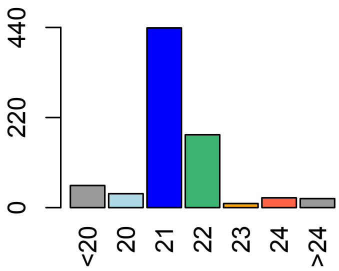

Allenscore: 4

Target Interaction:

```
5'- CUGGUUACUUAGACCCAGAGUA   AT4G00330.1
      | | | | | | | | | | | |   | | | | | | | |
3'- AACCAAUGAAUCUAGGUCUCAC   Cl_cpe-2017_503
```

Target Site: 1015

Superfamily: SupFam_257

miRNAin ccm: N/A

Published name: N/A

Confirmed Targeting

2nd-siRNA	*NanoPARE*
ccm	*ccm*
cpe-2015	*cpe-2015*
cpe-2017	
cgr-dp	*cgr-dp*
cgr-pm	
cgr-mass	
cin	*cin*

CRCK2

AT4G00330 - Cl_cpe-2017_503

Secondary siRNAs

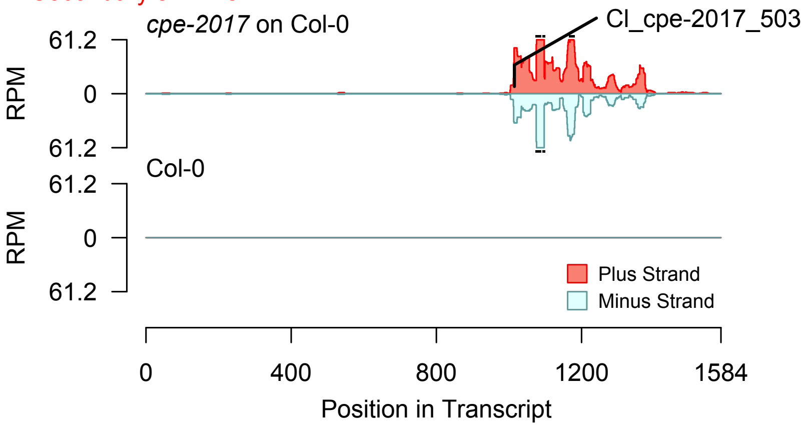

2nd siRNAs: Phase diagram

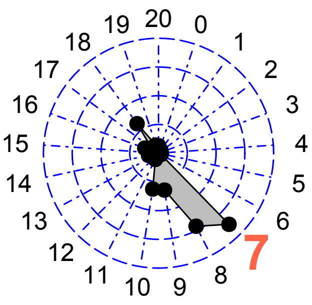

2nd siRNAs: Size distribution

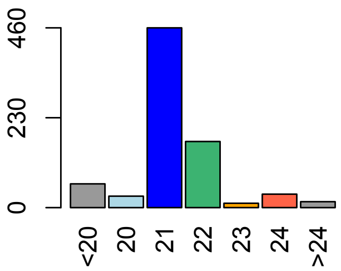

Degradome hits not found for sRNA

Allenscore: 5

Target Interaction:

```
5'- GGUUACUUAGACCCAGAGUA   AT4G00330.1
     ||||||||||| | |||||
3'- CCAAUGAAUCUAGAUCUCAC   Cl_cpe-2017_25056
```

Target Site: 1015

Superfamily: SupFam_257

miRNAin ccm: N/A

Published name: N/A

Confirmed Targeting

2nd-siRNA	*NanoPARE*
ccm	*ccm*
cpe-2015	*cpe-2015*
cpe-2017	
cgr-dp	*cgr-dp*
cgr-pm	
cgr-mass	
cin	*cin*

CRCK2

AT4G00330 - Cl_cpe-2017_25056

Secondary siRNAs

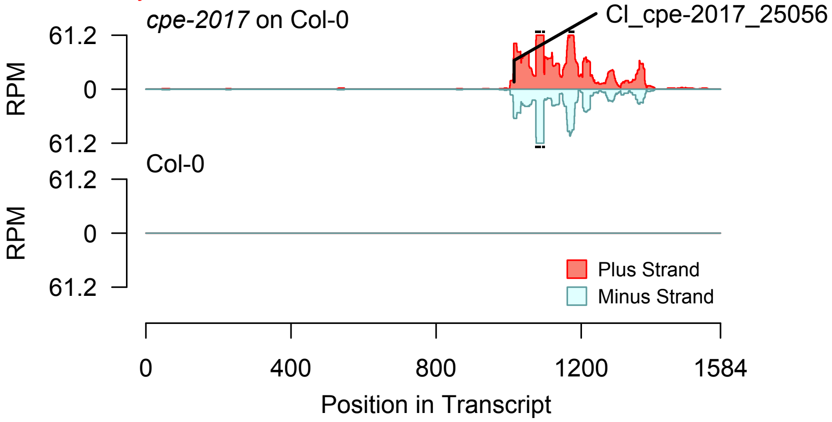

2nd siRNAs: Phase diagram

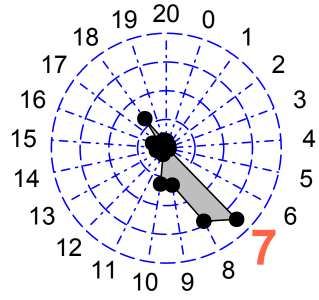

2nd siRNAs: Size distribution

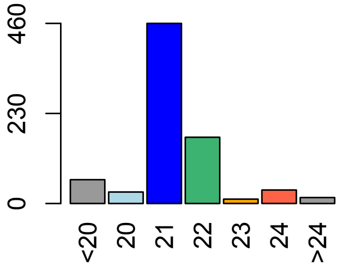

Degradome hits not found for sRNA

Allenscore: 5

Target Interaction:

```
5'- CAUCU-UCUUCUUCCCUCGAUUCA   AT3G51990.1
     |||||  ||||||||||| ||  ||||
3'- GUAGACAGAAGAAGGG-GC-AAGU   Cl_cpe-2015_44
```

Target Site: 221

Superfamily: SupFam_269

miRNAin ccm: Yes

Published name: N/A

Confirmed Targeting

2nd-siRNA	*NanoPARE*
ccm	*ccm*
cpe-2015	*cpe-2015*
cpe-2017	
cgr-dp	*cgr-dp*
cgr-pm	
cgr-mass	
cin	*cin*

Protein_kinase_superfamily_protein

AT3G51990 - Cl_cpe-2015_44

Secondary siRNAs

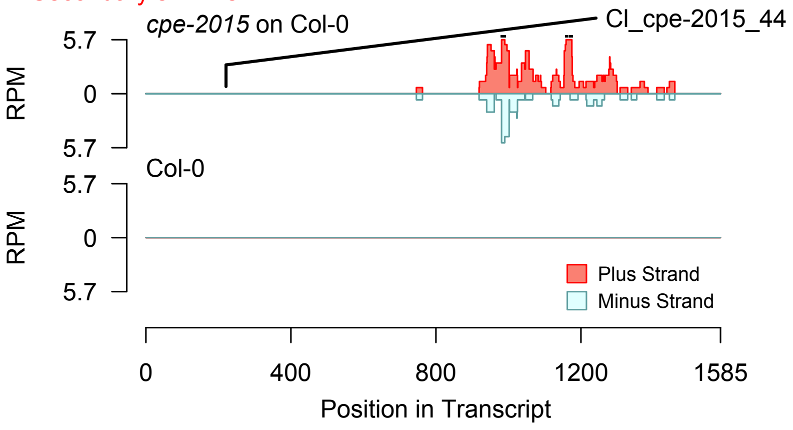

2nd siRNAs: Phase diagram

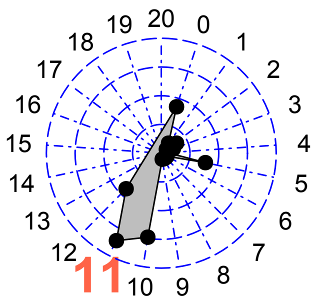

2nd siRNAs: Size distribution

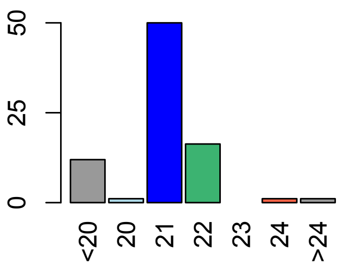

Degradome hits not found for sRNA

Allenscore: 5.5

Target Interaction:

```
5'- GGUUACUUAGAUCCAGAUUA   AT3G51990.1
     : | | | | | | | | | | | | | | | | |    |
3'- UCAAUGAAUCUAGGUCUCAC   CI_cpe-2015_45087
```

Target Site: 921

Superfamily: SupFam_257

miRNAin ccm: N/A

Published name: N/A

Confirmed Targeting

2nd-siRNA	*NanoPARE*
ccm	*ccm*
cpe-2015	*cpe-2015*
cpe-2017	
cgr-dp	*cgr-dp*
cgr-pm	
cgr-mass	
cin	*cin*

Protein_kinase_superfamily_protein

AT3G51990 - CI_cpe-2015_45087

2nd siRNAs: Phase diagram

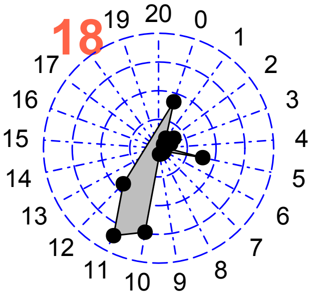

Secondary siRNAs

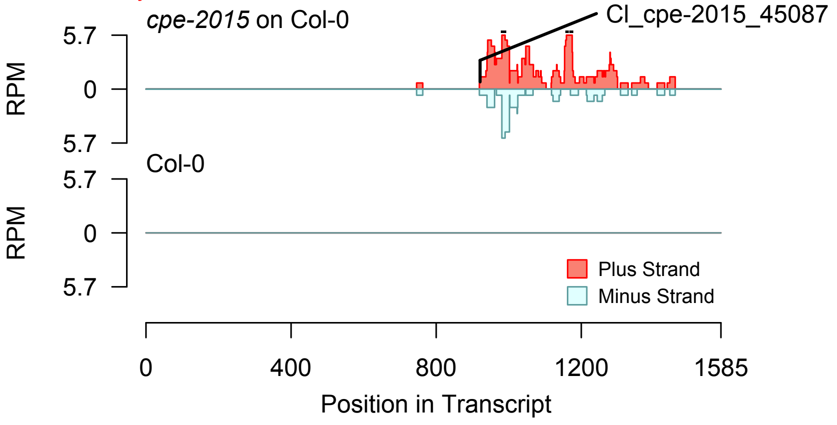

2nd siRNAs: Size distribution

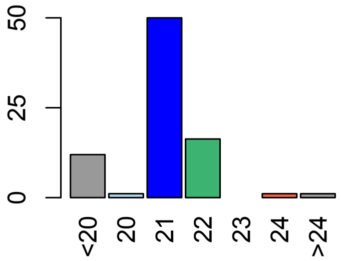

Degradome hits not found for sRNA

Allenscore: 5

Target Interaction:

```
5'- CAUCU-UCUUCUUCCCUCGAUUCA    AT3G51990.1
     |||||  ||||||||||| ||  ||||
3'- GUAGACAGAAGAAGGG-GC-AAGU    Cl_cpe-2017_47
```

Target Site: 221

Superfamily: SupFam_269

miRNAin ccm: Yes

Published name: N/A

Confirmed Targeting

2nd-siRNA	*NanoPARE*
ccm	*ccm*
cpe-2015	*cpe-2015*
cpe-2017	
cgr-dp	*cgr-dp*
cgr-pm	
cgr-mass	
cin	*cin*

Protein_kinase_superfamily_protein

AT3G51990 - Cl_cpe-2017_47

Secondary siRNAs

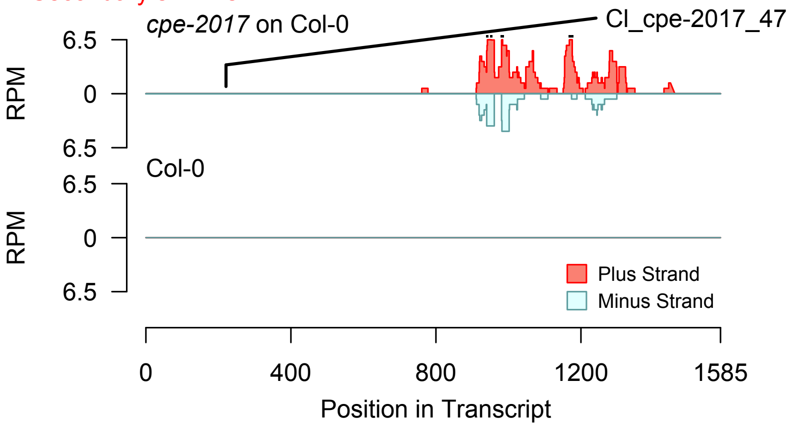

2nd siRNAs: Phase diagram

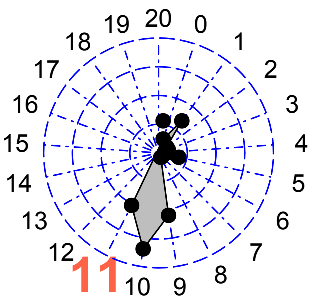

2nd siRNAs: Size distribution

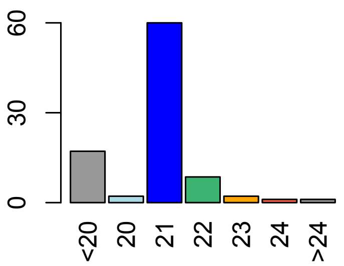

Degradome hits not found for sRNA

Allenscore: 3

Target Interaction:

```
5'- ACCGGAAGAAGAUGAGAAGCUUA    AT1G63910.1
     | | |   | | | | | | | | | | | : | | | | | : |
3'- UGGGCUUCUUCUACUUUUCGAGU    Cl_cgr-dp_279
```

Target Site: 216

Superfamily: SupFam_37

miRNAin ccm: N/A

Published name: N/A

Confirmed Targeting

2nd-siRNA	*NanoPARE*
ccm	*ccm*
cpe-2015	*cpe-2015*
cpe-2017	
cgr-dp	*cgr-dp*
cgr-pm	
cgr-mass	
cin	*cin*

AtMYB103

AT1G63910 - Cl_cgr-dp_279

Secondary siRNAs

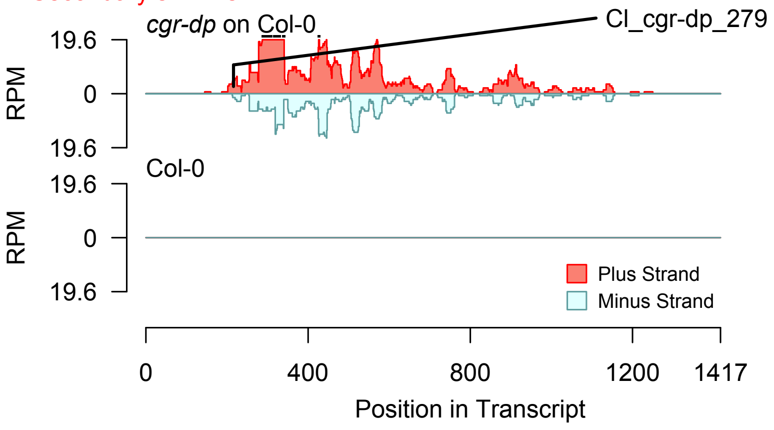

2nd siRNAs: Phase diagram

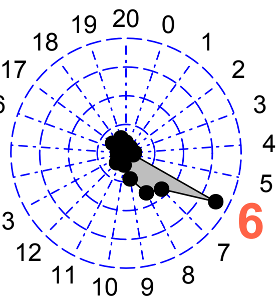

2nd siRNAs: Size distribution

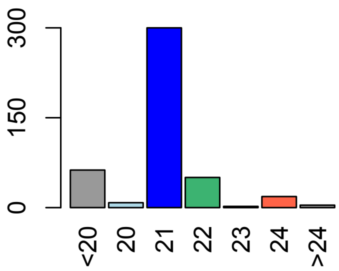

Degradome hits not found for sRNA

Allenscore: 3

Target Interaction:

```
5'- GGAAGAAGAUGAGAAGCUUA   AT1G63910.1
     |||||||||||:|||||:|
3'- GCUUCUUCUACUUUUCGAGU   Cl_cgr-dp_37384
```

Target Site: 216

Superfamily: SupFam_37

miRNAin ccm: N/A

Published name: N/A

Confirmed Targeting

2nd-siRNA	*NanoPARE*
ccm	*ccm*
cpe-2015	*cpe-2015*
cpe-2017	
cgr-dp	*cgr-dp*
cgr-pm	
cgr-mass	
cin	*cin*

AtMYB103

AT1G63910 - Cl_cgr-dp_37384

Secondary siRNAs

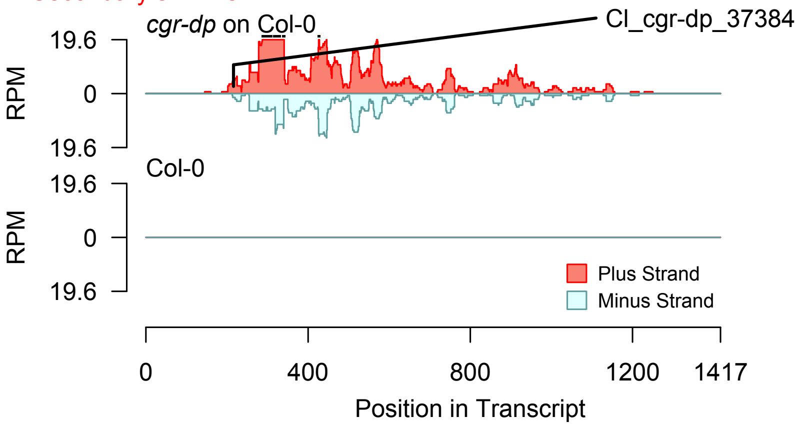

2nd siRNAs: Phase diagram

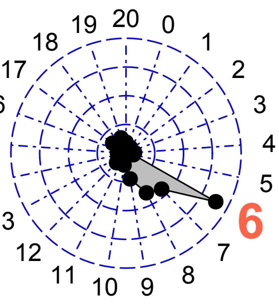

2nd siRNAs: Size distribution

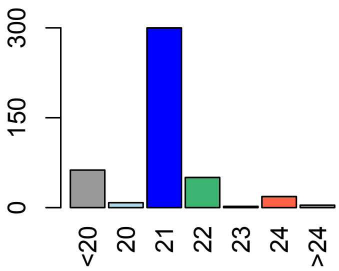

Degradome hits not found for sRNA

Allenscore: 2

Target Interaction:

```
5'- ACCGGAAGAAGAUGAGAAGCUUA   AT1G63910.1
     | | | | | | | | | | | | | | | | : | | | | | : |
3'- UGGCCUUCUUCUACUUUUCGAGU   Cl_cgr-pm_3376
```

Target Site: 216

Superfamily: SupFam_37

miRNAin ccm: N/A

Published name: N/A

Confirmed Targeting

2nd-siRNA	*NanoPARE*
ccm	*ccm*
cpe-2015	*cpe-2015*
cpe-2017	
cgr-dp	*cgr-dp*
cgr-pm	
cgr-mass	
cin	*cin*

AtMYB103

AT1G63910 - Cl_cgr-pm_3376

Secondary siRNAs

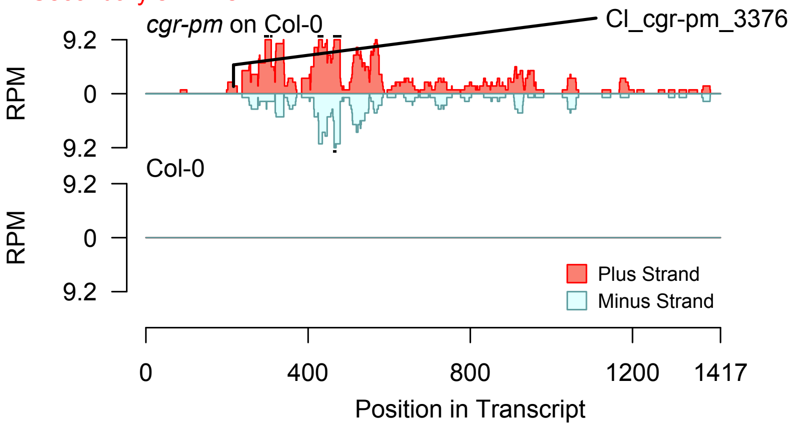

2nd siRNAs: Phase diagram

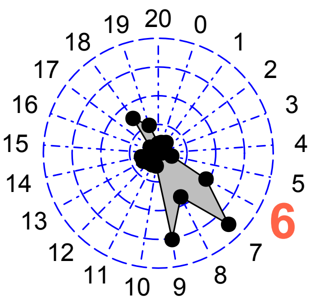

2nd siRNAs: Size distribution

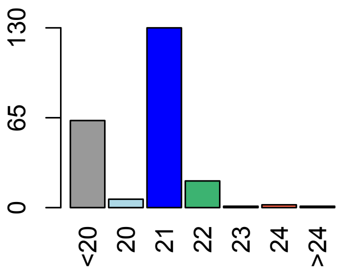

Degradome hits not found for sRNA

Allenscore: 4

Target Interaction:

```
5'- CCGGAAGAAGAUGAGAAGCUUA   AT1G63910.1
     ||   ||||||||:||||||:||:|
3'- GGACUUCUUCUGCUCUUUGAGU   Cl_cgr-pm_5111
```

Target Site: 216

Superfamily: SupFam_37

miRNAin ccm: N/A

Published name: N/A

Confirmed Targeting

2nd-siRNA	*NanoPARE*
ccm	*ccm*
cpe-2015	*cpe-2015*
cpe-2017	
cgr-dp	*cgr-dp*
cgr-pm	
cgr-mass	
cin	*cin*

AtMYB103

AT1G63910 - Cl_cgr-pm_5111

Secondary siRNAs

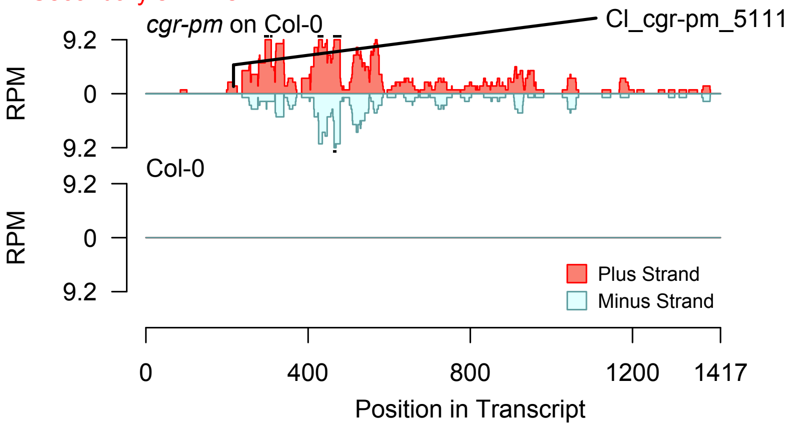

Degradome hits not found for sRNA

2nd siRNAs: Phase diagram

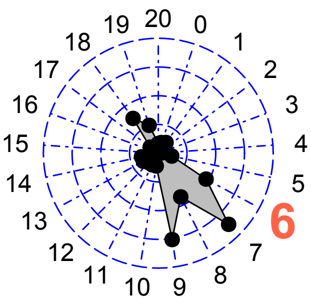

2nd siRNAs: Size distribution

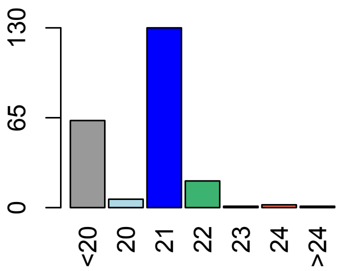

Allenscore: 4

Target Interaction:

```
5'- CCGGAAGAAGAUGAGAAGCUUA   AT1G63910.1
     | |   | | | | | | | | : | | | | | : | | : |
3'- GGACUUCUUCUGCUCUUUGAGU   Cl_cgr-mass_3016
```

Target Site: 216

Superfamily: SupFam_37

miRNAin ccm: N/A

Published name: N/A

Confirmed Targeting

2nd-siRNA	*NanoPARE*
ccm	*ccm*
cpe-2015	*cpe-2015*
cpe-2017	
cgr-dp	*cgr-dp*
cgr-pm	
cgr-mass	
cin	*cin*

AtMYB103

AT1G63910 - Cl_cgr-mass_3016

Secondary siRNAs

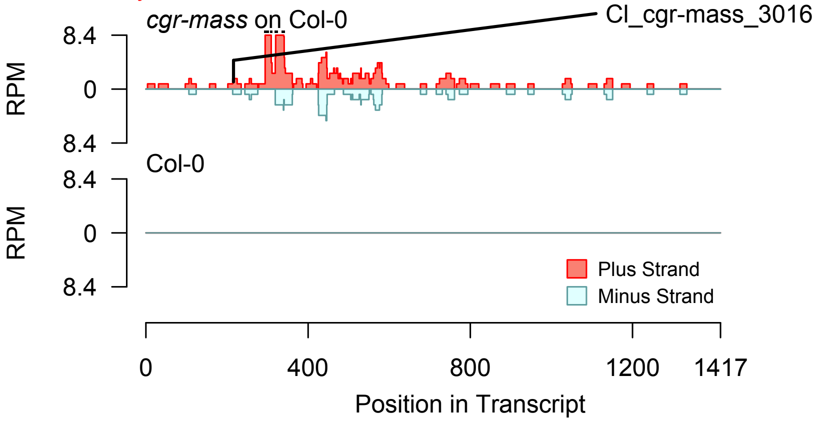

2nd siRNAs: Phase diagram

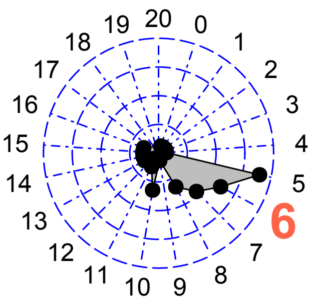

2nd siRNAs: Size distribution

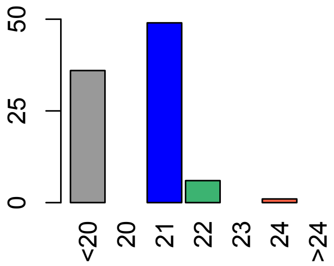

Degradome hits not found for sRNA

Allenscore: 2

Target Interaction:

```
5'- ACCGGAAGAAGAUGAGAAGCUUA    AT1G63910.1
    | | | | | | | | | | | | | | | : | | | | | : |
3'- UGGCCUUCUUCUACUUUUCGAGU    Cl_cgr-mass_4415
```

Target Site: 216

Superfamily: SupFam_37

miRNAin ccm: N/A

Published name: N/A

Confirmed Targeting

2nd-siRNA	*NanoPARE*
ccm	*ccm*
cpe-2015	*cpe-2015*
cpe-2017	
cgr-dp	*cgr-dp*
cgr-pm	
cgr-mass	
cin	*cin*

AtMYB103

AT1G63910 - Cl_cgr-mass_4415

Secondary siRNAs

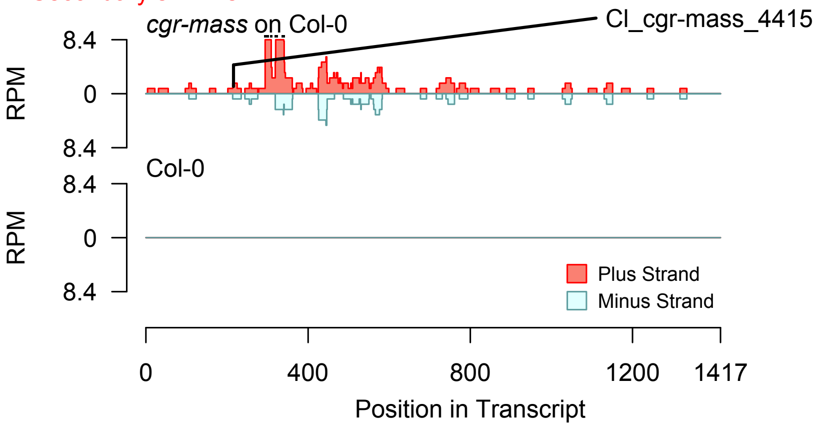

2nd siRNAs: Phase diagram

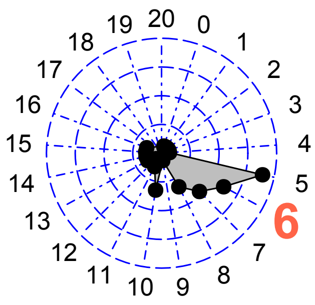

2nd siRNAs: Size distribution

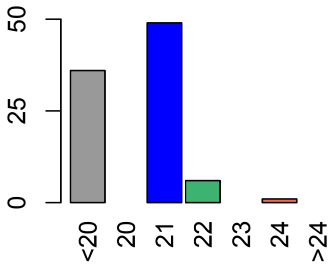

Degradome hits not found for sRNA

Allenscore: 6

Target Interaction:

```
5'- UCGUCAGCUCAAUACUUAUGGU    AT1G46264.1
        |   | | | | | | | | | | |    | | | | | |
3'- CUCUGUCGAGUUAUGUAUACCU    CI_ccm_19
```

Target Site: 457

Superfamily: SupFam_116

miRNAin ccm: Yes

Published name: ccm-MIR12494b

Confirmed Targeting

2nd-siRNA	*NanoPARE*
ccm	*ccm*
cpe-2015	*cpe-2015*
cpe-2017	
cgr-dp	*cgr-dp*
cgr-pm	
cgr-mass	
cin	*cin*

HSFB4

AT1G46264 - CI_ccm_19

Secondary siRNAs

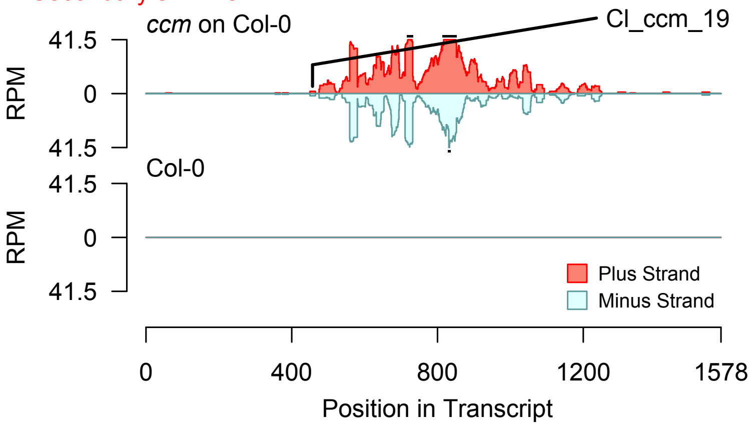

2nd siRNAs: Phase diagram

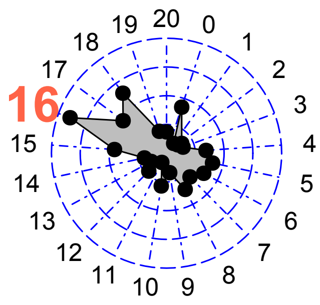

2nd siRNAs: Size distribution

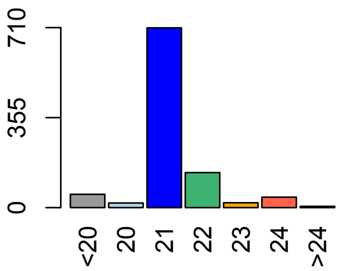

Degradome hits not found for sRNA

Allenscore: 6

Target Interaction:

```
5'- UCGUCAGCUCAAUACUUAUGGU    AT1G46264.1
       |  |||||||||||  |||||
3'- CUCUGUCGAGUUAUGUAUACCU    CI_cpe-2017_72
```

Target Site: 457

Superfamily: SupFam_116

miRNAin ccm: Yes

Published name: N/A

Confirmed Targeting

2nd-siRNA	*NanoPARE*
ccm	*ccm*
cpe-2015	*cpe-2015*
cpe-2017	
cgr-dp	*cgr-dp*
cgr-pm	
cgr-mass	
cin	*cin*

HSFB4

AT1G46264 - CI_cpe-2017_72

Secondary siRNAs

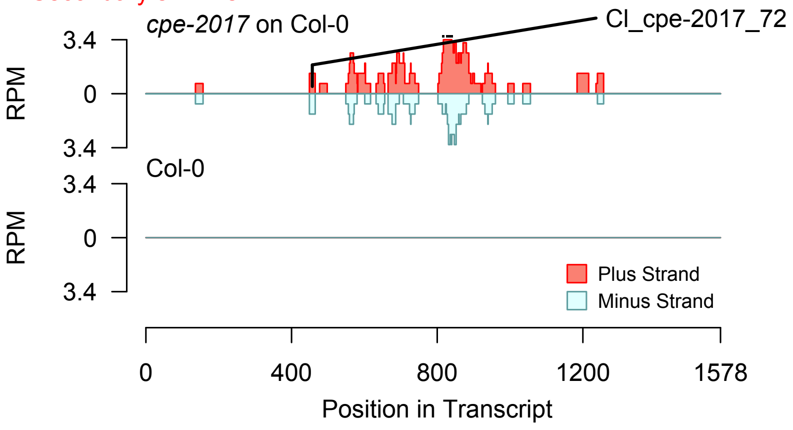

2nd siRNAs: Phase diagram

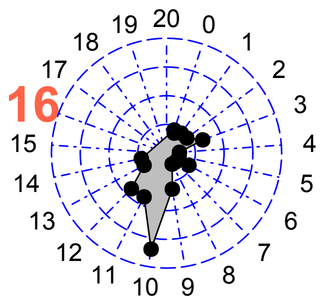

2nd siRNAs: Size distribution

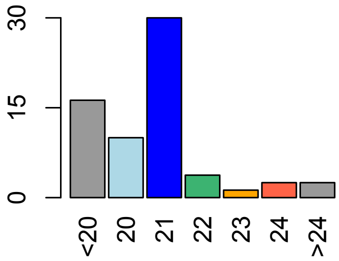

Degradome hits not found for sRNA

Allenscore: 5

Target Interaction:

```
5'- UCGUCAGCUCAAUACUUAUGGU    AT1G46264.1
     : | :   | | | | | | | | | | |   | | | | |
3'- GGUUGUCGAGUUAUGUAUACCU    CI_cpe-2017_3993
```

Target Site: 457

Superfamily: SupFam_116

miRNAin ccm: Yes

Published name: N/A

Confirmed Targeting

2nd-siRNA	*NanoPARE*
ccm	*ccm*
cpe-2015	*cpe-2015*
cpe-2017	
cgr-dp	*cgr-dp*
cgr-pm	
cgr-mass	
cin	*cin*

HSFB4

AT1G46264 - CI_cpe-2017_3993

Secondary siRNAs

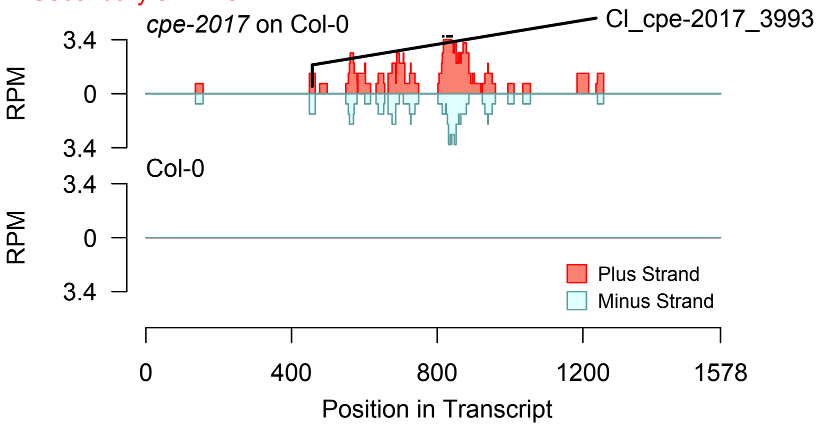

2nd siRNAs: Phase diagram

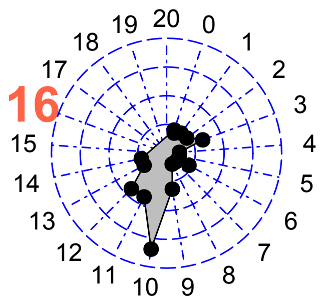

2nd siRNAs: Size distribution

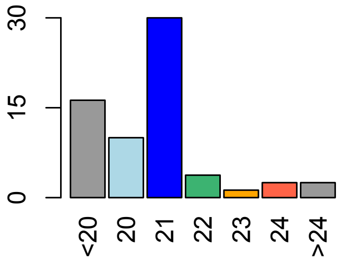

Degradome hits not found for sRNA

Allenscore: 4.5

Target Interaction:

```
5'- UACGGUUACAUUGCACCAGAAUA    AT5G61480.1
     : |   | | | | | |   : | | | | | | | | | | | | |
3'- GUCCCAAUGCGACGUGGUCUUAU    CI_ccm_818
```

Target Site: 3083

Superfamily: SupFam_124

miRNAin ccm: No

Published name: Not published

Confirmed Targeting

2nd-siRNA	*NanoPARE*
ccm	*ccm*
cpe-2015	*cpe-2015*
cpe-2017	
cgr-dp	*cgr-dp*
cgr-pm	
cgr-mass	
cin	*cin*

PXY

AT5G61480 - CI_ccm_818

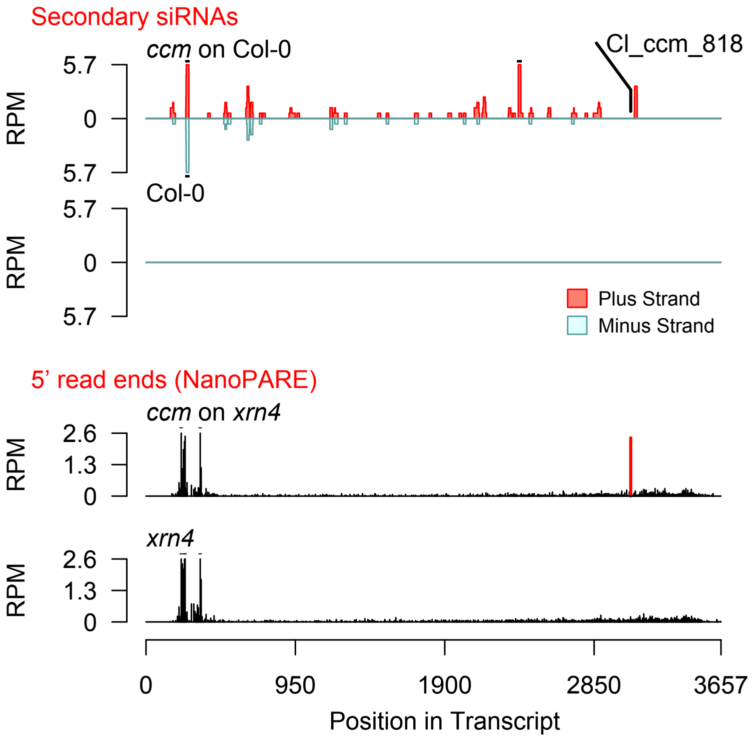

Diff. Exp. secondary siRNA
locus not found

Allenscore: 3

Target Interaction:

```
5'- AUGGAUGGUAAGUUUUAUGUGA   AT5G60570.1
     ||||||||  ||||:||||:||||
3'- UACCUACCUUUCGAAAUGCACU   CI_ccm_670
```

Target Site: 1307

Superfamily: SupFam_281

miRNAin ccm: Yes

Published name: Not published

Confirmed Targeting

2nd-siRNA	*NanoPARE*
ccm	*ccm*
cpe-2015	*cpe-2015*
cpe-2017	
cgr-dp	*cgr-dp*
cgr-pm	
cgr-mass	
cin	*cin*

Galactose_oxidase_superfamily_protein

AT5G60570 - CI_ccm_670

Secondary siRNAs

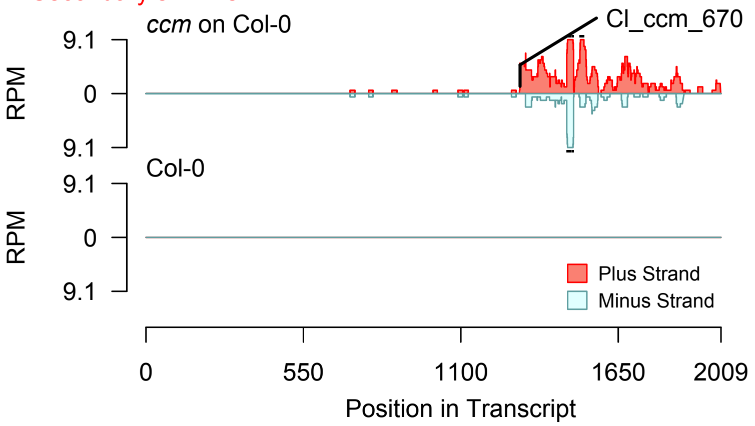

2nd siRNAs: Phase diagram

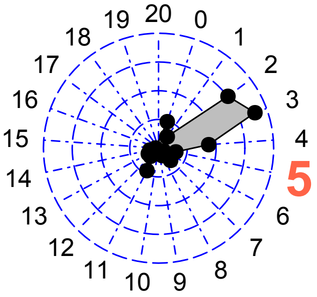

2nd siRNAs: Size distribution

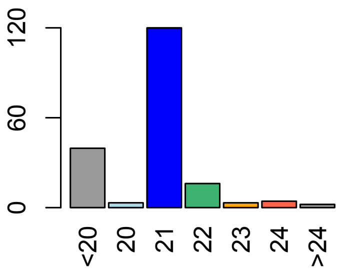

Degradome hits not found for sRNA

Allenscore: 3

Target Interaction:

```
5'- AUGGAUGGUAAGUUUUAUGUGA    AT5G60570.1
     | | | | | | | |   | | | : | | | | : | | | |
3'- UACCUACCUUUCGAAAUGCACU    Cl_cpe-2015_316
```

Target Site: 1307

Superfamily: SupFam_281

miRNAin ccm: Yes

Published name: N/A

Confirmed Targeting

2nd-siRNA	*NanoPARE*
ccm	*ccm*
cpe-2015	*cpe-2015*
cpe-2017	
cgr-dp	*cgr-dp*
cgr-pm	
cgr-mass	
cin	*cin*

Galactose_oxidase_superfamily_protein

AT5G60570 - Cl_cpe-2015_316

Secondary siRNAs

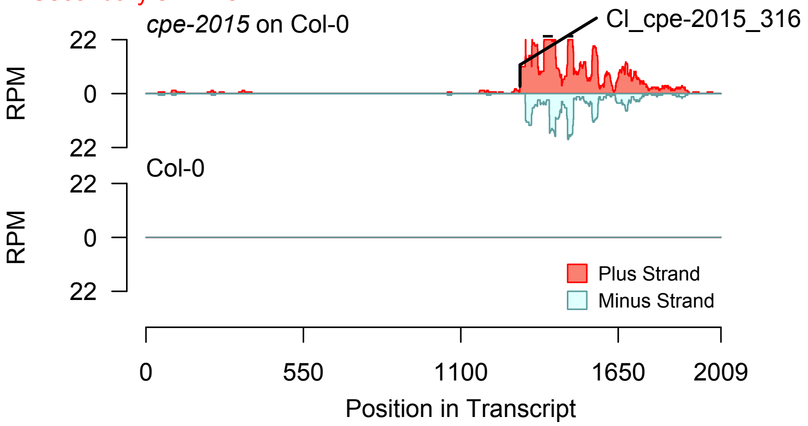

2nd siRNAs: Phase diagram

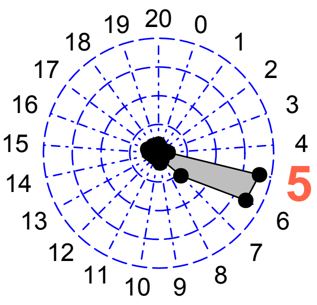

2nd siRNAs: Size distribution

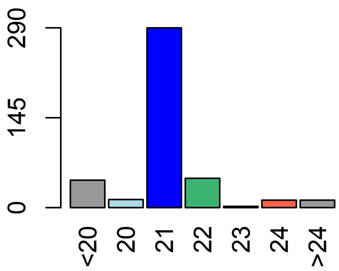

Degradome hits not found for sRNA

Allenscore: 3

Target Interaction:

```
5'- AUGGAUGGUAAGUUUUAUGUGA   AT5G60570.1
     ||||||||  |||:||||:||||
3'- UACCUACCUUUCGAAAUGCACU   CI_cpe-2017_305
```

Target Site: 1307

Superfamily: SupFam_281

miRNAin ccm: Yes

Published name: N/A

Confirmed Targeting

2nd-siRNA	*NanoPARE*
ccm	*ccm*
cpe-2015	*cpe-2015*
cpe-2017	
cgr-dp	*cgr-dp*
cgr-pm	
cgr-mass	
cin	*cin*

Galactose_oxidase_superfamily_protein

AT5G60570 - CI_cpe-2017_305

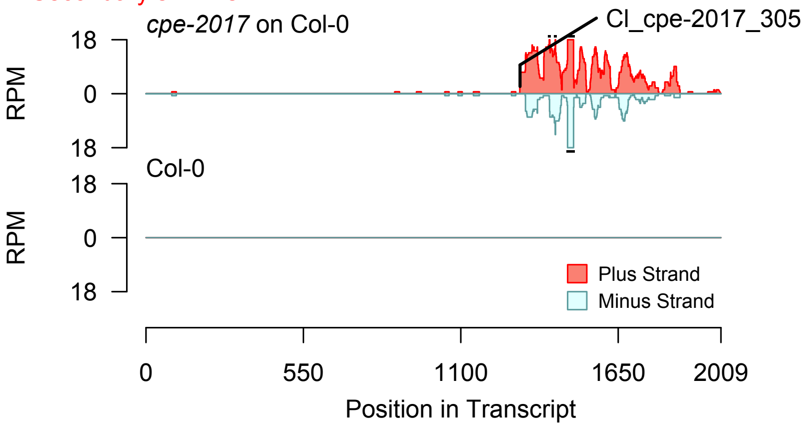

Degradome hits not found for sRNA

2nd siRNAs: Phase diagram

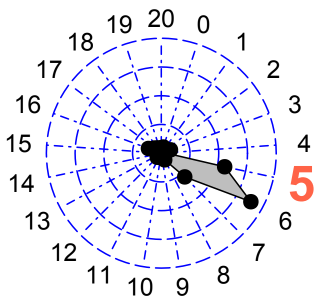

2nd siRNAs: Size distribution

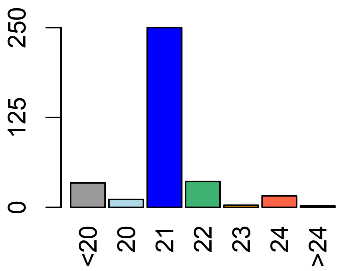

Allenscore: 2.5

Target Interaction:

```
5'- GAGGACUUCUAUAUCUUCACC   AT1G65800.1
     : | | | | | | | | | | | | | : | | | | | |
3'- UUCCUGAAGAUAUGGAAGUGU   Cl_cgr-dp_14631
```

Target Site: 2196

Superfamily: SupFam_49

miRNAin ccm: Yes

Published name: N/A

Confirmed Targeting

2nd-siRNA	*NanoPARE*
ccm	*ccm*
cpe-2015	*cpe-2015*
cpe-2017	
cgr-dp	*cgr-dp*
cgr-pm	
cgr-mass	
cin	*cin*

RK2

AT1G65800 - Cl_cgr-dp_14631

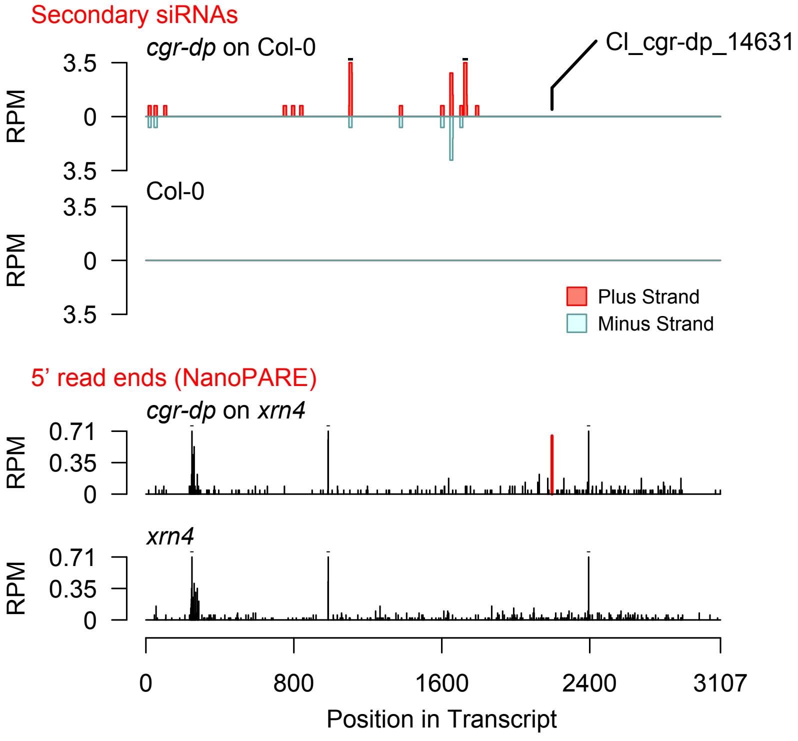

Diff. Exp. secondary siRNA locus not found

Allenscore: 2

Target Interaction:

5'- GGUUAUUUAGAUCCAGAGUA AT3G53840.1
: | | | | : | | | | | | | | | | | | | |
3'- UCAAUGAAUCUAGGUCUCAC CI_cpe-2015_45087

Target Site: 1583

Superfamily: SupFam_257

miRNAin ccm: N/A

Published name: N/A

Confirmed Targeting

2nd-siRNA	*NanoPARE*
ccm	*ccm*
cpe-2015	*cpe-2015*
cpe-2017	
cgr-dp	*cgr-dp*
cgr-pm	
cgr-mass	
cin	*cin*

Protein_kinase_superfamily_protein

AT3G53840 - CI_cpe-2015_45087

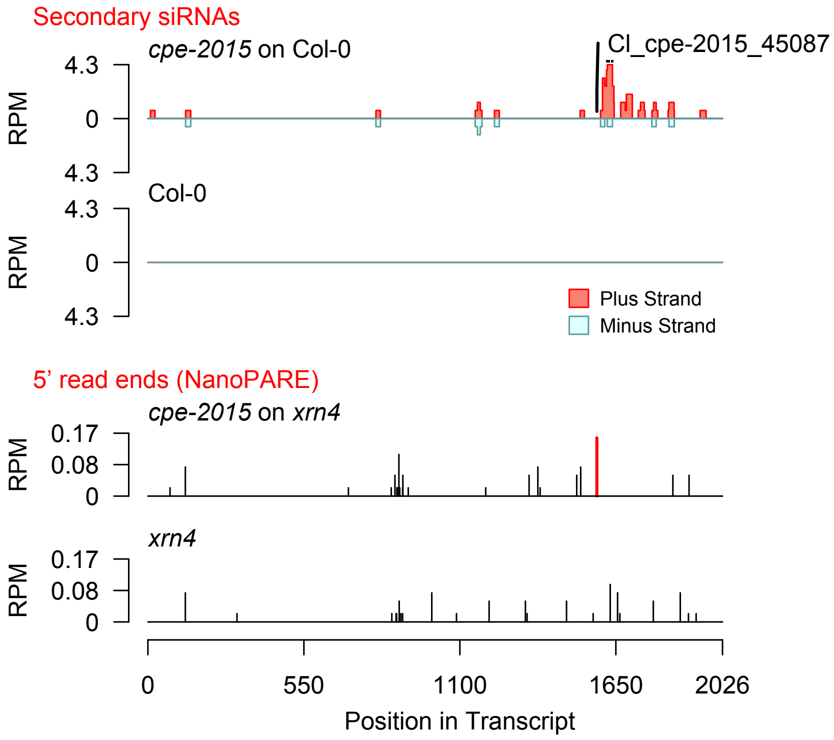

Diff. Exp. secondary siRNA locus not found

Allenscore: 1

Target Interaction:

```
5'- GCUGAAGCACAUGAACGACUUA    AT1G67310.1
     | |   | | | | | | | | | | | | | | | | | | |
3'- CG - CUUCGUGUACUUGCUGAAU    Cl_ccm_23
```

Target Site: 334

Superfamily: SupFam_39

miRNAin ccm: Yes

Published name: Not published

Confirmed Targeting

2nd-siRNA	*NanoPARE*
ccm	*ccm*
cpe-2015	*cpe-2015*
cpe-2017	
cgr-dp	*cgr-dp*
cgr-pm	
cgr-mass	
cin	*cin*

Calmodulin-binding_transcription_activat

AT1G67310 - Cl_ccm_23

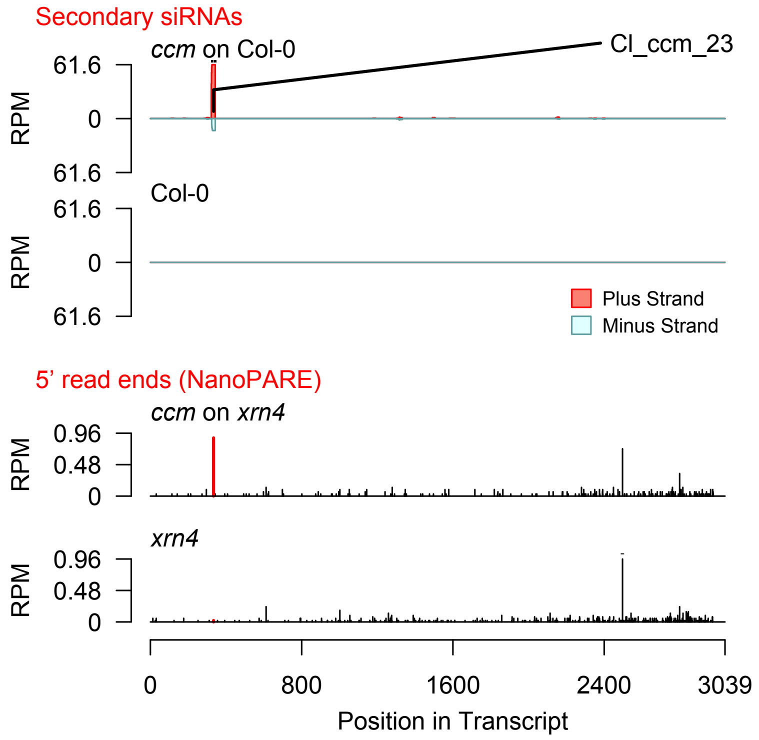

Diff. Exp. secondary siRNA locus not found

Allenscore: 4

Target Interaction:

```
5'- UGCUGAAGCACAUGAACGACUUA   AT1G67310.1
      ||  |||||||||||||| |||||
3'- UCG-CUUCGUGUACUUGGUGAAU   Cl_cgr-dp_47
```

Target Site: 334

Superfamily: SupFam_39

miRNAin ccm: Yes

Published name: N/A

Confirmed Targeting

2nd-siRNA	*NanoPARE*
ccm	*ccm*
cpe-2015	*cpe-2015*
cpe-2017	
cgr-dp	*cgr-dp*
cgr-pm	
cgr-mass	
cin	*cin*

Calmodulin-binding_transcription_activat

AT1G67310 - Cl_cgr-dp_47

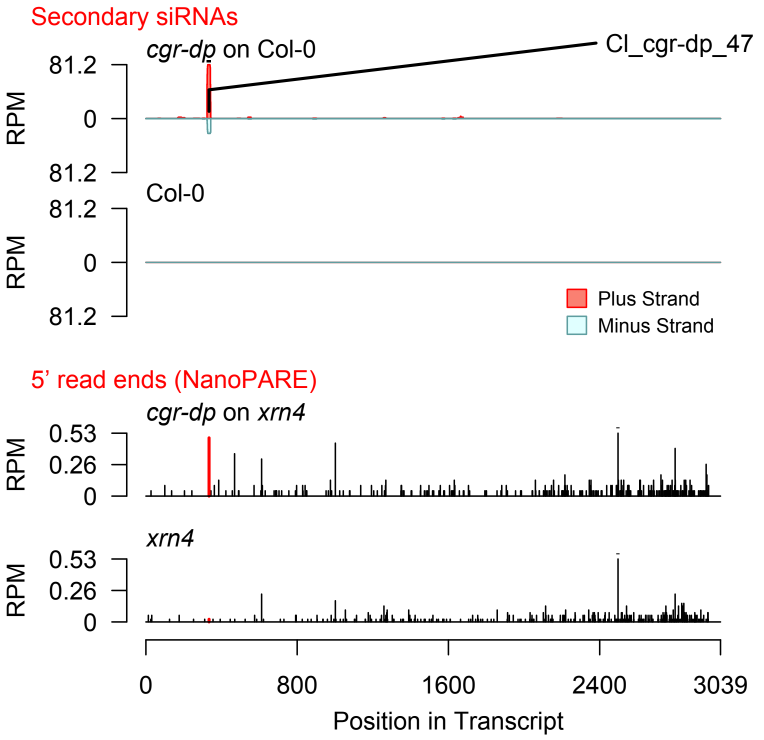

Diff. Exp. secondary siRNA locus not found

Allenscore: 3.5

Target Interaction:

```
5'- AUGGAUGUUAUCUGCCAAGCA   AT5G11200.1
    | | | | | | | | : | | | : : | | | | | |
3'- UACCUACAGUAGGUGGUUCGA   Cl_cin_42282
```

Target Site: 359

Superfamily: SupFam_1585

miRNAin ccm: N/A

Published name: N/A

Confirmed Targeting

2nd-siRNA	*NanoPARE*
ccm	*ccm*
cpe-2015	*cpe-2015*
cpe-2017	
cgr-dp	*cgr-dp*
cgr-pm	
cgr-mass	
cin	*cin*

UAP56b

AT5G11200 - Cl_cin_42282

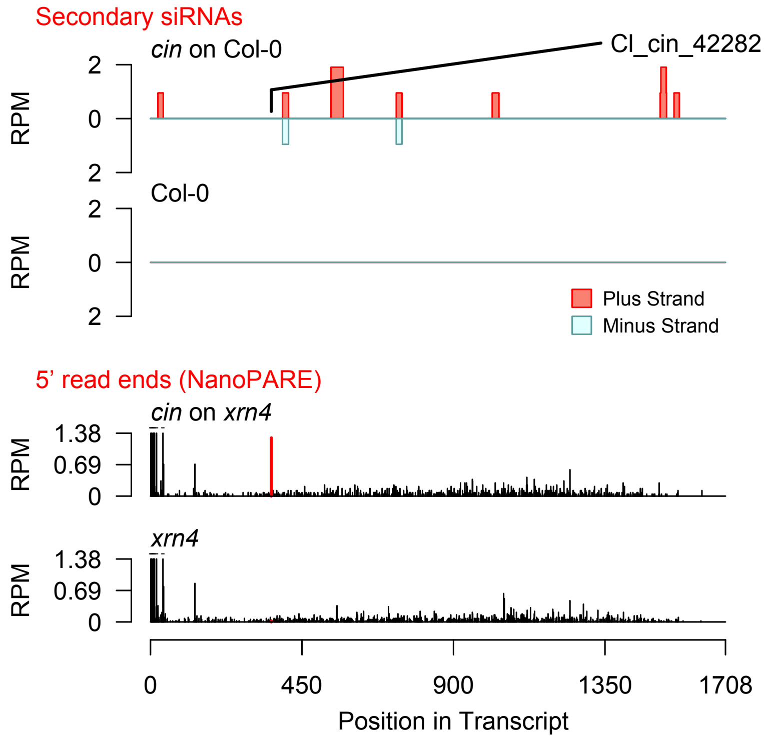

Diff. Exp. secondary siRNA locus not found

Allenscore: 1

Target Interaction:

```
5'- CUAGUCUACGAGUUUAUGCAAA    AT2G39660.1
    |||||||||||:||||||||||
3'- GAUCAGAUGCUUAAAUACGUUU    CI_ccm_7
```

Target Site: 870

Superfamily: SupFam_1

miRNAin ccm: Yes

Published name: ccm-MIR12463b

Confirmed Targeting

2nd-siRNA	*NanoPARE*
ccm	*ccm*
cpe-2015	*cpe-2015*
cpe-2017	
cgr-dp	*cgr-dp*
cgr-pm	
cgr-mass	
cin	*cin*

BIK1

AT2G39660 - CI_ccm_7

Secondary siRNAs

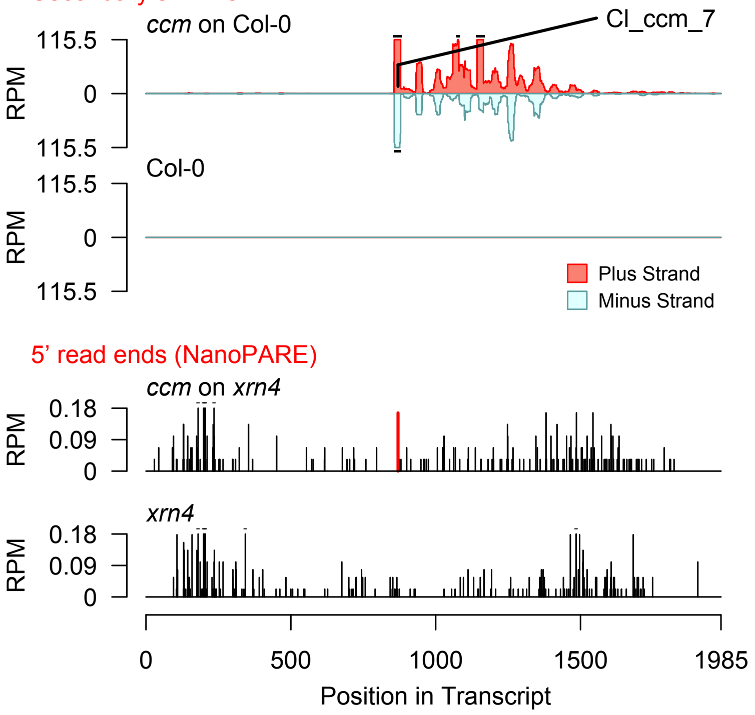

2nd siRNAs: Phase diagram

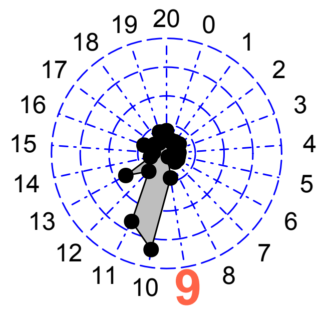

2nd siRNAs: Size distribution

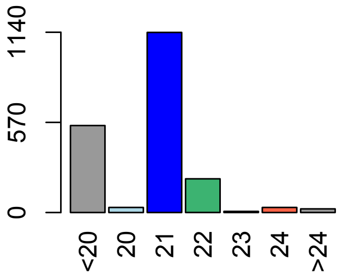

Allenscore: 2

Target Interaction:

```
5'- AGUCUACGAGUUUAUGCAAA   AT2G39660.1
      | | | | | | | | : | | | | | | | | | | |
3'- CCAGAUGCUUAAAUACGUUU   CI_ccm_7146
```

Target Site: 870

Superfamily: SupFam_1

miRNAin ccm: Yes

Published name: Not published

Confirmed Targeting

2nd-siRNA	*NanoPARE*
ccm	*ccm*
cpe-2015	*cpe-2015*
cpe-2017	
cgr-dp	*cgr-dp*
cgr-pm	
cgr-mass	
cin	*cin*

BIK1

AT2G39660 - CI_ccm_7146

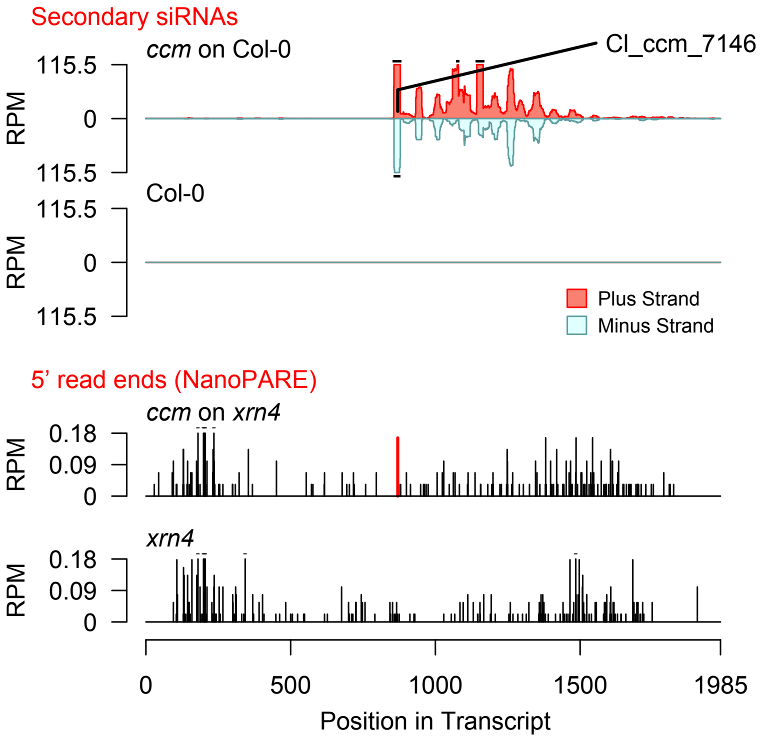

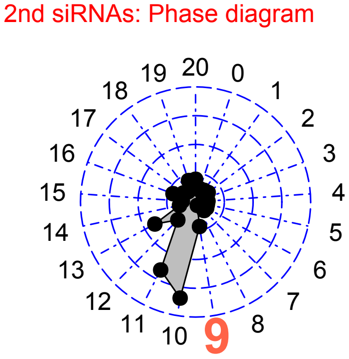

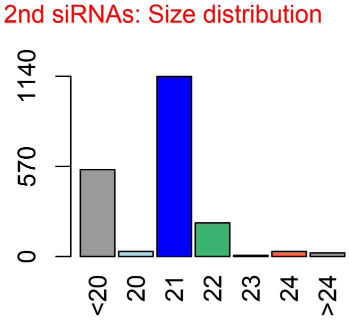

Allenscore: 2

Target Interaction:

```
5'- UAUGGGUACGCCGCGCCUGAGUA   AT2G39660.1
     ||||  |||||||||||||||||
3'- UUACCGAUGCGGCGCGGACUCAU   Cl_ccm_117
```

Target Site: 1156

Superfamily: SupFam_190

miRNAin ccm: Yes

Published name: Not published

Confirmed Targeting

2nd-siRNA	*NanoPARE*
ccm	*ccm*
cpe-2015	*cpe-2015*
cpe-2017	
cgr-dp	*cgr-dp*
cgr-pm	
cgr-mass	
cin	*cin*

BIK1

AT2G39660 - Cl_ccm_117

Secondary siRNAs

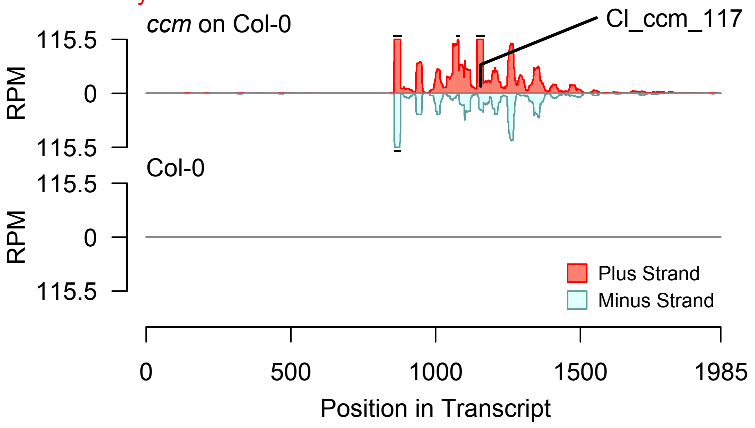

2nd siRNAs: Phase diagram

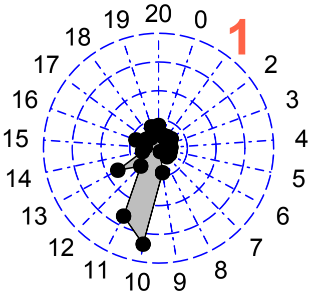

2nd siRNAs: Size distribution

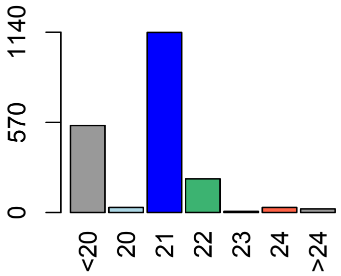

Degradome hits not found for sRNA

Allenscore: 1

Target Interaction:

```
5'- GGGUACGCCGCGCCUGAGUA   AT2G39660.1
     ||  |||||||||||||||||
3'- CCGAUGCGGCGCGGACUCAU   Cl_ccm_23740
```

Target Site: 1156

Superfamily: SupFam_190

miRNAin ccm: Yes

Published name: Not published

Confirmed Targeting

2nd-siRNA	*NanoPARE*
ccm	*ccm*
cpe-2015	*cpe-2015*
cpe-2017	
cgr-dp	*cgr-dp*
cgr-pm	
cgr-mass	
cin	*cin*

BIK1

AT2G39660 - Cl_ccm_23740

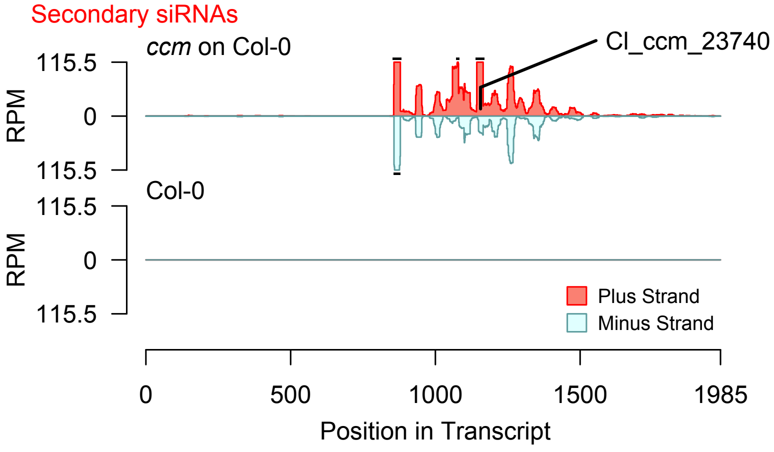

2nd siRNAs: Phase diagram

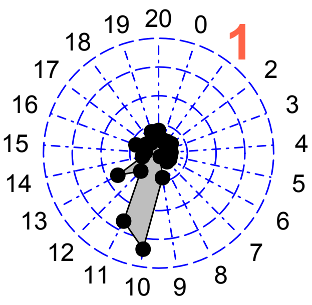

2nd siRNAs: Size distribution

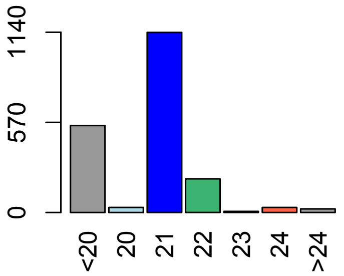

Degradome hits not found for sRNA

Allenscore: 1

Target Interaction:

```
5'- CUAGUCUACGAGUUUAUGCAAA   AT2G39660.1
    |||||||||||:||||||||||
3'- GAUCAGAUGCUUAAAUACGUUU   CI_cpe-2015_5
```

Target Site: 870

Superfamily: SupFam_1

miRNAin ccm: Yes

Published name: N/A

Confirmed Targeting

2nd-siRNA	*NanoPARE*
ccm	*ccm*
cpe-2015	*cpe-2015*
cpe-2017	
cgr-dp	*cgr-dp*
cgr-pm	
cgr-mass	
cin	*cin*

BIK1

AT2G39660 - CI_cpe-2015_5

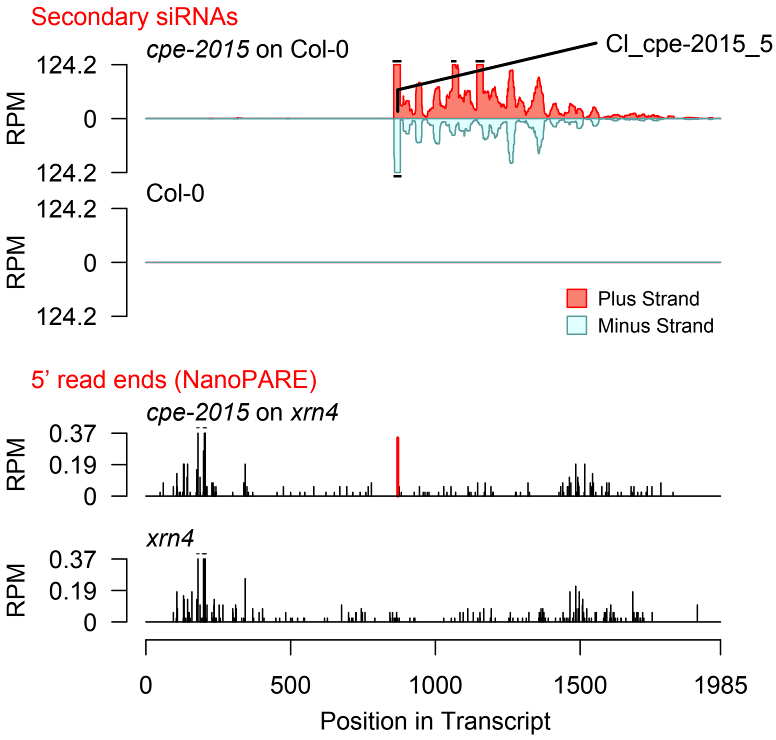

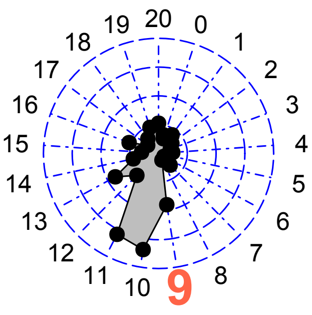

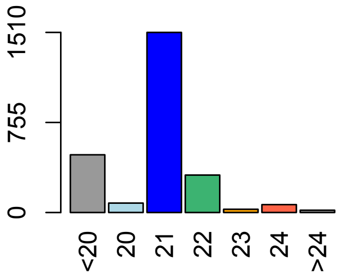

Allenscore: 2

Target Interaction:

```
5'- AGUCUACGAGUUUAUGCAAA    AT2G39660.1
      ||||||||:||||||||||
3'- CCAGAUGCUUAAAUACGUUU    CI_cpe-2015_4852
```

Target Site: 870

Superfamily: SupFam_1

miRNAin ccm: Yes

Published name: N/A

Confirmed Targeting

2nd-siRNA	*NanoPARE*
ccm	*ccm*
cpe-2015	*cpe-2015*
cpe-2017	
cgr-dp	*cgr-dp*
cgr-pm	
cgr-mass	
cin	*cin*

BIK1

AT2G39660 - CI_cpe-2015_4852

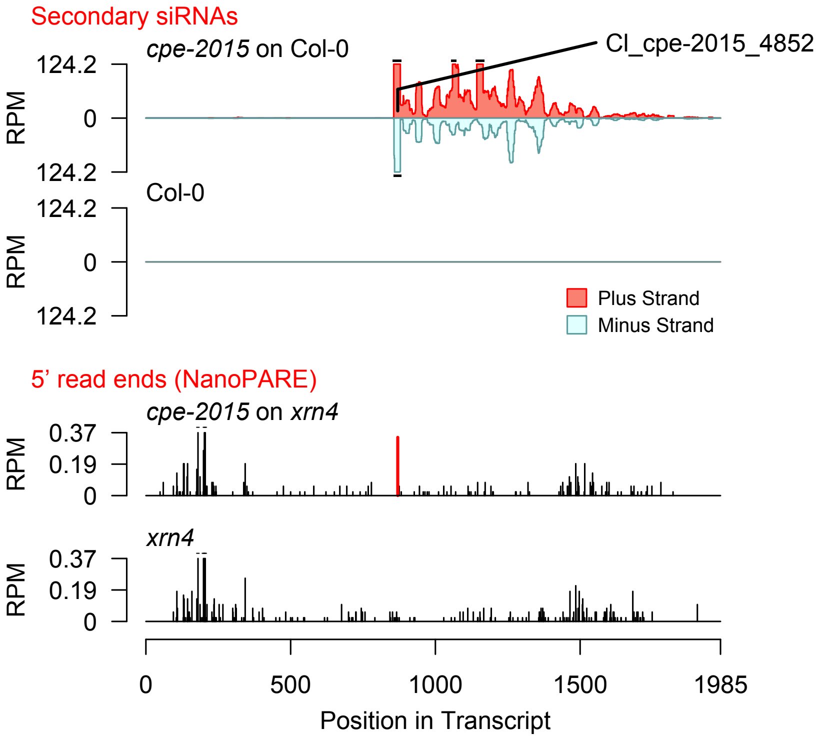

2nd siRNAs: Phase diagram

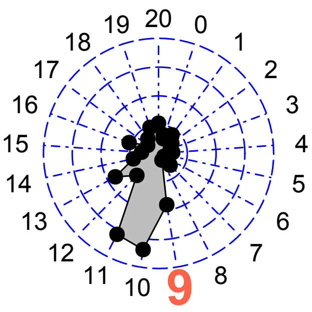

2nd siRNAs: Size distribution

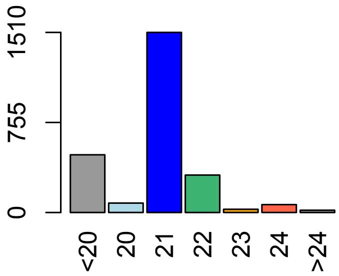

Allenscore: 2

Target Interaction:

```
5'- UAUGGGUACGCCGCGCCUGAGUA   AT2G39660.1
       ||||  ||||||||||||||||||
3'- UUACCGAUGCGGCGCGGACUCAU   Cl_cpe-2015_953
```

Target Site: 1156

Superfamily: SupFam_190

miRNAin ccm: Yes

Published name: N/A

Confirmed Targeting

2nd-siRNA	*NanoPARE*
ccm	*ccm*
cpe-2015	*cpe-2015*
cpe-2017	
cgr-dp	*cgr-dp*
cgr-pm	
cgr-mass	
cin	*cin*

BIK1

AT2G39660 - Cl_cpe-2015_953

Secondary siRNAs

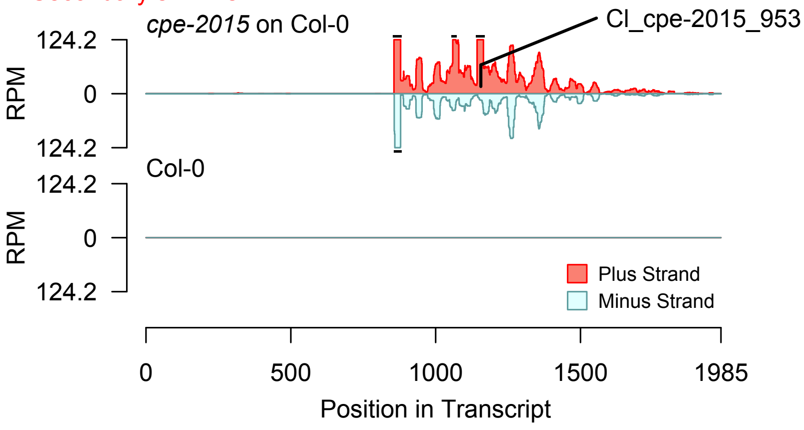

2nd siRNAs: Phase diagram

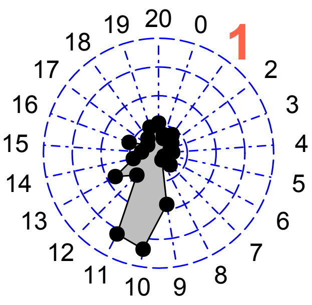

2nd siRNAs: Size distribution

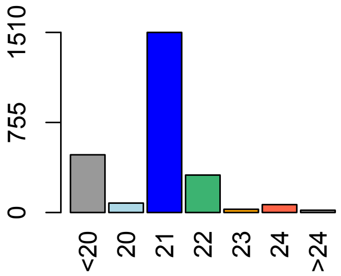

Degradome hits not found for sRNA

Allenscore: 1

Target Interaction:

```
5'- CUAGUCUACGAGUUUAUGCAAA    AT2G39660.1
     |||||||||||:||||||||||
3'- GAUCAGAUGCUUAAAUACGUUU    Cl_cpe-2017_9
```

Target Site: 870

Superfamily: SupFam_1

miRNAin ccm: Yes

Published name: N/A

Confirmed Targeting

2nd-siRNA	*NanoPARE*
ccm	*ccm*
cpe-2015	*cpe-2015*
cpe-2017	
cgr-dp	*cgr-dp*
cgr-pm	
cgr-mass	
cin	*cin*

BIK1

AT2G39660 - Cl_cpe-2017_9

Secondary siRNAs

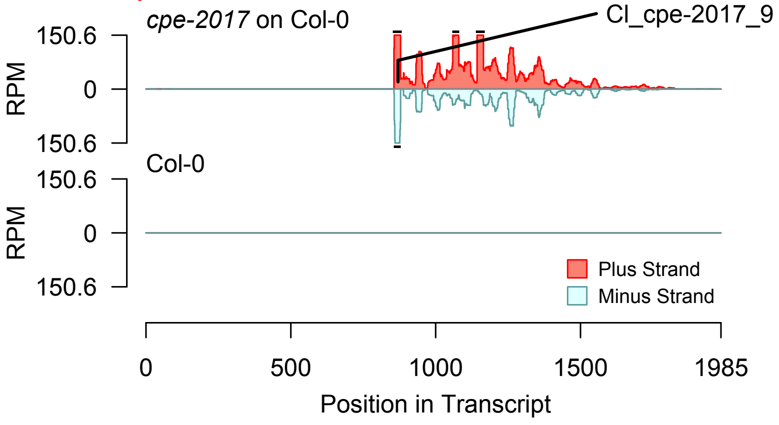

2nd siRNAs: Phase diagram

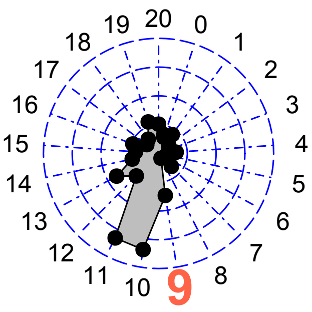

2nd siRNAs: Size distribution

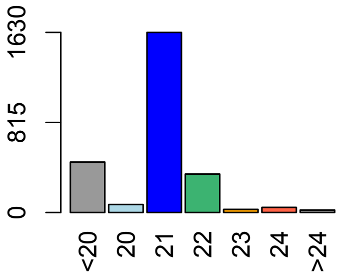

Degradome hits not found for sRNA

Allenscore: 2

Target Interaction:

```
5'- AGUCUACGAGUUUAUGCAAA   AT2G39660.1
     | | | | | | | | : | | | | | | | | | |
3'- CCAGAUGCUUAAAUACGUUU   Cl_cpe-2017_5671
```

Target Site: 870

Superfamily: SupFam_1

miRNAin ccm: Yes

Published name: N/A

Confirmed Targeting

2nd-siRNA	*NanoPARE*
ccm	*ccm*
cpe-2015	*cpe-2015*
cpe-2017	
cgr-dp	*cgr-dp*
cgr-pm	
cgr-mass	
cin	*cin*

BIK1

AT2G39660 - Cl_cpe-2017_5671

Secondary siRNAs

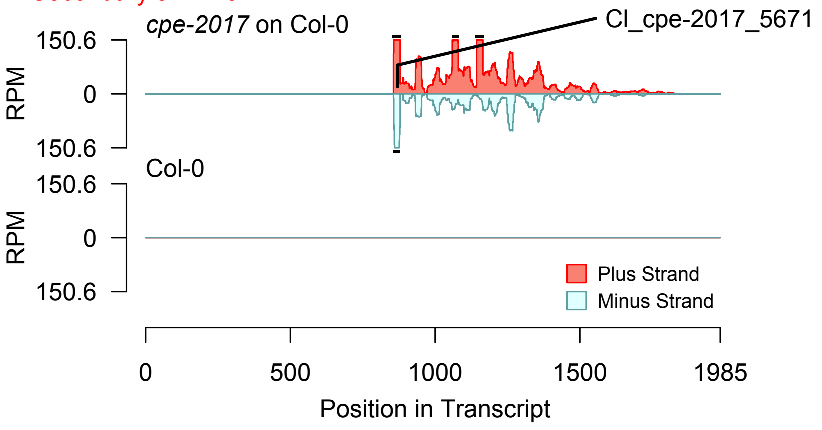

Degradome hits not found for sRNA

2nd siRNAs: Phase diagram

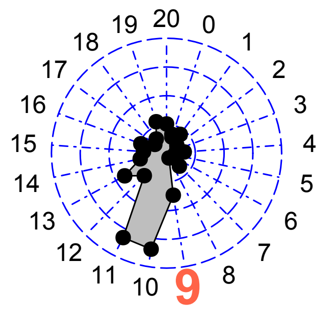

2nd siRNAs: Size distribution

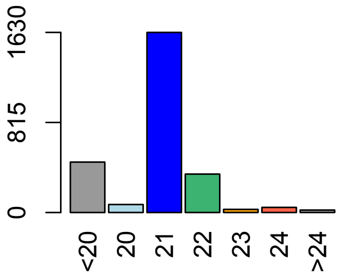

Allenscore: 2

Target Interaction:

```
5'- UAUGGGUACGCCGCGCCUGAGUA   AT2G39660.1
      ||||  ||||||||||||||||||
3'- UUACCGAUGCGGCGCGGACUCAU   CI_cpe-2017_695
```

Target Site: 1156

Superfamily: SupFam_190

miRNAin ccm: Yes

Published name: N/A

Confirmed Targeting

2nd-siRNA	*NanoPARE*
ccm	*ccm*
cpe-2015	*cpe-2015*
cpe-2017	
cgr-dp	*cgr-dp*
cgr-pm	
cgr-mass	
cin	*cin*

BIK1

AT2G39660 - CI_cpe-2017_695

Secondary siRNAs

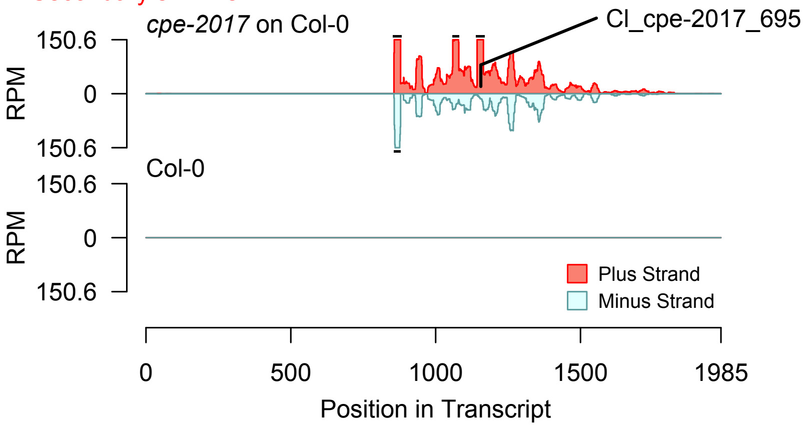

2nd siRNAs: Phase diagram

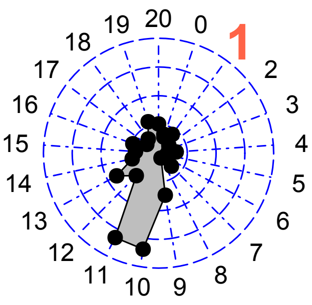

2nd siRNAs: Size distribution

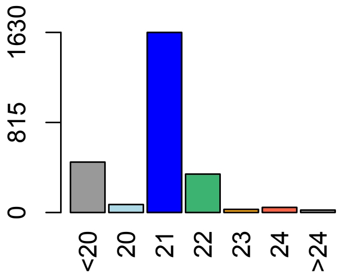

Degradome hits not found for sRNA

Allenscore: 4.5

Target Interaction:

```
5'- UUGUUUAUGAAAGGGAAUACA   AT1G10900.1
      | : | | | | | | | | | : | | | | | |
3'- UUCGAAUACUUUCUCUUAUGA   Cl_cgr-dp_539
```

Target Site: 1185

Superfamily: SupFam_370

miRNAin ccm: N/A

Published name: N/A

Confirmed Targeting

2nd-siRNA	*NanoPARE*
ccm	*ccm*
cpe-2015	*cpe-2015*
cpe-2017	
cgr-dp	*cgr-dp*
cgr-pm	
cgr-mass	
cin	*cin*

Phosphatidylinositol-4-phosphate_5-kinas

AT1G10900 - Cl_cgr-dp_539

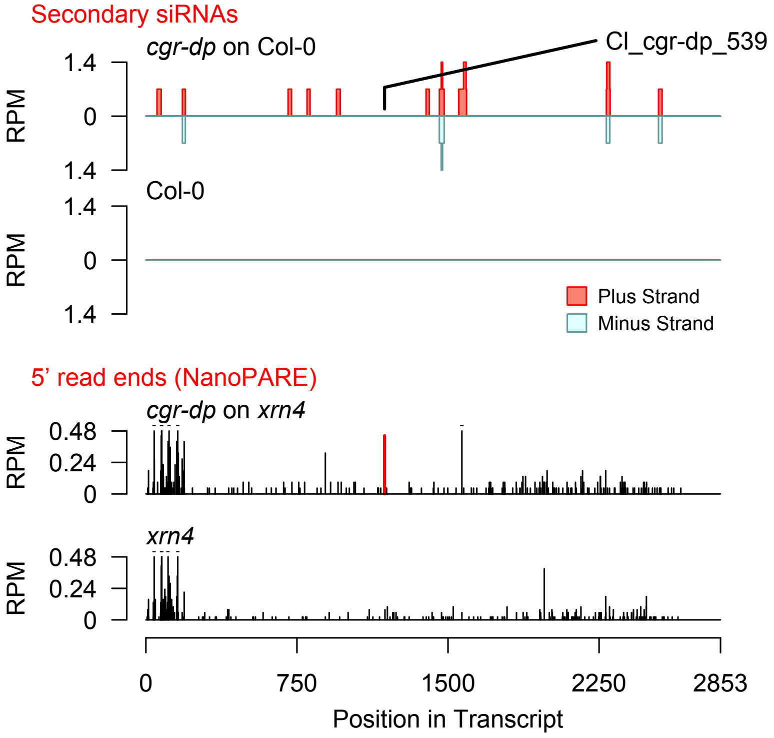

Diff. Exp. secondary siRNA locus not found

Allenscore: 4.5

Target Interaction:

```
5'- AGUGAGGAACUUAGAGGACUCG   AT3G09830.1
     | :   : | | : | | | | | | | | | | | | | | :
3'- UUCUUCUUUGAAUCUCCUGAGU   CI_cgr-dp_42
```

Target Site: 689

Superfamily: SupFam_81

miRNAin ccm: N/A

Published name: N/A

Confirmed Targeting

2nd-siRNA	*NanoPARE*
ccm	*ccm*
cpe-2015	*cpe-2015*
cpe-2017	
cgr-dp	*cgr-dp*
cgr-pm	
cgr-mass	
cin	*cin*

PCRK1

AT3G09830 - CI_cgr-dp_42

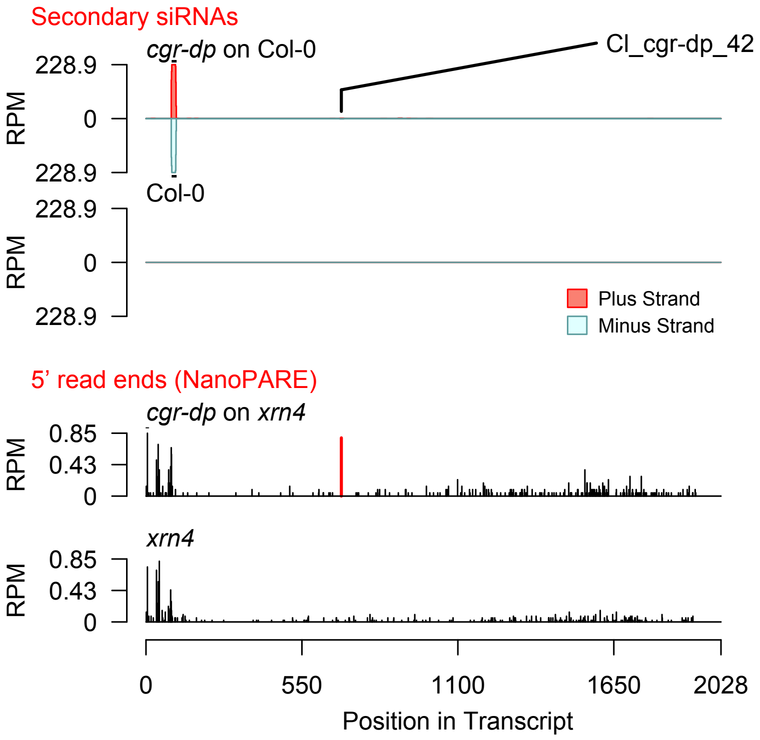

Diff. Exp. secondary siRNA locus not found

Allenscore: 4

Target Interaction:

```
5'- UGAGCUACUUACACCAAAAUA   AT4G38470.1
     | : | |   | | | :   | | | | | | | | | | |
3'- AUUCUAUGGUUGUGGUUUUAU   CI_cin_14840
```

Target Site: 1743

Superfamily: SupFam_189

miRNAin ccm: N/A

Published name: N/A

Confirmed Targeting

2nd-siRNA	*NanoPARE*
ccm	*ccm*
cpe-2015	*cpe-2015*
cpe-2017	
cgr-dp	*cgr-dp*
cgr-pm	
cgr-mass	
cin	*cin*

STY46

AT4G38470 - CI_cin_14840

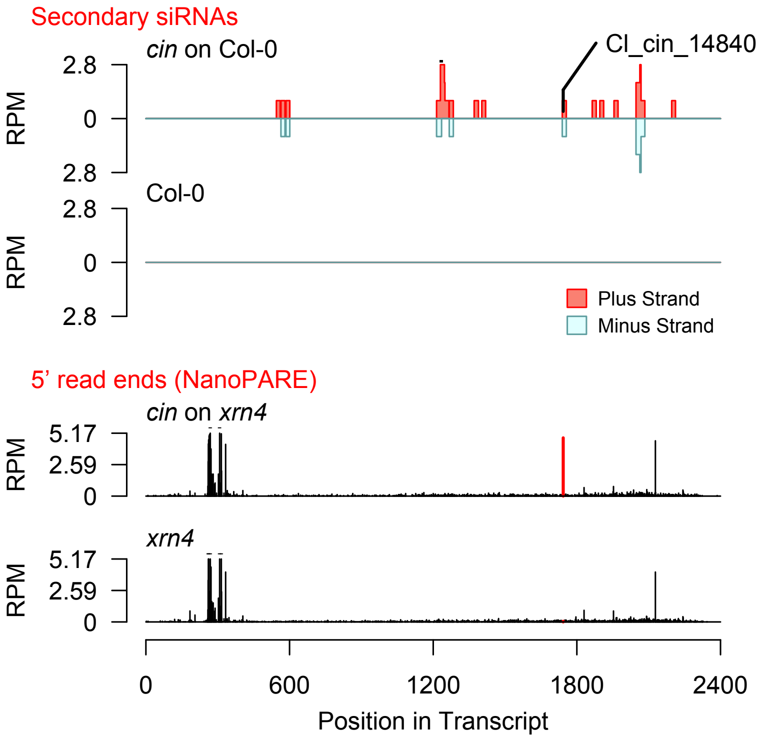

Diff. Exp. secondary siRNA locus not found

Allenscore: 3.5

Target Interaction:

```
5'- UGAGCUACUUACACCAAAAUA   AT4G38470.1
     | | | | | | | : |  | | | | | | | | | |
3'- CCUCGAUGGACGUGGUUUUAU   CI_cin_176
```

Target Site: 1743

Superfamily: SupFam_189

miRNAin ccm: N/A

Published name: N/A

Confirmed Targeting

2nd-siRNA	*NanoPARE*
ccm	*ccm*
cpe-2015	*cpe-2015*
cpe-2017	
cgr-dp	*cgr-dp*
cgr-pm	
cgr-mass	
cin	*cin*

STY46

AT4G38470 - CI_cin_176

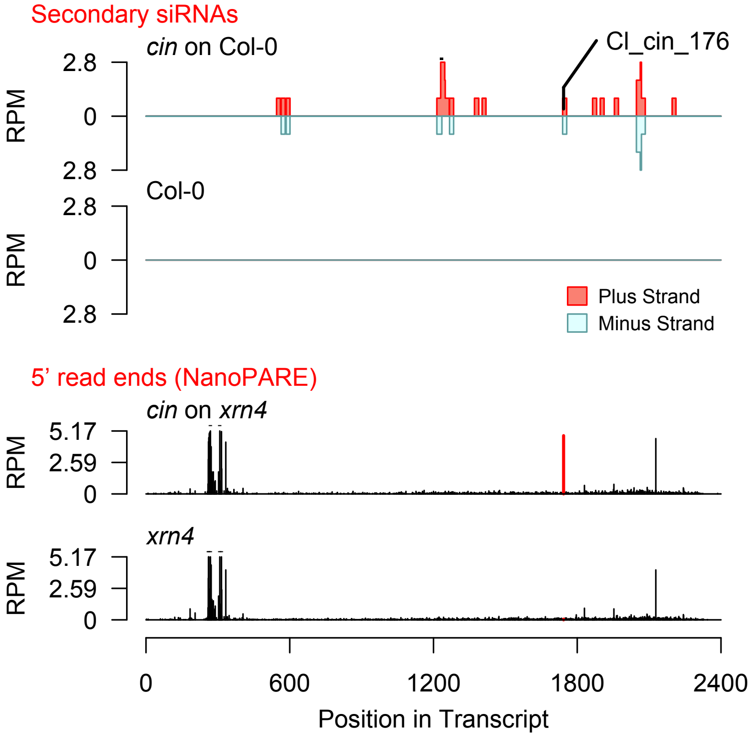

Diff. Exp. secondary siRNA locus not found

Allenscore: 4.5

Target Interaction:

```
5'- UGAGCUACUUACACCAAAAUA    AT4G38470.1
      | | | | | | | : | | | |   | | | | | : |
3'- UCUCGAUGGAUGUAGUUUUGU    Cl_cin_21889
```

Target Site: 1743

Superfamily: SupFam_189

miRNAin ccm: N/A

Published name: N/A

Confirmed Targeting

2nd-siRNA	*NanoPARE*
ccm	*ccm*
cpe-2015	*cpe-2015*
cpe-2017	
cgr-dp	*cgr-dp*
cgr-pm	
cgr-mass	
cin	*cin*

STY46

AT4G38470 - Cl_cin_21889

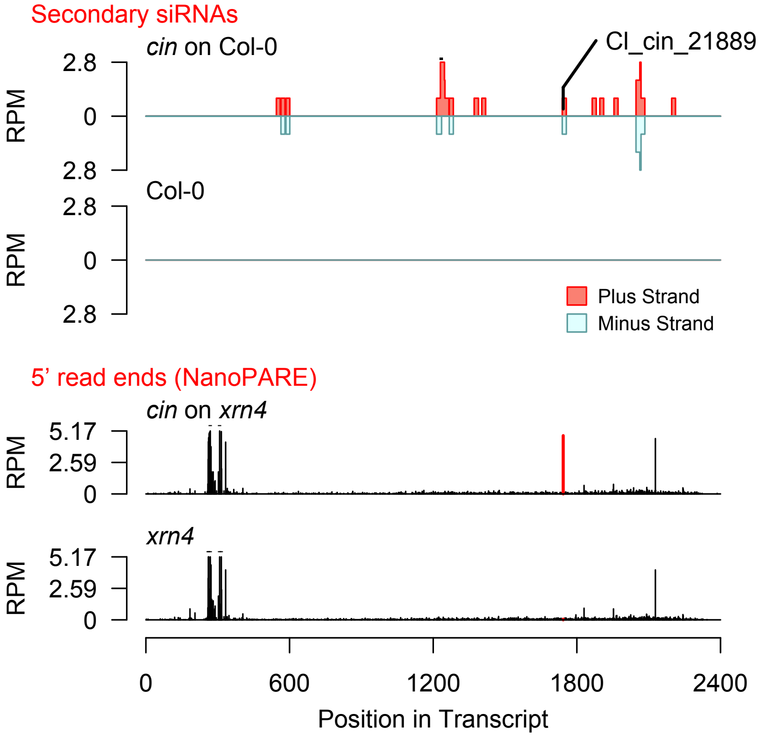

Diff. Exp. secondary siRNA locus not found
