# Supplementary material for: Compensatory sequence variation between trans-species small RNAs and their target sites
Source: eLife. 2019 Dec 17;8:e49750. doi: 10.7554/eLife.49750 (PMC6917502; doi:10.7554/eLife.49750)

AT5G66850 - MAPKKK5
36/36 homologs found in phytozome eudicots containing targetsite

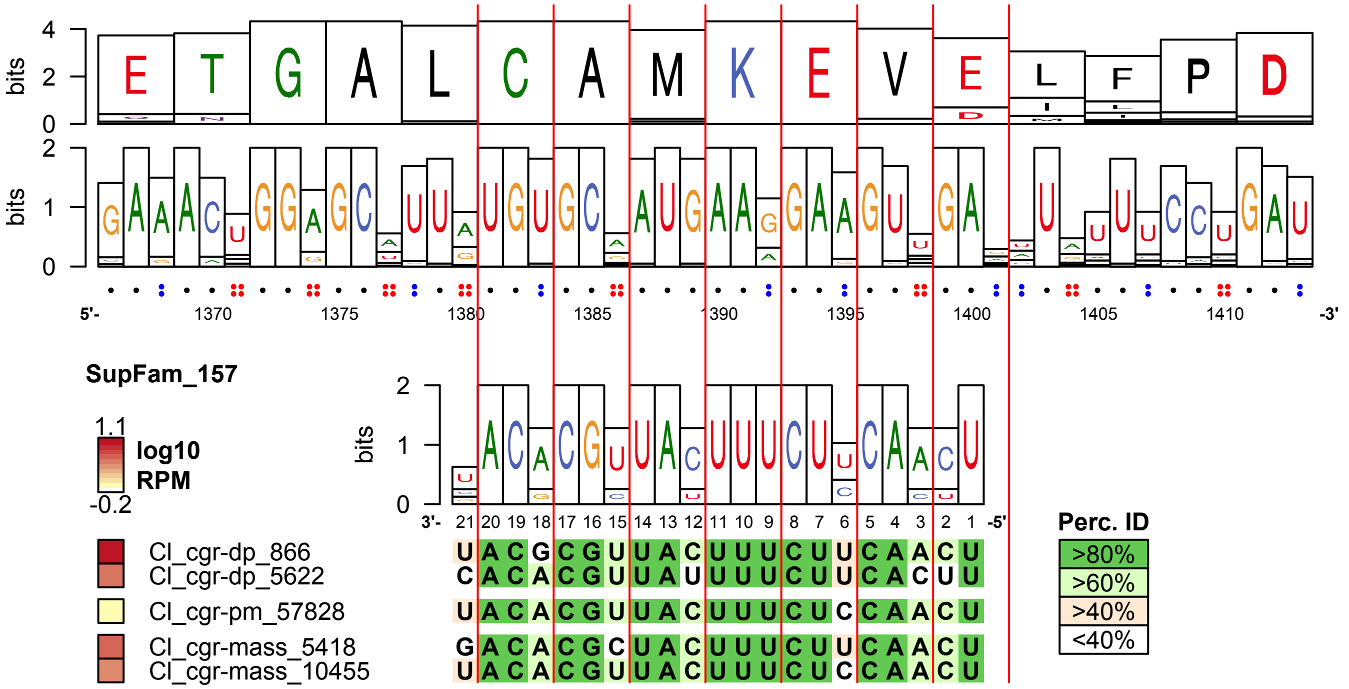

AT3G45640 - MPK3

35/36 homologs found in phytozome eudicots containing targetsite

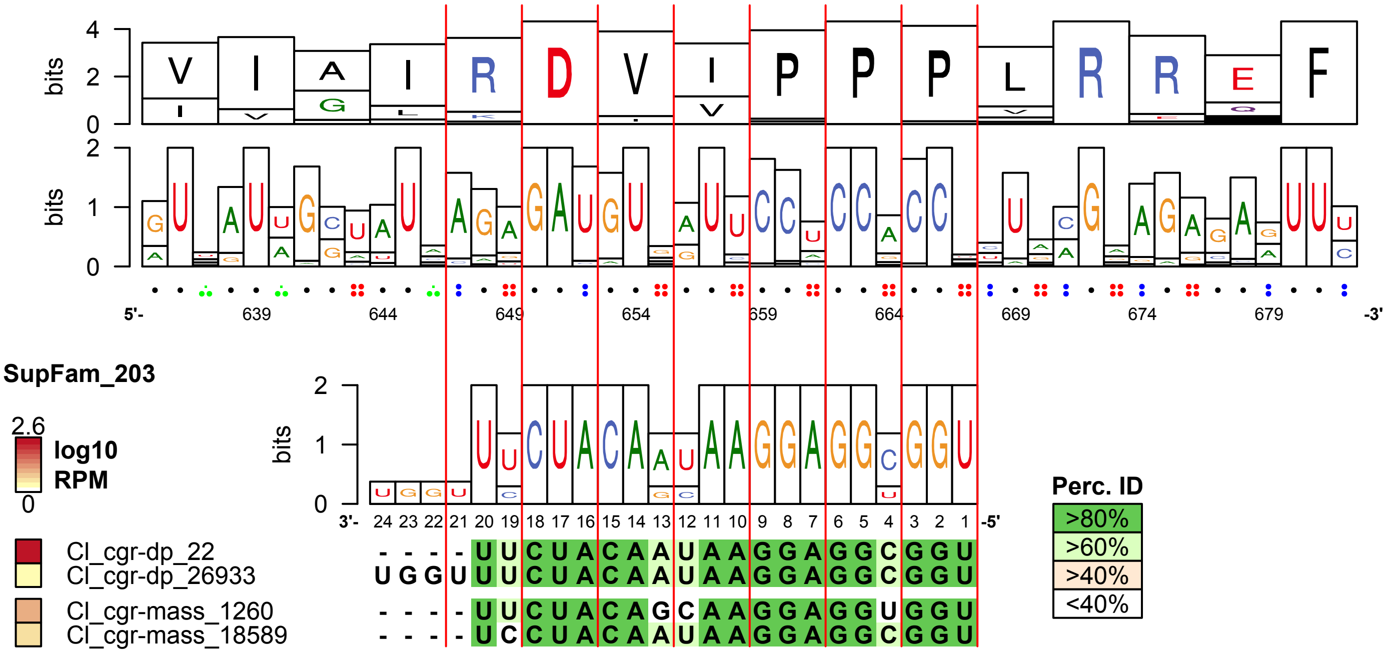

AT1G53440 - Leucine-rich_repeat_transmembrane_protein_kinase
36/36 homologs found in phytozome eudicots containing targetsite

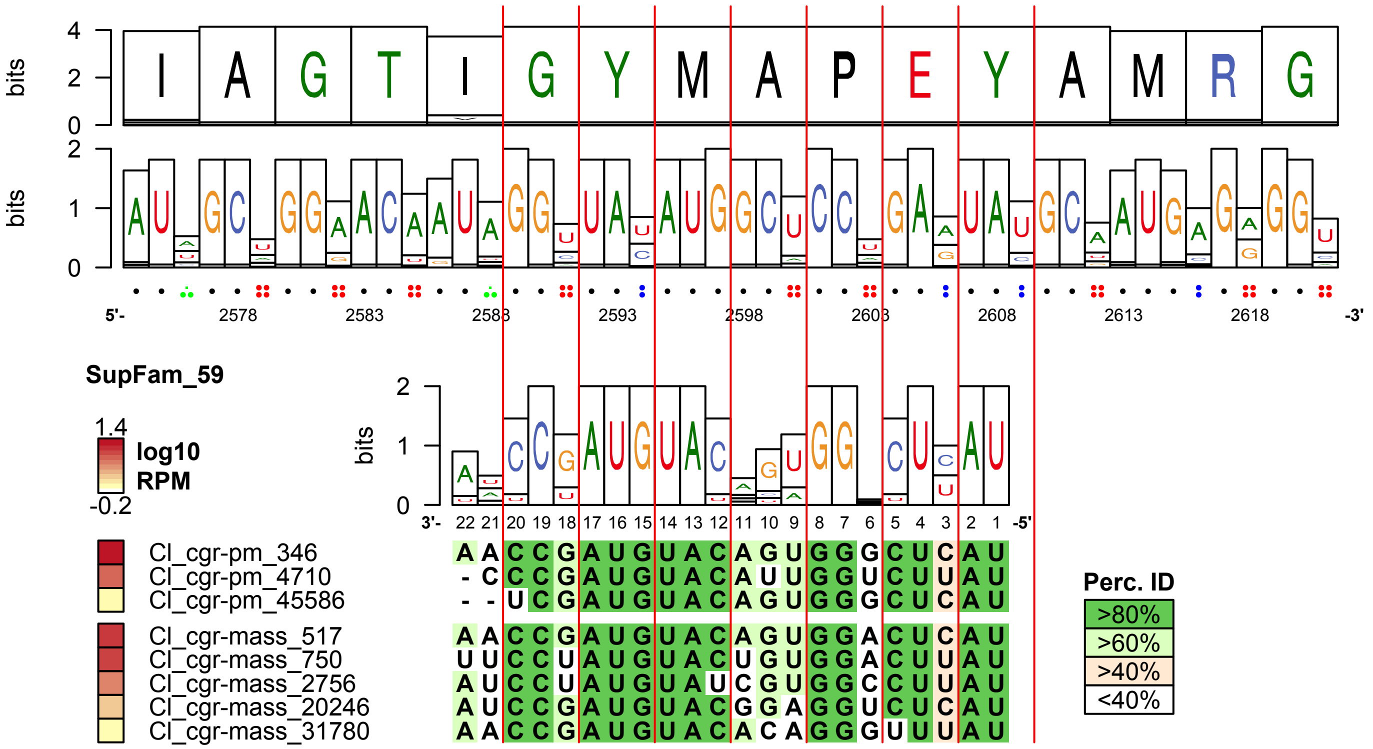

AT4G28490 - HAE

34/36 homologs found in phytozome eudicots containing targetsite

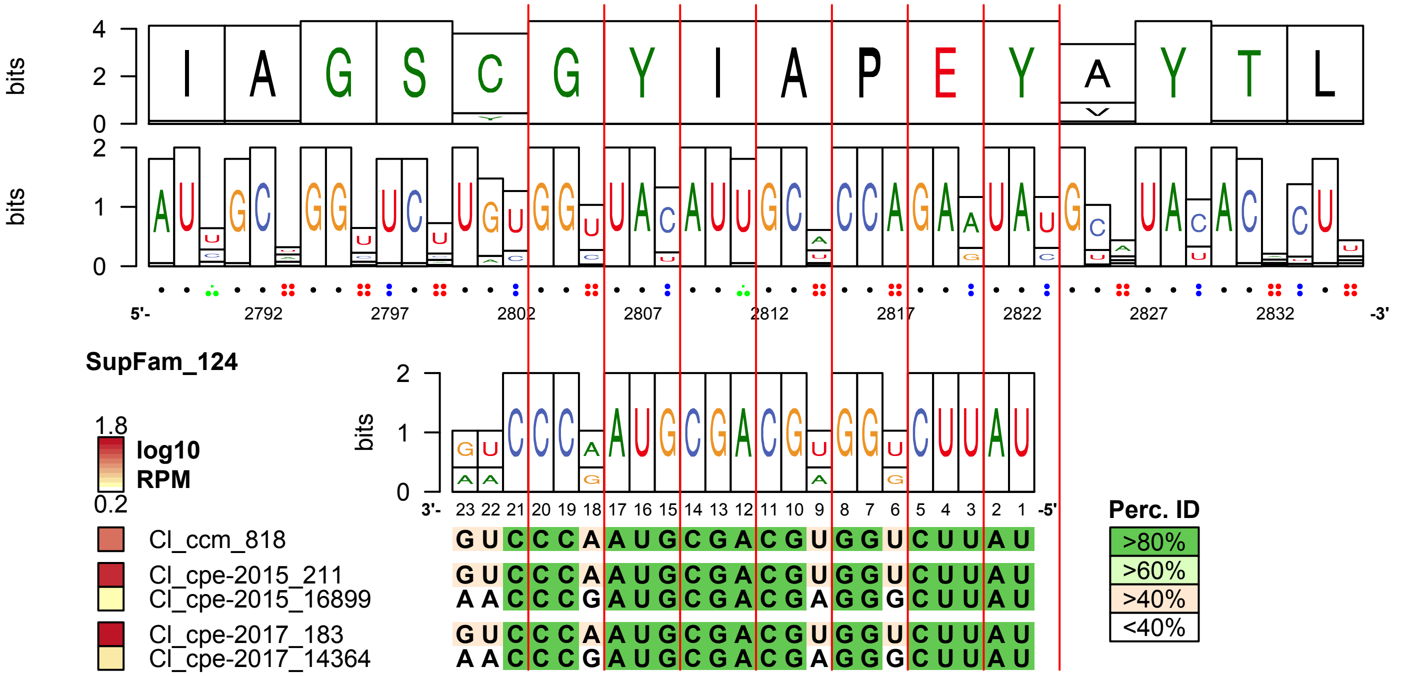

AT1G12820 - AFB3
36/36 homologs found in phytozome eudicots containing targetsite

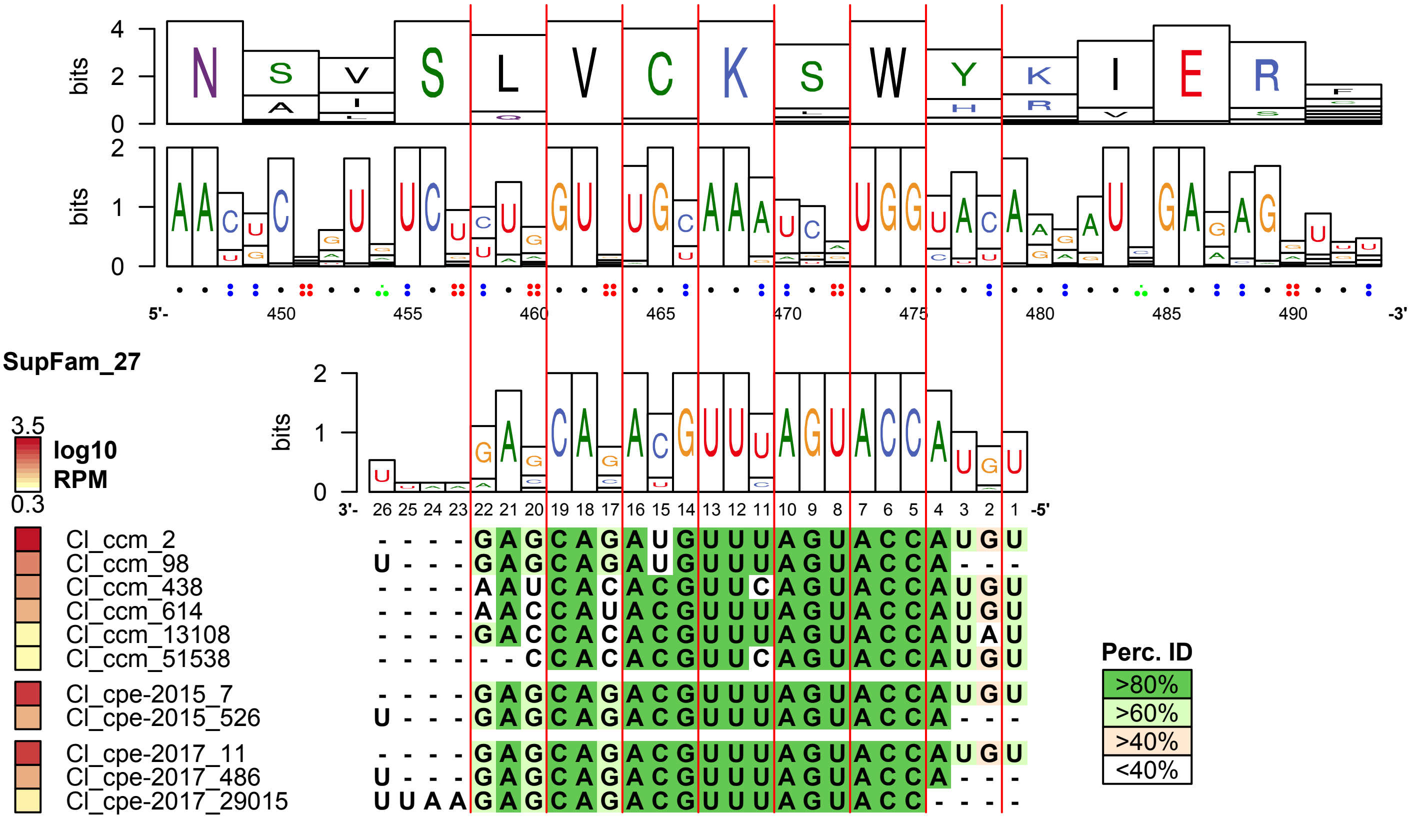

AT3G01680 - SEOR1
34/36 homologs found in phytozome eudicots containing targetsite

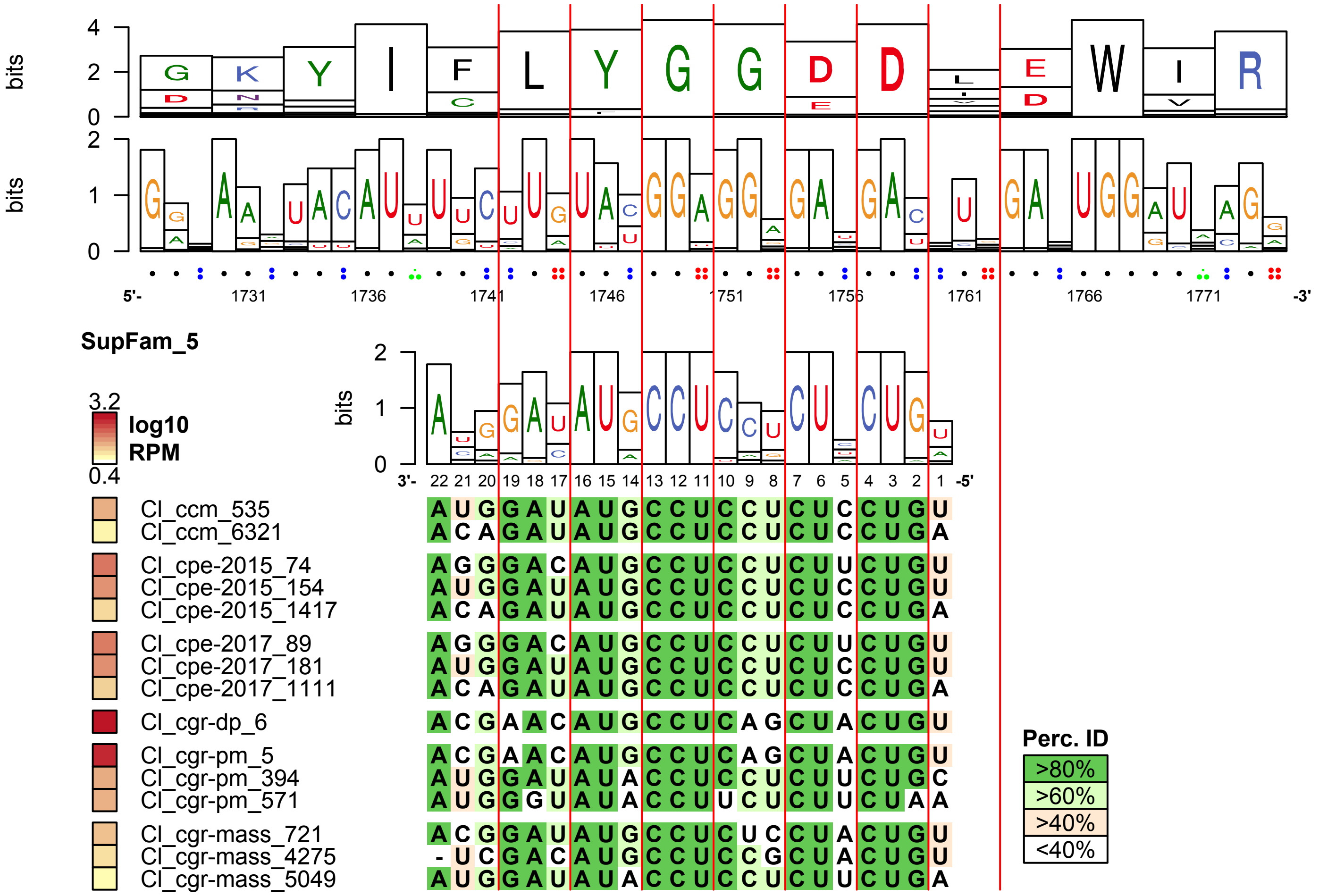

AT4G39400 - BRI1
36/36 homologs found in phytozome eudicots containing targetsite

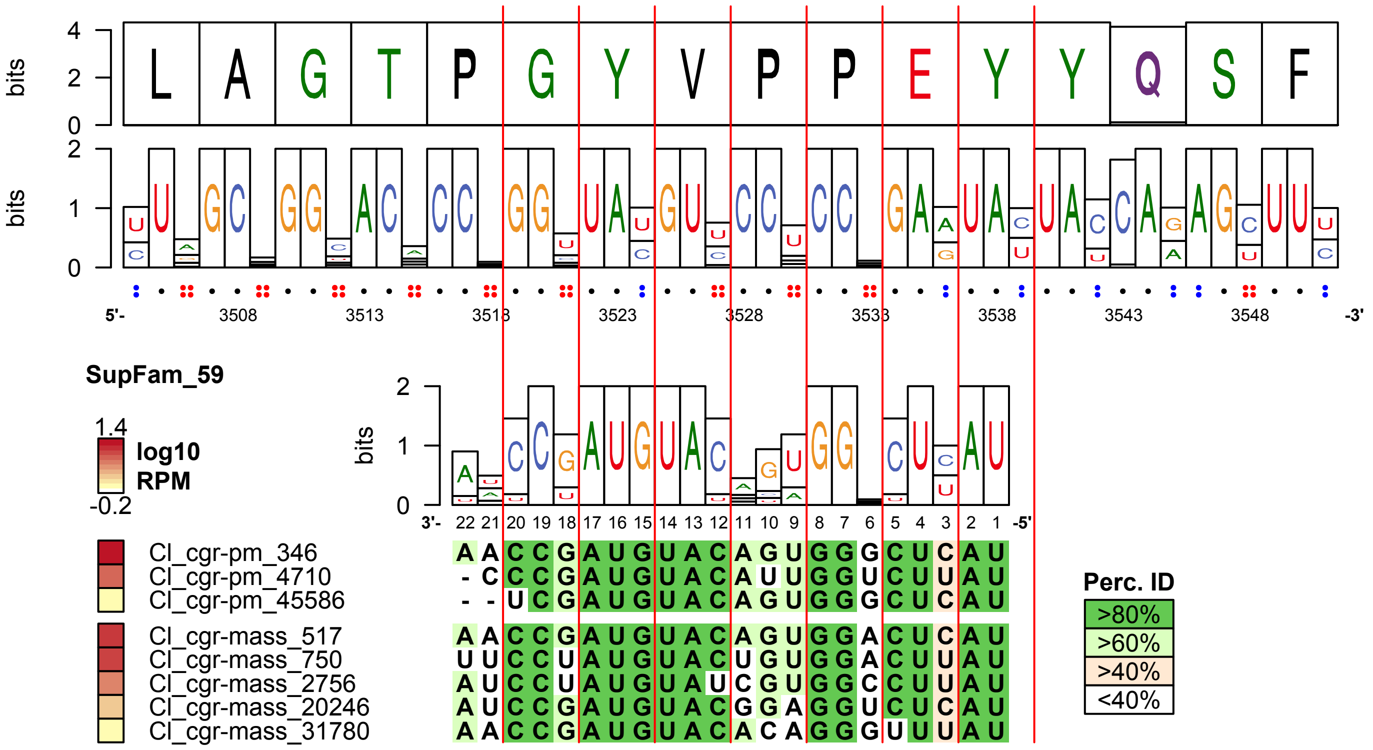

AT5G02290 - NAK

36/36 homologs found in phytozome eudicots containing targetsite

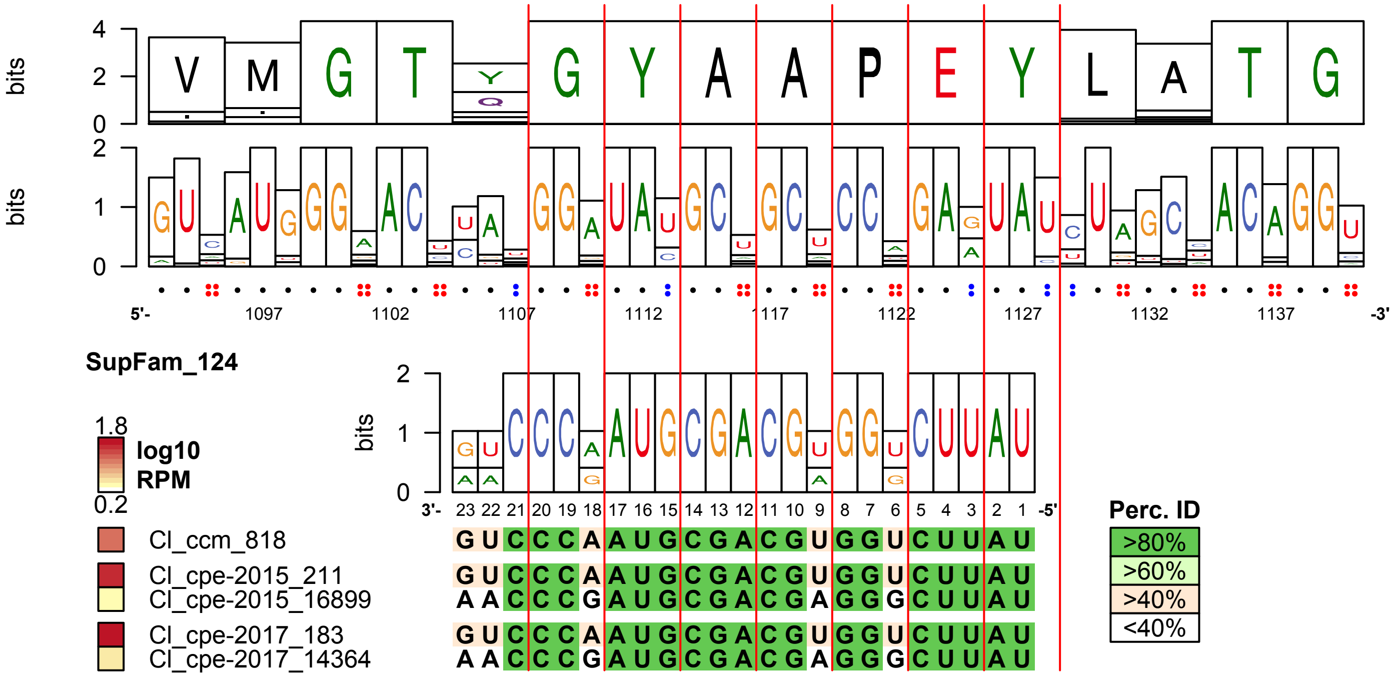

AT2G02800 - APK2B
33/36 homologs found in phytozome eudicots containing targetsite

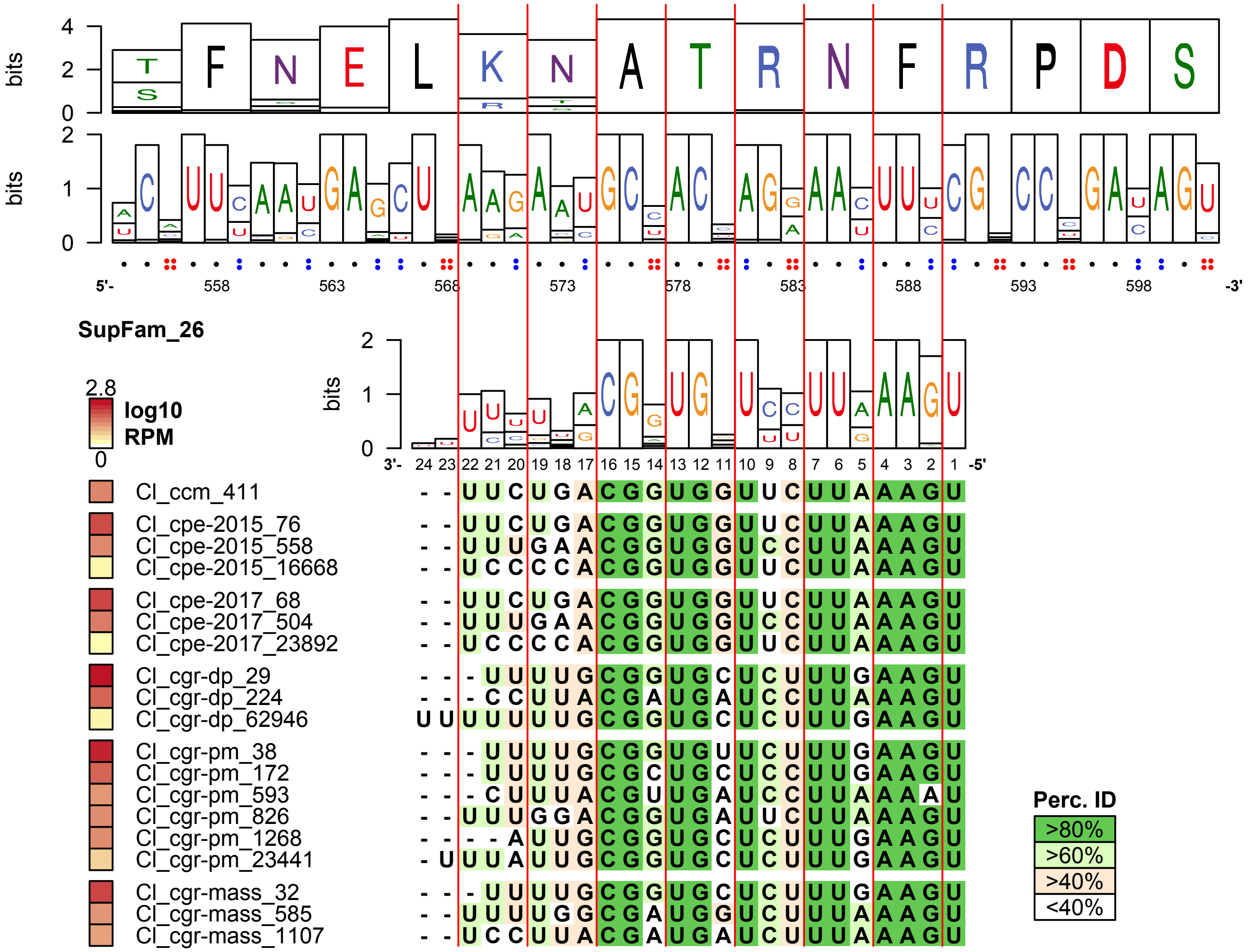

AT5G65430 - GRF8
36/36 homologs found in phytozome eudicots containing targetsite

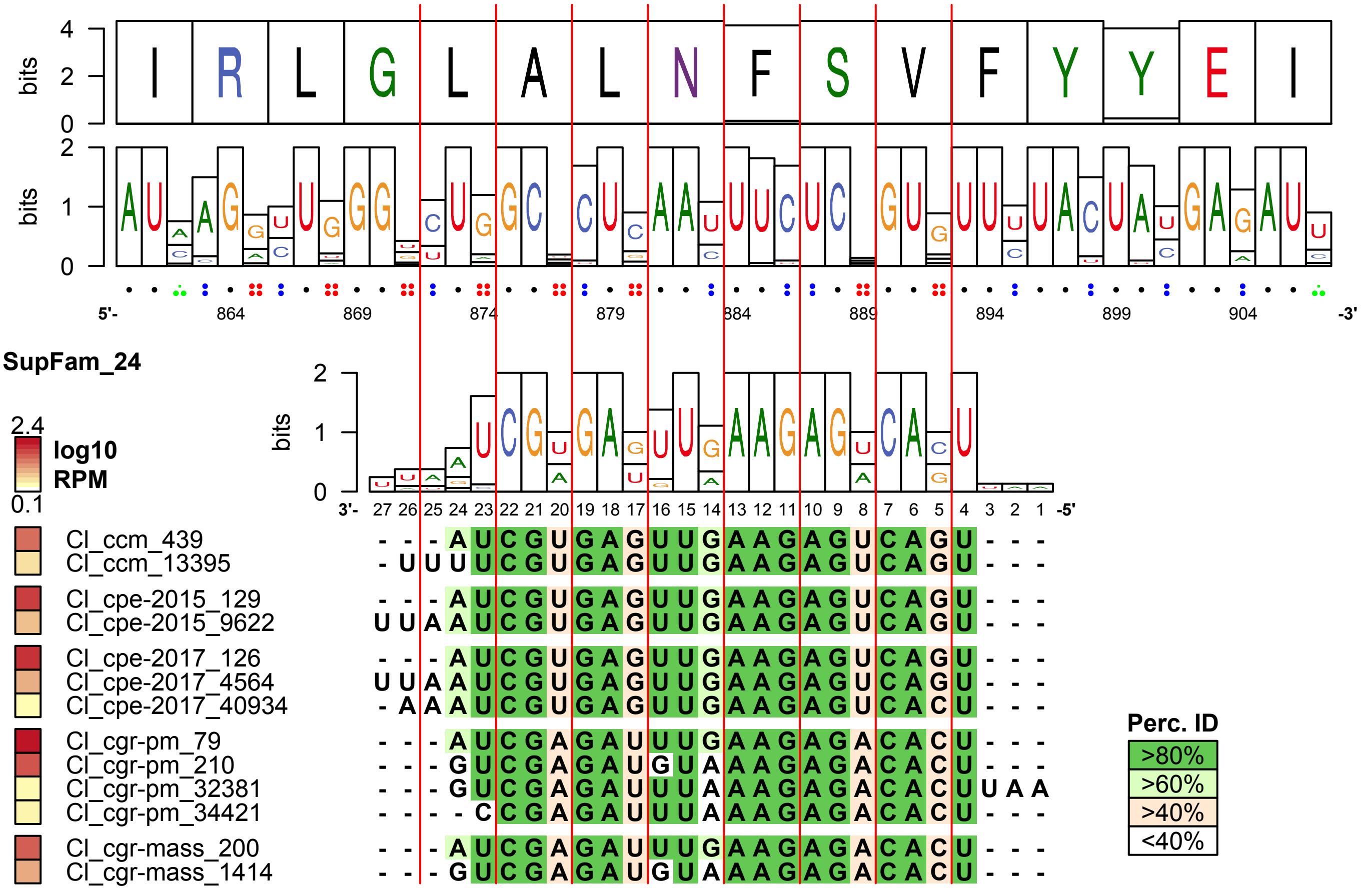

AT5G24010 - Protein_kinase_superfamily_protein
36/36 homologs found in phytozome eudicots containing targetsite

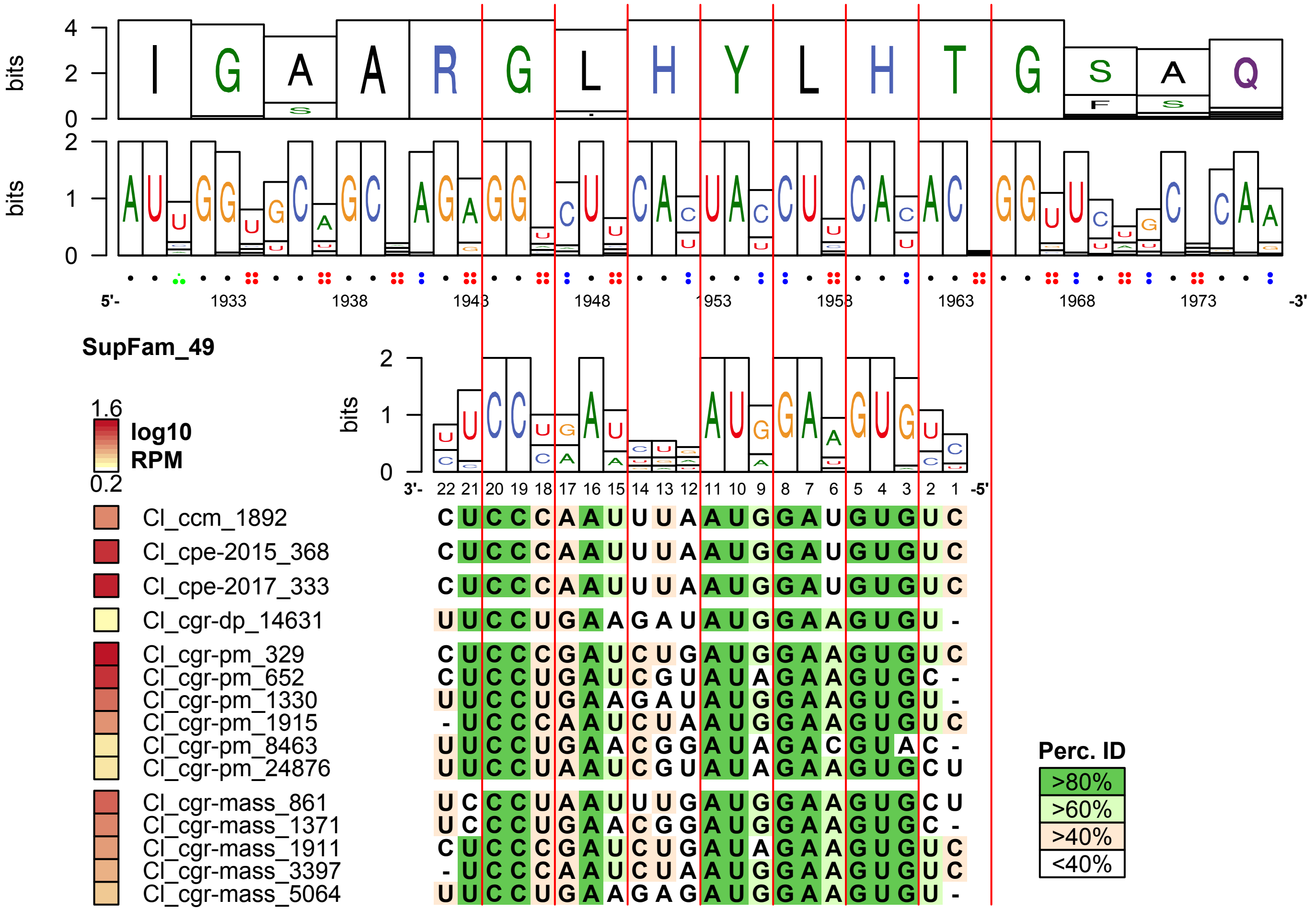

AT3G62980 - TIR1
35/36 homologs found in phytozome eudicots containing targetsite

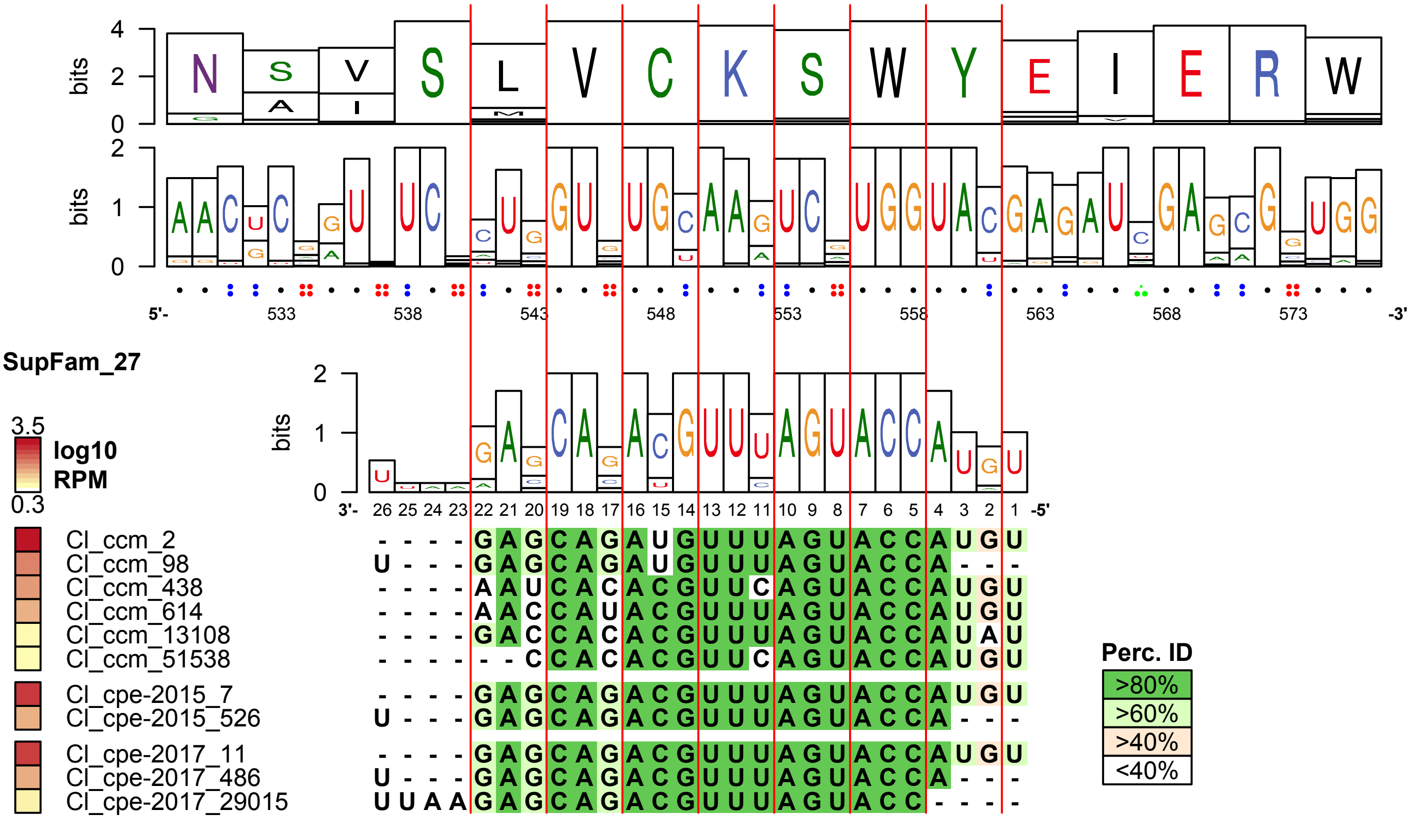

AT1G20696 - HMGB3
35/36 homologs found in phytozome eudicots containing targetsite

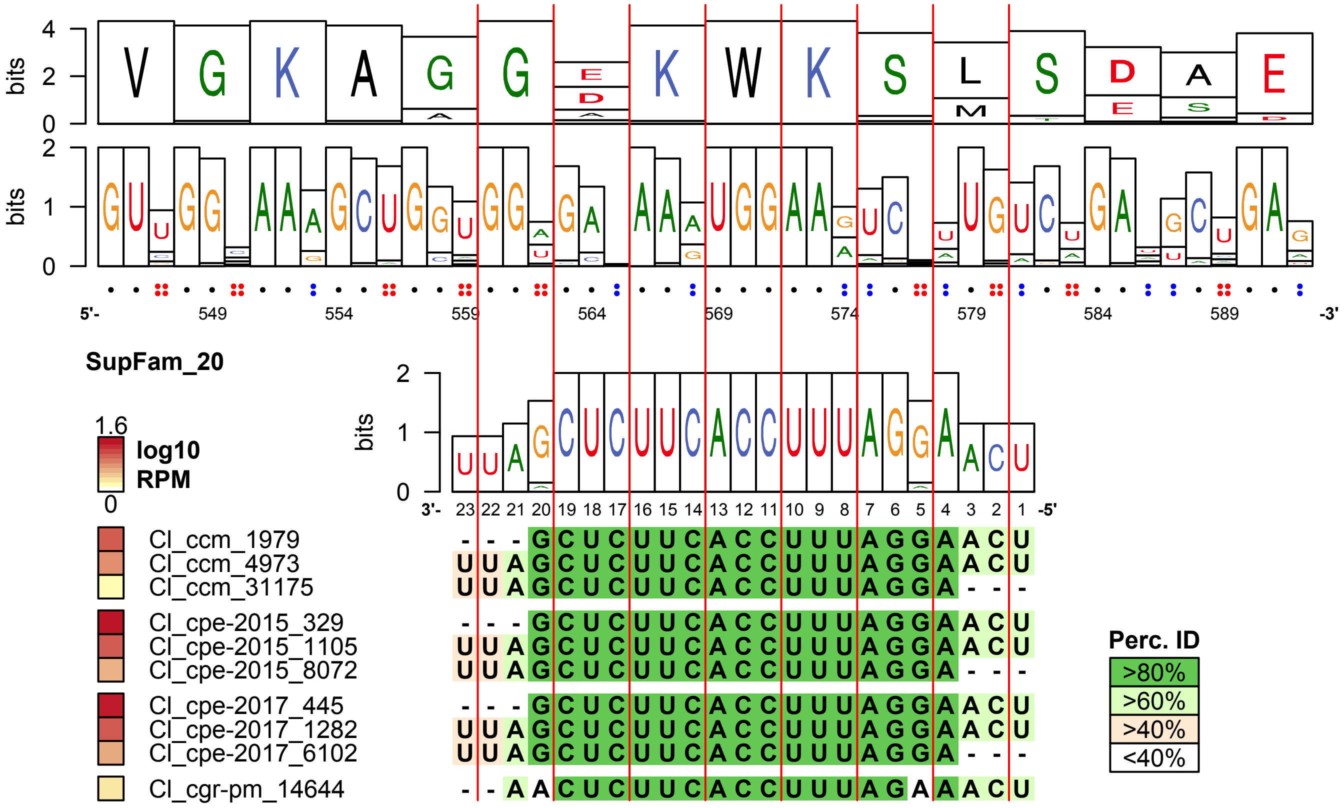

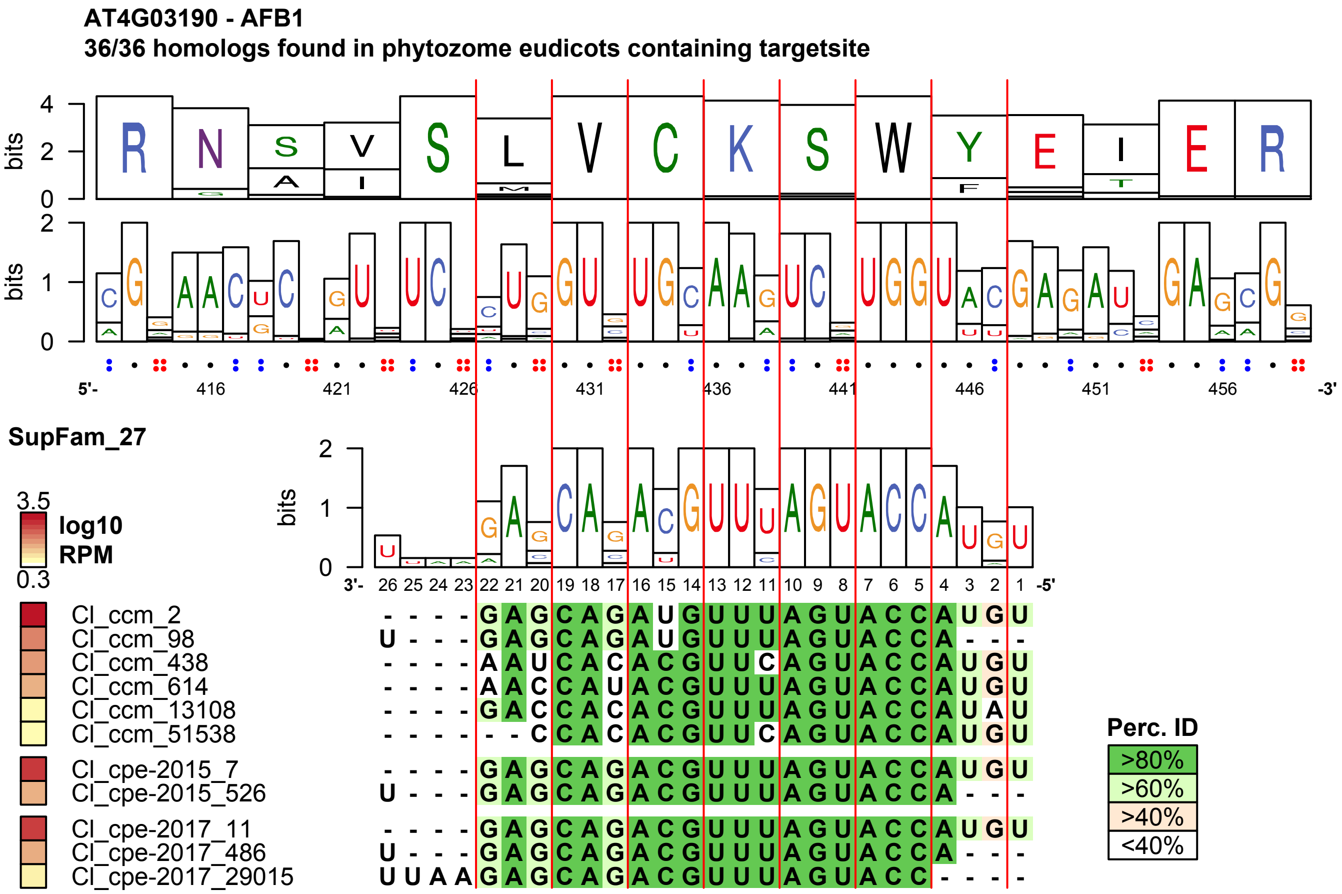
AT4G03190 - AFB1
36/36 homologs found in phytozome eudicots containing targetsite
bits
5'-
416
421
426
431
436
441
446
451
456
-3'
SupFam_27
log10
RPM
3.5
0.3
3'-
26 25 24 23 22 21 20 19 18 17 16 15 14 13 12 11 10 9 8 7 6 5 4 3 2 1
-5'
Cl_ccm_2 - - - - G A G C A G A U G U U U A G U A C C A U G U
Cl_ccm_98 U - - - G A G C A G A U G U U U A G U A C C A - - -
Cl_ccm_438 - - - - A A U C A C A C G U U C A G U A C C A U G U
Cl_ccm_614 - - - - A A C C A U A C G U U U A G U A C C A U G U
Cl_ccm_13108 - - - - G A C C A C A C G U U U A G U A C C A U A U
Cl_ccm_51538 - - - - - - C C A C A C G U U C A G U A C C A U G U
Cl_cpe-2015_7 - - - - G A G C A G A C G U U U A G U A C C A U G U
Cl_cpe-2015_526 U - - - G A G C A G A C G U U U A G U A C C A - - -
Cl_cpe-2017_11 - - - - G A G C A G A C G U U U A G U A C C A U G U
Cl_cpe-2017_486 U - - - G A G C A G A C G U U U A G U A C C A - - -
Cl_cpe-2017_29015 U U A A G A G C A G A C G U U U A G U A C C - - - -
Perc. ID
>80%
>60%
>40%
<40%

AT3G26810 - AFB2
36/36 homologs found in phytozome eudicots containing targetsite

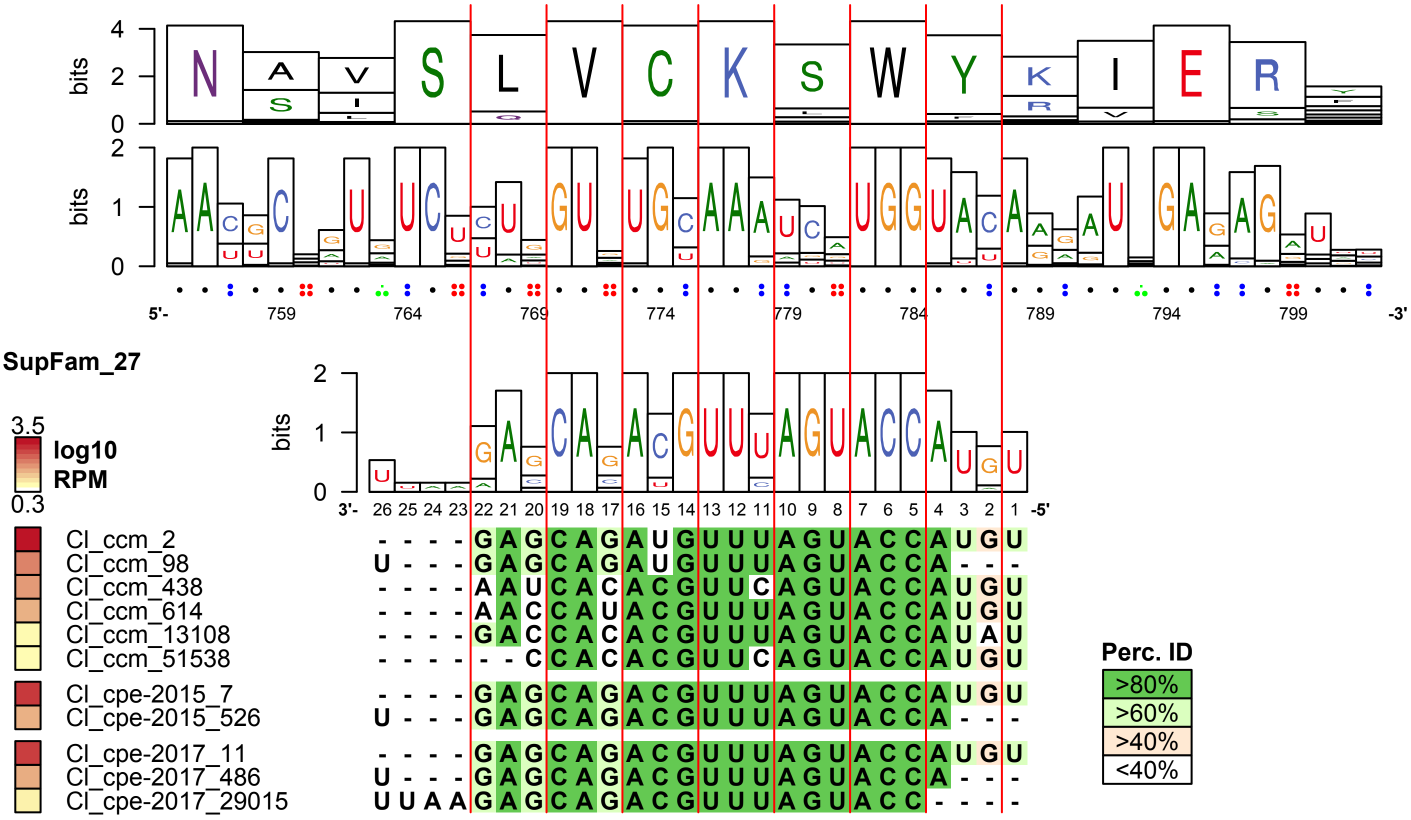

AT1G63910 - AtMYB103
36/36 homologs found in phytozome eudicots containing targetsite

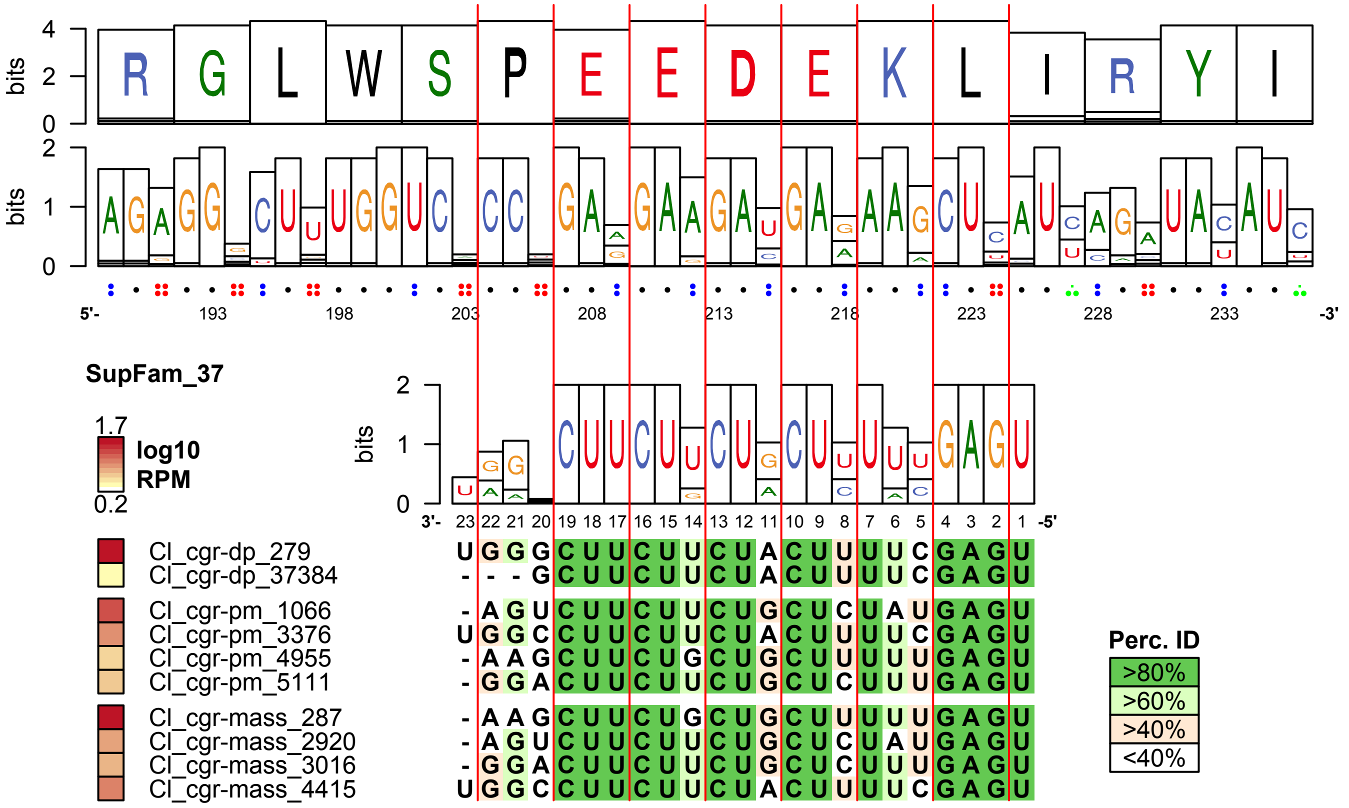

AT1G65800 - RK2

35/36 homologs found in phytozome eudicots containing targetsite

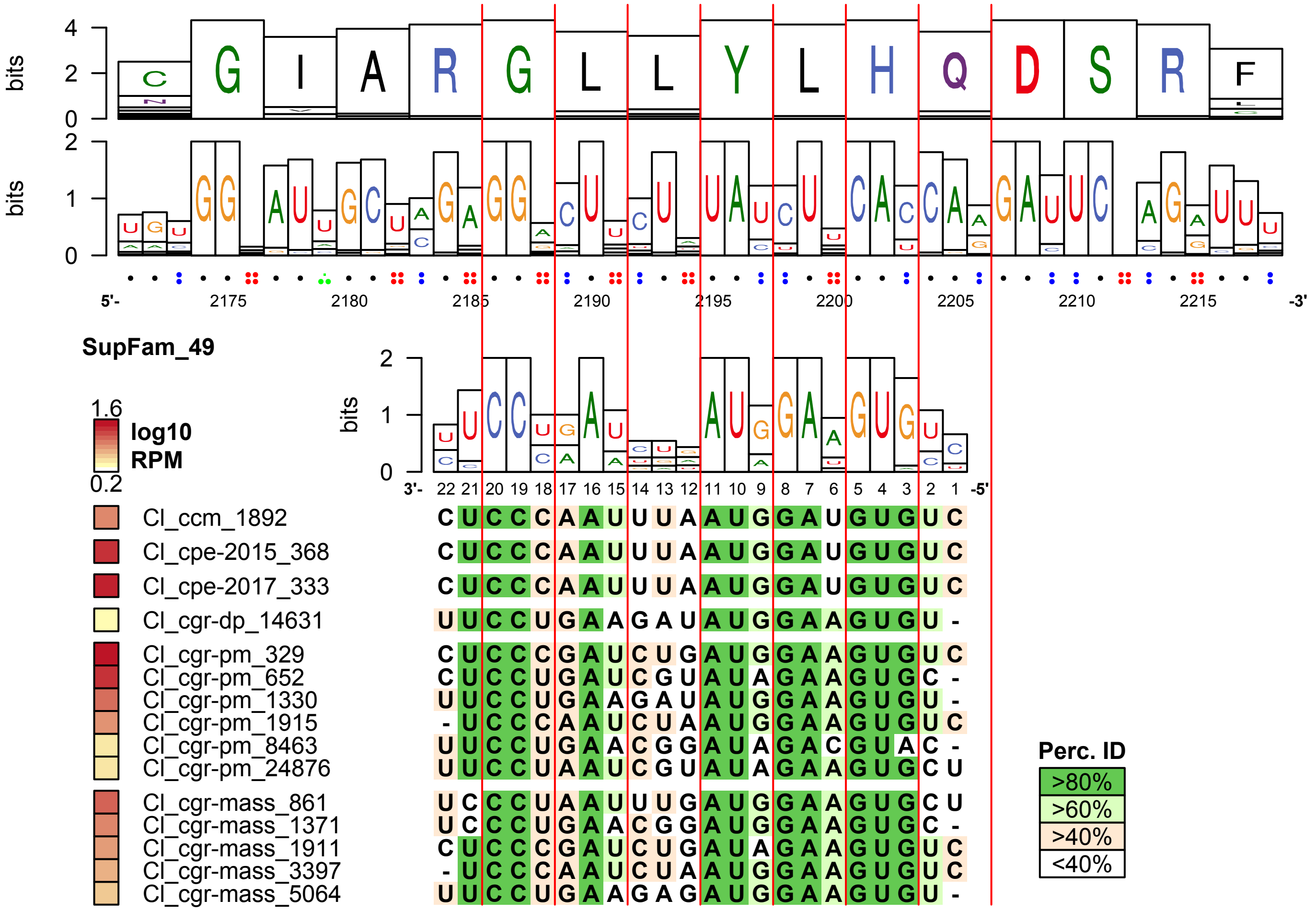

AT2G39660 - BIK1
36/36 homologs found in phytozome eudicots containing targetsite

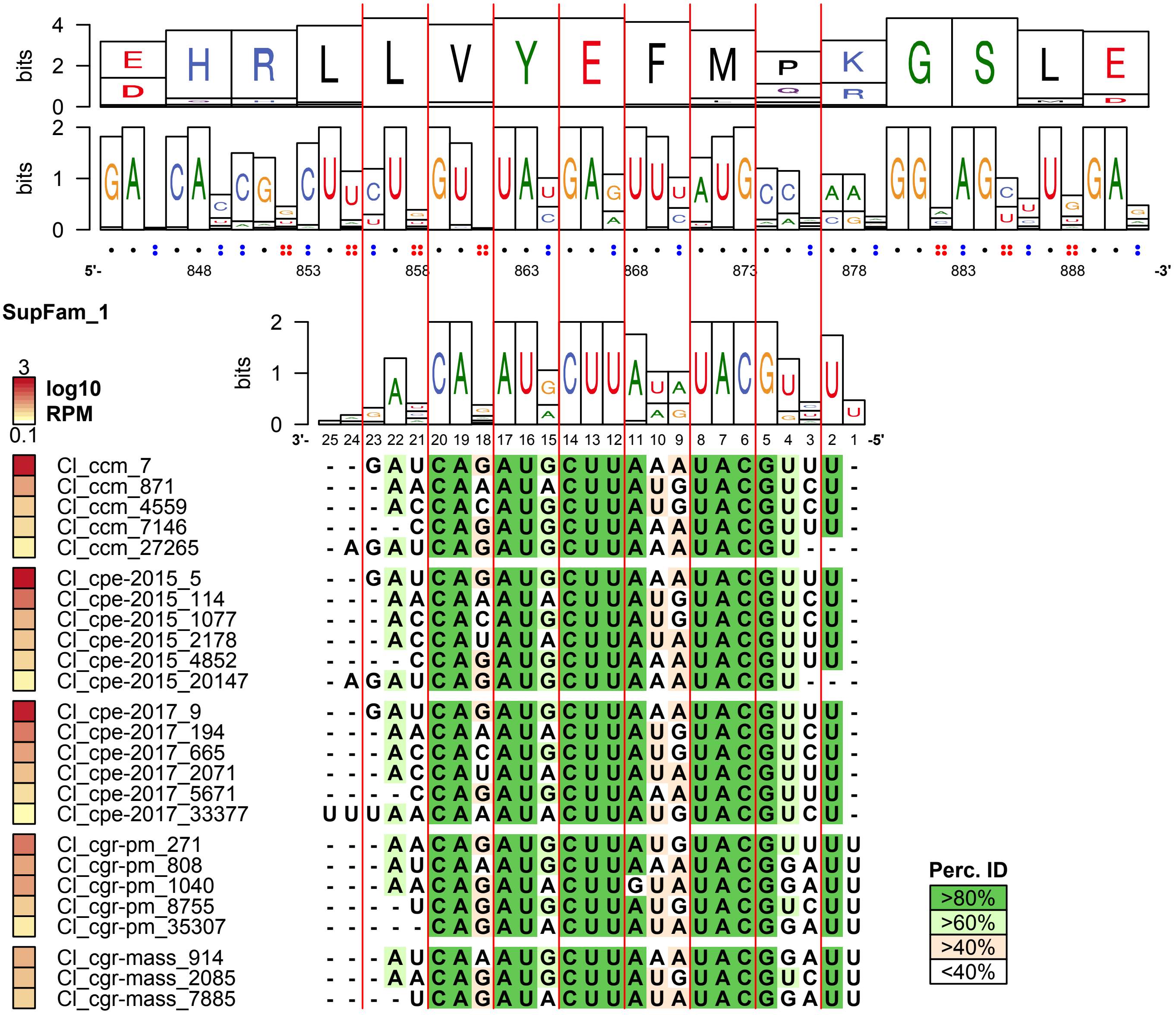

Supplement: Supplementary file 7. — Multiple sequence alignments of HI-sRNA superfamilies which have significant correlations between sRNA positional variation and target site variation. Alignment of eudicot homologs around target site also shown, with nucleotide and amino acid Shannon entropy shown as bits. Vertical red lines indicate the frame. Dots indicate the number of possible synonymous nucleotides at a position for the confirmed target’s sequence. Nucleotide positions are in reference to the position in the multiple sequence alignment. Format: PDF [file elife-49750-supp7.pdf]
