# Supplementary material for: Compensatory sequence variation between trans-species small RNAs and their target sites
Source: eLife. 2019 Dec 17;8:e49750. doi: 10.7554/eLife.49750 (PMC6917502; doi:10.7554/eLife.49750)

Ran BP2

AT3G15680
Niben101Scf00349g01014
ccm on *nbe*

sRNA	AS	tslice	phase	supfam	published_name
CI_ccm_253	1.5	261	9	11	ccm-MIR12486*
CI_ccm_23468	5.5	913	10		

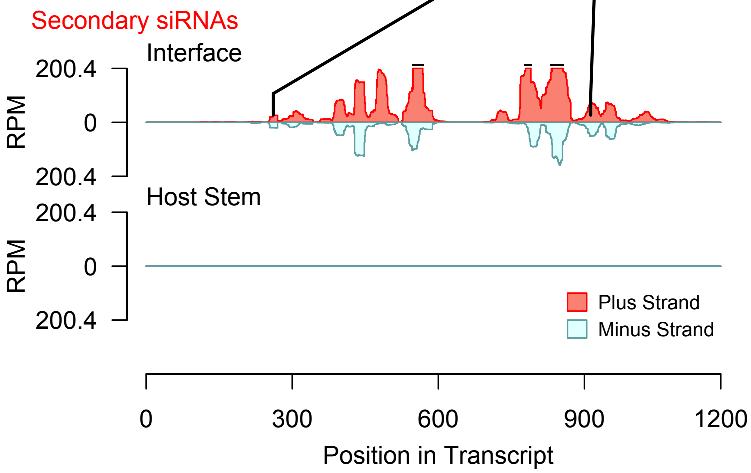

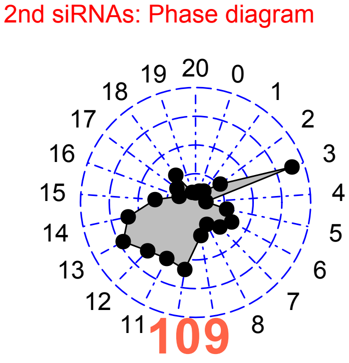

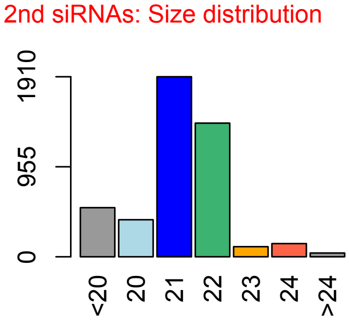

EIL3

AT1G73730

Niben101Scf00761g07020

ccm on *nbe*

sRNA	AS	tslice	phase	supfam	published_name
Cl_ccm_3720	3	508	4	305	

Secondary siRNAs

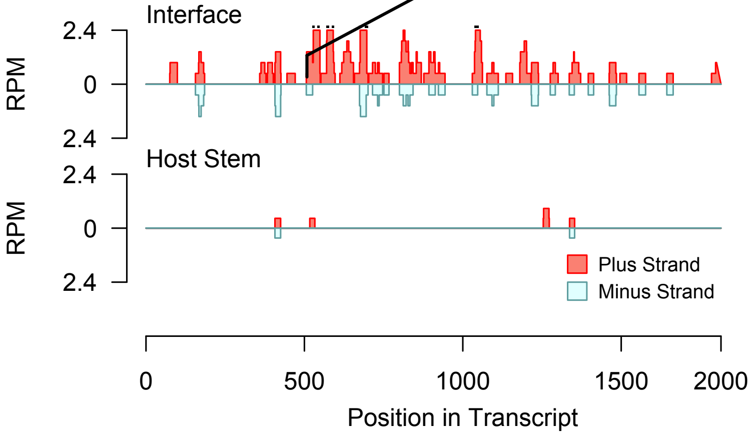

2nd siRNAs: Phase diagram

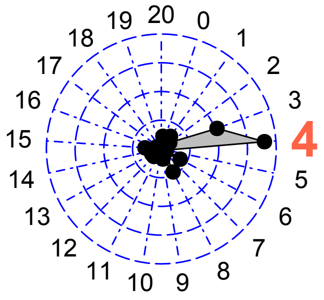

2nd siRNAs: Size distribution

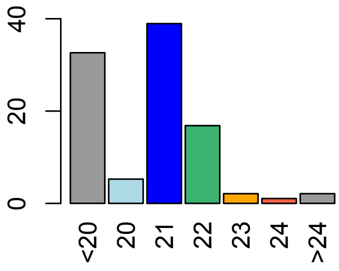

TIR1

AT3G62980
Niben101Scf00823g01010
ccm on *nbe*

sRNA	AS	tslice	phase	supfam	published_name
Cl_ccm_98	3.5	119	14	27	
Cl_ccm_2	3	122	17	27	ccm-MIR12497a*
Cl_ccm_614	5	122	17	27	ccm-MIR12497b

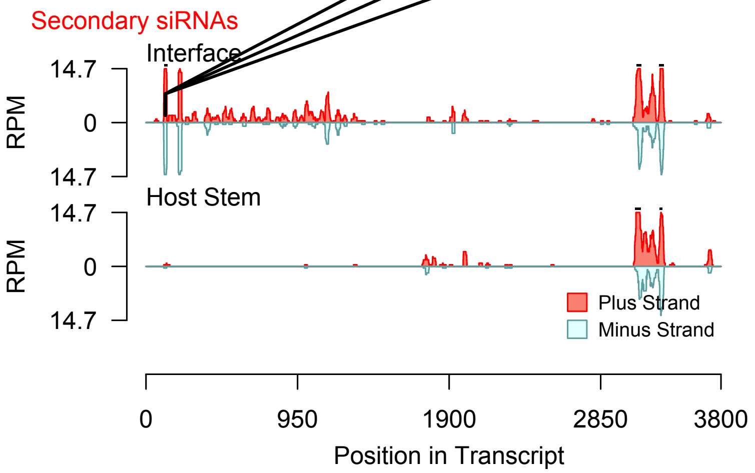

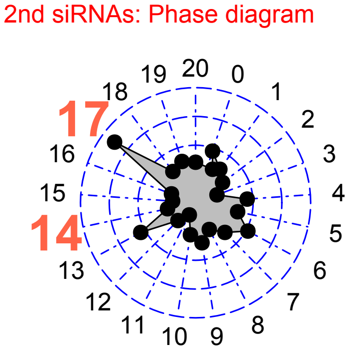

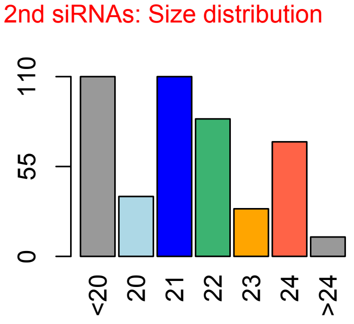

BIK1

AT2G39660
Niben101Scf01176g01025
ccm on *nbe*

sRNA	AS	tslice	phase	supfam	published_name
Cl_ccm_4717	3.5	204	15	310	ccm-MIR12468*
Cl_ccm_411	5.5	622	13	26	
Cl_ccm_9385	1	737	2	320	
Cl_ccm_7	2.5	877	16	1	ccm-MIR12463b
Cl_ccm_7146	2.5	877	16	1	
Cl_ccm_23740	4	1163	8	190	
Cl_ccm_117	5	1163	8	190	
Cl_ccm_934	5	1463	14	194	
Cl_ccm_3806	5	1928	17	306	ccm-MIR12476b*

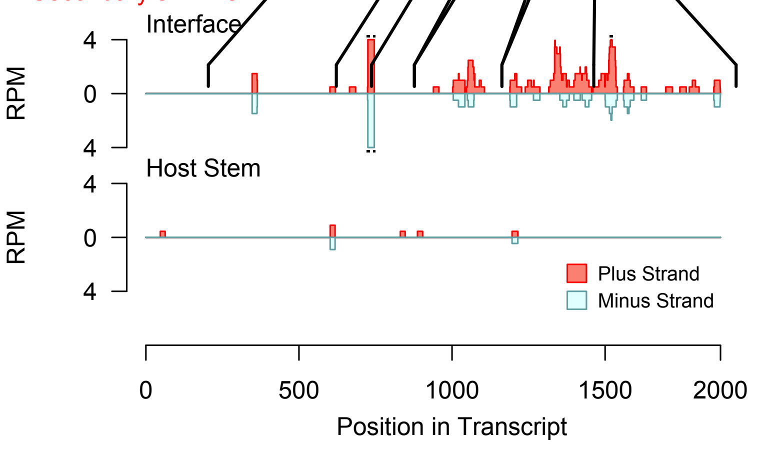

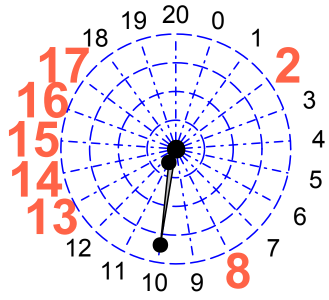

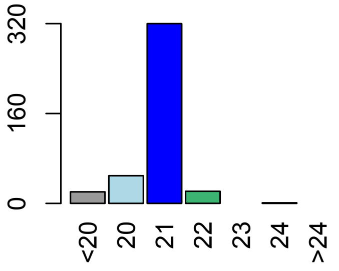

Ran BP2

AT3G15680
Niben101Scf01620g02009
ccm on *nbe*

sRNA	AS	tslice	phase	supfam	published_name
Cl_ccm_253	2.5	257	5	11	ccm-MIR12486*
Cl_ccm_23468	5.5	663	12		

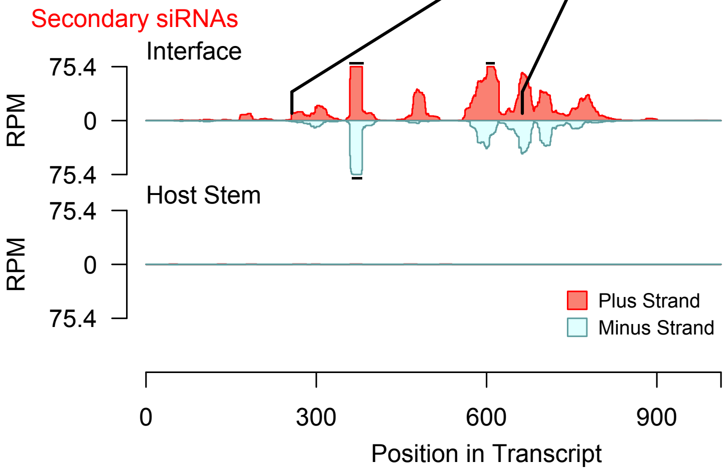

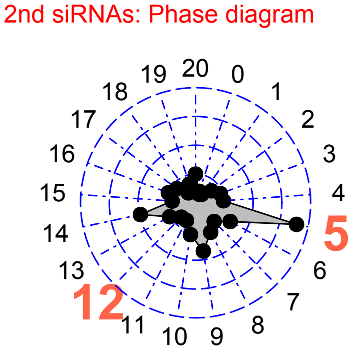

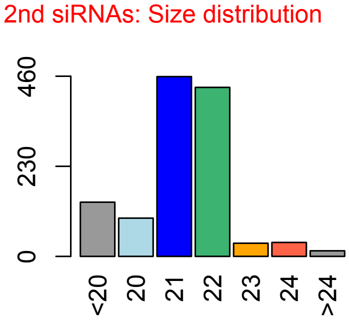

TIR1

AT3G62980

Niben101Scf01642g03012

ccm on *nbe*

sRNA	AS	tslice	phase	supfam	published_name
CI_ccm_98	4.5	233	2	27	
CI_ccm_2	4	236	5	27	ccm-MIR12497a*
CI_ccm_614	4.5	236	5	27	ccm-MIR12497b
CI_ccm_13108	6	236	5	27	

Secondary siRNAs

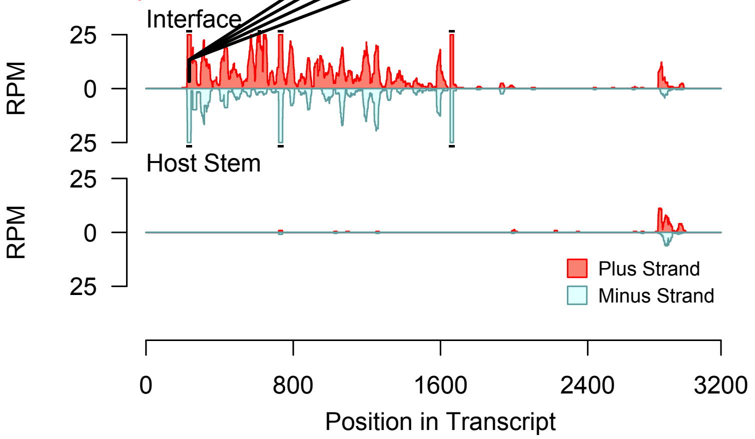

2nd siRNAs: Phase diagram

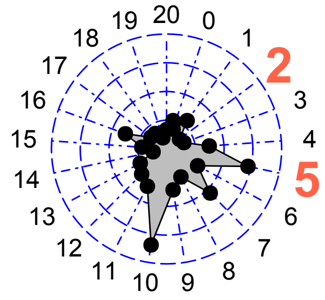

2nd siRNAs: Size distribution

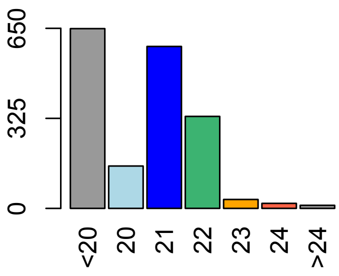

jmj-family

AT5G46910

Niben101Scf01822g04006

ccm on *nbe*

sRNA	AS	tslice	phase	supfam	published_name
Cl_ccm_17830	1.5	1053	3	329	

Secondary siRNAs

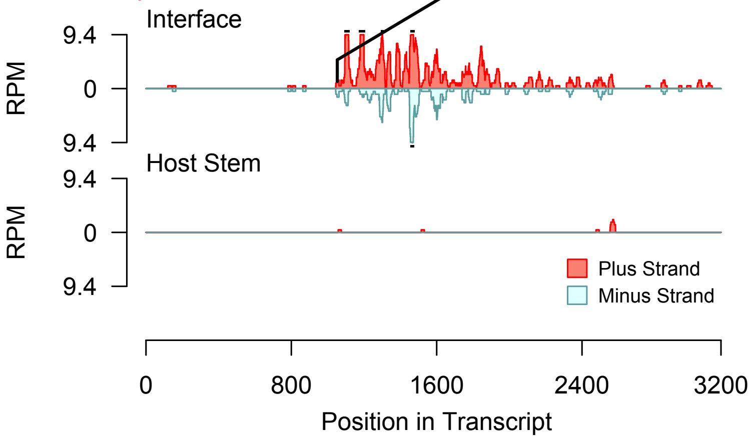

2nd siRNAs: Phase diagram

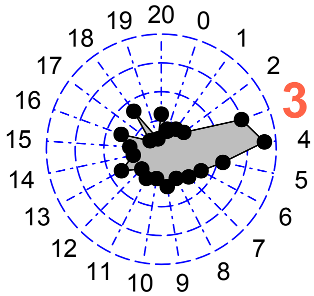

2nd siRNAs: Size distribution

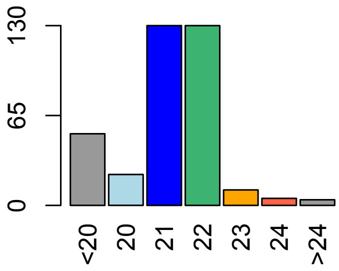

TIR1

AT3G62980

Niben101Scf04271g03003

ccm on *nbe*

sRNA	AS	tslice	phase	supfam	published_name
Cl_ccm_98	3.5	275	2	27	
Cl_ccm_2	3	278	5	27	ccm-MIR12497a*
Cl_ccm_614	5	278	5	27	ccm-MIR12497b

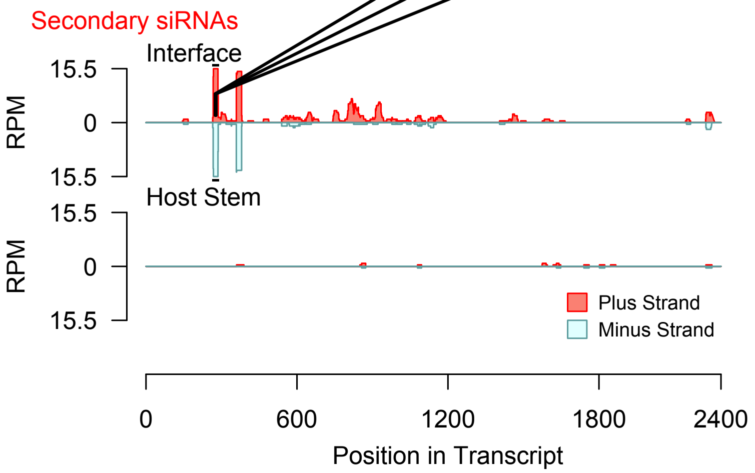

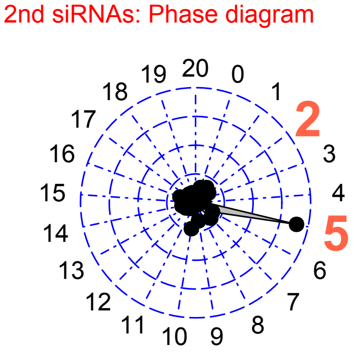

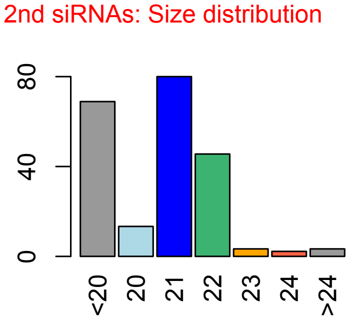

BIK1

AT2G39660

Niben101Scf04294g06004

ccm on *nbe*

sRNA	AS	tslice	phase	supfam	published_name
Cl_ccm_4717	3.5	134	8	310	ccm-MIR12468*
Cl_ccm_411	5.5	544	19	26	
Cl_ccm_9385	1	659	8	320	
Cl_ccm_7	2.5	799	1	1	ccm-MIR12463b
Cl_ccm_7146	2.5	799	1	1	
Cl_ccm_23740	4	1085	14	190	
Cl_ccm_117	5	1085	14	190	
Cl_ccm_934	5	1385	20	194	

Secondary siRNAs

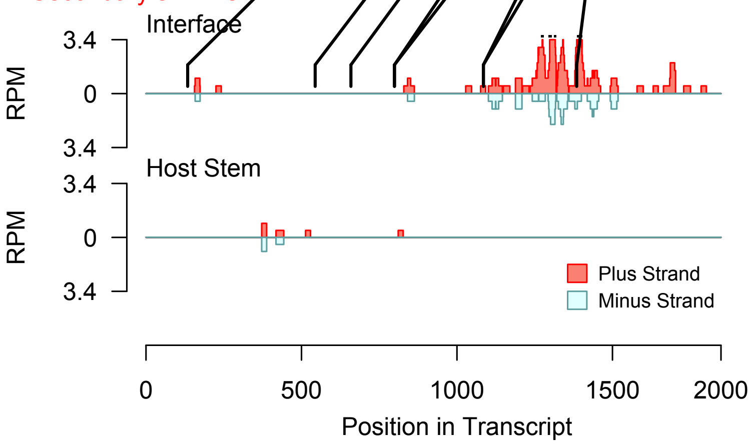

2nd siRNAs: Phase diagram

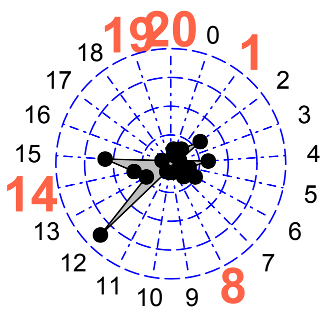

2nd siRNAs: Size distribution

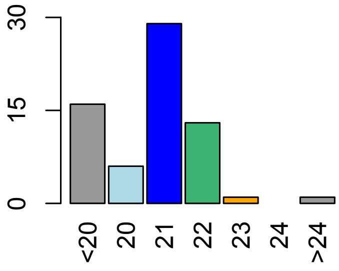

GalOx

AT2G02870

Niben101Scf09578g00002

ccm on *nbe*

sRNA	AS	tslice	phase	supfam	published_name
Cl_ccm_670	2	856	16	281	

Secondary siRNAs

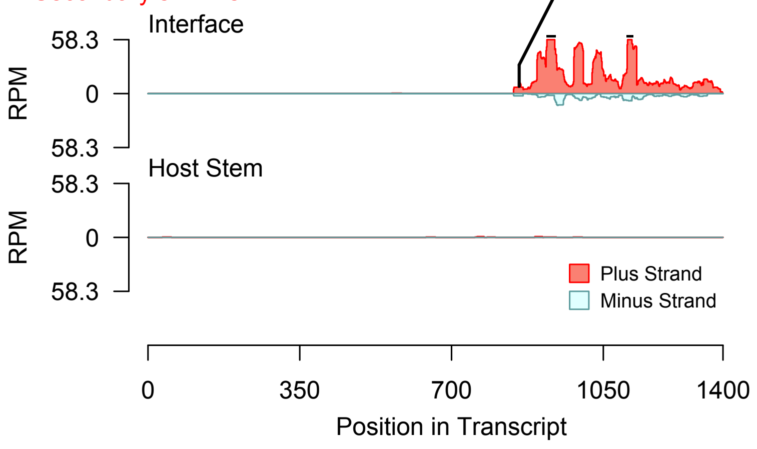

2nd siRNAs: Phase diagram

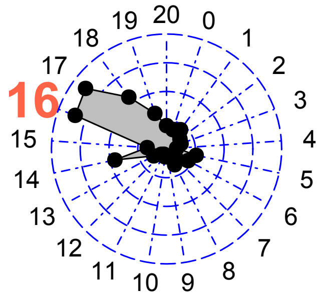

2nd siRNAs: Size distribution

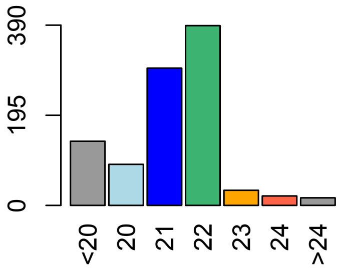

Protein_kinase

AT2G07180
Niben101Scf10157g01009
ccm on *nbe*

sRNA	AS	tslice	phase	supfam	Published_name
Cl_ccm_391	4.5	1098	6	123	
Cl_ccm_23740	5.5	1458	9	190	

Secondary siRNAs

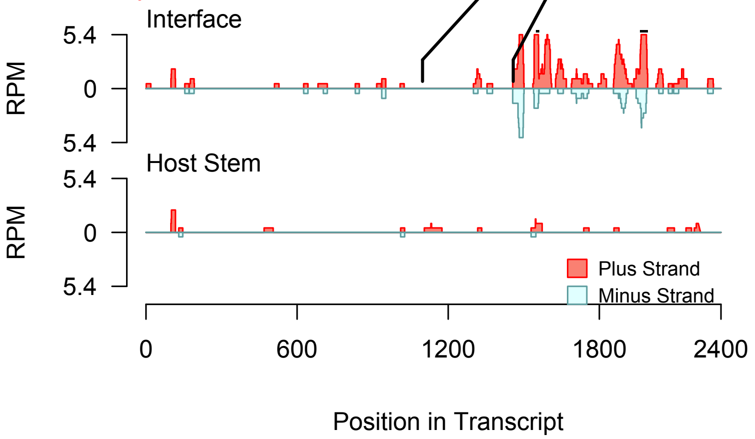

2nd siRNAs: Phase diagram

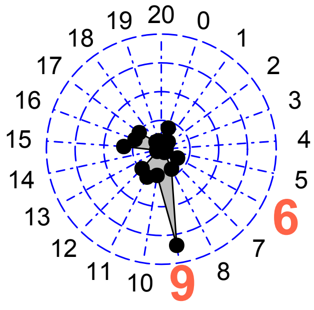

2nd siRNAs: Size distribution

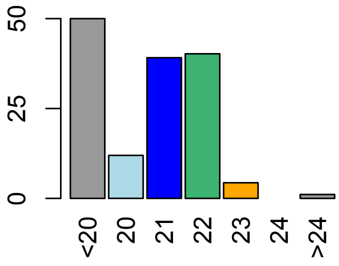

Protein_kinase

AT2G07180

Niben101Scf10157g01009

ccm on *nbe*

sRNA	AS	tslice	phase	supfam	published_name
Cl_ccm_391	4.5	1098	6	123	
Cl_ccm_23740	5.5	1458	9	190	

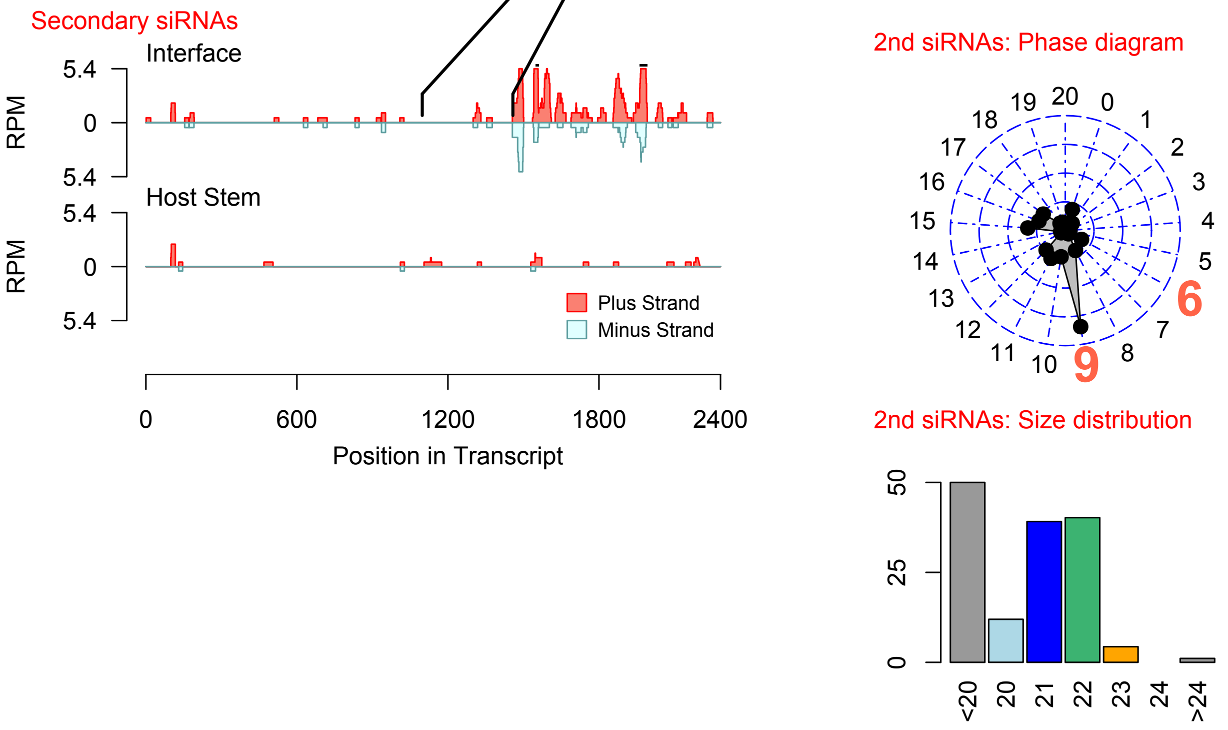

Supplement: Supplementary file 9. — Details of confirmed C. campestris HI-sRNA targets in N. benthamiana, including HI-sRNA-target complementarity, site, score, superfamily and the status of C. campestris superfamily members as a confirmed miRNA. sRNA distribution at target locus is shown for experimental interface and control, demonstrating secondary siRNA phasing and size distribution for up-regulated loci. Format: PDF [file elife-49750-supp9.pdf]
