# Supplementary material for: Compensatory sequence variation between trans-species small RNAs and their target sites
Source: eLife. 2019 Dec 17;8:e49750. doi: 10.7554/eLife.49750 (PMC6917502; doi:10.7554/eLife.49750)
Supplement: Supplementary file 11. — Multiple sequence alignments sRNA of superfamilies and conserved target motifs found in Arabidopsis transcriptome, with nucleotide and amino acid Shannon entropy shown as bits. Vertical red lines indicate the frame. Dots indicate the number of possible synonymous nucleotides at a position for the confirmed target’s sequence. Nucleotide positions are in reference to the position in the multiple sequence alignment. Color of gene names indicates if there is evidence for targeting in NanoPARE data (black - 0 replicates; orange - 1 or two replicates; red - three replicates, confirmed interaction). Format: PDF [file elife-49750-supp11.pdf]

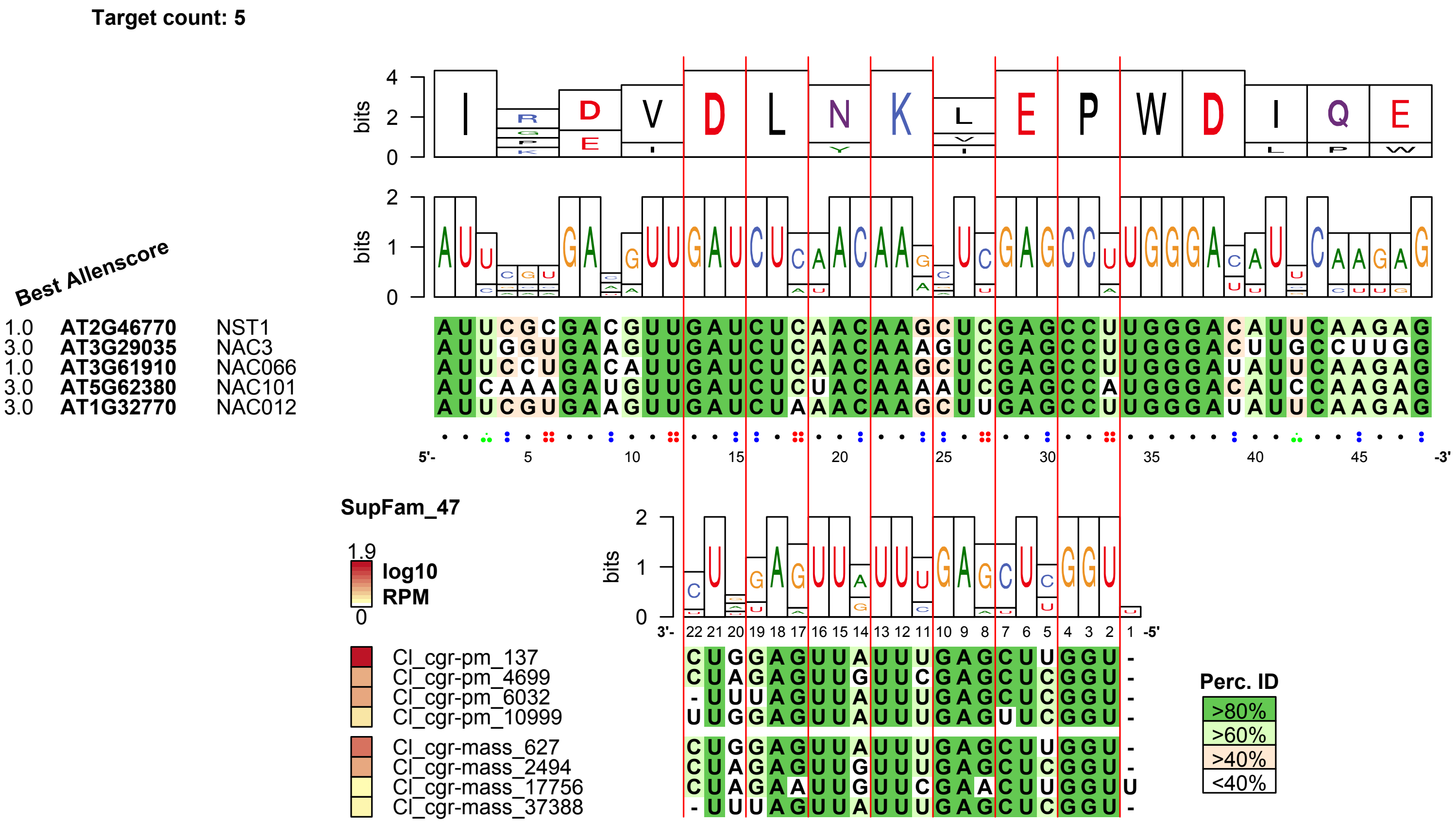
Target count: 5
Best Allenscore
1.0 AT2G46770 NST1
3.0 AT3G29035 NAC3
1.0 AT3G61910 NAC066
3.0 AT5G62380 NAC101
3.0 AT1G32770 NAC012
AUUCGCGACGUUGAUCUCAACAAGCUCGAGCCUUGGGACAUUCAAGAG
AUUGGUGAAGUUGAUCUCAACAAAGUCGAGCCUUGGGACUUGCCUUGG
AUUCCUGACAUUGAUCUCAACAAGCUCGAGCCUUGGGAUAUUCAAGAG
AUCAAAGAUGUUGAUCUCUACAAAAUCGAGCCAUGGGACAUCCAAGAG
AUUCGUGAAGUUGAUCUAAACAAGCUUGAGCCUUGGGAUAUUCAAGAG
I D V D L N K L E P W D I Q E
bits
5'- 5 10 15 20 25 30 35 40 45 -3'
SupFam_47
1.9 log10 RPM 0
Cl_cgr-pm_137
Cl_cgr-pm_4699
Cl_cgr-pm_6032
Cl_cgr-pm_10999
Cl_cgr-mass_627
Cl_cgr-mass_2494
Cl_cgr-mass_17756
Cl_cgr-mass_37388
3'- 22 21 20 19 18 17 16 15 14 13 12 11 10 9 8 7 6 5 4 3 2 1 -5'
CUGGAGUUAUUUGAGCUUGGU -
CUAGAGUUGUUCGAGCUCGGU -
-UUUAGUUAUUUGAGCUCGGU -
UUGGAGUUAUUUGAGUUCGGU -
CUGGAGUUAUUUGAGCUUGGU -
CUAGAGUUGUUUGAGCUCGGU -
CUAGAAUUGUUCGAACUUGGUU
-UUUAGUUAUUUGAGCUCGGU -
Perc. ID
>80%
>60%
>40%
<40%

Target count: 5

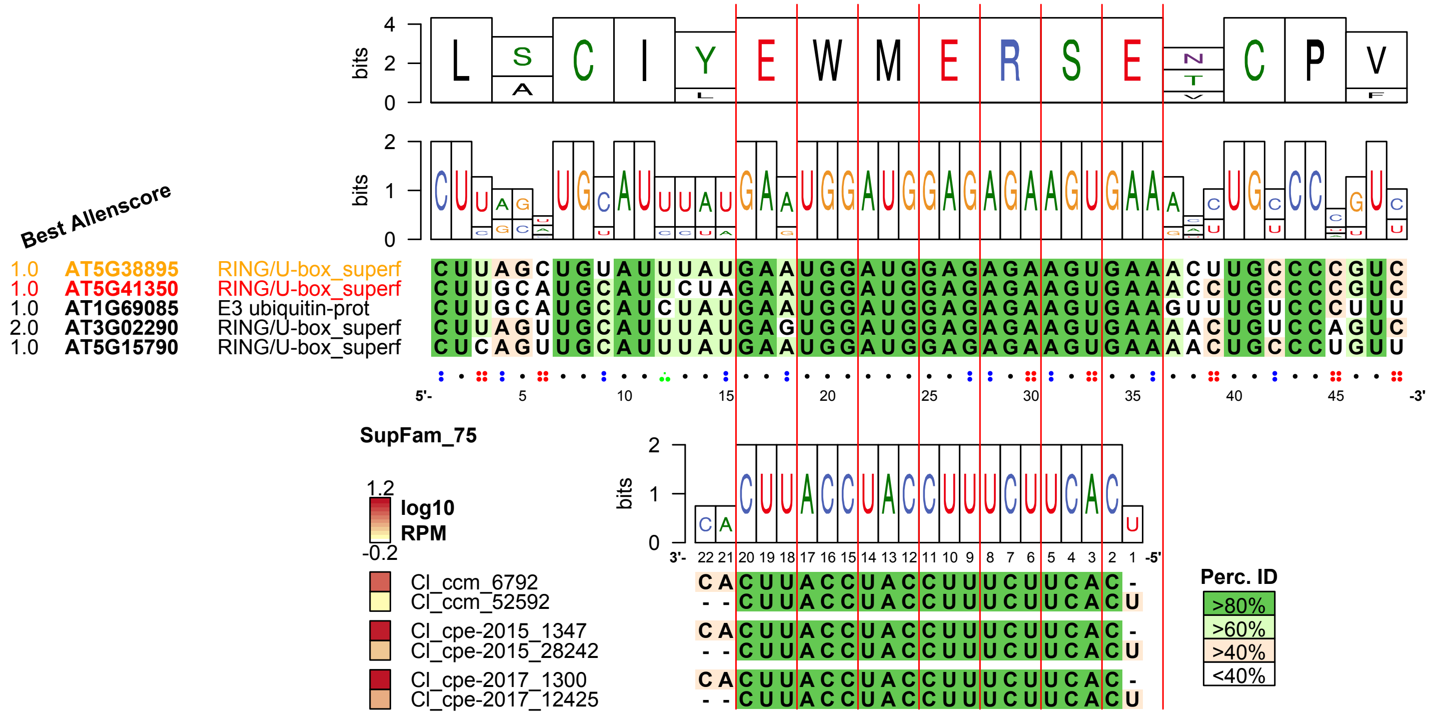

Target count: 18

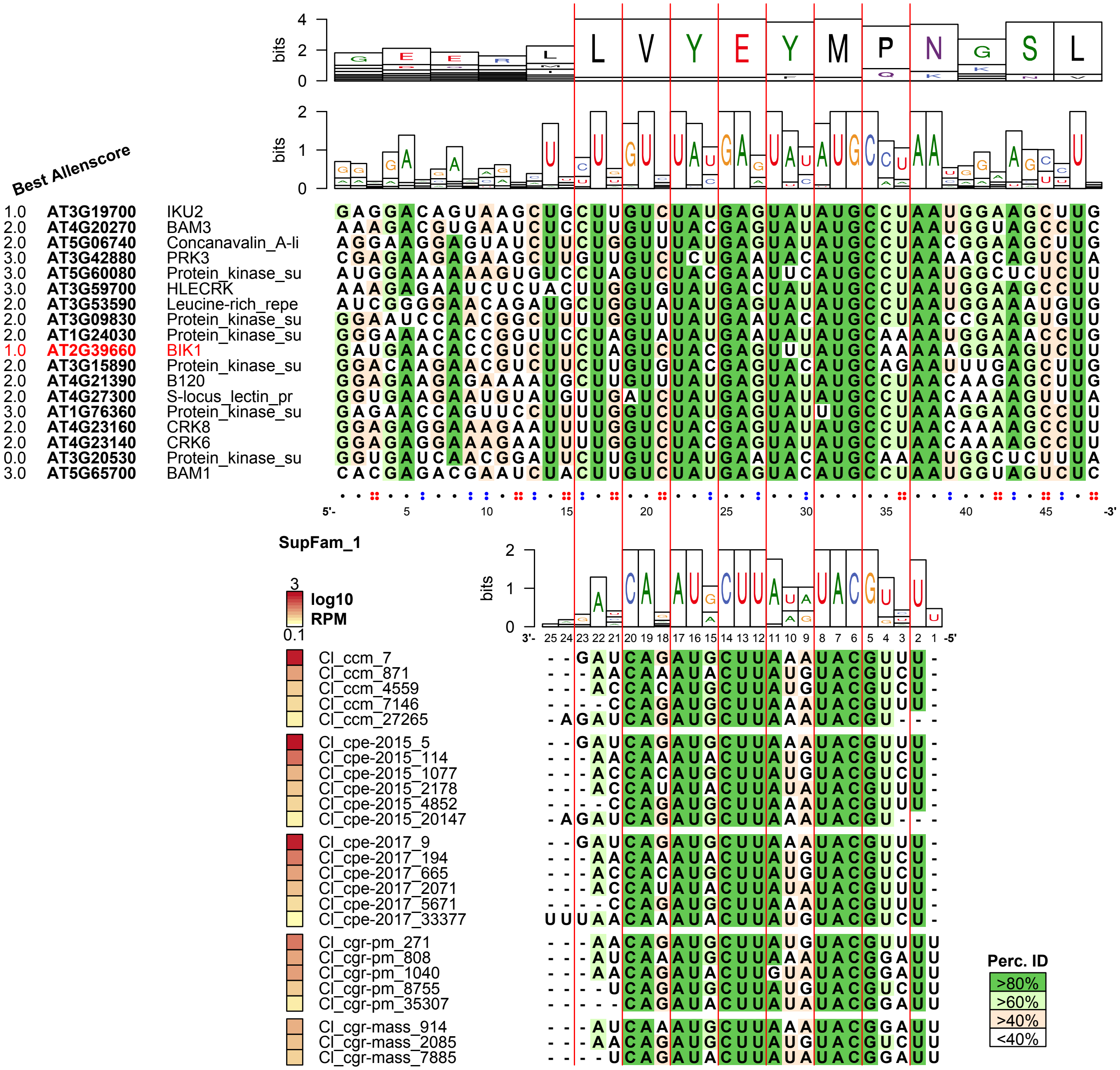

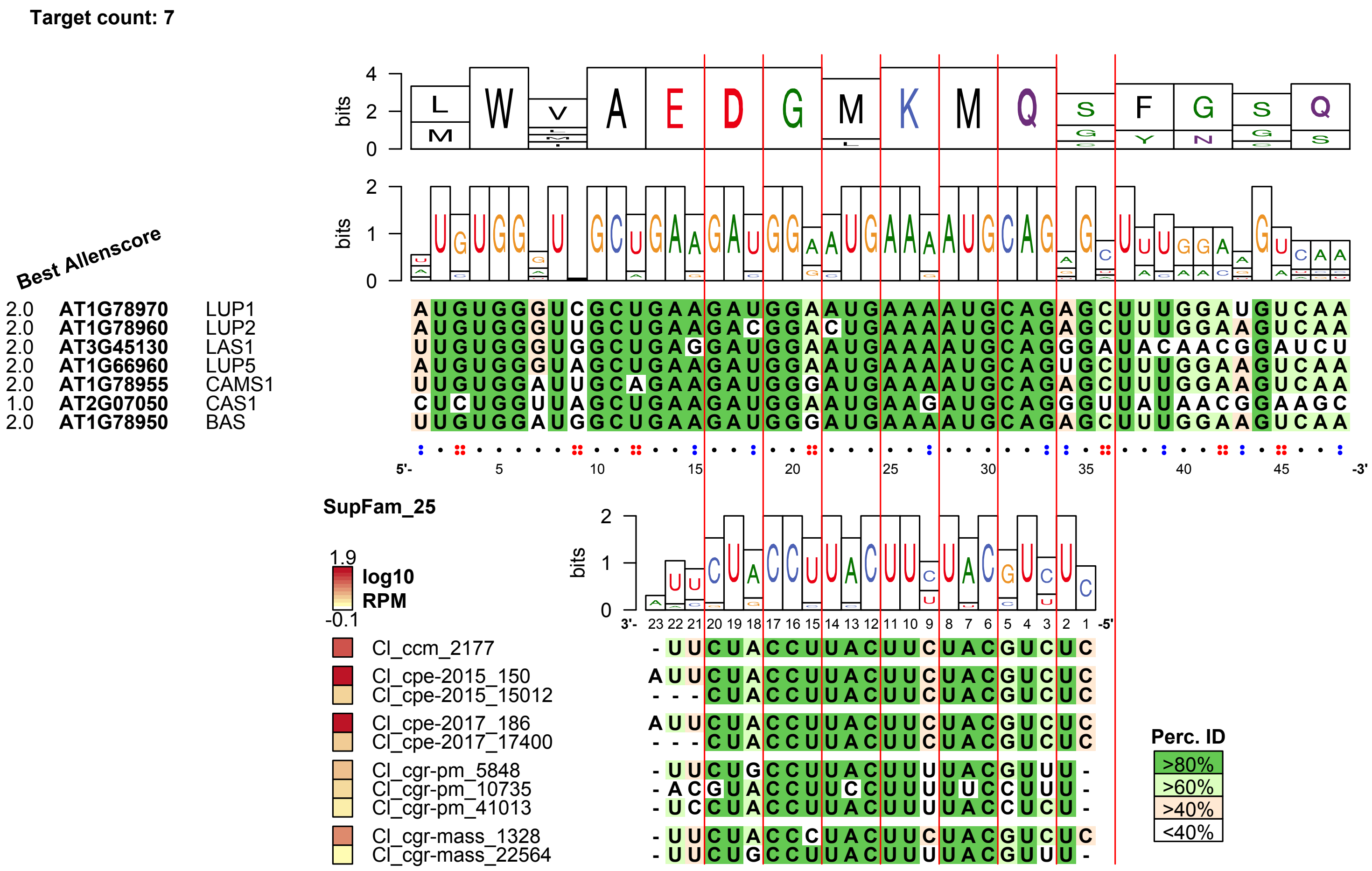

Target count: 7
Best Allenscore
2.0 AT1G78970 LUP1
2.0 AT1G78960 LUP2
2.0 AT3G45130 LAS1
2.0 AT1G66960 LUP5
2.0 AT1G78955 CAMS1
1.0 AT2G07050 CAS1
2.0 AT1G78950 BAS
SupFam_25
log10 RPM
Cl_ccm_2177
Cl_cpe-2015_150
Cl_cpe-2015_15012
Cl_cpe-2017_186
Cl_cpe-2017_17400
Cl_cgr-pm_5848
Cl_cgr-pm_10735
Cl_cgr-pm_41013
Cl_cgr-mass_1328
Cl_cgr-mass_22564
Perc. ID
>80%
>60%
>40%
<40%

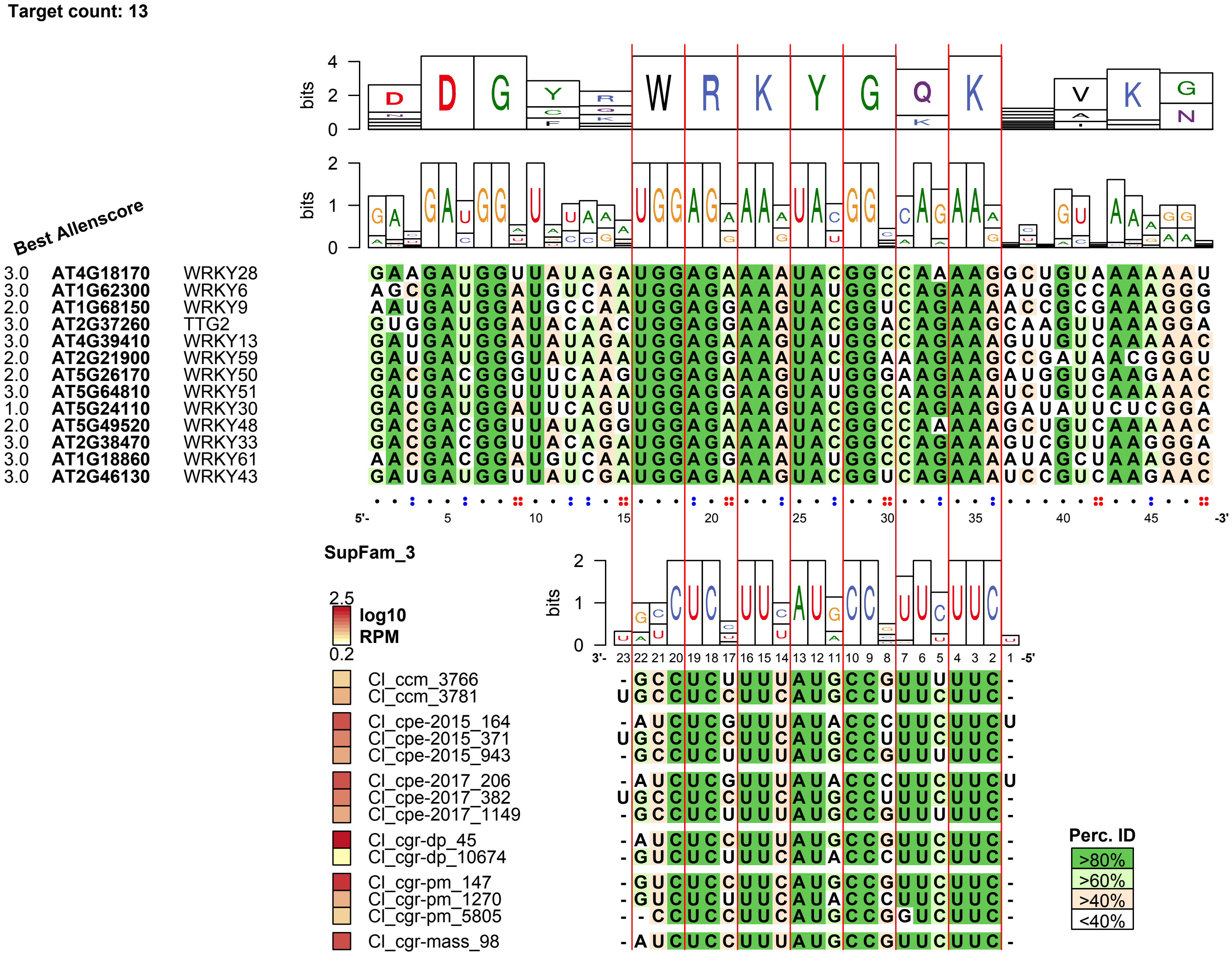
Target count: 13
Best Allenscore
3.0 AT4G18170 WRKY28
3.0 AT1G62300 WRKY6
2.0 AT1G68150 WRKY9
3.0 AT2G37260 TTG2
3.0 AT4G39410 WRKY13
2.0 AT2G21900 WRKY59
2.0 AT5G26170 WRKY50
3.0 AT5G64810 WRKY51
1.0 AT5G24110 WRKY30
2.0 AT5G49520 WRKY48
3.0 AT2G38470 WRKY33
3.0 AT1G18860 WRKY61
3.0 AT2G46130 WRKY43
bits
SupFam_3
log10 RPM
Cl_ccm_3766
Cl_ccm_3781
Cl_cpe-2015_164
Cl_cpe-2015_371
Cl_cpe-2015_943
Cl_cpe-2017_206
Cl_cpe-2017_382
Cl_cpe-2017_1149
Cl_cgr-dp_45
Cl_cgr-dp_10674
Cl_cgr-pm_147
Cl_cgr-pm_1270
Cl_cgr-pm_5805
Cl_cgr-mass_98
Perc. ID
>80%
>60%
>40%
<40%

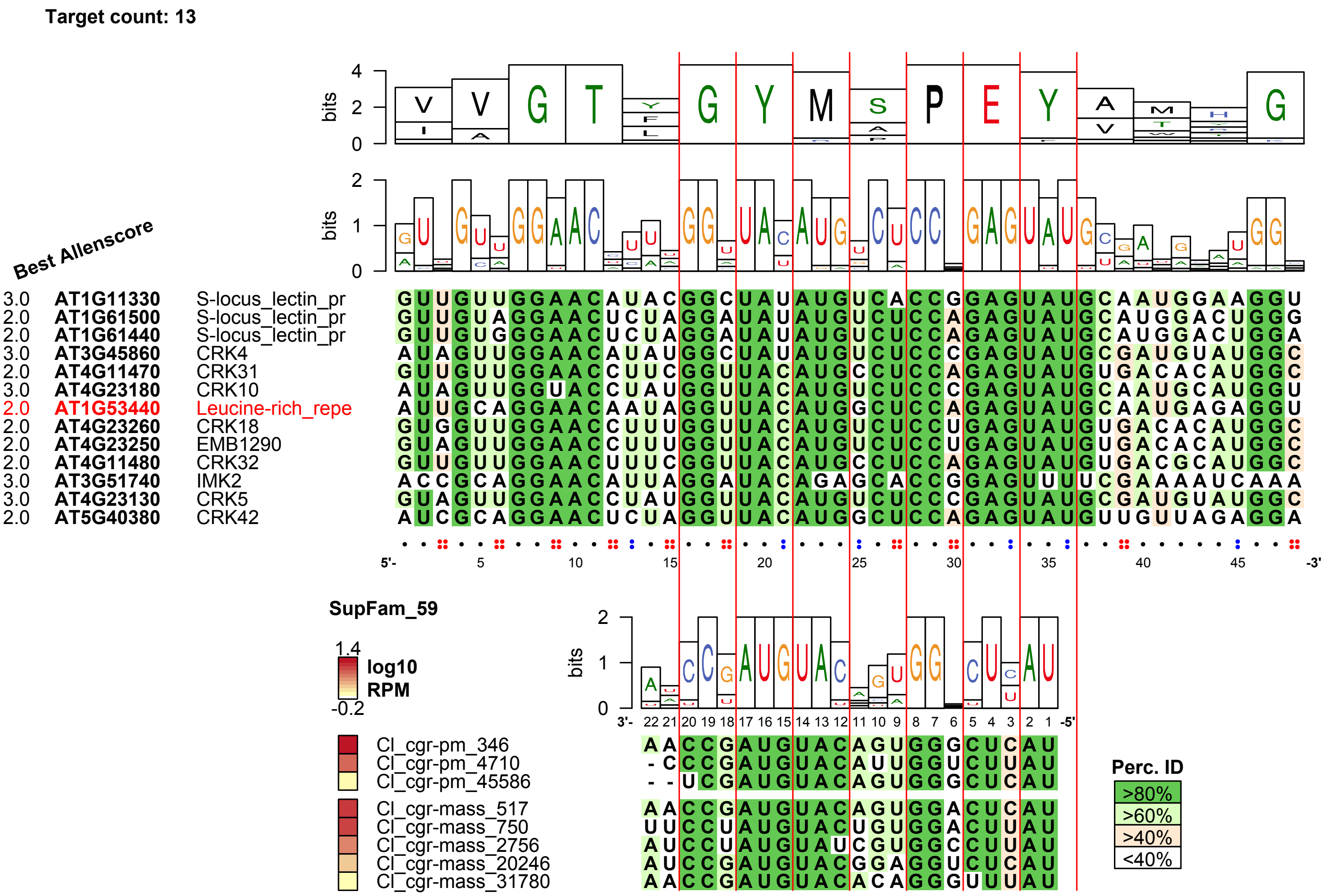

Target count: 13
Best Allenscore
3.0 AT1G11330 S-locus_lectin_pr
2.0 AT1G61500 S-locus_lectin_pr
2.0 AT1G61440 S-locus_lectin_pr
3.0 AT3G45860 CRK4
2.0 AT4G11470 CRK31
3.0 AT4G23180 CRK10
2.0 AT1G53440 Leucine-rich_repe
2.0 AT4G23260 CRK18
2.0 AT4G23250 EMB1290
2.0 AT4G11480 CRK32
3.0 AT3G51740 IMK2
3.0 AT4G23130 CRK5
2.0 AT5G40380 CRK42
bits
SupFam_59
log10 RPM
1.4
-0.2
Cl_cgr-pm_346
Cl_cgr-pm_4710
Cl_cgr-pm_45586
Cl_cgr-mass_517
Cl_cgr-mass_750
Cl_cgr-mass_2756
Cl_cgr-mass_20246
Cl_cgr-mass_31780
Perc. ID
>80%
>60%
>40%
<40%

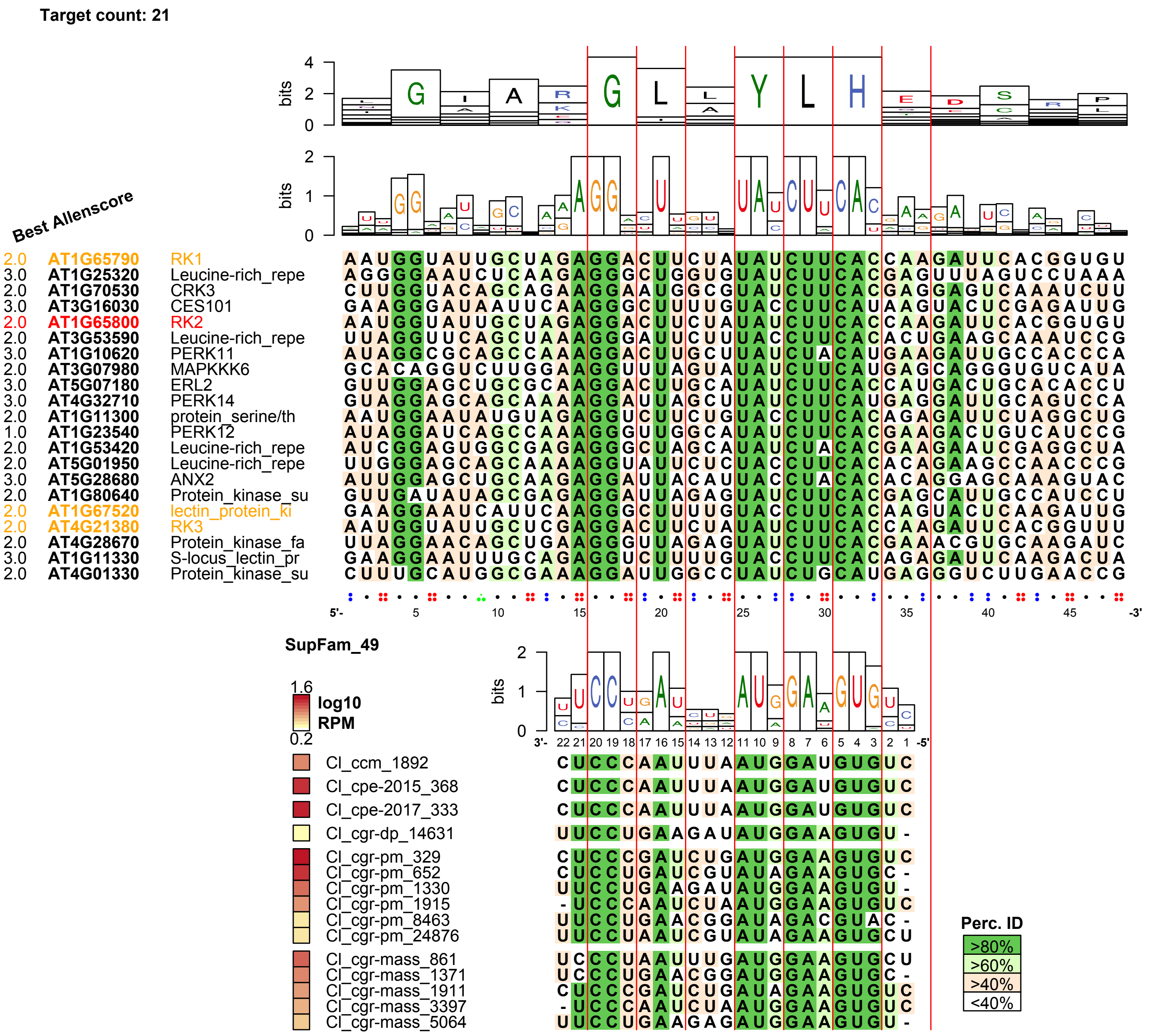
Target count: 21
Best Allenscore
bits
2.0 AT1G65790 RK1
3.0 AT1G25320 Leucine-rich_repe
2.0 AT1G70530 CRK3
3.0 AT3G16030 CES101
2.0 AT1G65800 RK2
2.0 AT3G53590 Leucine-rich_repe
3.0 AT1G10620 PERK11
2.0 AT3G07980 MAPKKK6
3.0 AT5G07180 ERL2
3.0 AT4G32710 PERK14
2.0 AT1G11300 protein_serine/th
1.0 AT1G23540 PERK12
2.0 AT1G53420 Leucine-rich_repe
2.0 AT5G01950 Leucine-rich_repe
3.0 AT5G28680 ANX2
2.0 AT1G80640 Protein_kinase_su
2.0 AT1G67520 lectin_protein_ki
2.0 AT4G21380 RK3
2.0 AT4G28670 Protein_kinase_fa
3.0 AT1G11330 S-locus_lectin_pr
2.0 AT4G01330 Protein_kinase_su
5'- 5 10 15 20 25 30 35 40 45 -3'
SupFam_49
1.6 log10 RPM 0.2
Cl_ccm_1892
Cl_cpe-2015_368
Cl_cpe-2017_333
Cl_cgr-dp_14631
Cl_cgr-pm_329
Cl_cgr-pm_652
Cl_cgr-pm_1330
Cl_cgr-pm_1915
Cl_cgr-pm_8463
Cl_cgr-pm_24876
Cl_cgr-mass_861
Cl_cgr-mass_1371
Cl_cgr-mass_1911
Cl_cgr-mass_3397
Cl_cgr-mass_5064
3'- 22 21 20 19 18 17 16 15 14 13 12 11 10 9 8 7 6 5 4 3 2 1 -5'
Perc. ID
>80%
>60%
>40%
<40%

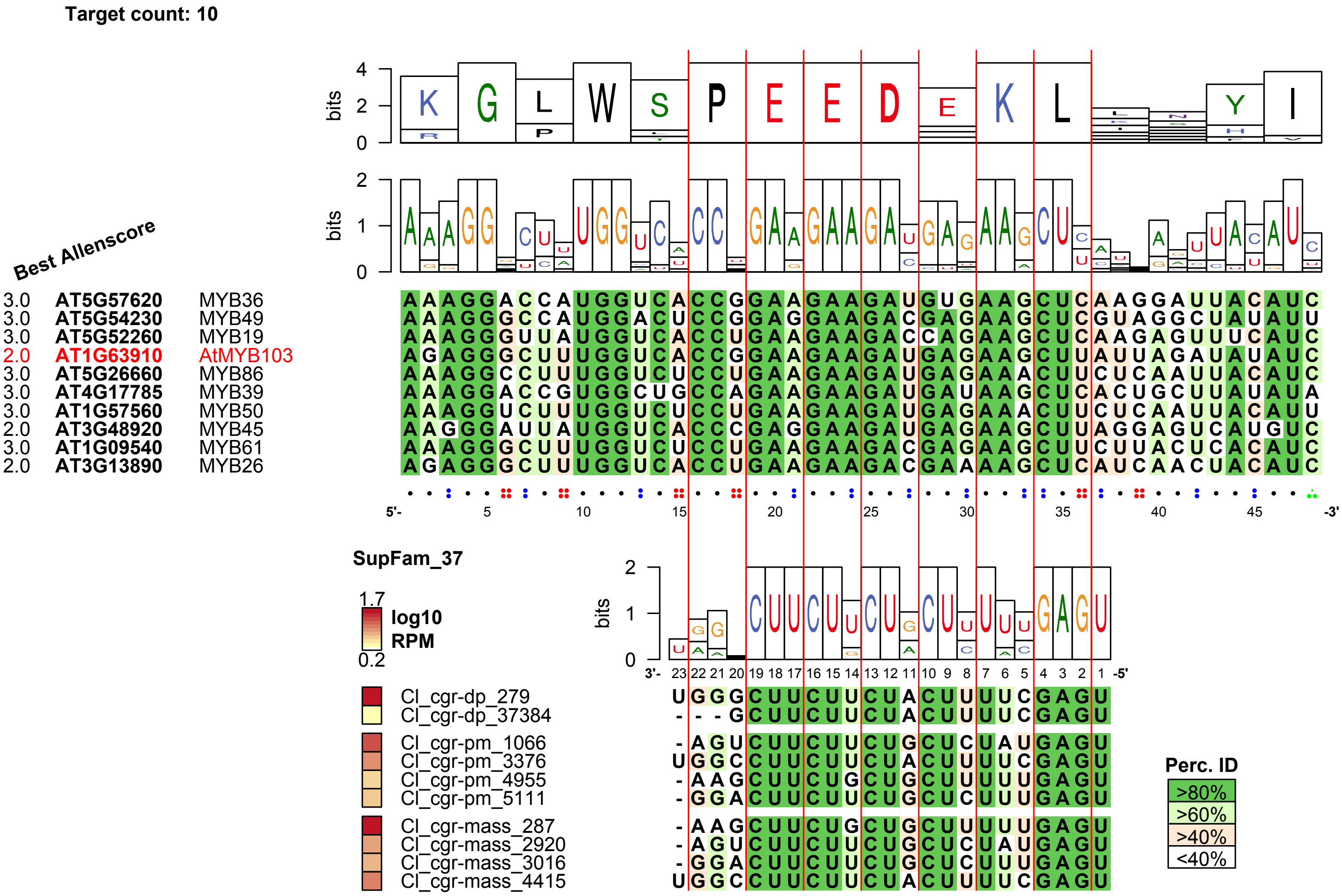
Target count: 10
Best Allenscore
3.0 AT5G57620 MYB36
3.0 AT5G54230 MYB49
3.0 AT5G52260 MYB19
2.0 AT1G63910 AtMYB103
3.0 AT5G26660 MYB86
3.0 AT4G17785 MYB39
3.0 AT1G57560 MYB50
2.0 AT3G48920 MYB45
3.0 AT1G09540 MYB61
2.0 AT3G13890 MYB26
bits
K G L W S P E E D E K L Y I
5'- 5 10 15 20 25 30 35 40 45 -3'
SupFam_37
1.7 log10 RPM 0.2
Cl_cgr-dp_279
Cl_cgr-dp_37384
Cl_cgr-pm_1066
Cl_cgr-pm_3376
Cl_cgr-pm_4955
Cl_cgr-pm_5111
Cl_cgr-mass_287
Cl_cgr-mass_2920
Cl_cgr-mass_3016
Cl_cgr-mass_4415
3'- 23 22 21 20 19 18 17 16 15 14 13 12 11 10 9 8 7 6 5 4 3 2 1 -5'
Perc. ID
>80%
>60%
>40%
<40%

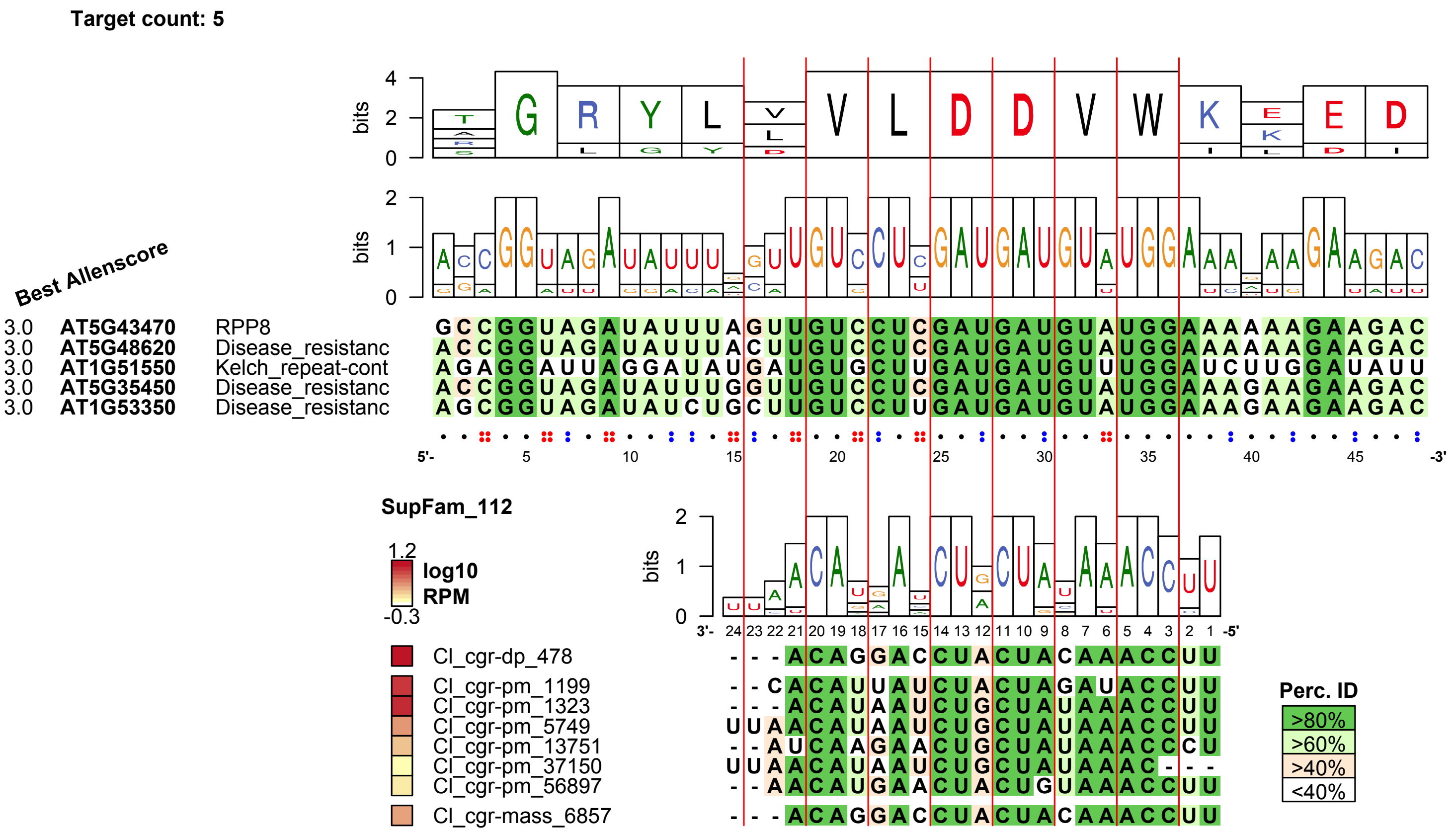
Target count: 5
Best Allenscore
3.0 AT5G43470 RPP8
3.0 AT5G48620 Disease_resistanc
3.0 AT1G51550 Kelch_repeat-cont
3.0 AT5G35450 Disease_resistanc
3.0 AT1G53350 Disease_resistanc
bits
SupFam_112
log10
RPM
1.2
-0.3
Cl_cgr-dp_478
Cl_cgr-pm_1199
Cl_cgr-pm_1323
Cl_cgr-pm_5749
Cl_cgr-pm_13751
Cl_cgr-pm_37150
Cl_cgr-pm_56897
Cl_cgr-mass_6857
Perc. ID
>80%
>60%
>40%
<40%

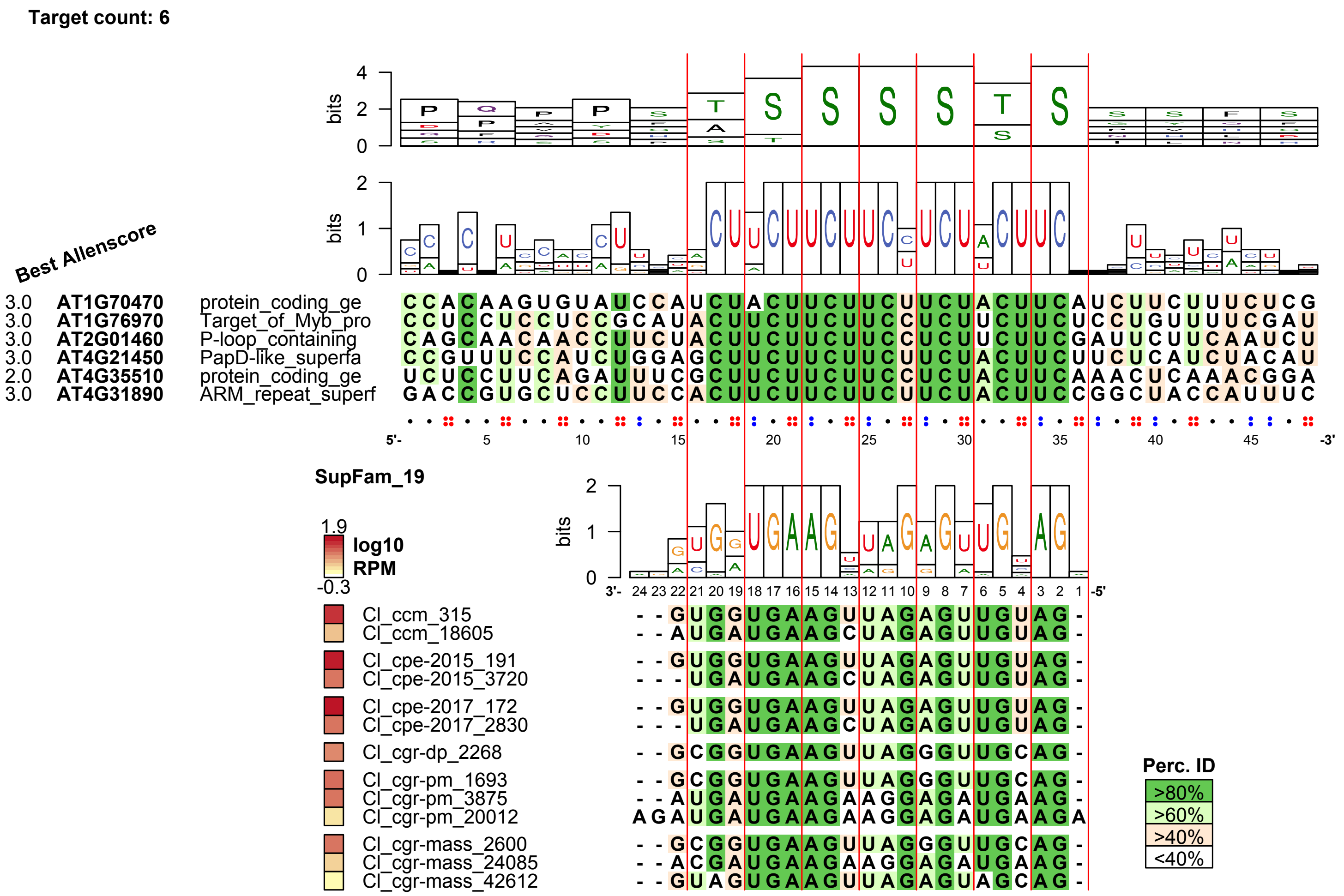
Target count: 6
bits
Best Allenscore
3.0 AT1G70470 protein_coding_ge
3.0 AT1G76970 Target_of_Myb_pro
3.0 AT2G01460 P-loop_containing
3.0 AT4G21450 PapD-like_superfa
2.0 AT4G35510 protein_coding_ge
3.0 AT4G31890 ARM_repeat_superf
5'-
-3'
SupFam_19
1.9
log10
RPM
-0.3
3'-
-5'
Cl_ccm_315
Cl_ccm_18605
Cl_cpe-2015_191
Cl_cpe-2015_3720
Cl_cpe-2017_172
Cl_cpe-2017_2830
Cl_cgr-dp_2268
Cl_cgr-pm_1693
Cl_cgr-pm_3875
Cl_cgr-pm_20012
Cl_cgr-mass_2600
Cl_cgr-mass_24085
Cl_cgr-mass_42612
Perc. ID
>80%
>60%
>40%
<40%
